# The R2R3-MYB transcription factor family in *Taxus chinensis*: identification, characterization, expression profiling and posttranscriptional regulation analysis

**DOI:** 10.7717/peerj.8473

**Published:** 2020-02-17

**Authors:** Xinling Hu, Lisha Zhang, Iain Wilson, Fenjuan Shao, Deyou Qiu

**Affiliations:** 1 State Key Laboratory of Tree Genetics and Breeding, Key Laboratory of Tree Breeding and Cultivation of State Forestry Administration, The Research Institute of Forestry, Chinese Academy of Forestry, Beijing, China; 2 CAS Key Laboratory of Pathogenic Microbiology and Immunology, Institute of Microbiology, Chinese Academy of Sciences, Beijing, China; 3 Agriculture and Food, CSIRO, Canberra, Australia

**Keywords:** MYB, Transcription factor, *Taxus chinensis*

## Abstract

The MYB transcription factor family is one of the largest gene families playing regulatory roles in plant growth and development. The MYB family has been studied in a variety of plant species but has not been reported in *Taxus chinensis*. Here we identified 72 putative R2R3-MYB genes in *T. chinensis* using a comprehensive analysis. Sequence features, conversed domains and motifs were characterized. The phylogenetic analysis showed TcMYBs and AtMYBs were clustered into 36 subgroups, of which 24 subgroups included members from *T. chinensis* and *Arabidopsis thaliana*, while 12 subgroups were specific to one species. This suggests the conservation and specificity in structure and function of plant R2R3-MYBs. The expression of *TcMYBs* in various tissues and different ages of xylem were investigated. Additionally, miRNA-mediated posttranscriptional regulation analysis revealed that *TcMYBs* were the targets of miR858, miR159 and miR828, suggesting the posttranscriptional regulation of MYBs is highly conserved in plants. The results provide a basis for further study the role of *TcMYBs* in the regulation of secondary metabolites of *T. chinensis*.

## Introduction

The MYB transcription factor family is one of the largest transcription factor families in plants, defined by the MYB domain, which is highly conserved at the N-terminus and consists of 1–4 repeats named 1R-, 2R-, 3R- and 4R-MYB (Baldoni, Genga & Cominelli, 2015). The MYB repeat usually is composed of about 52 amino acid residues and each repeated sequence form three *α*-helices. In one of the repeats, the second and third *α*-helices have a hydrophobic core due to three regularly spaced tryptophan or hydrophobic residues forming a helix-turn-helix structure (Baldoni, Genga & Cominelli, 2015). When it combines with a promoter, the third helix acts as a recognition helix to directly contact a particular DNA sequence motif (Dubos et al., 2010; Hou et al., 2018; Ogata et al., 1996). The C-terminus of MYB transcription factor usually contains a transcription activation region rich in acidic amino acids that is responsible for the diverse regulatory activity of the protein (Araki et al., 2004; Daniel, Sugano & Tobin, 2004; Morse et al., 2009).

In plants, MYB transcription factors are usually divided into four categories based on the number of MYB domains contained at the N terminus, of which the R2R3-MYB (2R) subfamily is the largest and contains more than 100 members in many species (Dubos et al., 2010; Hou et al., 2018; Wilkins et al., 2009). Numerous studies have shown that MYB transcription factors are involved in the regulation of a variety of biological processes in plants, including plant growth and development, cell morphogenesis and cell cycle, primary and secondary metabolism, biotic and abiotic stress response and defense (Dubos et al., 2010; Yanhui et al., 2006).

The R2R3-MYB proteins in Arabidopsis are classified into 25 or more subgroups based on phylogenetic relationships and functions (Dubos et al., 2010; Li & Lu, 2014). The correlation between phylogenetic relationship and function has been verified in recent studies. For instance, some R2R3-MYB proteins of subgroup 7 (AtMYB11/PFG1, AtMYB12/PFG1 and AtMYB111/PFG3) and subgroup 6 in *Arabidopsis thaliana* (AtMYB75/PAP1, AtMYB90/PAP2, AtMYB113 and AtMYB114) are involved in the regulation of flavonoid synthesis, while other R2R3-MYB proteins in subgroup 3 (AtMYB58, AtMYB63 and AtMYB85) and subgroup 21 (AtMYB52, AtMYB54 and AtMYB69) are related to the biosynthesis of lignin in the cell wall (Feng et al., 2004; Gonzalez et al., 2008; Stracke et al., 2007; Zhou et al., 2009). In tree species, it has been shown that many R2R3-MYBs play a potential role in wood formation through regulating cell wall component. For example, in *Eucalyptus, EgMYB2* has been shown to regulate the lignin biosynthesis and secondary wall formation in the xylem (Goicoechea et al., 2005). In poplar, *PtrMYB2, PtrMYB3, PtrMYB20* and *PtrMYB* 21 play an important role in the secondary cell wall biosynthesis (Zhang et al., 2014).

*Taxus chinensis* is an important medicinal woody species of the *Taxus* genus. Its bark can produce an active ingredient paclitaxel, which is one of the most effective natural anticancer drugs and widely used to treat numerous cancer, such as breast, ovarian and lung cancer (Wani et al., 1971). In addition, the heartwood of *T. chinensis* has high commercial value for its wood color (purple red brown) and texture density. It has been shown that MYBs may be involved in the regulation of taxol biosynthesis (Li et al., 2012; Wang et al., 2019). Moreover, MYBs also play an important role in the regulation of anthocyanin biosynthesis, that can be used to improve the tissue colors through metabolic genetic engineering approaches (Liu et al., 2018; Naing & Kim, 2018). In order to reveal the possible biological functions and regulatory mechanism of MYB family in the regulation of taxol biosynthesis and the heartwood color formation of *T. chinensis*, it is very important to make a comprehensive analysis of MYB family in *T. chinensis*. In this study, we performed a series of analysis and verifications based on transcriptome data, including the gene family identification, phylogenetic relationship, conserved domains and motifs analysis, expression profiling and miRNA-mediated posttranscriptional regulation of *TcMYBs*. The results provide a basis for the further study of their role in the regulation of secondary metabolites of *T. chinensis*.

## Materials & Methods

### Plant materials

*T. chinensis* plants used in this experiment were grown in the greenhouse of the Chinese Academy of Forestry, Beijing, China. The roots, phloem, xylem and leaf tissue and xylem of one to four years old were collected from ten-year-old plants of *T. chinensis*. The phloem and xylem were collected as described previously (Li et al., 2011). In brief, the bark was peeled from the developing stem, the phloem tissues were scraped from inside of the bark and the xylem tissues were from the peeled log. Each tissue was collected from at least three plants. All samples were stored in liquid nitrogen until RNA extraction.

### Identification of TcMYB genes

The 125 *A. thaliana* R2R3-MYBs protein sequences were downloaded from GenBank at NCBI. The tBLASTn algorithm were used to search for homologues of R2R3-MYB in *A. thaliana* against the assembly of transcriptome in *T. chinensis* (PRJNA580323) with an e-value cut-off of 1e^−10^, then manually examined and corrected the sequence of predicted genes by the BLASTx algorithm.

### Bioinformatic analysis

The molecular weight (Mw) and theoretical isoelectric point (pI) of TcMYBs were performed using the ExPASy server (http://web.expasy.org). MYB domain of TcMYBs were analyzed using the Conserved Domain Database with default parameters (http://www.ncbi.nlm.nih.gov/Structure/cdd/wrpsb.cgi). The amino acid sequences of MYB domain were aligned using clustalW. Sequence logos for R2 and R3 MYB domains were generated using the WebLogo platform (http://weblogo.berkeley.edu/). The neighbor-joining (NJ) Phylogenetic tree for full length TcMYBs and AtMYBs was constructed using MEGA7.0 with 1000 bootstrap replicates (Kumar, Stecher & Tamura, 2016).

### RNA isolation and quantitative real-time reverse transcription-PCR (qRT-PCR)

Total RNA was isolated from the tissues preserved in liquid nitrogen using the RNA extraction kit RN38 (Aidlab Biotech, China). RNA integrity was identifed with a 1.2% agarose gel, and RNA quantity and quality were determined by NanoDrop 1000C Spectro-photometer (Thermo Scientific, USA). The cDNA was obtained by reverse transcription of total RNA using the FastKing RT Kit (With gDNase) KR116 (TIANGEN, China) and used for qRT-PCR by SYBR® rapid quantitative PCR Kit (KAPA KK4601, USA). Gene-specific primers are listed in Table S2 with products lengths between 100 bp and 300 bp. *Tcactin* was used as a reference gene. Relative abundance of *TcMYBs* were calculated according to the comparative Cq method described in previous study (Li & Lu, 2014; Ma et al., 2012). The expression levels of miR159, miR828 and miR858 were analyzed using the method as described previously (Shi & Chiang, 2005). One-way ANOVA was calculated using IBM SPSS 19 software. *P* < 0.01 was considered statistically significant and was represented by asterisks. The heat maps of differential expression of *TcMYB* genes were performed using HemI 1.0 with gradient bar (Clustering Method is Average linkage and Similarity Metric is Pearson distance (default)) (Deng et al., 2014).

### Prediction of *TcMYBs* targeted by miRNA

The conserved miRNAs of *T. chinensis* were obtained from the previous study (Hao et al., 2012; Qiu et al., 2009). Target search of these miRNAs against the identified 72 *TcMYBs* were performed using psRNATarget with default parameters (Dai, Zhuang & Zhao, 2011).

## Results

### Identification of TcMYB genes

BLAST analysis of *A. thaliana* R2R3-MYBs against the assembly of transcriptome in *T. chinensis* was performed using the BLASTx algorithm and a total of 72 *T. chinensis* R2R3-MYB genes were identified. The 72 *TcMYBs* were manually examined according to the alignment between *TcMYBs* and other species R2R3-MYBs using the BLASTx algorithm. The deduced protein sequences of 72 *TcMYBs* shared high sequence identity with known plant R2R3-MYBs and contained the conserved R2R3-MYB domains, suggesting they are authentic R2R3-MYBs. The *TcMYBs* were termed TcMYB1 to TcMYB72, respectively. This may not be a complete set of MYB genes in *T. chinensis*, since the genome assembly of *T. chinensis* is currently not available. Sequence feature analysis of TcMYBs showed that the length of open reading frames (ORFs) ranged from 486 (TcMYB45) to 3,333 bp (TcMYB72) (Table S1). The size of deduced TcMYB proteins varied between 161 and 1,110 amino acids. The molecular weight in TcMYBs varied between 17.31 to 121.78 kDa, and the theoretical pI was from 4.75 to 9.94 (Table S1). The results suggest that there are significant differences in the structure of TcMYB family members that may reflect a diversity of functions in *T. chinensis*.

### Phylogenetic analysis of MYBs in *T. chinensis* and *A. thaliana*

**Figure 1:**
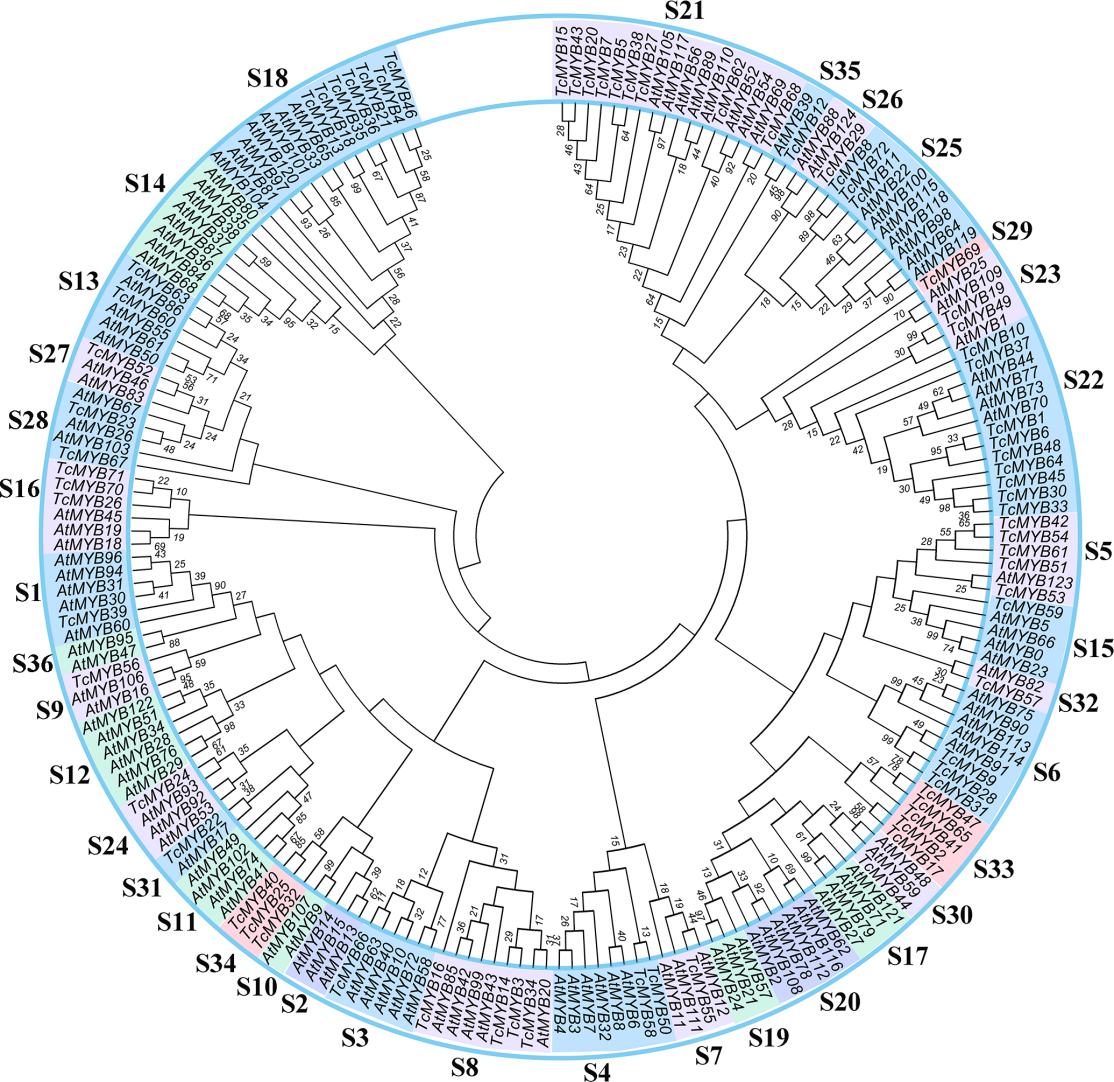
Phylogenetic tree of MYBs from *Taxus chinensis* and *Arabidopsis*. The phylogenetic relationships were constructed using MEGA 7.0 by the Neighbor-Joining (NJ) method (1,000 bootstrap replicates, values <10 are not shown). Thirty-six subgroups were indicated.

In order to analyze the evolutionary relationship of R2R3-MYB family members between *T. chinensis* and *A. thaliana*, a neighbor-joining phylogenetic tree for 72 TcMYBs and 125 AtMYBs proteins were constructed using MEGA7.0 (Fig. 1). The results showed that the R2R3-MYBs in *T. chinensis* and *A. thaliana* can be divided into 36 subgroups (named S1-S36) based on phylogenetic tree and previous studies (Dubos et al., 2010; Li & Lu, 2014). The subgroups were named according to the nomenclature revised by Stracke, Werber & Weisshaar (2001), Dubos et al. (2010) and Li & Lu (2014). S1-S28, S30-31 and S35-S36 were terms based on those pre-existing in *A. thaliana,* while S29 and S32-S34 were novel. 24 subgroups of R2R3-MYBs included members from *T. chinensis* and *A. thaliana,* while 9 subgroups were specific to *A. thaliana* (S2, S10, S11, S12, S14, S17, S19, S20 and S36) and 3 subgroups were specific to *T. chinensis* (S29, S33 and S34).

### Conserved MYB domains analysis

**Figure 2:**
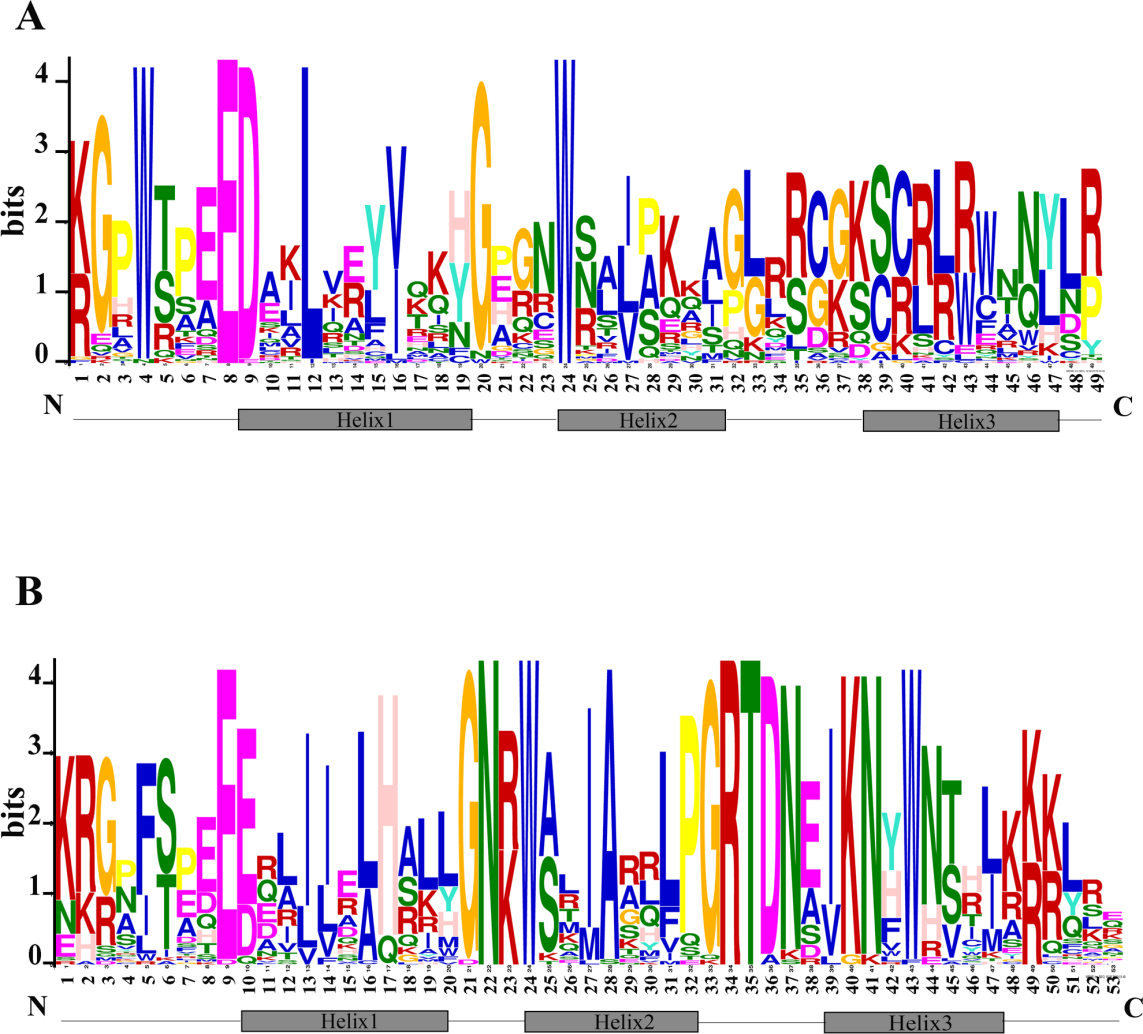
Conserved motifs of R2R3-TcMYBs. (A) Sequence logo of R2 in TcMYBs. (B) Sequence logo of R3 in TcMYBs. The motifs were identified by the MEME package.

The N terminal of MYB protein contains repeated conserved MYB domain (about 52 amino acid residues), and R2R3-MYB belongs to 2R, which includes two such repeats (Dubos et al., 2010). Sequence analysis of MYB domains in 72 TcMYBs showed they contained about 104 amino acid residues, which was the R2R3-MYB domains. With the aim to investigate the conservation of amino acids residues in each MYB domain, the R2 and R3 sequence logos of MYB in *T. chinensis* were analyzed by MEME Suit. The results showed that the R2 sequence in *T. chinensis* contained three tryptophan residues (W), forming a hydrophobic core zin HTH structure (Ogata et al., 1996) (Fig. 2A), and the first tryptophan is replaced by a phenylalanine (F) in R3 sequence (Fig. 2B), which is similar to *A. thaliana* (Li & Lu, 2014; Stracke, Werber & Weisshaar, 2001). It also has been shown that the R2R3-MYB domains usually contained three highly conserved tryptophan residues (W) and the first tryptophan residues (W) of R3 domain was replaced by a phenylalanine (F) residues in other plants, it suggested the R2R3 domains were highly conserved. In *T. chinensis*, the three tryptophan residues are located at 5, 25 and 45 positions of R2 respectively, while they are at 5, 26 and 46 positions in *A. thaliana* (Li & Lu, 2014; Stracke, Werber & Weisshaar, 2001). This may indicate that the divergence of amino acids residues at positions between the first and second helix in R2 sequence. In addition, the C-terminal of R2 sequence contains a conserved LRPD motif, which is consistent with *A. thaliana,* maize and *S. miltiorrhiza* (Du et al., 2012; Li & Lu, 2014; Stracke, Werber & Weisshaar, 2001). Moreover, motif analysis of the MYBs subgroups in *T. chinensis* and *A. thaliana* showed that each subgroup shared at least one motif (Fig. S3).

### Differential expression of TcMYB genes

**Figure 3:**
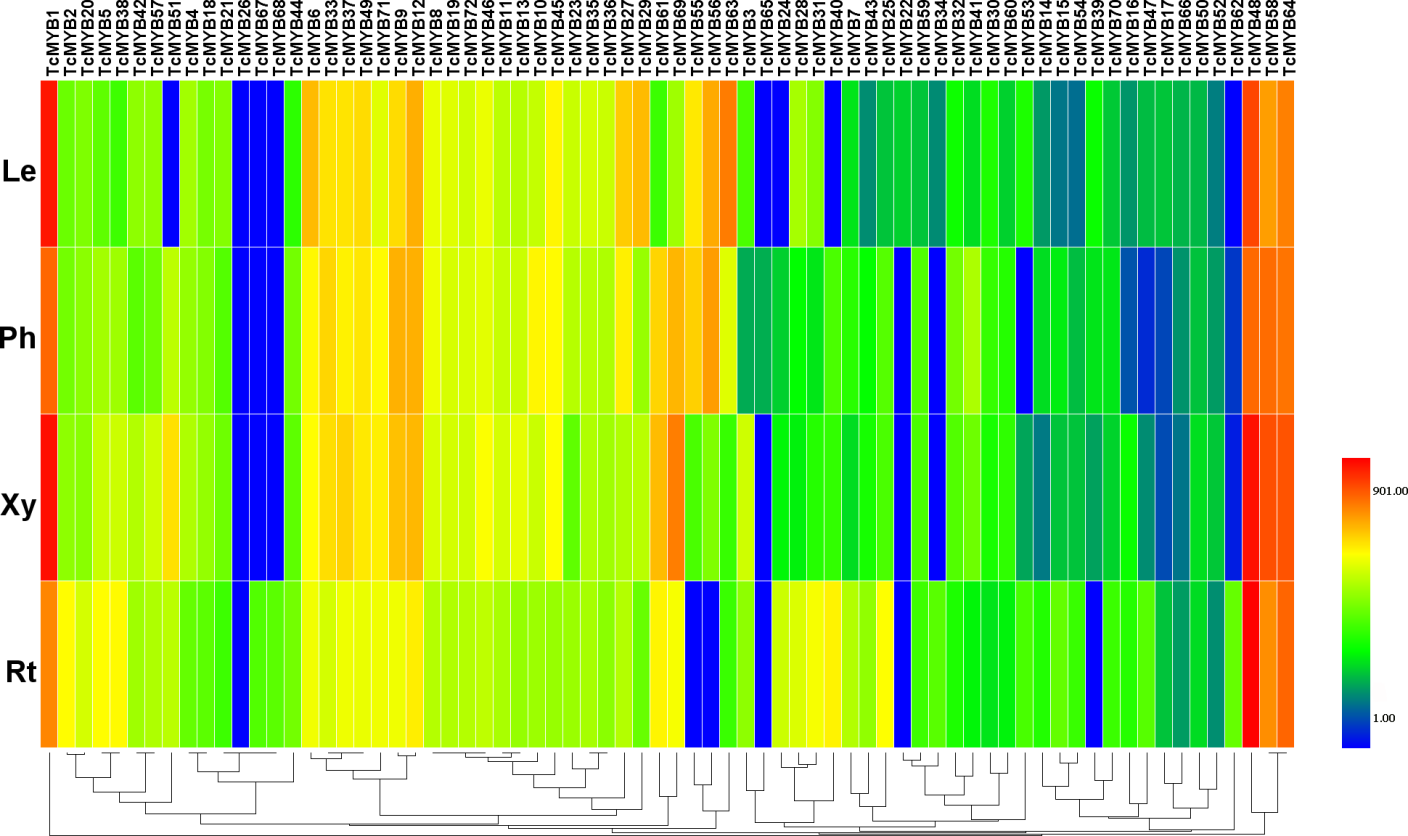
Differential expression of *TcMYB* genes in leaves (Le), phloems (Ph), xylems (Xy) and roots (Rt). The relative expression of *TcMYBs* were compared to *Tcactin* *10000. Statistic analysis using HemI 1.0.

**Figure 4:**
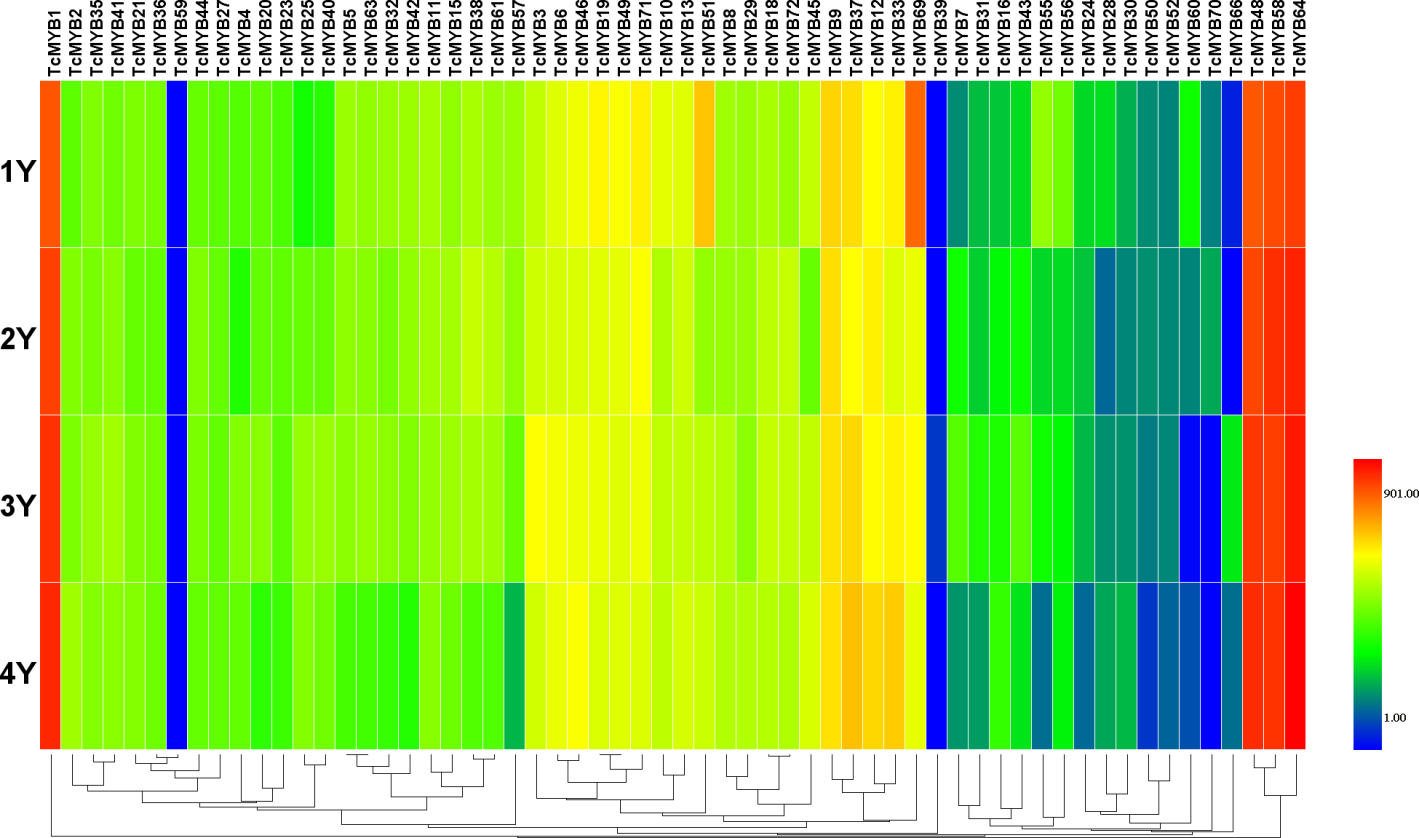
Differential expression of *TcMYB* genes in xylem from 1 to 4 years. The relative expression of *TcMYBs* were compared to *Tcactin* *10000. Statistic analysis using HemI 1.0.

To understand the possible functions of *TcMYBs* in the growth and development of *T. chinensis*, the expression level of 72 *TcMYBs* in leaves, phloem, xylem and roots were analyzed using qRT-PCR. The results showed that *TcMYB1*, *TcMYB48*, *TcMYB58* and *TcMYB64* showed high expression levels in all the analyzed tissues, while the expression levels of *TcMYB26, TcMYB65 and TcMYB66* were low in all tissues (Figs. 3 and 4). The results showed that *TcMYB67*, *TcMYB68, TcMYB34* and *TcMYB62* were only expressed in roots (Fig. 3 and Fig. S1), which indicates that they are root-specific and may play a role in the growth and development or metabolism of roots (Li & Lu, 2014). Whereas, *TcMYB* 39 which was clustered in S1 subgroup was only expressed in leaves. In addition, *TcMYB61*, *TcMYB* 69 and *TcMYB* 60 were highly expressed in xylem and phloem compared with roots and leaves, implying that they may be involved in material transport in *T. chinensis*. *TcMYB* 60 clustered into the S13 subgroup which regulates lignin biosynthesis in *A. thaliana* (Lee, Geisler & Springer, 2009; Newman et al., 2004), and implies a possible similar function in *T. chinensis*. Finally, it was worth noting that *TcMYB9* is highly expressed in the tissues other than root of *T. chinensis*, and is clustered to the S6 subgroup, which was designated as putative woody-expanded subgroup (Soler et al., 2015), suggesting *TcMYB9* may play a role in regulation of woody perennial plants.

### Differential expression of TcMYB genes in different ages of xylem

In order to analyze the possible biological function of R2R3-MYB in xylem development, the relative level of 60 *TcMYBs* expressed in different ages of *T. chinensis* xylem was measured. The results showed that the relative expression levels of most TcMYBs was not significantly different in different ages of xylem, except that *TcMYB51, TcMYB60 and TcMYB69* were more expressed in 1-year-old xylem (Fig. 4 and Fig. S2). TcMYB1, TcMYB48, TcMYB58 and TcMYB64 showed high expression levels in different ages of xylem, which were highly expressed in all tissues, implying that they play an important roles throughout the life of the plant. *TcMYB59* and *TcMYB39* were low in all the xylem from 1 to 4 years old, while *TcMYB35, TcMYB21, TcMYB36, TcMYB4, TcMYB46, TcMYB13* and *TcMYB18* were detected in xylem of all years which clustered in the S18 subgroup. These results provide possible candidate R2R3-MYB genes involved in regulation of xylem development in *T. chinensis*.

### miRNA-mediated posttranscriptional regulation of *TcMYB* genes

**Figure 5:**
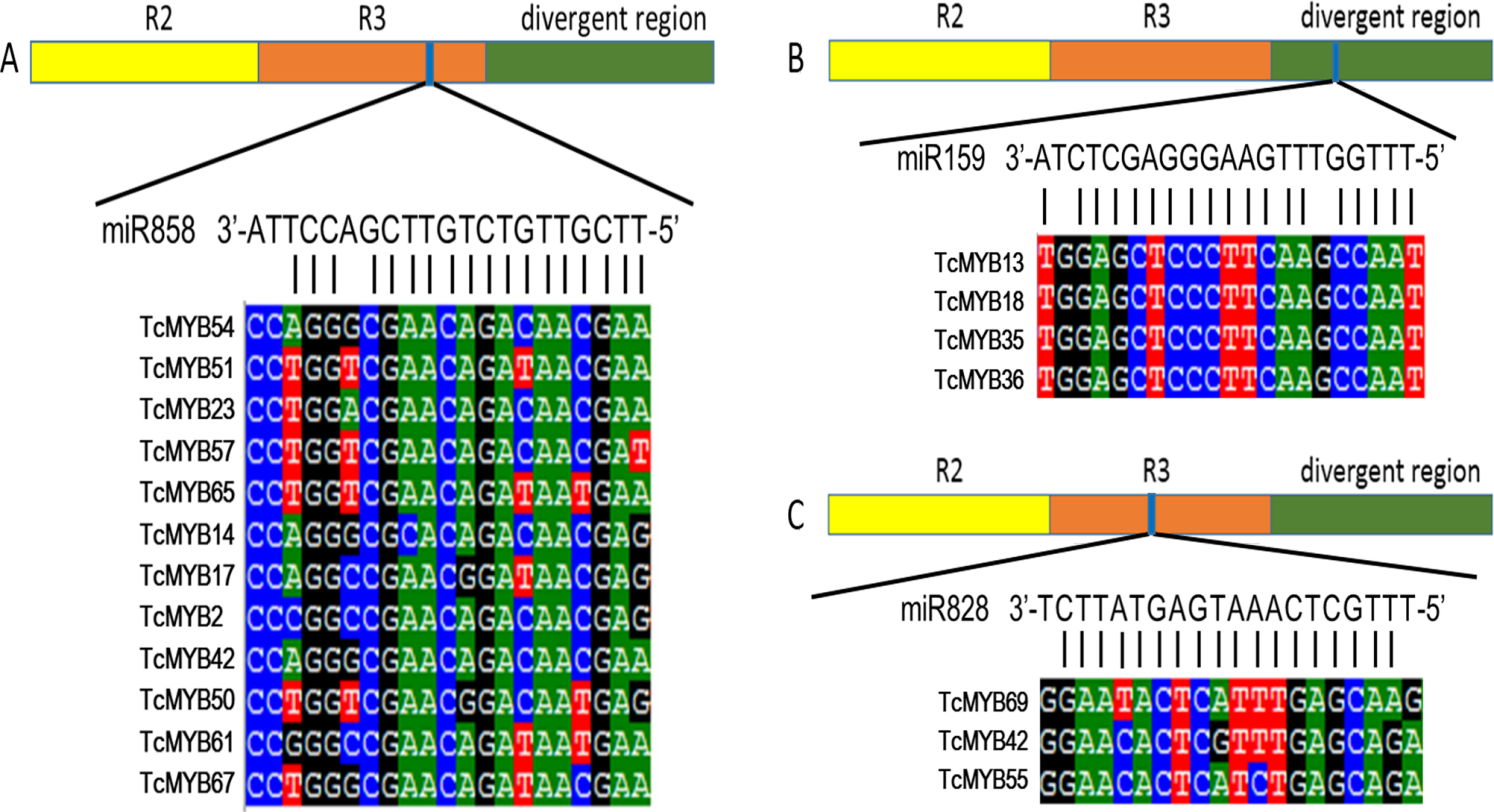
*TcMYBs* targeted by miR858, miR159 and miR828. (A) miR858 targeted sequences of *TcMYBs* and their location. (B) miR159 targeted sequences of *TcMYBs* and their location. (C) miR828 targeted sequences of *TcMYBs* and their location.

**Figure 6:**
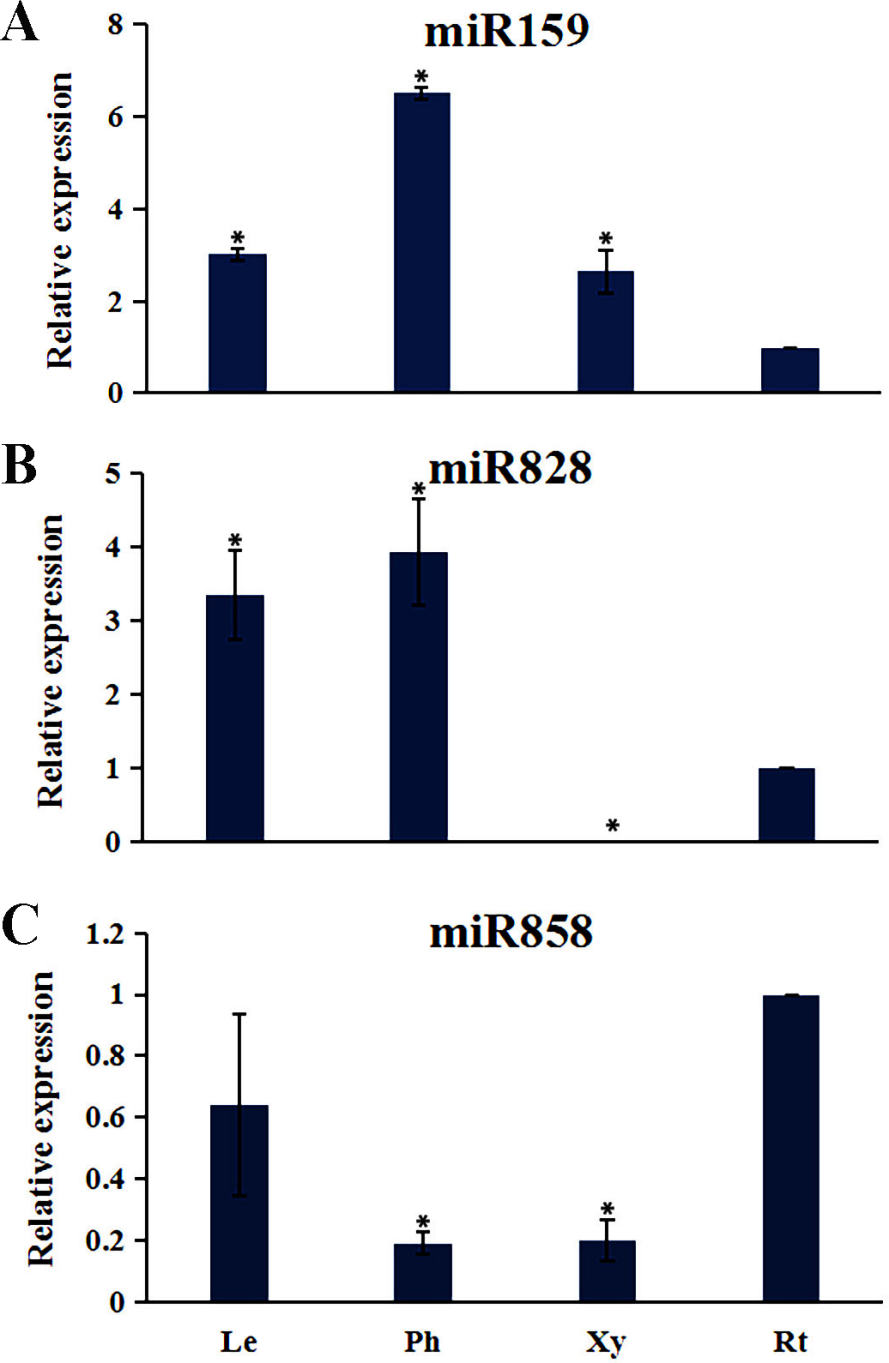
The expression patterns of the miRNAs. (A) The relative expression of miR159 in other tissues (leaves, phloem, and xylem) were compared to roots. (B) The relative expression of miR828 in other tissues (leaves, phloem, and xylem) were compared to roots. (C) The relative expression of miR858 in other tissues (leaves, phloem, and xylem) were compared to roots. One-way ANOVA was calculated using IBM SPSS 19 software. * represented *p* < 0.01.

Plant MYB transcription factors are known to be regulated by miRNA at the post-transcriptional level (Allen et al., 2007; Li & Lu, 2014). In order to investigate miRNA-mediated posttranscriptional regulation of *TcMYBs,* a target search of miRNA against *TcMYBs* were performed using psRNATarget (Dai & Zhao, 2011). The results showed that 18 *TcMYBs* contained potential targets of miR159, miR828 and miR858, with *TcMYB42* possibly targeted by both miR828 and miR858 (Fig. 5). The possible targets of miR159a were *TcMYB13, TcMYB18, TcMYB35 and TcMYB36,* all of which were clustered in the S18 subgroup, that is consistent with previous studies in *A. thaliana* (Allen et al., 2007; Li & Lu, 2014). These genes also showed similar expression patterns in different tissues and different ages of xylem (Figs. 3 and 4). The three targets of miR828 belong to S5, S7 and S29 respectively, including *TcMYB69, TcMYB42 and TcMYB55.* Twelve *TcMYBs* are targeted by miR858 (*TcMYB2, TcMYB14, TcMYB17, TcMYB23, TcMYB42, TcMYB50, TcMYB51, TcMYB54, TcMYB57, TcMYB65, TcMYB61 and TcMYB67*), which were grouped in S4, S5, S8, S28 and S33. The number of possible targets of miR858 is much higher than that of other miRNAs. In addition, the target sites of miR828 and miR858 are located in the R3 domain, whereas the target sites of miR159 is located in a divergent region. This is consistent with the research results of other species (Dubos et al., 2010; Li & Lu, 2014). The results suggested that miRNAs-mediated posttranscriptional regulation of MYBs is highly conserved in plants. In order to investigate the posttranscriptional regulation of miRNAs- mediated *TcMYBs,* miRNA-specific qRT-PCR was performed to examine the expression patterns of miR159, miR828 and miR858 (Fig. 6). The results showed that miR159 and miR858 were expressed in all tissues analyzed, miR828 was not detected in the xylem. MiR159 and miR828 were highly expressed in the phloem. We found that the expression level of some *TcMYB* genes were negatively correlated with the expression level of miR159, miR828 and miR858, for example, *TcMYB69* was highly expressed in xylem and phloem, whereas miR828 showed low expression in xylem and phloem, suggesting the negative regulation between them. Meanwhile, the negative regulation of *TcMYB50* and miR858 was also observed. However, there was little to no negative correlation observed between the expression of some *TcMYB* genes and small RNAs, this may be related to the complex regulatory network of small RNAs.

## Discussion

The MYB transcription factor family is one of the largest gene families in plants playing important roles in plant growth and development. The MYB family has been studied in a variety of plant species, but it has not been reported in *T. chinensis*. In this study, a total of 72 R2R3-MYB genes were identified in *T. chinensis*. The number of MYB genes in plants is significantly higher than that in fungi and animals (Riechmann et al., 2000; Yanhui et al., 2006), and it has been shown that some of the functions of MYB members are redundant (McCarthy, Zhong & Ye, 2009), often due to gene duplication. Previous studies have shown that the numbers of R2R3-MYB genes in *A. thaliana* (Dubos et al., 2010), poplar, grape rice, *Eucalyptus grandis* and *Salvia miltiorrhiza* are 126, 180,123, 106, 141 and 110, respectively (Li & Lu, 2014; Matus, Aquea & Arce-Johnson, 2008; Soler et al., 2015; Wilkins et al., 2009; Yanhui et al., 2006). The number of R2R3-MYB genes identified in *T. chinensis* compared with these species in this study is significantly lower. This maybe due to this study relying on transcriptome data as the assembled genome for *T. chinensis* has not yet been completed. If the number is significantly lower than other plant species, it may suggest that less gene duplication occurred in the evolution of *T. chinensis*.

Based on the phylogenetic tree, 24 subgroups were common between *T. chinensis* and *A. thaliana,* which indicates that these Gymnosperms and Angiosperms R2R3-MYBs were derived from a common ancestor. The specific R2R3-MYBs (9 subgroups and 3 subgroups) represent species specificity and the clustered R2R3-MYBs (24 subgroups) may have similar biological functions (Dubos et al., 2010; Li & Lu, 2014). Phylogenetic analysis of R2R3-MYBs in *T. chinensis* and *A. thaliana* (Fig. 1) indicated that 9 subgroups were specific to *A. thaliana* and three subgroups were specific to *T. chinensis,* these specific subgroups may play an important role in the separation and evolution of these species. In addition, we constructed phylogenetic trees using MYB proteins from *T. chinensis*, *A. thaliana* and *Salvia miltiorrhiza* (Fig. S4). They were divided into 42 subgroups, most of which were clustered similar to Fig. 1. Although the addition of *Salvia miltiorrhiza* changed the topological structure of the tree a little, such as the separation of S6 members, the overall clustering proved the reliability of the analysis in Fig. 1. One MYB belongs to the S5 subgroup in *A. thaliana* (AtMYB123) (Fig. 1), compared with 5 in *T. chinensis* (TcMYB42, TcMYB54, TcMYB61, TcMYB51, TcMYB53) and 16 in *Eucalyptus grandis*. The woody-expanded subgroup and specific subgroups of woody plants may be related to tolerate a more complex living environment (Soler et al., 2015; Wilkins et al., 2009). In plants, the first tryptophan in the R3 repeat of R2R3-MYB is often replaced by phenylalanine, which may be closely related to the evolution of the species. Many amino acid insertions or deletions occur in the first half of each MYB domain, especially in the first helix and adjacent regions (Fig. 2), which may be active regions for evolution and functional differentiation. The relationship between biological function and structural characteristics in MYB family needs to be further explored.

Tissue differential expression analysis showed that *TcMYB1, TcMYB48* and *TcMYB64* were significantly expressed in all the detected tissues of *T. chinensis* and clustered into the S22 subgroup of the phylogenetic tree, implying that they may have important functions in the development of *T. chinensis.* The counterpart *MYB* genes in S22 subgroup of *A. thaliana* are involved in the plant stress response (Jung et al., 2008; Shin et al., 2007), suggesting these *TcMYBs* may also be associated with the stress response in *T. chinensis*. *TcMYB58* was also significantly expressed in all the detected tissues of *T. chinensis*. *TcMYB58 and AtMYB4* clustered into the S4 subgroup of the phylogenetic tree. *AtMYB4* is involved in regulating the metabolism of phenylpropanoid through binding the C4H promoter (Jin et al., 2000), therefore it is possible that *TcMYB58* may be a regulator of phenylpropanoid metabolism in *T. chinensis*.

Taxol a terpenoid, is one of the most potent natural anticancer compounds, but is found in relatively low levels in plants, and is expensive to synthesize (Cragg et al., 1993; Nicolaou et al., 1994). The content of taxol in the bark of *T. chinensis* is remarkably higher than that in other tissues (Wickremesinhe & Arteca, 1994). Understanding the regulatory mechanism of taxol biosynthesis pathway is the basis to improve the yield of taxol in *T. chinensis* by genetic engineering and breeding. Previous studies showed that a number of R2R3-MYBs were involved in regulating the terpenoid biosynthesis. Our results showed that *TcMYB1, TcMYB9, TcMYB12, TcMYB33, TcMYB48 TcMYB56, TcMYB58, TcMYB63, TcMYB69* and *TcMYB64* are highly expressed in pholem (Fig. 3), suggesting these genes may be involved in the regulation of phloem development and taxol biosynthesis. Further study of the function of these *TcMYB* genes in the regulation of taxol biosynthesis will help us investigate the regulatory mechanism of MYBs in taxol biosynthesis.

It has been shown that some *AtMYB* genes were regulated by miR159/319, miR828 and miR858 in *A. thaliana* (Allen et al., 2007; Dubos et al., 2010; Li & Lu, 2014). Analysis of miRNA-mediated *TcMYB* genes post-transcriptional regulation revealed that some specific *TcMYBs* could also be targeted by miR858, miR159 and miR828 (Fig. 5). Both miR858 and miR828 target specific sequences in R3 domain, while miR159 target regions outside the MYB domain. Moreover, miR858 has potentially more *MYB* target genes in *T. chinensis*. These results are similar to other plants, for example *A. thaliana*, apple and *S. miltiorrhiza* (Li & Lu, 2014), suggesting the conservation of miRNA-mediated regulation of MYB genes. Moreover, transcription factors WD40 and bHLH are also involved in the regulation of MYB genes. In plants, MYB, WD40 and bHLH are grouped into the MYB–bHLH–WD40 (MBW) complex, which regulates the structural genes of the anthocyanin biosynthesis pathway. In Arabidopsis, these transcription factors such as TTG1 (WD40 TFs) and TT8, GL1, GL3 and EGL3 (bHLH TFs) are involved in plant flavonoid pigment synthesis and epidermal cell differentiation by regulating R2R3-MYB members (AtMYB75/PAP1, AtMYB90/PAP2, AtMYB113 and AtMYB114), which were clustered in the S6 subgroup (Gonzalez et al., 2008; Ishida et al., 2008; Nadeau, 2009). In this study, TcMYB9, TcMYB28 and TcMYB31 were clustered in the S6 subgroup with counterparts in A. *thaliana* (Fig. 1), implying these TcMYBs are involved in a similar biological process in *T. chinensis*. Our results provide insights into the regulation mechanism of secondary metabolites and the heartwood color formation.

## Conclusions

The R2R3-MYB gene family play an important role in the regulation of plant growth and secondary metabolism. With the aim to reveal the possible biological functions and regulatory mechanism of R2R3-MYB family in the regulation of taxol biosynthesis and the heartwood color formation of *T. chinensis*, a comprehensive analysis of R2R3-MYB family in *T. chinensis* were carried out. In this study, a total of 72 TcMYBs in *T. chinensis* were identified, and these TcMYBs and AtMYBs were clustered into 36 subgroups, of which 24 subgroups included members from *T. chinensis* and *A. thaliana*, while 12 subgroups were specific to one species. The specific MYBs (nine subgroups and three subgroups) represent species specificity and the clustered MYBs (24 subgroups) may have similar biological functions. The *TcMYBs* were differentially expressed in leaves, phloem, xylem, roots and different ages of xylem. Analysis of miRNA-mediated *TcMYB* genes post-transcriptional regulation revealed that 18 *TcMYBs* were targeted by miR858, miR159 and miR828. Our results provide a basis for identifying important candidate *TcMYBs* involved in the regulation of taxol and other secondary metabolites in *T. chinensis.*

##  Supplemental Information

10.7717/peerj.8473/supp-1 Table S1 Sequence features of R2R3-MYBs in *Taxus sp*

10.7717/peerj.8473/supp-2 Table S2 Primers used for qRT-PCR analysis of *TcMYB* genes and miRNAs

10.7717/peerj.8473/supp-3 Table S3 The sequence identity of the 72 TcMYBs with Arabidopsis MYBs

10.7717/peerj.8473/supp-4 Figure S1 Relative expression level of *TcMYB* genes in leaves (Le), phloems (Ph), xylems (Xy) and roots (Rt) The relative expression of TcMYBs were compared to Tcactin *10000.

10.7717/peerj.8473/supp-5 Figure S2 Relative expression level of *TcMYB* genes in xylem from 1 to 4 years The relative expression of *TcMYBs* were compared to *Tcactin* *10000.

10.7717/peerj.8473/supp-6 Figure S3 Motifs of the MYBs subgroups in *T. chinensis and A. thaliana* The motifs were identified by the MEME package.

10.7717/peerj.8473/supp-7 Figure S4 Phylogenetic tree of MYBs from *Taxus chinensis, Arabidopsis* and* Salvia miltiorrhiza* The phylogenetic relationships were constructed using MEGA 7.0 (100 bootstrap replicates, values <10 are not shown).

10.7717/peerj.8473/supp-8 Supplemental Information 1 q-PCR raw data: tissues

10.7717/peerj.8473/supp-9 Supplemental Information 2 q-PCR raw data: years

10.7717/peerj.8473/supp-10 Supplemental Information 3 q-PCR raw data: miRNA

## Additional Information and Declarations

### DNA Deposition

The following information was supplied regarding the deposition of DNA sequences: All new sequences are available in the Supplemental File.

### Data Availability

The following information was supplied regarding data availability: PCR data area available in the Supplemental Files.
