# Supplementary material for: The R2R3-MYB transcription factor family in Taxus chinensis: identification, characterization, expression profiling and posttranscriptional regulation analysis
Source: PeerJ. 2020 Feb 17;8:e8473. doi: 10.7717/peerj.8473 (PMC7032060; doi:10.7717/peerj.8473)
Supplement: Table S2 [file peerj-08-8473-s002.pdf]

Table S2. Primers used for qRT-PCR analysis of *TcMYB* genes and miRNAs

Name	Sequence(5' to 3')
TcActinF	AAGAGAAGCTTGCTTATGTAGC
TcActinR	TCTGATATCCACATCACACTTC
TcMYB1F	TGTCTATGATGTCCGCTGCC
TcMYB1R	GCGATCATTTCCCGCATGAC
TcMYB2F	TCCGTGGGCAGATCAAGATG
TcMYB2R	GCCTCCAGACCAAGACAACT
TcMYB3F	TGAGTAGCAACCAACCCTGT
TcMYB3R	ATGTCGCTCCAACGAATCCA
TcMYB4F	TACCCCTTTACCCACCGGAT
TcMYB4R	GGAGGACCGGACAGAAACTC
TcMYB5F	CGATCCGAGAATCAACCGCA
TcMYB5R	TCGGTTCGACCAGGAAACAA
TcMYB6F	CCGGCAGGAACAGAGCATTA
TcMYB6R	TAGACATAGCGTCCTCCGCT
TcMYB7F	GCCCAGCCAAGTACAGAGAT
TcMYB7R	CAACAAGCTGCACCCACAC
TcMYB8F	CACGTTATGCACCAGCTCCT
TcMYB8R	GAGAGGCCAGGAAGCCATTA
TcMYB9F	TACGCTGGAGCCTGAACTTG
TcMYB9R	CACTGGTGCAGAGGGTACAT
TcMYB10F	GAATCCTGCAACGAAGACATGCCT
TcMYB10R	CACCGTCAGTATCGTGCTGTGGA
TcMYB11F	GAGCAGCTGTGTTGGTTGTG
TcMYB11R	AGAGGCGGATTGAGAGCAAC
TcMYB12F	GGTTGGGCACGACACCTTTA
TcMYB12R	TGGAGTCTGTGCCTCCCTTA
TcMYB13F	TATCCTCTGACAGCCCACCT
TcMYB13R	ATTCCACTCATCCCTTGGGC
TcMYB14F	GTGTAGACCAAGCTGAGACGCA
TcMYB14R	CGGAATTCTCGAAGTAATCTGCT
TcMYB15F	GGTGCAGTTCAACCCAGGAA
TcMYB15R	TCCTGCTTCCATCTGTGACG
TcMYB16F	ATGCCAAGGAAGCATCGACT
TcMYB16R	CCTCCTGGAACACTGAGCTG
TcMYB17F	GATGCATCTCCATACAATCCTGCA
TcMYB17R	GTGTAGCAGATACGAATGGCGTGT
TcMYB18F	GCATTCGCTTTGATGGGCTT
TcMYB18R	GGACGAAACTACCATCCCCG
TcMYB19F	GCCTGAACAGAATGGGTGGA
TcMYB19R	TGCTGTGTCTGAGACGTGAA
TcMYB20F	TTCTCAGCATGGTCCTGGTC
TcMYB20R	GAATCTGTGGTCACCTGGCT
TcMYB21F	GACGGAGGCTTCCATGAGTT
TcMYB21R	ACACCTGCACCAAAGGACAT
TcMYB22F	CATGGTCATGCTGATCATAATCA
TcMYB22R	GGATGATGTCTGACCATGAATTA
TcMYB23F	AAGCATTTGCCTGGACGAAC
TcMYB23R	TGTGATACTTGTGGGCTGGTT
TcMYB24F	GATCATGGCTATGGACCTTCACT
TcMYB24R	CTATTGCTGGTTCGTGATGATA

TcMYB25F	CTTCGCCTGATTCTCCCACC
TcMYB25R	AATCCGCACCAGTGACAGTT
TcMYB26F	TGTGGAAAGAGTTGCAGGCT
TcMYB26R	AGAGTGAAGGCAGATGATGGC
TcMYB27F	GCTTTCAAACACCAGCCAGG
TcMYB27R	TGGTCTTGTCGAGGACATTGG
TcMYB28F	GCTCCTCGCCTTCTGTAACT
TcMYB28R	TTGCCTGTTAGGAGCCGAAT
TcMYB29F	AGGCGCAATGCAGAAACATC
TcMYB29R	CATCTGCTGCTAGGTTGGGA
TcMYB30F	TGGCGCTCACATCTACAGAC
TcMYB30R	AGGCTTTGAGTTGCTGGTCA
TcMYB31F	GCTCCTCGCCTTCTGTAACT
TcMYB31R	TTGCCTGTTAGGAGCCGAAT
TcMYB32F	TCCTATGTCTGCCCATCCAG
TcMYB32R	ATCAGTGCCGTCACAGTCAT
TcMYB33F	ACGGTGGCGCTCATATCTAC
TcMYB33R	GGAGAGGCTTAGACACGTCG
TcMYB34F	GCAGCGTCGAAACGTCAAAT
TcMYB34R	CTCGTTGGATTCCCAGCAGT
TcMYB35F	TGTGATCCCGTCACTCCTCT
TcMYB35R	TCAGTCTTGGTGTTTGCCCC
TcMYB36F	GGGTGGTGGTGGGATTAAGG
TcMYB36R	AGAGGAGTGACGGGATCACA
TcMYB37F	TGCCCAGCAGCAAGTTTTTC
TcMYB37R	TCATCATCAGCATGGCCTCG
TcMYB38F	CCGATCTCAGGGTTCACCAG
TcMYB38R	TGGAGGGATTGTACTGCACC
TcMYB39F	CTGATAGCCAATTGGAAGGCCT
TcMYB39R	CTGATTCATATAACTGAGCTGGT
TcMYB40F	CTTCGCCTGATTCTCCCACC
TcMYB40R	AATCCGCACCAGTGACAGTT
TcMYB41F	GAATTGCGCAGCGTTTACCT
TcMYB41R	GGGCATCGATTTCCTTCGCT
TcMYB42F	TAGGTGGTCACTGATAGCGG
TcMYB42R	TGGATTGGGGACACTGTTTGT
TcMYB43F	GGTGCAGTTCAACCCAGGAA
TcMYB43R	GCTTCCATCTGTGAGGCCAT
TcMYB44F	GGGATCCACACTCTCCTCCA
TcMYB44R	TTGAAGACCAGGCATCCACC
TcMYB45F	GCGTCGTTGAAAAGTGGTGA
TcMYB45R	GATTTCCCCGATCTCCCAGG
TcMYB46F	GGTGGTGGTGGGATTAAGCA
TcMYB46R	ACCCAGAGGAGTGATGGGAT
TcMYB47F	GATGAAGAGGCAAGAACAGAGTT
TcMYB47R	CAGTGAACAGTGATACTGGTCCA
TcMYB48F	GCCGTTATAGTGGAAGCCCA
TcMYB48R	CGTTCAAAGGCAGAAGCGAG
TcMYB49F	GCCTGAACAGAATGGGTGGA
TcMYB49R	TGCTGTGTCTGAGACGTGAA
TcMYB50F	GGCTGAGACAAGCTCATCGT
TcMYB50R	AGTGCAGTACACGATTGGGA
TcMYB51F	CTCGTCGTGAAGAAGCTCGT
TcMYB51R	GTCTGGCACCATGATTGGGA

TcMYB52F	GCCGATGGAATGGGCTATCA
TcMYB52R	TCGAACTCGAACTCGTCACC
TcMYB53F	GTCCATCTGATTCTCCCGTAGAT
TcMYB53R	CATGTCTGCACATACCGCAATT
TcMYB54F	TGTGAGAACAACTGCGCTGA
TcMYB54R	CCACGCATATCTGAGACCGT
TcMYB55F	GATTGCAGGGCTAATGCAGG
TcMYB55R	TGTGGGTCACTGGGTCAATC
TcMYB56F	CACTGTTCCAGCACTCCCTC
TcMYB56R	ATCGAGCAGCATGTCTGTGT
TcMYB57F	CCTGGTCGAACAGACAACGA
TcMYB57R	GCCTTCTCTGTCACCGTACT
TcMYB58F	GCGATCAATGCGACCAGGTA
TcMYB58R	GTCGAGCGTGACAGGTTTCT
TcMYB59F	GTCGACCAGAATCTTGAGAGTA
TcMYB59R	GTTATTCGGCTGCACATCGGTAA
TcMYB60F	GCAACAACATGGAGGTCAACAA
TcMYB60R	CTGTCGTAGGATGGATCAGTTA
TcMYB61F	GTTACAAGAGGACCCCAGCC
TcMYB61R	TGCTGCAGACAATCCCACAT
TcMYB62F	CCAGCCTAATGGCATGTGCT
TcMYB62R	CTTGTCGCATGCTTTGACCC
TcMYB63F	GCCAATCAAGCTGAACACTCG
TcMYB63R	CTGGTGCTTGTTCGAGGAGT
TcMYB64F	TACGCCGTTGTCTGCTCATT
TcMYB64R	CCTGACCTCCTGTTCATCGG
TcMYB65F	GATAGGACACCGCAGAGTAACAT
TcMYB65R	CTCCAATGTATCTGTTCGGTTGA
TcMYB66F	AAGCTCGTCGCCTACATACG
TcMYB66R	TCCACGTTTGAGATCGGGTC
TcMYB67F	CAGAAGCTCTTAATGGAGGTAT
TcMYB67R	CTTCTTCTATCAACGCATGGAGT
TcMYB68F	CACTGGAGACCAGCTGAAGATGA
TcMYB68R	CCATAACAACGTGCCAGTGGTT
TcMYB69F	GTCACACCCACGATAACTCCT
TcMYB69R	ACAATGTTGTTCCTGCTGCAC
TcMYB70F	CGTCCAATTGGTTCAGATGATAGA
TcMYB70R	CTGAGTGATGCGCAATCATATGT
TcMYB71F	AACCGAGGTCGAAGAAGGTG
TcMYB71R	CCCAGGCGTCCAGAATTAGT
TcMYB72F	TGAGGGCAACCATGAAAGCA
TcMYB72R	TTGAGATGCAGGGTAAGCCG
5.8SrRNA	GCGATACTTAGTGTGTGAATTGCA
miR828	TCTTGTTCAAATGAGTATTCT
miR858	TTCGTTGTCTGTTCGACCTTA
miR159	TTTGGTTTGAAGGGAGCTCTA
Reverse primer	GCGAGCACAGAATTAATACGAC
