# Supplementary material for: The R2R3-MYB transcription factor family in Taxus chinensis: identification, characterization, expression profiling and posttranscriptional regulation analysis
Source: PeerJ. 2020 Feb 17;8:e8473. doi: 10.7717/peerj.8473 (PMC7032060; doi:10.7717/peerj.8473)
Supplement: Table S3 [file peerj-08-8473-s003.pdf]

The sequence identity of the 72 TcMYBs with Arabidopsis MYBs

Query	subject	Identities	length	mismatch	gap	Query start	Query stop	subject start	subject stop	Expect	score
TcMYB1	ATMYB44	40.67	359	209	10	31	1095	2	285	7.00E-69	212
TcMYB1	ATMYB77	42.14	318	180	10	31	972	2	285	2.00E-66	206
TcMYB1	ATMYB73	37.78	352	219	7	34	1089	10	283	7.00E-63	197
TcMYB1	AtMYB109	45.07	213	109	5	37	651	54	257	2.00E-54	177
TcMYB1	AtMYB70	74.53	106	27	0	34	351	10	115	2.00E-53	172
TcMYB1	AtMYB1	41.63	233	125	5	34	699	52	262	2.00E-50	167
TcMYB1	ATMYB25	64.08	103	37	0	37	345	48	150	5.00E-45	152
TcMYB1	ATMYB105	48.76	121	62	0	43	405	107	227	4.00E-39	135
TcMYB1	ATMYB54	46.15	130	68	1	43	426	6	135	2.00E-38	131
TcMYB1	ATMYB69	45.52	134	69	1	43	432	19	152	6.00E-38	130
TcMYB1	AtMYB117	54.9	102	46	0	43	348	98	199	1.00E-37	132
TcMYB1	AtMYB56	36.46	192	122	1	43	618	93	278	6.00E-37	129
TcMYB1	ATMYB52	50.5	101	50	0	43	345	5	105	2.00E-36	125
TcMYB1	AtMYB98	56.73	104	45	0	40	351	216	319	3.00E-36	129
TcMYB1	AtMYB64	33.48	230	151	4	40	723	104	320	3.00E-35	127
TcMYB1	ATMYB119	51.33	113	55	0	40	378	104	216	1.00E-34	125
TcMYB1	ATMYB118	46.4	125	65	1	31	399	185	309	8.00E-34	123
TcMYB1	AtMYB115	37.13	167	105	1	43	543	158	318	2.00E-32	118
TcMYB1	ATMYB46	37.22	180	104	4	43	555	20	199	2.00E-32	116
TcMYB1	AtMYB82	36.13	191	111	6	43	582	14	194	4.00E-32	113
TcMYB1	ATMYB61	35.64	202	125	5	43	633	14	206	9.00E-32	116
TcMYB1	ATMYB35	31.05	248	150	6	43	723	14	261	1.00E-31	115
TcMYB1	ATMYB14	43.61	133	73	3	43	435	14	142	1.00E-31	113
TcMYB1	ATMYB121	49.54	109	53	2	43	363	29	136	2.00E-31	113
TcMYB1	ATMYB80	47.71	109	56	1	43	366	14	122	2.00E-31	114
TcMYB1	ATMYB110	47.17	106	56	0	43	360	67	172	2.00E-31	114
TcMYB1	ATMYB72	37.7	183	105	6	43	564	16	188	3.00E-31	113
TcMYB1	AtMYB45	40.12	162	91	4	43	510	20	171	6.00E-31	111
TcMYB1	ATMYB92	51.46	103	48	2	43	345	14	115	7.00E-31	113
TcMYB1	ATMYB95	38.82	152	90	3	43	489	14	164	8.00E-31	111
TcMYB1	ATMYB67	31.14	228	150	6	43	705	24	227	8.00E-31	112
TcMYB1	ATMYB37	43.7	135	74	3	43	441	14	146	1.00E-30	112
TcMYB1	ATMYB58	40.76	157	91	5	43	507	16	161	2.00E-30	110
TcMYB1	ATMYB101	33.19	229	125	5	43	645	20	244	2.00E-30	114
TcMYB1	AtMYB74	42.54	134	76	1	43	441	15	148	2.00E-30	111
TcMYB1	AtMYB41	44.8	125	68	1	43	414	14	138	3.00E-30	110
TcMYB1	AtMYB17	39.1	156	93	4	43	504	14	160	3.00E-30	110
TcMYB1	ATMYB102	32.04	206	139	2	43	657	14	216	3.00E-30	111
TcMYB1	AtMYB36	38.89	144	86	3	43	468	14	151	3.00E-30	111
TcMYB1	ATMYB86	35.35	215	125	6	43	645	14	223	5.00E-30	111
TcMYB1	ATMYB59-3	36.67	150	93	3	43	486	10	146	5.00E-30	108
TcMYB1	AtMYB19	35.27	207	127	6	43	642	14	205	5.00E-30	109
TcMYB1	AtMYB97	53.47	101	46	1	43	342	21	121	6.00E-30	111
TcMYB1	ATMYB63	32.88	222	116	6	43	609	16	236	6.00E-30	109
TcMYB1	ATMYB48	37.76	143	86	3	43	462	9	150	1.00E-29	108
TcMYB1	AtMYB27	39.38	160	90	5	43	501	11	161	1.00E-29	107
TcMYB1	ATMYB13	40.54	148	86	3	43	480	14	159	2.00E-29	107
TcMYB1	ATMYB34	47.71	109	56	1	43	366	14	122	2.00E-29	108
TcMYB1	AtMYB107	49.02	102	51	1	43	345	14	115	3.00E-29	108
TcMYB1	ATMYB106	30.47	256	168	6	43	780	57	283	3.00E-29	109
TcMYB1	ATMYB28	31.71	246	166	6	43	774	14	233	3.00E-29	109
TcMYB1	AtMYB79	46.49	114	59	3	43	378	8	116	3.00E-29	107
TcMYB1	AtMYB51	45.87	109	58	1	43	366	15	123	4.00E-29	108
TcMYB1	AtMYB50	41.61	149	85	4	43	483	14	153	5.00E-29	107
TcMYB1	AtMYB83	48.31	118	60	2	43	393	32	147	5.00E-29	108
TcMYB1	ATMYB26	48.25	114	49	2	43	354	14	127	6.00E-29	108
TcMYB1	ATMYB65	50.98	102	49	1	43	345	43	144	6.00E-29	110
TcMYB1	ATMYB120	29.48	268	182	7	43	825	28	283	7.00E-29	110
TcMYB1	ATMYB3	32.18	202	136	5	25	627	16	199	7.00E-29	105
TcMYB1	AtMYB53	49.51	103	50	2	43	345	14	115	8.00E-29	107
TcMYB1	AtMYB112	35.12	168	106	2	7	501	22	189	1.00E-28	105
TcMYB1	AtMYB60	43.36	113	59	2	43	366	14	122	1.00E-28	105
TcMYB1	ATMYB3	44.35	115	63	1	43	384	14	128	1.00E-28	105
TcMYB1	ATMYB33	50.98	102	49	1	43	345	34	135	2.00E-28	108
TcMYB1	AtMYB9	49.02	102	51	1	43	345	14	115	2.00E-28	106
TcMYB1	AtMYB6	35.29	170	100	4	43	522	14	183	2.00E-28	103
TcMYB1	ATMYB16	37.42	163	101	2	43	528	14	170	3.00E-28	105
TcMYB1	AtMYB49	38.73	142	86	1	43	465	14	155	3.00E-28	105
TcMYB1	ATMYB71	29.7	266	176	9	43	807	20	258	4.00E-28	104
TcMYB1	AtMYB116	44.35	115	63	2	31	372	16	126	5.00E-28	104
TcMYB1	MYB8	44.95	109	59	1	43	366	14	122	5.00E-28	102
TcMYB1	ATMYB15	37.09	151	90	3	43	480	14	163	7.00E-28	103
TcMYB1	ATMYB30	38.36	146	88	3	43	474	14	150	7.00E-28	104
TcMYB1	AtMYB10	41.94	124	65	3	43	393	16	138	1.00E-27	102
TcMYB1	AtMYB10	25.61	82	61	1	43	288	69	149	7.00E-04	33.1
TcMYB1	ATMYB87	46.73	107	55	2	43	357	14	120	1.00E-27	103
TcMYB1	AtMYB103	42.28	123	70	1	43	408	14	136	1.00E-27	104
TcMYB1	ATMYB2	35.63	174	105	5	43	543	22	191	1.00E-27	102

TcMYB1	ATMYB122	46.53	101	53	1	43	342	14	114	2.00E-27	103
TcMYB1	AtMYB40	33.73	166	108	4	43	534	14	169	2.00E-27	102
TcMYB1	AtMYB47	37.5	152	90	3	43	483	14	164	2.00E-27	102
TcMYB1	AtMYB32	40.26	154	90	4	43	498	14	159	2.00E-27	102
TcMYB1	ATMYB66	38.85	139	78	4	43	438	18	154	2.00E-27	100
TcMYB1	AtMYB100	40.71	113	65	1	52	384	29	141	2.00E-27	101
TcMYB1	AtMYB81	48.51	101	51	1	43	342	22	122	3.00E-27	104
TcMYB1	ATMYB55	46.96	115	47	2	43	345	14	127	3.00E-27	103
TcMYB1	AtMYB93	48.04	102	52	1	43	345	14	115	3.00E-27	103
TcMYB1	ATMYB68	48.54	103	51	2	43	345	14	116	4.00E-27	103
TcMYB1	ATMYB84	47.57	103	52	2	43	345	14	116	4.00E-27	102
TcMYB1	ATMYB38	31.53	222	126	5	43	630	14	233	4.00E-27	102
TcMYB1	AtMYB18	40.85	142	80	3	43	456	12	143	4.00E-27	101
TcMYB1	ATMYB96	41.8	122	66	2	43	393	14	131	5.00E-27	102
TcMYB1	AtMYB89	43.43	99	56	0	46	342	57	155	6.00E-27	99
TcMYB1	MYB7	35.48	155	99	2	43	504	14	167	7.00E-27	100
TcMYB1	ATMYB4	45.87	109	58	1	43	366	14	122	8.00E-27	100
TcMYB1	ATMYB123	45.05	111	59	2	19	345	8	117	1.00E-26	99.8
TcMYB1	ATMYB57	30.94	181	124	3	43	582	27	187	2.00E-26	98.2
TcMYB1	ATMYB31	36.69	139	66	3	43	393	14	148	2.00E-26	100
TcMYB1	AtMYB24	44.76	105	57	1	31	342	15	119	2.00E-26	98.2
TcMYB1	AtMYB42	38.81	134	80	2	43	438	14	146	2.00E-26	99.8
TcMYB1	ATMYB23	45.63	103	54	2	43	345	14	115	3.00E-26	97.8
TcMYB1	ATMYB111	36.59	164	102	3	43	528	14	172	4.00E-26	100
TcMYB1	ATMYB94	36.11	144	84	3	43	450	14	153	5.00E-26	99.8
TcMYB1	AtMYB62	44.64	112	60	3	43	372	21	127	6.00E-26	98.6
TcMYB1	ATMYB11	48.04	102	52	1	43	345	14	115	8.00E-26	99.4
TcMYB1	ATMYB0	36.49	148	89	3	43	471	16	158	1.00E-25	96.7
TcMYB1	AtMYB76	32.33	232	152	4	43	723	14	241	1.00E-25	99
TcMYB1	ATMYB29	30.97	226	147	5	43	693	14	220	1.00E-25	98.6
TcMYB1	ATMYB5	42.62	122	62	3	43	384	25	145	2.00E-25	96.7
TcMYB1	AtMYB108	35.48	155	93	4	43	486	21	174	2.00E-25	97.8
TcMYB1	AtMYB114	41.75	103	59	1	43	348	10	112	4.00E-25	92.4
TcMYB1	AtMYB20	44.66	103	55	2	43	345	14	115	7.00E-25	95.5
TcMYB1	AtMYB85	44.66	103	55	2	43	345	14	115	1.00E-24	94.4
TcMYB1	ATMYB12	29.9	194	135	2	43	621	14	205	3.00E-24	95.5
TcMYB1	AtMYB104	44.12	102	55	2	43	342	18	118	5.00E-24	94.7
TcMYB1	AtMYB113	33.99	153	94	4	43	480	10	152	9.00E-24	91.7
TcMYB1	AtMYB43	31.79	173	116	4	43	555	14	177	1.00E-23	92.8
TcMYB1	ATMYB90	40.95	105	61	1	43	354	10	114	1.00E-23	91.3
TcMYB1	ATMYB75	41.12	107	58	2	43	348	10	112	2.00E-23	90.9
TcMYB1	AtMYB22	34.88	129	82	2	4	384	41	168	3.00E-23	90.5
TcMYB1	AtMYB124	31.78	129	88	0	52	438	28	156	4.00E-22	89.7
TcMYB1	AtMYB124	19.88	171	129	5	43	531	77	237	2.00E-04	35
TcMYB1	ATMYB78	31.16	199	111	6	43	561	28	226	5.00E-22	88.2
TcMYB1	ATMYB88	37.86	103	64	0	52	360	33	135	5.00E-22	89.7
TcMYB1	ATMYB88	32.08	53	36	1	43	201	82	133	7.00E-04	33.5
TcMYB1	ATMYB99	39.52	124	63	4	43	378	15	134	2.00E-21	85.1
TcMYB1	ATMYB91	32.71	107	69	1	52	363	7	113	6.00E-16	70.9
TcMYB2	ATMYB13	55.38	130	58	0	31	420	7	136	4.00E-51	161
TcMYB2	ATMYB3	52.08	144	69	1	43	474	19	161	1.00E-50	159
TcMYB2	AtMYB79	60.91	110	43	0	40	369	4	113	4.00E-50	159
TcMYB2	ATMYB48	54.96	131	51	1	49	417	8	138	7.00E-50	159
TcMYB2	ATMYB59-3	54.81	135	61	1	37	441	5	135	7.00E-50	158
TcMYB2	ATMYB71	64.15	106	38	0	40	357	16	121	2.00E-49	158
TcMYB2	AtMYB108	39.18	245	141	6	34	744	15	249	1.00E-48	157
TcMYB2	MYB7	37.85	214	128	2	31	657	7	219	1.00E-48	155
TcMYB2	ATMYB63	55.47	128	57	0	31	414	9	136	2.00E-48	156
TcMYB2	ATMYB67	45.96	161	74	3	1	444	1	161	3.00E-48	156
TcMYB2	ATMYB14	38.4	237	108	5	31	627	7	243	3.00E-48	154
TcMYB2	AtMYB24	62.5	104	39	0	43	354	16	119	3.00E-48	153
TcMYB2	AtMYB85	37.84	222	136	5	31	690	7	219	4.00E-48	154
TcMYB2	ATMYB61	63.64	110	40	0	31	360	7	116	4.00E-48	157
TcMYB2	ATMYB86	47.5	160	82	1	31	504	7	166	4.00E-48	157
TcMYB2	ATMYB57	54.69	128	55	1	46	420	25	152	5.00E-48	152
TcMYB2	AtMYB41	60.91	110	43	0	31	360	7	116	6.00E-48	154
TcMYB2	ATMYB3	40.74	189	110	2	31	591	7	194	6.00E-48	154
TcMYB2	AtMYB42	52.21	136	65	0	31	438	7	142	9.00E-48	154
TcMYB2	AtMYB32	50.71	140	68	1	31	447	7	146	9.00E-48	154
TcMYB2	ATMYB46	55.22	134	60	1	40	441	16	147	1.00E-47	154
TcMYB2	AtMYB112	59.62	104	42	0	43	354	31	134	1.00E-47	152
TcMYB2	ATMYB4	47.83	161	77	2	31	492	7	167	1.00E-47	154
TcMYB2	AtMYB107	61.82	110	42	0	31	360	7	116	2.00E-47	154
TcMYB2	AtMYB50	41.54	195	92	3	31	549	7	201	2.00E-47	154
TcMYB2	ATMYB15	38.33	227	133	4	31	690	7	228	3.00E-47	152
TcMYB2	MYB8	59.09	110	45	0	31	360	7	116	4.00E-47	150
TcMYB2	AtMYB53	62.39	109	41	0	34	360	8	116	5.00E-47	153
TcMYB2	AtMYB9	60.91	110	43	0	31	360	7	116	6.00E-47	153
TcMYB2	ATMYB123	52.21	136	65	0	37	444	11	146	7.00E-47	151
TcMYB2	AtMYB20	60	110	44	0	31	360	7	116	9.00E-47	151

TcMYB2	ATMYB72	42.08	183	106	2	31	579	9	181	9.00E-47	152
TcMYB2	AtMYB74	35.86	290	153	8	31	801	7	286	9.00E-47	152
TcMYB2	AtMYB62	55.75	113	50	0	19	357	10	122	1.00E-46	151
TcMYB2	AtMYB10	49.3	142	72	0	31	456	9	150	2.00E-46	149
TcMYB2	ATMYB92	59.63	109	44	0	34	360	8	116	2.00E-46	152
TcMYB2	AtMYB103	45.09	173	79	4	31	501	7	179	2.00E-46	153
TcMYB2	ATMYB2	62.5	104	39	0	46	357	20	123	2.00E-46	150
TcMYB2	AtMYB83	45.66	173	89	2	34	537	26	195	2.00E-46	152
TcMYB2	ATMYB58	57.27	110	47	0	31	360	9	118	2.00E-46	150
TcMYB2	AtMYB43	60.91	110	43	0	31	360	7	116	3.00E-46	151
TcMYB2	ATMYB5	61.82	110	42	0	31	360	18	127	3.00E-46	149
TcMYB2	ATMYB102	60.91	110	43	0	31	360	7	116	3.00E-46	152
TcMYB2	ATMYB121	38.73	204	125	1	49	660	28	228	4.00E-46	149
TcMYB2	ATMYB106	61.82	110	42	0	31	360	50	159	5.00E-46	152
TcMYB2	AtMYB116	60.19	103	41	0	49	357	19	121	7.00E-46	149
TcMYB2	AtMYB17	60	110	44	0	31	360	7	116	1.00E-45	149
TcMYB2	AtMYB27	58.93	112	46	0	40	375	7	118	2.00E-45	147
TcMYB2	ATMYB16	51.45	138	67	0	31	444	7	144	2.00E-45	149
TcMYB2	AtMYB6	56.36	110	48	0	31	360	7	116	2.00E-45	146
TcMYB2	ATMYB30	44.63	177	94	3	31	549	7	176	4.00E-45	148
TcMYB2	ATMYB12	53.73	134	57	2	31	417	7	139	5.00E-45	149
TcMYB2	ATMYB23	46.04	139	75	0	49	465	13	151	2.00E-44	143
TcMYB2	AtMYB49	56.88	109	47	0	34	360	8	116	4.00E-44	145
TcMYB2	ATMYB101	52.89	121	53	1	46	396	18	138	5.00E-44	149
TcMYB2	ATMYB35	54.55	110	50	0	31	360	7	116	5.00E-44	145
TcMYB2	ATMYB26	48.91	137	61	1	31	414	7	143	5.00E-44	146
TcMYB2	AtMYB93	59.09	110	45	0	31	360	7	116	7.00E-44	146
TcMYB2	ATMYB99	54.7	117	46	1	31	360	8	124	1.00E-43	142
TcMYB2	AtMYB40	42.26	168	88	2	31	507	7	174	1.00E-43	142
TcMYB2	ATMYB66	56.73	104	45	0	49	360	17	120	2.00E-43	140
TcMYB2	ATMYB94	48.59	142	71	3	31	450	7	146	2.00E-43	144
TcMYB2	AtMYB51	61.9	105	40	0	46	360	13	117	2.00E-43	144
TcMYB2	AtMYB60	48.57	140	69	1	31	441	7	146	2.00E-43	142
TcMYB2	ATMYB65	47.86	140	66	1	37	435	38	177	4.00E-43	147
TcMYB2	ATMYB34	55.45	110	49	0	31	360	7	116	4.00E-43	142
TcMYB2	ATMYB87	36.15	260	165	9	31	807	7	233	6.00E-43	142
TcMYB2	ATMYB80	52.21	113	54	0	22	360	4	116	6.00E-43	142
TcMYB2	ATMYB78	55.56	117	38	1	46	354	26	142	6.00E-43	142
TcMYB2	ATMYB122	43.27	171	97	2	31	543	7	168	1.00E-42	142
TcMYB2	AtMYB81	36.94	222	131	6	37	675	17	238	1.00E-42	144
TcMYB2	ATMYB95	49.28	138	69	1	34	444	8	145	1.00E-42	140
TcMYB2	ATMYB11	56.36	110	48	0	31	360	7	116	2.00E-42	142
TcMYB2	ATMYB111	56.36	110	48	0	31	360	7	116	2.00E-42	141
TcMYB2	ATMYB31	42.04	157	91	2	31	501	7	153	3.00E-42	141
TcMYB2	ATMYB55	54.92	122	43	1	31	360	7	128	3.00E-42	141
TcMYB2	AtMYB36	47.37	133	69	1	31	426	7	139	3.00E-42	141
TcMYB2	ATMYB96	40.34	176	98	1	31	537	7	182	6.00E-42	140
TcMYB2	AtMYB82	47.69	130	64	2	31	408	3	131	2.00E-41	135
TcMYB2	AtMYB76	55.45	110	49	0	31	360	7	116	2.00E-41	139
TcMYB2	ATMYB0	51.38	109	53	0	34	360	10	118	3.00E-41	135
TcMYB2	ATMYB28	55.45	110	49	0	31	360	7	116	3.00E-41	139
TcMYB2	ATMYB84	44.76	143	78	1	31	456	7	149	4.00E-41	137
TcMYB2	AtMYB97	51.67	120	52	2	46	387	19	138	5.00E-41	139
TcMYB2	ATMYB29	55.45	110	49	0	31	360	7	116	9.00E-41	137
TcMYB2	AtMYB47	45.39	152	77	2	34	471	8	159	1.00E-40	135
TcMYB2	ATMYB38	44.74	152	78	3	31	468	7	154	3.00E-40	135
TcMYB2	ATMYB33	43.92	148	76	1	34	456	28	175	3.00E-40	139
TcMYB2	ATMYB68	50	122	60	1	31	393	7	128	4.00E-40	136
TcMYB2	ATMYB37	48.78	123	62	1	31	396	7	129	6.00E-40	135
TcMYB2	ATMYB120	56.19	105	46	0	46	360	26	130	7.00E-40	138
TcMYB2	AtMYB19	46.9	145	71	1	37	453	9	153	1.00E-39	132
TcMYB2	AtMYB18	49.25	134	66	1	40	435	8	141	3.00E-39	132
TcMYB2	AtMYB113	41.89	148	86	1	46	489	8	149	1.00E-38	129
TcMYB2	AtMYB114	50.47	107	53	0	46	366	8	114	4.00E-38	124
TcMYB2	AtMYB45	45.71	140	74	1	19	432	9	148	5.00E-38	128
TcMYB2	ATMYB75	50.47	107	53	0	46	366	8	114	3.00E-37	125
TcMYB2	ATMYB90	50.96	104	51	0	46	357	8	111	5.00E-36	122
TcMYB2	ATMYB25	37.21	172	107	4	52	564	50	215	5.00E-31	112
TcMYB2	ATMYB77	34.73	167	100	2	52	525	6	171	1.00E-29	107
TcMYB2	AtMYB109	50.49	103	50	2	52	357	56	156	1.00E-29	108
TcMYB2	AtMYB109	29.69	64	44	1	211	399	56	119	6.00E-05	36.2
TcMYB2	ATMYB119	45.79	107	57	2	40	357	101	205	1.00E-29	108
TcMYB2	ATMYB119	31.34	67	45	1	202	399	102	168	2.00E-07	44.3
TcMYB2	ATMYB73	47.06	102	54	1	52	357	13	113	3.00E-29	106
TcMYB2	ATMYB73	28.12	64	45	1	211	399	13	76	6.00E-05	36.2
TcMYB2	AtMYB98	42.37	118	68	2	4	357	204	317	1.00E-28	106
TcMYB2	AtMYB98	29.73	74	51	1	181	399	207	280	2.00E-08	47.4
TcMYB2	AtMYB70	48.04	102	53	1	52	357	13	113	1.00E-28	104
TcMYB2	ATMYB118	44.64	112	61	2	25	357	180	289	1.00E-28	106
TcMYB2	ATMYB118	26.56	64	46	1	211	399	189	252	5.00E-06	39.7

TcMYB2	AtMYB64	38.76	129	78	2	52	435	105	231	1.00E-28	106
TcMYB2	AtMYB1	51.96	102	49	1	52	357	55	155	2.00E-28	105
TcMYB2	AtMYB1	30.16	63	43	1	196	381	50	112	8.00E-05	35.8
TcMYB2	ATMYB44	35.85	159	96	4	52	510	6	158	5.00E-28	102
TcMYB2	AtMYB104	46.3	108	58	1	37	360	13	119	8.00E-28	103
TcMYB2	ATMYB52	38.55	166	79	5	52	480	5	168	9.00E-27	98.2
TcMYB2	ATMYB54	42.86	126	71	3	52	426	6	127	2.00E-26	97.4
TcMYB2	AtMYB117	44.9	98	53	2	46	336	96	191	4.00E-26	98.6
TcMYB2	AtMYB117	28.57	63	44	1	205	390	96	158	2.00E-04	34.7
TcMYB2	ATMYB105	33.95	162	89	6	37	468	102	259	1.00E-25	96.7
TcMYB2	AtMYB56	37.14	140	79	3	16	408	86	219	2.00E-25	96.3
TcMYB2	ATMYB110	37.14	140	87	4	52	468	67	197	2.00E-25	95.9
TcMYB2	AtMYB115	37.74	106	66	1	37	354	153	257	1.00E-24	94.7
TcMYB2	AtMYB115	23.44	64	48	1	211	399	158	221	6.00E-05	36.2
TcMYB2	AtMYB89	33.59	131	85	2	37	423	51	180	5.00E-23	86.7
TcMYB2	AtMYB124	38.66	119	73	2	61	417	28	138	2.00E-22	89
TcMYB2	AtMYB100	36.04	111	71	2	22	354	19	125	4.00E-22	85.5
TcMYB2	AtMYB100	25.58	43	32	0	271	399	47	89	3.00E-04	33.9
TcMYB2	ATMYB69	37.21	129	74	2	49	414	18	145	6.00E-22	85.1
TcMYB2	ATMYB91	28.96	183	113	4	61	558	7	188	1.00E-21	85.9
TcMYB2	ATMYB88	34.45	119	78	1	61	417	33	150	5.00E-20	82
TcMYB2	AtMYB22	33.33	111	73	3	25	354	45	152	2.00E-19	78.2
TcMYB2	AtMYB22	19.12	68	55	0	196	399	49	116	6.00E-04	32.7
TcMYB3	AtMYB20	69.11	123	35	1	4	363	32	154	3.00E-60	183
TcMYB3	AtMYB43	52	175	75	2	4	501	32	200	2.00E-59	182
TcMYB3	AtMYB42	68.91	119	35	1	4	354	32	150	3.00E-58	178
TcMYB3	ATMYB86	44.16	231	101	5	4	612	32	243	4.00E-58	180
TcMYB3	AtMYB85	48.45	194	81	4	4	528	32	223	2.00E-57	176
TcMYB3	ATMYB61	58.45	142	59	1	4	429	32	165	4.00E-55	172
TcMYB3	ATMYB99	50	190	89	5	7	558	41	206	7.00E-55	168
TcMYB3	AtMYB50	74.75	99	25	0	4	300	32	130	9.00E-55	170
TcMYB3	AtMYB9	53.75	160	64	3	4	453	32	190	6.00E-54	169
TcMYB3	AtMYB53	50	178	71	3	4	483	32	209	1.00E-53	167
TcMYB3	MYB7	63.79	116	42	0	4	351	32	147	5.00E-53	164
TcMYB3	AtMYB17	73.47	98	26	0	4	297	32	129	5.00E-53	165
TcMYB3	AtMYB107	54.49	156	61	3	4	441	32	186	6.00E-53	166
TcMYB3	ATMYB4	71.43	98	28	0	4	297	32	129	5.00E-52	162
TcMYB3	ATMYB92	72.16	97	27	0	4	294	32	128	1.00E-51	162
TcMYB3	AtMYB93	47.57	185	80	4	4	507	32	213	2.00E-51	163
TcMYB3	ATMYB16	66.67	114	38	0	4	345	32	145	2.00E-51	162
TcMYB3	AtMYB6	70.41	98	29	0	4	297	32	129	3.00E-51	159
TcMYB3	ATMYB80	71.72	99	28	0	4	300	32	130	4.00E-51	161
TcMYB3	ATMYB55	52.9	155	61	2	4	432	32	184	5.00E-51	161
TcMYB3	AtMYB74	72.45	98	27	0	1	294	32	129	1.00E-50	160
TcMYB3	AtMYB76	53.21	156	67	3	4	453	32	179	2.00E-50	160
TcMYB3	AtMYB32	62.07	116	44	1	4	351	32	146	2.00E-50	158
TcMYB3	AtMYB40	67.96	103	32	1	4	309	32	134	2.00E-50	157
TcMYB3	ATMYB102	73.2	97	26	0	4	294	32	128	3.00E-50	159
TcMYB3	ATMYB28	48.78	164	84	1	4	495	32	194	1.00E-49	158
TcMYB3	ATMYB3	69.39	98	30	0	4	297	32	129	1.00E-49	155
TcMYB3	ATMYB106	72.92	96	26	0	4	291	75	170	2.00E-49	158
TcMYB3	AtMYB49	74.23	97	25	0	4	294	32	128	2.00E-49	156
TcMYB3	ATMYB35	69	100	31	0	1	300	31	130	3.00E-49	156
TcMYB3	MYB8	68.37	98	31	0	4	297	32	129	6.00E-49	152
TcMYB3	ATMYB67	47.65	170	88	2	4	510	42	210	7.00E-49	155
TcMYB3	AtMYB41	71.13	97	28	0	4	294	32	128	1.00E-48	154
TcMYB3	ATMYB5	65.69	102	35	0	7	312	44	145	3.00E-48	151
TcMYB3	AtMYB51	71.43	98	28	0	4	297	33	130	2.00E-47	152
TcMYB3	ATMYB29	68.69	99	31	0	4	300	32	130	4.00E-47	151
TcMYB3	ATMYB34	65.69	102	35	0	1	306	31	132	4.00E-47	150
TcMYB3	AtMYB103	65.35	101	35	0	4	306	32	132	1.00E-45	148
TcMYB3	ATMYB122	66.33	98	33	0	4	297	32	129	5.00E-44	143
TcMYB3	ATMYB95	46.9	145	77	1	4	438	32	161	2.00E-43	140
TcMYB3	ATMYB13	40.8	201	102	3	1	552	31	218	2.00E-43	139
TcMYB3	ATMYB46	70.45	88	26	0	4	267	38	125	2.00E-43	140
TcMYB3	ATMYB15	47.83	161	83	3	4	483	32	176	6.00E-42	136
TcMYB3	ATMYB14	68.6	86	27	0	1	258	31	116	7.00E-42	135
TcMYB3	AtMYB47	44.9	147	81	2	4	444	32	163	1.00E-41	135
TcMYB3	ATMYB72	70.59	85	25	0	4	258	34	118	1.00E-41	136
TcMYB3	AtMYB83	67.44	86	28	0	4	261	50	135	3.00E-41	136
TcMYB3	AtMYB19	43.82	178	89	5	7	507	33	202	3.00E-41	134
TcMYB3	AtMYB10	65.56	90	31	0	4	273	34	123	8.00E-41	132
TcMYB3	ATMYB101	68.6	86	27	0	4	261	38	123	9.00E-41	137
TcMYB3	ATMYB30	55.86	111	49	1	4	336	32	140	1.00E-40	134
TcMYB3	AtMYB116	52.99	117	55	1	4	354	38	153	1.00E-40	133
TcMYB3	ATMYB63	68.6	86	27	0	7	264	35	120	3.00E-40	132
TcMYB3	ATMYB111	37.93	203	116	3	4	582	32	211	3.00E-40	133
TcMYB3	ATMYB58	68.24	85	27	0	4	258	34	118	4.00E-40	131
TcMYB3	AtMYB82	47.37	133	61	2	4	375	32	161	5.00E-40	129
TcMYB3	ATMYB26	64.89	94	24	1	4	258	32	125	1.00E-39	132

TcMYB3	AtMYB79	67.82	87	28	0	1	261	25	111	1.00E-39	130
TcMYB3	AtMYB62	45.83	144	78	2	4	435	39	171	1.00E-39	130
TcMYB3	ATMYB33	67.44	86	28	0	4	261	52	137	2.00E-39	134
TcMYB3	AtMYB18	43.51	154	83	4	4	453	30	180	2.00E-39	130
TcMYB3	ATMYB94	65.91	88	30	0	4	267	32	119	2.00E-39	131
TcMYB3	ATMYB57	57.29	96	41	0	4	291	45	140	2.00E-39	127
TcMYB3	ATMYB3	43.84	146	82	2	4	441	40	181	2.00E-39	128
TcMYB3	ATMYB31	67.05	88	29	0	4	267	32	119	3.00E-39	130
TcMYB3	ATMYB120	67.82	87	28	0	4	264	46	132	3.00E-39	134
TcMYB3	ATMYB71	66.67	87	29	0	1	261	37	123	4.00E-39	129
TcMYB3	ATMYB12	65.88	85	29	0	4	258	32	116	6.00E-39	130
TcMYB3	ATMYB123	65.88	85	29	0	4	258	34	118	8.00E-39	127
TcMYB3	AtMYB81	62.22	90	34	1	4	273	40	128	1.00E-38	130
TcMYB3	ATMYB96	64.77	88	31	0	4	267	32	119	2.00E-38	129
TcMYB3	AtMYB108	40.8	174	90	3	4	486	39	206	2.00E-38	128
TcMYB3	ATMYB66	63.53	85	31	0	4	258	36	120	3.00E-38	124
TcMYB3	AtMYB60	52.1	119	57	1	4	360	32	146	4.00E-38	126
TcMYB3	ATMYB11	63.53	85	31	0	4	258	32	116	1.00E-37	127
TcMYB3	AtMYB97	63.22	87	32	0	4	264	39	125	2.00E-37	127
TcMYB3	AtMYB36	44.9	147	73	2	13	429	36	177	2.00E-37	125
TcMYB3	ATMYB65	62.79	86	32	0	4	261	61	146	3.00E-37	129
TcMYB3	AtMYB45	38.69	168	98	3	4	492	38	189	4.00E-37	123
TcMYB3	ATMYB23	61.18	85	33	0	4	258	32	116	5.00E-37	122
TcMYB3	ATMYB84	68.29	82	26	0	13	258	36	117	8.00E-37	124
TcMYB3	AtMYB112	57.61	92	39	0	1	276	51	142	9.00E-37	122
TcMYB3	ATMYB37	38.05	205	98	4	13	540	36	233	1.00E-36	124
TcMYB3	AtMYB24	59.55	89	36	0	4	270	37	125	1.00E-36	120
TcMYB3	ATMYB121	37.75	204	108	6	4	558	47	240	1.00E-36	122
TcMYB3	ATMYB68	67.07	82	27	0	13	258	36	117	3.00E-36	123
TcMYB3	ATMYB0	60	85	34	0	4	258	34	118	5.00E-36	119
TcMYB3	AtMYB114	50.94	106	52	1	4	321	28	123	1.00E-35	116
TcMYB3	ATMYB38	53.64	110	50	1	13	339	36	145	1.00E-35	120
TcMYB3	ATMYB2	36.51	189	111	3	1	540	39	226	1.00E-35	119
TcMYB3	ATMYB59-3	59.09	88	36	0	1	264	27	114	3.00E-35	117
TcMYB3	ATMYB87	62.79	86	31	1	4	258	32	117	6.00E-35	119
TcMYB3	ATMYB75	60.71	84	33	0	4	255	28	111	6.00E-35	117
TcMYB3	ATMYB90	60.71	84	33	0	4	255	28	111	6.00E-35	117
TcMYB3	ATMYB48	57.95	88	37	0	1	264	26	113	8.00E-35	117
TcMYB3	AtMYB113	36.46	181	108	3	4	525	28	206	7.00E-34	114
TcMYB3	AtMYB27	60.24	83	33	0	7	255	30	112	6.00E-33	112
TcMYB3	ATMYB78	40	160	82	3	4	441	46	202	8.00E-33	113
TcMYB3	AtMYB104	48.89	90	46	1	4	273	36	124	3.00E-28	102
TcMYB3	ATMYB119	35.19	162	90	7	4	444	123	279	2.00E-23	89.7
TcMYB3	ATMYB119	18.06	144	107	4	100	498	102	233	5.00E-04	32.7
TcMYB3	ATMYB118	45.92	98	50	3	1	285	206	301	6.00E-23	88.2
TcMYB3	ATMYB25	36.13	155	91	4	52	492	83	233	8.00E-23	87.4
TcMYB3	AtMYB64	47.06	85	44	2	4	255	123	205	8.00E-23	87.8
TcMYB3	AtMYB98	51.47	68	33	0	52	255	250	317	1.00E-22	87.4
TcMYB3	AtMYB98	26.98	63	45	1	79	264	207	269	3.00E-05	36.2
TcMYB3	AtMYB100	45.57	79	41	1	52	282	59	137	3.00E-22	84
TcMYB3	AtMYB109	55.88	68	30	0	52	255	89	156	9.00E-22	84.7
TcMYB3	ATMYB73	47.06	85	44	2	4	255	31	113	2.00E-21	83.2
TcMYB3	AtMYB1	54.41	68	31	0	52	255	88	155	2.00E-21	83.6
TcMYB3	AtMYB1	25.51	98	62	2	94	354	50	147	8.00E-05	35
TcMYB3	AtMYB115	36.11	108	67	1	52	369	191	298	5.00E-21	82.4
TcMYB3	ATMYB54	45.45	88	47	2	4	264	24	109	2.00E-20	79.3
TcMYB3	ATMYB44	34.56	136	74	4	52	414	39	174	2.00E-20	80.1
TcMYB3	ATMYB105	40.2	102	61	2	34	339	136	231	3.00E-20	79.7
TcMYB3	AtMYB70	47.06	85	44	2	4	255	31	113	4.00E-20	79.3
TcMYB3	ATMYB52	47.67	86	39	2	34	273	34	117	7.00E-20	77.8
TcMYB3	AtMYB117	46.75	77	41	1	34	264	127	201	8.00E-20	79
TcMYB3	ATMYB77	48.53	68	35	0	52	255	39	106	1.00E-19	77.8
TcMYB3	ATMYB110	46.48	71	38	0	52	264	100	170	1.00E-19	77.8
TcMYB3	AtMYB56	32.31	130	80	1	52	417	126	255	2.00E-19	77.4
TcMYB3	AtMYB22	42.47	73	42	0	52	270	86	158	4.00E-19	75.9
TcMYB3	ATMYB91	28.23	209	123	5	46	591	38	243	1.00E-18	75.9
TcMYB3	ATMYB69	37.23	94	59	0	52	333	52	145	7.00E-18	72.4
TcMYB3	AtMYB124	43.28	67	38	0	61	261	61	127	7.00E-15	65.1
TcMYB3	ATMYB88	43.28	67	38	0	61	261	66	132	1.00E-14	64.7
TcMYB3	AtMYB89	32.47	77	52	0	52	282	89	165	5.00E-14	60.8
TcMYB4	ATMYB33	65.52	174	57	2	43	555	4	174	1.00E-77	235
TcMYB4	ATMYB120	66.87	163	52	2	67	549	9	167	6.00E-74	226
TcMYB4	ATMYB65	69.18	146	45	1	118	555	41	185	2.00E-73	225
TcMYB4	ATMYB101	84.03	119	19	0	118	474	18	136	8.00E-73	222
TcMYB4	AtMYB97	71.32	136	39	1	91	498	10	142	2.00E-69	210
TcMYB4	AtMYB81	56.42	179	70	3	91	603	7	182	9.00E-67	204
TcMYB4	AtMYB17	65.38	104	36	0	118	429	12	115	3.00E-51	161
TcMYB4	ATMYB5	50.74	136	66	2	118	522	23	155	3.00E-51	159
TcMYB4	AtMYB74	51.7	147	68	3	118	549	13	156	4.00E-51	161
TcMYB4	ATMYB102	65.38	104	36	0	118	429	12	115	5.00E-51	162

TcMYB4	AtMYB107	65.38	104	36	0	118	429	12	115	7.00E-51	160
TcMYB4	ATMYB3	63.46	104	38	0	118	429	12	115	1.00E-50	158
TcMYB4	ATMYB66	42.19	192	92	5	79	597	3	192	2.00E-50	156
TcMYB4	AtMYB41	61.54	104	40	0	118	429	12	115	2.00E-50	158
TcMYB4	AtMYB9	62.62	107	40	0	118	438	12	118	6.00E-50	159
TcMYB4	AtMYB6	65.69	102	35	0	124	429	14	115	9.00E-50	155
TcMYB4	MYB8	62.5	104	39	0	118	429	12	115	2.00E-49	154
TcMYB4	ATMYB14	59.63	109	44	0	118	444	12	120	3.00E-49	154
TcMYB4	ATMYB15	44.17	163	91	0	118	606	12	174	3.00E-49	155
TcMYB4	AtMYB79	62.86	105	39	0	121	435	7	111	4.00E-49	154
TcMYB4	ATMYB57	60.19	108	43	0	112	435	23	130	4.00E-49	152
TcMYB4	MYB7	63.46	104	38	0	118	429	12	115	4.00E-49	154
TcMYB4	ATMYB106	51.8	139	66	2	118	531	55	190	1.00E-48	156
TcMYB4	ATMYB28	49.08	163	75	4	103	567	8	166	2.00E-48	155
TcMYB4	ATMYB96	52.94	136	63	1	118	522	12	147	2.00E-48	155
TcMYB4	AtMYB32	54.62	130	58	2	124	510	14	138	2.00E-48	153
TcMYB4	ATMYB4	64.71	102	36	0	124	429	14	115	2.00E-48	153
TcMYB4	ATMYB3	54.4	125	52	1	79	438	2	126	3.00E-48	151
TcMYB4	ATMYB92	63.46	104	38	0	118	429	12	115	3.00E-48	154
TcMYB4	AtMYB53	61.54	104	40	0	118	429	12	115	5.00E-48	153
TcMYB4	AtMYB51	54.07	135	60	2	118	516	13	147	7.00E-48	154
TcMYB4	AtMYB24	54.31	116	53	0	79	426	4	119	9.00E-48	149
TcMYB4	ATMYB71	60.95	105	41	0	121	435	19	123	1.00E-47	151
TcMYB4	ATMYB16	60.58	104	41	0	118	429	12	115	1.00E-47	152
TcMYB4	ATMYB13	48.67	150	65	2	118	531	12	151	1.00E-47	150
TcMYB4	AtMYB49	55.56	126	53	2	118	486	12	137	1.00E-47	152
TcMYB4	ATMYB30	60.38	106	42	0	112	429	10	115	1.00E-47	152
TcMYB4	AtMYB76	47.68	151	64	2	103	510	8	157	1.00E-47	152
TcMYB4	AtMYB50	48.59	142	70	2	118	534	12	153	2.00E-47	151
TcMYB4	ATMYB94	58.88	107	44	0	118	438	12	118	3.00E-47	151
TcMYB4	AtMYB60	61.54	104	40	0	118	429	12	115	4.00E-47	150
TcMYB4	AtMYB93	61.54	104	40	0	118	429	12	115	5.00E-47	152
TcMYB4	ATMYB58	47.1	138	73	0	118	531	14	151	6.00E-47	149
TcMYB4	ATMYB63	58.88	107	44	0	118	438	14	120	7.00E-47	149
TcMYB4	ATMYB29	62.04	108	41	1	103	426	8	114	1.00E-46	150
TcMYB4	ATMYB86	54.33	127	57	2	118	495	12	135	1.00E-46	150
TcMYB4	ATMYB121	51.08	139	59	1	121	510	28	166	1.00E-46	148
TcMYB4	ATMYB31	60.58	104	41	0	118	429	12	115	3.00E-46	149
TcMYB4	ATMYB34	59.81	107	43	0	118	438	12	118	3.00E-46	148
TcMYB4	AtMYB20	53.17	126	57	2	118	489	12	137	4.00E-46	147
TcMYB4	ATMYB23	57.28	103	44	0	121	429	13	115	5.00E-46	145
TcMYB4	ATMYB99	52.55	137	54	4	118	495	13	146	5.00E-46	146
TcMYB4	AtMYB116	50.83	120	59	1	79	438	6	124	9.00E-46	146
TcMYB4	AtMYB43	52.99	134	61	2	118	513	12	145	9.00E-46	147
TcMYB4	AtMYB85	50	140	68	3	118	531	12	150	1.00E-45	145
TcMYB4	ATMYB87	58.49	106	43	1	115	429	11	116	2.00E-45	146
TcMYB4	ATMYB46	57.01	107	46	0	118	438	18	124	2.00E-45	145
TcMYB4	AtMYB62	51.64	122	59	1	73	438	6	125	2.00E-45	145
TcMYB4	ATMYB61	58.88	107	44	0	118	438	12	118	2.00E-45	147
TcMYB4	ATMYB80	56.19	105	46	0	115	429	11	115	4.00E-45	145
TcMYB4	ATMYB67	49.28	138	69	2	106	516	18	152	4.00E-45	145
TcMYB4	AtMYB42	58.65	104	43	0	118	429	12	115	5.00E-45	144
TcMYB4	AtMYB82	52.14	117	53	1	106	447	8	124	6.00E-45	142
TcMYB4	ATMYB111	57.69	104	44	0	118	429	12	115	7.00E-45	145
TcMYB4	ATMYB0	57.28	103	44	0	121	429	15	117	9.00E-45	142
TcMYB4	ATMYB95	57.69	104	44	0	118	429	12	115	2.00E-44	142
TcMYB4	ATMYB72	56.73	104	45	0	118	429	14	117	3.00E-44	143
TcMYB4	AtMYB112	52.83	106	50	0	118	435	32	137	3.00E-44	141
TcMYB4	ATMYB122	45.96	161	73	3	118	558	12	172	3.00E-44	144
TcMYB4	AtMYB108	54.21	107	49	0	118	438	19	125	4.00E-44	143
TcMYB4	ATMYB12	55.77	104	46	0	118	429	12	115	4.00E-44	144
TcMYB4	AtMYB83	41.77	158	86	1	94	549	22	179	6.00E-44	143
TcMYB4	ATMYB59-3	54.87	113	50	1	121	456	9	121	6.00E-44	140
TcMYB4	ATMYB84	44.65	159	73	2	115	546	11	169	7.00E-44	142
TcMYB4	ATMYB2	54.72	106	48	0	118	435	20	125	8.00E-44	141
TcMYB4	AtMYB36	47.22	144	71	3	115	531	11	146	9.00E-44	142
TcMYB4	ATMYB123	54.81	104	47	0	118	429	14	117	1.00E-43	140
TcMYB4	ATMYB35	54.29	105	48	0	115	429	11	115	1.00E-43	142
TcMYB4	ATMYB11	54.81	104	47	0	118	429	12	115	2.00E-43	142
TcMYB4	ATMYB48	53.98	113	51	1	121	456	8	120	2.00E-43	139
TcMYB4	ATMYB55	55.08	118	41	1	118	435	12	129	3.00E-43	141
TcMYB4	ATMYB68	43.29	164	88	2	115	591	11	174	4.00E-43	142
TcMYB4	AtMYB40	48.15	135	67	2	118	513	12	146	5.00E-43	139
TcMYB4	AtMYB10	56.73	104	45	0	118	429	14	117	5.00E-43	138
TcMYB4	AtMYB103	55.14	107	48	0	118	438	12	118	7.00E-43	141
TcMYB4	ATMYB26	52.21	113	45	1	118	429	12	124	8.00E-42	138
TcMYB4	AtMYB47	52.73	110	51	1	118	444	12	121	9.00E-42	135
TcMYB4	ATMYB38	48.12	133	68	2	115	510	11	142	9.00E-42	136
TcMYB4	AtMYB104	52.99	117	55	0	124	474	18	134	1.00E-41	138
TcMYB4	AtMYB114	48.6	107	55	0	109	429	5	111	2.00E-41	131

TcMYB4	ATMYB37	54.29	105	47	1	118	429	12	116	7.00E-41	135
TcMYB4	AtMYB45	43.14	153	78	2	121	552	19	169	2.00E-40	132
TcMYB4	AtMYB27	53.77	106	49	0	115	432	8	113	1.00E-39	129
TcMYB4	ATMYB75	47.66	107	56	0	109	429	5	111	1.00E-39	129
TcMYB4	ATMYB78	46.28	121	51	1	118	438	26	146	4.00E-39	130
TcMYB4	AtMYB113	48.08	104	54	0	118	429	8	111	1.00E-38	127
TcMYB4	AtMYB19	52.29	109	52	0	121	447	13	121	1.00E-38	127
TcMYB4	ATMYB90	47.66	107	56	0	109	429	5	111	3.00E-38	126
TcMYB4	AtMYB18	50.49	103	51	0	121	429	11	113	2.00E-35	120
TcMYB4	AtMYB98	45.93	135	72	4	31	432	195	317	2.00E-34	120
TcMYB4	AtMYB1	44.97	149	69	3	22	429	9	155	2.00E-34	119
TcMYB4	AtMYB70	53.92	102	47	1	124	429	13	113	8.00E-34	116
TcMYB4	ATMYB119	50.46	109	53	3	109	432	100	205	2.00E-33	117
TcMYB4	ATMYB73	51.96	102	49	1	124	429	13	113	3.00E-33	115
TcMYB4	ATMYB118	44.53	137	76	2	115	525	186	320	4.00E-33	116
TcMYB4	AtMYB109	50.44	113	55	2	94	429	46	156	1.00E-32	114
TcMYB4	AtMYB115	43.48	138	76	4	19	426	129	257	2.00E-32	113
TcMYB4	AtMYB64	50	104	51	3	124	432	105	205	9.00E-32	112
TcMYB4	ATMYB77	47.66	107	56	1	124	444	6	111	8.00E-31	108
TcMYB4	ATMYB44	50	102	51	1	124	429	6	106	8.00E-31	108
TcMYB4	AtMYB100	49.07	108	54	2	118	438	24	129	1.00E-30	105
TcMYB4	ATMYB25	49.54	109	54	2	106	429	44	150	2.00E-30	108
TcMYB4	AtMYB117	41.73	139	75	3	55	453	70	206	4.00E-30	107
TcMYB4	ATMYB105	45.22	115	62	2	97	438	98	210	5.00E-30	106
TcMYB4	AtMYB56	48.57	105	54	1	124	438	93	196	6.00E-30	106
TcMYB4	ATMYB52	46.96	115	60	2	124	465	5	117	8.00E-30	104
TcMYB4	ATMYB69	45.87	109	58	2	115	438	16	122	1.00E-29	103
TcMYB4	ATMYB54	36.57	175	109	5	124	642	6	171	1.00E-29	103
TcMYB4	ATMYB110	42.19	128	74	2	55	438	48	170	3.00E-28	101
TcMYB4	AtMYB22	40.3	134	80	3	37	438	30	156	4.00E-27	97.4
TcMYB4	ATMYB91	40.38	104	60	1	133	438	7	110	2.00E-25	94.7
TcMYB4	AtMYB89	36.09	133	82	3	127	516	57	178	1.00E-20	78.6
TcMYB4	AtMYB124	34.65	101	66	1	133	435	28	127	1.00E-18	76.3
TcMYB4	ATMYB88	31.68	101	69	1	133	435	33	132	2.00E-16	70.1
TcMYB5	AtMYB117	73.38	154	33	3	10	447	74	225	4.00E-77	228
TcMYB5	ATMYB105	80	135	23	2	7	399	85	219	2.00E-76	225
TcMYB5	AtMYB56	70.9	134	38	2	4	402	73	204	7.00E-67	200
TcMYB5	ATMYB54	79.82	109	22	0	52	378	3	111	2.00E-66	197
TcMYB5	ATMYB52	77.98	109	24	0	52	378	2	110	3.00E-66	196
TcMYB5	ATMYB110	63.57	140	51	0	46	465	62	201	5.00E-65	195
TcMYB5	ATMYB69	65.62	128	44	1	1	384	1	126	7.00E-61	182
TcMYB5	AtMYB89	61.68	107	41	0	64	384	57	163	4.00E-48	148
TcMYB5	ATMYB44	59.41	101	41	0	61	363	6	106	2.00E-43	140
TcMYB5	AtMYB109	58.65	104	43	0	61	372	56	159	4.00E-43	141
TcMYB5	ATMYB25	54.39	114	52	0	31	372	40	153	4.00E-42	137
TcMYB5	AtMYB70	57.43	101	43	0	61	363	13	113	2.00E-41	134
TcMYB5	ATMYB73	56.44	101	44	0	61	363	13	113	3.00E-41	134
TcMYB5	ATMYB77	56.44	101	44	0	61	363	6	106	7.00E-41	133
TcMYB5	AtMYB1	51.85	108	52	0	61	384	55	162	4.00E-39	130
TcMYB5	ATMYB119	39.26	163	86	2	46	495	100	262	3.00E-34	118
TcMYB5	AtMYB115	40.41	146	82	3	52	474	155	297	3.00E-33	114
TcMYB5	AtMYB64	49.11	112	57	1	61	396	105	213	8.00E-33	114
TcMYB5	AtMYB98	42.62	122	70	0	61	426	217	338	2.00E-32	113
TcMYB5	ATMYB23	45.13	113	59	2	61	390	14	126	2.00E-32	108
TcMYB5	ATMYB67	49.55	111	54	3	37	363	17	125	7.00E-32	109
TcMYB5	ATMYB72	37.95	166	101	4	37	528	9	170	2.00E-31	108
TcMYB5	ATMYB66	47.06	102	53	1	61	363	18	119	2.00E-31	106
TcMYB5	ATMYB118	49.06	106	54	1	61	378	189	291	3.00E-31	110
TcMYB5	AtMYB45	35.53	152	93	3	61	501	20	171	3.00E-31	107
TcMYB5	AtMYB103	45.53	123	66	2	37	402	7	128	4.00E-31	108
TcMYB5	ATMYB0	44.64	112	60	2	61	390	16	127	4.00E-31	105
TcMYB5	ATMYB63	44.83	116	62	3	37	378	9	122	6.00E-31	107
TcMYB5	AtMYB10	44.83	116	62	3	37	378	9	122	2.00E-30	104
TcMYB5	AtMYB18	44.44	108	59	1	61	381	12	119	3.00E-30	105
TcMYB5	AtMYB100	46.46	99	52	2	67	360	28	125	4.00E-30	103
TcMYB5	AtMYB114	43.64	110	61	1	40	366	3	112	4.00E-30	100
TcMYB5	ATMYB15	33.53	167	101	3	37	507	6	172	6.00E-30	104
TcMYB5	AtMYB19	47.12	104	53	2	61	366	14	116	6.00E-30	103
TcMYB5	ATMYB121	40.48	126	74	1	43	417	23	148	9.00E-30	103
TcMYB5	AtMYB82	47.06	102	53	1	61	363	14	115	2.00E-29	100
TcMYB5	AtMYB97	40.88	137	77	2	31	429	11	147	3.00E-29	104
TcMYB5	ATMYB80	43.64	110	61	1	37	363	6	115	3.00E-29	103
TcMYB5	AtMYB51	46.08	102	54	1	61	363	15	116	3.00E-29	103
TcMYB5	AtMYB27	46.6	103	53	2	61	363	11	112	5.00E-29	100
TcMYB5	AtMYB53	49.51	103	50	2	61	363	14	115	5.00E-29	102
TcMYB5	ATMYB58	44.55	110	60	2	37	363	9	117	5.00E-29	101
TcMYB5	ATMYB35	45.45	110	59	2	37	363	7	115	5.00E-29	102
TcMYB5	ATMYB75	43.64	110	61	1	40	366	3	112	6.00E-29	100
TcMYB5	ATMYB65	34.15	164	86	2	34	459	34	197	8.00E-29	104
TcMYB5	AtMYB41	44.35	115	57	3	40	363	2	115	8.00E-29	101

TcMYB5	AtMYB50	38.89	144	86	3	34	459	5	141	1.00E-28	101
TcMYB5	ATMYB122	45.19	104	56	1	37	345	6	109	2.00E-28	101
TcMYB5	ATMYB86	40.8	125	72	2	34	402	5	128	2.00E-28	101
TcMYB5	AtMYB104	41.23	114	66	2	31	369	8	118	3.00E-28	101
TcMYB5	AtMYB6	34.62	156	96	4	40	489	2	151	3.00E-28	98.6
TcMYB5	AtMYB49	46.08	102	54	1	61	363	14	115	4.00E-28	100
TcMYB5	ATMYB34	40.65	123	72	1	37	402	6	128	5.00E-28	99.4
TcMYB5	ATMYB55	40	125	62	2	34	369	5	129	6.00E-28	100
TcMYB5	ATMYB92	48.54	103	51	2	61	363	14	115	7.00E-28	99.8
TcMYB5	AtMYB40	39.85	133	75	3	61	444	14	145	8.00E-28	98.2
TcMYB5	ATMYB33	36.6	153	77	3	61	459	34	186	9.00E-28	101
TcMYB5	AtMYB36	37.76	143	87	2	61	483	14	156	9.00E-28	99.4
TcMYB5	ATMYB61	40.8	125	72	2	34	402	5	128	1.00E-27	99.8
TcMYB5	ATMYB90	39.67	121	72	1	40	399	3	123	1.00E-27	97.4
TcMYB5	ATMYB71	38.93	131	76	3	61	441	20	149	1.00E-27	97.8
TcMYB5	AtMYB81	45.37	108	58	1	49	369	18	125	1.00E-27	100
TcMYB5	ATMYB101	46.67	105	54	2	61	369	20	123	1.00E-27	100
TcMYB5	ATMYB26	40.32	124	64	2	34	375	5	128	1.00E-27	99.4
TcMYB5	AtMYB107	44.55	110	60	2	37	363	7	115	2.00E-27	98.6
TcMYB5	ATMYB46	40.52	116	68	1	34	378	11	126	2.00E-27	97.8
TcMYB5	ATMYB3	41.23	114	61	2	40	363	2	115	3.00E-27	96.7
TcMYB5	MYB8	39.47	114	63	2	40	363	2	115	3.00E-27	95.5
TcMYB5	AtMYB74	41.82	110	63	1	37	363	7	116	3.00E-27	97.8
TcMYB5	ATMYB13	44.35	115	62	3	61	399	14	125	3.00E-27	96.3
TcMYB5	ATMYB13	29.03	62	44	1	61	246	67	127	0.001	31.2
TcMYB5	AtMYB9	42.98	114	59	2	40	363	2	115	4.00E-27	97.8
TcMYB5	ATMYB120	37.23	137	78	2	61	447	28	164	4.00E-27	99.4
TcMYB5	ATMYB59-3	43.4	106	58	2	61	372	10	114	5.00E-27	95.5
TcMYB5	ATMYB48	44.34	106	57	2	61	372	9	113	5.00E-27	95.9
TcMYB5	ATMYB123	36.3	135	85	2	61	462	16	139	6.00E-27	95.9
TcMYB5	ATMYB4	35.57	149	89	4	40	465	2	144	6.00E-27	96.3
TcMYB5	ATMYB95	33.54	161	104	4	61	534	14	162	7.00E-27	95.9
TcMYB5	ATMYB102	42.73	110	62	1	37	363	6	115	8.00E-27	97.1
TcMYB5	ATMYB5	40	115	68	1	61	402	25	139	9.00E-27	95.1
TcMYB5	AtMYB79	38.57	140	68	3	61	426	8	146	2.00E-26	94.7
TcMYB5	AtMYB93	42.98	114	59	2	40	363	2	115	2.00E-26	96.3
TcMYB5	ATMYB37	42.74	117	65	3	37	381	7	122	2.00E-26	95.5
TcMYB5	ATMYB84	44.66	103	55	2	61	363	14	116	3.00E-26	95.1
TcMYB5	AtMYB32	41.23	114	61	2	40	363	2	115	3.00E-26	94.4
TcMYB5	ATMYB14	41.07	112	63	2	37	363	6	115	4.00E-26	93.6
TcMYB5	ATMYB16	41.74	115	60	3	40	363	2	115	5.00E-26	94.7
TcMYB5	ATMYB68	34.62	156	94	4	61	504	14	169	6.00E-26	95.1
TcMYB5	AtMYB113	40.95	105	61	1	61	372	10	114	7.00E-26	92.8
TcMYB5	AtMYB43	40.8	125	72	2	61	429	14	137	9.00E-26	94
TcMYB5	ATMYB28	41.74	115	66	1	61	402	14	128	1.00E-25	94.4
TcMYB5	AtMYB76	37.42	155	91	3	61	507	14	168	1.00E-25	94
TcMYB5	ATMYB106	38.6	114	69	1	25	363	45	158	1.00E-25	94.4
TcMYB5	ATMYB2	46.32	95	50	1	61	342	22	116	1.00E-25	92.4
TcMYB5	AtMYB62	38.89	126	75	2	1	372	1	125	2.00E-25	92.4
TcMYB5	AtMYB17	44.66	103	55	2	61	363	14	115	2.00E-25	92.4
TcMYB5	AtMYB83	41.67	108	62	1	61	381	32	139	3.00E-25	92.8
TcMYB5	MYB7	38.6	114	64	2	40	363	2	115	4.00E-25	91.3
TcMYB5	AtMYB60	40.71	113	66	1	61	396	14	126	5.00E-25	91.3
TcMYB5	AtMYB47	38.24	102	62	1	61	363	14	115	5.00E-25	90.9
TcMYB5	AtMYB112	44.21	95	52	1	61	342	34	128	7.00E-25	90.1
TcMYB5	ATMYB38	43.69	103	56	2	61	363	14	116	8.00E-25	90.9
TcMYB5	ATMYB94	41.9	105	60	1	61	372	14	118	1.00E-24	91.3
TcMYB5	ATMYB29	46.88	96	50	1	61	345	14	109	1.00E-24	91.3
TcMYB5	AtMYB22	41.24	97	57	1	70	360	57	152	1.00E-24	89.7
TcMYB5	AtMYB22	31.11	45	31	0	211	345	52	96	2.00E-04	33.5
TcMYB5	AtMYB116	39.05	105	63	1	61	372	20	124	3.00E-24	89.4
TcMYB5	AtMYB42	41.44	111	63	2	37	363	6	115	3.00E-24	89.4
TcMYB5	ATMYB96	40.95	105	61	1	61	372	14	118	3.00E-24	90.1
TcMYB5	ATMYB88	37.61	109	68	0	70	396	33	141	4.00E-24	90.9
TcMYB5	AtMYB108	32.19	146	98	2	1	435	8	146	4.00E-24	89.4
TcMYB5	ATMYB30	42.16	102	58	1	61	363	14	115	6.00E-24	89
TcMYB5	AtMYB20	42.45	106	59	2	61	372	14	118	9.00E-24	87.8
TcMYB5	ATMYB31	42.16	102	58	1	61	363	14	115	1.00E-23	88.2
TcMYB5	ATMYB57	40.74	108	62	2	61	378	27	133	1.00E-23	85.9
TcMYB5	AtMYB85	43.69	103	56	2	61	363	14	115	3.00E-23	86.3
TcMYB5	ATMYB87	36.94	111	68	2	37	363	6	116	3.00E-23	86.7
TcMYB5	ATMYB3	30.52	154	106	2	61	519	22	174	4.00E-23	85.1
TcMYB5	AtMYB124	37.86	103	64	0	70	378	28	130	4.00E-23	87.8
TcMYB5	ATMYB111	40.2	102	60	1	61	363	14	115	5.00E-23	86.7
TcMYB5	AtMYB24	41.05	95	55	1	61	342	19	113	6.00E-23	84.3
TcMYB5	ATMYB91	34.15	123	78	1	61	420	4	126	7.00E-23	86.7
TcMYB5	ATMYB12	39.22	102	61	1	61	363	14	115	3.00E-22	84.7
TcMYB5	ATMYB11	39.22	102	61	1	61	363	14	115	4.00E-22	84.3
TcMYB5	ATMYB78	31.43	140	81	2	61	435	28	167	1.00E-20	79.7
TcMYB5	ATMYB99	36.76	136	77	4	61	441	15	144	2.00E-20	78.6

TcMYB6	ATMYB77	46.47	269	139	9	1	792	1	235	1.00E-65	201
TcMYB6	ATMYB44	45.45	253	137	4	1	756	1	208	6.00E-63	194
TcMYB6	ATMYB73	43.6	250	141	3	7	756	10	211	1.00E-57	181
TcMYB6	AtMYB70	73.83	107	28	0	7	327	10	116	1.00E-54	173
TcMYB6	AtMYB109	66.67	114	38	1	10	351	54	163	4.00E-52	169
TcMYB6	AtMYB1	54.23	142	65	1	7	432	52	192	8.00E-50	163
TcMYB6	ATMYB25	65.05	103	36	0	10	318	48	150	2.00E-46	153
TcMYB6	ATMYB105	49.59	121	61	0	16	378	107	227	3.00E-40	136
TcMYB6	ATMYB54	55.45	101	45	0	16	318	6	106	1.00E-39	132
TcMYB6	ATMYB69	40.11	177	106	2	16	546	19	188	6.00E-39	130
TcMYB6	AtMYB117	55.88	102	45	0	16	321	98	199	6.00E-39	133
TcMYB6	ATMYB52	50.5	101	50	0	16	318	5	105	3.00E-37	126
TcMYB6	AtMYB98	56.73	104	45	0	13	324	216	319	6.00E-37	129
TcMYB6	AtMYB56	53.47	101	47	0	16	318	93	193	3.00E-36	125
TcMYB6	ATMYB119	51.33	113	55	0	13	351	104	216	4.00E-35	124
TcMYB6	ATMYB118	51.4	107	52	0	4	324	185	291	3.00E-34	122
TcMYB6	AtMYB64	49.56	113	57	0	13	351	104	216	9.00E-34	120
TcMYB6	ATMYB80	48.62	109	55	1	16	339	14	122	7.00E-33	116
TcMYB6	ATMYB110	42.86	133	65	1	16	381	67	199	1.00E-32	115
TcMYB6	AtMYB36	36.47	170	106	4	16	519	14	173	2.00E-32	115
TcMYB6	AtMYB115	48.54	103	53	0	16	324	158	260	2.00E-32	116
TcMYB6	AtMYB82	40	155	91	4	16	474	14	155	3.00E-32	112
TcMYB6	ATMYB14	43.61	133	73	3	16	408	14	142	3.00E-32	113
TcMYB6	ATMYB121	48.62	109	54	2	16	336	29	136	2.00E-31	112
TcMYB6	AtMYB45	41.67	156	88	5	16	474	20	168	2.00E-31	111
TcMYB6	ATMYB46	40.67	150	87	2	16	459	20	169	2.00E-31	112
TcMYB6	ATMYB92	51.46	103	48	2	16	318	14	115	2.00E-31	113
TcMYB6	ATMYB67	31.75	252	167	8	16	756	24	233	2.00E-31	112
TcMYB6	ATMYB61	42.25	142	80	3	16	435	14	151	2.00E-31	113
TcMYB6	AtMYB17	38.32	167	101	5	16	510	14	170	5.00E-31	111
TcMYB6	ATMYB37	42.25	142	80	3	16	435	14	153	6.00E-31	111
TcMYB6	ATMYB13	37.29	177	109	4	16	540	14	176	6.00E-31	109
TcMYB6	ATMYB58	43.36	143	79	4	16	438	16	152	8.00E-31	110
TcMYB6	AtMYB41	44.8	125	68	1	16	387	14	138	9.00E-31	110
TcMYB6	AtMYB74	42.54	134	76	1	16	414	15	148	1.00E-30	110
TcMYB6	ATMYB102	41.13	141	82	1	16	435	14	154	1.00E-30	111
TcMYB6	ATMYB59-3	37.33	150	92	3	16	459	10	146	1.00E-30	108
TcMYB6	ATMYB35	36.6	153	95	2	16	468	14	166	1.00E-30	110
TcMYB6	ATMYB95	38.16	152	91	3	16	462	14	164	1.00E-30	109
TcMYB6	AtMYB51	33.86	189	123	4	16	576	15	198	2.00E-30	110
TcMYB6	AtMYB27	41.26	143	77	4	16	423	11	150	3.00E-30	107
TcMYB6	ATMYB101	49.02	102	51	1	16	318	20	121	3.00E-30	112
TcMYB6	AtMYB79	34.41	186	118	5	16	561	8	178	3.00E-30	108
TcMYB6	ATMYB72	50.49	103	49	2	16	318	16	117	3.00E-30	108
TcMYB6	AtMYB107	49.02	102	51	1	16	318	14	115	7.00E-30	108
TcMYB6	AtMYB97	52.48	101	47	1	16	315	21	121	7.00E-30	109
TcMYB6	ATMYB48	34.15	164	104	3	16	495	9	171	8.00E-30	107
TcMYB6	ATMYB34	47.71	109	56	1	16	339	14	122	8.00E-30	107
TcMYB6	ATMYB65	33.8	216	138	4	16	648	43	243	1.00E-29	110
TcMYB6	AtMYB50	36.65	191	110	5	16	555	14	203	1.00E-29	107
TcMYB6	AtMYB60	37.09	151	88	3	16	447	14	160	1.00E-29	107
TcMYB6	AtMYB83	48.31	118	60	2	16	366	32	147	2.00E-29	107
TcMYB6	AtMYB53	49.51	103	50	2	16	318	14	115	2.00E-29	107
TcMYB6	ATMYB63	46.9	113	55	3	16	339	16	127	2.00E-29	106
TcMYB6	AtMYB49	39.44	142	85	1	16	438	14	155	2.00E-29	107
TcMYB6	ATMYB26	48.65	111	47	2	16	318	14	124	3.00E-29	107
TcMYB6	AtMYB47	35.06	174	101	3	16	501	14	186	4.00E-29	105
TcMYB6	ATMYB16	36.99	173	108	2	16	531	14	180	4.00E-29	106
TcMYB6	AtMYB32	40.91	154	89	4	16	471	14	159	4.00E-29	105
TcMYB6	ATMYB33	50.98	102	49	1	16	318	34	135	6.00E-29	108
TcMYB6	AtMYB9	49.02	102	51	1	16	318	14	115	6.00E-29	106
TcMYB6	AtMYB19	41.43	140	80	3	16	429	14	144	7.00E-29	104
TcMYB6	AtMYB103	38.89	144	87	1	16	444	14	157	7.00E-29	106
TcMYB6	ATMYB86	41.26	143	80	3	16	432	14	155	8.00E-29	106
TcMYB6	ATMYB15	37.75	151	89	3	16	453	14	163	1.00E-28	104
TcMYB6	ATMYB3	43.48	115	64	1	16	357	14	128	2.00E-28	103
TcMYB6	AtMYB100	31.32	182	123	3	25	564	29	206	2.00E-28	102
TcMYB6	ATMYB87	48.54	103	51	2	16	318	14	116	2.00E-28	104
TcMYB6	MYB8	47.06	102	53	1	16	318	14	115	2.00E-28	102
TcMYB6	AtMYB116	44.35	115	63	2	4	345	16	126	3.00E-28	103
TcMYB6	ATMYB71	45.63	103	54	2	16	318	20	121	3.00E-28	103
TcMYB6	ATMYB68	49.51	103	50	2	16	318	14	116	3.00E-28	105
TcMYB6	ATMYB84	48.54	103	51	2	16	318	14	116	3.00E-28	103
TcMYB6	ATMYB106	48.04	102	52	1	16	318	57	158	3.00E-28	105
TcMYB6	ATMYB3	37.68	138	85	3	4	414	18	152	4.00E-28	101
TcMYB6	ATMYB122	46.53	101	53	1	16	315	14	114	4.00E-28	103
TcMYB6	AtMYB89	44.44	99	55	0	19	315	57	155	5.00E-28	100
TcMYB6	ATMYB30	38.26	149	90	3	16	456	14	153	5.00E-28	103
TcMYB6	ATMYB28	40.56	143	82	3	16	435	14	156	7.00E-28	103
TcMYB6	AtMYB81	48.51	101	51	1	16	315	22	122	7.00E-28	104

TcMYB6	ATMYB55	46.96	115	47	2	16	318	14	127	7.00E-28	103
TcMYB6	AtMYB10	41.13	124	66	3	16	366	16	138	8.00E-28	101
TcMYB6	AtMYB93	48.04	102	52	1	16	318	14	115	9.00E-28	103
TcMYB6	ATMYB96	33.16	187	120	4	16	561	14	184	1.00E-27	102
TcMYB6	ATMYB2	44.74	114	61	2	16	351	22	134	1.00E-27	101
TcMYB6	ATMYB66	38.85	139	78	4	16	411	18	154	2.00E-27	99.4
TcMYB6	AtMYB18	47.57	103	53	1	16	321	12	114	2.00E-27	101
TcMYB6	AtMYB6	47.06	102	53	1	16	318	14	115	2.00E-27	100
TcMYB6	ATMYB120	47.52	101	52	1	16	315	28	128	3.00E-27	103
TcMYB6	AtMYB40	35.9	156	98	4	16	477	14	159	3.00E-27	100
TcMYB6	ATMYB4	48.04	102	52	1	16	318	14	115	3.00E-27	100
TcMYB6	ATMYB94	36.81	144	83	3	16	423	14	153	4.00E-27	101
TcMYB6	AtMYB112	35.26	156	98	2	16	474	34	189	4.00E-27	99.4
TcMYB6	ATMYB38	47.57	103	52	2	16	318	14	116	5.00E-27	100
TcMYB6	AtMYB42	33.52	176	115	3	16	537	14	180	5.00E-27	100
TcMYB6	ATMYB123	46.6	103	53	2	16	318	16	117	6.00E-27	99.4
TcMYB6	AtMYB24	44.76	105	57	1	4	315	15	119	6.00E-27	98.2
TcMYB6	ATMYB31	43.4	106	55	2	16	318	14	115	7.00E-27	100
TcMYB6	MYB7	44.04	109	60	1	16	339	14	122	1.00E-26	99
TcMYB6	ATMYB57	40.71	113	66	1	16	351	27	139	1.00E-26	97.4
TcMYB6	ATMYB0	32.43	185	124	3	16	567	16	194	1.00E-26	97.8
TcMYB6	ATMYB23	45.63	103	54	2	16	318	14	115	1.00E-26	97.4
TcMYB6	ATMYB11	46.67	105	55	1	16	327	14	118	3.00E-26	99
TcMYB6	ATMYB5	36.97	165	88	5	16	462	25	183	3.00E-26	97.1
TcMYB6	AtMYB62	44.64	112	60	3	16	345	21	127	4.00E-26	97.8
TcMYB6	ATMYB111	36.59	164	102	3	16	501	14	172	4.00E-26	98.6
TcMYB6	AtMYB108	37.68	138	82	3	16	417	21	157	6.00E-26	97.8
TcMYB6	AtMYB76	44.04	109	60	1	16	339	14	122	8.00E-26	97.8
TcMYB6	AtMYB114	41.75	103	59	1	16	321	10	112	1.00E-25	92.8
TcMYB6	ATMYB29	46.53	101	53	1	16	315	14	114	1.00E-25	97.1
TcMYB6	AtMYB20	44.66	103	55	2	16	318	14	115	2.00E-25	95.5
TcMYB6	ATMYB90	33.16	187	119	5	16	558	10	193	2.00E-25	94.7
TcMYB6	AtMYB104	44.86	107	57	3	16	330	18	122	3.00E-25	96.7
TcMYB6	AtMYB85	44.66	103	55	2	16	318	14	115	5.00E-25	94.4
TcMYB6	AtMYB22	30.32	188	129	4	16	573	54	231	5.00E-25	94
TcMYB6	AtMYB43	31.09	193	131	4	16	588	14	195	2.00E-24	94
TcMYB6	ATMYB12	44.12	102	56	1	16	318	14	115	2.00E-24	94.4
TcMYB6	ATMYB75	41.12	107	58	2	16	321	10	112	5.00E-24	91.3
TcMYB6	AtMYB113	40.19	107	59	2	16	321	10	112	9.00E-24	90.5
TcMYB6	ATMYB88	31.82	176	111	3	25	525	33	205	5.00E-23	91.3
TcMYB6	ATMYB88	32.08	53	36	1	16	174	82	133	5.00E-04	33.5
TcMYB6	AtMYB124	31.3	131	90	0	25	417	28	158	1.00E-22	90.1
TcMYB6	AtMYB124	32.08	53	36	1	16	174	77	128	5.00E-04	33.5
TcMYB6	ATMYB78	28.78	205	120	4	16	552	28	232	2.00E-22	88.2
TcMYB6	ATMYB99	41.59	113	54	4	16	318	15	123	1.00E-21	84.7
TcMYB6	ATMYB91	32.71	107	69	1	25	336	7	113	4.00E-16	70.5
TcMYB7	ATMYB105	87.65	81	10	0	64	306	139	219	4.00E-49	155
TcMYB7	AtMYB117	86.42	81	11	0	64	306	130	210	8.00E-49	155
TcMYB7	AtMYB56	60	115	46	0	40	384	117	231	1.00E-44	143
TcMYB7	ATMYB54	62.39	109	41	1	64	390	38	144	9.00E-44	139
TcMYB7	ATMYB52	75.95	79	19	0	64	300	37	115	6.00E-43	137
TcMYB7	ATMYB110	75	80	20	0	64	303	99	178	7.00E-42	135
TcMYB7	ATMYB69	76.39	72	17	0	64	279	51	122	3.00E-38	125
TcMYB7	AtMYB89	68.12	69	22	0	79	285	93	161	9.00E-32	106
TcMYB7	AtMYB109	56	75	33	0	64	288	88	162	1.00E-28	102
TcMYB7	ATMYB25	57.53	73	31	0	64	282	82	154	7.00E-28	100
TcMYB7	ATMYB66	33.77	151	86	4	73	483	54	203	9.00E-28	96.7
TcMYB7	AtMYB1	57.14	70	30	0	64	273	87	156	3.00E-27	99
TcMYB7	AtMYB1	32.31	65	43	2	58	249	42	97	2.00E-04	33.1
TcMYB7	ATMYB44	59.42	69	28	0	64	270	38	106	9.00E-27	96.3
TcMYB7	AtMYB70	59.42	69	28	0	64	270	45	113	9.00E-27	96.3
TcMYB7	ATMYB77	57.97	69	29	0	64	270	38	106	6.00E-26	94
TcMYB7	ATMYB73	55.07	69	31	0	64	270	45	113	1.00E-25	93.6
TcMYB7	ATMYB23	54.55	66	30	0	73	270	50	115	1.00E-25	91.3
TcMYB7	ATMYB0	54.55	66	30	0	73	270	52	117	2.00E-25	91.3
TcMYB7	ATMYB15	37.06	143	74	4	73	453	50	187	2.00E-24	89.7
TcMYB7	AtMYB27	49.35	77	39	1	70	300	46	120	1.00E-23	86.7
TcMYB7	ATMYB13	40.87	115	60	3	73	393	50	164	1.00E-23	86.7
TcMYB7	AtMYB10	52.11	71	34	0	73	285	52	122	2.00E-23	86.3
TcMYB7	AtMYB19	52.24	67	32	0	73	273	50	116	2.00E-23	86.7
TcMYB7	ATMYB58	38	100	62	0	73	372	52	151	2.00E-23	86.7
TcMYB7	AtMYB103	45.56	90	49	0	43	312	40	129	3.00E-23	87.8
TcMYB7	AtMYB32	42.55	94	54	0	73	354	50	143	4.00E-23	85.9
TcMYB7	AtMYB104	45.45	88	48	0	79	342	56	143	5.00E-23	87
TcMYB7	ATMYB5	51.43	70	34	0	73	282	61	130	6.00E-23	85.1
TcMYB7	ATMYB3	54.55	66	30	0	73	270	50	115	7.00E-23	85.1
TcMYB7	ATMYB67	54.29	70	32	0	73	282	60	129	1.00E-22	85.5
TcMYB7	ATMYB4	54.55	66	30	0	73	270	50	115	1.00E-22	85.1
TcMYB7	AtMYB45	34.13	126	74	2	73	423	56	181	1.00E-22	84.7
TcMYB7	ATMYB63	50.7	71	35	0	73	285	52	122	1.00E-22	85.1

TcMYB7	AtMYB18	38.14	97	60	0	73	363	48	144	3.00E-22	84
TcMYB7	AtMYB51	54.55	66	30	0	73	270	51	116	3.00E-22	84.7
TcMYB7	MYB8	53.03	66	31	0	73	270	50	115	3.00E-22	82.4
TcMYB7	AtMYB40	51.47	68	33	0	73	276	50	117	4.00E-22	83.2
TcMYB7	ATMYB121	41.35	104	56	1	61	357	61	164	5.00E-22	83.2
TcMYB7	ATMYB95	51.52	66	32	0	73	270	50	115	6.00E-22	82.8
TcMYB7	AtMYB114	51.52	66	32	0	76	273	47	112	7.00E-22	79.7
TcMYB7	AtMYB9	54.29	70	32	0	61	270	46	115	7.00E-22	83.6
TcMYB7	MYB7	51.52	66	32	0	73	270	50	115	8.00E-22	82.4
TcMYB7	ATMYB119	50.57	87	38	2	43	288	131	216	9.00E-22	84
TcMYB7	ATMYB119	36.59	41	25	1	133	252	108	148	3.00E-04	33.1
TcMYB7	ATMYB59-3	39.29	112	68	1	61	396	42	152	9.00E-22	81.6
TcMYB7	AtMYB6	51.52	66	32	0	73	270	50	115	9.00E-22	81.6
TcMYB7	AtMYB81	50	78	39	0	61	294	54	131	1.00E-21	83.6
TcMYB7	ATMYB122	56.67	60	26	0	73	252	50	109	1.00E-21	82.8
TcMYB7	ATMYB48	46.25	80	43	0	43	282	35	114	1.00E-21	81.6
TcMYB7	AtMYB97	36.75	117	74	1	40	390	46	159	1.00E-21	83.2
TcMYB7	AtMYB82	46.75	77	41	0	40	270	39	115	2.00E-21	80.1
TcMYB7	AtMYB53	52.86	70	33	0	61	270	46	115	2.00E-21	82
TcMYB7	ATMYB90	39.29	112	65	1	76	402	47	158	2.00E-21	80.9
TcMYB7	ATMYB55	35.71	126	62	3	73	393	62	185	3.00E-21	82
TcMYB7	ATMYB35	40	100	60	1	61	360	46	143	4.00E-21	81.3
TcMYB7	AtMYB107	52.86	70	33	0	61	270	46	115	6.00E-21	80.9
TcMYB7	AtMYB64	44.55	110	61	2	43	372	131	227	6.00E-21	81.6
TcMYB7	ATMYB14	46.15	78	42	0	73	306	50	127	6.00E-21	79.7
TcMYB7	AtMYB83	45.83	72	39	0	73	288	68	139	7.00E-21	80.9
TcMYB7	ATMYB46	39.76	83	50	0	40	288	45	127	7.00E-21	80.1
TcMYB7	AtMYB41	51.52	66	32	0	73	270	50	115	7.00E-21	80.1
TcMYB7	AtMYB79	39.39	99	60	0	40	336	33	131	7.00E-21	79.7
TcMYB7	AtMYB49	53.03	66	31	0	73	270	50	115	8.00E-21	80.5
TcMYB7	ATMYB34	48.57	70	36	0	73	282	50	119	8.00E-21	80.1
TcMYB7	ATMYB28	50	70	35	0	73	282	50	119	1.00E-20	80.5
TcMYB7	ATMYB71	41.86	86	50	0	73	330	56	141	1.00E-20	79.3
TcMYB7	ATMYB123	39.45	109	66	3	73	399	52	141	1.00E-20	79
TcMYB7	AtMYB98	43.02	86	49	0	64	321	249	334	1.00E-20	80.5
TcMYB7	AtMYB98	36.59	41	25	1	133	252	220	260	3.00E-04	32.7
TcMYB7	ATMYB118	47.3	74	39	0	64	285	221	294	1.00E-20	80.5
TcMYB7	ATMYB118	20.87	115	67	4	133	405	192	306	5.00E-04	32.3
TcMYB7	ATMYB61	37.96	108	66	2	73	393	50	151	1.00E-20	80.1
TcMYB7	AtMYB43	42.05	88	51	0	73	336	50	137	2.00E-20	79.7
TcMYB7	ATMYB75	51.52	66	32	0	76	273	47	112	2.00E-20	78.6
TcMYB7	AtMYB17	51.52	66	32	0	73	270	50	115	2.00E-20	79.3
TcMYB7	ATMYB65	44.87	78	43	0	61	294	75	152	2.00E-20	80.5
TcMYB7	AtMYB100	45.71	70	38	0	58	267	56	125	2.00E-20	78.2
TcMYB7	ATMYB72	45.83	72	39	0	73	288	52	123	2.00E-20	79
TcMYB7	ATMYB101	44.58	83	46	1	73	321	56	134	2.00E-20	80.1
TcMYB7	ATMYB33	38.54	96	59	0	73	360	70	165	2.00E-20	80.1
TcMYB7	AtMYB115	46.48	71	38	0	55	267	187	257	3.00E-20	79.3
TcMYB7	AtMYB47	46.97	66	35	0	73	270	50	115	3.00E-20	78.2
TcMYB7	ATMYB86	45.71	70	38	0	73	282	50	119	5.00E-20	78.6
TcMYB7	AtMYB74	50	66	33	0	73	270	51	116	5.00E-20	78.2
TcMYB7	AtMYB36	38.26	115	71	3	37	381	39	142	6.00E-20	78.2
TcMYB7	AtMYB113	47.83	69	36	0	76	282	47	115	6.00E-20	77
TcMYB7	ATMYB92	50	70	35	0	61	270	46	115	6.00E-20	78.2
TcMYB7	AtMYB116	41.86	86	50	0	70	327	55	140	7.00E-20	77.4
TcMYB7	ATMYB120	44.87	78	43	0	61	294	60	137	8.00E-20	78.6
TcMYB7	ATMYB3	42.86	84	48	1	40	291	47	129	8.00E-20	76.3
TcMYB7	ATMYB2	48.57	70	36	0	40	249	47	116	8.00E-20	77
TcMYB7	ATMYB57	29.2	137	97	2	73	483	63	188	1.00E-19	75.5
TcMYB7	AtMYB76	48.05	77	40	0	40	270	39	115	1.00E-19	77.4
TcMYB7	ATMYB111	40.38	104	62	1	73	384	50	151	1.00E-19	77.4
TcMYB7	ATMYB102	51.52	66	32	0	73	270	50	115	1.00E-19	77.4
TcMYB7	AtMYB62	34.78	115	65	2	73	387	57	171	2.00E-19	76.3
TcMYB7	ATMYB30	38.39	112	69	2	46	381	42	146	2.00E-19	76.6
TcMYB7	ATMYB68	34.75	118	74	2	37	381	39	152	2.00E-19	77
TcMYB7	ATMYB99	51.52	66	32	0	73	270	58	123	3.00E-19	75.1
TcMYB7	AtMYB93	50	70	35	0	61	270	46	115	4.00E-19	76.3
TcMYB7	AtMYB42	48.48	66	34	0	73	270	50	115	4.00E-19	75.5
TcMYB7	ATMYB94	47.89	71	37	0	76	288	51	121	4.00E-19	75.9
TcMYB7	AtMYB50	46.97	66	35	0	73	270	50	115	5.00E-19	75.5
TcMYB7	ATMYB80	48.48	66	34	0	73	270	50	115	5.00E-19	75.5
TcMYB7	ATMYB84	41.03	78	46	0	37	270	39	116	6.00E-19	75.1
TcMYB7	ATMYB106	44.16	77	43	0	40	270	82	158	7.00E-19	75.5
TcMYB7	AtMYB85	48.48	66	34	0	73	270	50	115	8.00E-19	74.3
TcMYB7	ATMYB29	55	60	27	0	73	252	50	109	8.00E-19	75.1
TcMYB7	ATMYB11	39	100	49	1	73	336	50	149	8.00E-19	75.1
TcMYB7	AtMYB60	38.94	113	60	3	46	357	42	153	9.00E-19	74.3
TcMYB7	AtMYB20	46.38	69	37	0	73	279	50	118	9.00E-19	74.3
TcMYB7	ATMYB26	48.48	66	34	0	73	270	59	124	9.00E-19	75.1
TcMYB7	ATMYB96	41.24	97	55	1	76	360	51	147	1.00E-18	74.7

TcMYB7	ATMYB12	48.48	66	34	0	73	270	50	115	1.00E-18	74.7
TcMYB7	AtMYB24	44.29	70	39	0	40	249	44	113	1.00E-18	72.8
TcMYB7	AtMYB108	47.14	70	37	0	40	249	46	115	2.00E-18	73.9
TcMYB7	AtMYB112	45.71	70	38	0	40	249	59	128	2.00E-18	72.8
TcMYB7	ATMYB87	37.04	81	51	0	28	270	36	116	2.00E-18	73.6
TcMYB7	ATMYB16	42.86	77	44	0	40	270	39	115	4.00E-18	73.2
TcMYB7	ATMYB37	43.06	72	41	0	73	288	51	122	5.00E-18	72.8
TcMYB7	ATMYB31	46.67	75	40	1	46	270	42	115	5.00E-18	72.8
TcMYB7	ATMYB38	45.45	66	36	0	73	270	51	116	2.00E-17	70.9
TcMYB7	AtMYB22	35.8	81	52	0	58	300	83	163	2.00E-16	67.4
TcMYB7	AtMYB22	33.33	45	30	0	118	252	52	96	4.00E-05	35.4
TcMYB7	ATMYB78	42.47	73	28	1	73	249	64	136	7.00E-15	63.9
TcMYB7	ATMYB88	35.71	70	45	0	76	285	66	135	1.00E-13	60.5
TcMYB7	AtMYB124	34.29	70	46	0	76	285	61	130	6.00E-13	58.5
TcMYB7	ATMYB91	37.18	78	49	0	76	309	43	120	1.00E-12	57.8
TcMYB8	AtMYB98	49.64	139	69	1	457	870	181	319	1.00E-45	162
TcMYB8	AtMYB98	37.89	95	59	1	406	690	217	310	4.00E-18	80.1
TcMYB8	ATMYB119	59.09	110	45	0	535	864	96	205	2.00E-42	153
TcMYB8	ATMYB119	33.68	95	63	1	406	690	105	198	7.00E-15	70.1
TcMYB8	ATMYB119	35.29	51	32	1	718	867	105	155	2.00E-04	36.6
TcMYB8	ATMYB118	59.62	104	42	0	553	864	186	289	3.00E-41	150
TcMYB8	ATMYB118	34.74	95	62	1	406	690	189	282	1.00E-15	72.8
TcMYB8	ATMYB118	33.96	53	34	1	712	867	187	239	3.00E-05	39.3
TcMYB8	AtMYB64	55.08	118	53	0	511	864	88	205	3.00E-41	149
TcMYB8	AtMYB64	30.53	95	66	1	406	690	105	198	6.00E-13	63.9
TcMYB8	AtMYB64	24.53	106	79	2	553	867	63	155	5.00E-04	35.4
TcMYB8	ATMYB73	58.49	106	44	0	547	864	8	113	4.00E-38	137
TcMYB8	ATMYB73	33.68	95	63	1	406	690	13	106	3.00E-15	70.1
TcMYB8	AtMYB1	53.77	106	49	0	559	876	54	159	1.00E-37	138
TcMYB8	AtMYB1	34.91	106	69	1	373	690	44	148	3.00E-17	77
TcMYB8	AtMYB115	54	100	46	0	562	861	158	257	5.00E-36	132
TcMYB8	AtMYB115	41.05	95	56	1	406	690	158	251	4.00E-18	79.3
TcMYB8	ATMYB44	53.77	106	49	0	559	876	5	110	5.00E-36	131
TcMYB8	ATMYB44	35.79	95	61	1	406	690	6	99	7.00E-15	68.9
TcMYB8	ATMYB77	48.72	117	60	0	559	909	5	121	8.00E-36	130
TcMYB8	ATMYB77	36.84	95	60	1	406	690	6	99	1.00E-16	73.9
TcMYB8	AtMYB70	56.86	102	44	0	559	864	12	113	1.00E-35	130
TcMYB8	AtMYB70	30.36	112	78	1	406	741	13	123	4.00E-14	66.6
TcMYB8	AtMYB109	53.64	110	51	0	559	888	55	164	5.00E-35	130
TcMYB8	AtMYB109	29.63	162	112	4	271	750	14	168	3.00E-15	70.9
TcMYB8	ATMYB105	50.93	108	53	0	562	885	107	214	1.00E-33	125
TcMYB8	ATMYB105	30.21	96	67	1	403	690	106	200	9.00E-14	65.9
TcMYB8	ATMYB105	29.51	122	64	4	247	546	91	204	2.00E-05	40
TcMYB8	AtMYB100	53.61	97	45	0	571	861	29	125	1.00E-33	122
TcMYB8	AtMYB100	35.79	95	61	1	406	690	26	119	5.00E-15	68.2
TcMYB8	AtMYB100	30.91	55	36	2	709	867	23	76	8.00E-04	34.3
TcMYB8	ATMYB69	49.52	105	53	0	550	864	15	119	2.00E-33	122
TcMYB8	ATMYB69	36.05	86	55	1	406	663	19	103	4.00E-15	68.6
TcMYB8	AtMYB56	53.47	101	47	0	562	864	93	193	2.00E-33	124
TcMYB8	AtMYB56	28.7	108	77	1	367	690	80	186	6.00E-13	63.2
TcMYB8	ATMYB54	49.5	101	51	0	562	864	6	106	4.00E-33	120
TcMYB8	ATMYB54	32.29	96	65	1	403	690	5	99	3.00E-15	68.9
TcMYB8	ATMYB54	26.42	106	78	2	229	546	18	103	3.00E-04	35.8
TcMYB8	ATMYB110	50.93	108	53	0	562	885	67	174	6.00E-33	122
TcMYB8	ATMYB110	29.17	96	68	1	403	690	66	160	7.00E-12	59.7
TcMYB8	ATMYB25	49.15	118	60	0	559	912	49	166	6.00E-33	124
TcMYB8	ATMYB25	32.43	111	75	1	370	702	38	147	1.00E-14	68.9
TcMYB8	AtMYB117	53.47	101	47	0	562	864	98	198	7.00E-33	123
TcMYB8	AtMYB117	25.48	157	117	2	220	690	46	191	6.00E-15	69.7
TcMYB8	AtMYB117	40	50	30	1	397	546	147	195	3.00E-05	39.3
TcMYB8	ATMYB52	48.51	101	52	0	562	864	5	105	7.00E-33	120
TcMYB8	ATMYB52	33.33	96	64	1	403	690	4	98	1.00E-15	70.5
TcMYB8	ATMYB52	25.47	106	79	2	229	546	17	102	2.00E-04	36.6
TcMYB8	AtMYB49	30.1	289	184	8	562	1374	14	291	1.00E-31	119
TcMYB8	AtMYB49	29.81	104	72	2	406	714	14	116	5.00E-12	60.5
TcMYB8	AtMYB89	48.62	109	56	0	565	891	57	165	2.00E-31	114
TcMYB8	AtMYB89	28.89	135	96	2	409	813	57	182	3.00E-14	64.7
TcMYB8	AtMYB89	32.56	86	58	3	403	660	107	182	8.00E-06	40
TcMYB8	ATMYB63	48.08	104	53	1	562	870	16	119	5.00E-30	113
TcMYB8	ATMYB63	31.86	113	76	2	406	741	16	127	3.00E-14	67
TcMYB8	ATMYB95	38.62	145	88	1	556	987	12	156	7.00E-30	112
TcMYB8	ATMYB65	49.04	104	52	1	556	864	41	144	2.00E-29	116
TcMYB8	ATMYB65	31.82	132	76	4	385	738	36	166	6.00E-13	64.3
TcMYB8	AtMYB53	47.62	105	54	1	556	867	12	116	2.00E-29	112
TcMYB8	AtMYB53	29.81	104	72	2	406	714	14	116	8.00E-12	59.7
TcMYB8	ATMYB92	48.57	105	53	1	556	867	12	116	2.00E-29	112
TcMYB8	ATMYB92	31.73	104	70	2	406	714	14	116	1.00E-12	62.4
TcMYB8	AtMYB9	47.62	105	54	1	556	867	12	116	2.00E-29	112
TcMYB8	AtMYB9	33.65	104	68	2	406	714	14	116	9.00E-14	65.9
TcMYB8	ATMYB58	46.67	105	55	1	562	873	16	120	5.00E-29	110

TcMYB8	ATMYB58	28.97	107	75	2	406	723	16	121	1.00E-11	58.5
TcMYB8	ATMYB26	35.11	188	100	3	562	1059	14	201	6.00E-29	112
TcMYB8	ATMYB26	27.73	119	76	3	406	732	14	127	7.00E-09	50.8
TcMYB8	ATMYB35	47.17	106	55	1	553	867	11	116	6.00E-29	110
TcMYB8	ATMYB35	31.13	106	72	2	406	720	14	118	8.00E-11	56.6
TcMYB8	ATMYB67	45.28	106	56	2	562	873	24	128	7.00E-29	110
TcMYB8	ATMYB67	30.63	111	76	3	406	735	24	129	1.00E-10	56.2
TcMYB8	AtMYB27	44.76	105	56	2	556	864	9	112	1.00E-28	108
TcMYB8	AtMYB27	34.34	99	64	2	406	699	11	108	6.00E-14	65.1
TcMYB8	AtMYB22	45.63	103	56	1	553	861	51	152	1.00E-28	108
TcMYB8	AtMYB22	37.5	96	60	2	403	690	53	146	1.00E-15	70.5
TcMYB8	AtMYB22	28.3	53	38	0	709	867	51	103	7.00E-06	40.8
TcMYB8	AtMYB45	35.91	181	113	3	562	1095	20	193	1.00E-28	108
TcMYB8	AtMYB45	36.89	103	64	2	397	702	17	118	2.00E-13	63.5
TcMYB8	AtMYB107	46.67	105	55	1	556	867	12	116	2.00E-28	109
TcMYB8	AtMYB107	31.73	104	70	2	406	714	14	116	6.00E-13	63.2
TcMYB8	ATMYB72	42.98	114	63	2	562	897	16	128	2.00E-28	108
TcMYB8	ATMYB72	28.44	109	77	2	391	714	11	118	3.00E-12	60.8
TcMYB8	AtMYB93	41.41	128	66	2	556	912	12	139	2.00E-28	110
TcMYB8	AtMYB93	29.81	104	72	2	406	714	14	116	6.00E-11	57.4
TcMYB8	ATMYB102	48.57	105	53	1	556	867	12	116	2.00E-28	110
TcMYB8	ATMYB102	30.77	104	71	2	406	714	14	116	7.00E-12	60.1
TcMYB8	ATMYB33	44.83	116	63	2	520	864	23	135	2.00E-28	112
TcMYB8	ATMYB33	32	125	71	4	406	738	34	157	2.00E-12	62.8
TcMYB8	AtMYB41	46.6	103	54	1	562	867	14	116	3.00E-28	108
TcMYB8	AtMYB41	29.81	104	72	2	406	714	14	116	3.00E-12	60.5
TcMYB8	ATMYB15	46.3	108	57	1	556	876	12	119	3.00E-28	108
TcMYB8	ATMYB15	33.33	105	69	2	406	717	14	117	3.00E-14	66.6
TcMYB8	AtMYB81	46.73	107	56	1	544	861	16	122	4.00E-28	110
TcMYB8	AtMYB81	30.69	101	69	2	403	702	21	120	2.00E-11	59.3
TcMYB8	ATMYB3	45.71	105	56	1	562	873	14	118	5.00E-28	106
TcMYB8	ATMYB3	31.43	105	71	2	403	714	13	116	1.00E-13	64.7
TcMYB8	ATMYB3	34.62	52	34	1	406	561	67	117	5.00E-04	35
TcMYB8	MYB8	42.72	103	58	1	562	867	14	116	6.00E-28	105
TcMYB8	MYB8	30.48	105	72	2	403	714	13	116	5.00E-12	58.9
TcMYB8	ATMYB59-3	40	110	65	1	538	864	2	111	8.00E-28	105
TcMYB8	ATMYB59-3	33.02	106	70	2	406	720	10	114	8.00E-16	70.5
TcMYB8	AtMYB6	43.4	106	59	1	562	876	14	119	1.00E-27	104
TcMYB8	AtMYB6	29.36	109	76	2	391	714	9	116	6.00E-12	58.9
TcMYB8	ATMYB122	46.6	103	54	1	556	861	12	114	2.00E-27	106
TcMYB8	ATMYB122	29	100	70	2	406	702	14	112	2.00E-11	58.9
TcMYB8	AtMYB51	28.3	311	222	10	556	1485	13	271	2.00E-27	107
TcMYB8	AtMYB51	29.25	106	74	2	406	720	15	119	2.00E-12	61.6
TcMYB8	ATMYB14	41.82	110	63	1	562	888	14	123	3.00E-27	104
TcMYB8	ATMYB14	30.84	107	73	2	406	723	14	119	6.00E-15	68.2
TcMYB8	AtMYB74	38.35	133	81	2	556	951	13	144	3.00E-27	106
TcMYB8	AtMYB74	30.19	106	73	2	406	720	15	119	8.00E-12	59.7
TcMYB8	AtMYB18	48.54	103	51	2	562	864	12	113	3.00E-27	105
TcMYB8	AtMYB18	31.36	118	80	3	397	747	9	120	4.00E-12	60.1
TcMYB8	AtMYB50	47.17	106	54	2	556	867	12	116	3.00E-27	105
TcMYB8	AtMYB50	31.13	106	72	2	406	720	14	118	2.00E-10	55.1
TcMYB8	ATMYB48	43.14	102	57	1	562	864	9	110	3.00E-27	104
TcMYB8	ATMYB48	33.96	106	69	2	406	720	9	113	8.00E-16	70.9
TcMYB8	ATMYB86	47.17	106	54	2	556	867	12	116	3.00E-27	106
TcMYB8	ATMYB86	28.3	106	75	2	406	720	14	118	9.00E-10	53.5
TcMYB8	ATMYB80	42.06	107	61	1	553	870	11	117	3.00E-27	105
TcMYB8	ATMYB80	30.19	106	73	3	406	720	14	114	8.00E-10	53.5
TcMYB8	AtMYB32	35.06	174	107	4	562	1065	14	183	3.00E-27	104
TcMYB8	AtMYB32	31.13	106	72	2	400	714	12	116	5.00E-13	62.8
TcMYB8	ATMYB121	45.54	101	54	1	562	861	29	129	3.00E-27	104
TcMYB8	ATMYB121	28.93	159	112	2	406	879	29	186	2.00E-15	70.1
TcMYB8	AtMYB19	47.57	103	52	2	562	864	14	115	4.00E-27	104
TcMYB8	AtMYB19	30.19	106	73	2	388	702	8	112	1.00E-11	58.5
TcMYB8	AtMYB19	29.85	67	46	2	406	603	67	132	3.00E-05	38.9
TcMYB8	ATMYB55	44.44	117	52	1	556	867	12	128	4.00E-27	106
TcMYB8	ATMYB55	28.21	117	71	2	406	717	14	129	4.00E-10	54.7
TcMYB8	ATMYB2	42.16	102	58	1	562	864	22	123	4.00E-27	104
TcMYB8	ATMYB2	33.91	115	75	2	376	717	12	125	2.00E-14	67
TcMYB8	ATMYB71	44.34	106	57	2	562	873	20	124	6.00E-27	103
TcMYB8	ATMYB71	33.63	113	74	2	406	741	20	131	1.00E-14	67.4
TcMYB8	AtMYB79	45.63	103	54	2	562	864	8	109	6.00E-27	103
TcMYB8	AtMYB79	32.38	105	70	2	406	717	8	111	1.00E-13	64.3
TcMYB8	ATMYB61	48.11	106	53	2	556	867	12	116	7.00E-27	105
TcMYB8	ATMYB61	31.13	106	72	2	406	720	14	118	8.00E-11	57
TcMYB8	MYB7	43.81	105	58	1	562	873	14	118	1.00E-26	103
TcMYB8	MYB7	31.43	105	71	2	403	714	13	116	2.00E-12	61.2
TcMYB8	AtMYB112	39.09	110	66	1	535	861	25	134	1.00E-26	102
TcMYB8	AtMYB112	29.52	105	73	2	406	717	34	137	7.00E-11	55.8
TcMYB8	AtMYB20	46.23	106	55	2	556	867	12	116	1.00E-26	103
TcMYB8	AtMYB20	27.36	106	76	2	406	720	14	118	3.00E-10	54.3

TcMYB8	ATMYB46	44.66	103	56	1	562	867	20	122	2.00E-26	102
TcMYB8	ATMYB46	26.61	109	79	2	397	720	17	124	2.00E-08	48.9
TcMYB8	AtMYB97	46.6	103	54	1	556	861	19	121	2.00E-26	105
TcMYB8	AtMYB97	35	100	64	2	406	702	21	119	2.00E-12	62
TcMYB8	AtMYB103	46.6	103	54	1	562	867	14	116	2.00E-26	104
TcMYB8	AtMYB103	27.03	111	80	2	391	720	9	118	4.00E-10	54.7
TcMYB8	ATMYB0	39.66	116	69	1	547	891	11	126	2.00E-26	101
TcMYB8	ATMYB0	34.58	107	69	2	406	723	16	121	6.00E-15	67.8
TcMYB8	AtMYB43	46.23	106	55	2	556	867	12	116	2.00E-26	103
TcMYB8	AtMYB43	28.3	106	75	2	406	720	14	118	3.00E-10	54.7
TcMYB8	AtMYB85	47.12	104	53	2	562	867	14	116	2.00E-26	102
TcMYB8	AtMYB85	28.3	106	75	2	406	720	14	118	3.00E-10	54.3
TcMYB8	AtMYB17	46.3	108	56	2	556	873	12	118	3.00E-26	102
TcMYB8	AtMYB17	28.85	104	73	2	406	714	14	116	5.00E-12	60.1
TcMYB8	ATMYB13	33.33	183	112	5	556	1074	12	187	3.00E-26	101
TcMYB8	ATMYB13	30.77	104	71	2	406	714	14	116	1.00E-12	61.2
TcMYB8	ATMYB28	44.95	109	59	1	544	867	8	116	4.00E-26	103
TcMYB8	ATMYB28	30.77	104	71	2	406	714	14	116	6.00E-11	57.4
TcMYB8	ATMYB4	43.69	103	57	1	562	867	14	116	4.00E-26	101
TcMYB8	ATMYB4	31.19	109	74	2	391	714	9	116	2.00E-13	63.9
TcMYB8	ATMYB4	36.54	52	33	1	406	561	67	117	3.00E-04	35.8
TcMYB8	AtMYB116	41.18	102	59	1	562	864	20	121	5.00E-26	101
TcMYB8	AtMYB116	30.19	106	73	2	406	720	20	124	2.00E-11	58.2
TcMYB8	ATMYB66	41.75	103	59	1	562	867	18	120	6.00E-26	99
TcMYB8	ATMYB66	33.05	118	76	3	370	714	4	120	2.00E-16	71.6
TcMYB8	AtMYB42	46.15	104	54	2	562	867	14	116	7.00E-26	101
TcMYB8	AtMYB42	24.7	166	108	3	406	852	14	178	1.00E-09	52.8
TcMYB8	ATMYB57	40.91	110	64	1	535	861	18	127	7.00E-26	99
TcMYB8	ATMYB57	31.82	110	74	2	391	717	22	130	4.00E-12	58.9
TcMYB8	AtMYB108	41.35	104	60	1	553	861	18	121	7.00E-26	102
TcMYB8	AtMYB108	30.19	106	73	2	406	720	21	125	6.00E-11	57
TcMYB8	AtMYB10	43.27	104	57	2	562	867	16	118	8.00E-26	99.8
TcMYB8	AtMYB10	33.96	106	69	2	406	720	16	120	1.00E-14	67
TcMYB8	AtMYB24	40.78	103	60	1	562	867	19	121	8.00E-26	99
TcMYB8	AtMYB24	32.48	117	78	2	373	720	8	123	1.00E-12	60.5
TcMYB8	ATMYB101	48.57	105	52	2	556	864	18	121	1.00E-25	104
TcMYB8	ATMYB101	33.96	106	68	3	379	690	10	114	1.00E-11	60.1
TcMYB8	ATMYB16	41.9	105	60	1	556	867	12	116	1.00E-25	101
TcMYB8	ATMYB16	27.36	106	76	2	406	720	14	118	1.00E-10	55.8
TcMYB8	AtMYB40	40.57	106	61	2	556	867	12	116	1.00E-25	99.8
TcMYB8	AtMYB40	32.71	107	70	4	406	720	14	114	1.00E-08	49.7
TcMYB8	AtMYB40	34.62	52	33	2	406	558	67	116	9.00E-04	34.3
TcMYB8	ATMYB29	44.86	107	58	1	544	861	8	114	2.00E-25	101
TcMYB8	ATMYB29	32	100	67	2	406	702	14	112	1.00E-10	56.2
TcMYB8	AtMYB76	43.12	109	61	1	544	867	8	116	2.00E-25	100
TcMYB8	AtMYB76	29.91	107	74	2	406	723	14	119	9.00E-11	56.6
TcMYB8	ATMYB106	41.9	105	60	1	556	867	55	159	3.00E-25	101
TcMYB8	ATMYB106	28.3	106	75	2	406	720	57	161	8.00E-11	57
TcMYB8	ATMYB3	40.78	103	60	1	562	867	22	124	3.00E-25	97.8
TcMYB8	ATMYB3	25.91	193	125	5	373	897	6	192	9.00E-13	61.2
TcMYB8	AtMYB62	39.42	104	62	1	556	864	19	122	3.00E-25	99.4
TcMYB8	AtMYB62	31.78	107	71	4	406	720	21	125	3.00E-12	60.8
TcMYB8	ATMYB123	44.76	105	57	1	556	867	14	118	6.00E-25	97.8
TcMYB8	ATMYB123	26.72	116	84	2	403	747	15	129	3.00E-12	60.1
TcMYB8	ATMYB5	40.78	103	60	1	562	867	25	127	6.00E-25	97.4
TcMYB8	ATMYB5	28.7	108	72	3	406	714	25	127	1.00E-10	55.5
TcMYB8	AtMYB83	42.06	107	61	1	550	867	28	134	8.00E-25	99.4
TcMYB8	AtMYB83	29.63	108	75	2	406	726	32	138	3.00E-08	48.9
TcMYB8	ATMYB23	40.78	103	60	1	562	867	14	116	8.00E-25	96.3
TcMYB8	ATMYB23	33.33	111	73	2	385	714	7	116	6.00E-16	70.5
TcMYB8	ATMYB34	43.69	103	57	1	562	867	14	116	9.00E-25	98.2
TcMYB8	ATMYB34	28.3	106	75	2	406	720	14	118	9.00E-11	56.2
TcMYB8	AtMYB47	42.31	104	59	1	562	870	14	117	9.00E-25	97.4
TcMYB8	AtMYB47	23.64	110	79	3	406	720	14	118	6.00E-08	47.4
TcMYB8	ATMYB120	45.63	103	55	1	556	861	26	128	2.00E-24	100
TcMYB8	ATMYB120	34	100	65	2	406	702	28	126	5.00E-11	58.2
TcMYB8	ATMYB96	41.82	110	63	1	562	888	14	123	2.00E-24	98.6
TcMYB8	ATMYB96	32.46	114	76	2	406	744	14	126	4.00E-14	67
TcMYB8	ATMYB96	33.87	62	41	1	406	591	67	127	7.00E-05	38.1
TcMYB8	AtMYB36	42.99	107	59	2	553	867	11	117	3.00E-24	97.4
TcMYB8	AtMYB36	31.03	116	78	4	406	747	14	124	3.00E-11	58.2
TcMYB8	AtMYB114	42.59	108	57	2	556	864	8	111	3.00E-24	92
TcMYB8	AtMYB114	30.69	101	69	2	406	705	10	109	2.00E-13	61.2
TcMYB8	ATMYB31	43.93	107	55	2	562	867	14	116	5.00E-24	96.7
TcMYB8	ATMYB31	30.77	104	71	2	406	714	14	116	1.00E-11	59.3
TcMYB8	AtMYB60	44.66	103	56	1	562	867	14	116	7.00E-24	95.1
TcMYB8	AtMYB60	28.95	152	92	5	406	813	14	160	1.00E-13	65.1
TcMYB8	ATMYB94	42.72	103	58	1	562	867	14	116	7.00E-24	96.3
TcMYB8	ATMYB94	31.13	106	72	2	406	720	14	118	5.00E-13	63.5
TcMYB8	AtMYB124	39.8	98	59	0	571	864	28	125	1.00E-23	97.1

TcMYB8	AtMYB124	28.7	108	77	1	415	738	28	134	1.00E-13	66.2
TcMYB8	ATMYB11	39.32	117	70	1	562	909	14	130	2.00E-23	95.5
TcMYB8	ATMYB11	30.77	104	71	2	406	714	14	116	9.00E-11	56.6
TcMYB8	ATMYB30	42.72	103	58	1	562	867	14	116	2.00E-23	94.7
TcMYB8	ATMYB30	29.84	124	82	3	391	747	9	127	5.00E-13	63.5
TcMYB8	ATMYB99	43.36	113	55	3	556	867	13	124	2.00E-23	92.8
TcMYB8	ATMYB99	29.73	111	70	4	406	714	15	124	8.00E-10	52.8
TcMYB8	ATMYB37	42.45	106	59	2	562	873	14	119	2.00E-23	94.7
TcMYB8	ATMYB37	33.03	109	67	4	406	714	14	117	4.00E-11	57.8
TcMYB8	ATMYB75	42.59	108	57	2	556	864	8	111	4.00E-23	92
TcMYB8	ATMYB75	30.69	101	69	2	406	705	10	109	1.00E-12	61.2
TcMYB8	ATMYB90	29.19	209	143	4	556	1167	8	198	4.00E-23	92
TcMYB8	ATMYB90	30.69	101	69	2	406	705	10	109	6.00E-12	59.3
TcMYB8	ATMYB111	40	110	65	1	556	882	12	121	5.00E-23	94
TcMYB8	ATMYB111	32.69	104	69	2	406	714	14	116	7.00E-12	60.1
TcMYB8	AtMYB82	37.8	127	78	1	562	939	14	140	5.00E-23	90.5
TcMYB8	AtMYB82	31.48	108	71	3	391	705	7	113	5.00E-13	61.6
TcMYB8	ATMYB84	42.06	107	60	2	553	867	11	117	8.00E-23	92.8
TcMYB8	ATMYB84	29.55	132	82	5	406	768	14	144	4.00E-11	57.4
TcMYB8	ATMYB12	30.82	159	109	2	562	1035	14	165	2.00E-22	92.8
TcMYB8	ATMYB12	26.73	217	147	7	406	1020	14	219	3.00E-12	61.6
TcMYB8	ATMYB68	41.12	107	61	2	553	867	11	117	2.00E-22	92.8
TcMYB8	ATMYB68	28.48	158	104	5	406	852	14	164	6.00E-11	57.4
TcMYB8	ATMYB78	34.78	115	60	2	562	861	28	142	3.00E-22	91.3
TcMYB8	ATMYB78	27.73	119	72	2	406	720	28	146	1.00E-09	52.8
TcMYB8	ATMYB87	39.62	106	62	2	562	873	14	119	3.00E-22	90.9
TcMYB8	ATMYB87	33.63	113	73	4	406	738	14	121	5.00E-12	60.1
TcMYB8	ATMYB88	37.76	98	61	0	571	864	33	130	5.00E-22	92.8
TcMYB8	ATMYB88	29.46	112	79	1	415	750	33	143	6.00E-15	70.5
TcMYB8	AtMYB104	40.95	105	57	2	562	861	18	118	7.00E-22	91.3
TcMYB8	AtMYB104	32.67	101	63	3	403	690	17	112	3.00E-12	61.6
TcMYB8	AtMYB113	40.74	108	59	2	556	864	8	111	2.00E-21	87
TcMYB8	AtMYB113	31.68	101	68	2	406	705	10	109	3.00E-12	60.1
TcMYB8	ATMYB38	36.61	112	69	1	553	882	11	122	1.00E-20	86.3
TcMYB8	ATMYB38	31.78	107	71	4	406	720	14	115	2.00E-09	52.4
TcMYB8	ATMYB91	37.23	94	56	1	571	843	7	100	9.00E-17	75.5
TcMYB8	ATMYB91	29.23	130	89	3	415	795	7	129	5.00E-14	67
TcMYB8	ATMYB91	35.71	70	45	3	406	615	59	123	5.00E-04	35.4
TcMYB9	ATMYB91	70.83	144	42	1	1	432	1	141	5.00E-67	214
TcMYB9	ATMYB91	57.84	102	43	0	1252	1557	259	360	4.00E-35	128
TcMYB9	AtMYB82	42.34	111	64	1	7	339	13	121	3.00E-28	104
TcMYB9	ATMYB30	30.95	210	127	5	19	594	17	222	2.00E-27	105
TcMYB9	ATMYB15	28.43	197	138	2	7	588	13	207	8.00E-27	102
TcMYB9	ATMYB59-3	40.19	107	64	1	19	339	13	117	2.00E-26	100
TcMYB9	ATMYB66	42.86	112	63	2	19	351	21	130	3.00E-26	99
TcMYB9	ATMYB94	42.98	114	65	2	19	360	17	125	3.00E-26	102
TcMYB9	AtMYB60	41.44	111	65	1	19	351	17	125	3.00E-26	100
TcMYB9	AtMYB19	32.86	213	125	8	4	588	9	193	4.00E-26	100
TcMYB9	ATMYB48	39.25	107	65	1	19	339	12	116	4.00E-26	100
TcMYB9	AtMYB79	38.14	118	73	1	19	372	11	126	4.00E-26	100
TcMYB9	AtMYB62	32.23	211	131	7	10	606	21	213	5.00E-26	100
TcMYB9	ATMYB57	38.79	116	71	1	19	366	30	143	6.00E-26	98.2
TcMYB9	ATMYB120	26.88	372	234	12	19	1020	31	366	7.00E-26	103
TcMYB9	ATMYB96	43.27	104	59	1	19	330	17	118	1.00E-25	100
TcMYB9	AtMYB104	36.81	144	89	3	10	435	18	158	2.00E-25	100
TcMYB9	ATMYB31	44.12	102	57	1	19	324	17	116	4.00E-25	99
TcMYB9	AtMYB116	42.31	104	60	1	19	330	23	124	6.00E-25	97.4
TcMYB9	ATMYB123	29.65	226	140	5	10	630	16	239	9.00E-25	96.3
TcMYB9	ATMYB3	39.42	104	63	1	19	330	25	126	1.00E-24	95.1
TcMYB9	ATMYB63	36.67	120	76	1	7	366	15	132	2.00E-24	95.9
TcMYB9	ATMYB71	35.59	118	76	1	19	372	23	138	3.00E-24	95.1
TcMYB9	ATMYB80	39.62	106	64	1	7	324	13	116	4.00E-24	95.9
TcMYB9	AtMYB18	41.18	119	67	3	7	354	8	120	4.00E-24	95.1
TcMYB9	ATMYB2	39.62	106	64	1	19	336	25	128	4.00E-24	94.7
TcMYB9	ATMYB0	42.16	102	59	1	19	324	19	118	4.00E-24	93.6
TcMYB9	AtMYB45	38.79	116	68	2	4	342	15	128	4.00E-24	94.4
TcMYB9	ATMYB23	40.2	102	61	1	19	324	17	116	5.00E-24	93.2
TcMYB9	AtMYB97	41.96	112	65	2	19	354	24	127	5.00E-24	96.7
TcMYB9	AtMYB108	28.57	217	138	4	7	606	20	231	5.00E-24	95.5
TcMYB9	AtMYB112	39.62	106	64	1	10	327	34	137	6.00E-24	93.6
TcMYB9	ATMYB101	40.18	112	67	2	19	354	23	126	6.00E-24	97.4
TcMYB9	ATMYB121	37.96	108	67	1	19	342	32	137	8.00E-24	94
TcMYB9	AtMYB113	43.81	105	59	1	19	333	13	115	8.00E-24	93.2
TcMYB9	AtMYB114	42.57	101	58	1	19	321	13	111	1.00E-23	89.7
TcMYB9	AtMYB50	42.86	105	60	1	19	333	17	119	1.00E-23	94.4
TcMYB9	ATMYB86	41.9	105	61	1	19	333	17	119	1.00E-23	94.7
TcMYB9	ATMYB75	42.48	113	61	2	19	345	13	123	2.00E-23	92.4
TcMYB9	ATMYB84	41.96	112	63	3	19	348	17	126	2.00E-23	93.6
TcMYB9	AtMYB24	39.22	102	62	1	19	324	22	121	2.00E-23	91.3
TcMYB9	AtMYB56	39.82	113	68	1	10	348	93	202	2.00E-23	93.6

TcMYB9	ATMYB68	31.31	198	127	5	19	585	17	212	2.00E-23	94.4
TcMYB9	AtMYB51	40.95	105	62	1	19	333	18	120	2.00E-23	94
TcMYB9	AtMYB36	40	115	68	2	19	360	17	129	3.00E-23	93.6
TcMYB9	ATMYB65	40.18	112	67	2	19	354	46	149	3.00E-23	95.5
TcMYB9	ATMYB90	43.56	101	57	1	19	321	13	111	4.00E-23	91.3
TcMYB9	ATMYB33	40.18	112	67	2	19	354	37	140	5.00E-23	94.7
TcMYB9	AtMYB10	37.61	117	73	1	7	357	15	129	6.00E-23	90.5
TcMYB9	ATMYB34	42.86	105	60	1	19	333	17	119	7.00E-23	91.7
TcMYB9	ATMYB55	33.51	188	109	4	19	534	17	191	8.00E-23	92.4
TcMYB9	AtMYB17	44.12	102	57	1	19	324	17	116	1.00E-22	91.3
TcMYB9	ATMYB14	38.89	108	66	1	7	330	13	118	1.00E-22	90.1
TcMYB9	ATMYB5	36.13	119	76	2	7	363	24	135	1.00E-22	90.1
TcMYB9	AtMYB53	41.18	102	60	1	19	324	17	116	1.00E-22	91.3
TcMYB9	AtMYB49	41.18	102	60	1	19	324	17	116	1.00E-22	91.3
TcMYB9	ATMYB28	33.12	160	106	2	19	495	17	174	2.00E-22	91.7
TcMYB9	ATMYB61	40	105	63	1	19	333	17	119	3.00E-22	90.9
TcMYB9	AtMYB41	42.16	102	59	1	19	324	17	116	3.00E-22	89.4
TcMYB9	ATMYB29	41.9	105	61	1	19	333	17	119	4.00E-22	90.1
TcMYB9	ATMYB46	40.38	104	62	1	19	330	23	124	4.00E-22	89
TcMYB9	AtMYB107	39.22	102	62	1	19	324	17	116	4.00E-22	89.7
TcMYB9	ATMYB16	39.22	102	62	1	19	324	17	116	5.00E-22	89.7
TcMYB9	AtMYB103	38.89	108	66	1	7	330	13	118	6.00E-22	90.1
TcMYB9	ATMYB67	39.62	106	64	1	7	324	23	126	7.00E-22	89
TcMYB9	ATMYB3	39.22	102	62	1	19	324	17	116	7.00E-22	87.8
TcMYB9	ATMYB119	36.61	112	71	1	16	351	107	215	7.00E-22	90.5
TcMYB9	ATMYB37	40	115	69	1	7	351	13	126	1.00E-21	88.6
TcMYB9	ATMYB106	39.22	102	62	1	19	324	60	159	1.00E-21	89.4
TcMYB9	ATMYB92	39.22	102	62	1	19	324	17	116	1.00E-21	88.6
TcMYB9	AtMYB81	44.68	94	52	1	19	300	25	116	1.00E-21	89.7
TcMYB9	AtMYB9	39.22	102	62	1	19	324	17	116	1.00E-21	88.6
TcMYB9	ATMYB26	29.03	217	119	5	7	552	13	228	1.00E-21	89
TcMYB9	AtMYB74	42.16	102	59	1	19	324	18	117	2.00E-21	88.2
TcMYB9	ATMYB122	40.95	105	62	1	19	333	17	119	2.00E-21	88.2
TcMYB9	ATMYB58	36.7	109	69	1	7	333	15	121	2.00E-21	87
TcMYB9	AtMYB64	36.03	136	82	3	16	408	107	239	3.00E-21	88.6
TcMYB9	AtMYB76	41.18	102	60	1	19	324	17	116	3.00E-21	87.4
TcMYB9	ATMYB13	31.35	185	127	5	19	573	17	173	4.00E-21	85.5
TcMYB9	ATMYB4	41.18	102	60	1	19	324	17	116	4.00E-21	86.3
TcMYB9	AtMYB27	39.05	105	63	2	10	321	11	113	4.00E-21	85.1
TcMYB9	MYB7	39.05	105	64	1	19	333	17	119	6.00E-21	85.5
TcMYB9	ATMYB95	39.05	105	64	1	19	333	17	119	6.00E-21	85.5
TcMYB9	AtMYB32	39.05	105	64	1	19	333	17	119	6.00E-21	85.5
TcMYB9	ATMYB102	41.18	102	60	1	19	324	17	116	7.00E-21	86.7
TcMYB9	ATMYB87	37.38	107	66	2	19	336	17	121	7.00E-21	85.9
TcMYB9	ATMYB52	36.52	115	73	1	1	345	2	113	7.00E-21	84.7
TcMYB9	ATMYB72	36.79	106	67	1	7	324	15	118	1.00E-20	85.1
TcMYB9	ATMYB12	36.79	106	67	1	7	324	13	116	2.00E-20	85.9
TcMYB9	MYB8	35.29	102	66	1	19	324	17	116	2.00E-20	82.8
TcMYB9	AtMYB98	38.32	107	66	1	16	336	219	322	2.00E-20	86.3
TcMYB9	AtMYB98	32.39	71	47	1	124	333	200	270	2.00E-06	42
TcMYB9	AtMYB47	36.7	109	69	1	7	333	13	119	2.00E-20	84
TcMYB9	AtMYB85	42.16	102	59	1	19	324	17	116	2.00E-20	83.6
TcMYB9	ATMYB105	37.96	108	67	1	10	333	107	211	3.00E-20	84.3
TcMYB9	AtMYB42	40.74	108	64	1	1	324	11	116	3.00E-20	83.6
TcMYB9	AtMYB83	36.84	114	72	1	19	360	35	146	4.00E-20	84.3
TcMYB9	ATMYB35	39.22	102	62	1	19	324	17	116	5.00E-20	83.6
TcMYB9	AtMYB20	39.42	104	63	1	19	330	17	118	6.00E-20	82.8
TcMYB9	ATMYB54	32.62	141	85	3	1	393	3	140	1.00E-19	81.3
TcMYB9	AtMYB117	38.32	107	66	1	10	330	98	201	1.00E-19	83.2
TcMYB9	ATMYB69	39.45	109	65	2	7	330	18	122	1.00E-19	81.3
TcMYB9	ATMYB38	38.32	107	65	2	7	324	13	117	1.00E-19	82
TcMYB9	ATMYB118	34.51	113	74	1	16	354	191	300	1.00E-19	83.6
TcMYB9	AtMYB6	36.27	102	65	1	19	324	17	116	2.00E-19	80.5
TcMYB9	AtMYB93	38.24	102	63	1	19	324	17	116	2.00E-19	82.4
TcMYB9	ATMYB111	35.71	98	63	1	7	300	13	108	2.00E-19	82
TcMYB9	ATMYB11	33.96	106	70	1	7	324	13	116	4.00E-19	81.3
TcMYB9	AtMYB43	40.38	104	62	1	19	330	17	118	5.00E-19	80.9
TcMYB9	ATMYB25	40.4	99	59	1	4	300	48	143	7.00E-19	80.9
TcMYB9	ATMYB78	31.43	140	82	3	10	387	28	162	8.00E-19	80.1
TcMYB9	ATMYB44	30.1	206	142	6	19	630	9	180	8.00E-19	79.7
TcMYB9	ATMYB99	39.45	109	59	2	19	324	18	124	2.00E-18	77.8
TcMYB9	AtMYB115	36.04	111	69	2	16	342	160	267	4.00E-18	78.6
TcMYB9	ATMYB73	37.23	94	59	1	19	300	16	106	8.00E-18	77
TcMYB9	AtMYB70	37.23	94	59	1	19	300	16	106	1.00E-17	76.3
TcMYB9	ATMYB77	28.38	222	149	7	19	654	9	218	2.00E-17	75.9
TcMYB9	AtMYB109	31.34	134	92	1	4	405	54	184	2.00E-17	76.6
TcMYB9	AtMYB40	34.58	107	70	1	7	327	13	117	4.00E-17	73.9
TcMYB9	ATMYB88	30.23	129	88	2	4	384	26	151	9.00E-17	75.1
TcMYB9	ATMYB110	34.26	108	71	1	10	333	67	171	1.00E-16	73.6
TcMYB9	AtMYB22	34.62	104	67	3	10	318	54	152	8.00E-16	70.1

TcMYB9	AtMYB124	31.36	118	79	2	4	351	21	135	2.00E-15	70.9
TcMYB9	AtMYB1	37.23	94	59	1	19	300	58	148	2.00E-15	70.5
TcMYB9	AtMYB100	34.23	111	72	4	7	336	25	130	2.00E-15	68.2
TcMYB9	AtMYB89	30.43	115	79	2	19	360	59	170	2.00E-14	64.3
TcMYB10	AtMYB70	43.81	105	57	2	49	357	15	115	2.00E-18	76.6
TcMYB10	ATMYB44	52.86	70	33	0	49	258	8	77	3.00E-18	76.3
TcMYB10	ATMYB73	41.75	103	60	1	49	357	15	115	9.00E-18	75.1
TcMYB10	ATMYB77	39.47	114	67	2	49	384	8	117	1.00E-17	74.3
TcMYB10	ATMYB25	40.74	108	50	2	25	306	44	151	2.00E-17	74.7
TcMYB10	AtMYB109	48.24	85	40	1	16	258	43	127	2.00E-17	74.7
TcMYB10	AtMYB1	38.74	111	51	2	4	285	37	147	5.00E-15	67.4
TcMYB10	ATMYB3	28.23	209	130	8	13	579	9	211	2.00E-12	58.2
TcMYB10	AtMYB56	31.2	125	62	3	52	354	96	220	9.00E-12	57.4
TcMYB10	ATMYB54	31.4	121	62	3	52	351	9	129	1.00E-11	56.2
TcMYB10	ATMYB69	28.79	132	82	2	52	411	22	147	2.00E-11	55.8
TcMYB10	ATMYB52	34.44	90	47	1	52	285	8	97	6.00E-11	54.3
TcMYB10	ATMYB63	26.97	152	110	4	49	501	18	158	2.00E-10	53.1
TcMYB10	AtMYB18	30.54	167	99	5	52	501	15	180	6.00E-10	51.6
TcMYB10	AtMYB19	30.46	174	100	8	52	510	17	181	2.00E-09	50.1
TcMYB10	AtMYB117	32.22	90	49	1	52	285	101	190	4.00E-09	49.3
TcMYB10	ATMYB105	32.22	90	49	1	52	285	110	199	5.00E-09	48.9
TcMYB10	AtMYB112	28.91	128	77	4	49	390	36	153	9.00E-09	47.8
TcMYB10	AtMYB107	24.44	225	123	7	10	543	9	227	9.00E-09	48.1
TcMYB10	AtMYB108	27.23	202	123	7	1	534	13	207	9.00E-09	48.1
TcMYB10	ATMYB121	35.4	113	58	4	34	327	26	137	1.00E-08	47.8
TcMYB10	ATMYB59-3	31.3	131	71	6	49	384	12	141	1.00E-08	47.4
TcMYB10	ATMYB80	33.33	99	51	3	52	303	17	115	2.00E-08	47.4
TcMYB10	ATMYB37	34.4	125	65	7	49	372	16	139	2.00E-08	47.4
TcMYB10	ATMYB48	29.86	144	85	6	1	384	2	137	2.00E-08	46.6
TcMYB10	AtMYB79	33.07	127	70	5	10	345	3	122	2.00E-08	46.6
TcMYB10	ATMYB71	33.62	116	62	5	10	312	15	123	2.00E-08	46.6
TcMYB10	ATMYB2	27.71	166	99	6	49	483	24	188	2.00E-08	46.6
TcMYB10	ATMYB61	27.92	197	114	9	52	558	17	204	6.00E-08	45.8
TcMYB10	ATMYB106	35.14	74	47	1	31	249	53	126	6.00E-08	45.8
TcMYB10	AtMYB45	28.4	162	83	6	52	438	23	183	7.00E-08	45.1
TcMYB10	AtMYB103	27.16	162	99	6	52	480	17	175	8.00E-08	45.4
TcMYB10	AtMYB98	32.56	86	56	1	7	258	203	288	8.00E-08	45.4
TcMYB10	AtMYB53	33.9	118	62	6	10	315	9	119	9.00E-08	45.1
TcMYB10	ATMYB88	38.46	52	32	0	46	201	31	82	9.00E-08	45.4
TcMYB10	AtMYB116	28.46	123	73	4	13	336	10	131	1.00E-07	44.7
TcMYB10	ATMYB16	36.76	68	42	1	49	249	16	83	1.00E-07	44.7
TcMYB10	ATMYB46	29.03	155	92	6	52	462	23	174	1.00E-07	44.3
TcMYB10	AtMYB64	22.86	175	121	4	52	534	108	275	1.00E-07	44.7
TcMYB10	ATMYB119	40	50	30	0	52	201	108	157	2.00E-07	44.7
TcMYB10	AtMYB82	37.62	101	47	5	52	306	17	116	2.00E-07	43.5
TcMYB10	ATMYB14	25.79	159	100	6	49	471	16	171	2.00E-07	43.5
TcMYB10	AtMYB51	25.68	183	122	7	4	510	8	176	2.00E-07	43.9
TcMYB10	AtMYB93	34.57	81	52	2	10	249	9	83	2.00E-07	43.9
TcMYB10	ATMYB91	25.97	231	138	7	52	645	7	232	2.00E-07	43.9
TcMYB10	ATMYB87	32.73	110	60	4	49	336	16	124	3.00E-07	43.5
TcMYB10	AtMYB10	26.09	138	86	4	49	414	18	154	3.00E-07	43.1
TcMYB10	ATMYB92	34.48	87	53	3	31	279	10	95	3.00E-07	43.5
TcMYB10	AtMYB24	27.66	141	86	4	10	384	5	143	3.00E-07	42.7
TcMYB10	ATMYB118	33.33	78	50	1	52	279	192	269	4.00E-07	43.5
TcMYB10	AtMYB27	32.26	93	49	3	49	285	13	104	4.00E-07	42.7
TcMYB10	AtMYB17	31.88	69	45	2	49	249	16	83	5.00E-07	42.7
TcMYB10	AtMYB124	36.54	52	33	0	46	201	26	77	6.00E-07	42.7
TcMYB10	ATMYB72	31.03	116	64	5	49	348	18	129	6.00E-07	42.4
TcMYB10	ATMYB12	28.8	125	67	5	52	360	17	139	8.00E-07	42.4
TcMYB10	ATMYB58	32	100	53	3	49	303	18	117	8.00E-07	42
TcMYB10	AtMYB104	27.72	101	59	3	52	312	21	120	1.00E-06	42
TcMYB10	AtMYB97	28.3	106	63	2	7	285	9	114	1.00E-06	42
TcMYB10	AtMYB74	30.84	107	61	3	4	285	8	108	1.00E-06	41.6
TcMYB10	ATMYB57	25.95	131	83	4	49	399	29	158	1.00E-06	40.8
TcMYB10	AtMYB36	30.53	131	71	8	49	381	16	143	2.00E-06	41.2
TcMYB10	ATMYB35	28.75	160	77	6	52	420	17	175	2.00E-06	40.8
TcMYB10	AtMYB9	30.48	105	60	3	10	285	9	107	2.00E-06	40.8
TcMYB10	AtMYB62	27.93	111	65	4	49	336	23	132	3.00E-06	40.4
TcMYB10	ATMYB15	28.03	132	75	4	49	384	16	147	4.00E-06	40
TcMYB10	ATMYB110	32.31	65	44	0	52	246	70	134	4.00E-06	40
TcMYB10	ATMYB84	32.41	108	58	5	49	327	16	121	4.00E-06	40
TcMYB10	ATMYB30	28.99	138	78	6	34	387	11	147	4.00E-06	40
TcMYB10	AtMYB6	25	172	115	4	52	525	17	186	4.00E-06	39.7
TcMYB10	ATMYB102	31.17	77	52	1	31	258	10	86	4.00E-06	40
TcMYB10	AtMYB115	30.43	69	48	0	52	258	161	229	4.00E-06	40
TcMYB10	ATMYB34	32.94	85	55	3	10	258	9	86	5.00E-06	39.7
TcMYB10	ATMYB68	32.73	110	57	5	49	327	16	124	6.00E-06	39.7
TcMYB10	ATMYB120	28.26	92	53	2	49	285	30	121	6.00E-06	39.7
TcMYB10	AtMYB76	22.16	167	128	4	28	522	9	157	7.00E-06	39.3
TcMYB10	AtMYB49	29.35	92	52	2	49	285	16	107	9.00E-06	38.9

TcMYB10	AtMYB41	31.17	77	52	1	31	258	10	86	1.00E-05	38.5
TcMYB10	AtMYB50	31.07	103	55	4	52	312	17	118	1.00E-05	38.5
TcMYB10	ATMYB78	27.5	80	57	1	49	285	30	109	2.00E-05	38.1
TcMYB10	ATMYB94	28.46	130	78	2	34	378	11	140	2.00E-05	38.1
TcMYB10	ATMYB11	30.77	91	50	2	52	285	17	107	2.00E-05	38.1
TcMYB10	ATMYB28	30.38	79	53	2	28	258	9	86	2.00E-05	38.1
TcMYB10	AtMYB40	26.77	127	77	5	49	381	16	137	2.00E-05	37.7
TcMYB10	AtMYB81	29.55	88	60	3	49	306	24	105	2.00E-05	38.1
TcMYB10	MYB7	28.97	145	86	4	52	435	17	160	2.00E-05	37.7
TcMYB10	ATMYB111	30.77	91	50	2	52	285	17	107	2.00E-05	37.7
TcMYB10	AtMYB85	24.34	189	116	7	34	519	11	198	2.00E-05	37.4
TcMYB10	ATMYB29	30.38	79	53	2	28	258	9	86	3.00E-05	37.4
TcMYB10	ATMYB96	28.79	132	79	4	34	384	11	135	3.00E-05	37.4
TcMYB10	ATMYB13	30.69	101	54	4	49	303	16	115	3.00E-05	37
TcMYB10	AtMYB60	25	172	111	5	49	510	16	184	3.00E-05	37
TcMYB10	ATMYB3	28.06	139	83	5	52	417	17	150	4.00E-05	36.6
TcMYB10	ATMYB123	27.1	155	98	5	52	471	19	171	4.00E-05	36.6
TcMYB10	ATMYB101	27.27	99	59	2	28	285	15	113	5.00E-05	37
TcMYB10	ATMYB31	33.93	56	32	2	49	201	16	67	7.00E-05	36.2
TcMYB10	ATMYB86	31	100	53	4	52	303	17	115	7.00E-05	36.2
TcMYB10	ATMYB5	31.52	92	50	2	49	285	27	118	7.00E-05	35.8
TcMYB10	ATMYB33	27.1	107	65	3	4	285	22	127	8.00E-05	36.2
TcMYB10	ATMYB65	27.17	92	54	2	49	285	45	136	8.00E-05	36.2
TcMYB10	ATMYB38	42.55	47	25	2	49	183	16	62	8.00E-05	35.8
TcMYB10	ATMYB67	24.32	148	95	6	52	444	27	173	9.00E-05	35.8
TcMYB10	AtMYB89	37.5	40	25	0	52	171	59	98	1.00E-04	35
TcMYB10	AtMYB32	26.72	131	75	4	52	381	17	146	1.00E-04	35.4
TcMYB10	ATMYB66	28.97	107	62	3	7	285	6	111	1.00E-04	35
TcMYB10	MYB8	27.47	91	53	2	52	285	17	107	1.00E-04	35
TcMYB10	AtMYB42	25.18	139	76	5	49	381	16	153	2.00E-04	34.7
TcMYB10	AtMYB100	27.54	69	49	2	43	246	26	93	2.00E-04	34.3
TcMYB10	AtMYB20	28.1	121	59	6	49	327	16	135	3.00E-04	34.3
TcMYB10	ATMYB4	30.77	91	50	2	52	285	17	107	3.00E-04	33.9
TcMYB10	ATMYB122	29.58	71	48	2	52	258	17	86	4.00E-04	33.9
TcMYB10	ATMYB26	29.09	110	54	4	52	309	17	126	4.00E-04	33.9
TcMYB10	AtMYB83	31.31	99	53	3	52	303	35	133	7.00E-04	33.1
TcMYB11	ATMYB118	39.22	204	103	2	229	777	187	390	2.00E-43	154
TcMYB11	ATMYB118	37.04	108	66	2	49	366	177	283	2.00E-18	80.9
TcMYB11	AtMYB98	45.64	149	80	1	94	537	169	317	3.00E-43	154
TcMYB11	AtMYB98	37.86	103	62	2	64	366	210	311	5.00E-18	79.3
TcMYB11	ATMYB119	46.75	154	75	2	208	648	96	247	4.00E-41	148
TcMYB11	ATMYB119	38.54	96	59	1	79	366	105	199	8.00E-16	72.4
TcMYB11	AtMYB64	51.69	118	57	0	184	537	88	205	8.00E-40	144
TcMYB11	AtMYB64	34.31	102	67	1	61	366	99	199	1.00E-14	68.9
TcMYB11	AtMYB1	47.59	145	75	1	232	663	54	198	5.00E-39	141
TcMYB11	AtMYB1	37.27	110	69	1	46	375	44	152	9.00E-18	78.2
TcMYB11	ATMYB73	54.72	106	48	0	220	537	8	113	7.00E-38	136
TcMYB11	ATMYB73	35.14	111	69	2	52	375	1	110	5.00E-17	75.1
TcMYB11	AtMYB115	56.73	104	45	1	235	546	158	260	1.00E-37	136
TcMYB11	AtMYB115	41.67	96	56	1	79	366	158	252	2.00E-19	82.8
TcMYB11	AtMYB70	54.9	102	46	0	232	537	12	113	3.00E-36	131
TcMYB11	AtMYB70	34.23	111	70	2	52	375	1	110	1.00E-15	70.9
TcMYB11	ATMYB44	50.94	106	52	0	232	549	5	110	2.00E-35	129
TcMYB11	ATMYB44	37.37	99	62	1	79	375	6	103	1.00E-16	73.6
TcMYB11	ATMYB77	40.28	144	86	1	232	663	5	147	6.00E-35	127
TcMYB11	ATMYB77	38.38	99	61	1	79	375	6	103	4.00E-17	75.1
TcMYB11	AtMYB109	51.96	102	49	0	232	537	55	156	4.00E-34	127
TcMYB11	AtMYB109	36.45	107	68	1	55	375	48	153	9.00E-16	72
TcMYB11	ATMYB25	49.02	102	52	0	232	537	49	150	6.00E-33	123
TcMYB11	ATMYB25	35.59	118	76	2	22	375	33	147	1.00E-15	71.6
TcMYB11	ATMYB54	52.63	95	45	0	235	519	6	100	7.00E-33	119
TcMYB11	ATMYB54	36.08	97	62	1	76	366	5	100	4.00E-17	73.9
TcMYB11	AtMYB100	48.54	103	53	0	226	534	23	125	1.00E-32	119
TcMYB11	AtMYB100	33.03	109	73	1	79	405	26	133	6.00E-15	67.4
TcMYB11	ATMYB52	51.58	95	46	0	235	519	5	99	3.00E-32	118
TcMYB11	ATMYB52	37.11	97	61	1	76	366	4	99	1.00E-17	75.9
TcMYB11	ATMYB52	36	50	32	1	70	219	54	102	5.00E-04	34.7
TcMYB11	AtMYB53	32.91	234	147	6	229	900	12	238	3.00E-32	119
TcMYB11	AtMYB53	34	100	65	2	79	375	14	112	2.00E-13	63.9
TcMYB11	AtMYB49	50.94	106	51	1	226	540	11	116	7.00E-32	119
TcMYB11	AtMYB49	36	100	63	2	79	375	14	112	4.00E-15	69.3
TcMYB11	AtMYB56	38.82	170	104	2	235	744	93	255	1.00E-31	118
TcMYB11	AtMYB56	31.19	109	75	1	40	366	80	187	2.00E-14	67
TcMYB11	AtMYB117	50.5	101	50	0	235	537	98	198	1.00E-31	119
TcMYB11	AtMYB117	32.29	96	65	1	79	366	98	192	4.00E-14	66.6
TcMYB11	AtMYB117	36	50	32	1	70	219	147	195	3.00E-04	35.8
TcMYB11	ATMYB105	50.5	101	50	0	235	537	107	207	2.00E-31	118
TcMYB11	ATMYB105	32.99	97	65	1	76	366	106	201	1.00E-14	68.2
TcMYB11	ATMYB105	34	50	33	1	70	219	156	204	3.00E-04	35.4
TcMYB11	ATMYB69	44.44	117	64	1	223	570	15	131	2.00E-31	115

TcMYB11	ATMYB69	33.33	96	64	1	79	366	19	113	1.00E-15	70.1
TcMYB11	AtMYB107	49.52	105	52	1	229	540	12	116	3.00E-31	117
TcMYB11	AtMYB107	34	100	65	2	79	375	14	112	2.00E-14	67.4
TcMYB11	AtMYB9	48.57	105	53	1	229	540	12	116	6.00E-31	116
TcMYB11	AtMYB9	36	100	63	2	79	375	14	112	8.00E-15	68.6
TcMYB11	ATMYB0	44.26	122	67	1	220	582	11	132	7.00E-31	113
TcMYB11	ATMYB0	32.5	120	80	2	40	396	3	121	8.00E-14	63.9
TcMYB11	AtMYB41	48.54	103	52	1	235	540	14	116	7.00E-31	115
TcMYB11	AtMYB41	34	100	65	2	79	375	14	112	5.00E-14	65.5
TcMYB11	ATMYB15	49.07	108	54	1	229	549	12	119	7.00E-31	115
TcMYB11	ATMYB15	36	100	63	2	79	375	14	112	3.00E-15	69.3
TcMYB11	ATMYB65	44.72	123	67	2	172	537	25	144	8.00E-31	119
TcMYB11	ATMYB33	44.26	122	67	2	175	537	17	135	9.00E-31	119
TcMYB11	ATMYB67	50.96	104	49	2	235	540	24	126	9.00E-31	115
TcMYB11	ATMYB67	33.67	98	64	2	79	369	24	120	2.00E-13	64.3
TcMYB11	AtMYB93	30.26	228	150	4	229	885	12	239	1.00E-30	116
TcMYB11	AtMYB93	34	100	65	2	79	375	14	112	2.00E-13	64.3
TcMYB11	ATMYB35	33.94	221	139	5	235	876	14	222	1.00E-30	115
TcMYB11	ATMYB35	31.13	106	72	2	79	393	14	118	1.00E-10	55.5
TcMYB11	AtMYB74	49.52	105	52	1	229	540	13	117	1.00E-30	115
TcMYB11	AtMYB74	33.96	106	69	2	79	393	15	119	7.00E-15	68.6
TcMYB11	ATMYB23	40.28	144	80	3	226	639	11	151	1.00E-30	112
TcMYB11	ATMYB23	35.19	108	69	2	58	378	7	113	1.00E-15	68.9
TcMYB11	ATMYB110	49.02	102	52	0	235	540	67	168	2.00E-30	114
TcMYB11	ATMYB110	32.67	101	68	1	64	366	62	161	1.00E-12	61.6
TcMYB11	ATMYB95	48.57	105	53	1	229	540	12	116	2.00E-30	113
TcMYB11	ATMYB95	30	110	72	3	79	393	14	118	1.00E-09	52.4
TcMYB11	ATMYB66	35.68	185	111	5	220	750	13	194	2.00E-30	111
TcMYB11	ATMYB66	35.4	113	71	3	79	411	18	129	2.00E-16	70.9
TcMYB11	ATMYB92	50	106	51	2	229	540	12	116	4.00E-30	114
TcMYB11	ATMYB92	35	100	64	2	79	375	14	112	1.00E-14	67.8
TcMYB11	ATMYB2	46.08	102	54	1	235	537	22	123	4.00E-30	112
TcMYB11	ATMYB2	35	100	64	2	79	375	22	120	4.00E-11	56.6
TcMYB11	AtMYB10	33.15	184	121	3	235	780	16	193	4.00E-30	111
TcMYB11	AtMYB10	33.96	106	69	2	79	393	16	120	7.00E-15	67.4
TcMYB11	ATMYB102	49.52	105	52	1	229	540	12	116	5.00E-30	114
TcMYB11	ATMYB102	35	100	64	2	79	375	14	112	1.00E-13	65.1
TcMYB11	ATMYB121	47.62	105	54	1	235	546	29	133	7.00E-30	112
TcMYB11	ATMYB121	36.36	99	62	2	79	372	29	126	9.00E-13	61.6
TcMYB11	MYB8	45.63	103	55	1	235	540	14	116	1.00E-29	109
TcMYB11	MYB8	33.66	101	66	2	76	375	13	112	1.00E-12	60.5
TcMYB11	AtMYB24	37.68	138	84	2	226	633	16	153	1.00E-29	109
TcMYB11	AtMYB24	30.99	71	49	1	79	291	72	141	9.00E-06	39.7
TcMYB11	AtMYB81	49.5	101	50	1	235	534	22	122	3.00E-29	113
TcMYB11	AtMYB81	31.68	101	68	2	76	375	21	120	1.00E-10	55.8
TcMYB11	AtMYB51	47.62	105	54	1	229	540	13	117	3.00E-29	112
TcMYB11	AtMYB51	35.85	106	67	2	79	393	15	119	2.00E-16	73.9
TcMYB11	ATMYB72	39.31	145	85	4	235	660	16	148	4.00E-29	110
TcMYB11	ATMYB72	32	100	67	2	79	375	16	114	1.00E-12	61.6
TcMYB11	ATMYB5	37.42	155	94	4	235	690	25	169	5.00E-29	108
TcMYB11	ATMYB5	30.77	104	67	3	79	375	25	123	1.00E-10	55.1
TcMYB11	AtMYB22	43.69	103	58	1	226	534	51	152	5.00E-29	108
TcMYB11	AtMYB22	34.55	110	72	2	76	405	53	160	1.00E-15	70.1
TcMYB11	AtMYB6	46.23	106	56	1	235	549	14	119	6.00E-29	108
TcMYB11	AtMYB6	33.33	102	67	2	73	375	12	112	6.00E-13	61.6
TcMYB11	ATMYB3	46.6	103	54	1	235	540	14	116	7.00E-29	108
TcMYB11	ATMYB3	35.64	101	64	2	76	375	13	112	2.00E-14	66.6
TcMYB11	AtMYB112	36.51	126	79	1	208	582	25	150	1.00E-28	107
TcMYB11	ATMYB86	48.6	107	53	2	226	540	11	116	1.00E-28	110
TcMYB11	ATMYB86	31.13	106	72	2	79	393	14	118	1.00E-11	58.9
TcMYB11	AtMYB40	30.63	222	148	5	229	876	12	229	1.00E-28	108
TcMYB11	AtMYB40	32.2	118	67	6	79	393	14	127	3.00E-09	50.8
TcMYB11	ATMYB61	49.53	107	52	2	226	540	11	116	1.00E-28	110
TcMYB11	ATMYB61	32.08	106	71	2	79	393	14	118	1.00E-11	58.9
TcMYB11	ATMYB101	50	104	51	1	229	537	18	121	1.00E-28	112
TcMYB11	ATMYB101	30.77	104	71	2	67	375	16	118	5.00E-10	54.3
TcMYB11	AtMYB50	47.66	107	54	2	226	540	11	116	1.00E-28	109
TcMYB11	AtMYB50	33.02	106	70	2	79	393	14	118	7.00E-12	59.3
TcMYB11	ATMYB123	45.95	111	58	2	214	540	9	118	2.00E-28	107
TcMYB11	ATMYB123	29.91	107	74	2	76	393	15	120	1.00E-11	58.2
TcMYB11	ATMYB120	49.52	105	52	1	229	540	26	130	2.00E-28	112
TcMYB11	ATMYB120	32.2	118	72	3	46	375	10	126	6.00E-11	57.4
TcMYB11	AtMYB27	43.52	108	59	2	229	546	9	115	2.00E-28	106
TcMYB11	AtMYB27	35.35	99	63	2	79	372	11	108	1.00E-14	66.6
TcMYB11	AtMYB47	46.6	103	54	1	235	540	14	116	2.00E-28	107
TcMYB11	AtMYB47	28.18	110	74	3	79	393	14	118	5.00E-10	53.1
TcMYB11	AtMYB20	34.1	217	125	9	229	825	12	223	2.00E-28	107
TcMYB11	AtMYB20	29.25	106	74	2	79	393	14	118	2.00E-11	57.8
TcMYB11	ATMYB26	43.48	115	55	1	235	549	14	128	2.00E-28	109
TcMYB11	ATMYB26	28.32	113	71	2	67	375	10	121	4.00E-11	57.4

TcMYB11	AtMYB97	48.54	103	52	1	229	534	19	121	2.00E-28	110
TcMYB11	AtMYB97	26.83	205	135	7	79	648	21	215	5.00E-13	63.5
TcMYB11	AtMYB17	50	106	51	2	229	540	12	116	2.00E-28	108
TcMYB11	AtMYB17	35.29	102	63	3	79	375	14	112	3.00E-14	66.6
TcMYB11	ATMYB46	48.54	103	52	1	235	540	20	122	3.00E-28	107
TcMYB11	ATMYB46	28.18	110	78	2	67	393	16	124	7.00E-11	55.8
TcMYB11	AtMYB85	34.5	200	129	6	235	828	14	190	3.00E-28	107
TcMYB11	AtMYB85	30.19	106	73	2	79	393	14	118	2.00E-11	57.8
TcMYB11	AtMYB32	44.66	103	56	1	235	540	14	116	4.00E-28	107
TcMYB11	AtMYB32	34.31	102	66	2	73	375	12	112	6.00E-14	65.1
TcMYB11	ATMYB71	34.27	178	106	4	235	735	20	196	4.00E-28	106
TcMYB11	ATMYB71	36.36	99	62	2	79	372	20	117	1.00E-13	63.9
TcMYB11	ATMYB122	46.67	105	55	1	229	540	12	116	4.00E-28	108
TcMYB11	ATMYB122	32.73	110	69	3	79	393	14	118	3.00E-15	69.7
TcMYB11	ATMYB4	47.57	103	53	1	235	540	14	116	6.00E-28	106
TcMYB11	ATMYB4	36.27	102	64	2	73	375	12	112	1.00E-14	67.4
TcMYB11	MYB7	46.6	103	54	1	235	540	14	116	6.00E-28	106
TcMYB11	MYB7	35.64	101	64	2	76	375	13	112	8.00E-14	64.7
TcMYB11	ATMYB13	47.17	106	54	2	229	540	12	116	9.00E-28	105
TcMYB11	ATMYB13	32.71	107	69	3	79	390	14	117	1.00E-13	63.5
TcMYB11	ATMYB59-3	41.82	110	63	1	211	537	2	111	1.00E-27	104
TcMYB11	ATMYB59-3	35	100	64	2	79	375	10	108	1.00E-14	66.6
TcMYB11	AtMYB60	47.12	104	54	1	235	543	14	117	1.00E-27	105
TcMYB11	AtMYB60	32.37	139	86	5	34	426	4	132	2.00E-13	63.5
TcMYB11	ATMYB3	44.23	104	57	1	226	534	19	122	1.00E-27	103
TcMYB11	ATMYB3	33.96	106	69	2	79	393	22	126	1.00E-11	57.8
TcMYB11	ATMYB58	41.9	105	60	1	235	546	16	120	2.00E-27	105
TcMYB11	ATMYB58	24.27	239	177	8	79	783	16	230	5.00E-13	62.4
TcMYB11	AtMYB79	46.6	103	53	2	235	537	8	109	2.00E-27	104
TcMYB11	AtMYB79	34.34	99	64	2	79	372	8	105	3.00E-13	62.8
TcMYB11	ATMYB31	46.23	106	56	1	226	540	11	116	2.00E-27	106
TcMYB11	ATMYB31	34	100	65	2	79	375	14	112	2.00E-11	58.2
TcMYB11	AtMYB89	46.46	99	53	0	238	534	57	155	3.00E-27	102
TcMYB11	AtMYB89	30.46	151	104	3	37	486	41	182	2.00E-16	70.9
TcMYB11	AtMYB42	33.82	204	133	6	235	840	14	189	3.00E-27	104
TcMYB11	AtMYB42	25.3	166	107	3	79	525	14	178	3.00E-11	57
TcMYB11	ATMYB80	42.72	103	58	1	235	540	14	116	3.00E-27	105
TcMYB11	ATMYB80	30.48	105	72	2	79	390	14	117	7.00E-11	56.2
TcMYB11	ATMYB30	46.15	104	55	1	235	543	14	117	3.00E-27	105
TcMYB11	ATMYB30	33.64	110	68	3	79	393	14	118	1.00E-12	61.6
TcMYB11	AtMYB103	46.6	103	54	1	235	540	14	116	4.00E-27	106
TcMYB11	AtMYB103	30.36	112	75	3	67	393	10	118	4.00E-12	60.5
TcMYB11	ATMYB106	28.81	295	205	7	229	1098	55	341	5.00E-27	106
TcMYB11	ATMYB106	33.02	106	70	2	79	393	57	161	6.00E-14	66.2
TcMYB11	AtMYB45	46.53	101	53	1	235	534	20	120	6.00E-27	103
TcMYB11	AtMYB45	35.51	107	68	2	70	387	17	122	4.00E-14	65.5
TcMYB11	AtMYB83	44.86	107	58	1	223	540	28	134	6.00E-27	105
TcMYB11	AtMYB83	31.48	108	73	2	79	399	32	138	3.00E-10	54.3
TcMYB11	AtMYB43	48.11	106	53	2	229	540	12	116	6.00E-27	104
TcMYB11	AtMYB43	29.25	106	74	2	79	393	14	118	1.00E-10	55.8
TcMYB11	ATMYB63	43.69	103	57	1	235	540	16	118	6.00E-27	103
TcMYB11	ATMYB63	35.4	113	72	2	79	414	16	127	5.00E-18	77.8
TcMYB11	ATMYB63	33.33	63	42	1	79	267	69	130	6.00E-05	37.7
TcMYB11	ATMYB96	28.2	266	187	7	235	1020	14	264	7.00E-27	105
TcMYB11	ATMYB96	31.78	129	87	3	34	417	4	126	7.00E-13	62.8
TcMYB11	ATMYB96	26.92	104	76	2	79	390	67	158	2.00E-05	39.3
TcMYB11	AtMYB116	34.03	144	92	2	235	657	20	163	7.00E-27	103
TcMYB11	AtMYB116	24.08	245	175	7	79	780	20	257	4.00E-11	56.6
TcMYB11	AtMYB62	30.65	186	124	4	226	768	18	186	7.00E-27	103
TcMYB11	ATMYB16	42.86	105	59	1	229	540	12	116	8.00E-27	104
TcMYB11	ATMYB16	32.08	106	71	2	79	393	14	118	2.00E-13	63.9
TcMYB11	ATMYB14	42.99	107	60	1	235	552	14	120	8.00E-27	102
TcMYB11	ATMYB14	32.71	107	71	2	79	396	14	119	2.00E-16	72.4
TcMYB11	ATMYB94	41.41	128	67	3	235	594	14	139	9.00E-27	104
TcMYB11	ATMYB94	31.4	121	82	3	34	393	4	118	5.00E-12	60.1
TcMYB11	ATMYB48	44.12	102	56	1	235	537	9	110	1.00E-26	102
TcMYB11	ATMYB48	36	100	63	2	79	375	9	107	1.00E-14	67
TcMYB11	ATMYB57	43.69	103	57	1	235	540	27	129	1.00E-26	100
TcMYB11	ATMYB34	41.44	111	64	1	235	564	14	124	2.00E-26	102
TcMYB11	ATMYB34	32.08	106	71	2	79	393	14	118	2.00E-13	63.9
TcMYB11	AtMYB108	31.14	167	114	2	226	723	18	182	3.00E-26	102
TcMYB11	ATMYB55	40.68	118	57	1	226	540	11	128	4.00E-26	102
TcMYB11	ATMYB55	31.25	112	64	2	79	375	14	124	2.00E-11	58.2
TcMYB11	ATMYB28	38.85	139	84	2	229	642	12	149	5.00E-26	102
TcMYB11	ATMYB28	34	100	65	2	79	375	14	112	2.00E-12	61.6
TcMYB11	AtMYB18	45.1	102	55	1	235	537	12	113	8.00E-26	100
TcMYB11	AtMYB18	27.07	229	131	6	70	648	9	231	2.00E-14	67
TcMYB11	AtMYB76	43.81	105	58	1	229	540	12	116	1.00E-25	100
TcMYB11	AtMYB76	32.71	107	71	2	79	396	14	119	1.00E-12	61.6
TcMYB11	ATMYB29	44.76	105	57	1	229	540	12	116	2.00E-25	100

TcMYB11	ATMYB29	33	100	66	2	79	375	14	112	8.00E-12	59.3
TcMYB11	AtMYB19	36.18	152	93	4	235	678	14	161	4.00E-25	98.2
TcMYB11	AtMYB19	33.61	119	78	3	61	414	8	120	2.00E-15	69.3
TcMYB11	AtMYB19	28.36	67	47	2	79	276	67	132	1.00E-04	36.6
TcMYB11	ATMYB11	42.45	106	60	1	235	549	14	119	5.00E-25	99.4
TcMYB11	ATMYB11	35.64	101	64	2	79	378	14	113	2.00E-13	64.3
TcMYB11	ATMYB111	34.75	141	91	2	229	648	12	150	2.00E-24	97.4
TcMYB11	ATMYB111	34.62	104	67	2	79	387	14	116	2.00E-12	61.2
TcMYB11	AtMYB82	40.95	105	61	1	235	546	14	118	2.00E-24	94
TcMYB11	AtMYB82	31.43	105	71	2	79	390	14	117	2.00E-12	59.3
TcMYB11	ATMYB12	40.19	107	63	1	235	552	14	120	3.00E-24	97.4
TcMYB11	ATMYB12	33.66	101	66	2	79	378	14	113	3.00E-12	60.8
TcMYB11	AtMYB114	39.42	104	62	1	229	537	8	111	4.00E-24	91.3
TcMYB11	AtMYB114	29.63	108	75	2	58	378	3	109	6.00E-13	59.3
TcMYB11	ATMYB78	34.35	131	71	2	235	582	28	158	1.00E-23	95.1
TcMYB11	ATMYB78	30.09	113	65	2	79	375	28	140	4.00E-09	50.8
TcMYB11	AtMYB124	38.78	98	60	0	244	537	28	125	1.00E-23	96.7
TcMYB11	AtMYB124	30.56	108	75	1	88	411	28	134	2.00E-12	62
TcMYB11	AtMYB124	31.75	63	43	1	79	267	77	138	2.00E-04	36.6
TcMYB11	ATMYB99	44.25	113	54	3	229	540	13	124	2.00E-23	92.8
TcMYB11	ATMYB99	33.64	107	63	4	79	375	15	120	3.00E-11	56.6
TcMYB11	ATMYB75	40.38	104	61	1	229	537	8	111	2.00E-23	92.4
TcMYB11	ATMYB75	29.63	108	75	2	58	378	3	109	3.00E-12	59.7
TcMYB11	ATMYB87	40.38	104	60	2	235	540	14	117	3.00E-23	93.6
TcMYB11	ATMYB87	33.66	101	65	3	79	375	14	113	3.00E-10	54.3
TcMYB11	ATMYB90	32.72	162	98	3	229	681	8	168	6.00E-23	91.3
TcMYB11	ATMYB90	29.63	108	75	2	58	378	3	109	2.00E-11	57.4
TcMYB11	AtMYB36	40.38	104	60	2	235	540	14	117	2.00E-22	91.7
TcMYB11	AtMYB36	32.67	101	66	3	79	375	14	113	2.00E-10	55.1
TcMYB11	AtMYB113	38.68	106	64	1	229	543	8	113	5.00E-22	88.6
TcMYB11	AtMYB113	31.43	105	67	3	79	378	10	109	3.00E-11	56.6
TcMYB11	ATMYB68	36.22	127	79	3	235	609	14	136	7.00E-22	90.5
TcMYB11	ATMYB68	29.11	158	103	6	79	525	14	164	2.00E-10	55.5
TcMYB11	ATMYB88	36.73	98	62	0	244	537	33	130	7.00E-22	91.7
TcMYB11	ATMYB88	31.25	112	77	1	88	423	33	143	2.00E-14	68.2
TcMYB11	ATMYB88	47.22	36	19	1	79	186	82	116	2.00E-04	36.2
TcMYB11	ATMYB84	41.35	104	59	2	235	540	14	117	7.00E-22	89.4
TcMYB11	ATMYB84	32.41	108	70	4	79	393	14	120	5.00E-10	53.5
TcMYB11	AtMYB104	41.84	98	55	2	235	522	18	114	1.00E-21	89.7
TcMYB11	AtMYB104	33.33	102	63	3	76	366	17	113	3.00E-12	60.8
TcMYB11	ATMYB38	39.42	104	61	2	235	540	14	117	1.00E-20	85.5
TcMYB11	ATMYB38	31.68	101	67	3	79	375	14	113	6.00E-09	50.1
TcMYB11	ATMYB37	38.46	104	62	2	235	540	14	117	3.00E-20	85.1
TcMYB11	ATMYB37	32.38	105	65	4	79	375	14	113	1.00E-10	55.8
TcMYB11	ATMYB91	35.11	94	58	1	244	516	7	100	6.00E-16	72.4
TcMYB11	ATMYB91	32.28	127	83	3	88	459	7	126	2.00E-14	67.4
TcMYB12	ATMYB88	22.65	362	275	12	28	1098	33	356	4.00E-17	75.9
TcMYB12	ATMYB69	35.51	107	68	2	22	339	20	124	1.00E-16	72
TcMYB12	ATMYB54	40	90	53	1	7	273	2	91	3.00E-16	70.9
TcMYB12	AtMYB115	37.8	82	51	0	10	255	155	236	7.00E-16	71.2
TcMYB12	ATMYB77	28.1	153	95	3	22	435	7	159	8.00E-16	70.5
TcMYB12	ATMYB52	24.03	258	171	6	7	705	1	246	1.00E-15	68.9
TcMYB12	ATMYB66	35.16	91	57	2	16	282	17	107	2.00E-15	67.4
TcMYB12	AtMYB98	40	85	50	1	22	273	218	302	2.00E-15	70.1
TcMYB12	AtMYB98	29.9	97	65	4	73	354	182	277	1.00E-05	39.7
TcMYB12	AtMYB100	38.16	76	47	0	28	255	29	104	4.00E-15	67.4
TcMYB12	ATMYB105	31.01	129	87	3	13	393	105	232	6.00E-15	68.2
TcMYB12	ATMYB0	32.71	107	70	2	16	330	15	121	6.00E-15	66.6
TcMYB12	ATMYB44	30.25	119	80	2	22	369	7	125	8.00E-15	67.4
TcMYB12	AtMYB124	33.94	109	66	2	28	336	28	136	1.00E-14	68.2
TcMYB12	AtMYB56	25.69	144	106	1	22	450	94	237	1.00E-14	67.4
TcMYB12	ATMYB122	22.36	161	120	2	13	480	12	172	1.00E-14	67
TcMYB12	AtMYB64	38.82	85	51	1	22	273	106	190	2.00E-14	67.4
TcMYB12	AtMYB64	23.86	197	142	6	91	657	75	233	2.00E-05	38.5
TcMYB12	AtMYB82	35.16	91	58	2	13	282	12	99	2.00E-14	64.7
TcMYB12	AtMYB89	32.98	94	63	0	16	297	55	148	2.00E-14	64.3
TcMYB12	AtMYB114	32.97	91	60	2	13	282	8	95	2.00E-14	62.8
TcMYB12	AtMYB109	27.27	165	97	4	22	447	57	220	3.00E-14	66.6
TcMYB12	ATMYB110	25.79	159	110	2	22	474	68	223	3.00E-14	65.9
TcMYB12	AtMYB117	38.82	85	51	1	22	273	99	183	4.00E-14	65.9
TcMYB12	AtMYB70	30.69	101	69	1	22	321	14	114	5.00E-14	65.1
TcMYB12	ATMYB73	29.7	101	70	1	22	321	14	114	5.00E-14	65.1
TcMYB12	ATMYB118	38.82	85	51	1	22	273	190	274	7.00E-14	65.5
TcMYB12	ATMYB118	32	100	64	3	64	351	150	249	4.00E-05	37.7
TcMYB12	ATMYB23	32.97	91	59	2	16	282	13	103	8.00E-14	63.2
TcMYB12	ATMYB119	37.65	85	52	1	22	273	106	190	1.00E-13	64.7
TcMYB12	ATMYB119	28.57	77	53	2	106	330	82	158	0.001	33.5
TcMYB12	ATMYB15	26.72	131	94	3	13	399	12	139	1.00E-13	63.5
TcMYB12	AtMYB45	33.71	89	57	2	22	282	21	109	2.00E-13	62.8
TcMYB12	AtMYB10	28.97	107	74	2	13	327	14	120	2.00E-13	62.4

TcMYB12	ATMYB4	28.68	136	95	4	22	423	15	146	2.00E-13	62.8
TcMYB12	ATMYB3	29.6	125	86	3	22	390	15	124	2.00E-13	62.4
TcMYB12	ATMYB94	29.29	99	69	1	4	297	9	107	3.00E-13	62.8
TcMYB12	ATMYB75	31.87	91	61	2	13	282	8	95	4.00E-13	61.6
TcMYB12	ATMYB90	32.97	91	60	2	13	282	8	95	4.00E-13	61.6
TcMYB12	AtMYB17	28.75	160	112	4	4	477	9	162	6.00E-13	61.6
TcMYB12	AtMYB113	32.97	91	60	2	13	282	8	95	7.00E-13	60.8
TcMYB12	ATMYB5	28.37	141	94	4	13	414	23	162	7.00E-13	60.8
TcMYB12	ATMYB96	28.28	99	70	1	4	297	9	107	9.00E-13	61.6
TcMYB12	ATMYB31	27.27	99	71	1	4	297	9	107	2.00E-12	60.5
TcMYB12	ATMYB92	32.61	92	60	2	13	282	12	103	2.00E-12	60.5
TcMYB12	AtMYB93	31.52	92	61	2	13	282	12	103	2.00E-12	60.5
TcMYB12	ATMYB123	26.36	110	79	2	22	345	17	126	3.00E-12	59.3
TcMYB12	AtMYB60	28.28	99	70	1	4	297	9	107	4.00E-12	58.9
TcMYB12	ATMYB91	28.12	128	89	3	28	402	7	125	5.00E-12	59.3
TcMYB12	AtMYB49	30.43	92	62	2	13	282	12	103	6.00E-12	58.9
TcMYB12	ATMYB30	24.81	133	99	2	13	408	12	141	6.00E-12	58.9
TcMYB12	AtMYB53	31.52	92	61	2	13	282	12	103	7.00E-12	58.5
TcMYB12	ATMYB121	26.32	133	92	4	22	402	30	160	7.00E-12	58.2
TcMYB12	AtMYB97	30.85	94	63	2	7	282	17	110	8.00E-12	58.9
TcMYB12	AtMYB9	30.43	92	62	2	13	282	12	103	8.00E-12	58.5
TcMYB12	AtMYB19	27.36	106	75	2	22	333	15	120	9.00E-12	57.8
TcMYB12	ATMYB95	21.93	187	144	3	13	567	12	196	1.00E-11	57.8
TcMYB12	AtMYB18	26.13	111	80	2	22	348	13	123	1.00E-11	57.8
TcMYB12	ATMYB34	32.61	92	60	2	13	282	12	103	1.00E-11	57.8
TcMYB12	MYB7	30.16	126	84	4	22	387	15	123	1.00E-11	57.4
TcMYB12	AtMYB50	27.05	122	87	3	13	372	12	128	1.00E-11	57.8
TcMYB12	AtMYB107	30.43	92	62	2	13	282	12	103	2.00E-11	57
TcMYB12	ATMYB13	30.53	95	64	2	4	282	9	103	2.00E-11	56.2
TcMYB12	AtMYB22	32.89	76	51	1	28	255	57	131	3.00E-11	56.2
TcMYB12	AtMYB22	45.45	44	23	1	169	297	52	95	2.00E-06	41.6
TcMYB12	ATMYB72	27.17	92	65	2	13	282	14	105	3.00E-11	56.6
TcMYB12	ATMYB65	30.53	95	64	2	4	282	38	132	3.00E-11	57.4
TcMYB12	ATMYB67	23.93	117	87	3	13	357	22	133	4.00E-11	56.2
TcMYB12	ATMYB120	30.85	94	63	2	7	282	24	117	4.00E-11	57
TcMYB12	AtMYB1	26.8	97	70	1	22	309	56	152	4.00E-11	56.6
TcMYB12	ATMYB33	31.52	92	61	2	13	282	32	123	5.00E-11	56.6
TcMYB12	AtMYB104	33.33	87	56	2	28	282	21	107	6.00E-11	56.2
TcMYB12	AtMYB32	30.34	89	60	2	22	282	15	103	6.00E-11	55.5
TcMYB12	ATMYB14	29.35	92	63	2	13	282	12	103	6.00E-11	55.1
TcMYB12	ATMYB102	29.35	92	63	2	13	282	12	103	7.00E-11	55.8
TcMYB12	AtMYB40	24.5	151	111	4	4	447	9	158	7.00E-11	55.1
TcMYB12	ATMYB87	31.18	93	61	3	4	273	9	101	7.00E-11	55.5
TcMYB12	AtMYB83	23.87	155	112	5	13	459	30	175	8.00E-11	55.5
TcMYB12	AtMYB51	29.35	92	63	2	13	282	13	104	9.00E-11	55.5
TcMYB12	AtMYB79	31.46	89	59	2	22	282	9	97	9.00E-11	54.7
TcMYB12	ATMYB101	32.98	94	59	3	13	282	18	109	9.00E-11	55.8
TcMYB12	AtMYB85	24.14	145	108	3	4	432	9	152	1.00E-10	54.7
TcMYB12	AtMYB76	26.04	192	133	8	13	561	12	195	1.00E-10	55.1
TcMYB12	ATMYB86	23.77	122	91	3	13	372	12	128	1.00E-10	55.1
TcMYB12	ATMYB61	24.03	154	115	4	13	468	12	149	1.00E-10	55.1
TcMYB12	ATMYB11	27.07	133	88	3	4	375	9	141	1.00E-10	54.7
TcMYB12	AtMYB27	28.26	92	64	2	13	282	9	100	2.00E-10	53.5
TcMYB12	AtMYB81	30.34	89	60	2	22	282	23	111	2.00E-10	54.7
TcMYB12	ATMYB29	24.68	158	105	4	13	444	12	168	2.00E-10	54.3
TcMYB12	ATMYB106	26.04	96	70	1	13	297	55	150	2.00E-10	54.3
TcMYB12	ATMYB63	26.35	148	105	5	13	444	14	144	3.00E-10	53.5
TcMYB12	ATMYB25	31.76	85	57	1	22	273	51	135	3.00E-10	53.9
TcMYB12	AtMYB6	27.42	124	88	3	22	387	15	123	3.00E-10	52.8
TcMYB12	ATMYB71	30.34	89	60	2	22	282	21	109	4.00E-10	52.8
TcMYB12	AtMYB41	29.35	92	63	2	13	282	12	103	5.00E-10	52.8
TcMYB12	ATMYB16	24.24	99	74	1	4	297	9	107	6.00E-10	52.8
TcMYB12	AtMYB74	29.35	92	63	2	13	282	13	104	8.00E-10	52.4
TcMYB12	ATMYB2	31.52	92	61	2	13	282	20	111	8.00E-10	52
TcMYB12	ATMYB58	24.06	133	99	2	13	405	14	146	8.00E-10	52
TcMYB12	MYB8	23.81	147	110	3	22	456	15	146	8.00E-10	51.2
TcMYB12	ATMYB55	25.18	139	90	4	13	387	12	135	8.00E-10	52.4
TcMYB12	ATMYB28	25.78	128	94	2	13	393	12	135	9.00E-10	52.4
TcMYB12	AtMYB47	26.83	82	59	1	13	255	12	93	1.00E-09	51.6
TcMYB12	ATMYB35	25.23	107	78	2	13	327	12	118	1.00E-09	51.6
TcMYB12	ATMYB111	29.47	95	65	2	4	282	9	103	1.00E-09	51.6
TcMYB12	ATMYB12	28.42	95	66	2	4	282	9	103	2.00E-09	51.6
TcMYB12	AtMYB103	27.17	92	65	2	13	282	12	103	2.00E-09	51.2
TcMYB12	AtMYB43	23.78	164	114	5	4	462	9	171	2.00E-09	50.8
TcMYB12	ATMYB37	32.22	90	58	3	13	273	12	101	2.00E-09	50.8
TcMYB12	ATMYB57	26.09	92	66	2	13	282	25	116	2.00E-09	49.7
TcMYB12	AtMYB36	33.33	90	57	3	13	273	12	101	3.00E-09	50.8
TcMYB12	AtMYB42	25.89	112	81	2	4	333	9	120	3.00E-09	50.4
TcMYB12	ATMYB59-3	30.77	91	59	3	22	282	11	99	3.00E-09	49.7
TcMYB12	ATMYB48	30.77	91	59	3	22	282	10	98	4.00E-09	49.7

TcMYB12	ATMYB26	25.74	101	64	2	13	282	12	112	5.00E-09	50.1
TcMYB12	ATMYB46	23.91	92	68	2	13	282	18	109	6.00E-09	49.3
TcMYB12	AtMYB112	29.17	96	66	2	1	282	28	123	6.00E-09	48.9
TcMYB12	ATMYB38	32.22	90	58	3	13	273	12	101	7.00E-09	49.3
TcMYB12	AtMYB62	26.09	92	66	2	13	282	19	110	9.00E-09	48.9
TcMYB12	AtMYB24	27.17	92	65	2	13	282	17	108	2.00E-08	47.4
TcMYB12	ATMYB68	31.11	90	59	3	13	273	12	101	5.00E-08	47
TcMYB12	ATMYB80	25.84	89	64	2	13	273	12	100	5.00E-08	46.6
TcMYB12	ATMYB3	27.17	92	65	2	13	282	20	111	6.00E-08	45.8
TcMYB12	AtMYB108	28.26	92	64	2	13	282	19	110	7.00E-08	46.2
TcMYB12	ATMYB84	24.59	122	77	4	13	333	12	133	1.00E-07	45.4
TcMYB12	AtMYB116	29.07	86	59	2	22	273	21	106	1.00E-07	45.1
TcMYB12	ATMYB78	24.24	99	74	2	13	306	26	120	3.00E-07	44.3
TcMYB12	AtMYB20	21.74	138	106	3	4	411	9	141	8.00E-07	42.7
TcMYB12	ATMYB99	25.78	128	93	4	88	465	45	154	2.00E-06	41.2
TcMYB13	ATMYB65	39.9	406	214	11	94	1221	35	422	7.00E-78	244
TcMYB13	ATMYB33	42.74	358	182	11	103	1107	29	378	8.00E-75	236
TcMYB13	ATMYB101	40.92	369	187	12	103	1116	15	344	5.00E-70	222
TcMYB13	AtMYB81	39.19	370	205	13	109	1158	19	364	1.00E-66	212
TcMYB13	AtMYB97	69.34	137	41	1	91	498	12	148	7.00E-65	206
TcMYB13	ATMYB120	38.3	389	234	8	100	1248	22	389	2.00E-63	206
TcMYB13	ATMYB92	65.38	104	36	0	112	423	12	115	5.00E-47	157
TcMYB13	ATMYB102	64.42	104	37	0	112	423	12	115	5.00E-47	158
TcMYB13	AtMYB74	63.46	104	38	0	112	423	13	116	8.00E-47	157
TcMYB13	AtMYB9	62.5	104	39	0	112	423	12	115	3.00E-46	155
TcMYB13	AtMYB49	36.7	267	156	8	112	873	12	250	4.00E-46	155
TcMYB13	ATMYB71	56.3	119	47	1	91	432	6	124	4.00E-46	153
TcMYB13	ATMYB3	45.24	168	92	2	112	615	12	153	4.00E-46	153
TcMYB13	AtMYB51	41.32	242	126	8	112	789	13	252	8.00E-46	155
TcMYB13	AtMYB17	63.46	104	38	0	112	423	12	115	8.00E-46	153
TcMYB13	AtMYB41	60.58	104	41	0	112	423	12	115	1.00E-45	152
TcMYB13	AtMYB53	62.5	104	39	0	112	423	12	115	2.00E-45	153
TcMYB13	AtMYB107	60.58	104	41	0	112	423	12	115	2.00E-45	153
TcMYB13	ATMYB34	40.79	228	125	9	112	765	12	214	3.00E-45	152
TcMYB13	ATMYB14	58.65	104	43	0	112	423	12	115	3.00E-45	150
TcMYB13	AtMYB79	60	105	42	0	115	429	7	111	4.00E-45	150
TcMYB13	ATMYB13	60.58	104	41	0	112	423	12	115	6.00E-45	149
TcMYB13	AtMYB24	55.36	112	46	1	97	420	8	119	9.00E-45	148
TcMYB13	AtMYB93	62.5	104	39	0	112	423	12	115	1.00E-44	152
TcMYB13	ATMYB15	47.77	157	82	2	112	582	12	158	2.00E-44	149
TcMYB13	ATMYB3	54.87	113	46	1	97	420	10	122	2.00E-44	147
TcMYB13	AtMYB6	61.76	102	39	0	118	423	14	115	3.00E-44	147
TcMYB13	MYB8	59.62	104	42	0	112	423	12	115	6.00E-44	145
TcMYB13	ATMYB57	54.63	108	49	0	106	429	23	130	8.00E-44	145
TcMYB13	ATMYB66	54.05	111	51	0	91	423	9	119	1.00E-43	145
TcMYB13	ATMYB106	53.28	122	57	1	58	423	43	158	2.00E-43	149
TcMYB13	ATMYB122	38.25	251	142	8	112	825	12	249	2.00E-43	148
TcMYB13	ATMYB95	45.18	166	85	3	112	591	12	172	3.00E-43	145
TcMYB13	ATMYB121	44.65	159	88	1	115	591	28	183	4.00E-43	145
TcMYB13	ATMYB28	36.78	242	143	5	103	798	9	224	4.00E-43	148
TcMYB13	MYB7	59.62	104	42	0	112	423	12	115	4.00E-43	145
TcMYB13	ATMYB16	59.62	104	42	0	112	423	12	115	5.00E-43	147
TcMYB13	AtMYB116	53.1	113	53	0	91	429	11	123	6.00E-43	145
TcMYB13	ATMYB94	58.65	104	43	0	112	423	12	115	1.00E-42	146
TcMYB13	AtMYB60	58.65	104	43	0	112	423	12	115	2.00E-42	144
TcMYB13	AtMYB43	37.5	232	126	7	112	750	12	236	2.00E-42	145
TcMYB13	ATMYB5	56.73	104	45	0	112	423	23	126	2.00E-42	143
TcMYB13	ATMYB86	57.55	106	45	0	112	429	12	117	2.00E-42	145
TcMYB13	AtMYB85	47.33	150	78	4	112	558	12	145	3.00E-42	143
TcMYB13	ATMYB29	58.49	106	44	0	103	420	9	114	3.00E-42	145
TcMYB13	ATMYB96	57.69	104	44	0	112	423	12	115	3.00E-42	145
TcMYB13	ATMYB4	60.78	102	40	0	118	423	14	115	3.00E-42	143
TcMYB13	ATMYB23	41.21	165	97	2	85	579	3	163	3.00E-42	141
TcMYB13	AtMYB50	58.65	104	43	0	112	423	12	115	3.00E-42	144
TcMYB13	AtMYB32	59.8	102	41	0	118	423	14	115	4.00E-42	143
TcMYB13	ATMYB63	58.65	104	43	0	112	423	14	117	4.00E-42	143
TcMYB13	AtMYB36	59.43	106	42	1	109	423	11	116	5.00E-42	144
TcMYB13	ATMYB30	57.55	106	45	0	106	423	10	115	6.00E-42	144
TcMYB13	AtMYB76	57.01	107	46	0	103	423	9	115	6.00E-42	144
TcMYB13	AtMYB42	56.73	104	45	0	112	423	12	115	9.00E-42	142
TcMYB13	ATMYB59-3	54.87	113	50	1	115	450	9	121	1.00E-41	140
TcMYB13	ATMYB87	58.49	106	43	1	109	423	11	116	1.00E-41	142
TcMYB13	ATMYB72	57.69	104	44	0	112	423	14	117	1.00E-41	142
TcMYB13	ATMYB61	57.69	104	44	0	112	423	12	115	2.00E-41	144
TcMYB13	ATMYB48	54.87	113	50	1	115	450	8	120	2.00E-41	140
TcMYB13	AtMYB20	54.72	106	48	0	112	429	12	117	2.00E-41	141
TcMYB13	ATMYB58	55.77	104	46	0	112	423	14	117	4.00E-41	140
TcMYB13	ATMYB11	56.73	104	45	0	112	423	12	115	5.00E-41	142
TcMYB13	AtMYB82	48.76	121	62	0	100	462	8	128	5.00E-41	137
TcMYB13	ATMYB12	55.77	104	46	0	112	423	12	115	6.00E-41	142

TcMYB13	ATMYB99	54.05	111	44	1	112	423	13	123	7.00E-41	139
TcMYB13	AtMYB62	53.77	106	49	0	112	429	19	124	7.00E-41	140
TcMYB13	ATMYB31	56.73	104	45	0	112	423	12	115	1.00E-40	140
TcMYB13	ATMYB46	55.77	104	46	0	112	423	18	121	1.00E-40	139
TcMYB13	AtMYB104	32.51	366	231	15	118	1167	18	338	2.00E-40	141
TcMYB13	ATMYB0	55.34	103	46	0	115	423	15	117	2.00E-40	137
TcMYB13	AtMYB47	42.44	172	86	4	112	588	12	183	2.00E-40	138
TcMYB13	AtMYB112	52.83	106	50	0	112	429	32	137	2.00E-40	137
TcMYB13	ATMYB123	54.81	104	47	0	112	423	14	117	2.00E-40	138
TcMYB13	ATMYB80	55.24	105	47	0	109	423	11	115	2.00E-40	139
TcMYB13	ATMYB68	59.43	106	42	1	109	423	11	116	2.00E-40	140
TcMYB13	ATMYB35	54.29	105	48	0	109	423	11	115	5.00E-40	138
TcMYB13	ATMYB84	58.49	106	43	1	109	423	11	116	6.00E-40	138
TcMYB13	AtMYB83	33.46	269	130	6	91	750	23	290	7.00E-40	139
TcMYB13	AtMYB103	42.07	164	86	3	112	576	12	163	1.00E-39	139
TcMYB13	AtMYB108	53.77	106	49	0	112	429	19	124	1.00E-39	137
TcMYB13	ATMYB38	43.29	164	89	3	109	588	11	152	1.00E-39	137
TcMYB13	ATMYB2	52.83	106	50	0	112	429	20	125	1.00E-39	136
TcMYB13	AtMYB10	55.77	104	46	0	112	423	14	117	2.00E-39	134
TcMYB13	ATMYB111	55.77	104	46	0	112	423	12	115	5.00E-39	136
TcMYB13	ATMYB37	56.19	105	45	1	112	423	12	116	5.00E-39	136
TcMYB13	ATMYB55	53.39	118	43	1	112	429	12	129	5.00E-39	136
TcMYB13	ATMYB67	50.93	108	53	0	100	423	18	125	8.00E-39	135
TcMYB13	ATMYB26	39.66	174	96	4	112	606	12	176	3.00E-38	135
TcMYB13	AtMYB40	47.33	131	68	1	112	501	12	142	3.00E-38	132
TcMYB13	AtMYB27	53.7	108	50	0	109	432	8	115	8.00E-38	130
TcMYB13	AtMYB114	47.27	110	58	0	94	423	2	111	2.00E-37	126
TcMYB13	ATMYB75	47.27	110	58	0	94	423	2	111	4.00E-36	126
TcMYB13	AtMYB45	54.9	102	46	0	115	420	19	120	5.00E-36	126
TcMYB13	AtMYB19	34.56	217	131	4	115	732	13	210	9.00E-36	125
TcMYB13	ATMYB78	45.83	120	51	1	112	429	26	145	4.00E-35	125
TcMYB13	ATMYB90	45.13	113	62	0	94	432	2	114	8.00E-35	122
TcMYB13	AtMYB113	48.08	104	54	0	112	423	8	111	1.00E-34	122
TcMYB13	AtMYB18	50.49	103	51	0	115	423	11	113	1.00E-32	117
TcMYB13	AtMYB109	53.51	114	50	3	91	423	45	156	3.00E-31	116
TcMYB13	AtMYB98	44.72	123	66	2	109	471	214	335	1.00E-30	115
TcMYB13	ATMYB119	49.12	114	57	3	94	432	97	207	1.00E-30	115
TcMYB13	ATMYB73	51.96	102	49	1	118	423	13	113	2.00E-30	112
TcMYB13	AtMYB70	50.94	106	52	1	118	435	13	117	3.00E-30	112
TcMYB13	AtMYB1	50.88	114	54	2	88	423	43	155	1.00E-29	112
TcMYB13	ATMYB77	49.53	107	54	1	118	438	6	111	1.00E-29	110
TcMYB13	ATMYB25	50.91	110	53	2	97	423	43	150	1.00E-29	111
TcMYB13	AtMYB64	50	106	52	3	118	432	105	207	2.00E-29	111
TcMYB13	ATMYB118	47.22	108	57	2	109	432	186	291	2.00E-28	108
TcMYB13	AtMYB115	47.06	102	53	2	118	420	158	257	5.00E-28	106
TcMYB13	ATMYB44	50	102	51	1	118	423	6	106	7.00E-28	105
TcMYB13	AtMYB117	36.36	154	97	3	112	570	96	243	2.00E-27	104
TcMYB13	AtMYB56	42.65	136	74	3	79	474	78	212	3.00E-27	103
TcMYB13	AtMYB100	46.23	106	56	2	112	426	24	127	4.00E-27	101
TcMYB13	ATMYB105	42.98	114	64	2	91	429	98	209	1.00E-26	102
TcMYB13	ATMYB54	45.71	105	56	2	118	429	6	108	5.00E-26	98.6
TcMYB13	ATMYB110	46.15	104	56	1	118	429	67	169	6.00E-26	99.8
TcMYB13	ATMYB52	44.64	112	61	2	118	450	5	114	8.00E-26	98.2
TcMYB13	ATMYB69	45.87	109	57	2	109	429	16	121	8.00E-26	98.2
TcMYB13	AtMYB22	40.95	105	62	2	112	426	52	154	4.00E-22	87.8
TcMYB13	ATMYB91	37.86	103	62	1	127	429	7	109	4.00E-21	86.7
TcMYB13	AtMYB89	39.81	103	59	2	121	420	57	155	3.00E-19	78.2
TcMYB13	AtMYB124	32.67	101	68	1	127	429	28	127	2.00E-16	73.2
TcMYB13	ATMYB88	31.37	102	69	2	127	429	33	132	9.00E-15	68.2
TcMYB14	AtMYB85	55.71	219	86	3	1	624	1	217	9.00E-86	250
TcMYB14	AtMYB20	47.72	285	124	6	1	780	1	277	7.00E-84	246
TcMYB14	AtMYB43	61.34	194	72	3	1	573	1	190	1.00E-83	247
TcMYB14	AtMYB42	78.63	131	28	0	1	393	1	131	1.00E-79	236
TcMYB14	AtMYB40	52.79	197	91	3	1	585	1	194	2.00E-72	216
TcMYB14	ATMYB99	71.21	132	31	1	4	378	3	134	3.00E-70	210
TcMYB14	ATMYB16	69.92	133	40	0	1	399	1	133	1.00E-69	211
TcMYB14	AtMYB107	70.23	131	39	0	1	393	1	131	2.00E-69	211
TcMYB14	AtMYB17	72.09	129	36	0	1	387	1	129	3.00E-69	209
TcMYB14	AtMYB50	55.61	187	82	4	1	558	1	178	2.00E-67	205
TcMYB14	ATMYB92	59.51	163	58	2	1	465	1	163	6.00E-67	204
TcMYB14	AtMYB9	67.94	131	42	0	1	393	1	131	7.00E-67	204
TcMYB14	AtMYB93	67.94	131	42	0	1	393	1	131	1.00E-66	204
TcMYB14	AtMYB53	67.18	131	43	0	1	393	1	131	5.00E-66	201
TcMYB14	ATMYB61	44.8	250	132	4	1	732	1	226	8.00E-66	202
TcMYB14	AtMYB6	63.77	138	50	0	1	414	1	138	9.00E-66	198
TcMYB14	ATMYB102	70.31	128	38	0	1	384	1	128	1.00E-65	202
TcMYB14	AtMYB41	67.65	136	44	0	1	408	1	136	1.00E-65	199
TcMYB14	ATMYB106	64.75	139	49	0	13	429	48	186	8.00E-65	201
TcMYB14	AtMYB74	69.77	129	38	1	1	384	1	129	2.00E-64	198
TcMYB14	AtMYB32	59.86	147	59	1	1	441	1	146	3.00E-64	196

TcMYB14	ATMYB86	68.22	129	41	0	1	387	1	129	7.00E-64	197
TcMYB14	ATMYB4	53.23	186	86	3	1	555	1	170	2.00E-63	194
TcMYB14	MYB7	63.64	132	48	0	1	396	1	132	2.00E-63	193
TcMYB14	ATMYB5	60.56	142	56	0	13	438	16	157	1.00E-62	191
TcMYB14	ATMYB28	61.64	146	55	1	1	435	1	146	2.00E-62	194
TcMYB14	MYB8	59.57	141	57	0	1	423	1	141	3.00E-62	188
TcMYB14	AtMYB49	40.6	266	151	3	1	777	1	252	5.00E-62	191
TcMYB14	ATMYB13	48	200	101	2	1	591	1	183	7.00E-62	189
TcMYB14	ATMYB80	65.38	130	45	0	1	390	1	130	7.00E-62	191
TcMYB14	ATMYB35	41.54	260	150	5	1	774	1	247	1.00E-61	191
TcMYB14	ATMYB3	62.88	132	49	0	1	396	1	132	2.00E-61	188
TcMYB14	ATMYB29	60.14	143	57	0	1	429	1	143	2.00E-60	188
TcMYB14	AtMYB76	46.23	199	107	0	1	597	1	199	1.00E-59	186
TcMYB14	ATMYB67	41.7	247	143	6	1	738	12	240	3.00E-59	184
TcMYB14	ATMYB14	66.1	118	40	0	1	354	1	118	2.00E-58	180
TcMYB14	AtMYB51	54.32	162	69	2	1	471	1	162	2.00E-58	183
TcMYB14	ATMYB15	68.1	116	37	0	1	348	1	116	9.00E-58	179
TcMYB14	ATMYB111	56.08	148	64	1	1	441	1	148	3.00E-57	180
TcMYB14	ATMYB12	67.24	116	38	0	1	348	1	116	3.00E-57	181
TcMYB14	ATMYB58	47	200	106	3	4	603	4	168	4.00E-57	177
TcMYB14	ATMYB34	58.96	134	55	0	1	402	1	134	6.00E-57	177
TcMYB14	AtMYB10	44.86	214	110	5	7	624	5	212	2.00E-56	174
TcMYB14	ATMYB55	60.14	143	45	1	1	393	1	143	3.00E-56	177
TcMYB14	ATMYB63	49.16	179	90	2	4	537	4	177	1.00E-55	174
TcMYB14	ATMYB11	65.52	116	40	0	1	348	1	116	1.00E-55	176
TcMYB14	ATMYB72	58.33	144	60	1	4	435	4	137	1.00E-55	174
TcMYB14	ATMYB122	54.49	156	71	1	1	468	1	155	5.00E-55	174
TcMYB14	AtMYB47	36.7	267	137	5	1	705	1	267	7.00E-55	171
TcMYB14	AtMYB60	62.3	122	46	0	1	366	1	122	3.00E-54	170
TcMYB14	ATMYB30	57.24	145	59	1	1	426	1	145	5.00E-54	171
TcMYB14	AtMYB103	61.86	118	45	0	1	354	1	118	7.00E-54	172
TcMYB14	ATMYB94	52.26	155	74	1	1	465	1	143	2.00E-53	170
TcMYB14	ATMYB96	45.07	213	106	3	1	606	1	213	1.00E-52	168
TcMYB14	ATMYB31	63.03	119	44	0	1	357	1	119	2.00E-52	167
TcMYB14	ATMYB26	60.32	126	41	1	1	351	1	126	3.00E-52	167
TcMYB14	ATMYB95	59.38	128	52	0	1	384	1	128	8.00E-52	164
TcMYB14	ATMYB3	38.97	213	123	3	34	651	20	224	1.00E-50	159
TcMYB14	AtMYB24	42.42	198	114	3	34	627	17	205	1.00E-49	156
TcMYB14	AtMYB83	36.43	269	154	6	22	777	26	281	1.00E-49	160
TcMYB14	ATMYB46	56.2	137	59	1	34	441	18	154	2.00E-49	158
TcMYB14	AtMYB116	57.89	114	48	0	37	378	19	132	4.00E-48	154
TcMYB14	ATMYB84	60.17	118	46	1	1	351	1	118	4.00E-48	155
TcMYB14	AtMYB79	42.51	207	91	3	37	573	7	209	5.00E-48	154
TcMYB14	AtMYB62	40.59	202	120	3	34	639	19	197	6.00E-48	154
TcMYB14	ATMYB38	51.39	144	68	2	1	426	1	144	9.00E-48	154
TcMYB14	AtMYB108	60	115	46	0	22	366	15	129	1.00E-47	154
TcMYB14	ATMYB68	59.32	118	47	1	1	351	1	118	2.00E-47	155
TcMYB14	ATMYB37	56.91	123	52	1	1	366	1	123	3.00E-47	154
TcMYB14	AtMYB36	45.98	174	93	3	1	519	1	154	4.00E-47	153
TcMYB14	ATMYB101	45.25	179	97	4	31	564	17	184	6.00E-47	156
TcMYB14	ATMYB57	40.7	199	117	4	34	627	25	193	6.00E-47	149
TcMYB14	AtMYB19	58.12	117	49	1	4	354	5	118	9.00E-47	150
TcMYB14	ATMYB71	62.86	105	39	0	31	345	17	121	9.00E-47	150
TcMYB14	ATMYB87	38.11	244	140	7	1	699	1	228	2.00E-46	151
TcMYB14	ATMYB48	37.61	234	146	4	31	732	6	215	5.00E-46	148
TcMYB14	AtMYB112	53.54	127	51	2	25	381	29	155	1.00E-45	147
TcMYB14	ATMYB120	43.65	181	97	2	34	561	26	205	1.00E-44	151
TcMYB14	ATMYB59-3	61.17	103	40	0	37	345	9	111	2.00E-44	143
TcMYB14	AtMYB18	39.8	201	119	4	37	633	11	204	1.00E-43	143
TcMYB14	ATMYB121	59.62	104	42	0	31	342	26	129	1.00E-43	142
TcMYB14	ATMYB33	57.41	108	46	0	22	345	28	135	2.00E-43	147
TcMYB14	ATMYB123	42.46	179	97	3	34	552	14	181	9.00E-43	140
TcMYB14	ATMYB65	57.69	104	44	0	34	345	41	144	1.00E-42	145
TcMYB14	ATMYB23	39.33	178	92	3	37	522	13	190	2.00E-42	138
TcMYB14	ATMYB2	52.59	116	55	0	34	381	20	135	8.00E-42	138
TcMYB14	ATMYB66	57.69	104	44	0	37	348	17	120	9.00E-42	135
TcMYB14	AtMYB97	53.57	112	52	1	28	363	17	127	9.00E-41	138
TcMYB14	AtMYB27	58.65	104	43	0	34	345	9	112	3.00E-40	133
TcMYB14	ATMYB0	54.29	105	48	0	37	351	15	119	4.00E-40	132
TcMYB14	AtMYB81	52.21	113	54	1	25	363	17	128	5.00E-40	137
TcMYB14	AtMYB114	56.19	105	46	0	31	345	7	111	5.00E-40	129
TcMYB14	ATMYB78	48.06	129	53	1	22	366	22	150	7.00E-40	134
TcMYB14	AtMYB82	54.81	104	47	0	34	345	12	115	1.00E-39	130
TcMYB14	AtMYB45	53.92	102	47	0	37	342	19	120	2.00E-39	131
TcMYB14	ATMYB75	56.19	105	46	0	31	345	7	111	2.00E-38	128
TcMYB14	ATMYB90	57.14	105	45	0	31	345	7	111	3.00E-38	128
TcMYB14	AtMYB113	54.29	105	48	0	31	345	7	111	8.00E-37	124
TcMYB14	ATMYB119	33.7	181	119	4	34	573	103	265	1.00E-29	108
TcMYB14	AtMYB104	46.88	96	51	0	40	327	18	113	1.00E-28	105
TcMYB14	AtMYB64	47.42	97	50	2	40	327	105	199	1.00E-28	105

TcMYB14	AtMYB109	35.96	178	89	5	40	498	56	230	4.00E-27	101
TcMYB14	AtMYB98	42.34	111	63	2	25	354	212	320	5.00E-27	101
TcMYB14	ATMYB73	46.6	103	54	2	40	345	13	113	7.00E-27	99.8
TcMYB14	ATMYB25	47.57	103	53	2	40	345	50	150	3.00E-26	98.6
TcMYB14	AtMYB70	46.6	103	54	2	40	345	13	113	4.00E-26	97.4
TcMYB14	ATMYB118	43.7	119	64	3	7	354	176	292	5.00E-26	99
TcMYB14	ATMYB118	24.68	77	51	3	151	360	169	243	8.00E-05	35.8
TcMYB14	ATMYB77	43.69	103	57	2	40	345	6	106	7.00E-26	96.7
TcMYB14	ATMYB44	43.69	103	57	2	40	345	6	106	3.00E-25	95.1
TcMYB14	AtMYB1	43.81	105	58	2	40	351	55	157	2.00E-24	94
TcMYB14	AtMYB1	31.03	58	39	1	184	354	50	107	6.00E-04	33.1
TcMYB14	AtMYB115	41.67	96	56	1	40	327	158	252	5.00E-24	92.4
TcMYB14	AtMYB100	26.58	222	138	7	25	615	21	234	4.00E-23	87.8
TcMYB14	ATMYB105	40.74	108	63	2	25	345	102	207	5.00E-23	89.4
TcMYB14	ATMYB52	40.78	103	60	2	40	345	5	105	7.00E-22	84.7
TcMYB14	AtMYB117	40.95	105	61	2	34	345	96	198	8.00E-22	86.3
TcMYB14	ATMYB54	40.78	103	60	2	40	345	6	106	9.00E-22	84.3
TcMYB14	AtMYB56	37.25	102	64	1	40	345	93	193	9.00E-20	80.1
TcMYB14	ATMYB91	36.63	101	62	1	49	345	7	107	2.00E-19	79.7
TcMYB14	ATMYB110	35.58	104	66	2	40	348	67	168	3.00E-19	78.6
TcMYB14	ATMYB69	34.21	114	74	3	7	345	13	119	6.00E-19	76.6
TcMYB14	AtMYB22	35.29	102	66	1	25	330	49	148	1.00E-18	75.9
TcMYB14	ATMYB88	37.37	99	62	1	49	345	33	130	1.00E-16	71.6
TcMYB14	AtMYB89	30.91	110	76	1	43	372	57	165	2.00E-16	68.6
TcMYB14	AtMYB124	37.37	99	62	1	49	345	28	125	4.00E-16	70.1
TcMYB15	ATMYB105	78.63	131	28	1	124	516	90	219	1.00E-70	218
TcMYB15	AtMYB117	70.55	146	41	1	85	516	65	210	5.00E-70	217
TcMYB15	AtMYB56	59.51	163	66	0	106	594	69	231	9.00E-67	207
TcMYB15	ATMYB52	76.32	114	27	0	169	510	2	115	5.00E-64	198
TcMYB15	ATMYB54	65.97	144	49	1	169	600	3	144	6.00E-64	197
TcMYB15	ATMYB110	66.67	135	45	1	109	513	45	178	1.00E-61	194
TcMYB15	ATMYB69	73.58	106	28	0	172	489	17	122	4.00E-56	177
TcMYB15	AtMYB89	52.14	140	67	1	76	495	33	161	4.00E-47	152
TcMYB15	AtMYB109	45.89	146	79	2	61	498	42	162	6.00E-41	142
TcMYB15	ATMYB25	55.24	105	47	0	178	492	50	154	7.00E-40	138
TcMYB15	ATMYB44	55.45	101	45	0	178	480	6	106	9.00E-39	134
TcMYB15	AtMYB70	55.45	101	45	0	178	480	13	113	1.00E-37	131
TcMYB15	AtMYB1	45.39	141	77	2	61	483	26	156	7.00E-37	130
TcMYB15	ATMYB73	52.48	101	48	0	178	480	13	113	1.00E-36	128
TcMYB15	ATMYB77	52.48	101	48	0	178	480	6	106	6.00E-36	126
TcMYB15	ATMYB119	42.07	145	79	2	79	498	73	216	3.00E-32	119
TcMYB15	ATMYB66	34.22	187	108	5	178	693	18	203	9.00E-32	112
TcMYB15	AtMYB64	44.44	135	75	1	178	582	105	227	2.00E-30	114
TcMYB15	ATMYB23	48.04	102	52	1	178	480	14	115	1.00E-29	107
TcMYB15	ATMYB118	47.17	106	56	0	178	495	189	294	6.00E-29	109
TcMYB15	ATMYB67	45.31	128	63	3	130	492	3	129	1.00E-28	106
TcMYB15	ATMYB0	47.06	102	53	1	178	480	16	117	1.00E-28	104
TcMYB15	AtMYB115	43.12	109	62	0	151	477	149	257	1.00E-28	107
TcMYB15	ATMYB15	35.75	179	98	5	178	663	14	187	2.00E-28	105
TcMYB15	AtMYB10	46.3	108	56	2	178	495	16	122	2.00E-28	104
TcMYB15	AtMYB100	45.45	99	53	2	184	477	28	125	3.00E-28	103
TcMYB15	AtMYB103	47.01	117	60	2	178	522	14	129	3.00E-28	106
TcMYB15	ATMYB63	46.3	108	56	2	178	495	16	122	3.00E-28	105
TcMYB15	ATMYB121	41.26	143	77	3	160	567	23	164	5.00E-28	104
TcMYB15	AtMYB19	47.12	104	53	2	178	483	14	116	8.00E-28	103
TcMYB15	AtMYB27	44.25	113	61	3	178	510	11	120	8.00E-28	102
TcMYB15	AtMYB98	42.37	118	68	0	178	531	217	334	2.00E-27	105
TcMYB15	AtMYB18	37.31	134	82	2	178	573	12	144	4.00E-27	102
TcMYB15	ATMYB58	36.03	136	86	1	178	582	16	151	4.00E-27	101
TcMYB15	AtMYB51	47.06	102	53	1	178	480	15	116	4.00E-27	103
TcMYB15	AtMYB104	39.68	126	75	1	178	552	18	143	5.00E-27	103
TcMYB15	AtMYB45	34.97	163	95	4	178	633	20	181	6.00E-27	100
TcMYB15	AtMYB82	46.08	102	54	1	178	480	14	115	1.00E-26	98.6
TcMYB15	ATMYB13	38.82	152	83	5	178	603	14	164	2.00E-26	99.4
TcMYB15	ATMYB80	46.6	103	53	2	178	480	14	115	2.00E-26	100
TcMYB15	AtMYB97	38.73	142	86	2	178	600	21	159	3.00E-26	101
TcMYB15	ATMYB35	39.39	132	79	2	178	570	14	143	4.00E-26	99.8
TcMYB15	ATMYB33	34.36	163	106	2	85	570	10	165	5.00E-26	102
TcMYB15	ATMYB72	44.04	109	59	2	178	498	16	123	6.00E-26	99
TcMYB15	AtMYB40	45.71	105	55	2	178	486	14	117	8.00E-26	97.8
TcMYB15	AtMYB81	41.22	131	76	2	115	504	9	131	9.00E-26	100
TcMYB15	ATMYB101	41.73	127	72	3	157	531	13	134	1.00E-25	100
TcMYB15	AtMYB53	47.57	103	52	2	178	480	14	115	1.00E-25	98.6
TcMYB15	ATMYB59-3	37.93	145	88	3	178	606	10	152	1.00E-25	96.7
TcMYB15	ATMYB48	44.86	107	57	2	178	492	9	114	2.00E-25	96.7
TcMYB15	ATMYB46	40	115	67	2	160	498	14	127	2.00E-25	97.1
TcMYB15	AtMYB49	45.1	102	55	1	178	480	14	115	3.00E-25	97.4
TcMYB15	AtMYB41	44.12	102	56	1	178	480	14	115	3.00E-25	96.7
TcMYB15	AtMYB36	40	140	82	5	178	591	14	142	4.00E-25	97.4
TcMYB15	ATMYB68	37.76	143	84	4	178	591	14	152	4.00E-25	97.8

TcMYB15	ATMYB95	43.69	103	56	2	178	480	14	115	5.00E-25	95.9
TcMYB15	ATMYB122	46.88	96	50	1	178	462	14	109	5.00E-25	97.1
TcMYB15	ATMYB120	36.91	149	93	3	61	504	7	137	5.00E-25	99
TcMYB15	ATMYB34	41.38	116	67	2	148	492	9	119	5.00E-25	96.3
TcMYB15	AtMYB79	40.8	125	72	2	178	546	8	131	6.00E-25	95.5
TcMYB15	ATMYB65	35	140	86	2	100	504	13	152	6.00E-25	99
TcMYB15	ATMYB5	42.45	106	60	1	178	492	25	130	8.00E-25	94.7
TcMYB15	AtMYB114	40.78	103	60	1	178	483	10	112	8.00E-25	91.7
TcMYB15	ATMYB86	42.99	107	59	2	178	492	14	119	9.00E-25	96.7
TcMYB15	AtMYB32	38.93	131	78	2	178	564	14	143	1.00E-24	95.1
TcMYB15	ATMYB3	45.63	103	54	2	178	480	14	115	1.00E-24	94.7
TcMYB15	MYB8	42.16	102	58	1	178	480	14	115	1.00E-24	93.6
TcMYB15	ATMYB61	37.93	145	87	4	178	603	14	151	1.00E-24	96.7
TcMYB15	AtMYB107	45.1	102	55	1	178	480	14	115	1.00E-24	95.9
TcMYB15	ATMYB92	46.6	103	53	2	178	480	14	115	1.00E-24	95.9
TcMYB15	AtMYB9	45.1	102	55	1	178	480	14	115	1.00E-24	95.9
TcMYB15	ATMYB71	39.84	123	72	2	178	540	20	141	2.00E-24	94.4
TcMYB15	ATMYB84	43.69	103	56	2	178	480	14	116	2.00E-24	95.1
TcMYB15	AtMYB116	37.88	132	80	3	148	537	13	140	2.00E-24	94.4
TcMYB15	ATMYB14	42.61	115	64	2	178	516	14	127	2.00E-24	93.6
TcMYB15	AtMYB74	44.66	103	55	2	178	480	15	116	3.00E-24	94.7
TcMYB15	ATMYB123	36.55	145	91	4	178	609	16	141	4.00E-24	93.2
TcMYB15	ATMYB4	44.66	103	55	2	178	480	14	115	4.00E-24	93.6
TcMYB15	ATMYB55	33.14	175	84	5	178	603	14	185	4.00E-24	94.7
TcMYB15	AtMYB22	32.39	142	90	3	103	510	23	163	6.00E-24	92.4
TcMYB15	AtMYB50	43.69	103	56	2	178	480	14	115	6.00E-24	93.6
TcMYB15	ATMYB2	47.37	95	49	1	178	459	22	116	9.00E-24	92.4
TcMYB15	ATMYB28	43.4	106	59	1	178	492	14	119	9.00E-24	94
TcMYB15	AtMYB83	41.67	108	62	1	178	498	32	139	1.00E-23	93.6
TcMYB15	AtMYB17	44.66	103	55	2	178	480	14	115	1.00E-23	92.8
TcMYB15	ATMYB75	40.78	103	60	1	178	483	10	112	1.00E-23	91.7
TcMYB15	AtMYB47	40.78	103	59	2	178	480	14	115	1.00E-23	92
TcMYB15	ATMYB106	44.66	103	55	2	178	480	57	158	1.00E-23	94
TcMYB15	AtMYB6	42.72	103	57	2	178	480	14	115	1.00E-23	91.3
TcMYB15	AtMYB112	41.44	111	64	2	130	459	19	128	1.00E-23	91.3
TcMYB15	ATMYB90	34.9	149	93	2	178	612	10	158	1.00E-23	91.3
TcMYB15	ATMYB94	41.67	108	62	1	178	498	14	121	2.00E-23	92.8
TcMYB15	ATMYB30	36.69	139	87	2	178	591	14	146	2.00E-23	92.4
TcMYB15	ATMYB102	44.66	103	55	2	178	480	14	115	3.00E-23	92.4
TcMYB15	AtMYB62	34.87	152	87	4	178	597	21	171	3.00E-23	91.3
TcMYB15	MYB7	41.75	103	58	2	178	480	14	115	4.00E-23	90.5
TcMYB15	AtMYB93	43.14	102	57	1	178	480	14	115	4.00E-23	92
TcMYB15	ATMYB38	43.69	103	56	2	178	480	14	116	5.00E-23	90.9
TcMYB15	ATMYB16	42.16	102	58	1	178	480	14	115	5.00E-23	91.3
TcMYB15	ATMYB96	38.06	134	80	2	178	570	14	147	5.00E-23	91.7
TcMYB15	AtMYB60	37.14	140	78	3	178	567	14	153	6.00E-23	90.1
TcMYB15	AtMYB43	39.2	125	74	2	178	546	14	137	7.00E-23	90.9
TcMYB15	AtMYB76	45.1	102	55	1	178	480	14	115	8.00E-23	90.9
TcMYB15	ATMYB31	44.12	102	56	1	178	480	14	115	1.00E-22	90.5
TcMYB15	AtMYB113	38.68	106	64	1	178	492	10	115	1.00E-22	88.6
TcMYB15	ATMYB57	29.89	174	120	4	178	693	27	188	1.00E-22	87.4
TcMYB15	ATMYB3	41.28	109	63	2	178	501	22	129	2.00E-22	87.8
TcMYB15	AtMYB108	43.52	108	59	3	142	459	12	115	2.00E-22	89.7
TcMYB15	ATMYB111	37.14	140	87	2	178	594	14	151	2.00E-22	89.7
TcMYB15	ATMYB29	45.83	96	51	1	178	462	14	109	3.00E-22	89.4
TcMYB15	ATMYB37	40	110	63	2	178	498	14	122	4.00E-22	88.6
TcMYB15	AtMYB20	41.51	106	60	2	178	489	14	118	8.00E-22	87
TcMYB15	AtMYB42	42.72	103	57	2	178	480	14	115	9.00E-22	87
TcMYB15	ATMYB11	34.56	136	76	2	178	546	14	149	2.00E-21	87
TcMYB15	AtMYB85	42.72	103	57	2	178	480	14	115	2.00E-21	85.5
TcMYB15	AtMYB24	42.11	95	54	1	178	459	19	113	2.00E-21	84.3
TcMYB15	ATMYB26	39.64	111	57	2	178	480	14	124	2.00E-21	87
TcMYB15	ATMYB12	40.2	102	60	1	178	480	14	115	4.00E-21	86.3
TcMYB15	ATMYB88	36.89	103	65	0	187	495	33	135	6.00E-21	86.7
TcMYB15	ATMYB87	38.83	103	61	2	178	480	14	116	7.00E-21	84.7
TcMYB15	AtMYB124	36.89	103	65	0	187	495	28	130	9.00E-21	85.9
TcMYB15	ATMYB99	42.73	110	54	4	178	480	15	123	3.00E-20	82
TcMYB15	ATMYB91	35.9	117	72	1	178	519	4	120	6.00E-20	82.8
TcMYB15	ATMYB78	34.51	142	78	4	79	459	2	136	1.00E-19	81.3
TcMYB16	AtMYB20	84.62	130	20	0	1	390	1	130	2.00E-84	252
TcMYB16	AtMYB43	75.69	144	35	0	1	432	1	144	9.00E-83	249
TcMYB16	AtMYB42	65	180	62	2	1	537	1	179	1.00E-82	248
TcMYB16	AtMYB85	58.14	215	73	4	1	594	1	214	1.00E-82	247
TcMYB16	AtMYB40	56.04	207	88	3	1	612	1	202	1.00E-77	234
TcMYB16	AtMYB107	75.19	133	33	0	1	399	1	133	2.00E-71	219
TcMYB16	AtMYB9	72.93	133	36	0	1	399	1	133	1.00E-69	216
TcMYB16	ATMYB16	60.36	169	67	3	1	507	1	166	1.00E-69	215
TcMYB16	ATMYB99	58.52	176	57	2	4	483	3	178	2.00E-69	212
TcMYB16	AtMYB93	45.29	276	134	4	1	777	1	271	4.00E-69	215
TcMYB16	AtMYB17	70.54	129	38	0	1	387	1	129	4.00E-68	211

TcMYB16	ATMYB102	70.99	131	38	0	1	393	1	131	1.00E-66	208
TcMYB16	AtMYB6	69.47	131	40	0	1	393	1	131	1.00E-66	204
TcMYB16	ATMYB106	53.8	184	81	3	13	552	48	228	2.00E-66	209
TcMYB16	AtMYB41	49.32	219	94	2	1	606	1	219	4.00E-66	205
TcMYB16	ATMYB61	55.85	188	71	2	1	528	1	188	5.00E-66	207
TcMYB16	ATMYB92	69.47	131	40	0	1	393	1	131	6.00E-66	206
TcMYB16	AtMYB53	68.42	133	42	0	1	399	1	133	6.00E-66	205
TcMYB16	AtMYB50	49.25	201	101	2	1	600	1	194	4.00E-65	203
TcMYB16	ATMYB4	67.18	131	43	0	1	393	1	131	6.00E-65	202
TcMYB16	MYB7	66.41	131	44	0	1	393	1	131	2.00E-64	200
TcMYB16	AtMYB74	70.45	132	38	1	1	393	1	132	3.00E-64	201
TcMYB16	AtMYB32	55.25	181	81	3	1	543	1	171	5.00E-64	199
TcMYB16	MYB8	67.18	131	43	0	1	393	1	131	6.00E-64	197
TcMYB16	ATMYB86	66.67	138	45	1	1	411	1	138	1.00E-63	201
TcMYB16	ATMYB3	65.41	133	46	0	1	399	1	133	7.00E-63	196
TcMYB16	ATMYB15	50.49	206	81	4	1	555	1	200	4.00E-62	194
TcMYB16	ATMYB5	66.4	125	42	0	13	387	16	140	1.00E-61	192
TcMYB16	ATMYB80	59.86	147	55	1	1	429	1	147	1.00E-61	194
TcMYB16	ATMYB28	58.44	154	64	1	1	462	1	151	4.00E-61	194
TcMYB16	AtMYB76	49.75	201	101	3	1	603	1	187	1.00E-60	192
TcMYB16	AtMYB49	67.97	128	41	0	1	384	1	128	5.00E-60	190
TcMYB16	ATMYB29	52.06	194	81	4	1	546	1	188	5.00E-60	191
TcMYB16	ATMYB13	49.22	193	94	2	1	567	1	175	9.00E-60	187
TcMYB16	ATMYB35	58.5	147	60	1	1	438	1	147	2.00E-59	189
TcMYB16	ATMYB122	44.25	226	111	1	1	633	1	226	8.00E-59	187
TcMYB16	AtMYB51	50.77	195	95	3	1	582	1	193	2.00E-58	187
TcMYB16	ATMYB55	51.31	191	77	3	1	525	1	190	3.00E-58	186
TcMYB16	ATMYB14	69.49	118	36	0	1	354	1	118	1.00E-57	182
TcMYB16	ATMYB34	57.33	150	59	1	1	435	1	150	2.00E-57	182
TcMYB16	ATMYB111	68.1	116	37	0	1	348	1	116	3.00E-57	184
TcMYB16	ATMYB12	44.2	224	79	3	1	534	1	224	3.00E-57	184
TcMYB16	ATMYB67	46.7	182	97	1	16	561	16	194	4.00E-57	182
TcMYB16	ATMYB11	45.5	222	116	4	1	651	1	214	6.00E-57	183
TcMYB16	ATMYB58	69.57	115	35	0	4	348	4	118	4.00E-56	179
TcMYB16	AtMYB10	56.16	146	64	0	7	444	5	150	5.00E-56	177
TcMYB16	ATMYB72	40.07	287	129	7	4	735	4	289	1.00E-55	178
TcMYB16	AtMYB47	43.72	215	108	3	1	606	1	214	2.00E-55	177
TcMYB16	ATMYB63	68.7	115	36	0	4	348	4	118	4.00E-55	177
TcMYB16	ATMYB96	41.3	247	140	6	1	726	1	206	9.00E-55	177
TcMYB16	ATMYB94	45.16	217	117	5	1	645	1	194	2.00E-54	176
TcMYB16	AtMYB103	48.6	179	92	1	1	537	1	178	3.00E-54	177
TcMYB16	ATMYB30	55.86	145	64	1	1	435	1	143	3.00E-54	175
TcMYB16	AtMYB60	63.11	122	45	0	1	366	1	122	5.00E-54	173
TcMYB16	ATMYB31	64.66	116	41	0	1	348	1	116	6.00E-52	169
TcMYB16	ATMYB95	41.67	216	111	4	1	603	1	215	5.00E-51	165
TcMYB16	ATMYB26	63.2	125	37	1	1	348	1	125	8.00E-51	167
TcMYB16	ATMYB46	67.59	108	35	0	34	357	18	125	2.00E-49	161
TcMYB16	AtMYB83	65.45	110	38	0	22	351	26	135	3.00E-48	160
TcMYB16	ATMYB84	61.54	117	44	1	1	348	1	117	2.00E-47	157
TcMYB16	AtMYB19	66.99	103	34	0	37	345	13	115	2.00E-47	155
TcMYB16	ATMYB71	43.28	201	114	2	31	633	17	196	6.00E-47	154
TcMYB16	AtMYB108	39.41	203	112	2	22	597	15	216	2.00E-46	155
TcMYB16	ATMYB3	44.39	187	98	3	34	576	20	198	3.00E-46	151
TcMYB16	AtMYB79	66.67	105	35	0	37	351	7	111	6.00E-46	152
TcMYB16	ATMYB68	60.68	117	45	1	1	348	1	117	6.00E-46	155
TcMYB16	AtMYB36	53.1	145	67	3	1	432	1	141	6.00E-46	154
TcMYB16	ATMYB57	57.76	116	49	0	34	381	25	140	9.00E-46	149
TcMYB16	AtMYB62	43.98	191	105	3	34	600	19	189	1.00E-45	152
TcMYB16	ATMYB37	45.11	184	87	5	1	510	1	182	1.00E-45	153
TcMYB16	ATMYB87	58.97	117	47	1	1	348	1	117	2.00E-45	152
TcMYB16	ATMYB38	46.06	165	82	3	1	474	1	165	2.00E-45	151
TcMYB16	ATMYB101	45.56	180	98	4	31	570	17	185	5.00E-45	155
TcMYB16	AtMYB18	65.05	103	36	0	37	345	11	113	1.00E-44	149
TcMYB16	AtMYB112	57.02	114	49	0	25	366	29	142	1.00E-44	147
TcMYB16	AtMYB116	56.52	115	50	0	37	381	19	133	3.00E-44	148
TcMYB16	ATMYB33	59.65	114	46	1	22	363	28	140	1.00E-43	151
TcMYB16	ATMYB120	61.68	107	41	0	34	354	26	132	2.00E-43	151
TcMYB16	ATMYB121	61.68	107	41	0	31	351	26	132	3.00E-43	145
TcMYB16	AtMYB24	59.63	109	44	0	34	360	17	125	3.00E-43	143
TcMYB16	AtMYB81	47.3	148	78	2	25	468	17	162	6.00E-43	148
TcMYB16	ATMYB65	60	110	44	1	34	363	41	149	2.00E-42	149
TcMYB16	ATMYB23	59.62	104	42	0	37	348	13	116	2.00E-42	141
TcMYB16	ATMYB66	59.62	104	42	0	37	348	17	120	2.00E-42	140
TcMYB16	ATMYB0	58.65	104	43	0	37	348	15	118	4.00E-42	140
TcMYB16	ATMYB75	40.29	206	111	3	31	612	7	212	5.00E-42	141
TcMYB16	ATMYB2	38.58	197	89	3	34	528	20	216	9.00E-42	141
TcMYB16	ATMYB123	58.88	107	44	0	34	354	14	120	1.00E-41	140
TcMYB16	AtMYB82	41.71	175	87	4	34	513	12	186	1.00E-41	138
TcMYB16	AtMYB114	56.3	119	44	1	31	363	7	125	2.00E-41	135
TcMYB16	AtMYB97	59.05	105	43	0	28	342	17	121	4.00E-41	142

TcMYB16	ATMYB48	60	105	42	0	31	345	6	110	4.00E-41	139
TcMYB16	ATMYB59-3	61.17	103	40	0	37	345	9	111	5.00E-41	138
TcMYB16	AtMYB45	57.84	102	43	0	37	342	19	120	5.00E-41	139
TcMYB16	ATMYB90	60.95	105	41	0	31	345	7	111	2.00E-40	137
TcMYB16	AtMYB27	57.69	104	44	0	34	345	9	112	1.00E-39	134
TcMYB16	AtMYB113	42.41	191	96	4	1	531	1	186	7.00E-39	132
TcMYB16	ATMYB78	48.06	129	53	1	22	366	22	150	8.00E-38	132
TcMYB16	AtMYB64	36.51	189	114	5	40	588	105	288	5.00E-30	112
TcMYB16	AtMYB104	44.54	119	66	2	40	396	18	134	1.00E-29	111
TcMYB16	ATMYB119	47.79	113	53	3	34	354	103	213	2.00E-28	108
TcMYB16	ATMYB119	23.13	134	92	4	190	558	102	230	2.00E-04	35
TcMYB16	AtMYB98	45.05	111	60	2	25	354	212	320	3.00E-27	104
TcMYB16	ATMYB25	48.54	103	52	2	40	345	50	150	1.00E-26	102
TcMYB16	ATMYB44	40.46	131	73	4	40	417	6	134	6.00E-26	99.4
TcMYB16	AtMYB115	41.51	106	62	2	40	357	158	261	9.00E-26	99.8
TcMYB16	ATMYB118	43.24	111	62	2	25	354	184	292	2.00E-25	99.8
TcMYB16	ATMYB118	25.33	75	51	2	151	360	169	243	3.00E-05	38.1
TcMYB16	AtMYB109	41.48	135	70	4	40	417	56	188	3.00E-25	99
TcMYB16	ATMYB73	44.66	103	56	2	40	345	13	113	9.00E-25	96.3
TcMYB16	AtMYB1	45.63	103	55	2	40	345	55	155	2.00E-24	96.3
TcMYB16	AtMYB1	31.88	69	46	1	184	387	50	118	7.00E-04	33.5
TcMYB16	ATMYB77	43.69	103	57	2	40	345	6	106	2.00E-24	94.7
TcMYB16	AtMYB70	44.66	103	56	2	40	345	13	113	9.00E-24	93.2
TcMYB16	AtMYB100	41.18	119	67	3	25	372	21	137	2.00E-23	90.9
TcMYB16	ATMYB52	42.72	103	58	2	40	345	5	105	1.00E-22	89
TcMYB16	ATMYB105	34.53	139	90	2	25	438	102	238	1.00E-22	90.1
TcMYB16	ATMYB54	41.51	106	61	2	40	354	6	109	2.00E-22	88.2
TcMYB16	AtMYB117	31.55	168	104	4	34	504	96	258	9.00E-22	88.2
TcMYB16	ATMYB110	36.79	106	66	2	40	354	67	170	7.00E-20	82
TcMYB16	AtMYB22	34.82	112	73	1	25	360	49	158	2.00E-19	80.1
TcMYB16	ATMYB91	38.61	101	60	1	49	345	7	107	4.00E-19	80.5
TcMYB16	ATMYB69	36.11	108	68	2	25	345	14	119	9.00E-19	77.8
TcMYB16	AtMYB56	26.67	180	132	3	40	579	93	250	2.00E-18	78.2
TcMYB16	AtMYB89	33.65	104	69	1	43	354	57	159	1.00E-16	70.5
TcMYB16	AtMYB124	27.54	334	213	13	49	963	28	340	2.00E-16	73.2
TcMYB16	ATMYB88	38.61	101	62	1	49	351	33	132	2.00E-15	70.1
TcMYB17	MYB8	58.39	137	56	1	16	423	4	140	5.00E-57	176
TcMYB17	ATMYB3	41.53	248	141	4	16	747	4	231	2.00E-56	176
TcMYB17	ATMYB4	59.71	139	53	1	16	423	4	142	3.00E-56	176
TcMYB17	AtMYB6	50.3	165	82	1	16	510	4	163	4.00E-56	174
TcMYB17	ATMYB121	61.24	129	48	1	43	423	28	156	5.00E-56	175
TcMYB17	ATMYB13	60.43	139	52	1	22	429	6	144	9.00E-55	171
TcMYB17	AtMYB32	64.96	117	41	0	16	366	4	120	1.00E-54	172
TcMYB17	AtMYB79	42.51	207	117	2	43	657	7	209	1.00E-54	171
TcMYB17	MYB7	64.1	117	42	0	16	366	4	120	2.00E-54	171
TcMYB17	ATMYB5	40.35	228	131	4	1	669	10	232	4.00E-54	169
TcMYB17	AtMYB41	64.96	117	41	0	16	366	4	120	1.00E-53	169
TcMYB17	AtMYB107	65.81	117	40	0	16	366	4	120	1.00E-53	170
TcMYB17	ATMYB71	46.55	174	92	2	43	561	19	190	2.00E-53	168
TcMYB17	ATMYB102	66.67	117	39	0	16	366	4	120	3.00E-53	170
TcMYB17	AtMYB108	42.6	223	127	5	40	705	19	235	3.00E-53	169
TcMYB17	ATMYB3	58.33	120	50	0	4	363	8	127	3.00E-53	166
TcMYB17	AtMYB24	59.32	118	48	0	37	390	16	133	8.00E-53	165
TcMYB17	AtMYB74	66.09	115	39	0	22	366	7	121	2.00E-52	167
TcMYB17	AtMYB9	64.96	117	41	0	16	366	4	120	2.00E-52	167
TcMYB17	ATMYB66	53.79	145	61	3	1	417	1	141	7.00E-52	162
TcMYB17	AtMYB112	46.24	173	92	3	37	552	31	195	8.00E-52	163
TcMYB17	ATMYB48	46.2	171	92	2	43	555	8	153	8.00E-52	164
TcMYB17	ATMYB58	57.5	120	51	0	22	381	8	127	2.00E-51	163
TcMYB17	ATMYB63	56.49	131	56	1	22	411	8	138	2.00E-51	164
TcMYB17	ATMYB59-3	62.86	105	39	0	43	357	9	113	2.00E-51	162
TcMYB17	ATMYB106	64.96	117	41	0	16	366	47	163	3.00E-51	166
TcMYB17	ATMYB14	59.06	127	49	1	22	393	6	132	4.00E-51	162
TcMYB17	ATMYB12	57.86	140	53	2	22	423	6	145	4.00E-51	165
TcMYB17	AtMYB49	62.39	117	44	0	16	366	4	120	9.00E-51	163
TcMYB17	AtMYB51	44.44	207	114	5	22	639	7	197	2.00E-50	163
TcMYB17	ATMYB57	57.63	118	50	0	16	369	17	134	2.00E-50	158
TcMYB17	ATMYB23	53.57	140	65	2	10	429	2	138	3.00E-50	158
TcMYB17	ATMYB111	64.86	111	39	0	22	354	6	116	6.00E-50	161
TcMYB17	ATMYB2	51.43	140	67	1	1	417	7	146	8.00E-50	159
TcMYB17	ATMYB16	63.25	117	43	0	16	366	4	120	8.00E-50	160
TcMYB17	AtMYB10	52.52	139	64	1	1	411	1	139	9.00E-50	158
TcMYB17	ATMYB30	43.08	195	90	1	22	543	6	200	1.00E-49	160
TcMYB17	AtMYB62	58.33	120	49	1	25	381	14	133	1.00E-49	159
TcMYB17	ATMYB11	66.67	111	37	0	22	354	6	116	1.00E-49	160
TcMYB17	ATMYB86	63.48	115	42	0	22	366	6	120	1.00E-49	160
TcMYB17	ATMYB29	42.73	227	108	7	22	636	7	223	3.00E-49	159
TcMYB17	ATMYB67	54.62	130	51	1	1	366	1	130	4.00E-49	158
TcMYB17	AtMYB53	60.68	117	46	0	16	366	4	120	4.00E-49	158
TcMYB17	ATMYB123	60.17	118	47	0	1	354	1	118	4.00E-49	157

TcMYB17	AtMYB17	62.61	115	43	0	22	366	6	120	4.00E-49	158
TcMYB17	AtMYB116	58.47	118	48	1	28	378	14	131	6.00E-49	157
TcMYB17	AtMYB85	59.13	115	47	0	22	366	6	120	7.00E-49	156
TcMYB17	AtMYB93	62.39	117	44	0	16	366	4	120	8.00E-49	159
TcMYB17	ATMYB15	63.72	113	41	0	22	360	6	118	1.00E-48	156
TcMYB17	AtMYB50	60.87	115	45	0	22	366	6	120	1.00E-48	157
TcMYB17	ATMYB96	58.54	123	48	1	22	381	6	128	2.00E-48	158
TcMYB17	ATMYB61	56.59	129	53	1	22	399	6	134	2.00E-48	158
TcMYB17	AtMYB20	59.13	115	47	0	22	366	6	120	3.00E-48	155
TcMYB17	ATMYB31	58.54	123	51	0	22	390	6	128	4.00E-48	156
TcMYB17	ATMYB92	58.97	117	48	0	16	366	4	120	6.00E-48	156
TcMYB17	ATMYB94	59.48	116	47	0	22	369	6	121	8.00E-48	155
TcMYB17	AtMYB60	56.15	130	57	0	22	411	6	135	8.00E-48	154
TcMYB17	ATMYB78	48.34	151	63	2	10	417	16	166	8.00E-48	155
TcMYB17	ATMYB72	56.3	119	52	0	22	378	8	126	9.00E-48	154
TcMYB17	AtMYB42	61.26	111	43	0	22	354	6	116	9.00E-48	154
TcMYB17	AtMYB83	43.24	185	103	1	31	579	27	211	1.00E-47	155
TcMYB17	AtMYB43	50.7	142	61	1	22	420	6	147	2.00E-47	154
TcMYB17	ATMYB28	60	115	46	1	22	366	7	120	3.00E-47	155
TcMYB17	ATMYB34	49.66	145	73	2	22	456	7	145	3.00E-47	153
TcMYB17	ATMYB0	57.14	119	51	1	28	384	10	126	4.00E-47	150
TcMYB17	AtMYB76	58.33	120	50	1	7	366	2	120	5.00E-47	154
TcMYB17	ATMYB80	58.26	115	48	0	22	366	6	120	2.00E-46	152
TcMYB17	ATMYB46	58.77	114	47	0	37	378	17	130	2.00E-46	150
TcMYB17	AtMYB103	59.82	112	45	0	22	357	6	117	2.00E-46	153
TcMYB17	AtMYB82	52.52	139	64	2	28	438	6	143	2.00E-46	148
TcMYB17	ATMYB95	41.1	219	129	4	40	696	12	194	3.00E-46	150
TcMYB17	ATMYB122	59.13	115	47	0	22	366	6	120	5.00E-46	151
TcMYB17	ATMYB99	55.74	122	47	1	22	366	7	128	5.00E-46	148
TcMYB17	ATMYB35	42.25	187	100	3	22	558	6	192	6.00E-46	150
TcMYB17	AtMYB27	60.36	111	42	1	40	366	9	119	6.00E-46	148
TcMYB17	ATMYB101	42.44	172	98	3	40	552	18	187	1.00E-45	153
TcMYB17	ATMYB55	41.71	199	104	3	22	582	6	192	1.00E-44	147
TcMYB17	AtMYB40	57.39	115	49	0	22	366	6	120	2.00E-44	145
TcMYB17	ATMYB26	46.98	149	67	2	22	432	6	154	3.00E-44	147
TcMYB17	ATMYB37	54.17	120	54	1	22	378	6	125	3.00E-44	146
TcMYB17	AtMYB47	35.63	247	157	4	40	774	12	230	4.00E-44	144
TcMYB17	AtMYB36	57.14	112	47	1	22	354	6	117	5.00E-44	145
TcMYB17	AtMYB97	47.68	151	69	3	40	462	19	168	9.00E-44	146
TcMYB17	ATMYB68	49.26	136	68	1	22	426	6	141	9.00E-44	146
TcMYB17	ATMYB87	34.31	239	117	4	22	618	6	244	2.00E-43	143
TcMYB17	AtMYB19	53.49	129	59	1	31	414	9	137	2.00E-43	142
TcMYB17	ATMYB84	57.02	114	48	1	22	360	6	119	2.00E-43	143
TcMYB17	AtMYB18	55.81	129	57	1	43	429	11	133	2.00E-43	142
TcMYB17	ATMYB120	57.55	106	45	0	40	357	26	131	4.00E-42	144
TcMYB17	ATMYB65	52.21	113	51	1	22	351	32	144	5.00E-42	144
TcMYB17	ATMYB38	55.36	112	49	1	22	354	6	117	2.00E-41	138
TcMYB17	AtMYB114	55.77	104	46	0	40	351	8	111	5.00E-41	132
TcMYB17	AtMYB45	57.55	106	45	0	31	348	15	120	6.00E-41	135
TcMYB17	AtMYB113	41.67	156	91	2	40	507	8	157	9.00E-41	135
TcMYB17	AtMYB81	54.46	112	51	1	31	366	17	127	1.00E-40	139
TcMYB17	ATMYB90	49.61	127	64	1	40	420	8	130	3.00E-40	134
TcMYB17	ATMYB75	55.77	104	46	0	40	351	8	111	4.00E-40	133
TcMYB17	ATMYB33	51.3	115	56	1	7	351	25	135	4.00E-40	139
TcMYB17	ATMYB119	44.62	130	64	3	40	405	103	230	6.00E-31	112
TcMYB17	AtMYB64	32	225	135	5	46	666	105	324	2.00E-30	111
TcMYB17	AtMYB70	47.66	107	56	1	46	366	13	118	1.00E-29	107
TcMYB17	AtMYB104	43.86	114	64	1	25	366	11	123	9.00E-29	106
TcMYB17	ATMYB73	45.79	107	58	1	46	366	13	118	1.00E-28	104
TcMYB17	ATMYB44	42.61	115	66	1	46	390	6	119	5.00E-28	102
TcMYB17	AtMYB98	42.24	116	67	2	4	351	204	317	7.00E-28	104
TcMYB17	AtMYB98	35.48	62	39	1	175	357	207	268	2.00E-08	47.4
TcMYB17	AtMYB109	47.27	110	55	2	46	366	56	164	7.00E-28	103
TcMYB17	AtMYB115	40.68	118	70	2	28	381	152	267	3.00E-27	101
TcMYB17	ATMYB77	35.33	167	107	4	46	543	6	168	5.00E-27	100
TcMYB17	ATMYB118	44.95	109	59	2	28	351	183	289	5.00E-27	102
TcMYB17	ATMYB118	26.39	72	51	2	190	399	184	255	4.00E-06	40
TcMYB17	ATMYB25	46.73	107	56	2	34	351	45	150	1.00E-26	100
TcMYB17	AtMYB1	45.1	102	56	1	46	351	55	155	4.00E-26	99
TcMYB17	AtMYB1	32.86	70	45	2	205	408	55	124	1.00E-04	35
TcMYB17	ATMYB105	37.59	133	82	3	25	420	100	226	7.00E-25	94.7
TcMYB17	AtMYB117	41.74	115	65	3	13	351	86	198	1.00E-24	94.7
TcMYB17	AtMYB100	39.13	115	70	2	16	360	16	128	1.00E-24	92
TcMYB17	ATMYB54	35.1	151	97	3	46	495	6	148	3.00E-24	91.3
TcMYB17	ATMYB91	37.39	115	70	1	55	393	7	121	5.00E-24	92.8
TcMYB17	AtMYB56	31.25	160	110	2	46	525	93	244	4.00E-23	89.7
TcMYB17	ATMYB52	46.88	96	50	2	46	330	5	98	5.00E-23	88.2
TcMYB17	ATMYB110	37.5	104	65	1	46	357	67	169	2.00E-22	87.8
TcMYB17	ATMYB69	33.58	134	89	1	28	429	13	145	3.00E-21	83.2
TcMYB17	AtMYB22	36.52	115	73	3	16	360	44	155	1.00E-20	82

TcMYB17	AtMYB89	35.29	119	75	2	28	378	50	167	1.00E-20	80.5
TcMYB17	AtMYB124	25.82	213	155	4	55	684	28	224	3.00E-19	79.7
TcMYB17	ATMYB88	34.31	102	67	1	55	360	33	133	3.00E-16	70.9
TcMYB18	ATMYB33	36.6	530	311	17	19	1533	29	514	9.00E-80	251
TcMYB18	ATMYB65	35.41	545	311	17	10	1521	35	547	5.00E-79	250
TcMYB18	ATMYB101	34.26	540	320	19	19	1533	15	490	1.00E-69	224
TcMYB18	AtMYB81	39.19	370	205	13	25	1074	19	364	1.00E-65	212
TcMYB18	AtMYB97	69.34	137	41	1	7	414	12	148	6.00E-64	206
TcMYB18	ATMYB120	37.77	376	224	8	16	1113	22	377	4.00E-62	205
TcMYB18	ATMYB92	65.38	104	36	0	28	339	12	115	3.00E-46	157
TcMYB18	ATMYB102	64.42	104	37	0	28	339	12	115	3.00E-46	158
TcMYB18	AtMYB74	63.46	104	38	0	28	339	13	116	4.00E-46	157
TcMYB18	AtMYB9	62.5	104	39	0	28	339	12	115	1.00E-45	155
TcMYB18	AtMYB49	36.7	267	156	8	28	789	12	250	2.00E-45	155
TcMYB18	ATMYB71	56.3	119	47	1	7	348	6	124	2.00E-45	153
TcMYB18	ATMYB3	45.24	168	92	2	28	531	12	153	2.00E-45	153
TcMYB18	AtMYB51	41.32	242	126	8	28	705	13	252	4.00E-45	155
TcMYB18	AtMYB17	63.46	104	38	0	28	339	12	115	4.00E-45	153
TcMYB18	AtMYB41	60.58	104	41	0	28	339	12	115	5.00E-45	152
TcMYB18	AtMYB53	62.5	104	39	0	28	339	12	115	8.00E-45	153
TcMYB18	AtMYB107	60.58	104	41	0	28	339	12	115	1.00E-44	153
TcMYB18	ATMYB34	40.79	228	125	9	28	681	12	214	1.00E-44	152
TcMYB18	ATMYB14	58.65	104	43	0	28	339	12	115	2.00E-44	150
TcMYB18	AtMYB79	60	105	42	0	31	345	7	111	2.00E-44	150
TcMYB18	ATMYB13	60.58	104	41	0	28	339	12	115	3.00E-44	149
TcMYB18	AtMYB24	55.36	112	46	1	13	336	8	119	4.00E-44	148
TcMYB18	AtMYB93	62.5	104	39	0	28	339	12	115	5.00E-44	152
TcMYB18	ATMYB15	47.77	157	82	2	28	498	12	158	1.00E-43	149
TcMYB18	ATMYB3	54.87	113	46	1	13	336	10	122	1.00E-43	147
TcMYB18	AtMYB6	61.76	102	39	0	34	339	14	115	2.00E-43	147
TcMYB18	MYB8	59.62	104	42	0	28	339	12	115	3.00E-43	145
TcMYB18	ATMYB57	54.63	108	49	0	22	345	23	130	3.00E-43	145
TcMYB18	ATMYB66	54.05	111	51	0	7	339	9	119	4.00E-43	145
TcMYB18	ATMYB122	38.25	251	142	8	28	741	12	249	9.00E-43	148
TcMYB18	ATMYB95	45.18	166	85	3	28	507	12	172	1.00E-42	145
TcMYB18	ATMYB121	44.65	159	88	1	31	507	28	183	2.00E-42	145
TcMYB18	MYB7	59.62	104	42	0	28	339	12	115	2.00E-42	145
TcMYB18	ATMYB28	36.78	242	143	5	19	714	9	224	2.00E-42	148
TcMYB18	ATMYB16	59.62	104	42	0	28	339	12	115	2.00E-42	147
TcMYB18	ATMYB106	59.62	104	42	0	28	339	55	158	2.00E-42	148
TcMYB18	AtMYB116	53.1	113	53	0	7	345	11	123	3.00E-42	145
TcMYB18	ATMYB94	58.65	104	43	0	28	339	12	115	5.00E-42	146
TcMYB18	AtMYB60	58.65	104	43	0	28	339	12	115	7.00E-42	144
TcMYB18	AtMYB43	37.5	232	126	7	28	666	12	236	8.00E-42	145
TcMYB18	ATMYB5	56.73	104	45	0	28	339	23	126	8.00E-42	143
TcMYB18	ATMYB86	57.55	106	45	0	28	345	12	117	1.00E-41	145
TcMYB18	AtMYB85	47.33	150	78	4	28	474	12	145	1.00E-41	143
TcMYB18	ATMYB23	41.21	165	97	2	1	495	3	163	1.00E-41	141
TcMYB18	ATMYB29	58.49	106	44	0	19	336	9	114	1.00E-41	145
TcMYB18	ATMYB4	60.78	102	40	0	34	339	14	115	1.00E-41	143
TcMYB18	ATMYB96	57.69	104	44	0	28	339	12	115	1.00E-41	145
TcMYB18	AtMYB50	58.65	104	43	0	28	339	12	115	2.00E-41	144
TcMYB18	AtMYB32	59.8	102	41	0	34	339	14	115	2.00E-41	143
TcMYB18	ATMYB63	58.65	104	43	0	28	339	14	117	2.00E-41	143
TcMYB18	AtMYB36	59.43	106	42	1	25	339	11	116	2.00E-41	144
TcMYB18	ATMYB30	57.55	106	45	0	22	339	10	115	3.00E-41	144
TcMYB18	AtMYB76	57.01	107	46	0	19	339	9	115	3.00E-41	144
TcMYB18	ATMYB59-3	54.87	113	50	1	31	366	9	121	4.00E-41	140
TcMYB18	AtMYB42	56.73	104	45	0	28	339	12	115	4.00E-41	142
TcMYB18	ATMYB87	58.49	106	43	1	25	339	11	116	5.00E-41	142
TcMYB18	ATMYB72	57.69	104	44	0	28	339	14	117	5.00E-41	142
TcMYB18	ATMYB61	57.69	104	44	0	28	339	12	115	7.00E-41	144
TcMYB18	ATMYB48	54.87	113	50	1	31	366	8	120	7.00E-41	140
TcMYB18	AtMYB20	54.72	106	48	0	28	345	12	117	1.00E-40	141
TcMYB18	ATMYB58	55.77	104	46	0	28	339	14	117	2.00E-40	140
TcMYB18	AtMYB104	31.68	404	260	17	34	1197	18	367	2.00E-40	143
TcMYB18	ATMYB11	56.73	104	45	0	28	339	12	115	2.00E-40	142
TcMYB18	AtMYB82	48.76	121	62	0	16	378	8	128	2.00E-40	137
TcMYB18	ATMYB99	54.05	111	44	1	28	339	13	123	3.00E-40	139
TcMYB18	ATMYB12	55.77	104	46	0	28	339	12	115	3.00E-40	142
TcMYB18	AtMYB62	53.77	106	49	0	28	345	19	124	3.00E-40	140
TcMYB18	ATMYB31	56.73	104	45	0	28	339	12	115	4.00E-40	140
TcMYB18	ATMYB46	55.77	104	46	0	28	339	18	121	5.00E-40	139
TcMYB18	ATMYB0	55.34	103	46	0	31	339	15	117	6.00E-40	137
TcMYB18	AtMYB47	42.44	172	86	4	28	504	12	183	7.00E-40	138
TcMYB18	AtMYB112	52.83	106	50	0	28	345	32	137	7.00E-40	137
TcMYB18	ATMYB123	54.81	104	47	0	28	339	14	117	7.00E-40	138
TcMYB18	ATMYB80	55.24	105	47	0	25	339	11	115	9.00E-40	139
TcMYB18	ATMYB68	59.43	106	42	1	25	339	11	116	1.00E-39	140
TcMYB18	ATMYB35	54.29	105	48	0	25	339	11	115	2.00E-39	138

TcMYB18	ATMYB84	58.49	106	43	1	25	339	11	116	3.00E-39	138
TcMYB18	AtMYB83	33.46	269	130	6	7	666	23	290	3.00E-39	139
TcMYB18	AtMYB108	53.77	106	49	0	28	345	19	124	5.00E-39	137
TcMYB18	AtMYB103	42.07	164	86	3	28	492	12	163	5.00E-39	139
TcMYB18	ATMYB38	43.29	164	89	3	25	504	11	152	5.00E-39	137
TcMYB18	ATMYB2	52.83	106	50	0	28	345	20	125	5.00E-39	136
TcMYB18	AtMYB10	55.77	104	46	0	28	339	14	117	8.00E-39	134
TcMYB18	ATMYB111	55.77	104	46	0	28	339	12	115	2.00E-38	136
TcMYB18	ATMYB37	56.19	105	45	1	28	339	12	116	2.00E-38	136
TcMYB18	ATMYB55	53.39	118	43	1	28	345	12	129	2.00E-38	136
TcMYB18	ATMYB67	50.93	108	53	0	16	339	18	125	3.00E-38	135
TcMYB18	AtMYB40	47.33	131	68	1	28	417	12	142	1.00E-37	132
TcMYB18	ATMYB26	39.66	174	96	4	28	522	12	176	1.00E-37	135
TcMYB18	AtMYB27	53.7	108	50	0	25	348	8	115	3.00E-37	130
TcMYB18	AtMYB114	47.27	110	58	0	10	339	2	111	7.00E-37	126
TcMYB18	ATMYB75	47.27	110	58	0	10	339	2	111	1.00E-35	126
TcMYB18	AtMYB45	54.9	102	46	0	31	336	19	120	2.00E-35	126
TcMYB18	AtMYB19	34.56	217	131	4	31	648	13	210	3.00E-35	125
TcMYB18	ATMYB78	45.83	120	51	1	28	345	26	145	1.00E-34	125
TcMYB18	ATMYB90	45.13	113	62	0	10	348	2	114	2.00E-34	122
TcMYB18	AtMYB113	48.08	104	54	0	28	339	8	111	3.00E-34	122
TcMYB18	AtMYB18	50.49	103	51	0	31	339	11	113	3.00E-32	117
TcMYB18	AtMYB109	53.51	114	50	3	7	339	45	156	9.00E-31	116
TcMYB18	AtMYB98	44.72	123	66	2	25	387	214	335	3.00E-30	115
TcMYB18	ATMYB119	49.12	114	57	3	10	348	97	207	3.00E-30	115
TcMYB18	ATMYB73	51.96	102	49	1	34	339	13	113	6.00E-30	112
TcMYB18	AtMYB70	50.94	106	52	1	34	351	13	117	6.00E-30	112
TcMYB18	AtMYB1	50.88	114	54	2	4	339	43	155	2.00E-29	112
TcMYB18	ATMYB77	49.53	107	54	1	34	354	6	111	3.00E-29	110
TcMYB18	ATMYB25	50.91	110	53	2	13	339	43	150	3.00E-29	111
TcMYB18	AtMYB64	50	106	52	3	34	348	105	207	5.00E-29	111
TcMYB18	ATMYB118	47.22	108	57	2	25	348	186	291	5.00E-28	108
TcMYB18	AtMYB115	47.06	102	53	2	34	336	158	257	1.00E-27	106
TcMYB18	ATMYB44	50	102	51	1	34	339	6	106	2.00E-27	105
TcMYB18	AtMYB117	36.36	154	97	3	28	486	96	243	5.00E-27	104
TcMYB18	AtMYB100	46.23	106	56	2	28	342	24	127	9.00E-27	101
TcMYB18	AtMYB56	42.54	134	73	3	1	390	80	212	1.00E-26	103
TcMYB18	ATMYB105	42.98	114	64	2	7	345	98	209	3.00E-26	102
TcMYB18	ATMYB54	45.71	105	56	2	34	345	6	108	9.00E-26	98.6
TcMYB18	ATMYB110	46.15	104	56	1	34	345	67	169	1.00E-25	99.8
TcMYB18	ATMYB52	44.64	112	61	2	34	366	5	114	1.00E-25	98.2
TcMYB18	ATMYB69	45.87	109	57	2	25	345	16	121	1.00E-25	98.2
TcMYB18	AtMYB22	40.95	105	62	2	28	342	52	154	7.00E-22	87.8
TcMYB18	ATMYB91	37.86	103	62	1	43	345	7	109	7.00E-21	86.7
TcMYB18	AtMYB89	39.81	103	59	2	37	336	57	155	4.00E-19	78.2
TcMYB18	AtMYB124	32.67	101	68	1	43	345	28	127	3.00E-16	73.2
TcMYB18	ATMYB88	31.37	102	69	2	43	345	33	132	1.00E-14	68.2
TcMYB19	AtMYB109	38.84	363	216	11	10	1080	47	335	6.00E-68	216
TcMYB19	ATMYB25	33.41	419	272	13	13	1248	42	362	2.00E-56	184
TcMYB19	AtMYB1	36.67	360	224	10	7	1074	46	325	1.00E-55	183
TcMYB19	ATMYB44	55.47	137	61	1	28	438	3	111	6.00E-46	155
TcMYB19	ATMYB73	57.58	132	56	1	28	423	10	113	6.00E-46	155
TcMYB19	AtMYB70	57.04	135	58	1	28	432	10	116	1.00E-45	154
TcMYB19	ATMYB77	54.07	135	62	1	28	432	3	109	3.00E-44	150
TcMYB19	ATMYB54	44.19	129	72	1	37	423	6	106	6.00E-34	120
TcMYB19	ATMYB52	43.41	129	73	1	37	423	5	105	5.00E-33	118
TcMYB19	AtMYB117	43.08	130	74	1	37	426	98	199	7.00E-32	118
TcMYB19	ATMYB69	42.64	129	74	1	37	423	19	119	1.00E-31	115
TcMYB19	ATMYB105	42.31	130	75	1	37	426	107	208	3.00E-31	115
TcMYB19	ATMYB118	40.62	160	95	2	34	513	188	319	4.00E-31	117
TcMYB19	AtMYB56	42.64	129	74	1	37	423	93	193	2.00E-30	113
TcMYB19	AtMYB98	42.75	131	75	2	34	426	216	318	2.00E-30	115
TcMYB19	ATMYB110	40.31	129	77	1	37	423	67	167	3.00E-28	106
TcMYB19	ATMYB119	31.88	207	123	6	34	600	104	281	4.00E-28	108
TcMYB19	AtMYB51	35.67	171	107	4	37	540	15	156	5.00E-28	107
TcMYB19	ATMYB121	32.75	229	143	7	37	690	29	218	7.00E-28	105
TcMYB19	AtMYB64	41.54	130	76	2	34	423	104	205	7.00E-28	107
TcMYB19	AtMYB27	35.98	189	116	6	37	588	11	170	1.00E-26	100
TcMYB19	ATMYB59-3	32.32	198	128	4	25	600	6	174	2.00E-26	100
TcMYB19	AtMYB115	38.28	128	79	1	37	420	158	257	2.00E-26	102
TcMYB19	ATMYB48	40.46	131	76	3	37	423	9	110	5.00E-26	99.4
TcMYB19	ATMYB48	31.48	54	37	1	37	198	62	114	3.00E-04	34.7
TcMYB19	ATMYB66	32.76	232	137	8	7	645	8	199	5.00E-26	97.8
TcMYB19	AtMYB17	39.26	135	80	3	37	435	14	119	8.00E-26	99.8
TcMYB19	AtMYB19	33.85	192	125	4	4	573	3	162	1.00E-25	98.6
TcMYB19	ATMYB63	33.33	186	115	4	37	567	16	169	3.00E-25	98.2
TcMYB19	AtMYB45	33.33	192	126	4	25	594	16	168	3.00E-25	97.4
TcMYB19	ATMYB15	35.2	196	120	6	37	603	14	180	4.00E-25	97.4
TcMYB19	AtMYB107	40.15	137	79	3	37	438	14	122	5.00E-25	97.8
TcMYB19	ATMYB96	37.04	162	93	4	37	495	14	143	8.00E-25	97.8

TcMYB19	AtMYB36	40.15	137	80	3	37	441	14	122	9.00E-25	97.4
TcMYB19	AtMYB10	33.69	187	120	6	37	585	16	168	1.00E-24	95.1
TcMYB19	ATMYB5	40.76	157	89	5	37	495	25	153	1.00E-24	95.1
TcMYB19	AtMYB47	38.03	142	85	3	37	453	14	127	1.00E-24	95.5
TcMYB19	ATMYB31	34.76	164	102	4	37	513	14	144	2.00E-24	96.7
TcMYB19	ATMYB101	31.96	194	124	5	37	594	20	184	2.00E-24	98.6
TcMYB19	AtMYB9	40.88	137	78	3	37	438	14	122	2.00E-24	96.7
TcMYB19	AtMYB93	40.15	137	79	3	37	438	14	122	2.00E-24	96.7
TcMYB19	ATMYB30	34.71	170	105	4	37	528	14	151	3.00E-24	95.5
TcMYB19	AtMYB74	39.42	137	80	3	37	438	15	123	4.00E-24	95.5
TcMYB19	ATMYB95	37.84	148	89	3	37	471	14	133	4.00E-24	94.4
TcMYB19	ATMYB72	36.36	187	114	7	37	582	16	169	4.00E-24	94.7
TcMYB19	ATMYB120	40.31	129	76	2	37	420	28	128	4.00E-24	97.4
TcMYB19	AtMYB89	35.43	127	82	1	40	420	57	155	4.00E-24	92
TcMYB19	AtMYB53	39.85	133	78	3	37	429	14	117	5.00E-24	94.7
TcMYB19	AtMYB97	34.38	160	103	3	4	477	10	141	6.00E-24	95.9
TcMYB19	AtMYB60	40.3	134	75	3	37	423	14	115	6.00E-24	94
TcMYB19	AtMYB18	40.58	138	80	4	22	429	7	115	6.00E-24	94
TcMYB19	ATMYB67	34.07	182	115	6	22	552	18	165	7.00E-24	94.4
TcMYB19	ATMYB3	40.15	137	79	3	37	438	14	122	7.00E-24	93.2
TcMYB19	ATMYB122	35.12	168	106	4	37	531	14	153	7.00E-24	94.7
TcMYB19	AtMYB81	37.32	142	87	3	37	456	22	135	8.00E-24	95.9
TcMYB19	ATMYB14	29.52	210	143	6	37	651	14	183	9.00E-24	92.8
TcMYB19	AtMYB50	40.4	151	85	5	37	474	14	135	1.00E-23	93.6
TcMYB19	ATMYB34	39.19	148	86	4	37	468	14	133	1.00E-23	93.2
TcMYB19	ATMYB94	38.81	134	77	3	37	423	14	115	2.00E-23	93.6
TcMYB19	AtMYB41	37.96	137	82	3	37	438	14	122	2.00E-23	92.4
TcMYB19	ATMYB92	37.59	133	81	3	37	429	14	117	3.00E-23	93.2
TcMYB19	ATMYB106	37.18	156	91	4	37	483	57	184	3.00E-23	94
TcMYB19	ATMYB35	30.05	213	135	5	37	633	14	195	3.00E-23	92.8
TcMYB19	ATMYB65	39.23	130	78	2	37	423	43	144	3.00E-23	95.1
TcMYB19	ATMYB13	32.12	193	124	6	37	594	14	165	3.00E-23	91.3
TcMYB19	ATMYB80	36.57	134	84	2	37	435	14	119	3.00E-23	92.8
TcMYB19	ATMYB102	39.42	137	80	3	37	438	14	122	3.00E-23	93.2
TcMYB19	AtMYB49	36.49	148	91	3	4	438	3	122	4.00E-23	92.4
TcMYB19	ATMYB87	39.85	133	78	3	37	429	14	118	4.00E-23	92
TcMYB19	ATMYB84	32.95	176	108	5	37	534	14	159	4.00E-23	92
TcMYB19	MYB8	38.69	137	81	3	37	438	14	122	7.00E-23	89.4
TcMYB19	MYB8	31.17	77	53	3	37	267	67	137	6.00E-04	33.5
TcMYB19	AtMYB116	34.69	147	93	3	7	438	10	128	7.00E-23	90.9
TcMYB19	ATMYB55	33.69	187	104	5	37	537	14	171	7.00E-23	92
TcMYB19	ATMYB33	39.23	130	78	2	37	423	34	135	1.00E-22	93.2
TcMYB19	AtMYB42	33.14	172	113	5	37	546	14	151	1.00E-22	90.5
TcMYB19	ATMYB37	39.85	133	78	3	37	429	14	118	1.00E-22	91.3
TcMYB19	AtMYB83	34.92	189	122	4	10	573	23	175	1.00E-22	91.3
TcMYB19	AtMYB24	36.69	139	86	3	10	420	9	119	1.00E-22	88.6
TcMYB19	ATMYB4	36.53	167	100	5	37	519	14	151	1.00E-22	90.1
TcMYB19	ATMYB23	37.41	139	85	3	13	423	6	115	1.00E-22	88.6
TcMYB19	AtMYB6	32.81	192	116	5	37	573	14	177	1.00E-22	89
TcMYB19	ATMYB46	31.38	188	128	4	37	597	20	175	2.00E-22	89.4
TcMYB19	ATMYB16	36.54	156	90	4	37	477	14	141	3.00E-22	90.1
TcMYB19	AtMYB104	35.66	143	89	4	37	456	18	131	4.00E-22	90.5
TcMYB19	ATMYB38	39.1	133	79	3	37	429	14	118	4.00E-22	89
TcMYB19	ATMYB68	33.92	171	111	5	37	543	14	151	4.00E-22	90.1
TcMYB19	AtMYB82	37.4	131	80	3	37	423	14	115	6.00E-22	86.3
TcMYB19	ATMYB71	38.17	131	79	3	37	423	20	121	9.00E-22	87.4
TcMYB19	MYB7	38.69	137	81	3	37	438	14	122	9.00E-22	87.4
TcMYB19	MYB7	30.23	86	60	2	37	294	67	147	2.00E-04	35
TcMYB19	ATMYB2	38.46	130	79	2	37	423	22	123	1.00E-21	87.4
TcMYB19	AtMYB32	36.36	165	94	4	25	486	10	146	1.00E-21	87.4
TcMYB19	ATMYB26	28.91	256	167	7	37	759	14	237	1.00E-21	89
TcMYB19	ATMYB123	38.52	135	81	4	25	423	12	117	1.00E-21	87
TcMYB19	AtMYB20	31.84	179	118	5	37	561	14	163	1.00E-21	87.4
TcMYB19	AtMYB103	38.17	131	79	3	37	423	14	115	1.00E-21	88.6
TcMYB19	ATMYB61	39.69	131	77	3	37	423	14	115	2.00E-21	88.2
TcMYB19	ATMYB61	31.48	54	37	1	37	198	67	119	7.00E-04	33.9
TcMYB19	ATMYB0	36.57	134	83	3	37	432	16	120	2.00E-21	85.5
TcMYB19	ATMYB86	38.93	131	78	3	37	423	14	115	2.00E-21	87.8
TcMYB19	ATMYB28	36.81	163	95	5	22	486	9	139	2.00E-21	87.8
TcMYB19	ATMYB3	30.73	179	122	3	7	537	12	162	3.00E-21	85.1
TcMYB19	AtMYB40	37.68	138	82	5	37	438	14	122	3.00E-21	85.9
TcMYB19	AtMYB100	28.81	177	114	4	46	540	29	177	3.00E-21	85.1
TcMYB19	AtMYB79	29.56	203	134	5	37	618	8	181	4.00E-21	85.5
TcMYB19	AtMYB76	32.18	202	127	6	22	597	9	178	4.00E-21	86.7
TcMYB19	ATMYB58	34.38	160	104	2	37	513	16	147	5.00E-21	85.5
TcMYB19	ATMYB57	37.21	129	80	2	37	420	27	127	6.00E-21	83.6
TcMYB19	AtMYB85	38.41	138	81	5	37	438	14	122	7.00E-21	84.7
TcMYB19	ATMYB29	35.33	167	91	4	22	471	9	143	4.00E-20	84
TcMYB19	AtMYB114	34.35	131	85	3	37	426	10	112	4.00E-20	79.3
TcMYB19	AtMYB43	31.64	177	112	6	37	540	14	161	1.00E-19	82

TcMYB19	ATMYB111	37.69	130	80	2	37	423	14	115	2.00E-19	81.6
TcMYB19	ATMYB12	36.92	130	81	2	37	423	14	115	3.00E-19	81.6
TcMYB19	ATMYB99	37.58	149	78	6	37	438	15	130	4.00E-19	79.3
TcMYB19	AtMYB62	32.87	143	93	4	4	423	9	122	4.00E-19	80.1
TcMYB19	ATMYB11	36.84	133	83	2	37	432	14	118	4.00E-19	80.9
TcMYB19	ATMYB90	34.35	131	85	3	37	426	10	112	5.00E-19	79
TcMYB19	ATMYB75	33.59	131	86	3	37	426	10	112	7.00E-19	78.6
TcMYB19	AtMYB113	34.35	131	85	3	37	426	10	112	9.00E-19	78.2
TcMYB19	AtMYB112	34.11	129	84	3	37	420	34	134	4.00E-18	76.3
TcMYB19	AtMYB108	33.57	143	92	4	1	420	8	121	6.00E-18	77
TcMYB19	AtMYB22	28.02	182	131	6	37	582	54	199	1.00E-17	75.1
TcMYB19	ATMYB88	30.5	141	98	2	1	423	18	130	4.00E-17	75.9
TcMYB19	AtMYB124	30.56	144	96	3	4	423	10	125	8.00E-17	74.7
TcMYB19	AtMYB124	33.33	57	38	1	37	207	77	132	2.00E-04	35.8
TcMYB19	ATMYB78	34.11	129	84	3	37	420	28	142	9.00E-16	70.5
TcMYB19	ATMYB91	27.32	183	124	4	46	567	7	161	4.00E-15	68.9
TcMYB20	ATMYB105	78.63	131	28	1	295	687	90	219	1.00E-70	218
TcMYB20	AtMYB117	70.55	146	41	1	256	687	65	210	6.00E-70	217
TcMYB20	AtMYB56	49.53	212	87	2	190	765	20	231	6.00E-67	208
TcMYB20	ATMYB52	76.32	114	27	0	340	681	2	115	7.00E-64	198
TcMYB20	ATMYB54	65.97	144	49	1	340	771	3	144	8.00E-64	197
TcMYB20	ATMYB110	39.86	296	178	7	280	1167	45	304	1.00E-62	197
TcMYB20	ATMYB69	73.58	106	28	0	343	660	17	122	5.00E-56	177
TcMYB20	AtMYB89	52.14	140	67	1	247	666	33	161	5.00E-47	152
TcMYB20	ATMYB25	55.24	105	47	0	349	663	50	154	8.00E-40	138
TcMYB20	AtMYB109	52.17	115	55	1	325	669	50	162	1.00E-39	139
TcMYB20	ATMYB44	55.45	101	45	0	349	651	6	106	1.00E-38	134
TcMYB20	AtMYB70	55.45	101	45	0	349	651	13	113	1.00E-37	131
TcMYB20	AtMYB1	43.42	152	86	2	199	654	9	156	9.00E-37	130
TcMYB20	ATMYB73	52.48	101	48	0	349	651	13	113	2.00E-36	128
TcMYB20	ATMYB77	52.48	101	48	0	349	651	6	106	8.00E-36	126
TcMYB20	ATMYB119	42.07	145	79	2	250	669	73	216	3.00E-32	119
TcMYB20	AtMYB64	44.44	135	75	1	349	753	105	227	2.00E-30	114
TcMYB20	ATMYB23	48.04	102	52	1	349	651	14	115	2.00E-29	107
TcMYB20	ATMYB66	42.42	132	66	3	349	714	18	149	2.00E-29	106
TcMYB20	ATMYB15	32.74	226	135	5	349	975	14	238	3.00E-29	108
TcMYB20	ATMYB118	47.17	106	56	0	349	666	189	294	7.00E-29	109
TcMYB20	ATMYB67	45.31	128	63	3	301	663	3	129	1.00E-28	106
TcMYB20	ATMYB0	47.06	102	53	1	349	651	16	117	1.00E-28	104
TcMYB20	AtMYB115	43.12	109	62	0	322	648	149	257	2.00E-28	107
TcMYB20	AtMYB10	46.3	108	56	2	349	666	16	122	2.00E-28	104
TcMYB20	AtMYB100	45.45	99	53	2	355	648	28	125	3.00E-28	103
TcMYB20	AtMYB103	47.01	117	60	2	349	693	14	129	4.00E-28	106
TcMYB20	ATMYB63	46.3	108	56	2	349	666	16	122	4.00E-28	105
TcMYB20	ATMYB121	41.26	143	77	3	331	738	23	164	5.00E-28	104
TcMYB20	AtMYB45	33.71	175	105	4	349	840	20	193	7.00E-28	103
TcMYB20	AtMYB19	47.12	104	53	2	349	654	14	116	9.00E-28	103
TcMYB20	AtMYB27	44.25	113	61	3	349	681	11	120	9.00E-28	102
TcMYB20	AtMYB18	31.78	214	137	5	349	963	12	211	1.00E-27	103
TcMYB20	AtMYB98	42.37	118	68	0	349	702	217	334	2.00E-27	105
TcMYB20	ATMYB58	36.03	136	86	1	349	753	16	151	5.00E-27	101
TcMYB20	AtMYB51	47.06	102	53	1	349	651	15	116	5.00E-27	103
TcMYB20	AtMYB104	39.68	126	75	1	349	723	18	143	5.00E-27	103
TcMYB20	AtMYB82	46.08	102	54	1	349	651	14	115	1.00E-26	98.6
TcMYB20	ATMYB13	38.82	152	83	5	349	774	14	164	2.00E-26	99.4
TcMYB20	ATMYB80	46.6	103	53	2	349	651	14	115	3.00E-26	100
TcMYB20	AtMYB97	38.73	142	86	2	349	771	21	159	4.00E-26	101
TcMYB20	ATMYB35	39.39	132	79	2	349	741	14	143	5.00E-26	99.8
TcMYB20	ATMYB33	34.36	163	106	2	256	741	10	165	5.00E-26	102
TcMYB20	ATMYB72	44.04	109	59	2	349	669	16	123	7.00E-26	99
TcMYB20	AtMYB40	45.71	105	55	2	349	657	14	117	9.00E-26	97.8
TcMYB20	AtMYB81	41.22	131	76	2	286	675	9	131	1.00E-25	100
TcMYB20	ATMYB101	41.73	127	72	3	328	702	13	134	1.00E-25	100
TcMYB20	AtMYB53	47.57	103	52	2	349	651	14	115	1.00E-25	98.6
TcMYB20	ATMYB59-3	37.93	145	88	3	349	777	10	152	1.00E-25	96.7
TcMYB20	ATMYB48	44.86	107	57	2	349	663	9	114	2.00E-25	96.7
TcMYB20	ATMYB46	40	115	67	2	331	669	14	127	2.00E-25	97.1
TcMYB20	AtMYB49	45.1	102	55	1	349	651	14	115	3.00E-25	97.4
TcMYB20	AtMYB41	44.12	102	56	1	349	651	14	115	3.00E-25	96.7
TcMYB20	AtMYB36	40	140	82	5	349	762	14	142	4.00E-25	97.4
TcMYB20	AtMYB50	30.83	240	161	8	349	1053	14	226	4.00E-25	97.1
TcMYB20	ATMYB68	37.76	143	84	4	349	762	14	152	5.00E-25	97.8
TcMYB20	ATMYB95	43.69	103	56	2	349	651	14	115	5.00E-25	95.9
TcMYB20	ATMYB122	46.88	96	50	1	349	633	14	109	6.00E-25	97.1
TcMYB20	ATMYB34	41.38	116	67	2	319	663	9	119	6.00E-25	96.3
TcMYB20	AtMYB79	40.8	125	72	2	349	717	8	131	6.00E-25	95.5
TcMYB20	ATMYB65	35	140	86	2	271	675	13	152	6.00E-25	99
TcMYB20	ATMYB5	42.45	106	60	1	349	663	25	130	9.00E-25	94.7
TcMYB20	AtMYB114	40.78	103	60	1	349	654	10	112	9.00E-25	91.7
TcMYB20	ATMYB86	42.99	107	59	2	349	663	14	119	1.00E-24	96.7

TcMYB20	AtMYB32	38.93	131	78	2	349	735	14	143	1.00E-24	95.1
TcMYB20	ATMYB3	45.63	103	54	2	349	651	14	115	1.00E-24	94.7
TcMYB20	MYB8	42.16	102	58	1	349	651	14	115	1.00E-24	93.6
TcMYB20	ATMYB61	37.93	145	87	4	349	774	14	151	1.00E-24	96.7
TcMYB20	AtMYB107	45.1	102	55	1	349	651	14	115	1.00E-24	95.9
TcMYB20	ATMYB120	36.24	149	94	3	232	675	4	137	1.00E-24	97.8
TcMYB20	ATMYB92	46.6	103	53	2	349	651	14	115	1.00E-24	95.9
TcMYB20	AtMYB9	45.1	102	55	1	349	651	14	115	1.00E-24	95.9
TcMYB20	ATMYB71	39.84	123	72	2	349	711	20	141	2.00E-24	94.4
TcMYB20	ATMYB84	43.69	103	56	2	349	651	14	116	2.00E-24	95.1
TcMYB20	AtMYB116	37.88	132	80	3	319	708	13	140	2.00E-24	94.4
TcMYB20	ATMYB14	42.61	115	64	2	349	687	14	127	2.00E-24	93.6
TcMYB20	AtMYB74	44.66	103	55	2	349	651	15	116	3.00E-24	94.7
TcMYB20	ATMYB123	36.55	145	91	4	349	780	16	141	4.00E-24	93.2
TcMYB20	ATMYB4	44.66	103	55	2	349	651	14	115	4.00E-24	93.6
TcMYB20	ATMYB55	33.14	175	84	5	349	774	14	185	4.00E-24	94.7
TcMYB20	AtMYB22	32.39	142	90	3	274	681	23	163	7.00E-24	92.4
TcMYB20	ATMYB2	47.37	95	49	1	349	630	22	116	1.00E-23	92.4
TcMYB20	ATMYB28	43.4	106	59	1	349	663	14	119	1.00E-23	94
TcMYB20	AtMYB83	41.67	108	62	1	349	669	32	139	1.00E-23	93.6
TcMYB20	AtMYB17	44.66	103	55	2	349	651	14	115	1.00E-23	92.8
TcMYB20	ATMYB75	40.78	103	60	1	349	654	10	112	1.00E-23	91.7
TcMYB20	AtMYB47	40.78	103	59	2	349	651	14	115	1.00E-23	92
TcMYB20	AtMYB6	42.72	103	57	2	349	651	14	115	1.00E-23	91.3
TcMYB20	ATMYB106	44.66	103	55	2	349	651	57	158	1.00E-23	94
TcMYB20	AtMYB112	41.44	111	64	2	301	630	19	128	1.00E-23	91.3
TcMYB20	ATMYB90	34.9	149	93	2	349	783	10	158	2.00E-23	91.3
TcMYB20	ATMYB94	41.67	108	62	1	349	669	14	121	2.00E-23	92.8
TcMYB20	ATMYB30	36.69	139	87	2	349	762	14	146	2.00E-23	92.4
TcMYB20	ATMYB102	44.66	103	55	2	349	651	14	115	3.00E-23	92.4
TcMYB20	ATMYB96	30.87	230	149	5	349	1008	14	241	3.00E-23	92.4
TcMYB20	AtMYB62	34.87	152	87	4	349	768	21	171	3.00E-23	91.3
TcMYB20	MYB7	41.75	103	58	2	349	651	14	115	4.00E-23	90.5
TcMYB20	AtMYB93	43.14	102	57	1	349	651	14	115	5.00E-23	92
TcMYB20	ATMYB38	43.69	103	56	2	349	651	14	116	5.00E-23	90.9
TcMYB20	ATMYB16	42.16	102	58	1	349	651	14	115	5.00E-23	91.3
TcMYB20	AtMYB76	32.77	177	109	4	349	849	14	187	6.00E-23	91.3
TcMYB20	AtMYB60	37.14	140	78	3	349	738	14	153	7.00E-23	90.1
TcMYB20	AtMYB43	39.2	125	74	2	349	717	14	137	8.00E-23	90.9
TcMYB20	ATMYB31	44.12	102	56	1	349	651	14	115	1.00E-22	90.5
TcMYB20	ATMYB3	36.73	147	92	4	235	672	5	129	1.00E-22	88.2
TcMYB20	AtMYB113	38.68	106	64	1	349	663	10	115	1.00E-22	88.6
TcMYB20	ATMYB57	31.14	167	113	3	349	843	27	183	2.00E-22	87.4
TcMYB20	AtMYB108	43.52	108	59	3	313	630	12	115	2.00E-22	89.7
TcMYB20	ATMYB111	37.14	140	87	2	349	765	14	151	2.00E-22	89.7
TcMYB20	ATMYB29	45.83	96	51	1	349	633	14	109	3.00E-22	89.4
TcMYB20	ATMYB37	40	110	63	2	349	669	14	122	5.00E-22	88.6
TcMYB20	ATMYB87	29.65	226	143	7	349	978	14	238	5.00E-22	88.2
TcMYB20	AtMYB20	41.51	106	60	2	349	660	14	118	9.00E-22	87
TcMYB20	AtMYB42	42.72	103	57	2	349	651	14	115	9.00E-22	87
TcMYB20	ATMYB11	34.56	136	76	2	349	717	14	149	2.00E-21	87
TcMYB20	AtMYB24	42.11	95	54	1	349	630	19	113	2.00E-21	84.3
TcMYB20	AtMYB85	42.72	103	57	2	349	651	14	115	2.00E-21	85.5
TcMYB20	ATMYB26	39.64	111	57	2	349	651	14	124	2.00E-21	87
TcMYB20	ATMYB12	40.2	102	60	1	349	651	14	115	5.00E-21	86.3
TcMYB20	ATMYB88	36.89	103	65	0	358	666	33	135	7.00E-21	86.7
TcMYB20	AtMYB124	36.89	103	65	0	358	666	28	130	1.00E-20	85.9
TcMYB20	ATMYB99	42.73	110	54	4	349	651	15	123	3.00E-20	82
TcMYB20	ATMYB91	35.9	117	72	1	349	690	4	120	7.00E-20	82.8
TcMYB20	ATMYB78	34.51	142	78	4	250	630	2	136	1.00E-19	81.3
TcMYB21	ATMYB33	41.99	412	220	14	43	1221	4	374	1.00E-83	259
TcMYB21	ATMYB65	42.71	377	207	9	118	1221	41	387	3.00E-82	256
TcMYB21	ATMYB120	66.87	163	52	2	67	549	9	167	7.00E-71	226
TcMYB21	ATMYB101	54.63	216	77	3	118	702	18	229	2.00E-70	224
TcMYB21	AtMYB81	55.91	186	78	3	91	636	7	188	5.00E-67	213
TcMYB21	AtMYB97	71.32	136	39	1	91	498	10	142	2.00E-66	210
TcMYB21	AtMYB74	47.75	178	89	4	118	639	13	187	2.00E-49	164
TcMYB21	ATMYB5	41.75	194	106	4	118	678	23	209	4.00E-49	160
TcMYB21	AtMYB17	65.38	104	36	0	118	429	12	115	1.00E-48	161
TcMYB21	ATMYB102	65.38	104	36	0	118	429	12	115	2.00E-48	162
TcMYB21	ATMYB15	40.09	222	132	5	118	780	12	215	2.00E-48	160
TcMYB21	AtMYB107	65.38	104	36	0	118	429	12	115	3.00E-48	160
TcMYB21	ATMYB66	43.68	174	89	1	79	573	3	176	5.00E-48	156
TcMYB21	ATMYB3	63.46	104	38	0	118	429	12	115	6.00E-48	158
TcMYB21	AtMYB41	61.54	104	40	0	118	429	12	115	8.00E-48	158
TcMYB21	ATMYB96	35.86	290	143	6	118	858	12	301	2.00E-47	159
TcMYB21	AtMYB9	62.62	107	40	0	118	438	12	118	2.00E-47	159
TcMYB21	AtMYB6	65.69	102	35	0	124	429	14	115	3.00E-47	155
TcMYB21	MYB8	62.5	104	39	0	118	429	12	115	6.00E-47	154
TcMYB21	ATMYB14	59.63	109	44	0	118	444	12	120	9.00E-47	154

TcMYB21	AtMYB79	62.86	105	39	0	121	435	7	111	1.00E-46	154
TcMYB21	ATMYB57	60.19	108	43	0	112	435	23	130	1.00E-46	152
TcMYB21	MYB7	63.46	104	38	0	118	429	12	115	2.00E-46	154
TcMYB21	AtMYB32	47.34	169	88	3	124	627	14	162	2.00E-46	154
TcMYB21	ATMYB3	39.69	194	112	2	79	645	2	187	3.00E-46	152
TcMYB21	ATMYB106	51.8	139	66	2	118	531	55	190	5.00E-46	156
TcMYB21	ATMYB71	44.32	176	92	2	121	630	19	194	9.00E-46	152
TcMYB21	ATMYB4	64.71	102	36	0	124	429	14	115	9.00E-46	153
TcMYB21	ATMYB92	63.46	104	38	0	118	429	12	115	1.00E-45	154
TcMYB21	ATMYB121	38.49	239	136	5	121	804	28	245	1.00E-45	152
TcMYB21	AtMYB53	61.54	104	40	0	118	429	12	115	2.00E-45	153
TcMYB21	ATMYB13	47.47	158	83	3	118	591	12	154	2.00E-45	151
TcMYB21	ATMYB29	45.18	197	104	7	103	681	8	192	2.00E-45	153
TcMYB21	ATMYB28	52.08	144	68	3	103	531	8	147	2.00E-45	154
TcMYB21	AtMYB51	54.07	135	60	2	118	516	13	147	2.00E-45	154
TcMYB21	AtMYB24	54.31	116	53	0	79	426	4	119	3.00E-45	149
TcMYB21	ATMYB16	60.58	104	41	0	118	429	12	115	4.00E-45	152
TcMYB21	AtMYB49	55.56	126	53	2	118	486	12	137	4.00E-45	152
TcMYB21	ATMYB30	60.38	106	42	0	112	429	10	115	5.00E-45	152
TcMYB21	AtMYB76	47.68	151	64	2	103	510	8	157	5.00E-45	152
TcMYB21	AtMYB50	48.59	142	70	2	118	534	12	153	7.00E-45	151
TcMYB21	ATMYB94	58.88	107	44	0	118	438	12	118	1.00E-44	151
TcMYB21	AtMYB60	61.54	104	40	0	118	429	12	115	1.00E-44	150
TcMYB21	ATMYB58	41.34	179	105	2	118	654	14	184	1.00E-44	149
TcMYB21	ATMYB63	42.62	183	96	3	118	639	14	194	2.00E-44	150
TcMYB21	AtMYB93	61.54	104	40	0	118	429	12	115	2.00E-44	152
TcMYB21	ATMYB86	54.33	127	57	2	118	495	12	135	3.00E-44	150
TcMYB21	ATMYB31	60.58	104	41	0	118	429	12	115	8.00E-44	149
TcMYB21	ATMYB34	59.81	107	43	0	118	438	12	118	9.00E-44	148
TcMYB21	ATMYB23	57.28	103	44	0	121	429	13	115	1.00E-43	145
TcMYB21	AtMYB20	53.17	126	57	2	118	489	12	137	1.00E-43	147
TcMYB21	ATMYB99	52.55	137	54	4	118	495	13	146	1.00E-43	146
TcMYB21	AtMYB116	50.83	120	59	1	79	438	6	124	3.00E-43	146
TcMYB21	AtMYB43	52.99	134	61	2	118	513	12	145	3.00E-43	147
TcMYB21	AtMYB85	50	140	68	3	118	531	12	150	3.00E-43	145
TcMYB21	ATMYB87	58.49	106	43	1	115	429	11	116	4.00E-43	146
TcMYB21	ATMYB46	57.01	107	46	0	118	438	18	124	5.00E-43	145
TcMYB21	AtMYB62	51.64	122	59	1	73	438	6	125	5.00E-43	145
TcMYB21	ATMYB61	58.88	107	44	0	118	438	12	118	7.00E-43	147
TcMYB21	ATMYB80	56.19	105	46	0	115	429	11	115	1.00E-42	145
TcMYB21	ATMYB67	49.28	138	69	2	106	516	18	152	1.00E-42	145
TcMYB21	AtMYB42	58.65	104	43	0	118	429	12	115	1.00E-42	144
TcMYB21	AtMYB82	52.14	117	53	1	106	447	8	124	2.00E-42	142
TcMYB21	ATMYB111	57.69	104	44	0	118	429	12	115	2.00E-42	145
TcMYB21	ATMYB0	57.28	103	44	0	121	429	15	117	2.00E-42	142
TcMYB21	ATMYB72	40	190	102	4	118	651	14	199	5.00E-42	143
TcMYB21	ATMYB95	57.69	104	44	0	118	429	12	115	5.00E-42	142
TcMYB21	AtMYB83	42.24	161	90	2	94	567	22	169	6.00E-42	144
TcMYB21	ATMYB122	47.74	155	76	3	118	567	12	166	6.00E-42	144
TcMYB21	AtMYB112	52.83	106	50	0	118	435	32	137	7.00E-42	141
TcMYB21	AtMYB108	54.21	107	49	0	118	438	19	125	1.00E-41	143
TcMYB21	ATMYB12	55.77	104	46	0	118	429	12	115	1.00E-41	144
TcMYB21	ATMYB59-3	54.87	113	50	1	121	456	9	121	2.00E-41	140
TcMYB21	ATMYB84	44.65	159	73	2	115	546	11	169	2.00E-41	142
TcMYB21	ATMYB2	54.72	106	48	0	118	435	20	125	2.00E-41	141
TcMYB21	AtMYB36	47.22	144	71	3	115	531	11	146	2.00E-41	142
TcMYB21	ATMYB123	54.81	104	47	0	118	429	14	117	3.00E-41	140
TcMYB21	ATMYB35	54.29	105	48	0	115	429	11	115	3.00E-41	142
TcMYB21	ATMYB11	54.81	104	47	0	118	429	12	115	4.00E-41	142
TcMYB21	ATMYB48	53.98	113	51	1	121	456	8	120	5.00E-41	139
TcMYB21	ATMYB55	55.08	118	41	1	118	435	12	129	9.00E-41	141
TcMYB21	AtMYB40	48.15	135	67	2	118	513	12	146	1.00E-40	139
TcMYB21	AtMYB10	56.73	104	45	0	118	429	14	117	1.00E-40	138
TcMYB21	AtMYB103	55.14	107	48	0	118	438	12	118	2.00E-40	141
TcMYB21	ATMYB68	28.38	377	239	10	115	1152	11	371	2.00E-40	141
TcMYB21	AtMYB47	40.7	172	99	3	118	624	12	180	1.00E-39	136
TcMYB21	ATMYB26	52.21	113	45	1	118	429	12	124	2.00E-39	138
TcMYB21	ATMYB38	48.12	133	68	2	115	510	11	142	2.00E-39	136
TcMYB21	AtMYB104	52.99	117	55	0	124	474	18	134	2.00E-39	138
TcMYB21	AtMYB114	48.6	107	55	0	109	429	5	111	3.00E-39	131
TcMYB21	ATMYB37	54.29	105	47	1	118	429	12	116	2.00E-38	135
TcMYB21	AtMYB45	37.69	199	115	4	121	690	19	205	2.00E-38	133
TcMYB21	AtMYB27	53.77	106	49	0	115	432	8	113	2.00E-37	129
TcMYB21	ATMYB75	47.66	107	56	0	109	429	5	111	2.00E-37	129
TcMYB21	ATMYB78	46.28	121	51	1	118	438	26	146	6.00E-37	130
TcMYB21	AtMYB113	48.08	104	54	0	118	429	8	111	2.00E-36	127
TcMYB21	AtMYB19	52.29	109	52	0	121	447	13	121	2.00E-36	127
TcMYB21	ATMYB90	47.66	107	56	0	109	429	5	111	5.00E-36	126
TcMYB21	AtMYB18	50.49	103	51	0	121	429	11	113	2.00E-33	120
TcMYB21	AtMYB98	45.93	135	72	4	31	432	195	317	2.00E-32	120

TcMYB21	AtMYB1	44.97	149	69	3	22	429	9	155	3.00E-32	119
TcMYB21	AtMYB70	53.92	102	47	1	124	429	13	113	8.00E-32	116
TcMYB21	ATMYB119	50.46	109	53	3	109	432	100	205	2.00E-31	117
TcMYB21	ATMYB73	51.96	102	49	1	124	429	13	113	3.00E-31	115
TcMYB21	ATMYB118	44.53	137	76	2	115	525	186	320	4.00E-31	116
TcMYB21	AtMYB109	50.44	113	55	2	94	429	46	156	1.00E-30	114
TcMYB21	AtMYB115	43.48	138	76	4	19	426	129	257	2.00E-30	113
TcMYB21	AtMYB64	50	104	51	3	124	432	105	205	8.00E-30	112
TcMYB21	ATMYB77	47.66	107	56	1	124	444	6	111	6.00E-29	108
TcMYB21	ATMYB44	50	102	51	1	124	429	6	106	6.00E-29	108
TcMYB21	AtMYB100	36.17	188	116	5	118	669	24	205	7.00E-29	106
TcMYB21	ATMYB25	49.54	109	54	2	106	429	44	150	1.00E-28	108
TcMYB21	AtMYB117	41.73	139	75	3	55	453	70	206	3.00E-28	107
TcMYB21	ATMYB105	45.22	115	62	2	97	438	98	210	3.00E-28	106
TcMYB21	AtMYB56	48.57	105	54	1	124	438	93	196	4.00E-28	106
TcMYB21	ATMYB52	46.96	115	60	2	124	465	5	117	5.00E-28	104
TcMYB21	ATMYB69	45.87	109	58	2	115	438	16	122	7.00E-28	103
TcMYB21	ATMYB54	37.42	155	95	4	124	582	6	152	6.00E-27	101
TcMYB21	ATMYB110	42.19	128	74	2	55	438	48	170	2.00E-26	101
TcMYB21	AtMYB22	40.3	134	80	3	37	438	30	156	2.00E-25	97.4
TcMYB21	ATMYB91	40.38	104	60	1	133	438	7	110	8.00E-24	94.7
TcMYB21	AtMYB89	36.09	133	82	3	127	516	57	178	2.00E-19	78.6
TcMYB21	AtMYB124	34.65	101	66	1	133	435	28	127	2.00E-17	76.3
TcMYB21	ATMYB88	31.68	101	69	1	133	435	33	132	2.00E-15	70.1
TcMYB22	AtMYB17	63.26	215	79	2	1	645	1	205	7.00E-97	283
TcMYB22	ATMYB16	69.23	169	51	2	1	504	1	167	2.00E-85	255
TcMYB22	ATMYB106	41.64	365	201	11	10	1068	47	374	5.00E-85	256
TcMYB22	AtMYB9	43.55	310	161	6	1	888	1	305	4.00E-77	234
TcMYB22	AtMYB93	39.47	342	183	8	1	954	1	332	3.00E-75	230
TcMYB22	AtMYB107	74.05	131	34	0	1	393	1	131	3.00E-74	226
TcMYB22	ATMYB102	57.98	188	73	2	1	546	1	188	8.00E-74	226
TcMYB22	ATMYB92	74.81	131	33	0	1	393	1	131	4.00E-73	224
TcMYB22	AtMYB49	41.25	320	165	6	1	891	1	314	7.00E-70	215
TcMYB22	AtMYB53	60.61	165	65	0	1	495	1	165	8.00E-70	214
TcMYB22	AtMYB74	72.31	130	35	1	1	387	1	130	8.00E-70	215
TcMYB22	AtMYB41	68.99	129	40	0	1	387	1	129	1.00E-68	211
TcMYB22	AtMYB85	46.29	229	115	3	1	663	1	224	3.00E-68	209
TcMYB22	ATMYB3	63.04	138	51	0	1	414	1	138	6.00E-67	205
TcMYB22	ATMYB4	61.64	146	56	0	1	438	1	146	7.00E-67	206
TcMYB22	AtMYB6	59.6	151	54	1	1	432	1	151	1.00E-66	204
TcMYB22	AtMYB43	65.07	146	51	0	1	438	1	146	2.00E-66	206
TcMYB22	MYB8	66.67	126	42	0	1	378	1	126	3.00E-66	202
TcMYB22	ATMYB34	41.41	297	158	9	1	843	1	289	8.00E-66	204
TcMYB22	MYB7	63.31	139	51	0	1	417	1	139	1.00E-65	202
TcMYB22	AtMYB42	70.31	128	38	0	1	384	1	128	1.00E-65	203
TcMYB22	ATMYB5	61.54	143	48	1	10	417	15	157	3.00E-65	201
TcMYB22	AtMYB32	65.91	132	45	0	1	396	1	132	3.00E-65	201
TcMYB22	ATMYB13	75.86	116	28	0	1	348	1	116	4.00E-65	200
TcMYB22	ATMYB15	57.8	173	73	2	1	519	1	168	6.00E-65	201
TcMYB22	ATMYB14	74.14	116	30	0	1	348	1	116	2.00E-64	199
TcMYB22	AtMYB20	67.69	130	42	0	1	390	1	130	3.00E-64	199
TcMYB22	ATMYB28	56.71	164	59	1	1	456	1	164	3.00E-63	199
TcMYB22	AtMYB51	67.72	127	40	1	1	378	1	127	7.00E-63	198
TcMYB22	ATMYB61	51.02	196	92	1	1	576	1	196	1.00E-62	198
TcMYB22	ATMYB31	37.87	301	169	4	1	849	1	299	6.00E-62	195
TcMYB22	ATMYB80	64.62	130	46	0	1	390	1	130	1.00E-61	194
TcMYB22	ATMYB30	53.71	175	80	3	1	522	1	172	4.00E-61	192
TcMYB22	ATMYB122	66.67	126	42	0	1	378	1	126	5.00E-61	192
TcMYB22	ATMYB29	63.24	136	50	0	1	408	1	136	5.00E-61	192
TcMYB22	ATMYB96	37.37	297	174	7	1	855	1	291	6.00E-61	193
TcMYB22	ATMYB86	34.67	323	200	6	1	936	1	291	6.00E-61	193
TcMYB22	AtMYB50	64.12	131	47	0	1	393	1	131	8.00E-61	191
TcMYB22	AtMYB40	50.27	187	90	2	1	552	1	180	1.00E-60	189
TcMYB22	AtMYB60	71.55	116	33	0	1	348	1	116	5.00E-60	188
TcMYB22	ATMYB12	48.63	183	94	0	1	549	1	183	7.00E-60	191
TcMYB22	ATMYB94	35.67	342	195	9	1	951	1	323	7.00E-60	189
TcMYB22	AtMYB76	65.62	128	44	0	1	384	1	128	8.00E-60	189
TcMYB22	ATMYB35	51.43	175	85	2	1	525	1	162	9.00E-60	189
TcMYB22	ATMYB11	70.69	116	34	0	1	348	1	116	1.00E-59	189
TcMYB22	ATMYB111	68.97	116	36	0	1	348	1	116	2.00E-59	189
TcMYB22	ATMYB63	55.76	165	72	2	4	495	4	167	3.00E-59	187
TcMYB22	ATMYB99	61.36	132	44	1	4	378	3	134	7.00E-59	184
TcMYB22	ATMYB95	61.19	134	52	0	1	402	1	134	2.00E-58	184
TcMYB22	ATMYB58	69.57	115	35	0	4	348	4	118	3.00E-58	183
TcMYB22	ATMYB55	59.33	150	49	2	1	414	1	148	1.00E-56	182
TcMYB22	ATMYB72	68.7	115	36	0	4	348	4	118	2.00E-56	179
TcMYB22	ATMYB67	64.46	121	43	0	16	378	16	136	5.00E-56	179
TcMYB22	AtMYB10	54.11	146	67	0	7	444	5	150	5.00E-55	174
TcMYB22	AtMYB47	60.16	128	51	0	1	384	1	128	2.00E-54	173
TcMYB22	AtMYB103	55.88	136	60	0	1	408	1	136	6.00E-53	172

TcMYB22	ATMYB37	41.81	232	132	3	1	687	1	219	2.00E-52	170
TcMYB22	ATMYB87	62.71	118	43	1	1	351	1	118	2.00E-52	169
TcMYB22	ATMYB101	65.18	112	39	0	10	345	10	121	8.00E-51	170
TcMYB22	AtMYB36	63.56	118	42	1	1	351	1	118	1.00E-50	166
TcMYB22	ATMYB26	57.94	126	44	1	1	351	1	126	2.00E-50	166
TcMYB22	ATMYB38	54.11	146	62	2	1	423	1	146	3.00E-50	163
TcMYB22	ATMYB84	61.86	118	44	1	1	351	1	118	3.00E-49	161
TcMYB22	ATMYB66	63.46	104	38	0	37	348	17	120	5.00E-49	157
TcMYB22	ATMYB23	43.39	189	104	3	37	594	13	197	6.00E-49	157
TcMYB22	ATMYB68	61.86	118	44	1	1	351	1	118	8.00E-49	162
TcMYB22	ATMYB123	58.59	128	53	1	34	417	14	130	4.00E-48	157
TcMYB22	AtMYB24	57.5	120	51	0	34	393	17	136	8.00E-48	154
TcMYB22	AtMYB83	51.72	145	70	1	34	468	30	169	8.00E-48	158
TcMYB22	ATMYB46	62.96	108	40	0	34	357	18	125	9.00E-48	156
TcMYB22	ATMYB71	56.45	124	54	1	31	402	17	136	1.00E-47	156
TcMYB22	AtMYB79	64.08	103	37	0	37	345	7	109	3.00E-47	154
TcMYB22	ATMYB0	59.05	105	43	0	37	351	15	119	5.00E-46	150
TcMYB22	ATMYB120	63.64	110	40	1	34	363	26	134	7.00E-46	157
TcMYB22	ATMYB121	40.43	188	111	3	22	582	23	193	9.00E-46	151
TcMYB22	AtMYB81	58.41	113	47	1	25	363	17	128	1.00E-45	155
TcMYB22	ATMYB3	61.68	107	41	0	34	354	20	126	1.00E-45	149
TcMYB22	AtMYB82	59.62	104	42	0	34	345	12	115	2.00E-45	148
TcMYB22	AtMYB114	59.05	105	43	0	31	345	7	111	2.00E-45	145
TcMYB22	ATMYB33	51.47	136	66	2	34	441	32	164	6.00E-45	154
TcMYB22	AtMYB97	61.17	103	40	0	34	342	19	121	9.00E-45	151
TcMYB22	ATMYB65	58.65	104	43	0	34	345	41	144	2.00E-44	154
TcMYB22	ATMYB75	59.05	105	43	0	31	345	7	111	4.00E-44	146
TcMYB22	ATMYB57	60.95	105	41	0	34	348	25	129	9.00E-44	144
TcMYB22	AtMYB116	53.51	114	53	0	37	378	19	132	2.00E-43	145
TcMYB22	AtMYB113	55.65	115	51	1	1	345	1	111	2.00E-43	144
TcMYB22	ATMYB90	58.1	105	44	0	31	345	7	111	2.00E-42	141
TcMYB22	AtMYB112	56.25	112	47	1	25	354	29	140	2.00E-41	139
TcMYB22	AtMYB19	58.49	106	44	0	37	354	13	118	3.00E-41	139
TcMYB22	AtMYB62	52.63	114	54	0	34	375	19	132	3.00E-41	139
TcMYB22	ATMYB48	57.14	105	45	0	31	345	6	110	7.00E-41	137
TcMYB22	ATMYB59-3	57.28	103	44	0	37	345	9	111	2.00E-40	136
TcMYB22	AtMYB108	53.91	115	53	0	22	366	15	129	2.00E-40	138
TcMYB22	AtMYB18	57.55	106	45	0	37	354	11	116	3.00E-40	137
TcMYB22	ATMYB2	49.58	119	60	0	22	378	16	134	3.00E-40	136
TcMYB22	AtMYB45	55.88	102	45	0	37	342	19	120	6.00E-40	135
TcMYB22	AtMYB27	58.65	104	43	0	34	345	9	112	7.00E-40	134
TcMYB22	ATMYB78	29.65	317	202	8	22	909	22	295	2.00E-37	130
TcMYB22	AtMYB104	41.73	139	81	1	40	456	18	155	9.00E-36	127
TcMYB22	AtMYB109	40.28	144	66	3	40	411	56	197	6.00E-30	111
TcMYB22	AtMYB64	27.04	307	210	8	40	918	105	391	1.00E-29	110
TcMYB22	ATMYB77	48.04	102	53	1	40	345	6	106	2.00E-29	108
TcMYB22	ATMYB73	49.02	102	52	1	40	345	13	113	2.00E-29	108
TcMYB22	AtMYB98	44.44	108	60	1	22	345	211	317	4.00E-29	109
TcMYB22	AtMYB98	34.48	58	37	1	169	339	207	264	8.00E-07	42.7
TcMYB22	AtMYB70	50	102	51	1	40	345	13	113	7.00E-29	107
TcMYB22	ATMYB118	45.37	108	59	1	22	345	183	289	3.00E-28	107
TcMYB22	ATMYB118	31.67	60	40	1	184	360	184	243	1.00E-06	42.4
TcMYB22	ATMYB119	43.27	104	59	1	34	345	103	205	7.00E-28	106
TcMYB22	ATMYB119	27.03	74	53	1	199	417	105	178	1.00E-04	36.2
TcMYB22	ATMYB25	48.54	103	52	2	40	345	50	150	1.00E-27	104
TcMYB22	ATMYB44	47.06	102	54	1	40	345	6	106	1.00E-27	103
TcMYB22	ATMYB54	40.43	141	75	4	40	435	6	144	3.00E-27	101
TcMYB22	AtMYB1	32.34	201	116	4	40	582	55	242	6.00E-27	103
TcMYB22	AtMYB115	33.91	174	115	5	40	561	158	316	1.00E-26	101
TcMYB22	ATMYB52	45.1	102	56	1	40	345	5	105	1.00E-25	96.7
TcMYB22	ATMYB69	39.81	108	65	1	22	345	13	119	9.00E-24	91.7
TcMYB22	ATMYB105	41.12	107	63	1	25	345	102	207	1.00E-23	92.8
TcMYB22	AtMYB100	42.72	103	59	1	22	330	20	121	1.00E-23	90.9
TcMYB22	AtMYB117	42.31	104	60	1	34	345	96	198	6.00E-23	91.3
TcMYB22	ATMYB110	35.34	133	80	2	40	420	67	198	4.00E-22	88.2
TcMYB22	AtMYB56	39.22	102	62	1	40	345	93	193	1.00E-20	84.3
TcMYB22	ATMYB91	36.63	101	62	1	49	345	7	107	1.00E-19	82
TcMYB22	AtMYB124	39	100	61	1	46	345	27	125	1.00E-19	82.4
TcMYB22	AtMYB22	36.89	103	65	2	22	330	48	148	8.00E-19	77.8
TcMYB22	ATMYB88	37	100	63	1	46	345	32	130	2.00E-18	79
TcMYB22	AtMYB89	36	100	64	1	43	342	57	155	4.00E-18	74.3
TcMYB23	ATMYB67	73.72	137	36	0	10	420	14	150	4.00E-76	229
TcMYB23	AtMYB50	62.28	167	58	2	1	486	1	167	2.00E-75	228
TcMYB23	ATMYB61	62.15	177	51	2	1	483	1	177	9.00E-75	228
TcMYB23	ATMYB86	78.74	127	27	0	1	381	1	127	3.00E-74	226
TcMYB23	ATMYB55	68.21	151	36	1	1	417	1	151	2.00E-73	223
TcMYB23	ATMYB26	73.61	144	29	1	1	405	1	144	3.00E-73	224
TcMYB23	AtMYB103	67.12	146	48	1	1	438	1	144	3.00E-71	219
TcMYB23	AtMYB32	55.84	154	68	1	1	462	1	150	5.00E-63	194
TcMYB23	ATMYB3	61.07	131	51	0	1	393	1	131	9.00E-62	191

TcMYB23	MYB7	58.04	143	60	1	1	429	1	142	2.00E-61	190
TcMYB23	AtMYB6	58.09	136	57	0	1	408	1	136	3.00E-61	189
TcMYB23	AtMYB85	59.86	142	56	1	1	423	1	142	1.00E-60	188
TcMYB23	ATMYB4	56.83	139	60	0	1	417	1	139	9.00E-60	186
TcMYB23	AtMYB42	56.38	149	65	0	1	447	1	149	1.00E-59	186
TcMYB23	ATMYB16	57.75	142	58	1	1	420	1	142	1.00E-59	187
TcMYB23	AtMYB107	60.63	127	50	0	1	381	1	127	3.00E-59	186
TcMYB23	ATMYB106	59.12	137	54	1	16	420	49	185	4.00E-59	188
TcMYB23	ATMYB46	63.64	143	50	2	28	450	16	155	5.00E-59	184
TcMYB23	AtMYB49	50.58	172	81	2	1	504	1	167	1.00E-58	184
TcMYB23	AtMYB83	70.94	117	31	1	7	348	18	134	5.00E-58	184
TcMYB23	MYB8	52.98	151	68	1	1	444	1	151	1.00E-57	179
TcMYB23	AtMYB53	37.38	321	193	9	1	939	1	302	2.00E-57	181
TcMYB23	AtMYB17	60.63	127	50	0	1	381	1	127	2.00E-57	181
TcMYB23	AtMYB93	61.42	127	49	0	1	381	1	127	2.00E-57	183
TcMYB23	ATMYB35	60.31	131	52	0	1	393	1	131	6.00E-57	180
TcMYB23	AtMYB74	61.42	127	48	1	1	378	1	127	9.00E-57	180
TcMYB23	AtMYB9	59.06	127	52	0	1	381	1	127	3.00E-56	179
TcMYB23	ATMYB102	61.11	126	49	0	1	378	1	126	3.00E-56	179
TcMYB23	AtMYB41	43.66	213	107	4	1	600	1	208	3.00E-56	177
TcMYB23	AtMYB20	57.14	140	60	1	1	420	1	136	5.00E-56	177
TcMYB23	ATMYB80	60.63	127	50	0	1	381	1	127	3.00E-55	176
TcMYB23	ATMYB28	36.54	312	177	9	1	873	1	308	3.00E-55	177
TcMYB23	AtMYB43	53.85	143	66	0	1	429	1	143	3.00E-55	176
TcMYB23	AtMYB45	48.81	168	83	3	22	516	14	178	2.00E-54	172
TcMYB23	ATMYB29	42.03	207	120	2	1	621	1	200	3.00E-54	174
TcMYB23	ATMYB92	58.27	127	53	0	1	381	1	127	6.00E-54	173
TcMYB23	ATMYB14	45.63	206	96	5	1	570	1	200	2.00E-53	169
TcMYB23	ATMYB34	53.1	145	66	1	16	444	6	150	3.00E-53	170
TcMYB23	ATMYB95	56.62	136	59	1	1	408	1	132	3.00E-53	169
TcMYB23	ATMYB5	51.7	147	71	1	16	456	17	160	5.00E-53	168
TcMYB23	AtMYB40	56.93	137	59	0	1	411	1	137	5.00E-53	168
TcMYB23	ATMYB72	54	150	65	1	4	441	4	153	9.00E-53	169
TcMYB23	AtMYB51	52	150	67	2	16	450	6	155	1.00E-52	170
TcMYB23	AtMYB19	60	130	52	1	25	414	9	133	3.00E-52	166
TcMYB23	ATMYB99	44.89	176	77	2	4	471	3	178	3.00E-52	166
TcMYB23	ATMYB58	51.97	152	73	2	4	459	4	153	4.00E-52	166
TcMYB23	ATMYB15	60.34	116	46	0	1	348	1	116	1.00E-51	165
TcMYB23	ATMYB63	63.48	115	42	0	4	348	4	118	3.00E-51	165
TcMYB23	ATMYB13	48.17	164	81	1	1	480	1	164	3.00E-51	163
TcMYB23	ATMYB12	51.66	151	73	2	1	453	1	141	9.00E-51	166
TcMYB23	AtMYB10	52.59	135	64	0	16	420	8	142	1.00E-50	161
TcMYB23	ATMYB111	60.17	118	47	0	1	354	1	118	1.00E-50	164
TcMYB23	AtMYB18	58.14	129	54	1	28	414	8	135	3.00E-50	162
TcMYB23	AtMYB76	55.12	127	57	0	1	381	1	127	4.00E-50	163
TcMYB23	ATMYB37	49.1	167	73	3	1	465	1	161	7.00E-50	162
TcMYB23	ATMYB122	58.2	122	51	0	16	381	6	127	4.00E-49	160
TcMYB23	AtMYB47	54.26	129	59	0	1	387	1	129	4.00E-49	158
TcMYB23	ATMYB94	48.67	150	67	1	1	420	1	150	4.00E-48	157
TcMYB23	AtMYB36	50.35	141	69	1	1	420	1	141	9.00E-48	157
TcMYB23	ATMYB11	57.76	116	49	0	1	348	1	116	2.00E-47	156
TcMYB23	ATMYB31	58.12	117	49	0	1	351	1	117	3.00E-47	155
TcMYB23	AtMYB112	46.21	145	78	0	31	465	31	175	5.00E-47	152
TcMYB23	ATMYB66	62.5	104	39	0	37	348	17	120	9.00E-47	150
TcMYB23	ATMYB30	50.36	139	65	1	16	420	6	144	1.00E-46	153
TcMYB23	AtMYB60	56.9	116	50	0	1	348	1	116	2.00E-46	152
TcMYB23	ATMYB96	57.76	116	49	0	1	348	1	116	2.00E-46	154
TcMYB23	ATMYB57	56.03	116	51	0	34	381	25	140	3.00E-46	149
TcMYB23	ATMYB23	45.24	168	81	3	25	495	9	171	3.00E-46	149
TcMYB23	ATMYB123	61.11	108	42	0	25	348	11	118	3.00E-46	150
TcMYB23	ATMYB68	34.88	258	160	6	1	750	1	246	6.00E-46	153
TcMYB23	AtMYB82	61.68	107	41	0	25	345	9	115	1.00E-45	147
TcMYB23	AtMYB24	58.18	110	46	0	31	360	16	125	2.00E-45	147
TcMYB23	ATMYB0	58.33	108	45	0	28	351	12	119	3.00E-45	147
TcMYB23	ATMYB84	55.08	118	52	1	1	351	1	118	4.00E-45	149
TcMYB23	ATMYB38	54.62	119	53	1	1	354	1	119	6.00E-45	148
TcMYB23	ATMYB3	60.58	104	41	0	31	342	19	122	1.00E-44	145
TcMYB23	ATMYB87	49.01	151	70	4	1	432	1	149	3.00E-44	147
TcMYB23	ATMYB121	41.94	186	99	3	25	555	24	208	6.00E-44	145
TcMYB23	AtMYB108	35.21	213	128	1	22	630	15	227	3.00E-43	144
TcMYB23	AtMYB79	63.11	103	38	0	37	345	7	109	4.00E-43	142
TcMYB23	AtMYB81	57.55	106	45	0	25	342	17	122	5.00E-43	146
TcMYB23	AtMYB116	46.32	136	73	0	37	444	19	154	7.00E-43	142
TcMYB23	ATMYB33	60.58	104	41	0	34	345	32	135	1.00E-42	147
TcMYB23	ATMYB71	62.14	103	39	0	37	345	19	121	3.00E-42	140
TcMYB23	ATMYB65	55.26	114	51	0	4	345	31	144	6.00E-42	145
TcMYB23	ATMYB101	50.79	126	62	1	34	411	18	142	1.00E-41	144
TcMYB23	ATMYB120	55.93	118	52	1	34	387	26	142	3.00E-41	143
TcMYB23	AtMYB97	47.3	148	71	2	34	456	19	165	7.00E-41	140
TcMYB23	AtMYB62	43.88	139	78	0	28	444	17	155	9.00E-41	137

TcMYB23	ATMYB2	47.83	115	60	0	34	378	20	134	9.00E-41	137
TcMYB23	AtMYB114	55.77	104	46	0	34	345	8	111	9.00E-41	132
TcMYB23	ATMYB78	48.46	130	53	1	34	381	26	155	2.00E-39	134
TcMYB23	AtMYB27	52.25	111	53	0	25	357	6	116	4.00E-39	131
TcMYB23	ATMYB75	53.85	104	48	0	34	345	8	111	2.00E-38	130
TcMYB23	ATMYB59-3	53.27	107	50	0	25	345	5	111	4.00E-38	129
TcMYB23	ATMYB90	54.81	104	47	0	34	345	8	111	4.00E-38	129
TcMYB23	ATMYB48	54.37	103	47	0	37	345	8	110	3.00E-37	127
TcMYB23	AtMYB113	52.88	104	49	0	34	345	8	111	6.00E-37	126
TcMYB23	AtMYB104	32.35	272	181	7	25	831	13	256	4.00E-35	124
TcMYB23	ATMYB73	42.54	134	77	1	40	441	13	145	2.00E-30	110
TcMYB23	AtMYB115	49.02	102	52	1	25	330	153	253	3.00E-30	110
TcMYB23	ATMYB119	47.06	102	54	1	40	345	105	205	1.00E-29	110
TcMYB23	ATMYB118	46.3	108	57	2	25	345	184	289	2.00E-29	109
TcMYB23	ATMYB118	27.27	55	39	1	199	360	189	243	2.00E-04	35
TcMYB23	AtMYB98	46.73	107	57	1	25	345	212	317	5.00E-29	108
TcMYB23	AtMYB64	43.4	106	60	1	28	345	101	205	1.00E-28	107
TcMYB23	ATMYB44	50	102	51	1	40	345	6	106	4.00E-28	103
TcMYB23	ATMYB25	50.93	108	52	3	25	345	46	150	1.00E-27	103
TcMYB23	AtMYB70	41.43	140	76	2	40	441	13	151	2.00E-27	102
TcMYB23	ATMYB105	44.54	119	65	3	4	357	98	211	5.00E-27	101
TcMYB23	ATMYB77	49.02	102	52	1	40	345	6	106	6.00E-27	100
TcMYB23	AtMYB109	51.46	103	49	2	40	345	56	156	6.00E-27	102
TcMYB23	AtMYB1	48.08	104	54	1	40	351	55	157	2.00E-26	100
TcMYB23	ATMYB52	45.1	102	56	1	40	345	5	105	5.00E-26	97.1
TcMYB23	ATMYB54	46.6	103	54	2	40	345	6	106	2.00E-25	95.1
TcMYB23	AtMYB117	45.63	103	55	2	40	345	98	198	3.00E-25	97.1
TcMYB23	AtMYB100	37.07	116	73	1	25	372	21	135	1.00E-24	92.8
TcMYB23	ATMYB69	40.87	115	67	2	4	345	6	119	3.00E-24	92.4
TcMYB23	AtMYB56	40.74	108	64	1	22	345	87	193	3.00E-23	90.9
TcMYB23	ATMYB110	40.57	106	63	1	40	357	67	171	6.00E-23	89.7
TcMYB23	ATMYB88	38.66	119	72	2	10	363	19	136	6.00E-22	88.6
TcMYB23	AtMYB124	33.57	140	93	2	49	468	28	164	4.00E-21	85.9
TcMYB23	AtMYB22	36.21	116	74	2	25	372	49	162	9.00E-21	82.8
TcMYB23	ATMYB91	41.24	97	55	1	40	324	4	100	1.00E-18	78.2
TcMYB23	AtMYB89	36.04	111	71	1	25	357	51	160	2.00E-18	75.1
TcMYB24	AtMYB93	52.56	293	103	8	1	771	1	292	3.00E-89	269
TcMYB24	ATMYB16	69.61	181	48	3	1	522	1	181	5.00E-87	262
TcMYB24	AtMYB107	83.97	131	21	0	1	393	1	131	1.00E-84	255
TcMYB24	AtMYB9	85.5	131	19	0	1	393	1	131	3.00E-84	255
TcMYB24	ATMYB106	66.67	180	53	3	10	528	47	225	2.00E-83	254
TcMYB24	ATMYB92	60.66	211	64	5	1	576	1	211	2.00E-80	245
TcMYB24	AtMYB53	77.24	145	33	0	1	435	1	145	9.00E-80	243
TcMYB24	ATMYB102	74.83	143	36	1	1	429	1	141	2.00E-79	243
TcMYB24	AtMYB17	54.27	234	88	5	1	645	1	233	1.00E-78	239
TcMYB24	AtMYB74	75	144	35	2	1	429	1	142	1.00E-78	240
TcMYB24	AtMYB41	56.22	217	87	4	1	627	1	216	2.00E-78	238
TcMYB24	AtMYB49	72.03	143	40	1	1	429	1	141	4.00E-73	226
TcMYB24	AtMYB85	73.44	128	34	0	1	384	1	128	1.00E-68	213
TcMYB24	MYB8	66.19	139	43	1	1	405	1	139	2.00E-67	207
TcMYB24	AtMYB6	69.29	127	39	0	1	381	1	127	3.00E-67	207
TcMYB24	AtMYB43	70.23	131	39	0	1	393	1	131	3.00E-67	211
TcMYB24	ATMYB29	65.07	146	51	0	1	438	1	146	3.00E-66	208
TcMYB24	ATMYB28	65.52	145	50	0	1	435	1	145	4.00E-66	209
TcMYB24	MYB7	67.72	127	41	0	1	381	1	127	5.00E-66	206
TcMYB24	ATMYB4	67.97	128	41	0	1	384	1	128	6.00E-66	206
TcMYB24	AtMYB32	68.75	128	40	0	1	384	1	128	6.00E-66	206
TcMYB24	AtMYB20	69.53	128	39	0	1	384	1	128	1.00E-65	205
TcMYB24	ATMYB3	67.72	127	41	0	1	381	1	127	3.00E-65	203
TcMYB24	AtMYB42	68.42	133	42	0	1	399	1	133	3.00E-65	204
TcMYB24	ATMYB34	71.09	128	37	0	1	384	1	128	5.00E-65	204
TcMYB24	AtMYB51	70.31	128	37	1	1	381	1	128	2.00E-64	204
TcMYB24	AtMYB76	70.31	128	38	0	1	384	1	128	4.00E-64	203
TcMYB24	ATMYB14	75	116	29	0	1	348	1	116	5.00E-64	200
TcMYB24	ATMYB5	67.2	125	41	0	10	384	15	139	7.00E-64	199
TcMYB24	ATMYB80	58.23	158	66	2	1	474	1	153	4.00E-63	200
TcMYB24	ATMYB122	67.18	131	43	0	1	393	1	131	8.00E-63	199
TcMYB24	ATMYB30	48.39	217	107	4	1	636	1	215	3.00E-62	197
TcMYB24	ATMYB15	72.41	116	32	0	1	348	1	116	1.00E-61	195
TcMYB24	ATMYB72	73.04	115	31	0	4	348	4	118	7.00E-61	193
TcMYB24	AtMYB50	63.64	132	48	0	1	396	1	132	2.00E-60	193
TcMYB24	ATMYB13	74.14	116	30	0	1	348	1	116	2.00E-60	191
TcMYB24	ATMYB99	63.91	133	41	1	4	381	3	135	2.00E-60	190
TcMYB24	ATMYB86	66.41	128	43	0	1	384	1	128	4.00E-60	193
TcMYB24	ATMYB94	69.75	119	36	0	1	357	1	119	5.00E-60	192
TcMYB24	AtMYB40	61.36	132	51	0	1	396	1	132	5.00E-60	190
TcMYB24	AtMYB60	60.69	145	57	1	1	435	1	142	6.00E-60	190
TcMYB24	ATMYB63	70.94	117	34	0	4	354	4	120	9.00E-60	190
TcMYB24	ATMYB61	64.39	132	47	0	1	396	1	132	2.00E-59	192
TcMYB24	ATMYB35	59.7	134	54	0	1	402	1	134	2.00E-59	190

TcMYB24	ATMYB96	69.83	116	35	0	1	348	1	116	5.00E-59	190
TcMYB24	ATMYB58	70.43	115	34	0	4	348	4	118	3.00E-58	186
TcMYB24	ATMYB95	63.85	130	47	0	1	390	1	130	4.00E-58	185
TcMYB24	ATMYB31	67.23	119	39	0	1	357	1	119	5.00E-58	187
TcMYB24	ATMYB67	63.64	121	44	0	16	378	16	136	4.00E-57	184
TcMYB24	AtMYB10	65.55	119	41	0	7	363	5	123	7.00E-57	181
TcMYB24	ATMYB55	59.03	144	47	1	1	396	1	144	4.00E-56	182
TcMYB24	ATMYB111	67.24	116	38	0	1	348	1	116	4.00E-56	182
TcMYB24	ATMYB12	66.38	116	39	0	1	348	1	116	2.00E-55	181
TcMYB24	AtMYB47	61.72	128	49	0	1	384	1	128	9.00E-55	176
TcMYB24	ATMYB11	66.38	116	39	0	1	348	1	116	4.00E-54	177
TcMYB24	AtMYB103	58.59	128	53	0	1	384	1	128	7.00E-53	174
TcMYB24	ATMYB37	61.79	123	46	1	1	366	1	123	2.00E-52	172
TcMYB24	ATMYB38	54	150	64	2	1	435	1	150	2.00E-52	171
TcMYB24	AtMYB36	65.25	118	40	1	1	351	1	118	2.00E-51	170
TcMYB24	ATMYB26	57.94	126	44	1	1	351	1	126	7.00E-51	169
TcMYB24	ATMYB84	63.56	118	42	1	1	351	1	118	4.00E-50	166
TcMYB24	ATMYB68	63.56	118	42	1	1	351	1	118	5.00E-50	167
TcMYB24	ATMYB87	60.17	118	46	1	1	351	1	118	2.00E-48	161
TcMYB24	ATMYB33	58.54	123	40	1	10	345	13	135	3.00E-48	166
TcMYB24	AtMYB83	66.67	105	35	0	34	348	30	134	2.00E-47	159
TcMYB24	ATMYB101	64.76	105	37	0	31	345	17	121	9.00E-47	161
TcMYB24	ATMYB65	65.38	104	36	0	34	345	41	144	1.00E-46	162
TcMYB24	ATMYB46	62.04	108	41	0	34	357	18	125	2.00E-46	155
TcMYB24	ATMYB71	63.55	107	39	0	25	345	15	121	1.00E-45	152
TcMYB24	ATMYB123	63.81	105	38	0	34	348	14	118	1.00E-45	152
TcMYB24	ATMYB66	58.88	107	44	0	28	348	14	120	2.00E-45	150
TcMYB24	AtMYB114	61.9	105	40	0	31	345	7	111	2.00E-45	147
TcMYB24	AtMYB24	60.55	109	43	0	34	360	17	125	5.00E-45	149
TcMYB24	AtMYB79	65.05	103	36	0	37	345	7	109	7.00E-45	150
TcMYB24	AtMYB19	65.09	106	37	0	37	354	13	118	8.00E-45	150
TcMYB24	ATMYB23	57.41	108	46	0	25	348	9	116	1.00E-44	148
TcMYB24	AtMYB81	57.63	118	46	2	22	363	12	128	1.00E-44	154
TcMYB24	ATMYB120	61.82	110	42	1	34	363	26	134	2.00E-44	155
TcMYB24	ATMYB121	62.26	106	40	0	25	342	24	129	4.00E-44	149
TcMYB24	ATMYB3	56.67	120	52	0	4	363	10	129	7.00E-44	146
TcMYB24	AtMYB112	62.14	103	39	0	34	342	32	134	8.00E-44	147
TcMYB24	ATMYB57	53.33	120	56	0	22	381	21	140	1.00E-43	145
TcMYB24	AtMYB108	58.26	115	48	0	22	366	15	129	1.00E-43	149
TcMYB24	AtMYB82	56.64	113	46	1	16	345	3	115	1.00E-43	145
TcMYB24	ATMYB75	60.95	105	41	0	31	345	7	111	2.00E-43	146
TcMYB24	AtMYB18	63.21	106	39	0	37	354	11	116	3.00E-43	146
TcMYB24	AtMYB113	57.39	115	49	1	1	345	1	111	7.00E-43	144
TcMYB24	AtMYB45	52.67	131	62	1	37	429	19	148	1.00E-42	144
TcMYB24	ATMYB0	56.19	105	46	0	37	351	15	119	1.00E-42	143
TcMYB24	AtMYB97	59.22	103	42	0	34	342	19	121	4.00E-42	146
TcMYB24	ATMYB90	60	105	42	0	31	345	7	111	4.00E-42	142
TcMYB24	AtMYB116	54.78	115	52	0	37	381	19	133	6.00E-42	143
TcMYB24	ATMYB2	50.43	115	57	0	22	366	16	130	2.00E-40	139
TcMYB24	AtMYB62	53.91	115	53	0	22	366	15	129	4.00E-40	138
TcMYB24	AtMYB27	57.94	107	45	0	25	345	6	112	2.00E-39	135
TcMYB24	ATMYB48	55.14	107	48	0	25	345	4	110	5.00E-39	134
TcMYB24	ATMYB78	49.61	129	51	1	22	366	22	150	3.00E-38	134
TcMYB24	ATMYB59-3	55.34	103	46	0	37	345	9	111	5.00E-38	131
TcMYB24	AtMYB104	49.07	108	55	1	40	363	18	124	1.00E-31	117
TcMYB24	ATMYB73	50.98	102	50	1	40	345	13	113	1.00E-30	113
TcMYB24	AtMYB70	50.98	102	50	1	40	345	13	113	4.00E-30	111
TcMYB24	ATMYB44	50	102	51	1	40	345	6	106	8.00E-30	110
TcMYB24	ATMYB77	49.02	102	52	1	40	345	6	106	9.00E-29	107
TcMYB24	ATMYB25	42.07	145	81	3	40	465	50	192	1.00E-28	108
TcMYB24	AtMYB115	36.42	173	105	6	40	543	158	321	3.00E-28	107
TcMYB24	AtMYB1	47.22	108	53	2	40	351	55	157	2.00E-27	105
TcMYB24	ATMYB54	43.51	131	73	3	40	429	6	128	2.00E-27	102
TcMYB24	ATMYB119	47.62	105	54	2	34	345	103	205	3.00E-27	105
TcMYB24	ATMYB119	27.27	55	39	1	199	360	105	159	9.00E-04	33.5
TcMYB24	AtMYB98	41.18	136	78	3	34	435	215	341	6.00E-27	104
TcMYB24	ATMYB52	47.57	103	53	2	40	345	5	105	1.00E-26	100
TcMYB24	AtMYB64	45.63	103	55	2	40	345	105	205	3.00E-26	102
TcMYB24	AtMYB109	48.54	103	52	2	40	345	56	156	7.00E-26	101
TcMYB24	ATMYB118	44.12	102	57	1	40	345	189	289	2.00E-25	100
TcMYB24	ATMYB118	30	60	41	1	184	360	184	243	2.00E-04	35.4
TcMYB24	ATMYB69	38.05	113	70	2	7	345	13	119	5.00E-24	93.2
TcMYB24	AtMYB56	44.12	102	57	1	40	345	93	193	1.00E-23	94
TcMYB24	ATMYB105	42.86	105	59	2	34	345	105	207	1.00E-23	94
TcMYB24	AtMYB117	43.81	105	58	2	34	345	96	198	2.00E-23	94
TcMYB24	AtMYB100	43.69	103	57	2	25	330	21	121	2.00E-22	88.6
TcMYB24	ATMYB110	40	105	63	1	40	354	67	170	3.00E-21	86.7
TcMYB24	AtMYB22	38.24	102	63	2	25	330	49	148	4.00E-19	79.3
TcMYB24	AtMYB22	25	52	39	0	184	339	49	100	2.00E-04	35
TcMYB24	AtMYB89	38	100	62	1	43	342	57	155	4.00E-18	75.1

TcMYB24	ATMYB91	35.64	101	63	1	49	345	7	107	6.00E-18	77.4
TcMYB24	ATMYB88	38.38	99	61	1	49	345	33	130	3.00E-17	75.9
TcMYB24	AtMYB124	40.4	99	59	1	49	345	28	125	5.00E-17	75.1
TcMYB25	AtMYB17	40	240	116	7	7	642	44	283	1.00E-47	153
TcMYB25	AtMYB85	45.51	178	78	3	7	483	44	221	5.00E-47	151
TcMYB25	ATMYB92	49.11	169	86	2	7	513	44	208	1.00E-46	152
TcMYB25	AtMYB9	37.5	264	123	7	7	672	44	299	2.00E-46	151
TcMYB25	AtMYB74	39.04	251	141	6	7	723	45	283	4.00E-46	150
TcMYB25	ATMYB102	71.84	103	25	1	7	303	44	146	7.00E-46	150
TcMYB25	AtMYB49	52.76	163	69	5	7	471	44	201	1.00E-45	149
TcMYB25	MYB7	55.12	127	56	1	4	381	43	169	1.00E-45	147
TcMYB25	AtMYB53	80.72	83	16	0	7	255	44	126	1.00E-45	149
TcMYB25	AtMYB107	78.31	83	18	0	7	255	44	126	1.00E-45	149
TcMYB25	AtMYB41	78.57	84	18	0	7	258	44	127	4.00E-45	146
TcMYB25	AtMYB32	66.35	104	35	0	4	315	43	146	5.00E-45	146
TcMYB25	ATMYB15	44.44	207	99	7	7	579	44	234	7.00E-45	146
TcMYB25	ATMYB3	60.34	116	46	0	4	351	43	158	4.00E-44	143
TcMYB25	AtMYB6	73.81	84	22	0	4	255	43	126	8.00E-44	142
TcMYB25	ATMYB4	73.81	84	22	0	4	255	43	126	1.00E-43	143
TcMYB25	ATMYB16	78.31	83	18	0	7	255	44	126	3.00E-43	143
TcMYB25	ATMYB14	84.93	73	11	0	7	225	44	116	3.00E-43	140
TcMYB25	ATMYB106	78.31	83	18	0	7	255	87	169	4.00E-43	144
TcMYB25	ATMYB5	73.49	83	22	0	7	255	55	137	6.00E-43	140
TcMYB25	AtMYB20	75.9	83	20	0	7	255	44	126	7.00E-43	140
TcMYB25	AtMYB42	50.68	148	65	2	7	426	44	180	1.00E-42	140
TcMYB25	ATMYB13	42.51	207	117	6	7	621	44	227	1.00E-42	139
TcMYB25	ATMYB58	40.09	212	120	5	7	621	46	232	2.00E-42	139
TcMYB25	MYB8	70.24	84	25	0	4	255	43	126	2.00E-42	137
TcMYB25	ATMYB99	73.49	83	22	0	7	255	52	134	2.00E-42	138
TcMYB25	AtMYB10	67.01	97	27	1	7	282	46	142	3.00E-42	138
TcMYB25	AtMYB43	75.9	83	20	0	7	255	44	126	3.00E-42	140
TcMYB25	AtMYB93	75.9	83	20	0	7	255	44	126	3.00E-42	141
TcMYB25	ATMYB28	46.75	169	90	3	7	513	44	199	5.00E-42	140
TcMYB25	ATMYB61	75.9	83	20	0	7	255	44	126	1.00E-41	139
TcMYB25	ATMYB72	70.21	94	25	1	7	279	46	139	3.00E-41	137
TcMYB25	ATMYB35	59.13	115	46	3	7	348	44	151	4.00E-41	137
TcMYB25	ATMYB122	45.2	177	78	3	7	480	44	218	7.00E-41	137
TcMYB25	AtMYB51	73.49	83	22	0	7	255	45	127	8.00E-41	137
TcMYB25	ATMYB86	75.9	83	20	0	7	255	44	126	1.00E-40	136
TcMYB25	AtMYB50	72.29	83	23	0	7	255	44	126	2.00E-40	135
TcMYB25	AtMYB76	49.03	155	79	3	7	471	44	188	2.00E-40	135
TcMYB25	ATMYB80	64.58	96	34	1	7	294	44	136	3.00E-40	135
TcMYB25	AtMYB40	64.84	91	32	0	7	279	44	134	6.00E-40	132
TcMYB25	ATMYB95	51.59	126	61	0	7	384	44	169	2.00E-39	131
TcMYB25	ATMYB63	80.82	73	14	0	7	225	46	118	2.00E-39	132
TcMYB25	ATMYB34	72.29	83	23	0	7	255	44	126	3.00E-39	131
TcMYB25	ATMYB29	47.85	163	78	4	7	474	44	206	4.00E-39	132
TcMYB25	ATMYB67	50	124	62	1	7	378	54	176	6.00E-39	131
TcMYB25	ATMYB55	73.17	82	22	0	10	255	57	138	1.00E-38	131
TcMYB25	AtMYB47	35.56	225	140	5	7	666	44	246	3.00E-38	128
TcMYB25	AtMYB83	79.45	73	15	0	7	225	62	134	7.00E-38	129
TcMYB25	ATMYB46	43.43	175	97	4	7	525	50	203	1.00E-37	127
TcMYB25	ATMYB23	56.07	107	44	1	10	321	45	151	1.00E-36	123
TcMYB25	ATMYB11	75.34	73	18	0	7	225	44	116	3.00E-36	125
TcMYB25	ATMYB123	78.08	73	16	0	7	225	46	118	4.00E-36	122
TcMYB25	ATMYB12	38.73	204	118	7	7	597	44	231	9.00E-36	124
TcMYB25	ATMYB66	70.83	72	21	0	10	225	49	120	1.00E-35	120
TcMYB25	AtMYB103	73.97	73	19	0	7	225	44	116	7.00E-35	122
TcMYB25	ATMYB37	39.41	170	103	1	1	510	43	211	9.00E-35	120
TcMYB25	AtMYB60	70.51	78	23	0	10	243	45	122	9.00E-35	119
TcMYB25	AtMYB82	70.83	72	21	0	7	222	44	115	1.00E-34	117
TcMYB25	ATMYB101	45.03	151	83	4	7	459	50	190	2.00E-34	122
TcMYB25	ATMYB0	67.12	73	24	0	10	228	47	119	2.00E-34	117
TcMYB25	ATMYB111	71.23	73	21	0	7	225	44	116	2.00E-34	120
TcMYB25	ATMYB31	73.33	75	20	0	10	234	45	119	2.00E-34	119
TcMYB25	ATMYB30	69.23	78	24	0	10	243	45	122	4.00E-34	119
TcMYB25	AtMYB19	45.89	146	76	1	7	435	44	189	5.00E-34	117
TcMYB25	AtMYB24	59.34	91	37	1	4	276	48	135	1.00E-33	115
TcMYB25	ATMYB94	70.67	75	22	0	10	234	45	119	3.00E-33	117
TcMYB25	ATMYB3	50	124	61	3	4	372	51	169	6.00E-33	113
TcMYB25	AtMYB114	64.86	74	26	0	1	222	38	111	6.00E-33	110
TcMYB25	ATMYB96	69.33	75	23	0	10	234	45	119	7.00E-33	116
TcMYB25	ATMYB26	69.44	72	22	0	13	228	55	126	9.00E-33	116
TcMYB25	AtMYB79	70.83	72	21	0	7	222	38	109	9.00E-33	114
TcMYB25	ATMYB71	70.83	72	21	0	7	222	50	121	2.00E-32	113
TcMYB25	ATMYB68	69.86	73	22	0	10	228	46	118	4.00E-32	114
TcMYB25	ATMYB84	68.49	73	23	0	10	228	46	118	5.00E-32	113
TcMYB25	AtMYB62	33.6	247	161	8	7	738	51	279	6.00E-32	112
TcMYB25	AtMYB36	68.49	73	23	0	10	228	46	118	7.00E-32	113
TcMYB25	ATMYB38	51.4	107	44	1	7	303	45	151	1.00E-31	112

TcMYB25	AtMYB116	57.14	84	36	0	4	255	49	132	1.00E-31	111
TcMYB25	ATMYB120	66.23	77	26	1	10	240	59	134	2.00E-31	114
TcMYB25	ATMYB57	64.86	74	26	0	4	225	56	129	2.00E-31	108
TcMYB25	AtMYB18	57.61	92	39	0	7	282	42	133	2.00E-31	110
TcMYB25	AtMYB97	64.47	76	27	1	13	240	53	127	3.00E-31	112
TcMYB25	AtMYB81	56.25	96	38	2	7	282	52	146	4.00E-31	112
TcMYB25	ATMYB87	65.75	73	25	0	10	228	46	118	6.00E-31	110
TcMYB25	ATMYB2	36.84	171	97	3	4	483	51	220	9.00E-31	108
TcMYB25	ATMYB75	65.28	72	25	0	7	222	40	111	1.00E-30	108
TcMYB25	ATMYB90	63.51	74	27	0	1	222	38	111	1.00E-30	108
TcMYB25	ATMYB121	69.01	71	22	0	7	219	59	129	1.00E-30	108
TcMYB25	ATMYB59-3	62.5	72	27	0	7	222	40	111	2.00E-30	107
TcMYB25	AtMYB27	33.94	221	145	6	1	660	39	235	2.00E-30	107
TcMYB25	ATMYB48	62.5	72	27	0	7	222	39	110	2.00E-30	107
TcMYB25	ATMYB33	65.71	70	24	0	13	222	66	135	3.00E-30	111
TcMYB25	AtMYB113	60.81	74	29	0	1	222	38	111	1.00E-29	105
TcMYB25	ATMYB65	62.86	70	26	0	13	222	75	144	3.00E-29	108
TcMYB25	AtMYB112	59.76	82	31	1	4	243	63	144	4.00E-29	103
TcMYB25	AtMYB108	60.76	79	31	0	7	243	51	129	4.00E-29	105
TcMYB25	AtMYB45	60.27	73	29	0	1	219	48	120	1.00E-27	100
TcMYB25	ATMYB78	50.54	93	32	1	7	243	58	150	3.00E-24	92.4
TcMYB25	AtMYB104	41.44	111	59	3	10	324	49	158	1.00E-22	88.6
TcMYB25	ATMYB77	40.48	126	75	3	19	396	39	160	2.00E-22	87
TcMYB25	ATMYB25	58.82	68	28	0	19	222	83	150	4.00E-22	87
TcMYB25	AtMYB98	52.94	68	32	0	19	222	250	317	6.00E-22	87
TcMYB25	AtMYB98	32.76	58	38	1	46	216	207	264	2.00E-06	40.8
TcMYB25	ATMYB73	55.88	68	30	0	19	222	46	113	9.00E-22	85.5
TcMYB25	ATMYB73	36.17	47	29	1	76	213	13	59	5.00E-04	33.1
TcMYB25	ATMYB44	33.33	159	71	3	19	390	39	197	1.00E-21	85.1
TcMYB25	AtMYB1	54.29	70	32	0	19	228	88	157	2.00E-21	85.5
TcMYB25	AtMYB1	36.21	58	36	1	61	231	50	107	2.00E-05	37.4
TcMYB25	AtMYB70	54.41	68	31	0	19	222	46	113	3.00E-21	84
TcMYB25	AtMYB70	32.73	55	36	1	76	237	13	67	2.00E-04	34.3
TcMYB25	ATMYB118	47.06	85	43	1	19	267	222	306	4.00E-21	84.7
TcMYB25	ATMYB118	30	60	41	1	61	237	184	243	8.00E-06	38.9
TcMYB25	AtMYB115	52.17	69	33	1	19	225	191	258	6.00E-21	83.6
TcMYB25	AtMYB115	26.23	61	43	2	49	225	148	208	3.00E-05	37
TcMYB25	ATMYB119	50	68	34	0	19	222	138	205	1.00E-20	83.6
TcMYB25	ATMYB119	29.09	55	38	1	76	237	105	159	6.00E-05	36.2
TcMYB25	AtMYB100	33.54	161	102	6	19	486	59	205	1.00E-20	80.9
TcMYB25	ATMYB54	54.41	68	31	0	19	222	39	106	2.00E-20	80.5
TcMYB25	ATMYB54	33.96	53	34	1	76	231	6	58	6.00E-05	35.8
TcMYB25	AtMYB109	55.88	68	30	0	19	222	89	156	3.00E-20	82
TcMYB25	AtMYB109	35.85	53	33	1	76	231	56	108	0.001	32.3
TcMYB25	ATMYB52	54.41	68	31	0	19	222	38	105	3.00E-20	80.1
TcMYB25	ATMYB52	32.08	53	35	1	76	231	5	57	3.00E-04	33.5
TcMYB25	AtMYB64	48.53	68	35	0	19	222	138	205	1.00E-19	80.5
TcMYB25	AtMYB64	29.41	51	35	1	67	216	102	152	1.00E-04	35
TcMYB25	ATMYB69	43.27	104	52	2	19	309	52	154	2.00E-19	78.2
TcMYB25	ATMYB105	36.89	122	60	2	19	333	140	261	4.00E-19	78.2
TcMYB25	AtMYB117	50	68	34	0	19	222	131	198	7.00E-19	77.8
TcMYB25	ATMYB110	47.83	69	36	0	19	225	100	168	3.00E-18	75.5
TcMYB25	AtMYB56	47.06	68	36	0	19	222	126	193	2.00E-17	73.6
TcMYB25	AtMYB22	44.44	63	35	0	19	207	86	148	7.00E-17	70.9
TcMYB25	AtMYB124	47.69	65	34	0	28	222	61	125	7.00E-16	69.3
TcMYB25	ATMYB88	47.69	65	34	0	28	222	66	130	8.00E-16	69.3
TcMYB25	AtMYB89	45.07	71	39	1	19	231	89	158	2.00E-15	65.9
TcMYB25	ATMYB91	41.43	70	41	0	13	222	38	107	5.00E-15	66.6
TcMYB26	ATMYB67	65.81	117	40	0	4	354	9	125	9.00E-58	177
TcMYB26	AtMYB103	55.13	156	69	2	19	483	4	155	3.00E-56	176
TcMYB26	ATMYB15	42.55	188	108	1	25	588	6	192	3.00E-53	165
TcMYB26	ATMYB26	63.64	121	35	1	19	354	4	124	3.00E-53	167
TcMYB26	AtMYB50	64.29	112	40	0	19	354	4	115	4.00E-53	166
TcMYB26	ATMYB61	63.39	112	41	0	19	354	4	115	4.00E-52	165
TcMYB26	AtMYB41	53.25	154	70	2	25	480	6	157	5.00E-52	162
TcMYB26	ATMYB86	64.29	112	40	0	19	354	4	115	5.00E-52	164
TcMYB26	ATMYB72	50.3	165	82	1	25	519	8	170	7.00E-52	162
TcMYB26	AtMYB107	62.73	110	41	0	25	354	6	115	1.00E-51	162
TcMYB26	ATMYB13	42	200	101	3	25	579	6	205	2.00E-51	159
TcMYB26	ATMYB58	65.45	110	38	0	25	354	8	117	2.00E-51	160
TcMYB26	ATMYB63	53.52	142	63	1	25	441	8	149	7.00E-51	159
TcMYB26	ATMYB16	62.73	110	41	0	25	354	6	115	8.00E-51	160
TcMYB26	ATMYB106	62.73	110	41	0	25	354	49	158	9.00E-51	162
TcMYB26	ATMYB102	62.73	110	41	0	25	354	6	115	1.00E-50	160
TcMYB26	ATMYB96	44.26	183	80	3	25	507	6	188	2.00E-50	160
TcMYB26	AtMYB74	63.96	111	39	1	25	354	6	116	2.00E-50	159
TcMYB26	ATMYB3	61.82	110	42	0	25	354	6	115	3.00E-50	157
TcMYB26	ATMYB30	42.86	196	94	2	25	558	6	201	4.00E-50	158
TcMYB26	ATMYB14	60.5	119	41	1	25	363	6	124	5.00E-50	156
TcMYB26	ATMYB55	58.87	124	39	1	19	354	4	127	6.00E-50	159

TcMYB26	ATMYB34	60.91	110	43	0	25	354	6	115	6.00E-50	157
TcMYB26	ATMYB80	60.18	113	45	0	25	363	6	118	1.00E-49	157
TcMYB26	AtMYB9	61.82	110	42	0	25	354	6	115	2.00E-49	157
TcMYB26	AtMYB83	51.37	146	69	1	31	462	26	171	3.00E-49	157
TcMYB26	AtMYB93	61.82	110	42	0	25	354	6	115	3.00E-49	157
TcMYB26	AtMYB49	65.38	104	36	0	43	354	12	115	6.00E-49	155
TcMYB26	ATMYB94	60.91	110	43	0	25	354	6	115	9.00E-49	155
TcMYB26	MYB7	59.09	110	45	0	25	354	6	115	1.00E-48	153
TcMYB26	AtMYB32	60	110	44	0	25	354	6	115	3.00E-48	152
TcMYB26	AtMYB6	58.18	110	46	0	25	354	6	115	3.00E-48	151
TcMYB26	MYB8	46.95	164	82	3	25	501	6	166	3.00E-48	150
TcMYB26	AtMYB51	58.18	110	46	0	25	354	7	116	4.00E-48	154
TcMYB26	ATMYB4	59.09	110	45	0	25	354	6	115	4.00E-48	152
TcMYB26	AtMYB53	64.42	104	37	0	43	354	12	115	4.00E-48	153
TcMYB26	ATMYB111	58.47	118	49	0	25	378	6	123	6.00E-48	153
TcMYB26	ATMYB37	57.03	128	54	1	25	405	6	133	6.00E-48	153
TcMYB26	ATMYB31	60.91	110	43	0	25	354	6	115	6.00E-48	153
TcMYB26	AtMYB19	63.55	107	39	0	34	354	9	115	7.00E-48	151
TcMYB26	ATMYB95	63.46	104	38	0	43	354	12	115	8.00E-48	151
TcMYB26	ATMYB28	59.09	110	45	0	25	354	6	115	1.00E-47	153
TcMYB26	ATMYB29	59.63	109	44	0	25	351	6	114	2.00E-47	152
TcMYB26	AtMYB60	59.09	110	45	0	25	354	6	115	2.00E-47	150
TcMYB26	ATMYB46	52.63	133	59	1	37	423	16	148	2.00E-47	150
TcMYB26	AtMYB17	59.09	110	45	0	25	354	6	115	3.00E-47	150
TcMYB26	ATMYB92	61.54	104	40	0	43	354	12	115	5.00E-47	150
TcMYB26	ATMYB5	58.18	110	46	0	25	354	17	126	7.00E-47	148
TcMYB26	ATMYB122	56.88	109	47	0	25	351	6	114	7.00E-47	150
TcMYB26	AtMYB10	57.02	121	52	1	25	387	8	127	8.00E-47	147
TcMYB26	ATMYB12	58.77	114	47	0	25	366	6	119	9.00E-47	151
TcMYB26	ATMYB38	60.36	111	43	1	25	354	6	116	9.00E-47	149
TcMYB26	ATMYB35	56.36	110	48	0	25	354	6	115	2.00E-46	149
TcMYB26	AtMYB76	57.8	109	46	0	28	354	7	115	3.00E-46	149
TcMYB26	ATMYB84	61.26	111	42	1	25	354	6	116	5.00E-46	147
TcMYB26	ATMYB11	57.02	114	49	0	25	366	6	119	1.00E-45	147
TcMYB26	AtMYB36	58.62	116	47	1	25	369	6	121	2.00E-45	147
TcMYB26	AtMYB18	63.11	103	38	0	46	354	11	113	2.00E-45	145
TcMYB26	ATMYB66	56.19	105	46	0	46	360	17	121	2.00E-45	142
TcMYB26	ATMYB123	60.75	107	42	0	34	354	11	117	3.00E-45	144
TcMYB26	AtMYB82	57.94	107	45	0	34	354	9	115	3.00E-45	142
TcMYB26	ATMYB23	40.64	187	111	2	13	573	2	158	6.00E-45	142
TcMYB26	AtMYB45	57.01	107	46	0	31	351	14	120	6.00E-45	143
TcMYB26	AtMYB42	58.18	110	46	0	25	354	6	115	8.00E-45	144
TcMYB26	ATMYB87	58.56	111	45	1	25	354	6	116	9.00E-45	144
TcMYB26	ATMYB68	60.36	111	43	1	25	354	6	116	2.00E-44	145
TcMYB26	AtMYB47	59.62	104	42	0	43	354	12	115	4.00E-44	141
TcMYB26	AtMYB85	56.36	110	48	0	25	354	6	115	8.00E-44	140
TcMYB26	AtMYB20	56.36	110	48	0	25	354	6	115	8.00E-44	141
TcMYB26	AtMYB43	57.27	110	47	0	25	354	6	115	8.00E-44	142
TcMYB26	AtMYB112	44.38	169	78	3	43	501	32	200	7.00E-43	137
TcMYB26	ATMYB0	54.37	103	47	0	46	354	15	117	1.00E-42	136
TcMYB26	AtMYB24	53.39	118	52	1	43	387	17	134	2.00E-42	135
TcMYB26	AtMYB40	54.55	110	50	0	25	354	6	115	2.00E-42	137
TcMYB26	AtMYB108	43.29	164	92	1	43	531	19	182	3.00E-42	138
TcMYB26	ATMYB3	58.25	103	43	0	43	351	20	122	5.00E-42	135
TcMYB26	ATMYB57	54.37	103	47	0	43	351	25	127	3.00E-41	132
TcMYB26	ATMYB65	54.46	112	50	1	22	354	33	144	3.00E-41	139
TcMYB26	ATMYB33	57.14	105	45	0	40	354	31	135	4.00E-41	139
TcMYB26	AtMYB81	53.77	106	49	0	34	351	17	122	5.00E-41	137
TcMYB26	ATMYB121	47.01	134	69	2	7	402	18	148	2.00E-40	132
TcMYB26	AtMYB114	49.54	109	55	0	31	357	4	112	6.00E-40	127
TcMYB26	ATMYB101	52.83	106	50	0	37	354	16	121	6.00E-40	135
TcMYB26	AtMYB79	56.31	103	45	0	46	354	7	109	1.00E-39	130
TcMYB26	ATMYB99	48.72	117	53	1	25	354	7	123	1.00E-39	129
TcMYB26	ATMYB71	55.34	103	46	0	46	354	19	121	1.00E-38	127
TcMYB26	AtMYB97	54.37	103	47	0	43	351	19	121	2.00E-38	129
TcMYB26	ATMYB75	48.62	109	56	0	31	357	4	112	6.00E-38	125
TcMYB26	ATMYB2	40.91	154	87	3	43	492	20	167	8.00E-38	125
TcMYB26	ATMYB90	48.62	109	56	0	31	357	4	112	3.00E-37	123
TcMYB26	ATMYB120	53.4	103	48	0	43	351	26	128	3.00E-37	128
TcMYB26	AtMYB113	46.79	109	58	0	31	357	4	112	7.00E-37	122
TcMYB26	AtMYB27	51.4	107	52	0	34	354	6	112	1.00E-36	121
TcMYB26	ATMYB78	50.43	117	44	1	43	351	26	142	2.00E-36	122
TcMYB26	AtMYB116	50.49	103	51	0	46	354	19	121	9.00E-36	120
TcMYB26	AtMYB62	50.96	104	51	0	43	354	19	122	9.00E-36	120
TcMYB26	ATMYB59-3	34.43	183	120	3	46	594	9	171	1.00E-35	119
TcMYB26	ATMYB48	46.6	103	55	0	46	354	8	110	7.00E-35	117
TcMYB26	AtMYB104	42.2	109	63	0	34	360	13	121	2.00E-32	113
TcMYB26	ATMYB105	44.62	130	69	3	16	396	93	218	4.00E-30	106
TcMYB26	ATMYB119	39.1	133	80	2	7	402	91	221	5.00E-29	105
TcMYB26	ATMYB73	50	102	51	1	49	354	13	113	9.00E-29	102

TcMYB26	AtMYB64	39.84	128	76	3	49	429	105	224	2.00E-28	103
TcMYB26	AtMYB64	30.77	52	33	2	202	348	103	152	5.00E-04	32.7
TcMYB26	AtMYB109	45.99	137	66	4	37	423	52	186	2.00E-28	102
TcMYB26	ATMYB52	39.69	131	69	2	49	411	5	134	4.00E-28	99.8
TcMYB26	ATMYB44	46.36	110	55	2	49	366	6	110	4.00E-28	100
TcMYB26	AtMYB115	41.51	106	62	1	34	351	153	257	4.00E-28	101
TcMYB26	AtMYB1	35.88	170	99	2	49	528	55	223	4.00E-28	102
TcMYB26	ATMYB69	42.28	123	69	3	34	396	14	134	7.00E-28	99
TcMYB26	AtMYB70	47.62	105	55	1	49	363	13	116	1.00E-27	99.8
TcMYB26	AtMYB117	47.06	102	54	1	49	354	98	198	2.00E-27	99.8
TcMYB26	ATMYB54	47.92	96	50	1	49	336	6	100	3.00E-27	97.1
TcMYB26	ATMYB25	48.04	102	53	1	49	354	50	150	5.00E-27	99
TcMYB26	ATMYB77	36.05	147	94	1	49	489	6	151	2.00E-26	96.3
TcMYB26	ATMYB118	38.26	115	71	1	13	357	177	290	6.00E-26	96.7
TcMYB26	ATMYB110	41.74	115	67	2	10	354	59	167	3.00E-25	93.2
TcMYB26	AtMYB56	44	100	56	1	37	336	89	187	4.00E-25	93.2
TcMYB26	AtMYB98	40.19	107	64	1	34	354	212	317	1.00E-24	93.2
TcMYB26	AtMYB89	37.17	113	71	1	13	351	44	155	2.00E-22	83.2
TcMYB26	AtMYB100	38.1	105	62	2	58	363	29	132	3.00E-22	84
TcMYB26	ATMYB91	31.85	157	98	2	46	489	3	159	6.00E-21	82
TcMYB26	AtMYB22	33.04	112	72	2	34	360	49	158	7.00E-18	72.4
TcMYB26	ATMYB88	37.37	99	62	1	58	354	33	130	9.00E-18	73.6
TcMYB26	AtMYB124	36.36	99	63	1	58	354	28	125	2.00E-17	72.4
TcMYB27	AtMYB56	44.92	236	110	4	37	684	88	322	1.00E-58	181
TcMYB27	AtMYB117	70.18	114	33	1	49	387	97	210	1.00E-57	179
TcMYB27	ATMYB105	71.68	113	31	1	52	387	107	219	3.00E-57	178
TcMYB27	ATMYB54	60.16	128	44	1	43	405	3	130	4.00E-53	164
TcMYB27	ATMYB110	36.23	265	130	6	4	681	46	304	1.00E-52	165
TcMYB27	ATMYB69	40.51	237	116	5	46	681	17	247	2.00E-52	163
TcMYB27	ATMYB52	66.02	103	35	0	43	351	2	104	2.00E-51	160
TcMYB27	AtMYB89	61.62	99	38	0	55	351	57	155	3.00E-44	140
TcMYB27	ATMYB44	57.89	95	40	0	52	336	6	100	4.00E-39	130
TcMYB27	ATMYB73	55.79	95	42	0	52	336	13	107	2.00E-38	129
TcMYB27	AtMYB70	50.89	112	55	1	1	336	1	107	3.00E-37	125
TcMYB27	ATMYB77	38.1	168	94	2	52	525	6	173	8.00E-37	124
TcMYB27	AtMYB109	44.93	138	74	2	52	459	56	193	2.00E-36	125
TcMYB27	AtMYB1	54.74	95	43	0	52	336	55	149	9.00E-36	123
TcMYB27	ATMYB25	51.58	95	46	0	52	336	50	144	1.00E-33	117
TcMYB27	ATMYB119	39.16	143	87	2	4	432	92	220	5.00E-31	111
TcMYB27	ATMYB72	32.85	207	119	6	52	612	16	215	2.00E-30	107
TcMYB27	AtMYB64	40.16	127	76	1	52	432	105	220	2.00E-30	109
TcMYB27	ATMYB118	45.13	113	62	1	52	390	189	299	4.00E-30	108
TcMYB27	AtMYB115	33.52	176	101	2	31	510	151	326	6.00E-30	107
TcMYB27	ATMYB66	36.94	157	98	3	52	519	18	157	2.00E-29	102
TcMYB27	ATMYB63	34.97	183	94	4	52	525	16	197	2.00E-29	104
TcMYB27	ATMYB23	30.57	193	117	2	52	579	14	206	2.00E-29	102
TcMYB27	AtMYB82	43.55	124	69	2	52	420	14	126	3.00E-29	102
TcMYB27	AtMYB10	40.46	131	76	2	52	438	16	145	4.00E-29	102
TcMYB27	ATMYB35	48.96	96	48	1	52	336	14	109	4.00E-29	104
TcMYB27	AtMYB100	48.39	93	48	0	58	336	28	120	6.00E-29	102
TcMYB27	AtMYB36	39.33	150	89	3	52	495	14	162	7.00E-29	103
TcMYB27	AtMYB19	43.94	132	72	4	1	390	1	126	9.00E-29	102
TcMYB27	AtMYB45	36	175	111	5	52	573	20	170	2.00E-28	101
TcMYB27	ATMYB121	36.67	150	93	2	34	477	23	171	2.00E-28	101
TcMYB27	AtMYB27	45.37	108	57	2	52	369	11	117	3.00E-28	100
TcMYB27	ATMYB84	40.58	138	80	4	52	459	14	144	4.00E-28	101
TcMYB27	ATMYB15	35.17	145	93	1	52	483	14	158	5.00E-28	100
TcMYB27	AtMYB114	44.95	109	59	1	37	360	5	113	5.00E-28	97.1
TcMYB27	ATMYB80	47.92	96	49	1	52	336	14	109	9.00E-28	100
TcMYB27	ATMYB13	31	200	133	5	52	636	14	199	1.00E-27	99
TcMYB27	ATMYB68	40.58	138	80	4	52	459	14	144	1.00E-27	101
TcMYB27	ATMYB67	35.44	158	100	2	52	519	24	180	1.00E-27	100
TcMYB27	ATMYB0	45.54	101	54	1	52	351	16	116	2.00E-27	98.2
TcMYB27	ATMYB123	37.6	125	77	2	52	423	16	138	2.00E-27	98.6
TcMYB27	ATMYB46	39.72	141	83	3	34	450	14	152	2.00E-27	99
TcMYB27	AtMYB98	45.71	105	57	1	52	366	217	319	3.00E-27	100
TcMYB27	AtMYB18	45.83	96	51	1	52	336	12	107	3.00E-27	98.6
TcMYB27	ATMYB14	37.59	141	83	3	52	459	14	152	4.00E-27	97.8
TcMYB27	ATMYB58	36.6	153	94	3	52	501	16	168	4.00E-27	98.2
TcMYB27	ATMYB4	30.92	207	141	7	52	666	14	197	1.00E-26	97.1
TcMYB27	ATMYB122	34.55	165	105	2	52	537	14	178	3.00E-26	97.1
TcMYB27	ATMYB75	44.95	109	59	1	37	360	5	113	4.00E-26	95.1
TcMYB27	ATMYB37	32.23	211	124	6	52	627	14	223	4.00E-26	96.7
TcMYB27	ATMYB59-3	38.06	134	81	3	52	447	10	132	4.00E-26	94.7
TcMYB27	ATMYB48	34.81	158	101	3	52	519	9	154	4.00E-26	95.1
TcMYB27	AtMYB112	41.38	116	67	1	52	396	34	149	6.00E-26	94.4
TcMYB27	AtMYB41	46.39	97	50	2	52	336	14	109	7.00E-26	95.1
TcMYB27	ATMYB88	27.31	216	139	3	61	654	33	248	7.00E-26	97.4
TcMYB27	ATMYB88	33.9	59	39	1	52	228	82	139	2.00E-05	37
TcMYB27	AtMYB83	30.57	193	121	4	52	591	32	224	8.00E-26	95.9

TcMYB27	AtMYB124	31.29	163	103	3	61	522	28	187	1.00E-25	96.3
TcMYB27	AtMYB124	28.79	66	47	1	52	249	77	141	8.00E-05	35.4
TcMYB27	ATMYB71	32.58	178	118	4	52	579	20	192	1.00E-25	94
TcMYB27	AtMYB74	44.79	96	52	1	52	336	15	110	2.00E-25	94.7
TcMYB27	AtMYB104	39.62	106	63	1	22	336	8	113	2.00E-25	95.5
TcMYB27	AtMYB43	35.76	165	86	5	52	486	14	177	2.00E-25	94.7
TcMYB27	ATMYB87	36.05	147	92	4	52	486	14	155	2.00E-25	94.4
TcMYB27	ATMYB90	42.5	120	67	2	37	390	5	124	2.00E-25	93.2
TcMYB27	AtMYB53	40.74	135	78	3	52	450	14	142	2.00E-25	94.4
TcMYB27	ATMYB34	46.88	96	50	1	52	336	14	109	2.00E-25	94
TcMYB27	AtMYB51	45.83	96	51	1	52	336	15	110	3.00E-25	94.4
TcMYB27	ATMYB38	39.72	141	83	4	52	468	14	149	9.00E-25	92.4
TcMYB27	ATMYB92	47.42	97	49	2	52	336	14	109	1.00E-24	92.8
TcMYB27	ATMYB2	46.32	95	50	1	52	333	22	116	1.00E-24	91.7
TcMYB27	AtMYB79	36.23	138	86	3	52	459	8	143	1.00E-24	91.3
TcMYB27	AtMYB6	35.25	139	88	3	52	462	14	147	2.00E-24	90.1
TcMYB27	AtMYB49	44.79	96	52	1	52	336	14	109	2.00E-24	91.7
TcMYB27	AtMYB22	40.52	116	64	2	4	336	33	147	3.00E-24	90.1
TcMYB27	ATMYB3	44.33	97	52	2	52	336	14	109	3.00E-24	90.1
TcMYB27	AtMYB62	29.55	247	142	8	4	648	12	250	4.00E-24	90.5
TcMYB27	ATMYB61	33.95	162	91	4	52	489	14	174	4.00E-24	91.7
TcMYB27	AtMYB103	27.96	211	137	3	52	639	14	224	4.00E-24	91.7
TcMYB27	AtMYB50	37.86	140	74	4	52	432	14	149	4.00E-24	90.9
TcMYB27	AtMYB32	43.75	96	53	1	52	336	14	109	4.00E-24	90.1
TcMYB27	ATMYB55	30.85	188	100	5	52	525	14	196	4.00E-24	91.3
TcMYB27	ATMYB57	31.98	172	115	5	52	561	27	191	5.00E-24	88.6
TcMYB27	ATMYB94	30.81	198	131	4	52	627	14	196	5.00E-24	90.9
TcMYB27	AtMYB113	42.86	105	58	2	52	360	10	114	5.00E-24	89.4
TcMYB27	ATMYB26	32.94	170	93	4	52	498	14	180	5.00E-24	91.3
TcMYB27	ATMYB86	44.33	97	52	2	52	336	14	109	6.00E-24	90.9
TcMYB27	ATMYB5	42.71	96	54	1	52	336	25	120	8.00E-24	89
TcMYB27	AtMYB76	33.51	194	109	5	52	573	14	203	1.00E-23	90.1
TcMYB27	AtMYB107	43.75	96	53	1	52	336	14	109	1.00E-23	89.7
TcMYB27	AtMYB40	44.33	97	52	2	52	336	14	109	1.00E-23	88.6
TcMYB27	AtMYB9	43.75	96	53	1	52	336	14	109	2.00E-23	89.4
TcMYB27	MYB8	39.58	96	57	1	52	336	14	109	2.00E-23	87
TcMYB27	ATMYB95	40.62	96	56	1	52	336	14	109	2.00E-23	88.2
TcMYB27	ATMYB102	43.75	96	53	1	52	336	14	109	3.00E-23	89
TcMYB27	AtMYB108	40.17	117	68	2	52	396	21	136	3.00E-23	88.6
TcMYB27	MYB7	41.24	97	55	2	52	336	14	109	6.00E-23	87
TcMYB27	ATMYB106	30.16	189	112	5	52	558	57	245	6.00E-23	88.6
TcMYB27	ATMYB28	35.17	145	82	2	52	450	14	158	9.00E-23	87.8
TcMYB27	ATMYB16	43.3	97	53	2	52	336	14	109	1.00E-22	87
TcMYB27	AtMYB93	43.75	96	53	1	52	336	14	109	1.00E-22	87.4
TcMYB27	ATMYB65	41.67	96	55	1	52	336	43	138	2.00E-22	87.8
TcMYB27	AtMYB97	35.43	127	75	2	52	411	21	147	3.00E-22	86.7
TcMYB27	ATMYB30	34.78	138	89	2	52	462	14	140	3.00E-22	85.9
TcMYB27	AtMYB60	32.24	152	97	3	52	489	14	154	3.00E-22	85.1
TcMYB27	AtMYB47	37.5	96	59	1	52	336	14	109	4.00E-22	84.7
TcMYB27	ATMYB96	35.46	141	87	3	52	462	14	143	4.00E-22	85.9
TcMYB27	ATMYB29	44.79	96	52	1	52	336	14	109	5.00E-22	85.5
TcMYB27	AtMYB116	30.22	225	154	7	4	669	8	201	5.00E-22	84.7
TcMYB27	AtMYB85	32.07	184	121	4	52	591	14	194	5.00E-22	84.3
TcMYB27	ATMYB101	30.49	164	106	3	52	519	20	182	6.00E-22	86.3
TcMYB27	AtMYB24	30.94	181	106	5	52	537	19	199	1.00E-21	82.4
TcMYB27	AtMYB81	41.67	96	55	1	52	336	22	117	2.00E-21	84.7
TcMYB27	AtMYB42	43.3	97	53	2	52	336	14	109	3.00E-21	82.8
TcMYB27	ATMYB31	34.51	142	89	3	52	465	14	144	3.00E-21	83.2
TcMYB27	AtMYB20	29.28	222	127	5	52	627	14	234	5.00E-21	82
TcMYB27	ATMYB3	33.59	128	84	1	52	432	22	149	7.00E-21	80.5
TcMYB27	ATMYB33	40.62	96	56	1	52	336	34	129	7.00E-21	83.2
TcMYB27	AtMYB17	41.24	97	55	2	52	336	14	109	2.00E-20	80.5
TcMYB27	ATMYB111	38.54	96	58	1	52	336	14	109	2.00E-20	80.9
TcMYB27	ATMYB78	33.14	175	87	6	52	486	28	200	1.00E-19	78.6
TcMYB27	ATMYB120	26.67	180	115	2	52	540	28	207	2.00E-19	79.3
TcMYB27	ATMYB11	37.5	96	59	1	52	336	14	109	2.00E-19	78.2
TcMYB27	ATMYB12	37.5	96	59	1	52	336	14	109	3.00E-19	78.2
TcMYB27	ATMYB99	34.31	137	82	4	52	438	15	149	4.00E-19	76.3
TcMYB27	ATMYB91	28.75	160	109	3	52	516	4	154	6.00E-19	77
TcMYB28	ATMYB91	45.41	392	211	9	1	1167	1	358	3.00E-109	319
TcMYB28	ATMYB48	28.87	239	141	6	19	648	12	242	7.00E-29	106
TcMYB28	AtMYB82	41.23	114	67	1	7	348	13	124	1.00E-28	104
TcMYB28	AtMYB97	29.23	284	187	5	19	828	24	278	2.00E-28	108
TcMYB28	ATMYB120	31.09	238	145	4	19	675	31	260	2.00E-28	109
TcMYB28	ATMYB120	29.05	148	92	4	322	726	229	375	8.00E-06	40
TcMYB28	ATMYB59-3	42.24	116	66	2	19	363	13	126	3.00E-28	104
TcMYB28	ATMYB57	38.46	130	71	2	19	381	30	157	1.00E-27	102
TcMYB28	AtMYB79	36.17	141	90	2	19	441	11	145	5.00E-27	101
TcMYB28	AtMYB116	41.96	112	65	1	19	354	23	132	8.00E-27	101
TcMYB28	ATMYB96	28.24	255	175	6	19	759	17	264	1.00E-26	102

TcMYB28	ATMYB63	38.97	136	77	4	7	396	15	148	2.00E-26	100
TcMYB28	AtMYB76	36.25	160	102	2	19	498	17	172	3.00E-26	101
TcMYB28	ATMYB101	42.72	103	59	1	19	327	23	123	3.00E-26	102
TcMYB28	AtMYB19	39.52	124	72	2	4	366	9	130	4.00E-26	99.4
TcMYB28	AtMYB51	30.04	273	181	9	19	807	18	271	4.00E-26	100
TcMYB28	ATMYB30	27.24	257	181	4	19	771	17	247	5.00E-26	100
TcMYB28	ATMYB80	40.95	105	62	1	7	321	13	115	7.00E-26	99.8
TcMYB28	ATMYB121	37.72	114	71	1	19	360	32	143	8.00E-26	98.6
TcMYB28	ATMYB66	37.19	121	76	1	19	381	21	139	9.00E-26	96.7
TcMYB28	AtMYB45	38.58	127	74	3	4	372	15	139	1.00E-25	97.8
TcMYB28	AtMYB60	38.66	119	73	1	19	375	17	133	1.00E-25	98.2
TcMYB28	ATMYB75	35.33	167	101	5	19	498	13	171	2.00E-25	97.1
TcMYB28	AtMYB62	36.05	147	89	3	10	435	21	156	2.00E-25	97.8
TcMYB28	ATMYB71	40.57	106	63	1	19	336	23	126	2.00E-25	97.4
TcMYB28	ATMYB37	37.97	158	89	3	7	453	13	169	2.00E-25	98.6
TcMYB28	AtMYB81	30.85	201	120	4	19	564	25	223	2.00E-25	99.8
TcMYB28	AtMYB50	32.38	210	114	6	19	564	17	224	3.00E-25	97.8
TcMYB28	AtMYB24	35.07	134	85	2	19	414	22	153	4.00E-25	95.1
TcMYB28	ATMYB90	39.83	118	71	1	19	372	13	128	5.00E-25	95.9
TcMYB28	ATMYB0	32.9	155	94	2	19	453	19	171	6.00E-25	95.1
TcMYB28	ATMYB65	41.75	103	60	1	19	327	46	146	6.00E-25	99.4
TcMYB28	AtMYB27	41.51	106	62	1	10	327	11	114	7.00E-25	95.1
TcMYB28	ATMYB33	41.75	103	60	1	19	327	37	137	8.00E-25	99
TcMYB28	AtMYB114	39.67	121	73	2	19	381	13	130	8.00E-25	92
TcMYB28	AtMYB112	39.62	106	64	1	10	327	34	137	1.00E-24	94.7
TcMYB28	ATMYB84	33.33	165	105	4	19	498	17	178	1.00E-24	95.9
TcMYB28	AtMYB36	34.09	176	115	4	19	543	17	178	1.00E-24	96.3
TcMYB28	ATMYB31	31.67	240	151	10	19	699	17	239	2.00E-24	95.9
TcMYB28	ATMYB3	38.53	109	67	1	19	345	25	131	2.00E-24	93.6
TcMYB28	ATMYB14	34.69	147	90	3	7	429	13	155	2.00E-24	94
TcMYB28	ATMYB94	27.49	251	153	6	19	684	17	264	3.00E-24	95.5
TcMYB28	AtMYB74	32.04	181	107	3	19	513	18	196	3.00E-24	95.1
TcMYB28	ATMYB102	33.14	175	82	3	19	438	17	189	3.00E-24	95.5
TcMYB28	AtMYB17	44.55	101	56	1	19	321	17	115	4.00E-24	94.4
TcMYB28	ATMYB3	34.85	132	83	2	19	405	17	146	5.00E-24	93.2
TcMYB28	ATMYB123	38.02	121	72	2	10	363	16	134	5.00E-24	93.2
TcMYB28	AtMYB103	37.7	122	76	1	7	372	13	132	5.00E-24	95.1
TcMYB28	ATMYB2	38.83	103	63	1	19	327	25	125	7.00E-24	93.2
TcMYB28	ATMYB119	37.07	116	73	1	16	363	107	219	7.00E-24	95.5
TcMYB28	ATMYB119	29.49	78	51	2	175	396	105	182	6.00E-04	33.9
TcMYB28	ATMYB61	28.47	281	191	8	19	831	17	284	7.00E-24	94.7
TcMYB28	ATMYB86	27.65	217	155	3	19	663	17	227	8.00E-24	94.4
TcMYB28	AtMYB53	41.58	101	59	1	19	321	17	115	9.00E-24	93.6
TcMYB28	AtMYB113	39.83	118	71	1	19	372	13	128	1.00E-23	92
TcMYB28	ATMYB67	28.57	203	133	5	7	579	23	207	1.00E-23	93.2
TcMYB28	ATMYB92	26.58	237	173	3	19	726	17	247	1.00E-23	93.6
TcMYB28	AtMYB98	42.86	105	60	1	16	330	219	320	1.00E-23	94.7
TcMYB28	AtMYB98	32.38	105	70	2	25	336	169	271	9.00E-10	52.4
TcMYB28	ATMYB28	34.16	161	101	3	19	486	17	168	1.00E-23	94
TcMYB28	ATMYB55	38.76	129	69	2	19	375	17	143	1.00E-23	93.6
TcMYB28	AtMYB18	35.77	137	77	3	7	384	8	142	1.00E-23	92.4
TcMYB28	ATMYB5	33.58	137	81	3	7	387	24	158	1.00E-23	91.7
TcMYB28	ATMYB26	28.17	252	169	7	7	726	13	243	2.00E-23	93.6
TcMYB28	ATMYB38	28.31	219	148	3	7	636	13	230	2.00E-23	92.4
TcMYB28	AtMYB41	39.66	116	70	1	19	366	17	130	2.00E-23	92
TcMYB28	AtMYB104	39.25	107	65	1	10	330	18	122	2.00E-23	93.6
TcMYB28	ATMYB23	38.61	101	62	1	19	321	17	115	2.00E-23	90.5
TcMYB28	AtMYB107	39.6	101	61	1	19	321	17	115	3.00E-23	92.4
TcMYB28	ATMYB46	41.03	117	67	2	19	363	23	137	3.00E-23	91.7
TcMYB28	AtMYB108	38.89	108	66	1	7	330	20	125	3.00E-23	92.4
TcMYB28	ATMYB122	38.4	125	77	1	19	393	17	139	3.00E-23	92.4
TcMYB28	ATMYB15	40	105	63	1	7	321	13	115	4.00E-23	91.3
TcMYB28	AtMYB64	40	105	63	1	16	330	107	208	5.00E-23	92.8
TcMYB28	AtMYB64	28.57	70	49	1	130	336	90	159	2.00E-04	35.4
TcMYB28	AtMYB9	39.6	101	61	1	19	321	17	115	6.00E-23	91.7
TcMYB28	MYB8	31.45	124	85	1	19	390	17	138	6.00E-23	89
TcMYB28	AtMYB49	40.59	101	60	1	19	321	17	115	9.00E-23	90.9
TcMYB28	AtMYB10	38.89	108	66	1	7	330	15	120	1.00E-22	89
TcMYB28	ATMYB16	37.5	112	70	1	19	354	17	126	2.00E-22	90.1
TcMYB28	ATMYB29	35.15	165	107	4	19	513	17	161	2.00E-22	90.1
TcMYB28	ATMYB34	40.18	112	67	1	19	354	17	126	2.00E-22	89.4
TcMYB28	ATMYB106	37.5	112	70	1	19	354	60	169	2.00E-22	90.5
TcMYB28	AtMYB93	40.59	101	60	1	19	321	17	115	3.00E-22	90.1
TcMYB28	ATMYB58	37.27	110	69	1	7	336	15	122	3.00E-22	88.6
TcMYB28	ATMYB95	37.29	118	74	1	19	372	17	132	5.00E-22	87.8
TcMYB28	ATMYB4	42.27	97	56	1	19	309	17	111	6.00E-22	87.8
TcMYB28	ATMYB118	37.04	108	68	1	16	339	191	295	6.00E-22	89.7
TcMYB28	AtMYB42	38.98	118	72	1	1	354	11	126	1.00E-21	87
TcMYB28	ATMYB12	37.14	105	66	1	7	321	13	115	1.00E-21	88.2
TcMYB28	AtMYB32	36.61	112	71	1	19	354	17	126	1.00E-21	86.7

TcMYB28	AtMYB85	40.18	112	67	1	19	354	17	126	2.00E-21	86.3
TcMYB28	MYB7	36.61	112	71	1	19	354	17	126	2.00E-21	86.3
TcMYB28	ATMYB72	33.61	119	79	1	7	363	15	131	2.00E-21	86.7
TcMYB28	ATMYB68	38.79	116	66	3	19	351	17	130	3.00E-21	87
TcMYB28	AtMYB20	36.97	119	75	1	19	375	17	133	4.00E-21	85.5
TcMYB28	ATMYB87	38.24	102	62	2	19	321	17	116	7.00E-21	85.1
TcMYB28	ATMYB105	30	180	120	4	10	531	107	263	7.00E-21	85.5
TcMYB28	AtMYB56	38.32	107	66	1	10	330	93	196	9.00E-21	85.1
TcMYB28	AtMYB47	36.44	118	75	2	1	354	12	126	1.00E-20	84
TcMYB28	AtMYB83	35.65	115	74	1	19	363	35	147	1.00E-20	85.1
TcMYB28	AtMYB6	33.93	112	74	1	19	354	17	126	1.00E-20	83.2
TcMYB28	AtMYB117	28.33	180	129	2	10	549	98	272	1.00E-20	85.1
TcMYB28	ATMYB35	36.36	121	75	2	19	375	17	135	2.00E-20	84.3
TcMYB28	ATMYB13	34.29	140	92	3	19	438	17	143	2.00E-20	82.8
TcMYB28	AtMYB115	38.61	101	62	1	16	318	160	257	2.00E-20	84.3
TcMYB28	ATMYB25	42.42	99	57	1	4	300	48	143	3.00E-20	84
TcMYB28	ATMYB52	33.62	116	77	1	1	348	2	114	4.00E-20	82
TcMYB28	ATMYB69	33.61	119	79	1	7	363	18	133	4.00E-20	82
TcMYB28	AtMYB43	39.29	112	68	1	19	354	17	126	6.00E-20	82.8
TcMYB28	ATMYB111	33.33	105	70	1	7	321	13	115	7.00E-20	82.8
TcMYB28	ATMYB11	34.29	105	69	1	7	321	13	115	9.00E-20	82.4
TcMYB28	ATMYB73	40.43	94	56	1	19	300	16	106	1.00E-19	82
TcMYB28	ATMYB78	33.88	121	66	2	10	330	28	146	1.00E-19	82
TcMYB28	ATMYB110	33.33	129	86	1	10	396	67	192	2.00E-19	80.9
TcMYB28	ATMYB54	33.63	113	75	1	1	339	3	112	3.00E-19	79.3
TcMYB28	AtMYB70	39.81	103	58	2	4	300	7	106	3.00E-19	80.5
TcMYB28	AtMYB109	32.84	134	90	1	4	405	54	184	5.00E-19	80.9
TcMYB28	ATMYB99	37.74	106	61	1	19	321	18	123	5.00E-19	78.6
TcMYB28	ATMYB77	22.65	287	218	4	19	867	9	265	2.00E-18	78.2
TcMYB28	ATMYB44	27.35	234	164	5	19	702	9	221	2.00E-18	77.8
TcMYB28	AtMYB1	39.36	94	57	1	19	300	58	148	3.00E-18	78.2
TcMYB28	AtMYB40	33.62	116	77	1	7	354	13	126	8.00E-18	75.5
TcMYB28	AtMYB100	27.52	149	108	2	7	453	25	167	2.00E-17	73.9
TcMYB28	ATMYB88	29.77	131	88	2	4	384	24	151	3.00E-16	72.8
TcMYB28	AtMYB22	30	120	84	2	10	369	54	169	9.00E-16	69.3
TcMYB28	AtMYB124	28.12	128	86	3	4	369	19	143	1.00E-14	67.8
TcMYB28	AtMYB89	32.98	94	63	1	19	300	59	149	1.00E-13	62
TcMYB29	AtMYB124	34.43	517	332	14	58	1587	19	436	6.00E-75	237
TcMYB29	ATMYB88	32.31	523	346	12	43	1587	19	484	4.00E-73	233
TcMYB29	ATMYB118	42.24	116	67	0	85	432	192	307	5.00E-33	123
TcMYB29	ATMYB118	27.5	120	85	3	88	441	144	259	2.00E-07	45.4
TcMYB29	ATMYB119	38.76	129	73	1	85	453	108	236	7.00E-30	114
TcMYB29	AtMYB64	47.06	102	54	0	85	390	108	209	1.00E-29	113
TcMYB29	AtMYB64	29.73	74	51	1	232	450	105	178	7.00E-05	37.4
TcMYB29	AtMYB100	32.47	194	117	4	58	597	20	213	8.00E-28	104
TcMYB29	AtMYB115	42.99	107	61	0	85	405	161	267	2.00E-27	105
TcMYB29	AtMYB115	29.01	131	92	2	232	621	158	281	1.00E-08	48.9
TcMYB29	AtMYB98	45.1	102	56	0	85	390	220	321	3.00E-26	103
TcMYB29	AtMYB98	28.57	84	59	1	202	450	207	290	4.00E-08	47.8
TcMYB29	AtMYB98	34.38	64	42	2	43	234	262	320	4.00E-05	38.1
TcMYB29	AtMYB70	38.89	126	76	1	58	432	7	132	6.00E-26	100
TcMYB29	AtMYB79	38.46	130	78	3	85	468	11	137	1.00E-25	99
TcMYB29	ATMYB66	46.85	111	53	3	85	399	21	131	9.00E-25	94.7
TcMYB29	ATMYB121	35.38	130	83	1	85	471	32	161	2.00E-24	95.9
TcMYB29	ATMYB73	41.12	107	62	1	58	375	7	113	3.00E-24	96.3
TcMYB29	ATMYB77	44.23	104	57	1	67	375	3	106	1.00E-23	94
TcMYB29	ATMYB0	43.97	116	58	3	85	411	19	134	1.00E-23	92
TcMYB29	ATMYB71	35.66	129	82	2	85	468	23	149	2.00E-23	92.4
TcMYB29	ATMYB59-3	41.75	103	59	1	85	390	13	115	3.00E-23	91.3
TcMYB29	ATMYB48	42.72	103	58	1	85	390	12	114	3.00E-23	91.7
TcMYB29	ATMYB110	41.9	105	60	1	85	396	70	174	6.00E-23	92
TcMYB29	ATMYB2	31.84	179	119	4	7	534	4	175	6.00E-23	91.3
TcMYB29	ATMYB23	46	100	52	2	85	378	17	116	1.00E-22	89.4
TcMYB29	ATMYB44	44.23	104	57	1	67	375	3	106	1.00E-22	91.3
TcMYB29	ATMYB3	34.31	137	89	3	85	492	25	157	2.00E-22	88.6
TcMYB29	ATMYB35	44.12	102	56	2	85	387	17	115	2.00E-22	90.5
TcMYB29	ATMYB35	33.33	66	44	2	52	249	63	123	2.00E-04	35.4
TcMYB29	AtMYB116	36.04	111	70	1	85	414	23	133	2.00E-22	89.7
TcMYB29	ATMYB105	36.19	105	67	0	85	399	110	214	3.00E-22	90.5
TcMYB29	AtMYB1	39.47	114	67	1	64	399	51	164	3.00E-22	91.3
TcMYB29	ATMYB123	33.8	142	86	3	85	486	19	160	4.00E-22	88.6
TcMYB29	AtMYB89	32.58	132	89	1	7	402	34	164	6.00E-22	86.3
TcMYB29	ATMYB46	34.87	152	94	3	85	525	23	174	8.00E-22	88.2
TcMYB29	AtMYB82	40.87	115	67	2	85	426	17	129	8.00E-22	86.3
TcMYB29	AtMYB109	42.27	97	56	0	85	375	59	155	1.00E-21	89.7
TcMYB29	AtMYB109	39.06	64	37	2	232	417	56	119	3.00E-06	41.6
TcMYB29	AtMYB109	33.33	48	32	1	85	228	111	157	3.00E-04	35.4
TcMYB29	AtMYB117	37.5	104	65	0	85	396	101	204	1.00E-21	89
TcMYB29	AtMYB117	32.81	64	41	2	232	417	98	161	3.00E-05	38.5
TcMYB29	AtMYB27	39.05	105	62	2	85	393	14	117	1.00E-21	86.7

TcMYB29	AtMYB62	36.04	111	70	1	85	414	24	134	2.00E-21	87.4
TcMYB29	ATMYB69	36.61	112	71	0	85	420	22	133	2.00E-21	86.3
TcMYB29	AtMYB22	31.34	134	92	2	4	405	34	162	2.00E-21	86.3
TcMYB29	AtMYB22	31.51	73	50	0	232	450	54	126	9.00E-07	42.7
TcMYB29	AtMYB112	33.82	136	89	2	10	414	22	147	3.00E-21	85.5
TcMYB29	AtMYB24	44.44	90	49	1	85	351	22	111	5.00E-21	84.3
TcMYB29	ATMYB57	36.97	119	74	1	85	438	30	148	6.00E-21	84
TcMYB29	ATMYB61	39.81	108	64	1	85	405	17	124	8.00E-21	86.7
TcMYB29	ATMYB14	32.17	143	88	3	85	486	17	157	1.00E-20	84.3
TcMYB29	ATMYB13	32.09	134	88	3	85	477	17	149	1.00E-20	84
TcMYB29	ATMYB52	37.86	103	64	0	85	393	8	110	2.00E-20	83.6
TcMYB29	ATMYB52	31.25	64	42	2	232	417	5	68	3.00E-05	38.1
TcMYB29	ATMYB67	25.84	209	147	4	85	687	27	234	2.00E-20	84.3
TcMYB29	AtMYB83	41.28	109	60	2	85	399	35	140	3.00E-20	84.7
TcMYB29	ATMYB65	34.78	138	79	4	4	384	10	146	5.00E-20	85.5
TcMYB29	ATMYB54	36.89	103	65	0	85	393	9	111	5.00E-20	82
TcMYB29	MYB7	25.78	256	177	8	85	813	17	248	8.00E-20	82
TcMYB29	AtMYB50	34.97	143	88	2	85	498	17	159	9.00E-20	82.8
TcMYB29	AtMYB103	37.72	114	70	1	85	423	17	130	9.00E-20	83.6
TcMYB29	ATMYB87	33.85	130	84	2	85	468	17	146	1.00E-19	82.4
TcMYB29	ATMYB3	34.13	126	81	2	85	456	17	142	1.00E-19	81.3
TcMYB29	ATMYB86	40.78	103	59	2	85	387	17	119	1.00E-19	82.8
TcMYB29	ATMYB80	41.35	104	60	2	85	393	17	117	2.00E-19	82
TcMYB29	ATMYB80	31.17	77	51	3	85	309	70	136	4.00E-04	34.7
TcMYB29	AtMYB108	35.14	111	71	1	85	414	24	134	2.00E-19	82
TcMYB29	ATMYB33	38.61	101	61	1	85	384	37	137	2.00E-19	83.6
TcMYB29	AtMYB10	32.17	143	94	4	85	504	19	158	2.00E-19	80.1
TcMYB29	ATMYB5	32.88	146	86	3	85	486	28	173	3.00E-19	80.1
TcMYB29	ATMYB34	39.81	103	61	1	85	390	17	119	3.00E-19	80.9
TcMYB29	ATMYB34	31.48	54	35	2	85	240	70	120	5.00E-04	34.3
TcMYB29	AtMYB32	34.68	124	66	2	85	411	17	140	3.00E-19	80.5
TcMYB29	ATMYB101	34.65	101	65	1	85	384	23	123	4.00E-19	82.4
TcMYB29	ATMYB25	38.78	98	59	1	85	375	53	150	5.00E-19	81.3
TcMYB29	ATMYB58	37.96	108	66	2	85	405	19	123	5.00E-19	79.7
TcMYB29	AtMYB9	36.73	98	61	1	85	375	17	114	7.00E-19	80.5
TcMYB29	AtMYB41	36.73	98	61	1	85	375	17	114	8.00E-19	79.3
TcMYB29	AtMYB41	29.41	68	46	3	52	249	63	123	3.00E-04	35
TcMYB29	ATMYB15	32.84	134	89	2	85	483	17	147	9.00E-19	79.3
TcMYB29	AtMYB56	37.23	94	59	0	85	366	96	189	1.00E-18	79.7
TcMYB29	AtMYB56	27.71	83	57	3	169	408	71	153	6.00E-04	34.3
TcMYB29	ATMYB26	39.25	107	55	1	85	375	17	123	1.00E-18	80.1
TcMYB29	AtMYB47	33.58	137	87	3	85	483	17	153	1.00E-18	78.6
TcMYB29	ATMYB94	32.19	146	95	3	85	510	17	161	2.00E-18	79.3
TcMYB29	ATMYB12	41	100	57	2	85	378	17	116	2.00E-18	79.7
TcMYB29	AtMYB6	35.96	114	71	2	85	420	17	130	2.00E-18	77.4
TcMYB29	AtMYB45	33.09	136	89	3	85	486	23	156	2.00E-18	77.8
TcMYB29	ATMYB63	31.48	162	108	4	85	561	19	179	2.00E-18	78.2
TcMYB29	AtMYB107	36.73	98	61	1	85	375	17	114	2.00E-18	78.6
TcMYB29	AtMYB36	34.31	137	86	3	85	483	17	153	3.00E-18	78.6
TcMYB29	ATMYB92	38	100	60	2	85	378	17	116	3.00E-18	78.6
TcMYB29	AtMYB74	37.76	98	60	1	85	375	18	115	3.00E-18	78.2
TcMYB29	AtMYB74	31.82	66	45	2	52	249	64	124	4.00E-04	34.7
TcMYB29	AtMYB60	31.82	132	89	3	85	477	17	142	4.00E-18	77.4
TcMYB29	ATMYB4	41.18	102	56	3	85	378	17	116	4.00E-18	77.4
TcMYB29	ATMYB4	24.21	95	70	3	85	363	70	157	7.00E-05	37
TcMYB29	ATMYB95	36.89	103	63	2	85	387	17	119	4.00E-18	77
TcMYB29	MYB8	30.67	150	87	3	85	483	17	166	5.00E-18	75.5
TcMYB29	AtMYB93	39	100	59	2	85	378	17	116	7.00E-18	77.8
TcMYB29	AtMYB114	34.21	114	74	2	85	423	13	125	7.00E-18	73.2
TcMYB29	ATMYB84	31.08	148	100	2	85	522	17	164	7.00E-18	77
TcMYB29	AtMYB19	38	100	59	2	85	375	17	114	7.00E-18	76.3
TcMYB29	AtMYB97	32.35	102	68	1	85	387	24	125	8.00E-18	77.8
TcMYB29	AtMYB51	32.09	134	89	2	85	480	18	151	8.00E-18	77.4
TcMYB29	AtMYB18	34.51	113	73	1	85	420	15	127	9.00E-18	76.3
TcMYB29	AtMYB40	37.86	103	62	3	85	387	17	115	9.00E-18	75.9
TcMYB29	AtMYB53	39	100	59	2	85	378	17	116	9.00E-18	76.6
TcMYB29	AtMYB53	33.33	54	36	1	88	249	72	123	3.00E-04	35
TcMYB29	AtMYB81	33.66	101	66	1	85	384	25	125	1.00E-17	77.8
TcMYB29	ATMYB72	37.04	108	65	3	85	399	19	125	1.00E-17	76.3
TcMYB29	ATMYB11	38	100	60	2	85	378	17	116	1.00E-17	76.6
TcMYB29	ATMYB102	36.73	98	61	1	85	375	17	114	3.00E-17	75.9
TcMYB29	AtMYB17	37.37	99	60	2	85	375	17	114	3.00E-17	75.1
TcMYB29	ATMYB37	39.09	110	65	3	85	408	17	123	3.00E-17	75.5
TcMYB29	ATMYB78	33.6	125	68	2	85	414	31	155	3.00E-17	75.1
TcMYB29	ATMYB122	36.89	103	64	1	85	390	17	119	4.00E-17	75.1
TcMYB29	ATMYB122	35.85	53	34	2	91	249	73	123	3.00E-05	38.5
TcMYB29	ATMYB68	36.94	111	68	3	85	411	17	124	4.00E-17	75.5
TcMYB29	ATMYB111	37	100	61	2	85	378	17	116	4.00E-17	75.1
TcMYB29	ATMYB55	34.55	110	59	1	85	375	17	126	6.00E-17	74.7
TcMYB29	ATMYB55	31.75	63	43	1	85	273	82	143	1.00E-04	36.6

TcMYB29	AtMYB49	35.71	98	62	1	85	375	17	114	6.00E-17	74.3
TcMYB29	ATMYB96	36.75	117	73	3	85	432	17	131	6.00E-17	74.7
TcMYB29	AtMYB113	32.59	135	88	3	82	477	12	138	1.00E-16	72.4
TcMYB29	ATMYB75	38.71	93	56	1	85	360	13	105	1.00E-16	72.4
TcMYB29	ATMYB75	37.5	48	30	2	88	231	68	113	8.00E-04	33.5
TcMYB29	ATMYB38	37.86	103	62	3	85	387	17	116	1.00E-16	73.2
TcMYB29	AtMYB104	34.31	102	66	1	85	387	21	122	1.00E-16	73.9
TcMYB29	ATMYB91	31.71	123	80	2	85	441	7	129	2.00E-16	73.6
TcMYB29	ATMYB29	27.52	218	146	3	85	702	17	231	2.00E-16	73.2
TcMYB29	ATMYB120	33.33	99	65	1	85	378	31	129	2.00E-16	74.3
TcMYB29	ATMYB28	37.76	98	60	1	85	375	17	114	3.00E-16	72.8
TcMYB29	ATMYB28	31.58	57	37	2	85	249	70	123	4.00E-04	34.7
TcMYB29	AtMYB76	38.78	98	59	1	85	375	17	114	3.00E-16	72.4
TcMYB29	AtMYB20	36.89	103	62	3	85	384	17	118	3.00E-16	71.6
TcMYB29	AtMYB20	24.41	127	86	5	142	492	88	201	2.00E-04	35.8
TcMYB29	ATMYB90	37.74	106	65	2	85	399	13	115	4.00E-16	70.9
TcMYB29	ATMYB16	32.31	130	79	4	85	447	17	145	4.00E-16	72
TcMYB29	AtMYB42	33.9	118	77	2	85	435	17	131	6.00E-16	70.9
TcMYB29	ATMYB31	34.17	120	75	3	85	432	17	136	7.00E-16	71.2
TcMYB29	AtMYB85	27.75	173	117	4	85	579	17	182	9.00E-16	70.1
TcMYB29	ATMYB106	34.69	98	63	1	85	375	60	157	1.00E-15	71.2
TcMYB29	ATMYB30	30.09	113	78	2	85	420	17	128	2.00E-15	69.7
TcMYB29	ATMYB99	33.04	115	58	2	175	462	56	170	5.00E-15	67.4
TcMYB29	AtMYB43	36.89	103	62	3	85	384	17	118	3.00E-14	66.2
TcMYB29	AtMYB43	28.57	91	65	2	142	414	88	163	2.00E-04	35.4
TcMYB30	ATMYB73	50.22	229	113	3	22	705	7	213	2.00E-71	216
TcMYB30	AtMYB70	48.55	241	116	5	22	720	7	233	1.00E-68	209
TcMYB30	ATMYB77	47.11	225	116	3	31	696	3	218	1.00E-65	201
TcMYB30	ATMYB44	46.61	236	119	3	31	717	3	214	8.00E-64	196
TcMYB30	AtMYB109	68.93	103	32	0	34	342	54	156	2.00E-51	167
TcMYB30	AtMYB109	21.33	225	175	10	202	870	58	260	3.00E-04	34.3
TcMYB30	AtMYB1	68.27	104	33	0	31	342	52	155	6.00E-49	160
TcMYB30	ATMYB25	60.55	109	43	0	16	342	42	150	3.00E-46	152
TcMYB30	ATMYB54	55.45	101	45	0	40	342	6	106	1.00E-41	137
TcMYB30	ATMYB52	52.48	101	48	0	40	342	5	105	6.00E-40	133
TcMYB30	AtMYB117	52.83	106	50	0	40	357	98	203	1.00E-39	135
TcMYB30	AtMYB56	56.44	101	44	0	40	342	93	193	3.00E-39	133
TcMYB30	ATMYB105	54.46	101	46	0	40	342	107	207	6.00E-39	132
TcMYB30	ATMYB69	52.48	101	48	0	40	342	19	119	1.00E-38	129
TcMYB30	ATMYB110	47.32	112	57	1	13	342	56	167	3.00E-35	122
TcMYB30	AtMYB64	39.33	150	80	2	37	453	104	253	3.00E-33	119
TcMYB30	ATMYB119	40.97	144	81	2	7	426	94	237	4.00E-33	119
TcMYB30	AtMYB98	46.96	115	61	0	4	348	205	319	6.00E-33	118
TcMYB30	AtMYB47	39.63	164	90	3	40	504	14	177	7.00E-33	115
TcMYB30	ATMYB118	49.11	112	57	0	13	348	180	291	1.00E-32	117
TcMYB30	ATMYB34	52.94	102	47	1	40	342	14	115	2.00E-32	114
TcMYB30	AtMYB49	48.7	115	58	1	1	342	1	115	2.00E-32	115
TcMYB30	ATMYB121	52.48	101	47	1	40	339	29	129	2.00E-32	114
TcMYB30	ATMYB96	33.61	241	150	8	22	714	8	228	4.00E-32	115
TcMYB30	AtMYB51	34.86	218	128	4	40	651	15	232	5.00E-32	114
TcMYB30	ATMYB92	50.98	102	49	1	40	342	14	115	5.00E-32	114
TcMYB30	ATMYB80	50	104	51	1	40	348	14	117	8.00E-32	113
TcMYB30	AtMYB97	52.48	101	47	1	40	339	21	121	9.00E-32	114
TcMYB30	ATMYB46	41.38	145	84	2	40	471	20	158	1.00E-31	112
TcMYB30	AtMYB41	51.96	102	48	1	40	342	14	115	1.00E-31	112
TcMYB30	ATMYB31	31.12	241	155	6	22	711	8	239	1.00E-31	113
TcMYB30	ATMYB23	46.96	115	60	1	1	342	1	115	1.00E-31	110
TcMYB30	ATMYB95	51.96	102	48	1	40	342	14	115	2.00E-31	111
TcMYB30	AtMYB89	45.45	99	54	0	43	339	57	155	2.00E-31	109
TcMYB30	AtMYB60	43.41	129	68	3	40	411	14	126	2.00E-31	111
TcMYB30	ATMYB94	39.47	152	82	5	40	465	14	159	3.00E-31	112
TcMYB30	ATMYB14	50.96	104	50	1	40	348	14	117	4.00E-31	110
TcMYB30	ATMYB35	51.96	102	48	1	40	342	14	115	4.00E-31	111
TcMYB30	ATMYB63	48.18	110	56	1	40	366	16	125	5.00E-31	110
TcMYB30	ATMYB63	25.97	77	57	1	31	261	66	141	4.00E-04	33.5
TcMYB30	ATMYB30	35.18	199	105	6	40	564	14	201	6.00E-31	111
TcMYB30	AtMYB107	50	102	50	1	40	342	14	115	1.00E-30	110
TcMYB30	ATMYB106	51.96	102	48	1	40	342	57	158	1.00E-30	111
TcMYB30	AtMYB115	41.86	129	75	1	40	426	158	279	2.00E-30	110
TcMYB30	AtMYB82	49.02	102	51	1	40	342	14	115	2.00E-30	107
TcMYB30	ATMYB66	50	102	50	1	40	342	18	119	2.00E-30	107
TcMYB30	ATMYB13	44.19	129	70	3	22	402	8	127	2.00E-30	108
TcMYB30	ATMYB26	47.83	115	50	2	40	354	14	128	2.00E-30	110
TcMYB30	AtMYB74	50.98	102	49	1	40	342	15	116	3.00E-30	109
TcMYB30	ATMYB48	46.3	108	57	2	22	342	4	110	3.00E-30	107
TcMYB30	ATMYB16	50.98	102	49	1	40	342	14	115	3.00E-30	109
TcMYB30	ATMYB101	50.98	102	49	1	40	342	20	121	3.00E-30	111
TcMYB30	AtMYB53	45.22	115	62	1	1	342	1	115	3.00E-30	108
TcMYB30	AtMYB79	49.07	108	54	2	22	342	3	109	4.00E-30	107
TcMYB30	AtMYB45	38.41	164	100	3	40	528	20	172	4.00E-30	107

TcMYB30	ATMYB72	44.19	129	68	4	40	414	16	143	4.00E-30	108
TcMYB30	ATMYB59-3	45.1	102	55	1	40	342	10	111	4.00E-30	107
TcMYB30	AtMYB17	48.54	103	51	2	40	342	14	115	5.00E-30	108
TcMYB30	AtMYB9	50.98	102	49	1	40	342	14	115	7.00E-30	108
TcMYB30	ATMYB122	49.5	101	50	1	40	339	14	114	1.00E-29	108
TcMYB30	AtMYB6	48.21	112	57	1	22	354	8	119	1.00E-29	105
TcMYB30	AtMYB62	33.7	181	119	4	7	546	10	171	1.00E-29	107
TcMYB30	ATMYB4	49.07	108	54	1	22	342	8	115	2.00E-29	106
TcMYB30	AtMYB93	48.04	102	52	1	40	342	14	115	2.00E-29	107
TcMYB30	MYB8	45.37	108	58	1	22	342	8	115	3.00E-29	103
TcMYB30	ATMYB3	47.22	108	56	1	22	342	8	115	3.00E-29	105
TcMYB30	ATMYB0	44.17	120	65	2	40	393	16	135	4.00E-29	104
TcMYB30	AtMYB83	50.47	107	52	1	40	357	32	138	4.00E-29	106
TcMYB30	ATMYB71	44.55	110	60	1	40	366	20	129	4.00E-29	105
TcMYB30	ATMYB102	50	102	50	1	40	342	14	115	5.00E-29	106
TcMYB30	ATMYB15	31.58	209	137	3	22	630	8	199	6.00E-29	105
TcMYB30	AtMYB32	41.72	151	87	2	40	489	14	155	7.00E-29	104
TcMYB30	AtMYB116	43.52	108	60	1	22	342	14	121	8.00E-29	104
TcMYB30	ATMYB67	49.02	102	51	1	40	342	24	125	9.00E-29	105
TcMYB30	ATMYB37	49.51	103	50	2	40	342	14	116	9.00E-29	105
TcMYB30	AtMYB36	47.57	103	52	2	40	342	14	116	1.00E-28	105
TcMYB30	ATMYB65	47.06	102	53	1	40	342	43	144	1.00E-28	107
TcMYB30	ATMYB38	49.51	103	50	2	40	342	14	116	1.00E-28	104
TcMYB30	AtMYB18	46.09	115	61	2	1	342	1	113	2.00E-28	103
TcMYB30	ATMYB87	45.95	111	58	2	40	366	14	124	2.00E-28	104
TcMYB30	ATMYB123	48.15	108	55	2	22	342	12	117	2.00E-28	103
TcMYB30	ATMYB61	35.09	171	110	2	40	549	14	179	2.00E-28	105
TcMYB30	MYB7	49.02	102	51	1	40	342	14	115	2.00E-28	103
TcMYB30	AtMYB103	50	102	50	1	40	342	14	115	2.00E-28	105
TcMYB30	ATMYB120	49.5	101	50	1	40	339	28	128	2.00E-28	106
TcMYB30	ATMYB2	44.35	115	63	2	22	363	16	129	2.00E-28	103
TcMYB30	ATMYB58	33.5	206	133	4	40	645	16	189	5.00E-28	102
TcMYB30	AtMYB27	45.63	103	54	2	40	342	11	112	6.00E-28	101
TcMYB30	ATMYB33	47.06	102	53	1	40	342	34	135	1.00E-27	104
TcMYB30	AtMYB81	48.51	101	51	1	40	339	22	122	1.00E-27	103
TcMYB30	ATMYB11	48.15	108	55	1	22	342	8	115	2.00E-27	102
TcMYB30	ATMYB68	47.57	103	52	2	40	342	14	116	2.00E-27	102
TcMYB30	ATMYB86	49.02	102	51	1	40	342	14	115	2.00E-27	102
TcMYB30	AtMYB10	46.6	103	53	2	40	342	16	117	2.00E-27	99.8
TcMYB30	ATMYB84	46.6	103	53	2	40	342	14	116	2.00E-27	101
TcMYB30	ATMYB3	43.12	109	61	1	16	339	14	122	3.00E-27	99.4
TcMYB30	ATMYB55	37.65	162	77	4	40	453	14	171	3.00E-27	101
TcMYB30	AtMYB19	45.61	114	58	3	13	342	3	115	3.00E-27	100
TcMYB30	AtMYB24	44.55	101	55	1	40	339	19	119	5.00E-27	98.2
TcMYB30	ATMYB28	48.04	102	52	1	40	342	14	115	5.00E-27	101
TcMYB30	ATMYB5	47.06	102	53	1	40	342	25	126	6.00E-27	99
TcMYB30	AtMYB50	47.06	102	53	1	40	342	14	115	7.00E-27	100
TcMYB30	ATMYB12	33.83	201	112	5	22	561	8	207	8.00E-27	100
TcMYB30	ATMYB57	46.53	101	53	1	40	339	27	127	9.00E-27	97.4
TcMYB30	ATMYB111	47.22	108	56	1	22	342	8	115	1.00E-26	99.8
TcMYB30	AtMYB76	47.06	102	53	1	40	342	14	115	2.00E-26	99.4
TcMYB30	AtMYB100	41.51	106	61	3	25	339	23	125	2.00E-26	97.1
TcMYB30	ATMYB29	47.52	101	52	1	40	339	14	114	3.00E-26	99
TcMYB30	AtMYB112	41.51	106	61	1	25	339	29	134	3.00E-26	97.1
TcMYB30	AtMYB40	38.17	131	72	3	40	405	14	143	3.00E-26	97.4
TcMYB30	AtMYB108	42.99	107	60	1	22	339	15	121	3.00E-26	98.6
TcMYB30	AtMYB104	45.1	102	54	2	40	339	18	118	3.00E-26	99.4
TcMYB30	AtMYB20	38.64	132	72	3	22	390	8	138	2.00E-25	95.5
TcMYB30	AtMYB114	41.18	102	59	1	40	342	10	111	9.00E-25	90.1
TcMYB30	AtMYB42	44.12	102	56	1	40	342	14	115	2.00E-24	92.8
TcMYB30	AtMYB85	44.12	102	56	1	40	342	14	115	8.00E-24	90.9
TcMYB30	ATMYB75	37.29	118	73	2	40	390	10	124	1.00E-23	90.1
TcMYB30	AtMYB43	41.28	109	62	2	22	342	8	115	4.00E-23	90.1
TcMYB30	ATMYB78	37.1	124	63	2	13	339	19	142	9.00E-23	89
TcMYB30	ATMYB88	36.89	103	65	0	49	357	33	135	1.00E-22	89.7
TcMYB30	ATMYB88	30.16	63	44	1	40	228	82	143	2.00E-05	37.7
TcMYB30	ATMYB90	40.2	102	60	1	40	342	10	111	2.00E-22	86.7
TcMYB30	AtMYB22	33.77	151	86	4	31	441	51	199	2.00E-22	86.7
TcMYB30	ATMYB99	41.74	115	59	2	22	342	9	123	3.00E-22	86.3
TcMYB30	AtMYB113	39.62	106	59	2	40	342	10	111	3.00E-22	86.3
TcMYB30	AtMYB124	33.33	123	82	1	49	417	28	143	6.00E-22	87.8
TcMYB30	AtMYB124	30.56	72	50	1	40	255	77	147	5.00E-06	39.7
TcMYB30	ATMYB91	29.2	226	155	4	49	711	7	216	1.00E-21	86.3
TcMYB31	ATMYB91	45.15	392	212	9	1	1167	1	358	6.00E-109	318
TcMYB31	ATMYB48	28.87	239	141	6	19	648	12	242	5.00E-29	106
TcMYB31	AtMYB82	41.23	114	67	1	7	348	13	124	8.00E-29	104
TcMYB31	ATMYB120	31.09	238	145	4	19	675	31	260	2.00E-28	109
TcMYB31	ATMYB120	29.05	148	92	4	322	726	229	375	7.00E-06	40
TcMYB31	ATMYB59-3	42.24	116	66	2	19	363	13	126	2.00E-28	104
TcMYB31	AtMYB97	32.08	240	149	5	19	696	24	234	5.00E-28	106

TcMYB31	ATMYB57	38.46	130	71	2	19	381	30	157	8.00E-28	102
TcMYB31	AtMYB79	36.17	141	90	2	19	441	11	145	4.00E-27	101
TcMYB31	AtMYB116	41.96	112	65	1	19	354	23	132	6.00E-27	101
TcMYB31	ATMYB96	28.24	255	175	6	19	759	17	264	9.00E-27	102
TcMYB31	ATMYB63	38.97	136	77	4	7	396	15	148	2.00E-26	100
TcMYB31	AtMYB76	36.25	160	102	2	19	498	17	172	2.00E-26	101
TcMYB31	ATMYB101	42.72	103	59	1	19	327	23	123	3.00E-26	102
TcMYB31	AtMYB19	39.52	124	72	2	4	366	9	130	3.00E-26	99.4
TcMYB31	AtMYB51	30.04	273	181	9	19	807	18	271	3.00E-26	100
TcMYB31	ATMYB30	27.24	257	181	4	19	771	17	247	4.00E-26	100
TcMYB31	ATMYB80	40.95	105	62	1	7	321	13	115	5.00E-26	99.8
TcMYB31	ATMYB121	37.72	114	71	1	19	360	32	143	7.00E-26	98.6
TcMYB31	ATMYB66	37.19	121	76	1	19	381	21	139	7.00E-26	96.7
TcMYB31	AtMYB45	38.58	127	74	3	4	372	15	139	9.00E-26	97.8
TcMYB31	AtMYB60	38.66	119	73	1	19	375	17	133	1.00E-25	98.2
TcMYB31	ATMYB75	35.33	167	101	5	19	498	13	171	1.00E-25	97.1
TcMYB31	AtMYB62	36.05	147	89	3	10	435	21	156	1.00E-25	97.8
TcMYB31	ATMYB71	40.57	106	63	1	19	336	23	126	2.00E-25	97.4
TcMYB31	ATMYB37	37.97	158	89	3	7	453	13	169	2.00E-25	98.6
TcMYB31	AtMYB81	30.85	201	120	4	19	564	25	223	2.00E-25	99.8
TcMYB31	AtMYB50	32.38	210	114	6	19	564	17	224	2.00E-25	97.8
TcMYB31	AtMYB24	35.07	134	85	2	19	414	22	153	3.00E-25	95.1
TcMYB31	ATMYB90	39.83	118	71	1	19	372	13	128	4.00E-25	95.9
TcMYB31	ATMYB0	32.9	155	94	2	19	453	19	171	5.00E-25	95.1
TcMYB31	ATMYB65	41.75	103	60	1	19	327	46	146	5.00E-25	99.4
TcMYB31	AtMYB27	41.51	106	62	1	10	327	11	114	6.00E-25	95.1
TcMYB31	ATMYB94	25.71	319	208	8	19	888	17	317	6.00E-25	97.1
TcMYB31	ATMYB33	41.75	103	60	1	19	327	37	137	6.00E-25	99
TcMYB31	AtMYB114	39.67	121	73	2	19	381	13	130	7.00E-25	92
TcMYB31	AtMYB112	39.62	106	64	1	10	327	34	137	9.00E-25	94.7
TcMYB31	ATMYB84	33.33	165	105	4	19	498	17	178	1.00E-24	95.9
TcMYB31	AtMYB36	34.09	176	115	4	19	543	17	178	1.00E-24	96.3
TcMYB31	ATMYB31	31.67	240	151	10	19	699	17	239	1.00E-24	95.9
TcMYB31	ATMYB3	38.53	109	67	1	19	345	25	131	2.00E-24	93.6
TcMYB31	ATMYB14	34.69	147	90	3	7	429	13	155	2.00E-24	94
TcMYB31	AtMYB74	32.04	181	107	3	19	513	18	196	2.00E-24	95.1
TcMYB31	ATMYB102	33.14	175	82	3	19	438	17	189	3.00E-24	95.5
TcMYB31	AtMYB17	44.55	101	56	1	19	321	17	115	3.00E-24	94.4
TcMYB31	ATMYB3	34.85	132	83	2	19	405	17	146	4.00E-24	93.2
TcMYB31	ATMYB123	38.02	121	72	2	10	363	16	134	4.00E-24	93.2
TcMYB31	AtMYB103	37.7	122	76	1	7	372	13	132	4.00E-24	95.1
TcMYB31	ATMYB2	38.83	103	63	1	19	327	25	125	5.00E-24	93.2
TcMYB31	ATMYB119	37.07	116	73	1	16	363	107	219	5.00E-24	95.5
TcMYB31	ATMYB119	29.49	78	51	2	175	396	105	182	6.00E-04	33.9
TcMYB31	ATMYB86	27.65	217	155	3	19	663	17	227	7.00E-24	94.4
TcMYB31	AtMYB53	41.58	101	59	1	19	321	17	115	7.00E-24	93.6
TcMYB31	AtMYB113	39.83	118	71	1	19	372	13	128	8.00E-24	92
TcMYB31	ATMYB67	28.57	203	133	5	7	579	23	207	9.00E-24	93.2
TcMYB31	ATMYB92	26.58	237	173	3	19	726	17	247	1.00E-23	93.6
TcMYB31	AtMYB98	42.86	105	60	1	16	330	219	320	1.00E-23	94.7
TcMYB31	AtMYB98	32.38	105	70	2	25	336	169	271	8.00E-10	52.4
TcMYB31	ATMYB61	28.47	281	191	8	19	831	17	284	1.00E-23	94
TcMYB31	ATMYB28	34.16	161	101	3	19	486	17	168	1.00E-23	94
TcMYB31	ATMYB55	38.76	129	69	2	19	375	17	143	1.00E-23	93.6
TcMYB31	AtMYB18	35.77	137	77	3	7	384	8	142	1.00E-23	92.4
TcMYB31	ATMYB5	33.58	137	81	3	7	387	24	158	1.00E-23	91.7
TcMYB31	ATMYB26	28.17	252	169	7	7	726	13	243	1.00E-23	93.6
TcMYB31	ATMYB38	28.31	219	148	3	7	636	13	230	1.00E-23	92.4
TcMYB31	AtMYB41	39.66	116	70	1	19	366	17	130	2.00E-23	92
TcMYB31	AtMYB104	39.25	107	65	1	10	330	18	122	2.00E-23	93.6
TcMYB31	ATMYB23	38.61	101	62	1	19	321	17	115	2.00E-23	90.5
TcMYB31	AtMYB107	39.6	101	61	1	19	321	17	115	2.00E-23	92.4
TcMYB31	ATMYB46	41.03	117	67	2	19	363	23	137	2.00E-23	91.7
TcMYB31	AtMYB108	38.89	108	66	1	7	330	20	125	2.00E-23	92.4
TcMYB31	ATMYB122	38.4	125	77	1	19	393	17	139	2.00E-23	92.4
TcMYB31	ATMYB15	40	105	63	1	7	321	13	115	3.00E-23	91.3
TcMYB31	AtMYB64	40	105	63	1	16	330	107	208	4.00E-23	92.8
TcMYB31	AtMYB64	28.57	70	49	1	130	336	90	159	2.00E-04	35.4
TcMYB31	AtMYB9	39.6	101	61	1	19	321	17	115	5.00E-23	91.7
TcMYB31	MYB8	31.45	124	85	1	19	390	17	138	5.00E-23	89
TcMYB31	AtMYB49	40.59	101	60	1	19	321	17	115	7.00E-23	90.9
TcMYB31	AtMYB10	38.89	108	66	1	7	330	15	120	9.00E-23	89
TcMYB31	ATMYB16	37.5	112	70	1	19	354	17	126	1.00E-22	90.1
TcMYB31	ATMYB29	35.15	165	107	4	19	513	17	161	2.00E-22	90.1
TcMYB31	ATMYB34	40.18	112	67	1	19	354	17	126	2.00E-22	89.4
TcMYB31	ATMYB106	37.5	112	70	1	19	354	60	169	2.00E-22	90.5
TcMYB31	AtMYB93	40.59	101	60	1	19	321	17	115	2.00E-22	90.1
TcMYB31	ATMYB58	37.27	110	69	1	7	336	15	122	2.00E-22	88.6
TcMYB31	ATMYB95	37.29	118	74	1	19	372	17	132	4.00E-22	87.8
TcMYB31	ATMYB4	42.27	97	56	1	19	309	17	111	5.00E-22	87.8

TcMYB31	ATMYB118	37.04	108	68	1	16	339	191	295	5.00E-22	89.7
TcMYB31	AtMYB42	38.98	118	72	1	1	354	11	126	1.00E-21	87
TcMYB31	ATMYB12	37.14	105	66	1	7	321	13	115	1.00E-21	88.2
TcMYB31	AtMYB32	36.61	112	71	1	19	354	17	126	1.00E-21	86.7
TcMYB31	AtMYB85	40.18	112	67	1	19	354	17	126	1.00E-21	86.3
TcMYB31	MYB7	36.61	112	71	1	19	354	17	126	1.00E-21	86.3
TcMYB31	ATMYB72	33.61	119	79	1	7	363	15	131	2.00E-21	86.7
TcMYB31	ATMYB68	38.79	116	66	3	19	351	17	130	3.00E-21	87
TcMYB31	AtMYB20	36.97	119	75	1	19	375	17	133	3.00E-21	85.5
TcMYB31	ATMYB105	30	180	120	4	10	531	107	263	6.00E-21	85.5
TcMYB31	ATMYB87	38.24	102	62	2	19	321	17	116	6.00E-21	85.1
TcMYB31	AtMYB56	38.32	107	66	1	10	330	93	196	7.00E-21	85.1
TcMYB31	AtMYB47	36.44	118	75	2	1	354	12	126	9.00E-21	84
TcMYB31	AtMYB83	35.65	115	74	1	19	363	35	147	9.00E-21	85.1
TcMYB31	AtMYB6	33.93	112	74	1	19	354	17	126	1.00E-20	83.2
TcMYB31	AtMYB117	28.33	180	129	2	10	549	98	272	1.00E-20	85.1
TcMYB31	ATMYB35	36.36	121	75	2	19	375	17	135	1.00E-20	84.3
TcMYB31	ATMYB13	34.29	140	92	3	19	438	17	143	2.00E-20	82.8
TcMYB31	AtMYB115	38.61	101	62	1	16	318	160	257	2.00E-20	84.3
TcMYB31	ATMYB25	42.42	99	57	1	4	300	48	143	3.00E-20	84
TcMYB31	ATMYB52	33.62	116	77	1	1	348	2	114	3.00E-20	82
TcMYB31	ATMYB69	33.61	119	79	1	7	363	18	133	3.00E-20	82
TcMYB31	AtMYB43	39.29	112	68	1	19	354	17	126	5.00E-20	82.8
TcMYB31	ATMYB111	33.33	105	70	1	7	321	13	115	6.00E-20	82.8
TcMYB31	ATMYB11	34.29	105	69	1	7	321	13	115	8.00E-20	82.4
TcMYB31	ATMYB73	40.43	94	56	1	19	300	16	106	8.00E-20	82
TcMYB31	ATMYB78	33.88	121	66	2	10	330	28	146	8.00E-20	82
TcMYB31	ATMYB110	33.33	129	86	1	10	396	67	192	2.00E-19	80.9
TcMYB31	ATMYB54	33.63	113	75	1	1	339	3	112	2.00E-19	79.3
TcMYB31	AtMYB70	39.81	103	58	2	4	300	7	106	2.00E-19	80.5
TcMYB31	AtMYB109	32.84	134	90	1	4	405	54	184	4.00E-19	80.9
TcMYB31	ATMYB99	37.74	106	61	1	19	321	18	123	5.00E-19	78.6
TcMYB31	ATMYB44	27.35	234	164	5	19	702	9	221	2.00E-18	77.8
TcMYB31	AtMYB1	39.36	94	57	1	19	300	58	148	3.00E-18	78.2
TcMYB31	ATMYB77	22.65	287	218	4	19	867	9	265	3.00E-18	77
TcMYB31	AtMYB40	33.62	116	77	1	7	354	13	126	7.00E-18	75.5
TcMYB31	AtMYB100	27.52	149	108	2	7	453	25	167	2.00E-17	73.9
TcMYB31	ATMYB88	29.77	131	88	2	4	384	24	151	2.00E-16	72.8
TcMYB31	AtMYB22	30	120	84	2	10	369	54	169	8.00E-16	69.3
TcMYB31	AtMYB124	28.12	128	86	3	4	369	19	143	9.00E-15	67.8
TcMYB31	AtMYB89	32.98	94	63	1	19	300	59	149	1.00E-13	62
TcMYB32	ATMYB14	81.9	116	21	0	1	348	1	116	1.00E-71	219
TcMYB32	AtMYB107	74.4	125	32	0	1	375	1	125	2.00E-70	218
TcMYB32	AtMYB9	75.2	125	31	0	1	375	1	125	1.00E-69	216
TcMYB32	ATMYB15	81.03	116	22	0	1	348	1	116	2.00E-69	214
TcMYB32	ATMYB13	54.9	204	81	4	1	579	1	192	8.00E-69	211
TcMYB32	AtMYB85	62.5	160	59	2	1	477	1	158	2.00E-68	211
TcMYB32	ATMYB102	37.32	343	213	8	1	1023	1	316	3.00E-68	213
TcMYB32	AtMYB17	70.15	134	40	0	1	402	1	134	4.00E-68	211
TcMYB32	AtMYB42	57.14	182	74	1	1	534	1	182	6.00E-68	211
TcMYB32	AtMYB93	45.56	259	127	5	1	735	1	252	7.00E-68	213
TcMYB32	AtMYB41	74.4	125	32	0	1	375	1	125	1.00E-67	210
TcMYB32	AtMYB74	72.66	128	34	1	1	381	1	128	3.00E-67	210
TcMYB32	ATMYB16	68.12	138	44	0	1	414	1	138	4.00E-67	210
TcMYB32	ATMYB58	78.26	115	25	0	4	348	4	118	3.00E-66	206
TcMYB32	ATMYB92	50.7	213	87	4	1	585	1	207	5.00E-65	204
TcMYB32	ATMYB106	68.89	135	37	1	10	399	47	181	2.00E-64	205
TcMYB32	AtMYB43	72.8	125	34	0	1	375	1	125	2.00E-64	203
TcMYB32	AtMYB53	56.07	173	66	2	1	489	1	173	4.00E-64	201
TcMYB32	ATMYB72	69.23	130	40	1	4	393	4	128	4.00E-63	198
TcMYB32	ATMYB5	69.42	121	37	0	13	375	16	136	6.00E-63	196
TcMYB32	AtMYB20	69.6	125	38	0	1	375	1	125	8.00E-63	197
TcMYB32	AtMYB10	68.5	127	40	0	7	387	5	131	2.00E-62	195
TcMYB32	ATMYB63	74.78	115	29	0	4	348	4	118	6.00E-62	195
TcMYB32	AtMYB6	66.4	125	42	0	1	375	1	125	1.00E-61	192
TcMYB32	MYB8	64	125	45	0	1	375	1	125	3.00E-61	191
TcMYB32	ATMYB3	64.06	128	46	0	1	384	1	128	9.00E-61	191
TcMYB32	AtMYB49	56.55	168	72	2	1	501	1	166	1.00E-60	193
TcMYB32	MYB7	64.8	125	44	0	1	375	1	125	4.00E-60	190
TcMYB32	ATMYB4	64.8	125	44	0	1	375	1	125	5.00E-60	190
TcMYB32	AtMYB32	65.6	125	43	0	1	375	1	125	6.00E-60	189
TcMYB32	ATMYB28	53.23	186	78	3	1	531	1	183	7.00E-60	192
TcMYB32	ATMYB12	68.1	116	37	0	1	348	1	116	2.00E-58	188
TcMYB32	AtMYB51	65.08	126	43	1	1	375	1	126	5.00E-58	187
TcMYB32	ATMYB11	68.1	116	37	0	1	348	1	116	6.00E-58	186
TcMYB32	AtMYB60	65.57	122	42	0	1	366	1	122	8.00E-58	184
TcMYB32	AtMYB40	64.8	125	44	0	1	375	1	125	2.00E-57	182
TcMYB32	ATMYB111	66.38	116	39	0	1	348	1	116	2.00E-57	185
TcMYB32	ATMYB34	64.8	125	44	0	1	375	1	125	2.00E-57	183
TcMYB32	ATMYB94	68.07	119	38	0	1	357	1	119	3.00E-57	184

TcMYB32	ATMYB30	52.05	171	78	1	1	501	1	171	4.00E-57	184
TcMYB32	ATMYB99	61.83	131	43	1	4	375	3	133	5.00E-57	181
TcMYB32	ATMYB31	67.5	120	39	0	1	360	1	120	5.00E-57	184
TcMYB32	ATMYB80	64	125	45	0	1	375	1	125	5.00E-57	183
TcMYB32	ATMYB122	64	125	45	0	1	375	1	125	1.00E-56	182
TcMYB32	ATMYB29	50	188	94	1	1	564	1	176	1.00E-56	182
TcMYB32	ATMYB96	66.39	119	40	0	1	357	1	119	3.00E-56	182
TcMYB32	AtMYB76	65.6	125	43	0	1	375	1	125	4.00E-56	181
TcMYB32	ATMYB86	65.6	125	43	0	1	375	1	125	9.00E-56	181
TcMYB32	ATMYB35	61.6	125	48	0	1	375	1	125	2.00E-55	179
TcMYB32	ATMYB61	63.2	125	46	0	1	375	1	125	1.00E-54	178
TcMYB32	AtMYB50	60.8	125	49	0	1	375	1	125	1.00E-53	174
TcMYB32	ATMYB37	55.03	149	66	2	1	444	1	148	1.00E-52	172
TcMYB32	ATMYB95	51.92	156	75	0	1	468	1	156	1.00E-52	170
TcMYB32	ATMYB55	40.16	244	129	5	1	681	1	237	1.00E-52	172
TcMYB32	ATMYB67	53.96	139	64	0	16	432	16	154	2.00E-52	171
TcMYB32	AtMYB103	49.71	173	86	2	1	516	1	169	3.00E-52	172
TcMYB32	ATMYB87	63.56	118	42	1	1	351	1	118	5.00E-52	170
TcMYB32	ATMYB84	64.41	118	41	1	1	351	1	118	3.00E-51	168
TcMYB32	ATMYB68	65.25	118	40	1	1	351	1	118	4.00E-51	169
TcMYB32	AtMYB36	64.41	118	41	1	1	351	1	118	1.00E-50	167
TcMYB32	ATMYB38	56.43	140	53	2	1	396	1	140	6.00E-50	164
TcMYB32	ATMYB26	57.94	126	44	1	1	351	1	126	1.00E-49	165
TcMYB32	AtMYB47	58.4	125	52	0	1	375	1	125	2.00E-49	162
TcMYB32	ATMYB66	47.24	163	86	2	37	525	17	174	1.00E-47	155
TcMYB32	ATMYB123	67.62	105	34	0	34	348	14	118	1.00E-47	157
TcMYB32	AtMYB24	48.45	161	83	1	34	516	17	175	5.00E-47	154
TcMYB32	ATMYB23	60.58	104	41	0	37	348	13	116	9.00E-47	153
TcMYB32	ATMYB46	62.96	108	40	0	34	357	18	125	2.00E-46	154
TcMYB32	ATMYB101	60	105	42	0	31	345	17	121	7.00E-46	158
TcMYB32	AtMYB83	60.55	109	43	0	22	348	26	134	8.00E-46	154
TcMYB32	AtMYB82	60.75	107	42	0	25	345	9	115	1.00E-45	149
TcMYB32	AtMYB108	59.13	115	47	0	22	366	15	129	5.00E-45	152
TcMYB32	ATMYB3	56.52	115	50	0	34	378	20	134	8.00E-45	148
TcMYB32	ATMYB0	57.14	105	45	0	37	351	15	119	2.00E-44	147
TcMYB32	AtMYB79	58.41	113	47	1	37	375	7	114	8.00E-44	147
TcMYB32	AtMYB116	57.27	110	47	0	37	366	19	128	1.00E-43	147
TcMYB32	ATMYB71	59.05	105	43	0	31	345	17	121	1.00E-43	146
TcMYB32	AtMYB114	58.1	105	44	0	31	345	7	111	3.00E-43	141
TcMYB32	ATMYB33	57.41	108	46	0	22	345	28	135	3.00E-43	151
TcMYB32	AtMYB62	44.85	165	91	1	34	528	19	177	3.00E-43	146
TcMYB32	ATMYB48	50.78	128	61	1	31	408	6	133	8.00E-43	144
TcMYB32	ATMYB121	59.43	106	43	0	25	342	24	129	9.00E-43	144
TcMYB32	AtMYB112	57.66	111	47	0	34	366	32	142	1.00E-42	143
TcMYB32	ATMYB120	59.81	107	43	1	34	354	26	131	3.00E-42	149
TcMYB32	ATMYB75	58.1	105	44	0	31	345	7	111	3.00E-42	142
TcMYB32	ATMYB57	58.1	105	44	0	34	348	25	129	6.00E-42	140
TcMYB32	ATMYB65	57.69	104	44	0	34	345	41	144	6.00E-42	148
TcMYB32	ATMYB2	55.86	111	49	0	34	366	20	130	6.00E-42	142
TcMYB32	AtMYB97	56.31	103	45	0	34	342	19	121	1.00E-41	144
TcMYB32	ATMYB59-3	52.99	117	53	1	37	381	9	125	1.00E-41	140
TcMYB32	AtMYB81	55.66	106	47	0	25	342	17	122	2.00E-41	144
TcMYB32	AtMYB19	58.18	110	46	0	25	354	9	118	3.00E-41	140
TcMYB32	AtMYB113	53.91	115	53	1	1	345	1	111	9.00E-41	138
TcMYB32	ATMYB90	57.14	105	45	0	31	345	7	111	1.00E-40	138
TcMYB32	ATMYB78	50.39	129	50	1	22	366	22	150	4.00E-39	136
TcMYB32	AtMYB18	56.6	106	46	0	37	354	11	116	2.00E-38	133
TcMYB32	AtMYB27	58.65	104	43	0	34	345	9	112	3.00E-38	131
TcMYB32	AtMYB45	53.92	102	47	0	37	342	19	120	2.00E-37	130
TcMYB32	AtMYB104	43.93	107	60	1	40	360	18	120	1.00E-28	108
TcMYB32	AtMYB1	40.85	142	68	2	40	417	55	195	3.00E-28	107
TcMYB32	AtMYB115	35.58	163	98	4	34	501	156	316	5.00E-28	106
TcMYB32	ATMYB77	46.08	102	55	1	40	345	6	106	1.00E-27	104
TcMYB32	ATMYB25	48.54	103	52	2	40	345	50	150	2.00E-27	105
TcMYB32	AtMYB98	42.86	112	64	1	25	360	212	322	4.00E-27	105
TcMYB32	AtMYB109	48.54	103	52	2	40	345	56	156	4.00E-27	104
TcMYB32	ATMYB73	46.08	102	55	1	40	345	13	113	6.00E-27	102
TcMYB32	AtMYB70	46.08	102	55	1	40	345	13	113	9.00E-27	102
TcMYB32	ATMYB44	35.98	164	82	3	40	462	6	168	1.00E-26	101
TcMYB32	ATMYB118	41.8	122	71	1	25	390	184	304	1.00E-26	103
TcMYB32	ATMYB119	41.12	107	63	1	40	360	105	210	2.00E-26	103
TcMYB32	ATMYB54	43.33	120	64	3	40	387	6	123	5.00E-26	98.6
TcMYB32	ATMYB52	44.34	106	59	1	28	345	1	105	2.00E-25	97.1
TcMYB32	ATMYB52	32.08	53	35	1	199	354	5	57	6.00E-04	33.5
TcMYB32	AtMYB64	39.25	107	65	1	40	360	105	210	6.00E-25	98.6
TcMYB32	ATMYB105	42.06	107	62	1	25	345	102	207	2.00E-23	92.8
TcMYB32	ATMYB69	36.59	123	78	1	25	393	14	135	4.00E-23	90.5
TcMYB32	AtMYB117	42.16	102	59	1	40	345	98	198	4.00E-22	89.7
TcMYB32	AtMYB100	41.18	102	60	1	25	330	21	121	5.00E-22	87
TcMYB32	AtMYB56	41.18	102	60	1	40	345	93	193	3.00E-21	86.7

TcMYB32	ATMYB110	39.81	103	62	1	40	348	67	168	4.00E-21	85.9
TcMYB32	AtMYB89	39.42	104	63	2	43	354	57	158	3.00E-19	78.2
TcMYB32	ATMYB88	41.41	99	58	1	49	345	33	130	4.00E-19	81.6
TcMYB32	AtMYB124	40.4	99	59	1	49	345	28	125	1.00E-18	79.7
TcMYB32	ATMYB91	35.24	105	66	1	37	345	3	107	2.00E-18	78.6
TcMYB32	AtMYB22	36.27	102	65	2	25	330	49	148	1.00E-17	75.1
TcMYB33	ATMYB73	41.81	342	196	7	22	1038	7	305	2.00E-78	236
TcMYB33	AtMYB70	43.08	318	172	9	22	948	7	273	1.00E-70	216
TcMYB33	ATMYB44	40.25	323	177	7	31	951	3	278	1.00E-67	208
TcMYB33	ATMYB77	38.39	310	188	5	31	951	3	271	5.00E-67	206
TcMYB33	AtMYB109	68.93	103	32	0	34	342	54	156	1.00E-50	167
TcMYB33	AtMYB1	33.72	347	220	9	31	1041	52	351	3.00E-49	163
TcMYB33	ATMYB25	60.55	109	43	0	16	342	42	150	1.00E-45	152
TcMYB33	ATMYB54	55.45	101	45	0	40	342	6	106	5.00E-41	137
TcMYB33	ATMYB52	52.48	101	48	0	40	342	5	105	2.00E-39	133
TcMYB33	AtMYB117	52.83	106	50	0	40	357	98	203	6.00E-39	135
TcMYB33	AtMYB56	56.44	101	44	0	40	342	93	193	1.00E-38	133
TcMYB33	ATMYB105	54.46	101	46	0	40	342	107	207	2.00E-38	132
TcMYB33	ATMYB69	52.48	101	48	0	40	342	19	119	5.00E-38	129
TcMYB33	ATMYB110	47.32	112	57	1	13	342	56	167	1.00E-34	122
TcMYB33	AtMYB64	39.33	150	80	2	37	453	104	253	1.00E-32	119
TcMYB33	ATMYB119	40.97	144	81	2	7	426	94	237	2.00E-32	119
TcMYB33	AtMYB98	46.96	115	61	0	4	348	205	319	2.00E-32	118
TcMYB33	AtMYB47	39.63	164	90	3	40	504	14	177	2.00E-32	115
TcMYB33	ATMYB118	49.11	112	57	0	13	348	180	291	4.00E-32	117
TcMYB33	ATMYB34	52.94	102	47	1	40	342	14	115	6.00E-32	114
TcMYB33	AtMYB49	48.7	115	58	1	1	342	1	115	7.00E-32	115
TcMYB33	ATMYB121	52.48	101	47	1	40	339	29	129	8.00E-32	114
TcMYB33	ATMYB92	50.98	102	49	1	40	342	14	115	2.00E-31	114
TcMYB33	ATMYB96	32.78	241	153	7	22	717	8	228	2.00E-31	114
TcMYB33	ATMYB80	50	104	51	1	40	348	14	117	3.00E-31	113
TcMYB33	AtMYB97	52.48	101	47	1	40	339	21	121	3.00E-31	114
TcMYB33	ATMYB46	41.38	145	84	2	40	471	20	158	3.00E-31	112
TcMYB33	AtMYB41	51.96	102	48	1	40	342	14	115	3.00E-31	112
TcMYB33	ATMYB23	46.96	115	60	1	1	342	1	115	4.00E-31	110
TcMYB33	AtMYB51	51.96	102	48	1	40	342	15	116	5.00E-31	113
TcMYB33	ATMYB95	51.96	102	48	1	40	342	14	115	5.00E-31	111
TcMYB33	AtMYB89	45.45	99	54	0	43	339	57	155	6.00E-31	109
TcMYB33	AtMYB60	43.41	129	68	3	40	411	14	126	6.00E-31	111
TcMYB33	ATMYB31	30.77	273	173	9	22	792	8	268	8.00E-31	112
TcMYB33	ATMYB94	39.47	152	82	5	40	465	14	159	9.00E-31	112
TcMYB33	ATMYB14	50.96	104	50	1	40	348	14	117	1.00E-30	110
TcMYB33	ATMYB35	51.96	102	48	1	40	342	14	115	1.00E-30	111
TcMYB33	ATMYB63	48.18	110	56	1	40	366	16	125	2.00E-30	110
TcMYB33	ATMYB63	25.97	77	57	1	31	261	66	141	5.00E-04	33.5
TcMYB33	AtMYB107	50	102	50	1	40	342	14	115	4.00E-30	110
TcMYB33	ATMYB30	35.18	199	105	6	40	564	14	201	4.00E-30	110
TcMYB33	ATMYB106	51.96	102	48	1	40	342	57	158	4.00E-30	111
TcMYB33	AtMYB115	41.86	129	75	1	40	426	158	279	5.00E-30	110
TcMYB33	AtMYB82	49.02	102	51	1	40	342	14	115	5.00E-30	107
TcMYB33	ATMYB66	50	102	50	1	40	342	18	119	6.00E-30	107
TcMYB33	ATMYB13	44.19	129	70	3	22	402	8	127	6.00E-30	108
TcMYB33	ATMYB26	47.83	115	50	2	40	354	14	128	8.00E-30	110
TcMYB33	ATMYB48	46.3	108	57	2	22	342	4	110	1.00E-29	107
TcMYB33	AtMYB74	50.98	102	49	1	40	342	15	116	1.00E-29	109
TcMYB33	ATMYB16	50.98	102	49	1	40	342	14	115	1.00E-29	109
TcMYB33	AtMYB53	45.22	115	62	1	1	342	1	115	1.00E-29	108
TcMYB33	ATMYB101	50.98	102	49	1	40	342	20	121	1.00E-29	111
TcMYB33	AtMYB79	49.07	108	54	2	22	342	3	109	1.00E-29	107
TcMYB33	AtMYB45	38.41	164	100	3	40	528	20	172	1.00E-29	107
TcMYB33	ATMYB72	44.19	129	68	4	40	414	16	143	1.00E-29	108
TcMYB33	ATMYB59-3	45.1	102	55	1	40	342	10	111	1.00E-29	107
TcMYB33	AtMYB17	48.54	103	51	2	40	342	14	115	2.00E-29	108
TcMYB33	AtMYB9	50.98	102	49	1	40	342	14	115	2.00E-29	108
TcMYB33	AtMYB6	35.91	181	115	3	22	561	8	177	2.00E-29	106
TcMYB33	ATMYB122	49.5	101	50	1	40	339	14	114	3.00E-29	108
TcMYB33	AtMYB62	33.7	181	119	4	7	546	10	171	4.00E-29	107
TcMYB33	ATMYB4	49.07	108	54	1	22	342	8	115	6.00E-29	106
TcMYB33	AtMYB93	48.04	102	52	1	40	342	14	115	7.00E-29	107
TcMYB33	MYB8	45.37	108	58	1	22	342	8	115	1.00E-28	103
TcMYB33	ATMYB3	47.22	108	56	1	22	342	8	115	1.00E-28	105
TcMYB33	ATMYB0	44.17	120	65	2	40	393	16	135	1.00E-28	104
TcMYB33	ATMYB71	44.55	110	60	1	40	366	20	129	1.00E-28	105
TcMYB33	AtMYB83	50.47	107	52	1	40	357	32	138	1.00E-28	106
TcMYB33	ATMYB102	50	102	50	1	40	342	14	115	1.00E-28	106
TcMYB33	AtMYB32	41.72	151	87	2	40	489	14	155	2.00E-28	104
TcMYB33	AtMYB116	43.52	108	60	1	22	342	14	121	2.00E-28	104
TcMYB33	ATMYB67	49.02	102	51	1	40	342	24	125	3.00E-28	105
TcMYB33	ATMYB37	49.51	103	50	2	40	342	14	116	3.00E-28	105
TcMYB33	ATMYB15	46.3	108	57	1	22	342	8	115	3.00E-28	104

TcMYB33	AtMYB36	47.57	103	52	2	40	342	14	116	4.00E-28	105
TcMYB33	ATMYB38	49.51	103	50	2	40	342	14	116	4.00E-28	104
TcMYB33	AtMYB18	46.09	115	61	2	1	342	1	113	5.00E-28	103
TcMYB33	ATMYB65	47.06	102	53	1	40	342	43	144	5.00E-28	107
TcMYB33	ATMYB87	45.95	111	58	2	40	366	14	124	5.00E-28	104
TcMYB33	ATMYB123	48.15	108	55	2	22	342	12	117	5.00E-28	103
TcMYB33	MYB7	49.02	102	51	1	40	342	14	115	7.00E-28	103
TcMYB33	AtMYB103	50	102	50	1	40	342	14	115	7.00E-28	105
TcMYB33	ATMYB2	44.35	115	63	2	22	363	16	129	7.00E-28	103
TcMYB33	ATMYB120	49.5	101	50	1	40	339	28	128	7.00E-28	106
TcMYB33	ATMYB58	33.96	212	136	5	40	663	16	194	1.00E-27	102
TcMYB33	ATMYB61	35.09	171	110	2	40	549	14	179	1.00E-27	104
TcMYB33	AtMYB27	45.63	103	54	2	40	342	11	112	2.00E-27	101
TcMYB33	AtMYB81	48.51	101	51	1	40	339	22	122	3.00E-27	103
TcMYB33	ATMYB33	47.06	102	53	1	40	342	34	135	3.00E-27	104
TcMYB33	ATMYB11	48.15	108	55	1	22	342	8	115	4.00E-27	102
TcMYB33	ATMYB68	47.57	103	52	2	40	342	14	116	5.00E-27	102
TcMYB33	ATMYB86	49.02	102	51	1	40	342	14	115	5.00E-27	102
TcMYB33	AtMYB10	46.6	103	53	2	40	342	16	117	7.00E-27	99.8
TcMYB33	ATMYB84	46.6	103	53	2	40	342	14	116	7.00E-27	101
TcMYB33	ATMYB3	43.12	109	61	1	16	339	14	122	7.00E-27	99.4
TcMYB33	AtMYB19	45.61	114	58	3	13	342	3	115	9.00E-27	100
TcMYB33	ATMYB55	37.65	162	77	4	40	453	14	171	9.00E-27	101
TcMYB33	AtMYB24	44.55	101	55	1	40	339	19	119	1.00E-26	98.2
TcMYB33	ATMYB28	48.04	102	52	1	40	342	14	115	2.00E-26	101
TcMYB33	ATMYB5	47.06	102	53	1	40	342	25	126	2.00E-26	99
TcMYB33	AtMYB50	47.06	102	53	1	40	342	14	115	2.00E-26	100
TcMYB33	ATMYB57	46.53	101	53	1	40	339	27	127	2.00E-26	97.4
TcMYB33	ATMYB111	47.22	108	56	1	22	342	8	115	4.00E-26	99.8
TcMYB33	ATMYB12	46.3	108	57	1	22	342	8	115	4.00E-26	100
TcMYB33	ATMYB12	25.23	107	77	2	31	342	64	169	1.00E-04	35.8
TcMYB33	AtMYB76	47.06	102	53	1	40	342	14	115	5.00E-26	99.4
TcMYB33	AtMYB100	41.51	106	61	3	25	339	23	125	6.00E-26	97.1
TcMYB33	ATMYB29	47.52	101	52	1	40	339	14	114	7.00E-26	99
TcMYB33	AtMYB112	41.51	106	61	1	25	339	29	134	7.00E-26	97.1
TcMYB33	AtMYB40	38.17	131	72	3	40	405	14	143	8.00E-26	97.4
TcMYB33	AtMYB108	42.99	107	60	1	22	339	15	121	8.00E-26	98.6
TcMYB33	AtMYB104	45.1	102	54	2	40	339	18	118	9.00E-26	99.4
TcMYB33	AtMYB20	38.64	132	72	3	22	390	8	138	5.00E-25	95.5
TcMYB33	AtMYB114	41.18	102	59	1	40	342	10	111	2.00E-24	90.1
TcMYB33	AtMYB42	44.12	102	56	1	40	342	14	115	5.00E-24	92.8
TcMYB33	AtMYB85	44.12	102	56	1	40	342	14	115	2.00E-23	90.9
TcMYB33	ATMYB75	37.29	118	73	2	40	390	10	124	3.00E-23	90.1
TcMYB33	AtMYB43	41.28	109	62	2	22	342	8	115	9.00E-23	90.1
TcMYB33	ATMYB78	37.1	124	63	2	13	339	19	142	2.00E-22	89
TcMYB33	ATMYB88	36.89	103	65	0	49	357	33	135	4.00E-22	89.7
TcMYB33	ATMYB88	30.16	63	44	1	40	228	82	143	3.00E-05	37.7
TcMYB33	ATMYB90	40.2	102	60	1	40	342	10	111	5.00E-22	86.7
TcMYB33	AtMYB22	33.77	151	86	4	31	441	51	199	5.00E-22	86.7
TcMYB33	ATMYB99	41.74	115	59	2	22	342	9	123	6.00E-22	86.3
TcMYB33	AtMYB113	39.62	106	59	2	40	342	10	111	6.00E-22	86.3
TcMYB33	ATMYB91	29.07	227	156	4	49	714	7	216	1.00E-21	87.4
TcMYB33	AtMYB124	33.33	123	82	1	49	417	28	143	1.00E-21	87.8
TcMYB33	AtMYB124	30.56	72	50	1	40	255	77	147	7.00E-06	39.7
TcMYB34	AtMYB20	84.62	130	20	0	1	390	1	130	3.00E-85	253
TcMYB34	AtMYB85	56.22	217	89	3	1	633	1	208	4.00E-84	249
TcMYB34	AtMYB43	78.52	135	29	0	1	405	1	135	8.00E-82	245
TcMYB34	AtMYB42	71.43	161	40	3	1	465	1	160	3.00E-81	242
TcMYB34	ATMYB99	57.89	190	72	3	4	549	3	188	5.00E-73	220
TcMYB34	AtMYB40	71.64	134	37	1	1	399	1	134	2.00E-71	216
TcMYB34	AtMYB107	55.17	203	86	5	1	594	1	198	1.00E-69	214
TcMYB34	AtMYB17	74.42	129	33	0	1	387	1	129	2.00E-69	213
TcMYB34	AtMYB9	62.05	166	61	3	1	492	1	165	2.00E-69	214
TcMYB34	ATMYB16	55.32	188	81	2	1	555	1	181	3.00E-69	213
TcMYB34	ATMYB102	72.52	131	36	0	1	393	1	131	1.00E-67	209
TcMYB34	AtMYB93	60.24	166	66	2	1	498	1	162	2.00E-67	209
TcMYB34	AtMYB41	70.99	131	38	0	1	393	1	131	2.00E-66	204
TcMYB34	AtMYB6	47.01	234	105	5	1	645	1	223	1.00E-65	201
TcMYB34	AtMYB74	70.45	132	38	1	1	393	1	132	2.00E-65	203
TcMYB34	AtMYB50	50.23	217	102	4	1	633	1	205	5.00E-65	202
TcMYB34	ATMYB4	56.14	171	74	2	1	510	1	167	1.00E-64	199
TcMYB34	ATMYB92	49.28	207	105	2	1	621	1	203	2.00E-64	201
TcMYB34	ATMYB106	59.26	162	66	1	13	498	48	202	5.00E-64	201
TcMYB34	MYB8	52.97	185	80	3	1	534	1	175	5.00E-64	196
TcMYB34	AtMYB53	66.41	131	44	0	1	393	1	131	6.00E-64	199
TcMYB34	ATMYB86	54.29	175	80	1	1	525	1	158	1.00E-63	199
TcMYB34	ATMYB3	58.12	160	67	1	1	480	1	152	2.00E-63	196
TcMYB34	MYB7	65.19	135	47	0	1	405	1	135	2.00E-63	196
TcMYB34	AtMYB32	59.21	152	62	0	1	456	1	152	2.00E-63	196
TcMYB34	ATMYB61	67.18	131	43	0	1	393	1	131	3.00E-61	194

TcMYB34	ATMYB80	53.37	178	66	3	1	483	1	178	4.00E-61	192
TcMYB34	ATMYB5	66.4	125	42	0	13	387	16	140	2.00E-60	187
TcMYB34	AtMYB49	68.75	128	40	0	1	384	1	128	3.00E-60	189
TcMYB34	ATMYB13	49.74	189	95	2	1	567	1	173	8.00E-60	186
TcMYB34	ATMYB28	62.88	132	49	0	1	396	1	132	2.00E-59	189
TcMYB34	ATMYB15	46.27	201	96	2	1	567	1	199	5.00E-59	185
TcMYB34	ATMYB29	64.62	130	46	0	1	390	1	130	2.00E-58	185
TcMYB34	ATMYB14	46.01	213	109	4	1	621	1	194	3.00E-58	182
TcMYB34	AtMYB76	45.73	199	107	2	1	594	1	197	4.00E-58	184
TcMYB34	ATMYB34	42.92	240	128	5	1	693	1	226	2.00E-57	181
TcMYB34	ATMYB35	45	220	115	4	1	642	1	212	5.00E-57	181
TcMYB34	ATMYB111	67.24	116	38	0	1	348	1	116	3.00E-56	180
TcMYB34	ATMYB63	50.85	177	86	2	4	531	4	175	6.00E-56	177
TcMYB34	ATMYB58	67.24	116	38	0	4	351	4	119	1.00E-55	176
TcMYB34	AtMYB51	63.78	127	45	1	1	378	1	127	1.00E-55	178
TcMYB34	ATMYB12	67.24	116	38	0	1	348	1	116	2.00E-55	179
TcMYB34	ATMYB67	63.64	121	44	0	16	378	16	136	2.00E-55	177
TcMYB34	ATMYB11	37.25	306	187	6	1	903	1	271	2.00E-55	177
TcMYB34	ATMYB122	61.83	131	50	0	1	393	1	131	1.00E-54	176
TcMYB34	ATMYB55	61.59	138	41	1	1	378	1	138	1.00E-54	176
TcMYB34	ATMYB72	66.38	116	39	0	4	351	4	119	1.00E-54	174
TcMYB34	ATMYB94	65.55	119	41	0	1	357	1	119	3.00E-54	174
TcMYB34	AtMYB60	63.93	122	44	0	1	366	1	122	4.00E-54	172
TcMYB34	AtMYB10	63.03	119	44	0	7	363	5	123	6.00E-54	171
TcMYB34	ATMYB30	63.93	122	44	0	1	366	1	122	6.00E-53	171
TcMYB34	ATMYB96	43.12	218	124	2	1	654	1	184	7.00E-53	171
TcMYB34	ATMYB31	65.55	119	41	0	1	357	1	119	8.00E-53	171
TcMYB34	ATMYB95	55.1	147	66	0	1	441	1	147	1.00E-52	168
TcMYB34	AtMYB47	58.82	136	56	1	1	408	1	135	2.00E-52	167
TcMYB34	AtMYB103	50.31	161	78	2	1	477	1	161	3.00E-52	170
TcMYB34	ATMYB26	62.4	125	38	1	1	348	1	125	8.00E-51	166
TcMYB34	ATMYB46	66.67	108	36	0	34	357	18	125	2.00E-48	158
TcMYB34	ATMYB38	54.74	137	60	2	1	405	1	137	1.00E-47	156
TcMYB34	AtMYB19	43.27	208	88	5	37	570	13	216	2.00E-47	154
TcMYB34	ATMYB84	60.68	117	45	1	1	348	1	117	4.00E-47	155
TcMYB34	ATMYB68	60.68	117	45	1	1	348	1	117	4.00E-47	157
TcMYB34	ATMYB37	59.83	117	46	1	1	348	1	117	6.00E-47	155
TcMYB34	AtMYB36	59.83	117	46	1	1	348	1	117	2.00E-46	154
TcMYB34	AtMYB83	62.39	109	41	0	22	348	26	134	6.00E-46	153
TcMYB34	ATMYB71	63.81	105	38	0	31	345	17	121	7.00E-46	150
TcMYB34	AtMYB79	64.08	103	37	0	37	345	7	109	3.00E-45	149
TcMYB34	AtMYB108	57.39	115	49	0	22	366	15	129	5.00E-45	150
TcMYB34	ATMYB87	57.26	117	49	1	1	348	1	117	1.00E-44	149
TcMYB34	ATMYB57	55.65	115	51	0	34	378	25	139	1.00E-44	145
TcMYB34	AtMYB18	64.08	103	37	0	37	345	11	113	2.00E-44	147
TcMYB34	ATMYB33	39.57	235	129	9	22	687	28	235	2.00E-44	152
TcMYB34	ATMYB101	58.62	116	48	1	31	378	17	131	4.00E-44	151
TcMYB34	ATMYB121	62.5	104	39	0	31	342	26	129	5.00E-44	146
TcMYB34	AtMYB62	57.66	111	47	0	34	366	19	129	6.00E-44	146
TcMYB34	ATMYB3	50	134	67	1	34	435	20	151	1.00E-43	143
TcMYB34	AtMYB116	55.26	114	51	0	37	378	19	132	2.00E-43	145
TcMYB34	AtMYB24	56.88	109	47	0	34	360	17	125	2.00E-43	142
TcMYB34	ATMYB123	60.75	107	42	0	34	354	14	120	2.00E-43	144
TcMYB34	ATMYB66	60.58	104	41	0	37	348	17	120	4.00E-43	141
TcMYB34	AtMYB112	55.26	114	51	0	25	366	29	142	4.00E-43	142
TcMYB34	ATMYB23	60.58	104	41	0	37	348	13	116	5.00E-43	142
TcMYB34	ATMYB120	59.02	122	50	2	34	399	26	145	6.00E-43	149
TcMYB34	ATMYB65	59.62	104	42	0	34	345	41	144	2.00E-42	147
TcMYB34	ATMYB0	57.69	104	44	0	37	348	15	118	9.00E-42	139
TcMYB34	AtMYB81	42.44	172	94	3	25	525	17	185	2.00E-41	143
TcMYB34	AtMYB97	58.04	112	47	1	28	363	17	127	3.00E-41	142
TcMYB34	ATMYB48	59.05	105	43	0	31	345	6	110	3.00E-41	138
TcMYB34	ATMYB59-3	60.19	103	41	0	37	345	9	111	3.00E-41	137
TcMYB34	AtMYB45	57.84	102	43	0	37	342	19	120	6.00E-41	137
TcMYB34	ATMYB2	34.51	255	140	6	34	717	20	262	2.00E-40	137
TcMYB34	AtMYB114	59.05	105	43	0	31	345	7	111	2.00E-40	132
TcMYB34	AtMYB82	49.24	132	58	1	34	402	12	143	2.00E-40	134
TcMYB34	ATMYB75	38.74	191	109	2	31	579	7	196	6.00E-40	134
TcMYB34	ATMYB90	60	105	42	0	31	345	7	111	2.00E-39	133
TcMYB34	AtMYB27	57.69	104	44	0	34	345	9	112	7.00E-39	131
TcMYB34	AtMYB113	54.78	115	52	1	1	345	1	111	9.00E-38	129
TcMYB34	ATMYB78	44.96	129	57	1	22	366	22	150	5.00E-37	129
TcMYB34	AtMYB104	39.87	153	92	3	40	498	18	160	2.00E-31	115
TcMYB34	AtMYB64	45.28	106	57	2	40	354	105	208	5.00E-29	108
TcMYB34	ATMYB119	46.3	108	57	2	34	354	103	208	1.00E-28	108
TcMYB34	ATMYB119	22.39	67	52	0	211	411	110	176	3.00E-05	37.7
TcMYB34	AtMYB98	45.05	111	60	2	25	354	212	320	3.00E-28	107
TcMYB34	ATMYB118	43.38	136	74	4	25	423	184	309	1.00E-27	105
TcMYB34	ATMYB25	45.54	112	60	2	40	372	50	159	1.00E-25	98.6
TcMYB34	ATMYB44	30.03	293	167	10	40	804	6	293	2.00E-25	97.4

TcMYB34	ATMYB73	45.37	108	58	2	40	360	13	118	2.00E-25	97.4
TcMYB34	AtMYB109	48.54	103	52	2	40	345	56	156	3.00E-25	98.2
TcMYB34	AtMYB70	45.37	108	58	2	40	360	13	118	2.00E-24	94.7
TcMYB34	AtMYB1	45.63	103	55	2	40	345	55	155	2.00E-24	95.9
TcMYB34	AtMYB1	21.88	192	138	4	184	723	50	236	2.00E-05	38.5
TcMYB34	ATMYB77	43.81	105	58	2	40	351	6	108	2.00E-24	94.4
TcMYB34	AtMYB115	42.71	96	55	1	40	327	158	252	5.00E-24	94
TcMYB34	AtMYB100	33.71	178	117	5	25	555	21	175	1.00E-23	90.9
TcMYB34	ATMYB105	40.74	108	63	2	25	345	102	207	3.00E-22	88.6
TcMYB34	ATMYB54	41.75	103	59	2	40	345	6	106	8.00E-22	85.9
TcMYB34	ATMYB52	40.78	103	60	2	40	345	5	105	2.00E-21	85.1
TcMYB34	AtMYB117	41.9	105	60	2	34	345	96	198	4.00E-21	85.9
TcMYB34	ATMYB110	38.46	104	63	2	40	348	67	168	8.00E-20	81.3
TcMYB34	AtMYB22	33.04	112	75	1	25	360	49	158	4.00E-19	78.6
TcMYB34	AtMYB56	37.25	102	64	1	40	345	93	193	5.00E-19	79.3
TcMYB34	ATMYB69	37.04	108	67	2	25	345	14	119	2.00E-18	76.6
TcMYB34	ATMYB91	38.61	101	60	1	49	345	7	107	2.00E-18	78.2
TcMYB34	AtMYB89	35.42	96	62	1	43	330	57	151	1.00E-15	67.4
TcMYB34	AtMYB124	37.37	99	62	1	49	345	28	125	3.00E-14	65.9
TcMYB34	ATMYB88	37.37	99	62	1	49	345	33	130	7.00E-14	64.7
TcMYB35	ATMYB65	38.36	451	262	13	13	1317	12	414	5.00E-79	251
TcMYB35	ATMYB33	40.83	436	244	15	43	1308	8	412	1.00E-78	249
TcMYB35	ATMYB101	39.51	405	233	9	106	1284	15	369	2.00E-73	234
TcMYB35	ATMYB120	60	190	57	3	64	576	9	188	6.00E-71	229
TcMYB35	AtMYB81	38.4	401	232	13	112	1269	19	367	4.00E-67	216
TcMYB35	AtMYB97	61.96	163	62	1	88	576	10	154	7.00E-67	214
TcMYB35	ATMYB102	34.19	351	218	11	115	1128	12	322	2.00E-50	169
TcMYB35	AtMYB74	51.33	150	70	2	115	555	13	162	4.00E-49	165
TcMYB35	AtMYB9	38.91	239	136	5	115	801	12	246	5.00E-49	165
TcMYB35	AtMYB107	66.35	104	35	0	115	426	12	115	6.00E-48	162
TcMYB35	ATMYB66	43.18	176	83	2	76	552	3	178	1.00E-47	157
TcMYB35	AtMYB17	64.42	104	37	0	115	426	12	115	1.00E-47	160
TcMYB35	AtMYB41	45.98	174	91	3	115	627	12	182	5.00E-47	158
TcMYB35	ATMYB3	63.46	104	38	0	115	426	12	115	6.00E-47	157
TcMYB35	ATMYB15	38.14	236	142	5	115	810	12	231	1.00E-46	157
TcMYB35	AtMYB6	65.69	102	35	0	121	426	14	115	1.00E-46	155
TcMYB35	MYB8	62.5	104	39	0	115	426	12	115	2.00E-46	154
TcMYB35	ATMYB5	50.74	136	63	3	115	510	23	155	5.00E-46	154
TcMYB35	ATMYB67	43.75	176	98	2	103	627	18	190	6.00E-46	156
TcMYB35	MYB7	63.46	104	38	0	115	426	12	115	6.00E-46	155
TcMYB35	ATMYB13	50.33	153	75	3	115	570	12	150	2.00E-45	153
TcMYB35	ATMYB57	48.59	142	73	1	109	534	23	142	2.00E-45	151
TcMYB35	AtMYB79	61.9	105	40	0	118	432	7	111	3.00E-45	153
TcMYB35	ATMYB63	47.37	152	75	1	115	555	14	165	3.00E-45	154
TcMYB35	ATMYB4	64.71	102	36	0	121	426	14	115	3.00E-45	153
TcMYB35	ATMYB14	48.05	154	80	1	115	576	12	164	5.00E-45	152
TcMYB35	ATMYB92	63.46	104	38	0	115	426	12	115	9.00E-45	154
TcMYB35	ATMYB58	42.16	185	97	4	115	639	14	182	1.00E-44	152
TcMYB35	ATMYB71	56.3	119	46	1	94	432	5	123	1.00E-44	151
TcMYB35	ATMYB121	47.71	153	80	1	118	576	28	178	1.00E-44	151
TcMYB35	AtMYB32	62.75	102	38	0	121	426	14	115	2.00E-44	151
TcMYB35	ATMYB3	41.42	169	95	2	94	588	13	181	3.00E-44	149
TcMYB35	AtMYB53	60.58	104	41	0	115	426	12	115	3.00E-44	151
TcMYB35	AtMYB49	61.54	104	40	0	115	426	12	115	8.00E-44	150
TcMYB35	ATMYB30	59.43	106	43	0	109	426	10	115	9.00E-44	150
TcMYB35	AtMYB51	42.08	202	115	5	115	714	13	194	9.00E-44	151
TcMYB35	ATMYB16	59.62	104	42	0	115	426	12	115	1.00E-43	150
TcMYB35	ATMYB106	60.58	104	41	0	115	426	55	158	1.00E-43	152
TcMYB35	AtMYB60	45.64	149	81	1	115	561	12	152	1.00E-43	149
TcMYB35	AtMYB93	61.54	104	40	0	115	426	12	115	1.00E-43	151
TcMYB35	ATMYB28	47.44	156	78	3	106	561	9	164	2.00E-43	150
TcMYB35	AtMYB24	52.94	119	50	1	85	423	1	119	4.00E-43	145
TcMYB35	ATMYB29	60.38	106	42	0	106	423	9	114	5.00E-43	149
TcMYB35	ATMYB86	53.54	127	58	2	115	492	12	135	5.00E-43	149
TcMYB35	ATMYB23	57.28	103	44	0	118	426	13	115	6.00E-43	145
TcMYB35	AtMYB20	57.94	107	45	0	115	435	12	118	6.00E-43	147
TcMYB35	AtMYB116	52.63	114	54	0	94	435	11	124	6.00E-43	147
TcMYB35	AtMYB50	60.58	104	41	0	115	426	12	115	7.00E-43	148
TcMYB35	AtMYB76	58.88	107	44	0	106	426	9	115	9.00E-43	148
TcMYB35	ATMYB34	58.88	107	44	0	115	435	12	118	1.00E-42	147
TcMYB35	ATMYB99	51.49	134	57	2	115	492	13	146	1.00E-42	145
TcMYB35	ATMYB96	47.52	141	74	1	115	537	12	142	1.00E-42	148
TcMYB35	ATMYB31	59.62	104	42	0	115	426	12	115	1.00E-42	147
TcMYB35	AtMYB103	43.53	170	94	3	115	618	12	174	2.00E-42	148
TcMYB35	ATMYB94	57.01	107	46	0	115	435	12	118	4.00E-42	146
TcMYB35	ATMYB46	42.11	171	99	2	115	627	18	174	4.00E-42	145
TcMYB35	AtMYB43	53.91	128	57	2	115	492	12	139	5.00E-42	146
TcMYB35	ATMYB111	57.69	104	44	0	115	426	12	115	5.00E-42	146
TcMYB35	ATMYB0	43.51	154	87	1	79	540	2	154	6.00E-42	143
TcMYB35	ATMYB87	58.49	106	43	1	112	426	11	116	6.00E-42	145

TcMYB35	AtMYB85	57.69	104	44	0	115	426	12	115	8.00E-42	144
TcMYB35	ATMYB80	36.62	213	126	5	112	723	11	221	1.00E-41	145
TcMYB35	ATMYB61	35.9	312	195	13	115	1035	12	291	1.00E-41	146
TcMYB35	ATMYB72	46.38	138	73	1	115	525	14	151	2.00E-41	144
TcMYB35	ATMYB123	55.77	104	46	0	115	426	14	117	2.00E-41	142
TcMYB35	AtMYB62	55.14	107	48	0	115	435	19	125	2.00E-41	143
TcMYB35	AtMYB82	52.14	117	53	1	103	444	8	124	2.00E-41	140
TcMYB35	AtMYB112	50	120	60	1	115	474	32	147	2.00E-41	142
TcMYB35	AtMYB42	57.69	104	44	0	115	426	12	115	3.00E-41	143
TcMYB35	ATMYB48	37.93	203	93	4	118	627	8	210	4.00E-41	141
TcMYB35	AtMYB108	51.67	120	58	1	115	474	19	134	6.00E-41	143
TcMYB35	ATMYB95	56.36	110	48	1	97	426	8	115	6.00E-41	141
TcMYB35	ATMYB122	45.12	164	82	3	115	582	12	175	1.00E-40	142
TcMYB35	AtMYB36	51.06	141	64	4	112	519	11	146	1.00E-40	142
TcMYB35	ATMYB12	55.77	104	46	0	115	426	12	115	1.00E-40	143
TcMYB35	ATMYB2	54.72	106	48	0	115	432	20	125	1.00E-40	140
TcMYB35	AtMYB83	44.37	151	75	2	115	540	30	180	1.00E-40	142
TcMYB35	ATMYB35	54.29	105	48	0	112	426	11	115	1.00E-40	142
TcMYB35	ATMYB55	55.08	118	41	1	115	432	12	129	1.00E-40	142
TcMYB35	AtMYB40	50.38	131	64	2	115	504	12	139	2.00E-40	140
TcMYB35	ATMYB59-3	53.98	113	51	1	118	453	9	121	2.00E-40	139
TcMYB35	AtMYB104	41.57	166	95	1	121	612	18	183	3.00E-40	142
TcMYB35	AtMYB10	56.73	104	45	0	115	426	14	117	4.00E-40	138
TcMYB35	ATMYB11	54.81	104	47	0	115	426	12	115	5.00E-40	141
TcMYB35	AtMYB47	40.57	175	102	3	97	615	8	180	6.00E-40	139
TcMYB35	ATMYB84	58.49	106	43	1	112	426	11	116	8.00E-40	139
TcMYB35	ATMYB68	57.55	106	44	1	112	426	11	116	3.00E-39	139
TcMYB35	ATMYB26	41.67	156	82	2	115	555	12	158	1.00E-38	137
TcMYB35	AtMYB114	49.04	104	53	0	115	426	8	111	3.00E-38	130
TcMYB35	ATMYB38	54.72	106	47	1	112	426	11	116	3.00E-38	135
TcMYB35	ATMYB37	53.33	105	48	1	115	426	12	116	2.00E-37	134
TcMYB35	AtMYB45	45.83	144	68	2	118	519	19	162	3.00E-37	131
TcMYB35	AtMYB19	46.62	133	71	0	118	516	13	145	1.00E-36	129
TcMYB35	ATMYB75	48.08	104	54	0	115	426	8	111	2.00E-36	128
TcMYB35	AtMYB27	52.83	106	50	0	112	429	8	113	2.00E-36	128
TcMYB35	ATMYB78	44.78	134	60	2	115	474	26	155	3.00E-36	130
TcMYB35	AtMYB113	48.08	104	54	0	115	426	8	111	8.00E-36	127
TcMYB35	ATMYB90	48.08	104	54	0	115	426	8	111	4.00E-35	125
TcMYB35	AtMYB18	50.49	103	51	0	118	426	11	113	8.00E-33	119
TcMYB35	AtMYB98	46.21	132	70	4	37	429	197	317	1.00E-31	119
TcMYB35	AtMYB70	52.94	102	48	1	121	426	13	113	8.00E-31	114
TcMYB35	ATMYB119	50.47	107	52	3	112	429	102	205	2.00E-30	116
TcMYB35	ATMYB118	42.86	140	79	4	112	528	186	312	2.00E-30	115
TcMYB35	ATMYB73	50.98	102	50	1	121	426	13	113	3.00E-30	113
TcMYB35	AtMYB1	53.92	102	47	1	121	426	55	155	6.00E-30	114
TcMYB35	AtMYB109	39.41	170	99	5	103	600	47	204	7.00E-30	114
TcMYB35	AtMYB115	50.5	101	50	1	121	423	158	257	7.00E-30	113
TcMYB35	AtMYB64	50	104	51	3	121	429	105	205	3.00E-29	112
TcMYB35	AtMYB100	34.72	193	113	5	79	618	17	203	1.00E-28	106
TcMYB35	ATMYB25	46.4	125	65	3	58	426	28	150	1.00E-28	109
TcMYB35	ATMYB77	46.73	107	57	1	121	441	6	111	5.00E-28	106
TcMYB35	ATMYB44	49.02	102	52	1	121	426	6	106	5.00E-28	106
TcMYB35	ATMYB105	37.35	166	90	4	94	549	98	259	8.00E-28	106
TcMYB35	ATMYB54	32.32	198	131	5	121	705	6	198	9.00E-28	104
TcMYB35	ATMYB52	46.96	115	60	2	121	462	5	117	1.00E-27	104
TcMYB35	AtMYB117	46.02	113	60	2	115	450	96	206	3.00E-27	105
TcMYB35	ATMYB110	34.64	179	117	3	31	567	41	201	3.00E-27	104
TcMYB35	AtMYB56	43.9	123	67	2	73	435	75	196	1.00E-26	103
TcMYB35	ATMYB69	44.44	108	60	1	112	435	16	122	6.00E-26	99.4
TcMYB35	AtMYB22	29.91	224	148	5	34	678	29	245	1.00E-24	95.9
TcMYB35	ATMYB91	40.38	104	60	1	130	435	7	110	2.00E-23	94.4
TcMYB35	AtMYB89	39	100	61	1	124	423	57	155	5.00E-19	77.8
TcMYB35	AtMYB124	34.65	101	66	1	130	432	28	127	3.00E-17	76.3
TcMYB35	ATMYB88	30.08	133	89	3	46	432	4	132	2.00E-15	70.9
TcMYB36	ATMYB65	38.36	451	262	13	13	1317	12	414	2.00E-79	251
TcMYB36	ATMYB33	42.96	398	214	14	43	1197	8	374	9.00E-79	248
TcMYB36	ATMYB101	40.25	395	224	9	106	1254	15	359	8.00E-74	234
TcMYB36	ATMYB120	60	190	57	3	64	576	9	188	2.00E-71	229
TcMYB36	AtMYB81	37.77	413	242	13	112	1305	19	379	4.00E-68	218
TcMYB36	AtMYB97	61.96	163	62	1	88	576	10	154	3.00E-67	214
TcMYB36	ATMYB102	34.19	351	218	11	115	1128	12	322	1.00E-50	169
TcMYB36	AtMYB74	51.33	150	70	2	115	555	13	162	2.00E-49	165
TcMYB36	AtMYB9	38.91	239	136	5	115	801	12	246	2.00E-49	165
TcMYB36	AtMYB107	66.35	104	35	0	115	426	12	115	3.00E-48	162
TcMYB36	ATMYB66	43.18	176	83	2	76	552	3	178	5.00E-48	157
TcMYB36	AtMYB17	64.42	104	37	0	115	426	12	115	7.00E-48	160
TcMYB36	AtMYB41	45.98	174	91	3	115	627	12	182	2.00E-47	158
TcMYB36	ATMYB3	63.46	104	38	0	115	426	12	115	3.00E-47	157
TcMYB36	ATMYB15	38.14	236	142	5	115	810	12	231	5.00E-47	157
TcMYB36	AtMYB6	65.69	102	35	0	121	426	14	115	7.00E-47	155

TcMYB36	MYB8	62.5	104	39	0	115	426	12	115	1.00E-46	154
TcMYB36	ATMYB5	50.74	136	63	3	115	510	23	155	3.00E-46	154
TcMYB36	ATMYB67	43.75	176	98	2	103	627	18	190	3.00E-46	156
TcMYB36	MYB7	63.46	104	38	0	115	426	12	115	3.00E-46	155
TcMYB36	ATMYB13	50.33	153	75	3	115	570	12	150	9.00E-46	153
TcMYB36	ATMYB57	48.59	142	73	1	109	534	23	142	1.00E-45	151
TcMYB36	AtMYB79	61.9	105	40	0	118	432	7	111	1.00E-45	153
TcMYB36	ATMYB63	47.37	152	75	1	115	555	14	165	2.00E-45	154
TcMYB36	ATMYB4	64.71	102	36	0	121	426	14	115	2.00E-45	153
TcMYB36	ATMYB14	48.05	154	80	1	115	576	12	164	3.00E-45	152
TcMYB36	ATMYB92	63.46	104	38	0	115	426	12	115	5.00E-45	154
TcMYB36	ATMYB58	42.16	185	97	4	115	639	14	182	5.00E-45	152
TcMYB36	ATMYB71	56.3	119	46	1	94	432	5	123	7.00E-45	151
TcMYB36	ATMYB121	47.71	153	80	1	118	576	28	178	8.00E-45	151
TcMYB36	AtMYB32	62.75	102	38	0	121	426	14	115	1.00E-44	151
TcMYB36	ATMYB3	41.42	169	95	2	94	588	13	181	1.00E-44	149
TcMYB36	AtMYB53	60.58	104	41	0	115	426	12	115	2.00E-44	151
TcMYB36	AtMYB49	61.54	104	40	0	115	426	12	115	4.00E-44	150
TcMYB36	ATMYB30	59.43	106	43	0	109	426	10	115	5.00E-44	150
TcMYB36	AtMYB51	42.08	202	115	5	115	714	13	194	5.00E-44	151
TcMYB36	ATMYB16	59.62	104	42	0	115	426	12	115	5.00E-44	150
TcMYB36	ATMYB106	60.58	104	41	0	115	426	55	158	6.00E-44	152
TcMYB36	AtMYB60	45.64	149	81	1	115	561	12	152	6.00E-44	149
TcMYB36	AtMYB93	61.54	104	40	0	115	426	12	115	7.00E-44	151
TcMYB36	ATMYB28	47.44	156	78	3	106	561	9	164	1.00E-43	150
TcMYB36	AtMYB24	52.94	119	50	1	85	423	1	119	2.00E-43	145
TcMYB36	ATMYB29	60.38	106	42	0	106	423	9	114	2.00E-43	149
TcMYB36	ATMYB86	53.54	127	58	2	115	492	12	135	3.00E-43	149
TcMYB36	ATMYB23	57.28	103	44	0	118	426	13	115	3.00E-43	145
TcMYB36	AtMYB20	57.94	107	45	0	115	435	12	118	3.00E-43	147
TcMYB36	AtMYB116	52.63	114	54	0	94	435	11	124	4.00E-43	147
TcMYB36	AtMYB50	60.58	104	41	0	115	426	12	115	4.00E-43	148
TcMYB36	AtMYB76	58.88	107	44	0	106	426	9	115	5.00E-43	148
TcMYB36	ATMYB34	58.88	107	44	0	115	435	12	118	7.00E-43	147
TcMYB36	ATMYB96	47.52	141	74	1	115	537	12	142	7.00E-43	148
TcMYB36	ATMYB99	51.49	134	57	2	115	492	13	146	7.00E-43	145
TcMYB36	ATMYB31	59.62	104	42	0	115	426	12	115	8.00E-43	147
TcMYB36	AtMYB103	43.53	170	94	3	115	618	12	174	1.00E-42	148
TcMYB36	ATMYB94	57.01	107	46	0	115	435	12	118	2.00E-42	146
TcMYB36	ATMYB46	42.11	171	99	2	115	627	18	174	2.00E-42	145
TcMYB36	AtMYB43	53.91	128	57	2	115	492	12	139	3.00E-42	146
TcMYB36	ATMYB111	57.69	104	44	0	115	426	12	115	3.00E-42	146
TcMYB36	ATMYB0	43.51	154	87	1	79	540	2	154	3.00E-42	143
TcMYB36	ATMYB87	58.49	106	43	1	112	426	11	116	3.00E-42	145
TcMYB36	AtMYB85	57.69	104	44	0	115	426	12	115	5.00E-42	144
TcMYB36	ATMYB80	36.62	213	126	5	112	723	11	221	6.00E-42	145
TcMYB36	ATMYB61	35.9	312	195	13	115	1035	12	291	7.00E-42	146
TcMYB36	ATMYB72	46.38	138	73	1	115	525	14	151	1.00E-41	144
TcMYB36	ATMYB123	55.77	104	46	0	115	426	14	117	1.00E-41	142
TcMYB36	AtMYB62	55.14	107	48	0	115	435	19	125	1.00E-41	143
TcMYB36	AtMYB82	52.14	117	53	1	103	444	8	124	1.00E-41	140
TcMYB36	AtMYB112	50	120	60	1	115	474	32	147	1.00E-41	142
TcMYB36	AtMYB42	57.69	104	44	0	115	426	12	115	1.00E-41	143
TcMYB36	ATMYB48	37.93	203	93	4	118	627	8	210	3.00E-41	141
TcMYB36	AtMYB108	51.67	120	58	1	115	474	19	134	3.00E-41	143
TcMYB36	ATMYB95	56.36	110	48	1	97	426	8	115	4.00E-41	141
TcMYB36	ATMYB122	45.12	164	82	3	115	582	12	175	6.00E-41	142
TcMYB36	AtMYB36	51.06	141	64	4	112	519	11	146	6.00E-41	142
TcMYB36	ATMYB12	55.77	104	46	0	115	426	12	115	7.00E-41	143
TcMYB36	ATMYB2	54.72	106	48	0	115	432	20	125	7.00E-41	140
TcMYB36	AtMYB83	44.37	151	75	2	115	540	30	180	8.00E-41	142
TcMYB36	ATMYB35	54.29	105	48	0	112	426	11	115	8.00E-41	142
TcMYB36	ATMYB55	55.08	118	41	1	115	432	12	129	8.00E-41	142
TcMYB36	AtMYB40	50.38	131	64	2	115	504	12	139	1.00E-40	140
TcMYB36	ATMYB59-3	53.98	113	51	1	118	453	9	121	1.00E-40	139
TcMYB36	AtMYB104	41.57	166	95	1	121	612	18	183	2.00E-40	142
TcMYB36	AtMYB10	56.73	104	45	0	115	426	14	117	2.00E-40	138
TcMYB36	ATMYB11	54.81	104	47	0	115	426	12	115	3.00E-40	141
TcMYB36	AtMYB47	40.57	175	102	3	97	615	8	180	3.00E-40	139
TcMYB36	ATMYB84	58.49	106	43	1	112	426	11	116	5.00E-40	139
TcMYB36	ATMYB68	57.55	106	44	1	112	426	11	116	2.00E-39	139
TcMYB36	ATMYB26	41.67	156	82	2	115	555	12	158	8.00E-39	137
TcMYB36	AtMYB114	49.04	104	53	0	115	426	8	111	2.00E-38	130
TcMYB36	ATMYB38	54.72	106	47	1	112	426	11	116	2.00E-38	135
TcMYB36	ATMYB37	53.33	105	48	1	115	426	12	116	1.00E-37	134
TcMYB36	AtMYB45	45.83	144	68	2	118	519	19	162	2.00E-37	131
TcMYB36	AtMYB19	46.62	133	71	0	118	516	13	145	9.00E-37	129
TcMYB36	ATMYB75	48.08	104	54	0	115	426	8	111	1.00E-36	128
TcMYB36	AtMYB27	52.83	106	50	0	112	429	8	113	1.00E-36	128
TcMYB36	ATMYB78	44.78	134	60	2	115	474	26	155	2.00E-36	130

TcMYB36	AtMYB113	48.08	104	54	0	115	426	8	111	5.00E-36	127
TcMYB36	ATMYB90	48.08	104	54	0	115	426	8	111	3.00E-35	125
TcMYB36	AtMYB18	50.49	103	51	0	118	426	11	113	5.00E-33	119
TcMYB36	AtMYB98	46.21	132	70	4	37	429	197	317	9.00E-32	119
TcMYB36	AtMYB70	52.94	102	48	1	121	426	13	113	6.00E-31	114
TcMYB36	ATMYB119	50.47	107	52	3	112	429	102	205	1.00E-30	116
TcMYB36	ATMYB118	42.86	140	79	4	112	528	186	312	2.00E-30	115
TcMYB36	ATMYB73	50.98	102	50	1	121	426	13	113	2.00E-30	113
TcMYB36	AtMYB1	53.92	102	47	1	121	426	55	155	4.00E-30	114
TcMYB36	AtMYB109	39.41	170	99	5	103	600	47	204	5.00E-30	114
TcMYB36	AtMYB115	50.5	101	50	1	121	423	158	257	5.00E-30	113
TcMYB36	AtMYB64	50	104	51	3	121	429	105	205	2.00E-29	112
TcMYB36	ATMYB25	46.4	125	65	3	58	426	28	150	9.00E-29	109
TcMYB36	AtMYB100	34.72	193	113	5	79	618	17	203	9.00E-29	106
TcMYB36	ATMYB77	46.73	107	57	1	121	441	6	111	4.00E-28	106
TcMYB36	ATMYB44	49.02	102	52	1	121	426	6	106	4.00E-28	106
TcMYB36	ATMYB105	37.35	166	90	4	94	549	98	259	6.00E-28	106
TcMYB36	ATMYB54	32.32	198	131	5	121	705	6	198	7.00E-28	104
TcMYB36	ATMYB52	46.96	115	60	2	121	462	5	117	8.00E-28	104
TcMYB36	AtMYB117	46.02	113	60	2	115	450	96	206	2.00E-27	105
TcMYB36	ATMYB110	34.64	179	117	3	31	567	41	201	2.00E-27	104
TcMYB36	AtMYB56	43.9	123	67	2	73	435	75	196	9.00E-27	103
TcMYB36	ATMYB69	44.44	108	60	1	112	435	16	122	5.00E-26	99.4
TcMYB36	AtMYB22	29.91	224	148	5	34	678	29	245	9.00E-25	95.9
TcMYB36	ATMYB91	40.38	104	60	1	130	435	7	110	2.00E-23	94.4
TcMYB36	AtMYB89	39	100	61	1	124	423	57	155	5.00E-19	77.8
TcMYB36	AtMYB124	34.65	101	66	1	130	432	28	127	3.00E-17	76.3
TcMYB36	ATMYB88	30.08	133	89	3	46	432	4	132	2.00E-15	70.9
TcMYB37	ATMYB77	44.1	229	115	5	4	651	16	211	3.00E-56	179
TcMYB37	ATMYB44	43.57	241	130	8	4	708	16	221	5.00E-54	173
TcMYB37	ATMYB73	43.57	241	128	5	13	711	26	223	2.00E-53	172
TcMYB37	AtMYB109	44.06	202	113	3	10	615	68	262	8.00E-51	167
TcMYB37	AtMYB70	79.31	87	18	0	16	276	27	113	7.00E-46	152
TcMYB37	AtMYB70	23.94	213	124	7	136	660	15	224	5.00E-08	46.2
TcMYB37	AtMYB1	41.46	205	119	4	10	621	67	261	9.00E-45	151
TcMYB37	ATMYB25	68.54	89	28	0	10	276	62	150	3.00E-41	141
TcMYB37	ATMYB54	46.61	118	58	1	16	354	20	137	3.00E-35	122
TcMYB37	ATMYB105	45.08	122	67	0	13	378	120	241	3.00E-34	122
TcMYB37	AtMYB117	47.41	116	58	1	13	351	111	226	7.00E-34	121
TcMYB37	ATMYB52	53.41	88	41	0	13	276	18	105	1.00E-33	118
TcMYB37	ATMYB69	54.55	88	40	0	13	276	32	119	6.00E-33	116
TcMYB37	AtMYB98	47.15	123	65	0	13	381	230	352	7.00E-33	120
TcMYB37	AtMYB56	55.68	88	39	0	13	276	106	193	2.00E-32	117
TcMYB37	ATMYB110	31.39	223	148	4	4	657	77	277	9.00E-32	114
TcMYB37	ATMYB48	36.77	155	94	5	19	471	24	172	6.00E-30	108
TcMYB37	ATMYB119	40.32	124	74	1	13	384	118	229	1.00E-28	108
TcMYB37	AtMYB64	44.04	109	61	1	13	339	118	222	4.00E-28	106
TcMYB37	AtMYB64	37.78	45	27	1	139	270	108	152	1.00E-04	35.8
TcMYB37	ATMYB118	47.75	111	57	2	13	342	202	311	5.00E-28	106
TcMYB37	AtMYB9	28.67	286	170	8	19	774	29	307	3.00E-27	103
TcMYB37	ATMYB59-3	45.63	103	54	2	19	321	25	126	4.00E-27	100
TcMYB37	AtMYB41	33.95	215	132	5	31	645	33	241	5.00E-27	101
TcMYB37	ATMYB28	37.66	154	79	4	73	483	48	200	7.00E-27	102
TcMYB37	ATMYB95	46.43	112	60	1	73	408	48	158	8.00E-27	100
TcMYB37	AtMYB97	48.15	108	48	2	19	318	36	143	1.00E-26	102
TcMYB37	AtMYB27	35.98	164	103	3	13	498	24	168	1.00E-26	99.4
TcMYB37	ATMYB67	46.43	112	57	3	31	357	43	153	1.00E-26	100
TcMYB37	ATMYB63	39.61	154	86	5	19	459	31	180	2.00E-26	99.8
TcMYB37	AtMYB115	49.43	87	44	0	13	273	171	257	3.00E-26	100
TcMYB37	AtMYB115	31.11	45	30	1	139	270	161	205	8.00E-04	33.1
TcMYB37	ATMYB35	40.77	130	74	3	31	411	33	162	3.00E-26	99.8
TcMYB37	ATMYB46	34.46	177	115	3	43	570	43	193	5.00E-26	98.6
TcMYB37	AtMYB100	51.81	83	39	1	28	273	43	125	7.00E-26	97.1
TcMYB37	ATMYB65	52.22	90	42	1	19	285	58	147	7.00E-26	101
TcMYB37	ATMYB101	50.54	93	45	1	10	285	32	124	7.00E-26	100
TcMYB37	AtMYB51	42.02	119	68	3	73	426	49	158	8.00E-26	99.4
TcMYB37	AtMYB36	44.86	107	56	2	40	351	37	143	8.00E-26	99
TcMYB37	ATMYB72	42.47	146	76	5	19	432	31	168	1.00E-25	97.8
TcMYB37	ATMYB26	49.45	91	46	0	73	345	57	147	2.00E-25	98.2
TcMYB37	ATMYB120	54.65	86	38	1	19	273	43	128	3.00E-25	99.4
TcMYB37	AtMYB89	43.68	87	49	0	13	273	69	155	3.00E-25	94
TcMYB37	ATMYB121	38.67	150	90	5	13	456	42	182	6.00E-25	95.5
TcMYB37	AtMYB47	42.02	119	65	2	73	417	48	165	7.00E-25	95.1
TcMYB37	ATMYB37	51.52	99	46	2	19	309	29	127	7.00E-25	96.3
TcMYB37	ATMYB33	51.11	90	43	1	19	285	49	138	7.00E-25	98.2
TcMYB37	AtMYB17	50.55	91	43	2	19	285	29	118	8.00E-25	95.5
TcMYB37	AtMYB83	43.22	118	67	2	73	426	66	175	2.00E-24	95.5
TcMYB37	ATMYB34	56.25	80	35	1	73	312	48	126	2.00E-24	94.4
TcMYB37	ATMYB57	38.03	142	85	3	43	459	50	191	3.00E-24	92
TcMYB37	AtMYB50	33.77	154	98	4	19	468	29	181	3.00E-24	94.4

TcMYB37	AtMYB10	34.36	195	124	6	25	597	33	210	4.00E-24	92.4
TcMYB37	ATMYB102	41.04	134	77	3	19	414	29	159	4.00E-24	94.4
TcMYB37	ATMYB29	35.77	137	88	2	73	483	48	182	5.00E-24	94
TcMYB37	ATMYB80	50.6	83	40	1	31	276	33	115	6.00E-24	93.6
TcMYB37	ATMYB4	46.6	103	54	1	73	378	48	150	6.00E-24	92.8
TcMYB37	ATMYB3	49.47	95	44	2	73	345	48	142	7.00E-24	92
TcMYB37	ATMYB92	48.89	90	44	2	19	282	29	117	9.00E-24	93.2
TcMYB37	ATMYB123	43.97	116	65	2	73	420	50	159	1.00E-23	91.7
TcMYB37	AtMYB103	42.5	120	69	1	73	432	48	163	1.00E-23	93.6
TcMYB37	ATMYB38	46	100	52	2	19	312	29	128	1.00E-23	92.4
TcMYB37	AtMYB74	49	100	49	2	31	324	34	133	1.00E-23	92.8
TcMYB37	ATMYB58	37.97	158	96	6	19	486	31	173	1.00E-23	91.7
TcMYB37	AtMYB6	37.4	131	76	2	73	447	48	178	2.00E-23	90.5
TcMYB37	MYB8	33.33	141	92	2	31	447	33	173	2.00E-23	89.7
TcMYB37	ATMYB66	37.98	129	61	3	19	348	33	160	2.00E-23	89.4
TcMYB37	AtMYB60	37.4	131	76	3	19	393	29	155	3.00E-23	90.9
TcMYB37	ATMYB14	35.07	134	85	2	31	426	33	165	3.00E-23	90.1
TcMYB37	AtMYB19	49.43	87	44	1	73	333	48	133	3.00E-23	90.5
TcMYB37	AtMYB79	47.62	84	42	2	31	276	27	109	4.00E-23	90.1
TcMYB37	ATMYB61	48.91	92	45	3	43	312	37	126	5.00E-23	91.7
TcMYB37	AtMYB76	55.71	70	31	0	73	282	48	117	5.00E-23	91.3
TcMYB37	ATMYB3	40	110	65	3	43	369	45	152	5.00E-23	89
TcMYB37	AtMYB82	50	88	42	2	19	276	29	115	6.00E-23	88.2
TcMYB37	ATMYB122	50	86	42	1	19	273	29	114	6.00E-23	90.9
TcMYB37	AtMYB53	48.89	90	44	2	19	282	29	117	6.00E-23	90.5
TcMYB37	MYB7	51.9	79	37	1	73	306	48	126	6.00E-23	89.7
TcMYB37	ATMYB106	43.86	114	61	3	19	351	72	185	8.00E-23	91.3
TcMYB37	ATMYB71	43.3	97	53	3	19	303	35	127	8.00E-23	89.4
TcMYB37	ATMYB86	46.15	104	54	3	43	348	37	138	1.00E-22	90.5
TcMYB37	AtMYB18	48.35	91	38	1	73	318	46	136	1.00E-22	89.4
TcMYB37	ATMYB13	40.74	135	75	4	31	420	33	151	1.00E-22	88.6
TcMYB37	AtMYB85	32.95	173	97	3	73	534	48	219	1.00E-22	89
TcMYB37	AtMYB32	46.81	94	49	1	73	351	48	141	1.00E-22	89
TcMYB37	AtMYB49	47.19	89	46	1	19	282	29	117	2.00E-22	89.4
TcMYB37	AtMYB81	48.84	86	43	1	19	273	37	122	2.00E-22	90.5
TcMYB37	ATMYB31	27.38	252	173	8	19	744	29	242	2.00E-22	89.4
TcMYB37	AtMYB42	51.9	79	37	1	73	306	48	126	3.00E-22	88.2
TcMYB37	ATMYB111	33.52	176	94	3	10	468	26	201	3.00E-22	89
TcMYB37	AtMYB107	46.07	89	47	1	19	282	29	117	3.00E-22	88.6
TcMYB37	AtMYB116	39.22	102	61	2	10	312	32	132	4.00E-22	87.8
TcMYB37	ATMYB23	48.81	84	41	2	31	276	33	115	6.00E-22	85.9
TcMYB37	ATMYB87	43.88	98	53	2	19	306	29	126	7.00E-22	87.4
TcMYB37	ATMYB16	49.43	87	43	1	19	276	29	115	9.00E-22	87.4
TcMYB37	AtMYB45	40.38	104	60	2	19	324	35	137	9.00E-22	86.3
TcMYB37	ATMYB0	38.98	118	71	2	31	381	35	148	9.00E-22	85.5
TcMYB37	ATMYB15	36.77	155	78	5	31	435	33	186	1.00E-21	86.7
TcMYB37	ATMYB94	30.99	171	113	5	19	516	29	180	1.00E-21	87.4
TcMYB37	AtMYB62	31.9	163	86	5	31	444	40	197	2.00E-21	85.9
TcMYB37	AtMYB24	31.45	159	95	4	43	477	42	199	2.00E-21	84.3
TcMYB37	ATMYB55	57.35	68	29	0	73	276	60	127	2.00E-21	86.7
TcMYB37	AtMYB22	45.12	82	45	0	28	273	71	152	2.00E-21	85.1
TcMYB37	ATMYB5	41.96	112	63	3	19	348	40	144	3.00E-21	84.7
TcMYB37	ATMYB30	39.2	125	65	4	19	360	29	148	3.00E-21	85.9
TcMYB37	ATMYB99	52.86	70	33	0	73	282	56	125	3.00E-21	84.3
TcMYB37	ATMYB68	48.78	82	41	1	40	282	37	118	4.00E-21	86.3
TcMYB37	AtMYB20	51.47	68	33	0	73	276	48	115	6.00E-21	84.3
TcMYB37	AtMYB40	38.68	106	62	3	43	351	37	141	6.00E-21	84
TcMYB37	AtMYB93	41.67	108	61	2	19	336	29	136	2.00E-20	84.3
TcMYB37	AtMYB112	30.87	149	79	3	31	405	53	200	2.00E-20	82.4
TcMYB37	ATMYB12	44.83	87	47	1	19	276	29	115	2.00E-20	84.3
TcMYB37	ATMYB84	46.34	82	43	1	40	282	37	118	2.00E-20	83.6
TcMYB37	ATMYB11	44.83	87	47	1	19	276	29	115	2.00E-20	84
TcMYB37	AtMYB104	45.98	87	45	2	19	273	33	118	2.00E-20	84.3
TcMYB37	AtMYB43	52.94	68	32	0	73	276	48	115	2.00E-20	83.6
TcMYB37	ATMYB96	32.81	128	81	3	19	387	29	140	6.00E-20	82.4
TcMYB37	AtMYB108	45.78	83	43	2	31	273	40	121	6.00E-20	82
TcMYB37	ATMYB2	45.24	84	44	2	31	276	41	123	2.00E-19	79.7
TcMYB37	AtMYB114	40	90	53	1	13	279	23	112	4.00E-19	75.9
TcMYB37	ATMYB88	31.37	153	98	3	31	468	49	199	2.00E-18	79
TcMYB37	ATMYB75	39.36	94	52	2	13	279	23	112	8.00E-18	74.7
TcMYB37	ATMYB90	31.33	150	102	2	19	465	25	163	2.00E-17	73.9
TcMYB37	AtMYB113	38.3	94	53	2	13	279	23	112	2.00E-17	73.6
TcMYB37	AtMYB124	37.8	82	51	0	31	276	44	125	3.00E-17	75.1
TcMYB37	ATMYB78	41.98	81	33	1	73	273	62	142	4.00E-16	70.9
TcMYB37	ATMYB91	37.23	94	56	1	19	291	19	112	1.00E-15	70.1
TcMYB38	AtMYB117	65.32	173	60	2	262	780	61	225	1.00E-73	229
TcMYB38	ATMYB105	60.53	190	64	4	196	732	30	219	1.00E-73	228
TcMYB38	AtMYB56	68.79	141	43	2	316	735	66	204	7.00E-64	202
TcMYB38	ATMYB110	55.88	170	72	2	298	798	36	201	2.00E-63	201
TcMYB38	ATMYB54	79.82	109	22	0	385	711	3	111	8.00E-63	197

TcMYB38	ATMYB52	77.98	109	24	0	385	711	2	110	1.00E-62	196
TcMYB38	ATMYB69	65.62	128	44	1	334	717	1	126	3.00E-57	182
TcMYB38	AtMYB89	61.68	107	41	0	397	717	57	163	5.00E-45	148
TcMYB38	ATMYB44	59.41	101	41	0	394	696	6	106	2.00E-40	140
TcMYB38	AtMYB109	58.65	104	43	0	394	705	56	159	5.00E-40	141
TcMYB38	ATMYB25	54.39	114	52	0	364	705	40	153	5.00E-39	137
TcMYB38	AtMYB70	57.43	101	43	0	394	696	13	113	2.00E-38	134
TcMYB38	ATMYB73	56.44	101	44	0	394	696	13	113	2.00E-38	134
TcMYB38	ATMYB77	56.44	101	44	0	394	696	6	106	6.00E-38	133
TcMYB38	AtMYB1	51.85	108	52	0	394	717	55	162	4.00E-36	130
TcMYB38	AtMYB115	37.78	180	107	5	385	909	155	325	4.00E-32	118
TcMYB38	ATMYB119	37.02	181	101	3	325	828	88	262	1.00E-31	118
TcMYB38	AtMYB98	37.65	162	101	2	274	759	183	338	7.00E-31	116
TcMYB38	AtMYB64	49.11	112	57	1	394	729	105	213	3.00E-30	114
TcMYB38	ATMYB15	30.09	216	141	4	370	987	6	213	6.00E-30	110
TcMYB38	ATMYB23	45.13	113	59	2	394	723	14	126	7.00E-30	108
TcMYB38	AtMYB103	39.15	189	113	6	370	930	7	182	7.00E-30	112
TcMYB38	ATMYB72	35.87	184	112	5	370	903	9	188	2.00E-29	109
TcMYB38	ATMYB67	49.55	111	54	3	370	696	17	125	2.00E-29	109
TcMYB38	ATMYB63	36.36	165	99	4	370	846	9	171	3.00E-29	108
TcMYB38	ATMYB66	47.06	102	53	1	394	696	18	119	4.00E-29	106
TcMYB38	ATMYB118	42.11	133	74	3	322	711	162	291	8.00E-29	110
TcMYB38	ATMYB0	44.64	112	60	2	394	723	16	127	1.00E-28	105
TcMYB38	AtMYB10	44.83	116	62	3	370	711	9	122	5.00E-28	104
TcMYB38	AtMYB45	35.57	149	91	3	394	825	20	168	6.00E-28	104
TcMYB38	AtMYB50	35.16	182	105	5	367	873	5	184	6.00E-28	105
TcMYB38	AtMYB18	44.44	108	59	1	394	714	12	119	6.00E-28	105
TcMYB38	AtMYB114	43.64	110	61	1	373	699	3	112	7.00E-28	100
TcMYB38	AtMYB100	46.46	99	52	2	400	693	28	125	8.00E-28	103
TcMYB38	AtMYB19	47.12	104	53	2	394	699	14	116	1.00E-27	103
TcMYB38	ATMYB35	34.67	199	123	7	370	945	7	194	2.00E-27	104
TcMYB38	ATMYB121	40.48	126	74	1	376	750	23	148	2.00E-27	103
TcMYB38	ATMYB101	28.57	315	211	11	394	1296	20	321	3.00E-27	106
TcMYB38	AtMYB82	47.06	102	53	1	394	696	14	115	4.00E-27	100
TcMYB38	AtMYB27	38.67	150	90	5	394	837	11	144	4.00E-27	101
TcMYB38	ATMYB86	35.54	166	105	4	367	858	5	159	5.00E-27	103
TcMYB38	AtMYB6	32.43	185	115	6	373	897	2	180	6.00E-27	101
TcMYB38	ATMYB80	43.64	110	61	1	370	696	6	115	6.00E-27	103
TcMYB38	AtMYB97	40.88	137	77	2	364	762	11	147	6.00E-27	104
TcMYB38	ATMYB61	26.91	327	227	8	367	1311	5	313	6.00E-27	103
TcMYB38	AtMYB51	46.08	102	54	1	394	696	15	116	7.00E-27	103
TcMYB38	AtMYB36	34.59	185	115	4	394	930	14	195	7.00E-27	103
TcMYB38	AtMYB9	28.79	264	176	8	373	1128	2	258	8.00E-27	103
TcMYB38	ATMYB58	44.55	110	60	2	370	696	9	117	9.00E-27	101
TcMYB38	AtMYB53	49.51	103	50	2	394	696	14	115	9.00E-27	102
TcMYB38	ATMYB75	43.64	110	61	1	373	699	3	112	1.00E-26	100
TcMYB38	ATMYB13	32.97	182	120	4	394	933	14	174	1.00E-26	100
TcMYB38	AtMYB41	44.35	115	57	3	373	696	2	115	1.00E-26	101
TcMYB38	ATMYB65	34.15	164	86	2	367	792	34	197	2.00E-26	104
TcMYB38	ATMYB122	45.19	104	56	1	370	678	6	109	3.00E-26	101
TcMYB38	MYB8	31.52	184	120	5	373	906	2	178	6.00E-26	97.8
TcMYB38	AtMYB104	41.23	114	66	2	364	702	8	118	7.00E-26	101
TcMYB38	AtMYB49	46.08	102	54	1	394	696	14	115	7.00E-26	100
TcMYB38	ATMYB34	40.65	123	72	1	370	735	6	128	9.00E-26	99.4
TcMYB38	ATMYB55	40	125	62	2	367	702	5	129	1.00E-25	100
TcMYB38	ATMYB120	30.69	202	130	4	394	969	28	228	1.00E-25	102
TcMYB38	ATMYB92	48.54	103	51	2	394	696	14	115	1.00E-25	99.8
TcMYB38	AtMYB40	39.85	133	75	3	394	777	14	145	1.00E-25	98.2
TcMYB38	AtMYB107	34.36	195	119	7	370	927	7	195	1.00E-25	99.4
TcMYB38	ATMYB90	39.67	121	72	1	373	732	3	123	2.00E-25	97.4
TcMYB38	ATMYB33	36.6	153	77	3	394	792	34	186	2.00E-25	101
TcMYB38	ATMYB71	38.93	131	76	3	394	774	20	149	2.00E-25	97.8
TcMYB38	ATMYB46	40.52	116	68	1	367	711	11	126	2.00E-25	97.8
TcMYB38	ATMYB26	40.32	124	64	2	367	708	5	128	3.00E-25	99.4
TcMYB38	AtMYB81	45.37	108	58	1	382	702	18	125	3.00E-25	100
TcMYB38	ATMYB3	41.23	114	61	2	373	696	2	115	4.00E-25	96.7
TcMYB38	AtMYB74	41.82	110	63	1	370	696	7	116	5.00E-25	97.8
TcMYB38	AtMYB76	34.24	184	118	3	394	936	14	196	6.00E-25	97.8
TcMYB38	ATMYB28	27.04	307	216	7	394	1290	14	300	6.00E-25	98.2
TcMYB38	ATMYB59-3	43.4	106	58	2	394	705	10	114	7.00E-25	95.5
TcMYB38	ATMYB48	44.34	106	57	2	394	705	9	113	7.00E-25	95.9
TcMYB38	ATMYB123	36.3	135	85	2	394	795	16	139	8.00E-25	95.9
TcMYB38	ATMYB4	35.57	149	89	4	373	798	2	144	9.00E-25	96.3
TcMYB38	ATMYB5	32.12	165	108	3	394	876	25	189	9.00E-25	95.5
TcMYB38	ATMYB37	33.51	188	122	6	370	924	7	191	1.00E-24	97.1
TcMYB38	ATMYB102	42.73	110	62	1	370	696	6	115	1.00E-24	97.1
TcMYB38	AtMYB79	38.57	140	68	3	394	759	8	146	2.00E-24	94.7
TcMYB38	ATMYB95	41.18	102	59	1	394	696	14	115	2.00E-24	94.7
TcMYB38	AtMYB93	42.98	114	59	2	373	696	2	115	3.00E-24	96.3
TcMYB38	ATMYB84	44.66	103	55	2	394	696	14	116	3.00E-24	95.1

TcMYB38	AtMYB32	41.23	114	61	2	373	696	2	115	4.00E-24	94.4
TcMYB38	ATMYB14	41.07	112	63	2	370	696	6	115	4.00E-24	93.6
TcMYB38	AtMYB108	28.45	232	153	7	316	972	2	216	4.00E-24	95.1
TcMYB38	ATMYB16	41.74	115	60	3	373	696	2	115	6.00E-24	94.7
TcMYB38	AtMYB113	40.95	105	61	1	394	705	10	114	7.00E-24	92.8
TcMYB38	ATMYB68	34.42	154	93	4	394	831	14	167	1.00E-23	94.7
TcMYB38	AtMYB43	40.8	125	72	2	394	762	14	137	1.00E-23	94
TcMYB38	AtMYB112	29.46	224	155	6	316	978	18	199	1.00E-23	92
TcMYB38	ATMYB2	46.32	95	50	1	394	675	22	116	2.00E-23	92.4
TcMYB38	ATMYB106	38.6	114	69	1	358	696	45	158	2.00E-23	94.4
TcMYB38	AtMYB62	38.89	126	75	2	334	705	1	125	2.00E-23	92.4
TcMYB38	AtMYB17	44.66	103	55	2	394	696	14	115	3.00E-23	92.4
TcMYB38	ATMYB87	25.33	300	202	9	370	1203	6	295	3.00E-23	92.4
TcMYB38	AtMYB83	41.67	108	62	1	394	714	32	139	3.00E-23	92.8
TcMYB38	MYB7	38.6	114	64	2	373	696	2	115	4.00E-23	91.3
TcMYB38	AtMYB60	40.71	113	66	1	394	729	14	126	5.00E-23	91.3
TcMYB38	AtMYB47	38.24	102	62	1	394	696	14	115	5.00E-23	90.9
TcMYB38	ATMYB96	29.89	184	127	2	394	939	14	197	7.00E-23	92
TcMYB38	AtMYB22	36.36	132	84	2	298	693	27	152	8.00E-23	90.1
TcMYB38	ATMYB38	43.69	103	56	2	394	696	14	116	8.00E-23	90.9
TcMYB38	AtMYB116	27.86	201	139	4	394	978	20	206	9.00E-23	90.5
TcMYB38	ATMYB94	41.9	105	60	1	394	705	14	118	1.00E-22	91.3
TcMYB38	ATMYB29	46.88	96	50	1	394	678	14	109	1.00E-22	91.3
TcMYB38	AtMYB20	28.38	222	127	5	394	963	14	231	1.00E-22	90.1
TcMYB38	AtMYB42	31.44	194	130	6	370	942	6	184	2.00E-22	89.7
TcMYB38	ATMYB3	28.37	215	147	5	394	1017	22	226	2.00E-22	88.2
TcMYB38	ATMYB88	33.57	140	91	1	316	729	2	141	3.00E-22	91.3
TcMYB38	AtMYB85	37.25	153	94	5	394	846	14	158	4.00E-22	88.2
TcMYB38	ATMYB30	42.16	102	58	1	394	696	14	115	6.00E-22	89
TcMYB38	ATMYB57	40.74	108	62	2	394	711	27	133	9.00E-22	85.9
TcMYB38	ATMYB31	42.16	102	58	1	394	696	14	115	1.00E-21	88.2
TcMYB38	AtMYB24	41.05	95	55	1	394	675	19	113	4.00E-21	84.3
TcMYB38	AtMYB124	37.86	103	64	0	403	711	28	130	4.00E-21	87.8
TcMYB38	ATMYB111	40.2	102	60	1	394	696	14	115	4.00E-21	86.7
TcMYB38	ATMYB91	34.15	123	78	1	394	753	4	126	5.00E-21	86.7
TcMYB38	ATMYB12	39.22	102	61	1	394	696	14	115	2.00E-20	84.7
TcMYB38	ATMYB11	39.22	102	61	1	394	696	14	115	3.00E-20	84.3
TcMYB38	ATMYB99	36.76	136	77	4	394	774	15	144	6.00E-19	78.6
TcMYB38	ATMYB78	31.43	140	81	2	394	768	28	167	7.00E-19	79.7
TcMYB39	ATMYB31	47.56	328	157	8	1	939	1	308	8.00E-97	283
TcMYB39	ATMYB94	45.24	347	162	7	1	957	1	320	1.00E-96	283
TcMYB39	ATMYB96	46.11	360	153	10	1	957	1	341	6.00E-94	276
TcMYB39	ATMYB30	47.34	338	154	8	1	942	1	307	8.00E-93	273
TcMYB39	AtMYB60	44.51	319	173	5	1	945	1	261	3.00E-87	257
TcMYB39	ATMYB16	45.98	224	105	6	1	624	1	223	1.00E-63	198
TcMYB39	ATMYB106	41.95	267	155	5	10	810	47	300	7.00E-61	192
TcMYB39	AtMYB17	38.14	291	151	5	1	786	1	287	2.00E-60	189
TcMYB39	ATMYB14	66.1	118	40	0	1	354	1	118	1.00E-59	185
TcMYB39	AtMYB107	64.66	116	41	0	1	348	1	116	2.00E-58	184
TcMYB39	ATMYB13	44.13	213	114	1	1	624	1	213	4.00E-58	181
TcMYB39	AtMYB41	67.24	116	38	0	1	348	1	116	4.00E-58	182
TcMYB39	AtMYB9	33.24	343	203	5	1	951	1	330	7.00E-58	183
TcMYB39	AtMYB32	38.02	263	155	3	1	765	1	262	5.00E-57	179
TcMYB39	AtMYB74	45.73	199	105	3	1	588	1	192	5.00E-57	181
TcMYB39	ATMYB15	61.24	129	48	1	1	381	1	129	1.00E-56	179
TcMYB39	ATMYB72	48.84	172	88	2	4	519	4	168	2.00E-55	176
TcMYB39	ATMYB12	40.87	208	111	3	1	588	1	208	2.00E-55	178
TcMYB39	AtMYB93	63.79	116	42	0	1	348	1	116	2.00E-55	177
TcMYB39	ATMYB102	62.18	119	45	0	1	357	1	119	9.00E-55	176
TcMYB39	AtMYB49	36.69	278	168	3	1	810	1	277	1.00E-54	174
TcMYB39	ATMYB3	62.07	116	44	0	1	348	1	116	4.00E-54	171
TcMYB39	ATMYB34	63.56	118	43	0	1	354	1	118	9.00E-54	171
TcMYB39	AtMYB43	40.08	247	135	5	1	702	1	237	1.00E-53	172
TcMYB39	MYB8	62.07	116	44	0	1	348	1	116	1.00E-53	168
TcMYB39	AtMYB6	62.93	116	43	0	1	348	1	116	1.00E-53	169
TcMYB39	MYB7	37.64	263	149	9	1	744	1	248	2.00E-53	170
TcMYB39	ATMYB80	62.93	116	43	0	1	348	1	116	3.00E-53	171
TcMYB39	ATMYB28	37.46	283	176	6	1	846	1	256	3.00E-53	172
TcMYB39	ATMYB92	62.07	116	44	0	1	348	1	116	4.00E-53	171
TcMYB39	ATMYB63	36.31	314	196	7	4	933	4	291	5.00E-53	169
TcMYB39	AtMYB20	59.66	119	48	0	1	357	1	119	5.00E-53	169
TcMYB39	ATMYB35	32.29	319	196	7	1	897	1	281	2.00E-52	169
TcMYB39	ATMYB11	59.06	127	51	1	1	378	1	127	2.00E-52	169
TcMYB39	ATMYB87	59.69	129	51	1	1	384	1	129	4.00E-52	167
TcMYB39	ATMYB4	61.21	116	45	0	1	348	1	116	4.00E-52	167
TcMYB39	AtMYB53	61.21	116	45	0	1	348	1	116	6.00E-52	167
TcMYB39	AtMYB51	63.87	119	42	1	1	354	1	119	6.00E-52	168
TcMYB39	AtMYB36	52.56	156	67	3	1	447	1	156	1.00E-51	167
TcMYB39	ATMYB38	58.09	136	56	1	1	405	1	136	2.00E-51	165
TcMYB39	ATMYB111	59.48	116	47	0	1	348	1	116	4.00E-51	166

TcMYB39	ATMYB68	50.94	159	72	3	1	459	1	156	1.00E-50	166
TcMYB39	ATMYB5	59.29	113	46	0	10	348	15	127	1.00E-50	162
TcMYB39	AtMYB85	58.82	119	49	0	1	357	1	119	2.00E-50	162
TcMYB39	AtMYB42	58.33	120	50	0	1	360	1	120	2.00E-50	162
TcMYB39	AtMYB10	55.64	133	59	0	7	405	5	137	2.00E-50	161
TcMYB39	ATMYB37	58.46	130	53	1	1	387	1	130	2.00E-50	164
TcMYB39	ATMYB29	61.21	116	45	0	1	348	1	116	2.00E-50	164
TcMYB39	ATMYB122	41.07	224	107	5	1	597	1	223	4.00E-50	163
TcMYB39	AtMYB76	39.69	257	137	5	1	717	1	247	5.00E-50	163
TcMYB39	ATMYB84	62.39	117	43	1	1	348	1	117	1.00E-49	161
TcMYB39	ATMYB58	59.13	115	47	0	4	348	4	118	1.00E-49	160
TcMYB39	ATMYB95	61.86	118	45	0	1	354	1	118	2.00E-49	159
TcMYB39	ATMYB86	34.22	301	192	6	1	885	1	276	4.00E-49	161
TcMYB39	ATMYB101	34.57	269	162	6	31	795	17	277	6.00E-49	164
TcMYB39	AtMYB40	38.37	245	147	5	1	723	1	230	8.00E-49	157
TcMYB39	AtMYB103	58.47	118	49	0	1	354	1	118	2.00E-48	160
TcMYB39	ATMYB99	56	125	48	1	4	357	3	127	8.00E-48	154
TcMYB39	AtMYB47	59.32	118	48	0	1	354	1	118	1.00E-47	155
TcMYB39	ATMYB71	33.9	295	193	10	31	909	17	268	1.00E-47	155
TcMYB39	AtMYB50	36	275	168	7	1	801	1	263	1.00E-46	153
TcMYB39	ATMYB61	57.63	118	50	0	1	354	1	118	3.00E-46	154
TcMYB39	ATMYB120	32.14	252	171	2	34	789	26	274	3.00E-45	154
TcMYB39	ATMYB67	36.09	266	159	7	16	780	16	239	5.00E-45	149
TcMYB39	ATMYB26	52	125	51	1	1	348	1	125	9.00E-45	150
TcMYB39	ATMYB66	57.69	104	44	0	37	348	17	120	1.00E-44	145
TcMYB39	ATMYB121	41.62	185	108	2	31	585	26	185	3.00E-44	146
TcMYB39	ATMYB23	57.69	104	44	0	37	348	13	116	4.00E-44	144
TcMYB39	AtMYB82	47.89	142	59	2	34	414	12	149	5.00E-44	143
TcMYB39	ATMYB0	44.67	150	82	2	37	483	15	158	1.00E-43	143
TcMYB39	ATMYB3	53.85	117	54	0	4	354	10	126	2.00E-43	142
TcMYB39	AtMYB79	46.63	163	82	2	37	510	7	169	2.00E-43	143
TcMYB39	AtMYB81	41.75	194	111	6	25	600	17	189	6.00E-43	146
TcMYB39	AtMYB113	40.3	201	119	6	1	600	1	181	6.00E-43	142
TcMYB39	ATMYB123	55.45	110	49	0	34	363	14	123	8.00E-43	142
TcMYB39	AtMYB114	47.69	130	68	1	31	420	7	132	9.00E-43	138
TcMYB39	ATMYB33	56.48	108	47	0	22	345	28	135	9.00E-43	147
TcMYB39	AtMYB24	41.28	172	92	3	34	522	17	185	1.00E-42	140
TcMYB39	ATMYB65	56.73	104	45	0	34	345	41	144	1.00E-42	147
TcMYB39	ATMYB46	39	200	120	3	34	627	18	214	1.00E-42	142
TcMYB39	AtMYB97	57.14	105	45	0	28	342	17	121	2.00E-42	144
TcMYB39	ATMYB57	40.44	183	102	3	34	561	25	197	3.00E-42	139
TcMYB39	AtMYB83	44.44	153	79	1	22	462	26	178	5.00E-42	142
TcMYB39	ATMYB2	53.91	115	53	0	34	378	20	134	7.00E-42	140
TcMYB39	AtMYB108	38.62	189	114	2	22	582	15	195	2.00E-41	140
TcMYB39	AtMYB116	50.42	119	59	0	37	393	19	137	3.00E-41	138
TcMYB39	ATMYB48	50.89	112	55	0	31	366	6	117	3.00E-41	137
TcMYB39	ATMYB59-3	51.82	110	53	0	37	366	9	118	4.00E-41	137
TcMYB39	AtMYB112	43.31	157	89	1	25	495	29	177	5.00E-41	137
TcMYB39	AtMYB62	38.03	213	127	6	34	657	19	209	7.00E-41	137
TcMYB39	ATMYB55	50.39	129	52	1	1	351	1	129	7.00E-41	139
TcMYB39	ATMYB75	54.29	105	48	0	31	345	7	111	1.00E-39	133
TcMYB39	ATMYB90	47.69	130	68	1	31	420	7	133	2.00E-39	133
TcMYB39	AtMYB45	45.04	131	64	1	37	405	19	149	4.00E-39	132
TcMYB39	AtMYB19	41.38	174	99	2	37	549	13	180	5.00E-39	132
TcMYB39	AtMYB18	35.06	251	160	5	37	780	11	254	4.00E-37	127
TcMYB39	ATMYB78	41.36	162	81	2	22	465	22	175	5.00E-37	128
TcMYB39	AtMYB27	50.96	104	51	0	34	345	9	112	4.00E-33	115
TcMYB39	AtMYB104	42.61	115	66	1	40	384	18	131	6.00E-30	110
TcMYB39	AtMYB70	50	102	51	1	40	345	13	113	8.00E-30	108
TcMYB39	ATMYB73	47.06	102	54	1	40	345	13	113	2.00E-28	105
TcMYB39	AtMYB109	47.17	106	56	1	40	357	56	160	5.00E-28	105
TcMYB39	ATMYB25	45.1	102	56	1	40	345	50	150	6.00E-28	104
TcMYB39	AtMYB1	49.02	102	52	1	40	345	55	155	6.00E-28	105
TcMYB39	ATMYB77	46.08	102	55	1	40	345	6	106	1.00E-26	100
TcMYB39	ATMYB44	45.1	102	56	1	40	345	6	106	6.00E-26	98.2
TcMYB39	ATMYB118	43.93	107	60	1	25	345	184	289	1.00E-25	99.4
TcMYB39	ATMYB118	34.48	58	37	1	184	354	184	241	7.00E-07	42.7
TcMYB39	ATMYB119	30.09	226	156	5	40	711	105	319	1.00E-25	99
TcMYB39	ATMYB105	39.13	115	70	1	25	369	102	215	4.00E-25	96.3
TcMYB39	AtMYB115	39.09	110	67	2	34	363	156	263	9.00E-25	95.9
TcMYB39	AtMYB98	40.95	105	62	1	40	354	217	320	9.00E-25	96.7
TcMYB39	AtMYB117	33.56	149	93	2	40	468	98	245	1.00E-24	95.5
TcMYB39	ATMYB54	39.45	109	66	1	40	366	6	113	1.00E-24	93.2
TcMYB39	ATMYB69	39.09	110	67	1	25	354	14	122	3.00E-24	92.4
TcMYB39	ATMYB52	42.16	102	59	1	40	345	5	105	8.00E-24	91.3
TcMYB39	AtMYB56	39.5	119	72	2	4	360	82	198	1.00E-23	92.4
TcMYB39	AtMYB64	38.1	105	65	1	40	354	105	208	3.00E-23	92.4
TcMYB39	ATMYB91	38.46	104	62	1	49	354	7	110	6.00E-23	90.9
TcMYB39	AtMYB124	39.6	101	61	1	49	351	28	127	3.00E-21	86.7
TcMYB39	ATMYB110	34.96	123	80	2	40	408	67	184	3.00E-21	85.1

TcMYB39	ATMYB88	39.6	101	61	1	49	351	33	132	6.00E-21	85.9
TcMYB39	AtMYB89	36.54	104	66	1	43	354	57	159	1.00E-20	81.3
TcMYB39	AtMYB100	39.45	109	66	2	25	351	21	127	5.00E-20	80.5
TcMYB39	AtMYB22	23.36	214	152	6	25	630	49	255	3.00E-19	78.6
TcMYB40	ATMYB15	50.8	250	107	7	1	702	1	234	5.00E-76	228
TcMYB40	AtMYB17	44.17	283	130	7	1	765	1	283	1.00E-75	227
TcMYB40	AtMYB9	39.32	351	171	9	1	927	1	331	1.00E-74	226
TcMYB40	AtMYB107	76.98	126	29	0	1	378	1	126	7.00E-74	223
TcMYB40	ATMYB14	82.76	116	20	0	1	348	1	116	9.00E-73	218
TcMYB40	ATMYB102	43.04	309	162	8	1	885	1	280	1.00E-72	221
TcMYB40	AtMYB85	50.23	221	91	3	1	606	1	221	9.00E-72	216
TcMYB40	ATMYB16	74.6	126	32	0	1	378	1	126	5.00E-71	216
TcMYB40	AtMYB41	74.8	127	32	0	1	381	1	127	8.00E-71	214
TcMYB40	AtMYB74	43.05	295	155	7	1	846	1	283	1.00E-70	215
TcMYB40	AtMYB93	74.6	126	32	0	1	378	1	126	2.00E-70	216
TcMYB40	ATMYB13	47.2	250	130	6	1	744	1	227	1.00E-69	210
TcMYB40	ATMYB58	45.28	254	132	5	4	744	4	232	6.00E-69	209
TcMYB40	ATMYB92	50	212	106	2	1	636	1	208	2.00E-68	210
TcMYB40	ATMYB5	72.13	122	34	0	13	378	16	137	3.00E-68	206
TcMYB40	ATMYB106	73.98	123	32	0	10	378	47	169	8.00E-68	210
TcMYB40	AtMYB53	73.02	126	34	0	1	378	1	126	1.00E-67	207
TcMYB40	AtMYB42	54.97	191	78	2	1	549	1	180	1.00E-67	206
TcMYB40	AtMYB43	74.6	126	32	0	1	378	1	126	4.00E-67	206
TcMYB40	AtMYB20	73.02	126	34	0	1	378	1	126	1.00E-66	203
TcMYB40	ATMYB3	58.23	158	66	0	1	474	1	158	4.00E-66	201
TcMYB40	MYB7	53.85	169	77	1	1	504	1	169	5.00E-66	201
TcMYB40	ATMYB72	69.12	136	39	1	4	402	4	139	2.00E-65	201
TcMYB40	AtMYB6	68.25	126	40	0	1	378	1	126	2.00E-65	199
TcMYB40	MYB8	66.67	126	42	0	1	378	1	126	3.00E-65	197
TcMYB40	AtMYB32	63.01	146	54	0	1	438	1	146	3.00E-65	199
TcMYB40	AtMYB10	65.94	138	42	1	7	405	5	142	1.00E-64	197
TcMYB40	ATMYB4	66.67	126	42	0	1	378	1	126	5.00E-64	197
TcMYB40	ATMYB63	76.52	115	27	0	4	348	4	118	2.00E-63	196
TcMYB40	AtMYB49	51.46	206	92	5	1	594	1	201	2.00E-63	196
TcMYB40	AtMYB40	64.93	134	47	0	1	402	1	134	3.00E-62	191
TcMYB40	ATMYB122	47.27	220	97	3	1	603	1	218	4.00E-62	193
TcMYB40	ATMYB28	48.11	212	110	3	1	636	1	199	5.00E-62	194
TcMYB40	AtMYB51	66.93	127	41	1	1	378	1	127	1.00E-61	192
TcMYB40	ATMYB12	43.72	247	132	7	1	720	1	231	3.00E-61	192
TcMYB40	ATMYB34	66.67	126	42	0	1	378	1	126	5.00E-61	189
TcMYB40	ATMYB111	70.69	116	34	0	1	348	1	116	6.00E-61	191
TcMYB40	ATMYB35	56.33	158	68	3	1	471	1	151	2.00E-60	189
TcMYB40	ATMYB80	61.15	139	54	1	1	417	1	136	2.00E-60	189
TcMYB40	ATMYB29	37.13	334	171	9	1	885	1	334	2.00E-60	189
TcMYB40	AtMYB60	67.21	122	40	0	1	366	1	122	4.00E-60	187
TcMYB40	ATMYB11	69.83	116	35	0	1	348	1	116	7.00E-60	188
TcMYB40	ATMYB99	62.88	132	42	1	4	378	3	134	1.00E-59	184
TcMYB40	ATMYB61	66.67	126	42	0	1	378	1	126	4.00E-59	187
TcMYB40	ATMYB94	67.23	119	39	0	1	357	1	119	2.00E-58	184
TcMYB40	ATMYB30	67.21	122	40	0	1	366	1	122	3.00E-58	183
TcMYB40	AtMYB76	48.99	198	101	3	1	594	1	188	3.00E-58	184
TcMYB40	ATMYB86	66.67	126	42	0	1	378	1	126	3.00E-58	184
TcMYB40	ATMYB31	67.23	119	39	0	1	357	1	119	9.00E-58	182
TcMYB40	ATMYB96	65.55	119	41	0	1	357	1	119	3.00E-57	181
TcMYB40	AtMYB50	62.7	126	47	0	1	378	1	126	4.00E-57	180
TcMYB40	ATMYB95	50.89	169	83	0	1	507	1	169	7.00E-57	178
TcMYB40	AtMYB47	37.69	268	162	5	1	789	1	246	1.00E-54	172
TcMYB40	ATMYB67	48.77	162	83	1	16	501	16	176	2.00E-54	172
TcMYB40	ATMYB55	59.42	138	44	1	1	378	1	138	7.00E-54	172
TcMYB40	ATMYB37	42.92	212	120	2	1	633	1	211	3.00E-53	170
TcMYB40	ATMYB87	61.86	118	44	1	1	351	1	118	2.00E-52	167
TcMYB40	ATMYB84	63.56	118	42	1	1	351	1	118	1.00E-51	166
TcMYB40	ATMYB38	53.64	151	61	2	1	426	1	151	3.00E-51	164
TcMYB40	ATMYB68	64.41	118	41	1	1	351	1	118	3.00E-51	166
TcMYB40	AtMYB103	62.07	116	44	0	1	348	1	116	2.00E-50	164
TcMYB40	AtMYB36	63.56	118	42	1	1	351	1	118	2.00E-50	163
TcMYB40	ATMYB23	53.24	139	62	1	37	444	13	151	7.00E-50	158
TcMYB40	ATMYB123	70.48	105	31	0	34	348	14	118	7.00E-50	159
TcMYB40	ATMYB26	57.94	126	44	1	1	351	1	126	2.00E-49	162
TcMYB40	ATMYB101	46.2	184	99	4	31	582	17	190	2.00E-49	164
TcMYB40	ATMYB66	62.5	104	39	0	37	348	17	120	1.00E-48	154
TcMYB40	AtMYB83	64.22	109	39	0	22	348	26	134	4.00E-48	157
TcMYB40	ATMYB46	42.51	207	117	4	34	648	18	203	6.00E-48	155
TcMYB40	AtMYB82	61.68	107	41	0	25	345	9	115	2.00E-47	151
TcMYB40	AtMYB24	57.38	122	52	1	34	399	17	135	8.00E-47	150
TcMYB40	ATMYB120	61.82	110	42	1	34	363	26	134	3.00E-46	156
TcMYB40	ATMYB0	59.05	105	43	0	37	351	15	119	5.00E-46	149
TcMYB40	ATMYB3	50.32	155	76	3	34	495	20	169	6.00E-46	148
TcMYB40	AtMYB114	60	105	42	0	31	345	7	111	1.00E-45	145
TcMYB40	ATMYB71	60.95	105	41	0	31	345	17	121	1.00E-45	149

TcMYB40	AtMYB79	62.14	103	39	0	37	345	7	109	2.00E-45	148
TcMYB40	ATMYB33	59.26	108	44	0	22	345	28	135	4.00E-45	153
TcMYB40	AtMYB81	52.67	131	58	2	25	405	17	146	5.00E-45	151
TcMYB40	AtMYB116	55.26	114	51	0	37	378	19	132	2.00E-44	146
TcMYB40	AtMYB62	35.48	279	177	8	34	861	19	279	2.00E-44	146
TcMYB40	AtMYB108	58.26	115	48	0	22	366	15	129	6.00E-44	146
TcMYB40	ATMYB75	59.05	105	43	0	31	345	7	111	7.00E-44	144
TcMYB40	ATMYB121	59.43	106	43	0	25	342	24	129	1.00E-43	144
TcMYB40	ATMYB65	59.62	104	42	0	34	345	41	144	1.00E-43	150
TcMYB40	AtMYB19	44.75	181	97	1	25	558	9	189	2.00E-43	143
TcMYB40	AtMYB97	57.27	110	47	1	34	363	19	127	2.00E-43	146
TcMYB40	ATMYB90	59.05	105	43	0	31	345	7	111	2.00E-43	142
TcMYB40	ATMYB2	37.62	202	115	3	34	606	20	220	3.00E-43	143
TcMYB40	ATMYB57	58.1	105	44	0	34	348	25	129	1.00E-42	139
TcMYB40	AtMYB113	54.78	115	52	1	1	345	1	111	2.00E-42	140
TcMYB40	AtMYB112	55.75	113	48	1	34	366	32	144	7.00E-42	138
TcMYB40	ATMYB48	55.24	105	47	0	31	345	6	110	1.00E-41	138
TcMYB40	ATMYB59-3	55.34	103	46	0	37	345	9	111	3.00E-41	136
TcMYB40	AtMYB18	52.85	123	58	0	37	405	11	133	2.00E-40	135
TcMYB40	AtMYB27	35.46	251	161	6	34	783	9	235	3.00E-40	134
TcMYB40	AtMYB45	54.9	102	46	0	37	342	19	120	3.00E-38	129
TcMYB40	ATMYB78	30.32	310	198	8	22	897	22	308	1.00E-37	129
TcMYB40	AtMYB104	38.78	147	84	3	25	447	13	158	8.00E-31	112
TcMYB40	ATMYB77	39.38	160	97	4	40	519	6	160	2.00E-29	107
TcMYB40	ATMYB73	48.04	102	53	1	40	345	13	113	6.00E-29	106
TcMYB40	AtMYB1	47.22	108	53	2	40	351	55	157	6.00E-29	107
TcMYB40	AtMYB1	36.21	58	36	1	184	354	50	107	3.00E-05	37.4
TcMYB40	AtMYB70	47.06	102	54	1	40	345	13	113	9.00E-29	105
TcMYB40	AtMYB70	32.73	55	36	1	199	360	13	67	4.00E-04	33.9
TcMYB40	AtMYB98	45.79	107	58	1	25	345	212	317	4.00E-28	105
TcMYB40	AtMYB115	44.66	103	57	2	40	348	158	258	7.00E-28	103
TcMYB40	ATMYB44	33.16	193	94	4	40	513	6	197	1.00E-27	102
TcMYB40	ATMYB25	48.54	103	52	2	40	345	50	150	2.00E-27	103
TcMYB40	ATMYB25	32.08	53	35	1	199	354	50	102	9.00E-04	32.7
TcMYB40	AtMYB109	47.57	103	53	2	40	345	56	156	4.00E-27	102
TcMYB40	ATMYB118	41.13	124	71	2	25	390	184	306	4.00E-27	102
TcMYB40	ATMYB119	43.27	104	59	1	34	345	103	205	8.00E-27	102
TcMYB40	ATMYB119	29.09	55	38	1	199	360	105	159	1.00E-04	35.8
TcMYB40	ATMYB52	45.28	106	58	1	28	345	1	105	6.00E-26	96.7
TcMYB40	ATMYB52	32.08	53	35	1	199	354	5	57	5.00E-04	33.1
TcMYB40	ATMYB54	47.57	103	53	2	40	345	6	106	7.00E-26	96.3
TcMYB40	ATMYB54	33.96	53	34	1	199	354	6	58	9.00E-05	35.4
TcMYB40	AtMYB64	41.18	102	60	1	40	345	105	205	4.00E-25	97.1
TcMYB40	ATMYB69	38.46	143	81	3	25	432	14	154	4.00E-24	91.7
TcMYB40	ATMYB105	34.78	161	88	3	25	456	102	261	2.00E-23	91.3
TcMYB40	AtMYB117	43.27	104	59	1	34	345	96	198	9.00E-23	89.7
TcMYB40	AtMYB100	31	200	133	7	25	609	21	205	3.00E-22	86.3
TcMYB40	ATMYB110	40.78	103	61	1	40	348	67	168	2.00E-21	85.1
TcMYB40	AtMYB56	41.18	102	60	1	40	345	93	193	4.00E-21	84.7
TcMYB40	ATMYB88	42.42	99	57	1	49	345	33	130	3.00E-20	83.2
TcMYB40	ATMYB91	35.24	105	66	1	37	345	3	107	2.00E-19	80.1
TcMYB40	AtMYB124	41.41	99	58	1	49	345	28	125	2.00E-19	80.5
TcMYB40	AtMYB89	40.38	104	62	2	43	354	57	158	3.00E-19	77
TcMYB40	AtMYB22	35.29	102	66	2	25	330	49	148	4.00E-17	72.4
TcMYB41	ATMYB59-3	59.68	62	24	1	25	207	53	114	7.00E-23	84.7
TcMYB41	ATMYB48	59.68	62	24	1	25	207	52	113	1.00E-22	84.7
TcMYB41	AtMYB108	52.56	78	37	0	40	273	70	147	2.00E-22	85.1
TcMYB41	AtMYB62	43.43	99	53	2	34	321	68	166	9.00E-22	82.8
TcMYB41	ATMYB2	56.34	71	31	1	40	252	71	137	3.00E-21	81.3
TcMYB41	AtMYB116	38.24	102	63	0	34	339	67	168	4.00E-21	80.9
TcMYB41	AtMYB27	56.67	60	26	0	40	219	60	119	8.00E-21	79.3
TcMYB41	AtMYB79	52	75	35	1	25	246	51	125	8.00E-21	79.7
TcMYB41	ATMYB3	57.89	57	24	0	40	210	71	127	9.00E-21	79
TcMYB41	ATMYB71	47.78	90	44	2	25	285	63	152	9.00E-21	79.7
TcMYB41	AtMYB24	57.63	59	25	0	40	216	68	126	1.00E-20	78.6
TcMYB41	AtMYB112	46.15	78	42	0	40	273	83	160	2.00E-20	78.6
TcMYB41	ATMYB3	66.67	54	18	0	40	201	63	116	4.00E-20	77.8
TcMYB41	MYB7	60	60	23	1	25	201	57	116	5.00E-20	77.8
TcMYB41	ATMYB57	55.93	59	26	0	40	216	76	134	5.00E-20	76.6
TcMYB41	ATMYB121	54.41	68	30	1	25	225	72	139	5.00E-20	77.8
TcMYB41	AtMYB32	61.67	60	22	1	25	201	57	116	7.00E-20	77.4
TcMYB41	ATMYB4	60	60	23	1	25	201	57	116	8.00E-20	77.4
TcMYB41	MYB8	60	60	23	1	25	201	57	116	1.00E-19	75.9
TcMYB41	AtMYB6	58.33	60	24	1	25	201	57	116	2.00E-19	75.9
TcMYB41	ATMYB63	53.12	64	29	1	25	213	59	122	2.00E-19	76.6
TcMYB41	AtMYB10	53.12	64	29	1	25	213	59	122	4.00E-19	74.7
TcMYB41	ATMYB66	50.75	67	29	1	34	222	65	131	9.00E-19	73.2
TcMYB41	ATMYB33	64.15	53	19	0	40	198	83	135	1.00E-18	75.5
TcMYB41	ATMYB78	43.48	92	38	1	40	273	77	168	2.00E-18	74.3
TcMYB41	AtMYB97	63.46	52	19	0	40	195	70	121	2.00E-18	74.7

TcMYB41	AtMYB85	55.36	56	25	0	40	207	63	118	2.00E-18	73.6
TcMYB41	ATMYB86	54.84	62	27	1	25	207	57	118	3.00E-18	73.9
TcMYB41	ATMYB120	57.14	56	24	0	40	207	77	132	3.00E-18	74.3
TcMYB41	ATMYB12	67.31	52	17	0	46	201	65	116	4.00E-18	73.6
TcMYB41	AtMYB9	40.54	111	64	3	40	366	63	167	4.00E-18	73.2
TcMYB41	ATMYB111	67.31	52	17	0	46	201	65	116	5.00E-18	73.2
TcMYB41	AtMYB83	52.24	67	31	1	25	222	75	141	5.00E-18	73.2
TcMYB41	ATMYB99	59.26	54	22	0	40	201	71	124	5.00E-18	72
TcMYB41	ATMYB14	53.23	62	28	1	25	207	57	118	5.00E-18	72
TcMYB41	ATMYB61	56.45	62	26	1	25	207	57	118	5.00E-18	73.2
TcMYB41	ATMYB30	55.93	59	26	0	40	216	63	121	6.00E-18	72.8
TcMYB41	ATMYB96	57.63	59	25	0	40	216	63	121	7.00E-18	72.8
TcMYB41	ATMYB58	47.76	67	34	1	25	222	59	125	7.00E-18	72
TcMYB41	ATMYB65	58.49	53	22	0	40	198	92	144	7.00E-18	73.2
TcMYB41	AtMYB50	56.45	62	26	1	25	207	57	118	7.00E-18	72.4
TcMYB41	ATMYB46	50.77	65	31	1	25	216	63	127	7.00E-18	72
TcMYB41	ATMYB15	56.14	57	25	0	34	204	61	117	8.00E-18	72
TcMYB41	AtMYB60	36.63	101	64	0	40	342	63	163	1.00E-17	71.6
TcMYB41	ATMYB101	56.6	53	23	0	40	198	69	121	1.00E-17	72.4
TcMYB41	AtMYB20	55.36	56	25	0	40	207	63	118	1.00E-17	71.2
TcMYB41	AtMYB107	59.26	54	22	0	40	201	63	116	1.00E-17	71.6
TcMYB41	ATMYB94	54.24	59	27	0	40	216	63	121	2.00E-17	71.6
TcMYB41	ATMYB23	40.4	99	51	2	34	306	61	156	2.00E-17	70.1
TcMYB41	ATMYB13	49.25	67	33	1	25	222	57	123	2.00E-17	70.5
TcMYB41	AtMYB42	57.14	56	24	0	40	207	63	118	2.00E-17	70.9
TcMYB41	AtMYB19	53.97	63	28	1	25	210	57	119	2.00E-17	70.5
TcMYB41	ATMYB80	57.41	54	23	0	40	201	63	116	3.00E-17	70.9
TcMYB41	ATMYB123	55.36	56	25	0	40	207	65	120	3.00E-17	70.1
TcMYB41	ATMYB5	59.26	54	22	0	40	201	74	127	4.00E-17	69.7
TcMYB41	AtMYB74	55.36	56	25	0	40	207	64	119	4.00E-17	70.5
TcMYB41	ATMYB11	63.46	52	19	0	46	201	65	116	4.00E-17	70.5
TcMYB41	ATMYB55	54.1	61	27	1	25	204	69	129	4.00E-17	70.5
TcMYB41	ATMYB0	51.79	56	27	0	34	201	63	118	5.00E-17	68.9
TcMYB41	AtMYB43	50	62	30	1	25	207	57	118	7.00E-17	69.7
TcMYB41	AtMYB81	55.17	58	25	1	25	195	65	122	7.00E-17	70.1
TcMYB41	ATMYB31	57.41	54	23	0	40	201	63	116	1.00E-16	69.3
TcMYB41	ATMYB102	55.56	54	24	0	40	201	63	116	1.00E-16	69.3
TcMYB41	AtMYB103	51.61	62	29	1	25	207	57	118	1.00E-16	69.3
TcMYB41	AtMYB53	31.91	141	94	3	40	456	63	197	1.00E-16	68.9
TcMYB41	AtMYB40	52.46	61	28	1	25	204	57	117	2.00E-16	68.2
TcMYB41	ATMYB35	51.79	56	27	0	40	207	63	118	2.00E-16	68.2
TcMYB41	AtMYB82	54.72	53	24	0	40	198	63	115	3.00E-16	66.6
TcMYB41	AtMYB41	50.88	57	28	0	40	210	63	119	3.00E-16	67.8
TcMYB41	AtMYB51	53.57	56	26	0	40	207	64	119	3.00E-16	68.2
TcMYB41	ATMYB26	50	60	29	1	25	201	66	125	3.00E-16	68.2
TcMYB41	AtMYB18	52.38	63	29	1	25	210	55	117	4.00E-16	67.4
TcMYB41	ATMYB25	54.55	55	25	0	34	198	96	150	4.00E-16	67.8
TcMYB41	ATMYB92	55.56	54	24	0	40	201	63	116	7.00E-16	67
TcMYB41	ATMYB72	44.78	67	36	1	25	222	59	125	7.00E-16	66.6
TcMYB41	AtMYB17	53.7	54	25	0	40	201	63	116	8.00E-16	66.6
TcMYB41	AtMYB45	50	60	29	1	25	201	63	122	8.00E-16	66.2
TcMYB41	ATMYB87	57.41	54	23	0	40	201	64	117	8.00E-16	66.6
TcMYB41	ATMYB106	55.36	56	25	0	40	207	106	161	8.00E-16	67
TcMYB41	ATMYB28	50	58	29	0	34	207	61	118	1.00E-15	66.6
TcMYB41	AtMYB49	45.07	71	37	1	40	246	63	133	1.00E-15	66.2
TcMYB41	ATMYB16	53.57	56	26	0	40	207	63	118	2.00E-15	65.9
TcMYB41	ATMYB84	55.56	54	24	0	40	201	64	117	2.00E-15	65.5
TcMYB41	AtMYB76	50	56	28	0	34	201	61	116	3.00E-15	65.1
TcMYB41	ATMYB67	43.33	60	33	1	25	201	67	126	4.00E-15	64.7
TcMYB41	AtMYB93	55.56	54	24	0	40	201	63	116	5.00E-15	64.7
TcMYB41	ATMYB68	53.7	54	25	0	40	201	64	117	7.00E-15	64.3
TcMYB41	AtMYB36	51.85	54	26	0	40	201	64	117	8.00E-15	63.9
TcMYB41	ATMYB29	53.7	54	25	0	34	195	61	114	8.00E-15	63.9
TcMYB41	ATMYB37	48.28	58	30	0	40	213	64	121	1.00E-14	63.2
TcMYB41	AtMYB114	49.09	55	28	0	34	198	57	111	2.00E-14	60.5
TcMYB41	AtMYB113	34.29	105	59	1	34	318	57	161	3.00E-14	61.6
TcMYB41	ATMYB34	48.21	56	29	0	40	207	63	118	3.00E-14	62
TcMYB41	AtMYB98	36.11	72	46	0	40	255	265	336	5.00E-14	62
TcMYB41	AtMYB98	38.78	49	29	1	64	207	221	269	1.00E-06	40.4
TcMYB41	ATMYB90	49.09	55	28	0	34	198	57	111	6.00E-14	60.8
TcMYB41	ATMYB38	50	54	27	0	40	201	64	117	6.00E-14	61.2
TcMYB41	ATMYB122	33.33	90	39	1	1	207	29	118	7.00E-14	61.2
TcMYB41	ATMYB75	49.09	55	28	0	34	198	57	111	1.00E-13	60.1
TcMYB41	ATMYB95	44.64	56	31	0	40	207	63	118	3.00E-13	59.3
TcMYB41	AtMYB100	36.99	73	46	1	40	258	74	145	3.00E-13	58.9
TcMYB41	AtMYB100	24.56	57	42	1	46	213	24	80	4.00E-04	32.3
TcMYB41	AtMYB104	48.15	54	28	0	34	195	65	118	3.00E-13	59.7
TcMYB41	ATMYB118	38.33	60	37	0	40	219	237	296	1.00E-12	57.8
TcMYB41	ATMYB105	37.5	64	40	0	34	225	153	216	3.00E-12	56.6
TcMYB41	AtMYB47	37.93	58	36	0	34	207	61	118	3.00E-12	56.2

TcMYB41	AtMYB109	45.45	55	30	0	34	198	102	156	4.00E-12	56.2
TcMYB41	AtMYB117	37.5	64	40	0	34	225	144	207	5.00E-12	55.8
TcMYB41	AtMYB70	40.32	62	37	0	34	219	59	120	9.00E-12	55.1
TcMYB41	ATMYB73	38.46	65	40	1	34	228	59	121	9.00E-12	55.1
TcMYB41	ATMYB119	41.07	56	33	0	40	207	153	208	1.00E-11	54.7
TcMYB41	ATMYB54	37.88	66	41	0	34	231	52	117	2.00E-11	53.9
TcMYB41	AtMYB64	41.07	56	33	0	40	207	153	208	2.00E-11	54.3
TcMYB41	ATMYB77	51.16	43	21	0	70	198	64	106	3.00E-11	53.5
TcMYB41	AtMYB115	38.46	52	32	0	40	195	206	257	3.00E-11	53.5
TcMYB41	AtMYB89	35.82	67	43	0	13	213	95	161	5.00E-11	52
TcMYB41	AtMYB1	45.28	53	29	0	40	198	103	155	7.00E-11	52.8
TcMYB41	ATMYB44	40	55	33	0	34	198	52	106	8.00E-11	52.4
TcMYB41	ATMYB91	31.25	64	44	0	34	225	53	116	2.00E-10	51.6
TcMYB41	AtMYB22	36.21	58	37	1	40	213	101	157	2.00E-10	51.2
TcMYB41	ATMYB110	40.68	59	35	1	37	213	115	172	2.00E-10	51.2
TcMYB41	ATMYB52	39.34	61	37	0	34	216	51	111	2.00E-10	50.8
TcMYB41	AtMYB56	30.3	66	46	0	34	231	139	204	5.00E-10	50.1
TcMYB41	ATMYB69	36.36	55	35	0	34	198	65	119	8.00E-09	46.2
TcMYB41	AtMYB124	44	50	28	0	49	198	76	125	8.00E-09	46.6
TcMYB41	ATMYB88	44	50	28	0	49	198	81	130	9.00E-09	46.6
TcMYB42	ATMYB123	66.67	111	32	1	28	345	43	153	6.00E-47	149
TcMYB42	ATMYB5	73.86	88	22	1	28	288	52	139	9.00E-45	143
TcMYB42	AtMYB6	61.11	108	34	1	28	327	41	148	1.00E-44	142
TcMYB42	ATMYB4	53.33	135	57	2	28	414	41	175	6.00E-44	142
TcMYB42	ATMYB66	47.4	154	78	2	31	483	46	199	2.00E-43	138
TcMYB42	ATMYB12	61.82	110	42	0	28	357	41	150	2.00E-43	142
TcMYB42	ATMYB3	78.95	76	16	0	28	255	41	116	3.00E-43	139
TcMYB42	AtMYB114	60.78	102	40	1	28	333	37	136	6.00E-43	135
TcMYB42	MYB7	77.22	79	18	0	28	264	41	119	1.00E-42	138
TcMYB42	MYB8	72.15	79	22	0	28	264	41	119	6.00E-42	134
TcMYB42	ATMYB111	80.26	76	15	0	28	255	41	116	7.00E-42	138
TcMYB42	AtMYB32	74.68	79	20	0	28	264	41	119	1.00E-41	135
TcMYB42	ATMYB75	43.9	164	89	3	28	510	37	197	7.00E-41	133
TcMYB42	ATMYB23	76	75	18	0	31	255	42	116	9.00E-41	132
TcMYB42	AtMYB107	55.93	118	46	1	28	363	41	158	1.00E-40	134
TcMYB42	AtMYB82	59.26	108	40	2	31	342	42	148	2.00E-40	130
TcMYB42	AtMYB49	70.79	89	20	1	28	276	41	129	3.00E-40	133
TcMYB42	ATMYB13	57.52	113	48	1	28	366	41	147	5.00E-40	130
TcMYB42	AtMYB9	57.52	113	42	1	28	348	41	153	5.00E-40	133
TcMYB42	ATMYB0	73.33	75	20	0	31	255	44	118	6.00E-40	130
TcMYB42	ATMYB90	60.78	102	40	1	28	333	37	137	8.00E-40	130
TcMYB42	ATMYB11	76.32	76	18	0	28	255	41	116	2.00E-39	131
TcMYB42	AtMYB85	38.84	224	112	8	28	624	41	264	3.00E-39	129
TcMYB42	AtMYB113	67.9	81	26	0	28	270	37	117	5.00E-39	128
TcMYB42	AtMYB53	75	76	19	0	28	255	41	116	6.00E-39	129
TcMYB42	AtMYB74	73.68	76	20	0	28	255	42	117	1.00E-38	129
TcMYB42	AtMYB42	65.22	92	32	0	28	303	41	132	1.00E-38	128
TcMYB42	ATMYB15	65.12	86	30	0	28	285	41	126	2.00E-38	127
TcMYB42	ATMYB86	60.36	111	37	2	28	339	41	151	2.00E-38	129
TcMYB42	ATMYB80	65.48	84	29	0	4	255	33	116	2.00E-38	128
TcMYB42	ATMYB102	75	76	19	0	28	255	41	116	3.00E-38	129
TcMYB42	ATMYB58	68.35	79	25	0	28	264	43	121	4.00E-38	126
TcMYB42	ATMYB84	70	80	24	0	28	267	42	121	5.00E-38	127
TcMYB42	AtMYB41	64.44	90	31	1	28	294	41	130	5.00E-38	126
TcMYB42	AtMYB17	73.68	76	20	0	28	255	41	116	7.00E-38	126
TcMYB42	ATMYB92	73.68	76	20	0	28	255	41	116	8.00E-38	127
TcMYB42	ATMYB61	48.89	135	65	1	28	420	41	175	1.00E-37	127
TcMYB42	ATMYB46	72	75	21	0	31	255	48	122	2.00E-37	125
TcMYB42	ATMYB35	65.48	84	29	0	4	255	33	116	2.00E-37	125
TcMYB42	ATMYB14	72.37	76	21	0	28	255	41	116	3.00E-37	124
TcMYB42	AtMYB50	69.62	79	24	0	28	264	41	119	3.00E-37	125
TcMYB42	ATMYB34	69.62	79	24	0	28	264	41	119	5.00E-37	124
TcMYB42	AtMYB51	72.15	79	22	0	28	264	42	120	6.00E-37	125
TcMYB42	ATMYB16	63.44	93	28	1	28	288	41	133	7.00E-37	124
TcMYB42	ATMYB106	73.68	76	20	0	28	255	84	159	1.00E-36	125
TcMYB42	ATMYB68	67.5	80	26	0	28	267	42	121	1.00E-36	125
TcMYB42	AtMYB83	70.13	77	23	0	31	261	60	136	2.00E-36	124
TcMYB42	ATMYB72	64.04	89	31	1	28	291	43	131	3.00E-36	122
TcMYB42	AtMYB103	69.74	76	23	0	28	255	41	116	3.00E-36	124
TcMYB42	ATMYB99	71.05	76	22	0	28	255	49	124	4.00E-36	120
TcMYB42	ATMYB63	68.42	76	24	0	28	255	43	118	4.00E-36	122
TcMYB42	ATMYB37	62.07	87	33	0	25	285	41	127	4.00E-36	122
TcMYB42	AtMYB10	61.63	86	33	0	28	285	43	128	4.00E-36	120
TcMYB42	AtMYB36	59.55	89	36	0	28	294	42	130	6.00E-36	122
TcMYB42	ATMYB29	69.62	79	24	0	28	264	41	119	9.00E-36	122
TcMYB42	AtMYB76	69.74	76	23	0	28	255	41	116	9.00E-36	122
TcMYB42	ATMYB67	64.47	76	27	0	28	255	51	126	1.00E-35	120
TcMYB42	ATMYB28	69.74	76	23	0	28	255	41	116	2.00E-35	122
TcMYB42	AtMYB47	51.89	106	51	0	4	321	33	138	2.00E-35	119
TcMYB42	ATMYB57	69.33	75	23	0	31	255	55	129	2.00E-35	117

TcMYB42	ATMYB95	52.78	108	50	1	4	324	33	140	3.00E-35	119
TcMYB42	AtMYB93	71.05	76	22	0	28	255	41	116	3.00E-35	121
TcMYB42	ATMYB122	68.35	79	25	0	28	264	41	119	3.00E-35	120
TcMYB42	ATMYB2	63.75	80	29	0	31	270	50	129	4.00E-35	119
TcMYB42	AtMYB43	69.74	76	23	0	28	255	41	116	4.00E-35	120
TcMYB42	ATMYB55	75	72	18	0	40	255	57	128	4.00E-35	120
TcMYB42	AtMYB20	69.74	76	23	0	28	255	41	116	5.00E-35	119
TcMYB42	ATMYB87	62.5	80	30	0	28	267	42	121	7.00E-35	119
TcMYB42	AtMYB60	39.46	185	109	5	28	573	41	212	5.00E-34	116
TcMYB42	ATMYB3	60	85	34	0	31	285	50	134	5.00E-34	114
TcMYB42	AtMYB40	64.1	78	28	0	22	255	39	116	6.00E-34	115
TcMYB42	AtMYB108	40.85	164	97	2	37	528	51	203	2.00E-33	115
TcMYB42	ATMYB120	68	75	24	0	31	255	56	130	3.00E-33	117
TcMYB42	AtMYB24	67.12	73	24	0	31	249	47	119	4.00E-33	112
TcMYB42	AtMYB112	63.75	80	29	0	31	270	62	141	6.00E-33	112
TcMYB42	ATMYB38	59.76	82	33	0	28	273	42	123	7.00E-33	113
TcMYB42	AtMYB116	64.94	77	27	0	22	252	45	121	7.00E-33	113
TcMYB42	AtMYB62	37	200	124	6	31	624	49	238	8.00E-33	113
TcMYB42	AtMYB79	68.92	74	23	0	31	252	36	109	9.00E-33	112
TcMYB42	ATMYB26	61.18	85	24	1	28	255	41	125	2.00E-32	114
TcMYB42	ATMYB94	65.79	76	26	0	28	255	41	116	2.00E-32	113
TcMYB42	ATMYB59-3	59.04	83	34	0	31	279	38	120	2.00E-32	110
TcMYB42	ATMYB31	67.11	76	25	0	28	255	41	116	3.00E-32	112
TcMYB42	ATMYB101	64.86	74	26	0	31	252	48	121	3.00E-32	115
TcMYB42	AtMYB19	62.79	86	32	1	28	285	41	125	3.00E-32	111
TcMYB42	ATMYB30	65.79	76	26	0	28	255	41	116	3.00E-32	112
TcMYB42	AtMYB18	63.86	83	30	0	28	276	39	121	4.00E-32	111
TcMYB42	ATMYB71	69.44	72	22	0	37	252	50	121	4.00E-32	110
TcMYB42	ATMYB48	56.32	87	38	0	19	279	33	119	4.00E-32	110
TcMYB42	ATMYB96	65.79	76	26	0	28	255	41	116	5.00E-32	112
TcMYB42	AtMYB97	65.75	73	25	0	31	249	49	121	6.00E-32	112
TcMYB42	AtMYB27	66.67	72	24	0	37	252	41	112	1.00E-31	108
TcMYB42	AtMYB81	66.2	71	24	0	37	249	52	122	4.00E-31	111
TcMYB42	ATMYB33	63.51	74	27	0	31	252	62	135	6.00E-31	111
TcMYB42	ATMYB121	60.92	87	32	1	31	285	57	143	7.00E-31	107
TcMYB42	ATMYB65	60.81	74	29	0	31	252	71	144	3.00E-30	109
TcMYB42	AtMYB45	54.12	85	39	0	28	282	47	131	3.00E-29	103
TcMYB42	ATMYB78	55.43	92	27	1	37	270	58	149	9.00E-29	103
TcMYB42	AtMYB104	38.52	135	81	4	28	426	45	166	5.00E-25	94
TcMYB42	AtMYB98	50	80	40	1	49	288	250	328	2.00E-23	90.1
TcMYB42	AtMYB98	29.85	67	46	1	46	243	197	263	9.00E-05	35
TcMYB42	ATMYB105	30.11	176	114	5	49	549	140	315	1.00E-22	86.7
TcMYB42	ATMYB25	58.82	68	28	0	49	252	83	150	1.00E-22	87
TcMYB42	ATMYB54	48.68	76	39	0	49	276	39	114	1.00E-22	85.1
TcMYB42	ATMYB52	52.94	68	32	0	49	252	38	105	6.00E-22	83.6
TcMYB42	AtMYB117	52.94	68	32	0	49	252	131	198	6.00E-22	85.1
TcMYB42	AtMYB100	30.22	182	127	5	49	594	59	219	6.00E-22	83.2
TcMYB42	ATMYB44	54.41	68	31	0	49	252	39	106	1.00E-21	83.6
TcMYB42	ATMYB73	54.41	68	31	0	49	252	46	113	2.00E-21	83.6
TcMYB42	ATMYB73	30.3	66	45	1	106	300	13	78	6.00E-05	35.4
TcMYB42	AtMYB115	49.25	67	34	0	49	249	191	257	2.00E-21	83.6
TcMYB42	AtMYB70	52.94	68	32	0	49	252	46	113	4.00E-21	82.4
TcMYB42	AtMYB1	52.94	68	32	0	49	252	88	155	4.00E-21	83.2
TcMYB42	AtMYB1	33.93	56	36	1	91	255	50	105	2.00E-04	33.9
TcMYB42	ATMYB118	51.47	68	33	0	49	252	222	289	5.00E-21	83.2
TcMYB42	ATMYB118	30.77	52	35	1	91	243	184	235	0.001	32
TcMYB42	AtMYB109	55.88	68	30	0	49	252	89	156	5.00E-21	82.8
TcMYB42	AtMYB109	37.04	54	33	1	106	264	56	109	9.00E-05	35
TcMYB42	AtMYB64	45.57	79	43	0	49	285	138	216	8.00E-21	82.4
TcMYB42	AtMYB64	32	50	33	1	97	243	102	151	5.00E-04	32.7
TcMYB42	ATMYB119	50	68	34	0	49	252	138	205	2.00E-20	81.3
TcMYB42	ATMYB119	34.04	47	30	1	106	243	105	151	2.00E-04	34.3
TcMYB42	ATMYB110	49.28	69	35	0	49	255	100	168	3.00E-20	79.7
TcMYB42	AtMYB56	42.68	82	47	0	43	288	124	205	4.00E-20	79.7
TcMYB42	ATMYB69	48.53	68	35	0	49	252	52	119	6.00E-20	78.2
TcMYB42	ATMYB77	50	68	34	0	49	252	39	106	8.00E-20	78.6
TcMYB42	AtMYB22	41.67	72	42	1	49	264	86	156	4.00E-18	73.2
TcMYB42	AtMYB89	39.24	79	48	0	49	285	89	167	8.00E-17	68.6
TcMYB42	ATMYB91	46.03	63	34	0	43	231	38	100	2.00E-16	69.3
TcMYB42	ATMYB88	44.62	65	36	0	58	252	66	130	9.00E-15	65.1
TcMYB42	AtMYB124	43.08	65	37	0	58	252	61	125	3.00E-14	63.5
TcMYB43	ATMYB105	78.63	131	28	1	295	687	90	219	6.00E-70	218
TcMYB43	AtMYB117	70.55	146	41	1	256	687	65	210	3.00E-69	217
TcMYB43	AtMYB56	59.51	163	66	0	277	765	69	231	5.00E-66	207
TcMYB43	ATMYB52	76.32	114	27	0	340	681	2	115	3.00E-63	198
TcMYB43	ATMYB54	65.97	144	49	1	340	771	3	144	3.00E-63	197
TcMYB43	ATMYB110	66.67	135	45	1	280	684	45	178	6.00E-61	194
TcMYB43	ATMYB69	73.58	106	28	0	343	660	17	122	2.00E-55	177
TcMYB43	AtMYB89	52.14	140	67	1	247	666	33	161	8.00E-47	153
TcMYB43	AtMYB109	45.52	145	79	2	235	669	40	162	2.00E-39	139

TcMYB43	ATMYB25	55.24	105	47	0	349	663	50	154	2.00E-39	138
TcMYB43	ATMYB44	55.45	101	45	0	349	651	6	106	3.00E-38	134
TcMYB43	AtMYB70	55.45	101	45	0	349	651	13	113	3.00E-37	131
TcMYB43	AtMYB1	47.06	136	72	2	247	654	24	156	9.00E-37	132
TcMYB43	ATMYB73	52.48	101	48	0	349	651	13	113	4.00E-36	128
TcMYB43	ATMYB77	52.48	101	48	0	349	651	6	106	2.00E-35	126
TcMYB43	ATMYB119	42.07	145	79	2	250	669	73	216	7.00E-32	119
TcMYB43	ATMYB66	34.22	187	108	5	349	864	18	203	2.00E-31	112
TcMYB43	AtMYB64	44.44	135	75	1	349	753	105	227	4.00E-30	114
TcMYB43	ATMYB23	48.04	102	52	1	349	651	14	115	3.00E-29	107
TcMYB43	ATMYB118	47.17	106	56	0	349	666	189	294	1.00E-28	109
TcMYB43	ATMYB0	47.06	102	53	1	349	651	16	117	3.00E-28	104
TcMYB43	ATMYB67	45.31	128	63	3	301	663	3	129	3.00E-28	106
TcMYB43	AtMYB115	43.12	109	62	0	322	648	149	257	3.00E-28	107
TcMYB43	ATMYB15	35.75	179	98	5	349	834	14	187	5.00E-28	105
TcMYB43	AtMYB10	46.3	108	56	2	349	666	16	122	5.00E-28	104
TcMYB43	AtMYB100	45.45	99	53	2	355	648	28	125	6.00E-28	103
TcMYB43	AtMYB103	47.01	117	60	2	349	693	14	129	7.00E-28	106
TcMYB43	ATMYB63	46.3	108	56	2	349	666	16	122	7.00E-28	105
TcMYB43	ATMYB121	41.26	143	77	3	331	738	23	164	1.00E-27	104
TcMYB43	AtMYB19	47.12	104	53	2	349	654	14	116	2.00E-27	103
TcMYB43	AtMYB27	44.25	113	61	3	349	681	11	120	2.00E-27	102
TcMYB43	AtMYB98	42.37	118	68	0	349	702	217	334	5.00E-27	105
TcMYB43	AtMYB18	37.31	134	82	2	349	744	12	144	7.00E-27	102
TcMYB43	ATMYB58	36.03	136	86	1	349	753	16	151	9.00E-27	101
TcMYB43	AtMYB51	47.06	102	53	1	349	651	15	116	9.00E-27	103
TcMYB43	AtMYB104	39.68	126	75	1	349	723	18	143	1.00E-26	103
TcMYB43	AtMYB45	34.97	163	95	4	349	804	20	181	1.00E-26	100
TcMYB43	AtMYB82	46.08	102	54	1	349	651	14	115	2.00E-26	98.6
TcMYB43	ATMYB13	38.82	152	83	5	349	774	14	164	3.00E-26	99.4
TcMYB43	ATMYB80	46.6	103	53	2	349	651	14	115	5.00E-26	100
TcMYB43	AtMYB97	38.73	142	86	2	349	771	21	159	7.00E-26	101
TcMYB43	ATMYB35	39.39	132	79	2	349	741	14	143	9.00E-26	99.8
TcMYB43	ATMYB72	44.04	109	59	2	349	669	16	123	1.00E-25	99
TcMYB43	AtMYB40	45.71	105	55	2	349	657	14	117	2.00E-25	97.8
TcMYB43	AtMYB81	41.22	131	76	2	286	675	9	131	2.00E-25	100
TcMYB43	AtMYB53	47.57	103	52	2	349	651	14	115	2.00E-25	98.6
TcMYB43	ATMYB101	41.73	127	72	3	328	702	13	134	2.00E-25	100
TcMYB43	ATMYB59-3	37.93	145	88	3	349	777	10	152	2.00E-25	96.7
TcMYB43	ATMYB120	36.91	149	93	3	232	675	4	137	3.00E-25	100
TcMYB43	ATMYB48	44.86	107	57	2	349	663	9	114	4.00E-25	96.7
TcMYB43	ATMYB46	40	115	67	2	331	669	14	127	4.00E-25	97.1
TcMYB43	AtMYB49	45.1	102	55	1	349	651	14	115	6.00E-25	97.4
TcMYB43	AtMYB41	44.12	102	56	1	349	651	14	115	6.00E-25	96.7
TcMYB43	AtMYB36	40	140	82	5	349	762	14	142	7.00E-25	97.4
TcMYB43	ATMYB68	37.76	143	84	4	349	762	14	152	9.00E-25	97.8
TcMYB43	ATMYB95	43.69	103	56	2	349	651	14	115	9.00E-25	95.9
TcMYB43	ATMYB122	46.88	96	50	1	349	633	14	109	1.00E-24	97.1
TcMYB43	ATMYB34	41.38	116	67	2	319	663	9	119	1.00E-24	96.3
TcMYB43	AtMYB79	40.8	125	72	2	349	717	8	131	1.00E-24	95.5
TcMYB43	ATMYB33	33.95	162	106	2	259	741	11	165	1.00E-24	99
TcMYB43	ATMYB65	35	140	86	2	271	675	13	152	1.00E-24	99
TcMYB43	AtMYB114	40.78	103	60	1	349	654	10	112	1.00E-24	91.7
TcMYB43	ATMYB5	42.45	106	60	1	349	663	25	130	2.00E-24	94.7
TcMYB43	ATMYB86	42.99	107	59	2	349	663	14	119	2.00E-24	96.7
TcMYB43	ATMYB3	45.63	103	54	2	349	651	14	115	2.00E-24	94.7
TcMYB43	AtMYB32	38.93	131	78	2	349	735	14	143	2.00E-24	95.1
TcMYB43	MYB8	42.16	102	58	1	349	651	14	115	2.00E-24	93.6
TcMYB43	ATMYB61	37.93	145	87	4	349	774	14	151	2.00E-24	96.7
TcMYB43	AtMYB107	45.1	102	55	1	349	651	14	115	2.00E-24	95.9
TcMYB43	ATMYB92	46.6	103	53	2	349	651	14	115	2.00E-24	95.9
TcMYB43	AtMYB9	45.1	102	55	1	349	651	14	115	3.00E-24	95.9
TcMYB43	ATMYB71	39.84	123	72	2	349	711	20	141	3.00E-24	94.4
TcMYB43	ATMYB84	43.69	103	56	2	349	651	14	116	3.00E-24	95.1
TcMYB43	AtMYB116	37.88	132	80	3	319	708	13	140	4.00E-24	94.4
TcMYB43	ATMYB14	42.61	115	64	2	349	687	14	127	4.00E-24	93.6
TcMYB43	AtMYB74	44.66	103	55	2	349	651	15	116	5.00E-24	94.7
TcMYB43	ATMYB123	36.55	145	91	4	349	780	16	141	6.00E-24	93.2
TcMYB43	ATMYB4	44.66	103	55	2	349	651	14	115	7.00E-24	93.6
TcMYB43	ATMYB55	33.14	175	84	5	349	774	14	185	7.00E-24	94.7
TcMYB43	AtMYB22	32.39	142	90	3	274	681	23	163	1.00E-23	92.4
TcMYB43	AtMYB50	43.69	103	56	2	349	651	14	115	1.00E-23	93.6
TcMYB43	ATMYB2	47.37	95	49	1	349	630	22	116	2.00E-23	92.4
TcMYB43	ATMYB28	43.4	106	59	1	349	663	14	119	2.00E-23	94
TcMYB43	AtMYB17	44.66	103	55	2	349	651	14	115	2.00E-23	92.8
TcMYB43	AtMYB83	41.67	108	62	1	349	669	32	139	2.00E-23	93.6
TcMYB43	ATMYB75	40.78	103	60	1	349	654	10	112	2.00E-23	91.7
TcMYB43	AtMYB47	40.78	103	59	2	349	651	14	115	2.00E-23	92
TcMYB43	AtMYB6	42.72	103	57	2	349	651	14	115	2.00E-23	91.3
TcMYB43	ATMYB106	44.66	103	55	2	349	651	57	158	2.00E-23	94

TcMYB43	AtMYB112	41.44	111	64	2	301	630	19	128	2.00E-23	91.3
TcMYB43	ATMYB90	34.9	149	93	2	349	783	10	158	3.00E-23	91.3
TcMYB43	ATMYB94	41.67	108	62	1	349	669	14	121	3.00E-23	92.8
TcMYB43	ATMYB30	36.69	139	87	2	349	762	14	146	3.00E-23	92.4
TcMYB43	ATMYB102	44.66	103	55	2	349	651	14	115	5.00E-23	92.4
TcMYB43	AtMYB62	34.87	152	87	4	349	768	21	171	5.00E-23	91.3
TcMYB43	MYB7	41.75	103	58	2	349	651	14	115	7.00E-23	90.5
TcMYB43	AtMYB93	43.14	102	57	1	349	651	14	115	8.00E-23	92
TcMYB43	ATMYB38	43.69	103	56	2	349	651	14	116	8.00E-23	90.9
TcMYB43	ATMYB16	42.16	102	58	1	349	651	14	115	9.00E-23	91.3
TcMYB43	ATMYB96	38.06	134	80	2	349	741	14	147	9.00E-23	91.7
TcMYB43	AtMYB60	37.14	140	78	3	349	738	14	153	1.00E-22	90.1
TcMYB43	AtMYB43	39.2	125	74	2	349	717	14	137	1.00E-22	90.9
TcMYB43	AtMYB76	45.1	102	55	1	349	651	14	115	1.00E-22	90.9
TcMYB43	ATMYB3	36.73	147	92	4	235	672	5	129	1.00E-22	88.6
TcMYB43	ATMYB31	44.12	102	56	1	349	651	14	115	2.00E-22	90.5
TcMYB43	AtMYB113	38.68	106	64	1	349	663	10	115	2.00E-22	88.6
TcMYB43	ATMYB57	29.89	174	120	4	349	864	27	188	2.00E-22	87.4
TcMYB43	AtMYB108	43.52	108	59	3	313	630	12	115	3.00E-22	89.7
TcMYB43	ATMYB111	37.14	140	87	2	349	765	14	151	4.00E-22	89.7
TcMYB43	ATMYB29	45.83	96	51	1	349	633	14	109	5.00E-22	89.4
TcMYB43	ATMYB37	40	110	63	2	349	669	14	122	8.00E-22	88.6
TcMYB43	AtMYB20	41.51	106	60	2	349	660	14	118	1.00E-21	87
TcMYB43	AtMYB42	42.72	103	57	2	349	651	14	115	1.00E-21	87
TcMYB43	ATMYB11	34.56	136	76	2	349	717	14	149	3.00E-21	87
TcMYB43	AtMYB24	42.11	95	54	1	349	630	19	113	3.00E-21	84.3
TcMYB43	AtMYB85	42.72	103	57	2	349	651	14	115	4.00E-21	85.5
TcMYB43	ATMYB26	39.64	111	57	2	349	651	14	124	4.00E-21	87
TcMYB43	ATMYB12	40.2	102	60	1	349	651	14	115	7.00E-21	86.3
TcMYB43	ATMYB88	36.89	103	65	0	358	666	33	135	1.00E-20	86.7
TcMYB43	ATMYB87	38.83	103	61	2	349	651	14	116	1.00E-20	84.7
TcMYB43	AtMYB124	36.89	103	65	0	358	666	28	130	2.00E-20	85.9
TcMYB43	ATMYB99	42.73	110	54	4	349	651	15	123	4.00E-20	82
TcMYB43	ATMYB91	35.9	117	72	1	349	690	4	120	1.00E-19	82.8
TcMYB43	ATMYB78	34.51	142	78	4	250	630	2	136	2.00E-19	81.3
TcMYB44	AtMYB108	79.49	78	16	0	10	243	50	127	3.00E-42	139
TcMYB44	AtMYB62	55.28	123	46	3	10	351	50	169	5.00E-42	137
TcMYB44	ATMYB3	58.18	110	46	1	13	342	52	154	6.00E-42	135
TcMYB44	ATMYB2	70.65	92	27	1	13	288	52	142	8.00E-42	136
TcMYB44	ATMYB48	48.55	138	70	2	13	423	39	167	3.00E-40	132
TcMYB44	AtMYB112	46.43	140	75	1	13	432	64	200	3.00E-40	131
TcMYB44	ATMYB59-3	42.5	160	75	3	13	441	40	199	4.00E-40	131
TcMYB44	AtMYB24	78.87	71	15	0	13	225	49	119	2.00E-39	129
TcMYB44	AtMYB116	69.05	84	26	1	13	264	50	130	6.00E-39	129
TcMYB44	ATMYB57	43.48	161	89	3	13	489	57	206	3.00E-38	125
TcMYB44	ATMYB78	67.39	92	16	1	10	243	57	148	1.00E-37	127
TcMYB44	ATMYB121	72.97	74	20	0	10	231	58	131	2.00E-37	125
TcMYB44	AtMYB79	74.65	71	18	0	13	225	38	108	1.00E-35	120
TcMYB44	AtMYB27	69.86	73	22	0	13	231	41	113	2.00E-35	119
TcMYB44	ATMYB71	74.65	71	18	0	13	225	50	120	2.00E-35	119
TcMYB44	ATMYB66	40.26	154	88	2	13	462	48	201	1.00E-33	113
TcMYB44	ATMYB23	39.07	151	86	2	13	447	44	194	5.00E-32	109
TcMYB44	AtMYB10	47.27	110	58	1	13	342	46	151	6.00E-32	110
TcMYB44	ATMYB58	34.95	206	118	5	13	582	46	237	8.00E-32	110
TcMYB44	ATMYB63	66.67	75	25	0	13	237	46	120	9.00E-32	110
TcMYB44	AtMYB32	67.61	71	23	0	13	225	44	114	2.00E-31	109
TcMYB44	ATMYB0	56.18	89	39	1	13	279	46	129	2.00E-31	108
TcMYB44	AtMYB40	40.58	138	80	2	13	420	44	180	3.00E-31	108
TcMYB44	AtMYB107	65.33	75	26	0	13	237	44	118	3.00E-31	110
TcMYB44	ATMYB99	67.61	71	23	0	13	225	52	122	5.00E-31	107
TcMYB44	AtMYB19	64.79	71	25	0	13	225	44	114	6.00E-31	108
TcMYB44	ATMYB123	64.56	79	24	1	13	237	46	124	6.00E-31	107
TcMYB44	ATMYB46	44.88	127	70	2	13	393	50	172	7.00E-31	108
TcMYB44	ATMYB14	47.54	122	59	3	13	363	44	160	7.00E-31	107
TcMYB44	AtMYB49	67.61	71	23	0	13	225	44	114	8.00E-31	108
TcMYB44	ATMYB3	67.61	71	23	0	13	225	44	114	9.00E-31	107
TcMYB44	ATMYB29	66.2	71	24	0	13	225	44	114	3.00E-30	107
TcMYB44	ATMYB5	67.61	71	23	0	13	225	55	125	3.00E-30	105
TcMYB44	AtMYB18	38.73	142	86	2	13	435	42	182	3.00E-30	106
TcMYB44	AtMYB76	64.79	71	25	0	13	225	44	114	4.00E-30	107
TcMYB44	AtMYB20	66.2	71	24	0	13	225	44	114	4.00E-30	106
TcMYB44	ATMYB28	48.62	109	49	1	13	318	44	152	4.00E-30	107
TcMYB44	ATMYB12	66.2	71	24	0	13	225	44	114	5.00E-30	107
TcMYB44	ATMYB13	42.75	138	74	1	13	411	44	181	5.00E-30	105
TcMYB44	MYB7	66.2	71	24	0	13	225	44	114	6.00E-30	105
TcMYB44	AtMYB85	63.38	71	26	0	13	225	44	114	8.00E-30	105
TcMYB44	AtMYB83	50.94	106	50	2	13	324	62	163	8.00E-30	106
TcMYB44	AtMYB103	66.2	71	24	0	13	225	44	114	9.00E-30	107
TcMYB44	ATMYB96	35.94	192	123	3	13	588	44	213	9.00E-30	106
TcMYB44	AtMYB51	67.61	71	23	0	13	225	45	115	9.00E-30	106

TcMYB44	AtMYB82	63.38	71	26	0	13	225	44	114	1.00E-29	103
TcMYB44	ATMYB4	66.2	71	24	0	13	225	44	114	1.00E-29	105
TcMYB44	ATMYB15	62.34	77	29	0	13	243	44	120	1.00E-29	105
TcMYB44	AtMYB6	64.79	71	25	0	13	225	44	114	1.00E-29	103
TcMYB44	AtMYB43	66.2	71	24	0	13	225	44	114	1.00E-29	105
TcMYB44	AtMYB53	62.67	75	28	0	13	237	44	118	1.00E-29	105
TcMYB44	MYB8	65.71	70	24	0	16	225	45	114	1.00E-29	103
TcMYB44	AtMYB9	62.67	75	28	0	13	237	44	118	1.00E-29	105
TcMYB44	ATMYB106	64.79	71	25	0	13	225	87	157	1.00E-29	106
TcMYB44	ATMYB16	64.79	71	25	0	13	225	44	114	2.00E-29	105
TcMYB44	AtMYB42	64.79	71	25	0	13	225	44	114	3.00E-29	103
TcMYB44	ATMYB86	35.52	183	111	5	13	540	44	223	3.00E-29	105
TcMYB44	ATMYB111	57.5	80	34	0	13	252	44	123	4.00E-29	104
TcMYB44	ATMYB33	63.01	73	27	0	13	231	64	136	4.00E-29	106
TcMYB44	AtMYB74	63.38	71	26	0	13	225	45	115	4.00E-29	104
TcMYB44	AtMYB50	66.2	71	24	0	13	225	44	114	7.00E-29	103
TcMYB44	AtMYB45	44.76	105	56	1	13	321	50	154	8.00E-29	102
TcMYB44	ATMYB61	64	75	27	0	13	237	44	118	1.00E-28	103
TcMYB44	ATMYB26	63.77	69	25	0	19	225	55	123	1.00E-28	103
TcMYB44	ATMYB65	60.27	73	29	0	13	231	73	145	1.00E-28	105
TcMYB44	ATMYB11	63.38	71	26	0	13	225	44	114	2.00E-28	103
TcMYB44	AtMYB36	52.63	95	44	1	16	297	46	140	2.00E-28	102
TcMYB44	AtMYB41	61.97	71	27	0	13	225	44	114	2.00E-28	101
TcMYB44	ATMYB94	48.18	110	50	3	13	321	44	150	3.00E-28	102
TcMYB44	ATMYB72	43.55	124	68	1	13	378	46	169	3.00E-28	101
TcMYB44	ATMYB38	47.12	104	55	0	13	324	45	148	3.00E-28	101
TcMYB44	ATMYB87	48.54	103	47	1	16	306	46	148	3.00E-28	101
TcMYB44	ATMYB102	61.97	71	27	0	13	225	44	114	3.00E-28	102
TcMYB44	ATMYB101	58.9	73	30	0	13	231	50	122	3.00E-28	103
TcMYB44	ATMYB67	57.75	71	30	0	13	225	54	124	3.00E-28	101
TcMYB44	ATMYB95	39.23	130	79	1	13	402	44	172	4.00E-28	100
TcMYB44	AtMYB97	58.9	73	30	0	19	237	53	125	4.00E-28	102
TcMYB44	ATMYB31	46.02	113	61	1	13	351	44	153	5.00E-28	101
TcMYB44	ATMYB84	43.31	127	72	2	16	396	46	150	5.00E-28	101
TcMYB44	ATMYB30	50.5	101	45	2	13	300	44	143	6.00E-28	101
TcMYB44	AtMYB17	61.97	71	27	0	13	225	44	114	8.00E-28	100
TcMYB44	AtMYB60	43.97	116	58	2	13	339	44	159	8.00E-28	100
TcMYB44	ATMYB55	64.29	70	25	0	16	225	57	126	9.00E-28	101
TcMYB44	ATMYB92	60	75	30	0	13	237	44	118	1.00E-27	100
TcMYB44	ATMYB120	57.33	75	32	0	13	237	58	132	1.00E-27	102
TcMYB44	ATMYB80	59.15	71	29	0	13	225	44	114	2.00E-27	100
TcMYB44	ATMYB37	60.56	71	28	0	13	225	45	115	2.00E-27	100
TcMYB44	ATMYB34	60	75	30	0	13	237	44	118	3.00E-27	99
TcMYB44	ATMYB122	61.97	71	27	0	13	225	44	114	4.00E-27	99.4
TcMYB44	AtMYB114	59.15	71	29	0	13	225	40	110	4.00E-27	94.7
TcMYB44	AtMYB81	57.53	73	31	0	13	231	52	124	4.00E-27	100
TcMYB44	AtMYB93	61.64	73	28	0	19	237	46	118	4.00E-27	99.8
TcMYB44	ATMYB35	34.55	165	97	3	13	474	44	208	1.00E-26	97.8
TcMYB44	ATMYB68	62.86	70	26	0	16	225	46	115	1.00E-26	98.6
TcMYB44	ATMYB90	47.92	96	50	1	13	300	40	132	1.00E-26	96.3
TcMYB44	ATMYB75	59.15	71	29	0	13	225	40	110	2.00E-26	95.5
TcMYB44	AtMYB113	59.15	71	29	0	13	225	40	110	3.00E-26	95.1
TcMYB44	AtMYB47	54.93	71	32	0	13	225	44	114	9.00E-26	94.4
TcMYB44	AtMYB104	52.94	68	32	0	13	216	48	115	4.00E-22	86.3
TcMYB44	AtMYB70	55.22	67	30	0	25	225	46	112	4.00E-21	82.4
TcMYB44	ATMYB25	50.7	71	35	0	25	237	83	153	1.00E-20	82
TcMYB44	ATMYB54	40.86	93	55	0	25	303	39	131	1.00E-20	80.1
TcMYB44	ATMYB73	52.24	67	32	0	25	225	46	112	3.00E-20	80.1
TcMYB44	ATMYB52	57.38	61	26	0	25	207	38	98	5.00E-20	78.6
TcMYB44	ATMYB44	48.61	72	37	0	25	240	39	110	8.00E-20	79
TcMYB44	AtMYB117	43.24	74	42	0	25	246	131	204	2.00E-19	78.6
TcMYB44	ATMYB105	52.46	61	29	0	25	207	140	200	2.00E-19	78.2
TcMYB44	ATMYB91	43.59	78	44	0	19	252	38	115	2.00E-19	78.2
TcMYB44	AtMYB109	41.05	95	55	1	25	306	89	183	4.00E-19	77.8
TcMYB44	AtMYB115	34.48	116	68	2	25	348	191	303	4.00E-19	77.4
TcMYB44	AtMYB56	46.84	79	36	1	19	237	124	202	4.00E-19	77
TcMYB44	AtMYB1	37.14	105	63	1	25	330	88	192	5.00E-19	77.4
TcMYB44	AtMYB64	34.86	109	71	1	25	351	138	245	8.00E-19	77
TcMYB44	ATMYB77	49.25	67	34	0	25	225	39	105	2.00E-18	75.1
TcMYB44	AtMYB100	44.93	69	38	0	25	231	59	127	2.00E-18	73.9
TcMYB44	ATMYB69	45.24	84	46	1	25	276	52	131	3.00E-18	73.9
TcMYB44	ATMYB119	42.47	73	42	0	25	243	138	210	3.00E-18	75.5
TcMYB44	ATMYB118	45.07	71	39	0	25	237	222	292	3.00E-18	75.5
TcMYB44	AtMYB98	41.03	78	46	0	25	258	250	327	4.00E-18	75.1
TcMYB44	AtMYB98	27.94	68	48	1	22	222	197	264	3.00E-05	36.6
TcMYB44	ATMYB110	43.66	71	40	0	25	237	100	170	2.00E-17	72
TcMYB44	AtMYB89	36.67	90	55	1	25	288	89	178	1.00E-16	68.6
TcMYB44	AtMYB22	29.29	140	89	2	25	414	86	225	3.00E-16	68.2
TcMYB44	AtMYB124	36.36	99	61	2	25	315	58	155	7.00E-15	65.5
TcMYB44	ATMYB88	41.33	75	44	1	25	249	63	136	5.00E-14	63.2

TcMYB45	AtMYB70	58	150	33	2	22	381	7	156	2.00E-53	164
TcMYB45	ATMYB73	57.04	142	57	1	22	435	7	148	2.00E-52	162
TcMYB45	ATMYB44	76.92	91	21	0	31	303	3	93	5.00E-51	158
TcMYB45	ATMYB77	74.73	91	23	0	31	303	3	93	5.00E-50	155
TcMYB45	AtMYB109	65.56	90	31	0	34	303	54	143	4.00E-43	140
TcMYB45	AtMYB1	64.84	91	32	0	31	303	52	142	6.00E-41	134
TcMYB45	ATMYB25	57.29	96	41	0	16	303	42	137	1.00E-38	128
TcMYB45	ATMYB54	55.68	88	39	0	40	303	6	93	1.00E-36	119
TcMYB45	ATMYB52	42.86	133	71	2	40	423	5	137	2.00E-36	119
TcMYB45	ATMYB69	53.41	88	41	0	40	303	19	106	5.00E-35	115
TcMYB45	ATMYB105	56.82	88	38	0	40	303	107	194	7.00E-35	117
TcMYB45	AtMYB117	56.82	88	38	0	40	303	98	185	1.00E-34	117
TcMYB45	AtMYB56	56.82	88	38	0	40	303	93	180	3.00E-34	115
TcMYB45	ATMYB110	47.47	99	50	1	13	303	56	154	8.00E-31	106
TcMYB45	ATMYB121	55.06	89	39	1	40	303	29	117	1.00E-29	102
TcMYB45	AtMYB49	50	102	50	1	1	303	1	102	3.00E-28	99.8
TcMYB45	ATMYB92	51.69	89	42	1	40	303	14	102	5.00E-28	99.4
TcMYB45	ATMYB23	48.04	102	52	1	1	303	1	102	1.00E-27	95.9
TcMYB45	ATMYB23	31.37	51	35	1	40	192	67	116	4.00E-04	32
TcMYB45	ATMYB34	52.81	89	41	1	40	303	14	102	3.00E-27	96.7
TcMYB45	ATMYB14	51.69	89	42	1	40	303	14	102	4.00E-27	95.5
TcMYB45	AtMYB97	51.69	89	42	1	40	303	21	109	4.00E-27	97.8
TcMYB45	ATMYB80	51.69	89	42	1	40	303	14	102	5.00E-27	96.7
TcMYB45	AtMYB79	51.58	95	45	2	22	303	3	96	5.00E-27	95.5
TcMYB45	AtMYB51	52.81	89	41	1	40	303	15	103	5.00E-27	97.1
TcMYB45	AtMYB60	49.46	93	42	2	40	303	14	102	7.00E-27	95.5
TcMYB45	AtMYB98	44	100	56	0	4	303	205	304	2.00E-26	96.3
TcMYB45	AtMYB98	37.21	43	27	1	40	168	269	310	6.00E-05	34.7
TcMYB45	ATMYB13	48.96	96	47	2	22	303	8	102	2.00E-26	93.6
TcMYB45	ATMYB13	30.19	53	37	1	40	198	67	118	6.00E-04	31.6
TcMYB45	ATMYB118	46.39	97	52	0	13	303	180	276	2.00E-26	96.3
TcMYB45	ATMYB118	29.51	61	43	2	40	222	241	298	9.00E-04	31.2
TcMYB45	ATMYB66	51.69	89	42	1	40	303	18	106	2.00E-26	92.4
TcMYB45	ATMYB66	31.37	51	35	1	40	192	71	120	4.00E-04	32
TcMYB45	ATMYB63	50.56	89	43	1	40	303	16	104	2.00E-26	94.4
TcMYB45	ATMYB63	25.97	77	57	1	31	261	66	141	1.00E-04	33.5
TcMYB45	ATMYB48	47.37	95	49	2	22	303	4	97	2.00E-26	93.6
TcMYB45	ATMYB119	42.42	99	57	0	7	303	94	192	3.00E-26	95.9
TcMYB45	ATMYB119	37.21	43	27	1	40	168	157	198	7.00E-04	31.6
TcMYB45	AtMYB82	50.56	89	43	1	40	303	14	102	3.00E-26	92
TcMYB45	AtMYB82	31.48	54	37	1	40	201	67	119	7.00E-04	31.2
TcMYB45	ATMYB59-3	46.07	89	47	1	40	303	10	98	3.00E-26	92.8
TcMYB45	ATMYB59-3	23.08	65	48	2	40	228	63	126	5.00E-04	31.6
TcMYB45	AtMYB53	45.1	102	55	1	1	303	1	102	4.00E-26	94
TcMYB45	AtMYB107	50.56	89	43	1	40	303	14	102	5.00E-26	94
TcMYB45	ATMYB31	46.46	99	48	2	22	303	8	102	5.00E-26	94
TcMYB45	ATMYB35	52.81	89	41	1	40	303	14	102	6.00E-26	93.6
TcMYB45	AtMYB89	43.68	87	49	0	43	303	57	143	6.00E-26	90.9
TcMYB45	ATMYB46	52.81	89	41	1	40	303	20	108	7.00E-26	92.8
TcMYB45	ATMYB46	29.09	55	39	1	31	195	70	123	6.00E-04	31.6
TcMYB45	ATMYB106	51.69	89	42	1	40	303	57	145	7.00E-26	94.4
TcMYB45	AtMYB116	45.26	95	51	1	22	303	14	108	7.00E-26	92.8
TcMYB45	ATMYB71	49.44	89	44	1	40	303	20	108	8.00E-26	92.4
TcMYB45	AtMYB17	47.78	90	45	2	40	303	14	102	9.00E-26	92.8
TcMYB45	AtMYB41	50.56	89	43	1	40	303	14	102	1.00E-25	92.4
TcMYB45	ATMYB72	50	90	43	2	40	303	16	104	1.00E-25	92.4
TcMYB45	AtMYB64	43.82	89	50	0	37	303	104	192	1.00E-25	94
TcMYB45	AtMYB62	44	100	55	1	7	303	10	109	1.00E-25	92
TcMYB45	AtMYB93	48.31	89	45	1	40	303	14	102	2.00E-25	92.8
TcMYB45	ATMYB101	50.56	89	43	1	40	303	20	108	2.00E-25	93.6
TcMYB45	ATMYB16	50.56	89	43	1	40	303	14	102	3.00E-25	92
TcMYB45	AtMYB45	50.56	89	43	1	40	303	20	108	3.00E-25	90.9
TcMYB45	ATMYB95	49.44	89	44	1	40	303	14	102	3.00E-25	90.9
TcMYB45	ATMYB95	25.86	58	43	1	40	213	67	123	4.00E-04	32
TcMYB45	AtMYB74	50.56	89	43	1	40	303	15	103	4.00E-25	91.7
TcMYB45	ATMYB15	46.32	95	50	1	22	303	8	102	4.00E-25	90.9
TcMYB45	ATMYB15	31.67	60	40	2	40	216	67	125	6.00E-04	31.6
TcMYB45	ATMYB30	47.31	93	44	2	40	303	14	102	5.00E-25	91.3
TcMYB45	ATMYB122	49.44	89	44	1	40	303	14	102	5.00E-25	91.3
TcMYB45	ATMYB0	50.56	89	43	1	40	303	16	104	6.00E-25	89.4
TcMYB45	ATMYB0	33.33	51	34	1	40	192	69	118	2.00E-04	33.1
TcMYB45	ATMYB26	50	98	39	2	40	303	14	111	6.00E-25	91.7
TcMYB45	AtMYB9	50.56	89	43	1	40	303	14	102	6.00E-25	91.3
TcMYB45	ATMYB2	47.37	95	49	1	22	303	16	110	6.00E-25	90.1
TcMYB45	ATMYB102	50.56	89	43	1	40	303	14	102	7.00E-25	91.3
TcMYB45	ATMYB96	44.44	99	50	2	22	303	8	102	7.00E-25	91.3
TcMYB45	AtMYB47	47.19	89	46	1	40	303	14	102	8.00E-25	89.7
TcMYB45	AtMYB115	44.32	88	49	0	40	303	158	245	1.00E-24	90.5
TcMYB45	AtMYB115	32.86	70	47	2	40	249	210	276	5.00E-04	32
TcMYB45	ATMYB67	50.56	89	43	1	40	303	24	112	1.00E-24	89.7

TcMYB45	ATMYB3	44.33	97	53	1	16	303	14	110	2.00E-24	88.2
TcMYB45	AtMYB27	46.67	90	46	2	40	303	11	99	2.00E-24	88.2
TcMYB45	ATMYB3	47.37	95	49	1	22	303	8	102	2.00E-24	88.6
TcMYB45	ATMYB94	46.24	93	45	2	40	303	14	102	2.00E-24	89.7
TcMYB45	MYB8	45.26	95	51	1	22	303	8	102	2.00E-24	87.4
TcMYB45	ATMYB4	48.42	95	48	1	22	303	8	102	3.00E-24	88.6
TcMYB45	AtMYB18	46.08	102	54	2	1	303	1	100	3.00E-24	88.6
TcMYB45	AtMYB24	46.07	89	47	1	40	303	19	107	3.00E-24	87
TcMYB45	AtMYB36	47.78	90	45	2	40	303	14	103	4.00E-24	89
TcMYB45	ATMYB38	50	90	43	2	40	303	14	103	5.00E-24	88.2
TcMYB45	AtMYB6	49.47	95	47	1	22	303	8	102	5.00E-24	87
TcMYB45	ATMYB120	48.31	89	45	1	40	303	28	116	6.00E-24	89.7
TcMYB45	ATMYB37	48.89	90	44	2	40	303	14	103	7.00E-24	88.2
TcMYB45	AtMYB103	50.56	89	43	1	40	303	14	102	8.00E-24	88.6
TcMYB45	ATMYB65	46.07	89	47	1	40	303	43	131	8.00E-24	89.4
TcMYB45	MYB7	49.44	89	44	1	40	303	14	102	1.00E-23	86.7
TcMYB45	ATMYB87	47.78	90	45	2	40	303	14	103	1.00E-23	87
TcMYB45	ATMYB57	47.19	89	46	1	40	303	27	115	2.00E-23	85.1
TcMYB45	AtMYB81	47.19	89	46	1	40	303	22	110	2.00E-23	88.2
TcMYB45	AtMYB32	51.69	89	42	1	40	303	14	102	2.00E-23	86.3
TcMYB45	AtMYB108	44.21	95	52	1	22	303	15	109	3.00E-23	86.7
TcMYB45	AtMYB112	42.55	94	53	1	25	303	29	122	3.00E-23	85.1
TcMYB45	AtMYB104	44.44	90	48	2	40	303	18	106	3.00E-23	87
TcMYB45	ATMYB58	48.31	89	45	1	40	303	16	104	4.00E-23	85.5
TcMYB45	ATMYB68	47.78	90	45	2	40	303	14	103	4.00E-23	86.7
TcMYB45	AtMYB83	50.56	89	43	1	40	303	32	120	4.00E-23	86.3
TcMYB45	ATMYB61	47.19	89	46	1	40	303	14	102	5.00E-23	86.3
TcMYB45	ATMYB61	26.39	72	53	1	31	246	64	134	1.00E-04	33.9
TcMYB45	AtMYB10	45.56	90	47	2	40	303	16	104	6.00E-23	84.3
TcMYB45	ATMYB86	48.31	89	45	1	40	303	14	102	6.00E-23	85.9
TcMYB45	ATMYB33	46.07	89	47	1	40	303	34	122	7.00E-23	86.7
TcMYB45	ATMYB84	46.67	90	46	2	40	303	14	103	8.00E-23	85.1
TcMYB45	AtMYB50	47.19	89	46	1	40	303	14	102	8.00E-23	85.1
TcMYB45	ATMYB11	46.32	95	50	1	22	303	8	102	1.00E-22	85.1
TcMYB45	ATMYB55	43.56	101	44	1	40	303	14	114	2.00E-22	84.7
TcMYB45	AtMYB19	44.55	101	52	3	13	303	3	102	2.00E-22	83.6
TcMYB45	ATMYB28	47.19	89	46	1	40	303	14	102	4.00E-22	84
TcMYB45	ATMYB29	47.19	89	46	1	40	303	14	102	4.00E-22	83.6
TcMYB45	ATMYB5	46.07	89	47	1	40	303	25	113	7.00E-22	81.6
TcMYB45	ATMYB5	29.2	113	68	6	43	345	80	189	7.00E-04	31.2
TcMYB45	ATMYB12	44.21	95	52	1	22	303	8	102	1.00E-21	82.8
TcMYB45	ATMYB12	28	75	51	2	31	246	64	137	8.00E-05	34.3
TcMYB45	AtMYB76	47.19	89	46	1	40	303	14	102	1.00E-21	82.4
TcMYB45	ATMYB111	45.26	95	51	1	22	303	8	102	1.00E-21	82.4
TcMYB45	ATMYB123	44.21	95	52	2	22	303	12	104	2.00E-21	80.5
TcMYB45	ATMYB91	45.35	86	44	1	49	297	7	92	5.00E-21	80.9
TcMYB45	AtMYB40	44.44	90	48	2	40	303	14	102	9.00E-21	79
TcMYB45	AtMYB114	41.57	89	51	1	40	303	10	98	1.00E-20	75.9
TcMYB45	AtMYB114	34.62	52	34	1	40	195	63	113	0.001	30.4
TcMYB45	AtMYB100	37.23	94	58	3	25	303	23	113	3.00E-20	77
TcMYB45	ATMYB88	38.82	85	52	0	49	303	33	117	5.00E-20	78.6
TcMYB45	ATMYB88	30.16	63	44	1	40	228	82	143	6.00E-06	37.7
TcMYB45	ATMYB78	37.5	112	55	2	13	303	19	130	8.00E-20	77
TcMYB45	AtMYB42	43.82	89	49	1	40	303	14	102	3.00E-19	75.1
TcMYB45	ATMYB75	40.45	89	52	1	40	303	10	98	5.00E-19	73.9
TcMYB45	AtMYB20	41.67	96	54	2	22	303	8	102	6.00E-19	74.3
TcMYB45	AtMYB124	36.47	85	54	0	49	303	28	112	7.00E-19	75.1
TcMYB45	AtMYB124	30.56	72	50	1	40	255	77	147	1.00E-06	39.7
TcMYB45	AtMYB85	43.82	89	49	1	40	303	14	102	1.00E-18	73.2
TcMYB45	ATMYB90	40.45	89	52	1	40	303	10	98	2.00E-18	72.4
TcMYB45	AtMYB113	39.78	93	51	2	40	303	10	98	3.00E-18	72
TcMYB45	AtMYB43	40.62	96	55	2	22	303	8	102	4.00E-18	72.4
TcMYB45	ATMYB99	41.18	102	52	2	22	303	9	110	1.00E-17	70.1
TcMYB45	AtMYB22	35.16	91	59	1	31	303	51	140	1.00E-15	65.1
TcMYB45	AtMYB22	35.19	54	35	2	40	201	105	156	6.00E-04	31.6
TcMYB46	ATMYB33	36.3	595	361	19	43	1773	4	519	2.00E-93	290
TcMYB46	ATMYB65	36.46	565	340	18	118	1755	41	550	1.00E-86	273
TcMYB46	ATMYB101	33.79	580	350	17	118	1755	18	489	3.00E-77	246
TcMYB46	ATMYB120	65.22	161	56	0	67	549	9	169	3.00E-68	223
TcMYB46	AtMYB81	55.91	186	78	3	91	636	7	188	2.00E-65	213
TcMYB46	AtMYB97	71.32	136	39	1	91	498	10	142	9.00E-65	210
TcMYB46	ATMYB5	41.75	194	106	4	118	678	23	209	5.00E-48	161
TcMYB46	AtMYB17	65.38	104	36	0	118	429	12	115	2.00E-47	161
TcMYB46	ATMYB15	40.09	222	132	5	118	780	12	215	4.00E-47	160
TcMYB46	ATMYB102	65.38	104	36	0	118	429	12	115	4.00E-47	162
TcMYB46	AtMYB107	65.38	104	36	0	118	429	12	115	5.00E-47	160
TcMYB46	AtMYB74	47.19	178	90	4	118	639	13	187	5.00E-47	160
TcMYB46	ATMYB66	43.68	174	89	1	79	573	3	176	7.00E-47	156
TcMYB46	ATMYB3	63.46	104	38	0	118	429	12	115	9.00E-47	158
TcMYB46	ATMYB96	35.86	290	144	6	118	861	12	301	1.00E-46	160

TcMYB46	AtMYB41	61.54	104	40	0	118	429	12	115	1.00E-46	158
TcMYB46	AtMYB9	62.62	107	40	0	118	438	12	118	4.00E-46	159
TcMYB46	AtMYB6	65.69	102	35	0	124	429	14	115	5.00E-46	155
TcMYB46	MYB8	62.5	104	39	0	118	429	12	115	9.00E-46	154
TcMYB46	ATMYB14	59.63	109	44	0	118	444	12	120	1.00E-45	154
TcMYB46	AtMYB79	62.86	105	39	0	121	435	7	111	2.00E-45	154
TcMYB46	ATMYB57	60.19	108	43	0	112	435	23	130	2.00E-45	152
TcMYB46	MYB7	63.46	104	38	0	118	429	12	115	2.00E-45	154
TcMYB46	AtMYB32	47.34	169	88	3	124	627	14	162	3.00E-45	154
TcMYB46	ATMYB106	51.8	139	66	2	118	531	55	190	8.00E-45	156
TcMYB46	ATMYB71	44.32	176	92	2	121	630	19	194	8.00E-45	153
TcMYB46	ATMYB4	64.71	102	36	0	124	429	14	115	1.00E-44	153
TcMYB46	ATMYB3	54.4	125	52	1	79	438	2	126	1.00E-44	151
TcMYB46	ATMYB92	63.46	104	38	0	118	429	12	115	2.00E-44	154
TcMYB46	ATMYB13	47.47	158	83	3	118	591	12	154	2.00E-44	151
TcMYB46	ATMYB28	49.68	155	77	3	103	564	8	158	2.00E-44	155
TcMYB46	AtMYB53	61.54	104	40	0	118	429	12	115	2.00E-44	153
TcMYB46	ATMYB29	45.18	197	104	7	103	681	8	192	3.00E-44	153
TcMYB46	AtMYB24	54.31	116	53	0	79	426	4	119	3.00E-44	149
TcMYB46	AtMYB51	54.07	135	60	2	118	516	13	147	3.00E-44	154
TcMYB46	ATMYB121	38.89	234	132	5	121	789	28	240	4.00E-44	151
TcMYB46	ATMYB63	43.72	183	94	4	118	639	14	194	4.00E-44	152
TcMYB46	ATMYB16	60.58	104	41	0	118	429	12	115	5.00E-44	152
TcMYB46	AtMYB49	55.56	126	53	2	118	486	12	137	6.00E-44	152
TcMYB46	ATMYB30	60.38	106	42	0	112	429	10	115	6.00E-44	152
TcMYB46	AtMYB76	47.68	151	64	2	103	510	8	157	6.00E-44	152
TcMYB46	AtMYB50	48.59	142	70	2	118	534	12	153	1.00E-43	151
TcMYB46	AtMYB60	61.54	104	40	0	118	429	12	115	1.00E-43	150
TcMYB46	ATMYB94	58.88	107	44	0	118	438	12	118	2.00E-43	151
TcMYB46	ATMYB58	41.34	179	105	2	118	654	14	184	2.00E-43	149
TcMYB46	AtMYB93	61.54	104	40	0	118	429	12	115	2.00E-43	152
TcMYB46	ATMYB86	54.33	127	57	2	118	495	12	135	4.00E-43	150
TcMYB46	ATMYB31	60.58	104	41	0	118	429	12	115	1.00E-42	149
TcMYB46	ATMYB34	59.81	107	43	0	118	438	12	118	1.00E-42	148
TcMYB46	ATMYB23	57.28	103	44	0	121	429	13	115	1.00E-42	145
TcMYB46	ATMYB99	52.55	137	54	4	118	495	13	146	1.00E-42	146
TcMYB46	AtMYB20	53.17	126	57	2	118	489	12	137	2.00E-42	147
TcMYB46	AtMYB116	50.83	120	59	1	79	438	6	124	3.00E-42	146
TcMYB46	AtMYB43	52.99	134	61	2	118	513	12	145	3.00E-42	147
TcMYB46	AtMYB85	50	140	68	3	118	531	12	150	4.00E-42	145
TcMYB46	ATMYB87	58.49	106	43	1	115	429	11	116	5.00E-42	146
TcMYB46	ATMYB46	57.01	107	46	0	118	438	18	124	5.00E-42	145
TcMYB46	AtMYB62	51.64	122	59	1	73	438	6	125	6.00E-42	145
TcMYB46	ATMYB61	58.88	107	44	0	118	438	12	118	8.00E-42	147
TcMYB46	ATMYB80	56.19	105	46	0	115	429	11	115	1.00E-41	145
TcMYB46	ATMYB67	49.28	138	69	2	106	516	18	152	1.00E-41	145
TcMYB46	AtMYB82	52.14	117	53	1	106	447	8	124	2.00E-41	142
TcMYB46	AtMYB42	58.65	104	43	0	118	429	12	115	2.00E-41	144
TcMYB46	ATMYB111	57.69	104	44	0	118	429	12	115	2.00E-41	145
TcMYB46	ATMYB0	57.28	103	44	0	121	429	15	117	2.00E-41	142
TcMYB46	ATMYB72	40	190	102	4	118	651	14	199	4.00E-41	144
TcMYB46	AtMYB83	42.24	161	90	2	94	567	22	169	4.00E-41	145
TcMYB46	ATMYB95	57.69	104	44	0	118	429	12	115	5.00E-41	142
TcMYB46	AtMYB112	52.83	106	50	0	118	435	32	137	7.00E-41	141
TcMYB46	ATMYB122	45.96	161	73	3	118	558	12	172	9.00E-41	144
TcMYB46	AtMYB108	54.21	107	49	0	118	438	19	125	1.00E-40	143
TcMYB46	ATMYB12	55.77	104	46	0	118	429	12	115	1.00E-40	144
TcMYB46	ATMYB59-3	54.87	113	50	1	121	456	9	121	1.00E-40	140
TcMYB46	ATMYB2	54.72	106	48	0	118	435	20	125	2.00E-40	141
TcMYB46	AtMYB36	47.22	144	71	3	115	531	11	146	2.00E-40	142
TcMYB46	ATMYB123	54.81	104	47	0	118	429	14	117	3.00E-40	140
TcMYB46	ATMYB68	32.31	294	175	7	115	924	11	295	3.00E-40	143
TcMYB46	ATMYB35	54.29	105	48	0	115	429	11	115	3.00E-40	142
TcMYB46	ATMYB48	46.48	142	75	2	121	543	8	147	3.00E-40	140
TcMYB46	ATMYB11	54.81	104	47	0	118	429	12	115	4.00E-40	142
TcMYB46	ATMYB55	55.08	118	41	1	118	435	12	129	9.00E-40	141
TcMYB46	ATMYB84	58.49	106	43	1	115	429	11	116	1.00E-39	140
TcMYB46	AtMYB40	48.15	135	67	2	118	513	12	146	1.00E-39	139
TcMYB46	AtMYB10	56.73	104	45	0	118	429	14	117	1.00E-39	138
TcMYB46	AtMYB103	55.14	107	48	0	118	438	12	118	2.00E-39	141
TcMYB46	AtMYB47	40.7	172	99	3	118	624	12	180	8.00E-39	136
TcMYB46	ATMYB26	52.21	113	45	1	118	429	12	124	2.00E-38	138
TcMYB46	ATMYB38	48.12	133	68	2	115	510	11	142	2.00E-38	136
TcMYB46	AtMYB114	48.6	107	55	0	109	429	5	111	2.00E-38	131
TcMYB46	AtMYB104	52.99	117	55	0	124	474	18	134	2.00E-38	138
TcMYB46	AtMYB45	37.69	199	115	4	121	690	19	205	9.00E-38	133
TcMYB46	ATMYB37	37.25	204	125	5	118	720	12	190	9.00E-38	135
TcMYB46	AtMYB27	53.77	106	49	0	115	432	8	113	1.00E-36	129
TcMYB46	ATMYB75	47.66	107	56	0	109	429	5	111	2.00E-36	129
TcMYB46	ATMYB78	46.28	121	51	1	118	438	26	146	5.00E-36	130

TcMYB46	AtMYB113	48.08	104	54	0	118	429	8	111	1.00E-35	127
TcMYB46	AtMYB19	52.29	109	52	0	121	447	13	121	1.00E-35	127
TcMYB46	ATMYB90	47.66	107	56	0	109	429	5	111	3.00E-35	126
TcMYB46	AtMYB18	50.49	103	51	0	121	429	11	113	9.00E-33	120
TcMYB46	AtMYB98	45.93	135	72	4	31	432	195	317	1.00E-31	120
TcMYB46	AtMYB1	44.97	149	69	3	22	429	9	155	1.00E-31	119
TcMYB46	AtMYB70	53.92	102	47	1	124	429	13	113	4.00E-31	116
TcMYB46	ATMYB119	50.46	109	53	3	109	432	100	205	1.00E-30	117
TcMYB46	ATMYB73	51.96	102	49	1	124	429	13	113	1.00E-30	115
TcMYB46	ATMYB118	44.53	137	76	2	115	525	186	320	2.00E-30	116
TcMYB46	AtMYB109	50.44	113	55	2	94	429	46	156	6.00E-30	114
TcMYB46	AtMYB115	43.48	138	76	4	19	426	129	257	8.00E-30	113
TcMYB46	AtMYB64	50	104	51	3	124	432	105	205	3.00E-29	112
TcMYB46	ATMYB77	47.66	107	56	1	124	444	6	111	2.00E-28	108
TcMYB46	ATMYB44	50	102	51	1	124	429	6	106	2.00E-28	108
TcMYB46	AtMYB100	49.07	108	54	2	118	438	24	129	3.00E-28	105
TcMYB46	ATMYB25	49.54	109	54	2	106	429	44	150	4.00E-28	108
TcMYB46	AtMYB117	41.73	139	75	3	55	453	70	206	1.00E-27	107
TcMYB46	ATMYB105	45.22	115	62	2	97	438	98	210	1.00E-27	106
TcMYB46	AtMYB56	48.57	105	54	1	124	438	93	196	1.00E-27	106
TcMYB46	ATMYB52	46.96	115	60	2	124	465	5	117	1.00E-27	104
TcMYB46	ATMYB69	45.87	109	58	2	115	438	16	122	2.00E-27	103
TcMYB46	ATMYB54	46.23	106	56	2	124	438	6	109	2.00E-26	100
TcMYB46	ATMYB110	42.19	128	74	2	55	438	48	170	5.00E-26	101
TcMYB46	AtMYB22	40.3	134	80	3	37	438	30	156	4.00E-25	97.4
TcMYB46	ATMYB91	40.38	104	60	1	133	438	7	110	2.00E-23	94.7
TcMYB46	AtMYB89	36.09	133	82	3	127	516	57	178	3.00E-19	78.6
TcMYB46	AtMYB124	34.65	101	66	1	133	435	28	127	4.00E-17	76.3
TcMYB46	ATMYB88	31.68	101	69	1	133	435	33	132	4.00E-15	70.1
TcMYB47	AtMYB79	53.11	177	73	2	28	528	7	181	7.00E-62	188
TcMYB47	ATMYB3	65.57	122	42	0	16	381	17	138	2.00E-61	186
TcMYB47	ATMYB71	79.61	103	21	0	28	336	19	121	7.00E-61	186
TcMYB47	AtMYB62	60.9	133	47	1	7	390	13	145	2.00E-60	186
TcMYB47	AtMYB112	63.91	133	43	2	7	390	26	158	2.00E-59	182
TcMYB47	AtMYB24	74.04	104	27	0	22	333	16	119	2.00E-59	181
TcMYB47	AtMYB116	42.67	232	121	5	10	669	13	243	2.00E-59	182
TcMYB47	ATMYB57	62.5	128	45	2	7	381	19	146	2.00E-57	175
TcMYB47	ATMYB2	56.39	133	52	2	10	390	15	146	3.00E-57	177
TcMYB47	ATMYB121	56.05	157	60	3	1	444	17	172	4.00E-57	177
TcMYB47	ATMYB59-3	64.04	114	41	0	7	348	2	115	7.00E-56	172
TcMYB47	AtMYB108	68.52	108	34	0	10	333	14	121	7.00E-56	175
TcMYB47	ATMYB48	62.4	125	46	2	10	381	3	126	3.00E-55	171
TcMYB47	ATMYB78	64.75	122	29	1	10	333	21	142	2.00E-54	171
TcMYB47	ATMYB3	48.62	181	80	2	13	516	8	188	2.00E-54	169
TcMYB47	ATMYB66	48.24	170	84	3	19	516	14	176	2.00E-52	162
TcMYB47	AtMYB27	41.13	231	122	6	4	654	3	231	1.00E-51	161
TcMYB47	MYB8	64.04	114	41	0	13	354	8	121	5.00E-51	159
TcMYB47	ATMYB13	63.79	116	39	1	13	351	8	123	6.00E-51	160
TcMYB47	MYB7	64.91	114	40	0	13	354	8	121	2.00E-50	159
TcMYB47	ATMYB4	65.79	114	39	0	13	354	8	121	2.00E-50	159
TcMYB47	ATMYB14	64.22	109	39	0	13	339	8	116	4.00E-50	158
TcMYB47	ATMYB23	62.39	109	41	0	13	339	8	116	6.00E-50	156
TcMYB47	AtMYB6	63.25	117	43	0	13	363	8	124	7.00E-50	157
TcMYB47	AtMYB49	66.97	109	36	0	13	339	8	116	9.00E-50	159
TcMYB47	AtMYB32	66.97	109	36	0	13	339	8	116	1.00E-49	157
TcMYB47	AtMYB107	65.14	109	38	0	13	339	8	116	4.00E-49	157
TcMYB47	AtMYB9	65.14	109	38	0	13	339	8	116	1.00E-48	157
TcMYB47	AtMYB85	65.71	105	36	0	25	339	12	116	2.00E-48	154
TcMYB47	ATMYB86	43.63	204	111	5	16	615	9	200	2.00E-48	156
TcMYB47	AtMYB74	63.64	110	40	0	10	339	8	117	2.00E-48	155
TcMYB47	ATMYB12	63.96	111	40	0	13	345	8	118	3.00E-48	156
TcMYB47	AtMYB41	63.3	109	40	0	13	339	8	116	4.00E-48	154
TcMYB47	ATMYB5	65.71	105	36	0	25	339	23	127	7.00E-48	152
TcMYB47	AtMYB42	65.71	105	36	0	25	339	12	116	9.00E-48	153
TcMYB47	ATMYB102	64.22	109	39	0	13	339	8	116	2.00E-47	154
TcMYB47	ATMYB0	58.56	111	46	0	7	339	8	118	2.00E-47	150
TcMYB47	ATMYB61	61.54	117	45	1	4	354	8	121	2.00E-47	154
TcMYB47	ATMYB11	65.14	109	38	0	13	339	8	116	4.00E-47	153
TcMYB47	AtMYB20	63.21	106	39	0	25	342	12	117	4.00E-47	151
TcMYB47	AtMYB51	65.71	105	36	0	25	339	13	117	1.00E-46	152
TcMYB47	AtMYB51	30.16	63	43	3	22	207	65	124	8.00E-04	32.3
TcMYB47	ATMYB15	59.46	111	45	0	13	345	8	118	1.00E-46	150
TcMYB47	AtMYB50	59.83	117	47	1	4	354	8	121	1.00E-46	150
TcMYB47	ATMYB92	58.72	109	45	0	13	339	8	116	2.00E-46	151
TcMYB47	ATMYB46	48.43	159	82	1	22	498	17	169	2.00E-46	149
TcMYB47	ATMYB34	58.77	114	47	0	13	354	8	121	2.00E-46	150
TcMYB47	ATMYB101	59.26	108	44	0	13	336	14	121	2.00E-46	154
TcMYB47	AtMYB82	54.03	124	55	1	7	372	4	127	4.00E-46	146
TcMYB47	ATMYB123	62.26	106	40	0	22	339	13	118	1.00E-45	147
TcMYB47	AtMYB83	56.45	124	54	1	22	393	29	149	1.00E-45	149

TcMYB47	ATMYB106	64.76	105	37	0	25	339	55	159	2.00E-45	149
TcMYB47	AtMYB97	62.14	103	39	0	25	333	19	121	3.00E-45	149
TcMYB47	AtMYB43	61.32	106	41	0	25	342	12	117	3.00E-45	147
TcMYB47	AtMYB93	61.47	109	42	0	13	339	8	116	5.00E-45	148
TcMYB47	AtMYB76	55.2	125	55	1	25	396	12	136	5.00E-45	147
TcMYB47	ATMYB111	61.47	109	42	0	13	339	8	116	6.00E-45	147
TcMYB47	ATMYB67	48.15	135	69	1	16	417	19	153	7.00E-45	146
TcMYB47	AtMYB103	53.17	126	59	1	16	393	9	129	7.00E-45	147
TcMYB47	AtMYB53	59.63	109	44	0	13	339	8	116	8.00E-45	146
TcMYB47	ATMYB16	63.81	105	38	0	25	339	12	116	8.00E-45	146
TcMYB47	AtMYB10	46.1	154	83	1	13	474	10	161	1.00E-44	144
TcMYB47	ATMYB29	60	110	44	0	25	354	12	121	1.00E-44	146
TcMYB47	ATMYB63	58.72	109	45	0	13	339	10	118	1.00E-44	145
TcMYB47	ATMYB31	60.55	109	43	0	13	339	8	116	2.00E-44	145
TcMYB47	ATMYB35	56.88	109	47	0	13	339	8	116	2.00E-44	145
TcMYB47	AtMYB17	59.09	110	45	0	25	354	12	121	2.00E-44	144
TcMYB47	ATMYB94	58.72	109	45	0	25	351	12	120	3.00E-44	145
TcMYB47	ATMYB28	58.18	110	46	0	25	354	12	121	3.00E-44	146
TcMYB47	ATMYB95	58.18	110	46	0	25	354	12	121	3.00E-44	143
TcMYB47	ATMYB99	58.62	116	41	1	13	339	9	124	3.00E-44	142
TcMYB47	AtMYB60	61.9	105	40	0	25	339	12	116	4.00E-44	143
TcMYB47	ATMYB96	57.52	113	48	0	13	351	8	120	1.00E-43	144
TcMYB47	ATMYB58	56.88	109	47	0	13	339	10	118	1.00E-43	142
TcMYB47	ATMYB80	40.2	204	119	4	13	615	8	191	1.00E-43	143
TcMYB47	ATMYB72	57.8	109	46	0	13	339	10	118	2.00E-43	142
TcMYB47	ATMYB120	59.43	106	43	0	25	342	26	131	2.00E-43	147
TcMYB47	ATMYB26	53.17	126	50	1	16	366	9	134	2.00E-43	144
TcMYB47	AtMYB40	41.62	185	103	3	25	564	12	181	3.00E-43	140
TcMYB47	ATMYB30	53.33	120	56	0	13	372	8	127	3.00E-43	142
TcMYB47	ATMYB122	59.09	110	45	0	25	354	12	121	4.00E-43	142
TcMYB47	AtMYB81	56.76	111	44	1	13	333	12	122	5.00E-43	144
TcMYB47	ATMYB33	58.65	104	43	0	25	336	32	135	9.00E-43	145
TcMYB47	ATMYB65	56.73	104	45	0	25	336	41	144	2.00E-42	144
TcMYB47	ATMYB55	54.26	129	47	2	4	354	8	133	2.00E-41	138
TcMYB47	ATMYB87	59.43	106	42	1	25	339	12	117	5.00E-41	136
TcMYB47	ATMYB37	47.37	133	69	1	13	408	8	140	4.00E-40	134
TcMYB47	AtMYB36	46.48	142	73	2	13	429	8	149	2.00E-39	132
TcMYB47	AtMYB19	57.28	103	44	0	28	336	13	115	2.00E-39	130
TcMYB47	AtMYB114	54.81	104	47	0	25	336	8	111	3.00E-39	126
TcMYB47	AtMYB47	52.73	110	52	0	25	354	12	121	4.00E-39	130
TcMYB47	AtMYB45	54.13	109	50	1	7	333	13	120	5.00E-39	129
TcMYB47	ATMYB84	55.45	110	48	1	13	339	8	117	1.00E-38	130
TcMYB47	AtMYB18	53.45	116	53	1	28	372	11	126	4.00E-38	128
TcMYB47	ATMYB75	54.81	104	47	0	25	336	8	111	8.00E-38	126
TcMYB47	ATMYB68	52.21	113	53	1	13	348	8	120	2.00E-37	128
TcMYB47	AtMYB113	52.78	108	51	0	25	348	8	115	2.00E-37	125
TcMYB47	ATMYB90	54.81	104	47	0	25	336	8	111	2.00E-37	125
TcMYB47	ATMYB38	51.82	110	52	1	13	339	8	117	7.00E-37	125
TcMYB47	AtMYB104	44.44	108	60	1	31	354	18	124	5.00E-31	111
TcMYB47	AtMYB98	31.93	166	113	3	13	510	211	365	9.00E-30	108
TcMYB47	ATMYB119	31.84	223	150	6	7	669	97	309	9.00E-30	108
TcMYB47	ATMYB118	47.12	104	55	1	25	336	187	289	1.00E-29	108
TcMYB47	AtMYB64	43.48	115	64	2	7	348	97	209	4.00E-29	106
TcMYB47	AtMYB70	45.87	109	59	1	10	336	6	113	5.00E-29	104
TcMYB47	AtMYB70	25.81	62	45	1	181	363	10	71	3.00E-04	33.5
TcMYB47	ATMYB77	43.14	102	58	1	31	336	6	106	2.00E-28	102
TcMYB47	ATMYB73	46.08	102	55	1	31	336	13	113	3.00E-28	102
TcMYB47	ATMYB73	30.51	59	40	2	190	363	13	70	6.00E-04	32.7
TcMYB47	ATMYB25	38.03	142	84	2	31	444	50	190	3.00E-28	103
TcMYB47	AtMYB109	40.46	131	78	2	31	423	56	183	2.00E-27	101
TcMYB47	AtMYB1	44.76	105	58	1	31	345	55	158	2.00E-26	99
TcMYB47	AtMYB115	34.87	152	99	5	10	465	151	290	7.00E-26	97.1
TcMYB47	ATMYB44	42.16	102	59	1	31	336	6	106	1.00E-25	95.5
TcMYB47	AtMYB117	34.42	154	94	4	31	471	98	249	2.00E-25	95.9
TcMYB47	ATMYB105	40.91	110	64	2	25	351	105	212	2.00E-25	95.1
TcMYB47	AtMYB100	31.97	147	100	3	13	453	20	163	9.00E-25	91.7
TcMYB47	ATMYB52	32.8	189	117	5	31	567	5	183	2.00E-24	91.3
TcMYB47	ATMYB54	43.69	103	57	2	31	336	6	106	1.00E-23	89
TcMYB47	ATMYB54	26.03	73	45	2	190	381	6	78	4.00E-05	36.2
TcMYB47	ATMYB110	34.58	107	70	1	31	351	67	172	4.00E-23	88.6
TcMYB47	ATMYB91	40.78	103	59	2	40	342	7	108	4.00E-23	89.4
TcMYB47	AtMYB124	36.92	130	76	2	40	411	28	156	9.00E-23	89
TcMYB47	AtMYB56	38.1	105	65	1	31	345	93	196	7.00E-22	85.5
TcMYB47	ATMYB88	38.74	111	68	1	40	372	33	142	7.00E-22	86.7
TcMYB47	ATMYB69	35.09	114	74	1	4	345	10	122	3.00E-21	82.4
TcMYB47	AtMYB89	31.85	135	85	3	7	390	48	181	6.00E-20	77.8
TcMYB47	AtMYB22	31.5	127	87	3	13	393	48	171	2.00E-19	77.8
TcMYB48	ATMYB44	39.78	362	212	10	46	1113	2	285	3.00E-68	211
TcMYB48	ATMYB77	41.82	318	181	9	46	987	2	285	1.00E-65	204
TcMYB48	ATMYB73	37.96	353	219	8	49	1107	10	283	3.00E-61	193

TcMYB48	AtMYB109	45.07	213	109	5	52	666	54	257	2.00E-54	177
TcMYB48	AtMYB70	74.53	106	27	0	49	366	10	115	2.00E-53	172
TcMYB48	AtMYB1	41.63	233	125	5	49	714	52	262	1.00E-50	167
TcMYB48	ATMYB25	38.91	221	129	5	52	696	48	242	8.00E-46	154
TcMYB48	ATMYB105	48.76	121	62	0	58	420	107	227	4.00E-39	135
TcMYB48	ATMYB54	46.15	130	68	1	58	441	6	135	2.00E-38	131
TcMYB48	ATMYB69	45.52	134	69	1	58	447	19	152	7.00E-38	130
TcMYB48	AtMYB117	54.9	102	46	0	58	363	98	199	1.00E-37	132
TcMYB48	AtMYB56	36.46	192	122	1	58	633	93	278	1.00E-36	129
TcMYB48	ATMYB52	50.5	101	50	0	58	360	5	105	3.00E-36	125
TcMYB48	AtMYB98	56.73	104	45	0	55	366	216	319	3.00E-36	129
TcMYB48	ATMYB119	51.33	113	55	0	55	393	104	216	1.00E-34	125
TcMYB48	AtMYB64	35.58	208	132	4	55	672	104	298	1.00E-34	125
TcMYB48	ATMYB118	46.4	125	65	1	46	414	185	309	9.00E-34	123
TcMYB48	ATMYB46	36.22	196	116	5	58	618	20	211	1.00E-32	117
TcMYB48	AtMYB115	37.13	167	105	1	58	558	158	318	2.00E-32	118
TcMYB48	AtMYB82	39.86	148	87	3	58	495	14	155	6.00E-32	112
TcMYB48	ATMYB61	33.77	228	144	7	58	720	14	228	1.00E-31	116
TcMYB48	ATMYB14	43.61	133	73	3	58	450	14	142	1.00E-31	113
TcMYB48	ATMYB121	49.54	109	53	2	58	378	29	136	2.00E-31	113
TcMYB48	ATMYB80	47.71	109	56	1	58	381	14	122	2.00E-31	114
TcMYB48	ATMYB110	47.17	106	56	0	58	375	67	172	2.00E-31	114
TcMYB48	ATMYB72	37.7	183	105	6	58	579	16	188	3.00E-31	113
TcMYB48	AtMYB45	40.12	162	91	4	58	525	20	171	7.00E-31	111
TcMYB48	ATMYB92	51.46	103	48	2	58	360	14	115	8.00E-31	113
TcMYB48	ATMYB95	38.82	152	90	3	58	504	14	164	9.00E-31	111
TcMYB48	ATMYB37	43.7	135	74	3	58	456	14	146	1.00E-30	112
TcMYB48	ATMYB58	40.76	157	91	5	58	522	16	161	2.00E-30	110
TcMYB48	AtMYB74	42.54	134	76	1	58	456	15	148	2.00E-30	111
TcMYB48	ATMYB120	29.85	268	181	7	58	840	28	283	3.00E-30	114
TcMYB48	AtMYB41	44.8	125	68	1	58	429	14	138	3.00E-30	110
TcMYB48	AtMYB17	39.1	156	93	4	58	519	14	160	3.00E-30	110
TcMYB48	ATMYB35	30.65	248	151	6	58	738	14	261	3.00E-30	111
TcMYB48	ATMYB67	30.26	228	152	6	58	720	24	227	3.00E-30	110
TcMYB48	ATMYB59-3	31.58	228	151	7	58	726	10	202	4.00E-30	108
TcMYB48	AtMYB36	38.89	144	86	3	58	483	14	151	4.00E-30	111
TcMYB48	ATMYB101	35.1	208	107	6	58	597	20	214	4.00E-30	113
TcMYB48	AtMYB97	53.47	101	46	1	58	357	21	121	7.00E-30	111
TcMYB48	ATMYB86	35.35	215	125	6	58	660	14	223	7.00E-30	110
TcMYB48	AtMYB50	35.26	190	120	3	58	618	14	202	1.00E-29	109
TcMYB48	ATMYB48	37.76	143	86	3	58	477	9	150	1.00E-29	108
TcMYB48	AtMYB27	39.38	160	90	5	58	516	11	161	2.00E-29	107
TcMYB48	ATMYB13	40.54	148	86	3	58	495	14	159	2.00E-29	107
TcMYB48	ATMYB102	40.43	141	83	1	58	477	14	154	2.00E-29	109
TcMYB48	ATMYB34	47.71	109	56	1	58	381	14	122	2.00E-29	108
TcMYB48	AtMYB19	41.43	140	80	3	58	471	14	144	2.00E-29	107
TcMYB48	AtMYB107	49.02	102	51	1	58	360	14	115	3.00E-29	108
TcMYB48	AtMYB79	46.49	114	59	3	58	393	8	116	3.00E-29	107
TcMYB48	ATMYB63	32.43	222	117	6	58	624	16	236	3.00E-29	107
TcMYB48	ATMYB28	31.71	246	166	6	58	789	14	233	4.00E-29	108
TcMYB48	AtMYB51	45.87	109	58	1	58	381	15	123	5.00E-29	108
TcMYB48	AtMYB83	48.31	118	60	2	58	408	32	147	6.00E-29	108
TcMYB48	ATMYB26	48.25	114	49	2	58	369	14	127	6.00E-29	108
TcMYB48	ATMYB65	50.98	102	49	1	58	360	43	144	7.00E-29	110
TcMYB48	AtMYB53	49.51	103	50	2	58	360	14	115	9.00E-29	107
TcMYB48	ATMYB106	29.96	257	169	6	58	795	57	283	1.00E-28	108
TcMYB48	ATMYB3	32.18	202	136	5	40	642	16	199	1.00E-28	104
TcMYB48	AtMYB112	35.12	168	106	2	22	516	22	189	1.00E-28	105
TcMYB48	AtMYB60	43.36	113	59	2	58	381	14	122	1.00E-28	105
TcMYB48	ATMYB87	29.05	241	154	5	58	729	14	244	2.00E-28	106
TcMYB48	ATMYB3	44.35	115	63	1	58	399	14	128	2.00E-28	105
TcMYB48	ATMYB33	50.98	102	49	1	58	360	34	135	3.00E-28	108
TcMYB48	AtMYB9	49.02	102	51	1	58	360	14	115	3.00E-28	106
TcMYB48	AtMYB6	35.29	170	100	4	58	537	14	183	3.00E-28	103
TcMYB48	ATMYB15	33.7	184	117	4	58	594	14	192	3.00E-28	105
TcMYB48	ATMYB16	37.42	163	101	2	58	543	14	170	3.00E-28	105
TcMYB48	AtMYB49	38.73	142	86	1	58	480	14	155	4.00E-28	105
TcMYB48	ATMYB71	29.7	266	176	9	58	822	20	258	4.00E-28	104
TcMYB48	AtMYB116	44.35	115	63	2	46	387	16	126	5.00E-28	104
TcMYB48	MYB8	44.95	109	59	1	58	381	14	122	6.00E-28	102
TcMYB48	ATMYB30	38.36	146	88	3	58	489	14	150	8.00E-28	104
TcMYB48	AtMYB10	41.94	124	65	3	58	408	16	138	1.00E-27	102
TcMYB48	AtMYB10	25.61	82	61	1	58	303	69	149	7.00E-04	33.1
TcMYB48	AtMYB103	42.28	123	70	1	58	423	14	136	1.00E-27	104
TcMYB48	AtMYB81	31.44	264	180	11	58	846	22	238	2.00E-27	105
TcMYB48	ATMYB2	35.63	174	105	5	58	558	22	191	2.00E-27	102
TcMYB48	ATMYB122	46.53	101	53	1	58	357	14	114	2.00E-27	103
TcMYB48	AtMYB40	33.73	166	108	4	58	549	14	169	2.00E-27	102
TcMYB48	AtMYB47	37.5	152	90	3	58	498	14	164	2.00E-27	102
TcMYB48	AtMYB32	40.26	154	90	4	58	513	14	159	2.00E-27	102

TcMYB48	ATMYB66	38.85	139	78	4	58	453	18	154	2.00E-27	100
TcMYB48	AtMYB100	40.71	113	65	1	67	399	29	141	3.00E-27	101
TcMYB48	ATMYB55	46.96	115	47	2	58	360	14	127	3.00E-27	103
TcMYB48	AtMYB93	48.04	102	52	1	58	360	14	115	4.00E-27	103
TcMYB48	ATMYB68	48.54	103	51	2	58	360	14	116	4.00E-27	103
TcMYB48	ATMYB84	47.57	103	52	2	58	360	14	116	4.00E-27	102
TcMYB48	AtMYB18	40.85	142	80	3	58	471	12	143	5.00E-27	101
TcMYB48	ATMYB96	41.8	122	66	2	58	408	14	131	6.00E-27	102
TcMYB48	AtMYB89	43.43	99	56	0	61	357	57	155	6.00E-27	99
TcMYB48	ATMYB38	31.53	222	126	5	58	645	14	233	6.00E-27	101
TcMYB48	MYB7	35.48	155	99	2	58	519	14	167	7.00E-27	100
TcMYB48	ATMYB4	45.87	109	58	1	58	381	14	122	9.00E-27	100
TcMYB48	ATMYB123	45.05	111	59	2	34	360	8	117	2.00E-26	99.8
TcMYB48	ATMYB31	36.69	139	66	3	58	408	14	148	2.00E-26	100
TcMYB48	AtMYB24	44.76	105	57	1	46	357	15	119	2.00E-26	98.2
TcMYB48	ATMYB57	40.71	113	66	1	58	393	27	139	2.00E-26	97.8
TcMYB48	AtMYB42	38.81	134	80	2	58	453	14	146	3.00E-26	99.8
TcMYB48	ATMYB23	45.63	103	54	2	58	360	14	115	3.00E-26	97.8
TcMYB48	ATMYB111	36.59	164	102	3	58	543	14	172	5.00E-26	100
TcMYB48	ATMYB94	36.11	144	84	3	58	465	14	153	5.00E-26	99.8
TcMYB48	AtMYB62	44.64	112	60	3	58	387	21	127	7.00E-26	98.6
TcMYB48	ATMYB11	48.04	102	52	1	58	360	14	115	9.00E-26	99.4
TcMYB48	ATMYB0	36.49	148	89	3	58	486	16	158	1.00E-25	96.7
TcMYB48	ATMYB5	42.62	122	62	3	58	399	25	145	2.00E-25	96.7
TcMYB48	AtMYB76	44.04	109	60	1	58	381	14	122	2.00E-25	98.2
TcMYB48	AtMYB108	35.48	155	93	4	58	501	21	174	2.00E-25	97.8
TcMYB48	AtMYB114	41.75	103	59	1	58	363	10	112	4.00E-25	92.4
TcMYB48	ATMYB29	46.53	101	53	1	58	357	14	114	5.00E-25	97.1
TcMYB48	AtMYB20	44.66	103	55	2	58	360	14	115	8.00E-25	95.5
TcMYB48	AtMYB85	44.66	103	55	2	58	360	14	115	2.00E-24	94.4
TcMYB48	ATMYB12	29.9	194	135	2	58	636	14	205	4.00E-24	95.1
TcMYB48	AtMYB104	44.12	102	55	2	58	357	18	118	6.00E-24	94.7
TcMYB48	AtMYB113	33.99	153	94	4	58	495	10	152	1.00E-23	91.7
TcMYB48	AtMYB43	31.79	173	116	4	58	570	14	177	1.00E-23	92.8
TcMYB48	ATMYB90	40.95	105	61	1	58	369	10	114	1.00E-23	91.3
TcMYB48	AtMYB22	33.83	133	86	2	7	399	37	168	2.00E-23	91.3
TcMYB48	ATMYB75	41.12	107	58	2	58	363	10	112	2.00E-23	90.9
TcMYB48	AtMYB124	31.78	129	88	0	67	453	28	156	4.00E-22	89.7
TcMYB48	AtMYB124	19.88	171	129	5	58	546	77	237	2.00E-04	35
TcMYB48	ATMYB78	31.16	199	111	6	58	576	28	226	5.00E-22	88.2
TcMYB48	ATMYB88	37.86	103	64	0	67	375	33	135	5.00E-22	89.7
TcMYB48	ATMYB88	32.08	53	36	1	58	216	82	133	7.00E-04	33.5
TcMYB48	ATMYB99	39.52	124	63	4	58	393	15	134	2.00E-21	85.1
TcMYB48	ATMYB91	32.71	107	69	1	67	378	7	113	6.00E-16	70.9
TcMYB49	AtMYB109	42.09	335	188	9	10	996	47	335	8.00E-75	233
TcMYB49	ATMYB25	35.04	391	247	11	13	1164	42	362	3.00E-62	199
TcMYB49	AtMYB1	37.99	329	203	7	7	990	46	325	4.00E-62	199
TcMYB49	ATMYB73	73.08	104	28	0	28	339	10	113	8.00E-52	170
TcMYB49	ATMYB44	69.72	109	33	0	28	354	3	111	1.00E-51	169
TcMYB49	AtMYB70	71.96	107	30	0	28	348	10	116	3.00E-51	168
TcMYB49	ATMYB77	68.22	107	34	0	28	348	3	109	4.00E-50	165
TcMYB49	ATMYB54	56.44	101	44	0	37	339	6	106	1.00E-39	135
TcMYB49	ATMYB52	55.45	101	45	0	37	339	5	105	1.00E-38	133
TcMYB49	AtMYB117	54.9	102	46	0	37	342	98	199	2.00E-37	133
TcMYB49	ATMYB69	54.46	101	46	0	37	339	19	119	2.00E-37	130
TcMYB49	ATMYB105	53.92	102	47	0	37	342	107	208	6.00E-37	130
TcMYB49	ATMYB118	48.48	132	68	0	34	429	188	319	2.00E-36	132
TcMYB49	AtMYB56	54.46	101	46	0	37	339	93	193	4.00E-36	128
TcMYB49	AtMYB98	53.4	103	48	0	34	342	216	318	9.00E-36	129
TcMYB49	ATMYB110	51.49	101	49	0	37	339	67	167	9.00E-34	121
TcMYB49	AtMYB51	42.66	143	79	3	37	456	15	156	2.00E-33	122
TcMYB49	AtMYB115	36.36	154	98	1	37	498	158	310	3.00E-33	121
TcMYB49	ATMYB119	39.6	149	79	2	34	447	104	251	3.00E-33	122
TcMYB49	ATMYB121	38.1	189	106	5	37	570	29	207	5.00E-33	119
TcMYB49	AtMYB64	50.98	102	50	0	34	339	104	205	9.00E-33	121
TcMYB49	ATMYB59-3	49.53	107	52	2	25	339	6	111	7.00E-32	114
TcMYB49	ATMYB59-3	21.49	121	95	2	37	399	63	175	1.00E-04	35.8
TcMYB49	AtMYB27	41.29	155	87	4	37	489	11	164	7.00E-32	114
TcMYB49	ATMYB66	37.25	204	109	7	7	561	8	199	1.00E-31	113
TcMYB49	ATMYB48	51.46	103	48	2	37	339	9	110	1.00E-31	114
TcMYB49	ATMYB48	31.48	54	37	1	37	198	62	114	3.00E-04	34.7
TcMYB49	AtMYB19	39.63	164	97	3	4	489	3	162	4.00E-31	113
TcMYB49	AtMYB17	49.53	107	52	2	37	351	14	119	5.00E-31	114
TcMYB49	ATMYB63	39.24	158	87	3	37	483	16	169	9.00E-31	113
TcMYB49	AtMYB45	39.74	156	92	3	25	486	16	160	1.00E-30	112
TcMYB49	AtMYB36	50.46	109	52	2	37	357	14	122	3.00E-30	112
TcMYB49	ATMYB96	44.78	134	65	3	37	411	14	143	3.00E-30	112
TcMYB49	AtMYB107	50.46	109	51	2	37	354	14	122	4.00E-30	112
TcMYB49	AtMYB47	47.37	114	57	2	37	369	14	127	5.00E-30	110
TcMYB49	ATMYB15	39.88	168	94	4	37	519	14	180	5.00E-30	110

TcMYB49	ATMYB31	41.91	136	74	3	37	429	14	144	6.00E-30	111
TcMYB49	AtMYB9	51.38	109	50	2	37	354	14	122	7.00E-30	111
TcMYB49	AtMYB10	37.91	153	93	3	37	489	16	162	1.00E-29	108
TcMYB49	ATMYB101	37.35	166	96	4	37	510	20	184	1.00E-29	113
TcMYB49	ATMYB95	46.67	120	61	2	37	387	14	133	1.00E-29	109
TcMYB49	AtMYB89	45.45	99	54	0	40	336	57	155	1.00E-29	107
TcMYB49	ATMYB30	41.55	142	77	3	37	444	14	151	1.00E-29	110
TcMYB49	AtMYB74	49.54	109	52	2	37	354	15	123	1.00E-29	110
TcMYB49	ATMYB5	47.29	129	64	3	37	411	25	153	2.00E-29	108
TcMYB49	AtMYB93	50.46	109	51	2	37	354	14	122	2.00E-29	110
TcMYB49	AtMYB60	50.94	106	47	2	37	339	14	115	2.00E-29	108
TcMYB49	ATMYB3	50.46	109	51	2	37	354	14	122	3.00E-29	108
TcMYB49	ATMYB67	40.26	154	87	5	22	468	18	165	3.00E-29	109
TcMYB49	AtMYB97	41.67	132	75	2	4	393	10	141	3.00E-29	110
TcMYB49	AtMYB53	50.48	105	50	2	37	345	14	117	4.00E-29	108
TcMYB49	AtMYB81	46.49	114	59	2	37	372	22	135	5.00E-29	110
TcMYB49	ATMYB34	48.33	120	58	3	37	384	14	133	6.00E-29	108
TcMYB49	AtMYB50	49.59	123	57	4	37	390	14	135	6.00E-29	108
TcMYB49	ATMYB14	34.07	182	115	5	37	567	14	183	8.00E-29	106
TcMYB49	ATMYB72	44.44	126	68	3	37	408	16	139	8.00E-29	107
TcMYB49	ATMYB94	49.06	106	49	2	37	339	14	115	8.00E-29	108
TcMYB49	AtMYB41	47.71	109	54	2	37	354	14	122	8.00E-29	107
TcMYB49	ATMYB35	34.59	185	107	4	37	549	14	195	8.00E-29	108
TcMYB49	AtMYB18	48.18	110	55	2	22	345	7	115	8.00E-29	107
TcMYB49	ATMYB92	47.62	105	53	2	37	345	14	117	1.00E-28	108
TcMYB49	ATMYB80	46.23	106	56	1	37	351	14	119	1.00E-28	107
TcMYB49	ATMYB106	45.31	128	63	3	37	399	57	184	1.00E-28	108
TcMYB49	ATMYB102	49.54	109	52	2	37	354	14	122	1.00E-28	108
TcMYB49	ATMYB122	40	140	81	2	37	447	14	153	2.00E-28	107
TcMYB49	AtMYB49	45	120	63	2	4	354	3	122	2.00E-28	107
TcMYB49	ATMYB87	50.48	105	50	2	37	345	14	118	2.00E-28	107
TcMYB49	ATMYB84	39.19	148	80	4	37	450	14	159	2.00E-28	107
TcMYB49	ATMYB65	50	102	50	1	37	339	43	144	2.00E-28	110
TcMYB49	MYB8	48.62	109	53	2	37	354	14	122	2.00E-28	104
TcMYB49	ATMYB120	50.5	101	49	1	37	336	28	128	2.00E-28	109
TcMYB49	ATMYB13	40.82	147	80	4	37	456	14	149	3.00E-28	105
TcMYB49	AtMYB116	42.86	119	65	2	7	354	10	128	3.00E-28	105
TcMYB49	ATMYB55	39.62	159	76	4	37	453	14	171	4.00E-28	107
TcMYB49	AtMYB24	45.95	111	58	2	10	336	9	119	5.00E-28	103
TcMYB49	ATMYB37	50.48	105	50	2	37	345	14	118	5.00E-28	106
TcMYB49	ATMYB23	46.85	111	57	2	13	339	6	115	5.00E-28	103
TcMYB49	AtMYB6	38.41	164	88	4	37	489	14	177	6.00E-28	103
TcMYB49	ATMYB4	43.88	139	72	4	37	435	14	151	6.00E-28	105
TcMYB49	AtMYB83	40.99	161	94	3	10	489	23	175	6.00E-28	106
TcMYB49	ATMYB33	50	102	50	1	37	339	34	135	8.00E-28	108
TcMYB49	ATMYB16	44.53	128	62	3	37	393	14	141	1.00E-27	105
TcMYB49	ATMYB46	44.07	118	65	1	37	387	20	137	2.00E-27	103
TcMYB49	ATMYB38	49.52	105	51	2	37	345	14	118	2.00E-27	103
TcMYB49	AtMYB104	44.35	115	61	3	37	372	18	131	2.00E-27	105
TcMYB49	MYB7	36.99	173	95	4	37	513	14	186	2.00E-27	103
TcMYB49	AtMYB82	47.57	103	52	2	37	339	14	115	2.00E-27	101
TcMYB49	ATMYB68	40.56	143	83	4	37	459	14	151	2.00E-27	105
TcMYB49	AtMYB42	38.19	144	87	3	37	462	14	151	3.00E-27	103
TcMYB49	ATMYB71	48.54	103	51	2	37	339	20	121	4.00E-27	102
TcMYB49	ATMYB2	49.02	102	51	1	37	339	22	123	4.00E-27	102
TcMYB49	AtMYB32	43.8	137	66	3	25	402	10	146	4.00E-27	102
TcMYB49	AtMYB103	48.54	103	51	2	37	339	14	115	8.00E-27	103
TcMYB49	ATMYB0	46.23	106	55	2	37	348	16	120	8.00E-27	100
TcMYB49	ATMYB61	50.49	103	49	2	37	339	14	115	1.00E-26	103
TcMYB49	ATMYB61	31.48	54	37	1	37	198	67	119	6.00E-04	33.9
TcMYB49	ATMYB26	32.46	228	139	6	37	675	14	237	1.00E-26	103
TcMYB49	ATMYB3	36.42	151	94	2	7	453	12	162	1.00E-26	100
TcMYB49	ATMYB123	45.79	107	56	2	25	339	12	117	1.00E-26	100
TcMYB49	ATMYB86	49.51	103	50	2	37	339	14	115	1.00E-26	102
TcMYB49	AtMYB40	45.45	110	56	3	37	354	14	122	1.00E-26	100
TcMYB49	ATMYB28	44.44	135	67	4	22	402	9	139	1.00E-26	102
TcMYB49	AtMYB100	33.56	149	87	2	46	456	29	177	2.00E-26	99.8
TcMYB49	AtMYB79	46.6	103	53	2	37	339	8	109	2.00E-26	100
TcMYB49	ATMYB57	47.52	101	52	1	37	336	27	127	2.00E-26	98.6
TcMYB49	AtMYB76	40.79	152	82	5	22	453	9	155	3.00E-26	101
TcMYB49	AtMYB20	36.42	151	92	3	37	477	14	163	3.00E-26	100
TcMYB49	ATMYB58	41.67	132	76	1	37	429	16	147	4.00E-26	99.8
TcMYB49	ATMYB29	42.45	139	63	3	22	387	9	143	2.00E-25	99
TcMYB49	AtMYB85	46.36	110	55	3	37	354	14	122	2.00E-25	97.4
TcMYB49	AtMYB114	41.75	103	59	1	37	342	10	112	3.00E-25	93.6
TcMYB49	ATMYB111	48.04	102	52	1	37	339	14	115	1.00E-24	96.7
TcMYB49	ATMYB99	46.28	121	50	5	37	354	15	130	2.00E-24	94.4
TcMYB49	ATMYB12	47.06	102	53	1	37	339	14	115	2.00E-24	96.7
TcMYB49	AtMYB62	40.87	115	65	3	4	339	9	122	2.00E-24	95.1
TcMYB49	ATMYB11	46.67	105	55	1	37	348	14	118	3.00E-24	95.9

TcMYB49	AtMYB43	36.24	149	86	4	37	456	14	161	5.00E-24	94.7
TcMYB49	ATMYB90	41.75	103	59	1	37	342	10	112	7.00E-24	92.8
TcMYB49	ATMYB75	40.78	103	60	1	37	342	10	112	9.00E-24	92.4
TcMYB49	AtMYB113	41.75	103	59	1	37	342	10	112	1.00E-23	92
TcMYB49	AtMYB108	41.74	115	64	3	1	336	8	121	4.00E-23	92
TcMYB49	AtMYB112	40.59	101	59	1	37	336	34	134	8.00E-23	89.7
TcMYB49	AtMYB22	32.47	154	104	4	37	498	54	199	1.00E-22	89.4
TcMYB49	AtMYB22	20.11	184	134	6	187	699	52	232	3.00E-04	34.7
TcMYB49	ATMYB88	37.17	113	71	0	1	339	18	130	6.00E-22	90.5
TcMYB49	AtMYB124	37.07	116	69	1	4	339	10	125	1.00E-21	89.4
TcMYB49	ATMYB91	32.26	155	96	3	46	483	7	161	4.00E-20	84
TcMYB49	ATMYB78	39.13	115	55	2	37	336	28	142	5.00E-20	83.2
TcMYB50	ATMYB4	74.67	150	28	1	1	420	1	150	1.00E-77	235
TcMYB50	AtMYB6	85.34	116	17	0	1	348	1	116	3.00E-73	222
TcMYB50	AtMYB32	70	150	44	2	1	447	1	147	7.00E-73	223
TcMYB50	ATMYB3	72.41	145	40	1	1	435	1	142	2.00E-72	221
TcMYB50	MYB7	84.48	116	18	0	1	348	1	116	5.00E-72	220
TcMYB50	MYB8	78.45	116	25	0	1	348	1	116	5.00E-68	208
TcMYB50	AtMYB107	44.12	238	130	3	1	705	1	225	3.00E-60	191
TcMYB50	AtMYB9	72.41	116	32	0	1	348	1	116	5.00E-60	191
TcMYB50	AtMYB41	59.72	144	58	0	1	432	1	144	1.00E-58	186
TcMYB50	AtMYB74	42.13	254	143	4	1	750	1	253	4.00E-58	186
TcMYB50	AtMYB93	37.9	314	184	8	1	909	1	290	2.00E-57	186
TcMYB50	ATMYB61	55.9	161	68	1	1	474	1	161	2.00E-57	186
TcMYB50	ATMYB111	55.76	165	68	2	1	480	1	164	3.00E-57	184
TcMYB50	AtMYB42	57.14	154	60	1	1	444	1	154	1.00E-56	181
TcMYB50	ATMYB102	37.42	310	178	4	1	882	1	306	1.00E-56	183
TcMYB50	ATMYB15	49.44	180	81	2	1	510	1	180	6.00E-56	179
TcMYB50	AtMYB43	44.64	224	117	3	1	651	1	214	7.00E-56	180
TcMYB50	ATMYB12	69.83	116	35	0	1	348	1	116	2.00E-55	180
TcMYB50	ATMYB13	49.47	188	81	3	1	522	1	181	4.00E-55	176
TcMYB50	ATMYB58	58.33	144	59	1	4	432	4	147	5.00E-55	176
TcMYB50	AtMYB17	65.52	116	40	0	1	348	1	116	5.00E-55	177
TcMYB50	AtMYB50	56.58	152	66	1	1	456	1	148	5.00E-55	177
TcMYB50	ATMYB34	45.16	217	117	4	1	645	1	201	6.00E-55	177
TcMYB50	AtMYB49	36.42	324	171	8	1	867	1	305	8.00E-55	177
TcMYB50	ATMYB16	66.95	118	39	0	1	354	1	118	1.00E-54	177
TcMYB50	ATMYB86	67.8	118	38	0	1	354	1	118	1.00E-54	177
TcMYB50	ATMYB63	62.99	127	47	0	4	384	4	130	2.00E-54	176
TcMYB50	AtMYB85	67.8	118	38	0	1	354	1	118	2.00E-54	174
TcMYB50	ATMYB106	59.85	137	55	0	10	420	47	183	2.00E-54	178
TcMYB50	ATMYB30	55.48	146	65	0	1	438	1	146	5.00E-54	175
TcMYB50	ATMYB5	65.22	115	40	0	10	354	15	129	7.00E-54	172
TcMYB50	AtMYB60	57.14	140	60	0	1	420	1	140	2.00E-53	172
TcMYB50	ATMYB80	64.1	117	42	0	1	351	1	117	3.00E-53	173
TcMYB50	ATMYB72	47.51	181	95	1	4	546	4	183	3.00E-53	172
TcMYB50	AtMYB103	62.71	118	44	0	1	354	1	118	3.00E-53	174
TcMYB50	ATMYB14	63.56	118	43	0	1	354	1	118	4.00E-53	171
TcMYB50	ATMYB29	49.16	179	73	2	1	483	1	179	7.00E-53	172
TcMYB50	ATMYB67	52.44	164	72	3	16	489	16	175	1.00E-52	171
TcMYB50	ATMYB94	52.83	159	75	2	1	477	1	155	1.00E-52	172
TcMYB50	ATMYB11	66.38	116	39	0	1	348	1	116	1.00E-52	172
TcMYB50	AtMYB20	64.41	118	42	0	1	354	1	118	1.00E-52	170
TcMYB50	ATMYB96	57.53	146	54	1	1	414	1	146	2.00E-52	172
TcMYB50	AtMYB53	65.52	116	40	0	1	348	1	116	3.00E-52	170
TcMYB50	AtMYB10	53.85	143	65	1	7	432	5	147	6.00E-52	167
TcMYB50	ATMYB122	63.56	118	43	0	1	354	1	118	1.00E-51	169
TcMYB50	ATMYB31	54.79	146	66	1	1	438	1	144	2.00E-51	169
TcMYB50	ATMYB35	52.78	144	68	0	1	432	1	144	2.00E-51	168
TcMYB50	ATMYB92	51.03	145	71	0	1	435	1	145	2.00E-51	169
TcMYB50	AtMYB51	65.55	119	40	1	1	354	1	119	2.00E-51	169
TcMYB50	ATMYB37	36.33	278	154	4	1	765	1	277	5.00E-51	167
TcMYB50	AtMYB36	41.13	231	121	6	1	648	1	225	8.00E-51	167
TcMYB50	ATMYB99	55.86	145	55	2	4	411	3	147	1.00E-50	164
TcMYB50	ATMYB46	48.35	182	94	2	40	585	20	178	2.00E-50	164
TcMYB50	ATMYB55	60.94	128	38	1	1	348	1	128	6.00E-50	165
TcMYB50	ATMYB28	62.93	116	43	0	1	348	1	116	7.00E-50	166
TcMYB50	ATMYB68	47.67	172	85	3	1	501	1	172	8.00E-50	166
TcMYB50	ATMYB84	52.82	142	64	2	1	417	1	142	1.00E-49	163
TcMYB50	ATMYB123	67.89	109	35	0	37	363	15	123	2.00E-49	161
TcMYB50	AtMYB40	62.07	116	44	0	1	348	1	116	5.00E-49	160
TcMYB50	ATMYB95	49.08	163	83	1	1	489	1	161	7.00E-49	160
TcMYB50	ATMYB26	60	125	41	1	1	348	1	125	7.00E-49	163
TcMYB50	AtMYB76	47.8	182	91	4	1	534	1	173	2.00E-48	161
TcMYB50	AtMYB83	58.33	120	50	0	25	384	27	146	3.00E-48	160
TcMYB50	ATMYB87	50.34	145	71	1	1	432	1	145	3.00E-48	159
TcMYB50	AtMYB19	46.45	183	93	3	25	558	9	187	1.00E-46	154
TcMYB50	ATMYB66	63.11	103	38	0	40	348	18	120	1.00E-46	152
TcMYB50	ATMYB23	56.52	115	50	0	40	384	14	128	2.00E-46	152
TcMYB50	ATMYB38	44.58	166	76	2	1	450	1	166	8.00E-46	153

TcMYB50	AtMYB47	44.97	169	91	1	1	501	1	169	1.00E-44	149
TcMYB50	ATMYB0	46.58	146	73	1	22	444	10	155	3.00E-44	147
TcMYB50	ATMYB90	47.5	160	82	2	40	513	10	166	1.00E-43	145
TcMYB50	AtMYB114	61.76	102	39	0	40	345	10	111	2.00E-43	141
TcMYB50	AtMYB82	57.94	107	45	0	25	345	9	115	3.00E-43	143
TcMYB50	AtMYB79	56.2	121	53	1	40	402	8	126	4.00E-43	145
TcMYB50	ATMYB3	45.52	145	79	0	4	438	10	154	4.00E-42	141
TcMYB50	AtMYB18	42.93	184	86	2	40	534	12	195	5.00E-42	142
TcMYB50	AtMYB112	46.79	156	77	3	40	489	34	189	9.00E-42	140
TcMYB50	AtMYB24	45.86	157	83	2	40	504	19	175	1.00E-41	139
TcMYB50	ATMYB57	59.63	109	44	0	22	348	21	129	1.00E-41	139
TcMYB50	ATMYB71	55.37	121	54	2	40	402	20	138	1.00E-41	141
TcMYB50	AtMYB45	47.55	143	69	2	25	435	15	157	1.00E-41	140
TcMYB50	ATMYB75	60.78	102	40	0	40	345	10	111	2.00E-41	140
TcMYB50	AtMYB113	38.5	200	114	3	1	573	1	186	4.00E-41	139
TcMYB50	ATMYB101	31.08	325	190	8	40	912	20	335	1.00E-40	143
TcMYB50	ATMYB121	48.55	138	68	1	40	444	29	166	3.00E-40	137
TcMYB50	AtMYB81	55.66	106	47	0	25	342	17	122	7.00E-40	140
TcMYB50	AtMYB97	59.41	101	41	0	40	342	21	121	2.00E-39	138
TcMYB50	ATMYB120	59.22	103	42	0	40	348	28	130	4.00E-39	140
TcMYB50	ATMYB33	56.86	102	44	0	40	345	34	135	5.00E-39	139
TcMYB50	AtMYB108	52.94	119	56	0	40	396	21	139	9.00E-39	135
TcMYB50	ATMYB2	48.8	125	64	0	22	396	16	140	1.00E-38	133
TcMYB50	ATMYB65	56.86	102	44	0	40	345	43	144	2.00E-38	138
TcMYB50	AtMYB62	38.92	203	109	4	22	585	15	212	4.00E-38	132
TcMYB50	AtMYB116	50.85	118	58	0	22	375	14	131	2.00E-37	130
TcMYB50	AtMYB27	59.8	102	41	0	40	345	11	112	3.00E-37	128
TcMYB50	ATMYB59-3	42.28	149	81	1	40	471	10	158	8.00E-37	127
TcMYB50	ATMYB78	33.62	232	112	4	22	591	22	252	6.00E-36	127
TcMYB50	ATMYB48	46.51	129	64	1	40	411	9	137	1.00E-35	124
TcMYB50	AtMYB115	41.01	139	81	3	22	435	152	288	9.00E-30	111
TcMYB50	ATMYB119	45.22	115	62	2	40	381	105	217	4.00E-29	110
TcMYB50	AtMYB104	47.57	103	54	1	22	330	13	114	8.00E-29	108
TcMYB50	AtMYB1	28.81	302	201	9	40	903	55	334	5.00E-28	107
TcMYB50	ATMYB77	37.34	158	90	3	40	486	6	162	1.00E-27	104
TcMYB50	AtMYB64	39.6	149	76	5	40	444	105	249	1.00E-27	106
TcMYB50	ATMYB73	50	102	51	1	40	345	13	113	1.00E-27	104
TcMYB50	ATMYB25	49.52	105	53	1	40	354	50	153	2.00E-27	105
TcMYB50	ATMYB118	39.87	153	90	4	22	474	183	331	3.00E-27	105
TcMYB50	AtMYB98	39.71	136	82	2	22	429	211	343	6.00E-27	104
TcMYB50	AtMYB70	49.02	102	52	1	40	345	13	113	8.00E-27	102
TcMYB50	AtMYB109	43.59	156	87	5	40	504	56	198	1.00E-26	103
TcMYB50	ATMYB54	38.17	131	80	3	37	426	5	125	3.00E-25	96.3
TcMYB50	AtMYB117	38.26	149	88	4	40	474	98	240	3.00E-25	98.6
TcMYB50	ATMYB44	47.06	102	54	1	40	345	6	106	6.00E-25	96.7
TcMYB50	ATMYB105	37.76	143	88	4	25	450	102	233	2.00E-24	95.5
TcMYB50	AtMYB100	35.25	139	90	2	22	438	20	155	4.00E-23	90.1
TcMYB50	ATMYB52	43.88	98	54	2	37	327	4	99	9.00E-23	89.4
TcMYB50	ATMYB69	29.45	163	115	2	7	495	13	169	1.00E-21	86.3
TcMYB50	ATMYB110	36.36	110	70	1	37	366	66	174	4.00E-21	85.9
TcMYB50	ATMYB91	34.29	140	85	2	49	447	7	146	5.00E-21	86.3
TcMYB50	AtMYB22	31.21	141	97	2	22	444	48	184	8.00E-21	84
TcMYB50	AtMYB89	37.04	108	68	1	43	366	57	163	5.00E-20	80.1
TcMYB50	AtMYB56	37.04	108	68	1	37	360	92	198	1.00E-19	82
TcMYB50	AtMYB124	33.08	133	89	1	49	447	28	159	1.00E-18	79.7
TcMYB50	ATMYB88	40.4	99	59	1	49	345	33	130	8.00E-18	77.4
TcMYB51	ATMYB3	58.33	168	66	2	1	492	1	167	7.00E-64	197
TcMYB51	AtMYB6	52.91	189	87	3	1	561	1	178	4.00E-63	194
TcMYB51	ATMYB12	72.65	117	32	1	1	351	1	116	1.00E-60	192
TcMYB51	ATMYB4	63.57	140	51	1	1	420	1	139	1.00E-60	189
TcMYB51	ATMYB111	51.04	192	74	3	1	516	1	190	1.00E-59	189
TcMYB51	AtMYB32	60.42	144	57	1	1	432	1	143	1.00E-59	186
TcMYB51	MYB7	59.46	148	60	1	1	444	1	147	2.00E-59	186
TcMYB51	MYB8	49.49	196	97	4	1	582	1	182	4.00E-59	183
TcMYB51	ATMYB5	68.6	121	38	1	13	375	16	135	5.00E-58	181
TcMYB51	ATMYB123	58.97	156	64	2	7	474	4	148	7.00E-58	181
TcMYB51	AtMYB74	69.23	117	36	0	1	351	1	117	8.00E-58	183
TcMYB51	ATMYB11	69.23	117	36	1	1	351	1	116	2.00E-57	183
TcMYB51	AtMYB41	38.57	293	161	7	1	822	1	280	4.00E-57	180
TcMYB51	ATMYB34	61.03	136	53	1	1	408	1	135	4.00E-57	181
TcMYB51	ATMYB13	46	200	103	4	1	585	1	195	8.00E-57	178
TcMYB51	ATMYB15	60.14	138	55	1	1	414	1	137	3.00E-56	178
TcMYB51	AtMYB9	37.96	274	153	6	1	771	1	269	7.00E-56	179
TcMYB51	ATMYB14	60.77	130	51	1	1	390	1	129	1.00E-55	175
TcMYB51	AtMYB51	68.38	117	37	0	1	351	1	117	1.00E-55	178
TcMYB51	ATMYB102	39.32	295	152	8	1	804	1	284	3.00E-55	177
TcMYB51	AtMYB107	67.52	117	38	1	1	351	1	116	5.00E-55	176
TcMYB51	ATMYB16	70.09	117	35	1	1	351	1	116	4.00E-54	174
TcMYB51	AtMYB82	53.25	154	60	2	19	444	3	156	5.00E-54	169
TcMYB51	ATMYB29	68.38	117	37	1	1	351	1	116	4.00E-53	171

TcMYB51	ATMYB28	65.29	121	42	1	1	363	1	120	1.00E-52	171
TcMYB51	ATMYB106	63.85	130	41	2	10	381	47	175	2.00E-52	171
TcMYB51	ATMYB86	38.95	285	158	7	1	807	1	277	2.00E-52	170
TcMYB51	ATMYB80	61.98	121	46	1	1	363	1	120	3.00E-52	169
TcMYB51	AtMYB17	65.81	117	40	1	1	351	1	116	3.00E-52	168
TcMYB51	AtMYB114	69.16	107	33	0	34	354	7	113	4.00E-52	162
TcMYB51	ATMYB92	65.81	117	40	1	1	351	1	116	4.00E-52	169
TcMYB51	ATMYB122	64.46	121	43	1	1	363	1	120	6.00E-52	168
TcMYB51	AtMYB60	43.44	221	122	6	1	654	1	202	8.00E-52	166
TcMYB51	ATMYB66	60.16	123	46	1	22	381	8	130	1.00E-51	164
TcMYB51	AtMYB85	64.96	117	41	1	1	351	1	116	2.00E-51	165
TcMYB51	AtMYB93	64.96	117	41	1	1	351	1	116	5.00E-51	167
TcMYB51	AtMYB42	49.44	180	88	3	1	531	1	170	5.00E-51	164
TcMYB51	ATMYB23	61.21	116	45	1	25	372	8	122	9.00E-51	162
TcMYB51	ATMYB61	63.64	121	44	1	1	363	1	120	1.00E-50	166
TcMYB51	ATMYB63	51.95	154	73	2	4	462	4	156	1.00E-50	164
TcMYB51	ATMYB72	38.74	253	148	5	4	741	4	238	1.00E-50	164
TcMYB51	AtMYB76	64.1	117	42	1	1	351	1	116	2.00E-50	164
TcMYB51	ATMYB90	68.81	109	34	0	34	360	7	115	2.00E-50	162
TcMYB51	AtMYB20	40.71	226	130	4	1	666	1	224	3.00E-50	162
TcMYB51	ATMYB75	67.29	107	35	0	34	354	7	113	3.00E-50	161
TcMYB51	ATMYB35	61.54	117	45	1	1	351	1	116	3.00E-50	163
TcMYB51	AtMYB36	63.56	118	42	2	1	351	1	117	3.00E-50	164
TcMYB51	ATMYB94	53.33	150	68	2	1	444	1	149	5.00E-50	163
TcMYB51	ATMYB96	51.97	152	61	2	1	420	1	151	5.00E-50	164
TcMYB51	AtMYB49	68.18	110	35	0	22	351	7	116	7.00E-50	162
TcMYB51	ATMYB99	44.79	192	93	5	4	540	3	190	1.00E-49	160
TcMYB51	ATMYB84	64.41	118	41	2	1	351	1	117	1.00E-49	162
TcMYB51	ATMYB71	55.56	135	58	1	28	426	15	149	2.00E-49	160
TcMYB51	AtMYB103	60.16	123	49	1	1	369	1	122	2.00E-49	162
TcMYB51	AtMYB113	62.07	116	44	1	1	348	1	111	3.00E-49	159
TcMYB51	ATMYB58	50	158	69	2	4	447	4	160	3.00E-49	159
TcMYB51	AtMYB50	53.06	147	69	1	1	441	1	146	3.00E-49	160
TcMYB51	AtMYB53	64.1	117	42	1	1	351	1	116	4.00E-49	160
TcMYB51	ATMYB0	61.95	113	43	0	43	381	16	128	5.00E-49	157
TcMYB51	AtMYB43	61.98	121	46	1	1	363	1	120	6.00E-49	160
TcMYB51	ATMYB87	53.74	147	62	4	1	423	1	146	7.00E-49	159
TcMYB51	ATMYB37	59.38	128	51	2	1	381	1	127	9.00E-49	160
TcMYB51	ATMYB68	62.71	118	43	2	1	351	1	117	1.00E-48	160
TcMYB51	ATMYB30	52.35	149	69	2	1	441	1	148	2.00E-48	159
TcMYB51	ATMYB31	62.39	117	44	1	1	351	1	116	5.00E-48	158
TcMYB51	AtMYB79	56.92	130	54	1	43	426	8	137	7.00E-48	155
TcMYB51	ATMYB3	54.74	137	54	1	22	408	15	151	7.00E-48	154
TcMYB51	ATMYB67	46.39	166	89	3	16	513	16	174	1.00E-47	156
TcMYB51	AtMYB24	53.52	142	65	2	22	444	12	149	1.00E-47	153
TcMYB51	ATMYB55	58.65	133	43	2	1	363	1	132	2.00E-47	157
TcMYB51	ATMYB95	61.98	121	46	1	1	363	1	120	2.00E-47	154
TcMYB51	AtMYB83	33.33	324	198	6	37	954	30	323	3.00E-47	156
TcMYB51	ATMYB38	61.02	118	45	2	1	351	1	117	5.00E-47	154
TcMYB51	ATMYB46	55.65	124	55	1	37	408	18	137	9.00E-47	153
TcMYB51	AtMYB40	59.5	121	49	1	1	363	1	120	1.00E-46	152
TcMYB51	AtMYB10	54.29	140	60	3	7	414	5	143	2.00E-46	151
TcMYB51	ATMYB121	55.73	131	58	2	34	426	26	149	4.00E-46	151
TcMYB51	AtMYB47	43.82	178	100	1	1	534	1	177	7.00E-46	150
TcMYB51	ATMYB57	55.65	115	51	0	25	369	21	135	1.00E-45	148
TcMYB51	AtMYB112	50.34	147	61	2	37	441	32	178	1.00E-45	149
TcMYB51	ATMYB26	52.52	139	57	2	1	390	1	138	3.00E-45	152
TcMYB51	ATMYB2	55.75	113	50	0	10	348	11	123	6.00E-45	148
TcMYB51	AtMYB62	61.47	109	42	0	22	348	14	122	3.00E-44	147
TcMYB51	ATMYB101	58.1	105	44	0	34	348	17	121	9.00E-44	150
TcMYB51	AtMYB108	64.08	103	37	0	37	345	19	121	1.00E-42	144
TcMYB51	AtMYB116	60.78	102	40	0	43	348	20	121	2.00E-42	142
TcMYB51	AtMYB97	58.42	101	42	0	37	339	19	119	6.00E-41	140
TcMYB51	ATMYB120	58.1	105	44	0	37	351	26	130	1.00E-40	142
TcMYB51	ATMYB48	47.66	128	67	0	25	408	3	130	1.00E-40	136
TcMYB51	AtMYB81	57.01	107	46	1	43	363	22	127	3.00E-40	139
TcMYB51	ATMYB33	45.32	139	70	1	37	435	32	170	7.00E-40	140
TcMYB51	AtMYB27	58.18	110	46	0	25	354	5	114	9.00E-40	134
TcMYB51	ATMYB59-3	54.81	104	47	0	43	354	10	113	2.00E-39	133
TcMYB51	AtMYB18	52.42	124	59	1	43	414	12	134	5.00E-39	133
TcMYB51	ATMYB78	54.55	121	41	1	25	345	22	142	1.00E-38	133
TcMYB51	ATMYB65	50.93	108	53	0	25	348	37	144	2.00E-38	137
TcMYB51	AtMYB19	58.82	102	42	0	43	348	14	115	3.00E-38	130
TcMYB51	AtMYB45	52.48	101	48	0	43	345	20	120	1.00E-35	123
TcMYB51	AtMYB104	44.9	98	54	0	43	336	18	115	1.00E-30	112
TcMYB51	ATMYB118	34.22	187	115	5	22	558	182	361	4.00E-29	109
TcMYB51	AtMYB117	44.04	109	61	1	22	348	91	198	4.00E-28	105
TcMYB51	ATMYB73	46.73	107	57	1	43	363	13	118	6.00E-28	104
TcMYB51	ATMYB73	34.85	66	42	1	202	396	13	78	8.00E-06	39.3
TcMYB51	ATMYB105	40.87	115	68	1	22	366	100	213	1.00E-27	103

TcMYB51	AtMYB98	47.57	103	54	1	22	330	210	311	8.00E-27	102
TcMYB51	AtMYB98	28.36	67	47	1	142	339	197	263	6.00E-04	33.5
TcMYB51	AtMYB115	32.78	180	121	5	37	576	156	320	2.00E-26	100
TcMYB51	AtMYB70	43.75	112	63	1	43	378	13	123	3.00E-26	99.4
TcMYB51	AtMYB70	35.94	64	40	1	202	390	13	76	8.00E-06	39.3
TcMYB51	ATMYB119	46.94	98	52	1	37	330	103	199	5.00E-26	100
TcMYB51	ATMYB119	27.42	62	44	1	202	384	105	166	4.00E-04	34.3
TcMYB51	AtMYB64	40.31	129	73	2	43	417	105	232	9.00E-26	99.8
TcMYB51	ATMYB77	39.1	133	81	3	43	441	6	133	2.00E-25	97.1
TcMYB51	ATMYB52	43.69	103	58	1	40	348	4	105	2.00E-25	95.9
TcMYB51	AtMYB100	41.75	103	60	1	25	333	20	121	4.00E-25	94.7
TcMYB51	ATMYB54	39.52	124	75	2	40	411	5	114	5.00E-25	94.7
TcMYB51	AtMYB1	46.23	106	53	2	43	348	55	155	6.00E-25	97.1
TcMYB51	AtMYB1	37.5	56	34	1	187	351	50	105	9.00E-05	36.2
TcMYB51	ATMYB25	46.08	102	55	1	43	348	50	150	9.00E-25	96.3
TcMYB51	ATMYB110	38.94	113	68	2	40	375	66	177	1.00E-24	94.7
TcMYB51	AtMYB109	40.32	124	74	1	43	414	56	178	2.00E-24	95.9
TcMYB51	ATMYB44	43.14	102	58	1	43	348	6	106	1.00E-23	92
TcMYB51	ATMYB44	33.33	66	43	1	202	396	6	71	3.00E-05	37.4
TcMYB51	AtMYB56	32.62	141	90	2	40	447	92	231	4.00E-23	90.9
TcMYB51	AtMYB124	39.13	115	70	1	52	396	28	141	2.00E-22	90.1
TcMYB51	ATMYB69	38.21	123	75	2	22	387	12	133	3.00E-22	87
TcMYB51	AtMYB89	40.62	96	57	1	46	333	57	151	2.00E-21	83.6
TcMYB51	ATMYB91	32.45	151	96	2	43	477	4	154	2.00E-21	86.7
TcMYB51	AtMYB22	32.23	121	82	1	25	387	48	166	3.00E-20	81.6
TcMYB51	ATMYB88	40.54	111	66	2	52	384	33	141	8.00E-20	82.8
TcMYB52	AtMYB83	71.13	142	41	1	22	447	27	164	2.00E-70	218
TcMYB52	ATMYB46	72.39	134	35	1	1	396	8	141	4.00E-68	209
TcMYB52	ATMYB86	40.65	310	156	8	22	867	9	290	1.00E-65	206
TcMYB52	AtMYB50	81.48	108	20	0	22	345	9	116	9.00E-63	197
TcMYB52	ATMYB61	79.82	109	22	0	22	348	9	117	1.00E-60	193
TcMYB52	ATMYB55	73.55	121	20	1	22	348	9	129	6.00E-59	188
TcMYB52	AtMYB103	33.92	342	170	8	28	885	11	350	3.00E-58	187
TcMYB52	ATMYB67	57.33	150	56	1	22	447	19	168	9.00E-57	181
TcMYB52	ATMYB26	69.67	122	28	1	22	360	9	130	6.00E-56	181
TcMYB52	AtMYB32	50.6	166	82	2	22	519	9	171	7.00E-53	170
TcMYB52	AtMYB9	34.24	295	175	5	31	858	12	302	1.00E-52	171
TcMYB52	AtMYB20	38.99	277	167	6	22	846	9	262	5.00E-52	168
TcMYB52	ATMYB106	37.83	267	156	5	22	792	52	292	5.00E-51	168
TcMYB52	ATMYB3	65.74	108	37	0	22	345	9	116	5.00E-51	164
TcMYB52	MYB7	65.74	108	37	0	22	345	9	116	1.00E-50	164
TcMYB52	ATMYB92	68.57	105	33	0	31	345	12	116	1.00E-50	166
TcMYB52	AtMYB53	69.52	105	32	0	31	345	12	116	1.00E-50	165
TcMYB52	AtMYB6	65.74	108	37	0	22	345	9	116	1.00E-50	163
TcMYB52	AtMYB107	69.52	105	32	0	31	345	12	116	2.00E-50	165
TcMYB52	AtMYB42	69.44	108	33	0	22	345	9	116	3.00E-50	163
TcMYB52	AtMYB74	68.18	110	35	0	16	345	8	117	9.00E-50	163
TcMYB52	ATMYB102	69.44	108	33	0	22	345	9	116	1.00E-49	164
TcMYB52	AtMYB93	34.4	375	226	14	31	1095	12	357	2.00E-49	163
TcMYB52	ATMYB4	64.81	108	38	0	22	345	9	116	3.00E-49	160
TcMYB52	ATMYB13	57.66	137	54	1	31	429	12	148	5.00E-49	159
TcMYB52	MYB8	56.25	128	50	1	22	387	9	136	9.00E-49	157
TcMYB52	AtMYB45	54.74	137	53	1	16	399	13	149	1.00E-48	158
TcMYB52	AtMYB85	68.57	105	33	0	31	345	12	116	2.00E-48	158
TcMYB52	AtMYB49	36.91	298	170	8	10	849	5	286	2.00E-48	160
TcMYB52	AtMYB43	66.97	109	36	0	22	348	9	117	4.00E-48	159
TcMYB52	ATMYB14	63.16	114	42	0	22	363	9	122	7.00E-48	156
TcMYB52	ATMYB95	46.91	162	75	1	31	483	12	173	1.00E-47	156
TcMYB52	AtMYB17	65.71	105	36	0	31	345	12	116	1.00E-47	157
TcMYB52	AtMYB19	46.49	185	96	3	22	567	9	183	2.00E-47	155
TcMYB52	AtMYB10	43.48	184	97	3	22	552	11	192	3.00E-47	154
TcMYB52	ATMYB12	54.23	142	60	2	22	432	9	149	8.00E-47	157
TcMYB52	AtMYB40	65.42	107	37	0	31	351	12	118	8.00E-47	154
TcMYB52	AtMYB41	64.81	108	38	0	22	345	9	116	1.00E-46	154
TcMYB52	ATMYB58	54.61	141	58	1	22	426	11	151	1.00E-46	154
TcMYB52	ATMYB80	62.04	108	41	0	22	345	9	116	1.00E-46	155
TcMYB52	AtMYB82	51.09	137	67	1	16	426	7	140	2.00E-46	151
TcMYB52	ATMYB23	51.08	139	67	1	1	414	1	139	2.00E-46	151
TcMYB52	ATMYB63	54.69	128	58	0	22	405	11	138	2.00E-46	154
TcMYB52	AtMYB18	47.06	170	89	2	34	540	11	177	4.00E-46	152
TcMYB52	ATMYB16	63.81	105	38	0	31	345	12	116	4.00E-46	154
TcMYB52	ATMYB71	61.4	114	44	0	34	375	19	132	5.00E-46	152
TcMYB52	AtMYB51	53.73	134	60	1	16	411	8	141	5.00E-46	154
TcMYB52	ATMYB72	59.17	120	49	0	22	381	11	130	5.00E-46	152
TcMYB52	ATMYB66	53.6	125	51	1	16	369	11	135	6.00E-46	149
TcMYB52	ATMYB34	61.32	106	41	0	31	348	12	117	7.00E-46	152
TcMYB52	ATMYB57	47.22	144	76	0	16	447	20	163	1.00E-45	149
TcMYB52	ATMYB99	62.5	112	35	1	31	345	13	124	2.00E-45	150
TcMYB52	AtMYB79	61.06	113	44	0	34	372	7	119	2.00E-45	150
TcMYB52	AtMYB47	39.9	203	98	3	31	567	12	214	2.00E-45	150

TcMYB52	ATMYB28	42.7	185	104	2	31	579	12	195	2.00E-45	153
TcMYB52	ATMYB3	51.91	131	63	0	13	405	14	144	3.00E-45	148
TcMYB52	ATMYB15	60.55	109	43	0	22	348	9	117	4.00E-45	150
TcMYB52	ATMYB0	50.4	125	62	0	34	408	15	139	5.00E-45	148
TcMYB52	ATMYB123	53.79	132	58	1	10	396	7	138	1.00E-44	148
TcMYB52	ATMYB11	61.11	108	42	0	22	345	9	116	1.00E-44	150
TcMYB52	ATMYB5	58.33	108	45	0	22	345	20	127	1.00E-44	147
TcMYB52	ATMYB30	38.14	236	129	6	22	678	9	241	2.00E-44	149
TcMYB52	AtMYB76	61.9	105	40	0	31	345	12	116	2.00E-44	150
TcMYB52	ATMYB29	62.86	105	39	0	31	345	12	116	2.00E-44	149
TcMYB52	ATMYB94	57.66	111	47	0	31	363	12	122	3.00E-44	149
TcMYB52	ATMYB111	61.9	105	40	0	31	345	12	116	3.00E-44	149
TcMYB52	AtMYB60	52	125	60	0	31	405	12	136	6.00E-44	147
TcMYB52	ATMYB35	60.19	108	43	0	22	345	9	116	1.00E-43	147
TcMYB52	ATMYB96	57.66	111	47	0	31	363	12	122	2.00E-43	147
TcMYB52	AtMYB24	54.76	126	57	1	28	405	16	134	2.00E-43	143
TcMYB52	AtMYB62	58.18	110	46	0	28	357	18	127	4.00E-43	145
TcMYB52	ATMYB122	62.86	105	39	0	31	345	12	116	4.00E-43	146
TcMYB52	ATMYB33	63.46	104	38	0	31	342	32	135	8.00E-43	149
TcMYB52	ATMYB48	38.16	228	127	5	34	675	8	223	2.00E-42	142
TcMYB52	ATMYB31	51.91	131	60	1	22	405	9	139	3.00E-42	144
TcMYB52	AtMYB108	34.17	278	156	6	16	768	14	281	3.00E-42	143
TcMYB52	ATMYB121	53.08	130	59	1	34	417	28	157	4.00E-42	142
TcMYB52	ATMYB37	51.15	131	63	2	22	411	9	132	7.00E-42	142
TcMYB52	ATMYB101	34.28	283	185	9	31	876	18	249	1.00E-41	145
TcMYB52	AtMYB116	56.48	108	47	0	34	357	19	126	3.00E-41	140
TcMYB52	ATMYB84	50.77	130	63	1	22	408	9	138	3.00E-41	140
TcMYB52	AtMYB114	57.69	104	44	0	31	342	8	111	5.00E-41	134
TcMYB52	AtMYB112	55.14	107	48	0	28	348	31	137	7.00E-41	137
TcMYB52	AtMYB81	57.55	106	45	0	22	339	17	122	7.00E-41	142
TcMYB52	ATMYB2	52.59	116	55	0	4	351	11	126	8.00E-41	138
TcMYB52	ATMYB65	59.62	104	42	0	31	342	41	144	8.00E-41	144
TcMYB52	AtMYB97	58.25	103	43	0	31	339	19	121	1.00E-40	140
TcMYB52	ATMYB120	58.49	106	44	0	31	348	26	131	1.00E-40	143
TcMYB52	ATMYB59-3	48.12	133	69	1	34	432	9	139	2.00E-40	136
TcMYB52	ATMYB38	39.09	197	107	6	22	573	9	192	3.00E-40	137
TcMYB52	AtMYB36	57.8	109	45	1	22	345	9	117	3.00E-40	138
TcMYB52	ATMYB68	52.5	120	56	1	22	378	9	128	7.00E-40	138
TcMYB52	ATMYB90	38.69	199	108	5	31	585	8	203	6.00E-39	132
TcMYB52	ATMYB75	56.73	104	45	0	31	342	8	111	9.00E-39	132
TcMYB52	ATMYB87	31.68	262	173	5	22	789	9	250	2.00E-38	133
TcMYB52	AtMYB27	53.21	109	51	0	31	357	9	117	7.00E-38	129
TcMYB52	AtMYB113	48.48	132	61	1	16	390	3	134	1.00E-37	129
TcMYB52	ATMYB78	48	125	51	1	16	348	21	145	5.00E-35	124
TcMYB52	AtMYB104	50	102	51	0	22	327	13	114	7.00E-33	119
TcMYB52	ATMYB73	48.25	114	59	1	1	342	1	113	3.00E-29	108
TcMYB52	AtMYB115	34.87	152	86	3	22	438	153	303	2.00E-28	107
TcMYB52	AtMYB70	47.37	114	60	1	1	342	1	113	2.00E-28	105
TcMYB52	ATMYB118	36.3	146	92	2	7	441	179	322	3.00E-28	107
TcMYB52	AtMYB98	38.51	148	82	3	22	438	212	358	1.00E-27	105
TcMYB52	AtMYB1	50.98	102	50	1	37	342	55	155	1.00E-27	105
TcMYB52	ATMYB105	37.41	139	86	2	4	417	96	232	2.00E-27	103
TcMYB52	AtMYB64	38.66	119	73	1	37	393	105	222	3.00E-27	104
TcMYB52	ATMYB69	38.13	139	86	2	22	438	14	148	3.00E-27	101
TcMYB52	ATMYB119	36.69	139	85	2	4	411	94	231	4.00E-27	104
TcMYB52	AtMYB109	46.72	122	62	3	37	393	56	175	4.00E-27	103
TcMYB52	ATMYB44	47.06	102	54	1	37	342	6	106	7.00E-27	101
TcMYB52	ATMYB54	38.64	132	80	2	37	429	6	135	8.00E-27	100
TcMYB52	ATMYB77	47.06	102	54	1	37	342	6	106	2.00E-26	100
TcMYB52	ATMYB25	49.51	103	51	2	37	342	50	150	2.00E-26	101
TcMYB52	AtMYB100	39.66	116	70	2	22	369	21	134	3.00E-25	95.5
TcMYB52	AtMYB117	40	120	70	3	10	363	88	205	3.00E-24	95.1
TcMYB52	ATMYB52	43.14	102	58	1	37	342	5	105	4.00E-24	92.8
TcMYB52	ATMYB110	34.71	121	79	1	37	399	67	186	7.00E-23	90.5
TcMYB52	ATMYB88	33.12	157	97	4	46	492	33	188	2.00E-22	90.9
TcMYB52	AtMYB56	37.74	106	66	1	37	354	93	197	5.00E-21	85.5
TcMYB52	AtMYB124	25.98	254	182	6	46	789	28	277	9.00E-21	85.9
TcMYB52	AtMYB89	32.35	136	92	1	16	423	49	183	3.00E-20	80.5
TcMYB52	AtMYB22	35.34	116	75	3	22	369	49	161	1.00E-19	80.5
TcMYB52	ATMYB91	37.04	108	66	1	46	363	7	114	1.00E-18	79
TcMYB53	ATMYB12	58.62	145	47	3	1	396	1	144	1.00E-55	178
TcMYB53	ATMYB5	64.8	125	44	1	13	387	16	139	1.00E-55	174
TcMYB53	ATMYB15	36.68	289	166	8	1	816	1	275	3.00E-55	174
TcMYB53	ATMYB123	70.18	114	34	0	7	348	4	117	5.00E-55	173
TcMYB53	AtMYB74	42.13	197	105	2	1	564	1	197	8.00E-55	174
TcMYB53	ATMYB23	45.5	200	91	4	37	582	12	210	9.00E-55	171
TcMYB53	ATMYB111	66.38	116	39	1	1	348	1	115	1.00E-54	174
TcMYB53	ATMYB3	52.9	155	73	3	1	465	1	141	3.00E-54	171
TcMYB53	AtMYB6	48.33	180	78	4	1	495	1	179	6.00E-54	169
TcMYB53	ATMYB66	44.21	190	106	4	37	606	16	189	9.00E-54	168

TcMYB53	ATMYB11	57.66	137	53	2	1	396	1	136	1.00E-53	172
TcMYB53	MYB8	61.02	118	46	1	1	354	1	117	1.00E-52	165
TcMYB53	ATMYB4	57.36	129	55	1	1	387	1	128	2.00E-52	167
TcMYB53	ATMYB102	37.7	244	143	5	1	705	1	238	3.00E-52	168
TcMYB53	AtMYB32	57.36	129	55	1	1	387	1	128	4.00E-52	166
TcMYB53	AtMYB114	64.35	115	41	0	34	378	7	121	6.00E-52	161
TcMYB53	MYB7	61.86	118	45	1	1	354	1	117	6.00E-52	165
TcMYB53	ATMYB13	62.07	116	44	1	1	348	1	115	3.00E-51	163
TcMYB53	ATMYB34	53.64	151	70	1	1	453	1	150	4.00E-51	164
TcMYB53	AtMYB41	41.18	204	120	2	1	612	1	200	5.00E-51	163
TcMYB53	AtMYB9	57.36	129	55	1	1	387	1	128	5.00E-51	165
TcMYB53	AtMYB107	56.59	129	56	1	1	387	1	128	7.00E-51	164
TcMYB53	ATMYB75	60.33	121	48	0	34	396	7	127	1.00E-50	161
TcMYB53	AtMYB82	62.28	114	43	0	40	381	13	126	1.00E-50	160
TcMYB53	ATMYB0	59.13	115	47	0	37	381	14	128	2.00E-50	160
TcMYB53	AtMYB85	43.59	195	106	4	1	573	1	191	4.00E-50	160
TcMYB53	AtMYB17	54.48	134	61	1	1	402	1	133	1.00E-49	160
TcMYB53	ATMYB90	63.25	117	43	1	34	384	7	120	2.00E-49	158
TcMYB53	ATMYB14	61.21	116	45	1	1	348	1	115	2.00E-49	158
TcMYB53	AtMYB42	50	164	81	3	1	489	1	160	2.00E-49	159
TcMYB53	ATMYB29	48.8	166	83	2	1	492	1	165	3.00E-49	160
TcMYB53	AtMYB51	57.03	128	55	0	1	384	1	128	5.00E-49	160
TcMYB53	ATMYB94	62.07	116	44	1	1	348	1	115	6.00E-49	159
TcMYB53	ATMYB28	47.2	161	85	1	1	483	1	160	6.00E-49	160
TcMYB53	AtMYB113	61.21	116	45	1	1	348	1	111	6.00E-49	157
TcMYB53	ATMYB16	44.15	188	105	2	1	564	1	184	7.00E-49	159
TcMYB53	AtMYB60	49.68	157	78	2	1	468	1	156	1.00E-48	157
TcMYB53	ATMYB31	50.99	151	69	2	1	438	1	150	1.00E-48	158
TcMYB53	ATMYB96	61.21	116	45	1	1	348	1	115	7.00E-48	157
TcMYB53	ATMYB121	48.72	156	68	1	34	465	26	181	1.00E-47	154
TcMYB53	ATMYB122	61.74	115	44	1	1	345	1	114	2.00E-47	155
TcMYB53	ATMYB30	49.67	153	71	2	1	441	1	152	7.00E-47	154
TcMYB53	AtMYB43	38.28	209	129	3	1	627	1	187	8.00E-47	154
TcMYB53	ATMYB63	38.5	213	122	4	4	615	4	214	1.00E-46	152
TcMYB53	ATMYB35	33.21	268	148	5	1	711	1	267	2.00E-46	152
TcMYB53	ATMYB92	56.59	129	56	1	1	387	1	128	2.00E-46	153
TcMYB53	ATMYB80	52.71	129	61	1	1	387	1	128	3.00E-46	152
TcMYB53	AtMYB20	53.49	129	60	1	1	387	1	128	3.00E-46	151
TcMYB53	AtMYB53	54.26	129	59	1	1	387	1	128	3.00E-46	152
TcMYB53	AtMYB79	59.48	116	47	0	37	384	6	121	3.00E-46	150
TcMYB53	ATMYB71	38.03	213	127	4	34	657	17	211	6.00E-46	150
TcMYB53	ATMYB72	56.1	123	54	1	4	372	4	125	6.00E-46	150
TcMYB53	ATMYB106	45.61	171	93	2	10	522	47	213	6.00E-46	153
TcMYB53	AtMYB93	53.49	129	60	1	1	387	1	128	7.00E-46	152
TcMYB53	AtMYB10	44.51	164	91	2	7	498	5	164	1.00E-45	148
TcMYB53	AtMYB36	47.44	156	81	3	1	465	1	152	1.00E-45	150
TcMYB53	ATMYB58	57.39	115	49	1	4	348	4	117	2.00E-45	148
TcMYB53	AtMYB50	47.1	155	82	1	1	465	1	154	7.00E-45	148
TcMYB53	ATMYB37	52.63	133	62	2	1	396	1	132	7.00E-45	149
TcMYB53	ATMYB86	59.48	116	47	1	1	348	1	115	2.00E-44	148
TcMYB53	AtMYB76	51.94	129	62	1	1	387	1	128	2.00E-44	148
TcMYB53	ATMYB87	51.2	125	60	2	1	372	1	124	3.00E-44	146
TcMYB53	ATMYB84	51.97	127	60	2	1	378	1	126	3.00E-44	146
TcMYB53	AtMYB49	61.26	111	43	0	22	354	7	117	4.00E-44	146
TcMYB53	ATMYB2	55.26	114	51	0	10	351	11	124	2.00E-43	143
TcMYB53	ATMYB68	52.42	124	58	2	1	369	1	123	3.00E-43	145
TcMYB53	ATMYB38	39.62	212	119	8	1	609	1	202	4.00E-43	143
TcMYB53	ATMYB61	45.45	165	79	3	1	462	1	160	4.00E-43	145
TcMYB53	ATMYB59-3	51.49	134	65	3	37	438	8	132	6.00E-43	141
TcMYB53	AtMYB40	38.12	223	131	7	1	648	1	205	1.00E-42	141
TcMYB53	ATMYB46	49.26	136	65	2	25	420	14	149	1.00E-42	141
TcMYB53	ATMYB95	57.94	107	45	0	28	348	9	115	1.00E-42	141
TcMYB53	AtMYB62	43.71	167	86	1	40	516	20	186	2.00E-42	141
TcMYB53	ATMYB48	38.01	221	133	5	34	684	6	219	5.00E-42	139
TcMYB53	ATMYB57	59.8	102	41	0	40	345	26	127	8.00E-42	137
TcMYB53	ATMYB99	52.46	122	51	2	4	348	3	123	8.00E-42	138
TcMYB53	AtMYB112	43.45	168	85	2	40	513	33	200	2.00E-41	137
TcMYB53	ATMYB3	58.82	102	42	0	40	345	21	122	3.00E-41	136
TcMYB53	AtMYB24	59.8	102	41	0	40	345	18	119	3.00E-41	136
TcMYB53	AtMYB103	53.45	116	54	1	1	348	1	115	4.00E-41	140
TcMYB53	AtMYB83	43.41	182	99	5	40	573	31	192	4.00E-41	139
TcMYB53	ATMYB55	53.12	128	48	2	1	348	1	127	6.00E-41	139
TcMYB53	ATMYB67	50.43	115	57	1	16	360	16	129	7.00E-41	137
TcMYB53	ATMYB101	56.19	105	46	0	34	348	17	121	1.00E-40	140
TcMYB53	AtMYB116	57.69	104	44	0	40	351	19	122	2.00E-40	136
TcMYB53	AtMYB47	53.27	107	50	0	28	348	9	115	3.00E-40	135
TcMYB53	ATMYB120	58.82	102	42	0	40	345	27	128	3.00E-39	137
TcMYB53	AtMYB27	55.36	112	50	0	25	360	5	116	4.00E-39	131
TcMYB53	AtMYB19	39.57	187	102	5	40	567	13	199	6.00E-39	131
TcMYB53	AtMYB108	58.82	102	42	0	40	345	20	121	8.00E-39	132

TcMYB53	AtMYB18	50	120	60	0	40	399	11	130	9.00E-39	131
TcMYB53	AtMYB45	34.93	209	133	3	40	657	19	217	1.00E-38	130
TcMYB53	ATMYB33	53.1	113	51	1	16	348	23	135	3.00E-38	135
TcMYB53	ATMYB65	53.7	108	50	0	25	348	37	144	7.00E-38	134
TcMYB53	ATMYB26	29.85	325	217	12	1	942	1	287	1.00E-37	130
TcMYB53	AtMYB97	52.94	102	48	0	40	345	20	121	8.00E-37	129
TcMYB53	AtMYB81	52.43	103	49	0	37	345	20	122	2.00E-36	128
TcMYB53	ATMYB78	52.59	116	41	1	40	345	27	142	5.00E-35	122
TcMYB53	AtMYB104	39.44	142	83	1	37	453	16	157	1.00E-33	120
TcMYB53	ATMYB105	36.23	138	82	2	22	417	100	236	2.00E-26	99.4
TcMYB53	ATMYB25	33.9	177	113	4	43	561	50	214	6.00E-26	99
TcMYB53	AtMYB117	37.69	130	76	2	22	396	91	219	7.00E-26	98.6
TcMYB53	ATMYB118	42.72	103	59	1	22	330	182	283	4.00E-25	97.4
TcMYB53	ATMYB118	26.19	126	84	5	55	405	144	261	1.00E-04	35.4
TcMYB53	AtMYB115	31.71	164	112	2	43	534	158	317	5.00E-25	96.3
TcMYB53	ATMYB54	34.19	155	96	4	43	489	6	154	7.00E-25	93.6
TcMYB53	AtMYB70	42.16	102	59	1	43	348	13	113	9.00E-25	94.7
TcMYB53	ATMYB73	42.16	102	59	1	43	348	13	113	1.00E-24	94.7
TcMYB53	ATMYB52	41.67	108	63	1	43	366	5	111	1.00E-24	93.2
TcMYB53	AtMYB1	41.67	108	63	1	43	366	55	161	2.00E-24	95.1
TcMYB53	AtMYB98	44.66	103	57	1	22	330	210	311	4.00E-24	94.4
TcMYB53	AtMYB98	28.75	80	53	2	142	369	197	276	2.00E-04	35
TcMYB53	AtMYB64	28.5	193	138	3	43	621	105	269	7.00E-24	93.6
TcMYB53	ATMYB110	32.75	171	113	4	43	549	67	221	8.00E-24	92
TcMYB53	ATMYB44	42.16	102	59	1	43	348	6	106	8.00E-24	92
TcMYB53	AtMYB109	43.69	103	57	2	43	348	56	156	2.00E-23	92.4
TcMYB53	ATMYB91	39.13	115	68	2	40	378	3	116	2.00E-23	92
TcMYB53	ATMYB119	44.79	96	53	1	43	330	105	199	2.00E-23	92.4
TcMYB53	AtMYB100	40.78	103	61	1	25	333	20	121	2.00E-22	86.7
TcMYB53	AtMYB56	36.75	117	74	2	43	393	93	206	2.00E-22	88.2
TcMYB53	ATMYB69	38.53	109	67	1	22	348	12	119	3.00E-22	86.7
TcMYB53	ATMYB77	40.2	102	61	1	43	348	6	106	3.00E-22	87.4
TcMYB53	AtMYB89	37.5	112	70	1	46	381	57	167	1.00E-21	83.6
TcMYB53	AtMYB124	40	100	60	1	52	351	28	126	4.00E-20	82.8
TcMYB53	AtMYB22	36.89	103	65	2	25	333	48	148	2.00E-19	79
TcMYB53	ATMYB88	37.1	124	78	3	52	423	33	149	3.00E-19	80.5
TcMYB54	ATMYB3	58.9	163	63	3	1	477	1	161	5.00E-65	200
TcMYB54	AtMYB6	49.74	195	98	2	1	585	1	185	2.00E-63	195
TcMYB54	ATMYB12	62.34	154	54	2	1	450	1	153	1.00E-62	197
TcMYB54	MYB8	60.42	144	57	1	1	432	1	143	5.00E-62	191
TcMYB54	ATMYB4	54.91	173	76	3	1	513	1	172	2.00E-61	192
TcMYB54	AtMYB32	62.59	147	52	3	1	432	1	146	5.00E-61	190
TcMYB54	ATMYB111	59.63	161	45	3	1	423	1	159	8.00E-61	192
TcMYB54	MYB7	65.65	131	45	1	1	393	1	130	1.00E-60	189
TcMYB54	ATMYB5	57.24	152	65	1	13	468	16	166	5.00E-60	187
TcMYB54	ATMYB123	58.39	161	67	2	7	489	4	152	9.00E-60	186
TcMYB54	ATMYB34	45.45	231	126	3	1	693	1	227	3.00E-59	186
TcMYB54	AtMYB107	46.76	216	112	3	1	639	1	204	3.00E-59	187
TcMYB54	AtMYB74	67.77	121	39	0	1	363	1	121	4.00E-59	187
TcMYB54	AtMYB9	41.2	267	154	6	1	792	1	247	2.00E-58	186
TcMYB54	ATMYB14	64.12	131	47	1	1	393	1	130	2.00E-58	182
TcMYB54	AtMYB41	67.77	121	39	1	1	363	1	120	6.00E-58	182
TcMYB54	ATMYB15	64.34	129	46	1	1	387	1	128	9.00E-58	182
TcMYB54	AtMYB51	57.62	151	63	1	1	450	1	151	1.00E-57	184
TcMYB54	ATMYB13	59.03	144	58	2	1	429	1	143	3.00E-57	179
TcMYB54	ATMYB11	70.09	117	35	1	1	351	1	116	4.00E-57	182
TcMYB54	ATMYB102	66.94	121	40	1	1	363	1	120	3.00E-55	177
TcMYB54	ATMYB96	43.97	232	109	5	1	633	1	226	3.00E-55	177
TcMYB54	ATMYB94	38.81	286	145	6	1	768	1	285	2.00E-54	175
TcMYB54	ATMYB16	62.77	137	51	2	1	411	1	135	4.00E-54	174
TcMYB54	ATMYB80	62.81	121	45	1	1	363	1	120	1.00E-53	172
TcMYB54	AtMYB17	65.29	121	42	1	1	363	1	120	1.00E-53	172
TcMYB54	AtMYB82	60.48	124	46	1	19	381	3	126	2.00E-53	168
TcMYB54	AtMYB85	60	135	54	1	1	405	1	134	4.00E-53	169
TcMYB54	ATMYB29	37.94	311	177	7	1	885	1	288	5.00E-53	171
TcMYB54	ATMYB28	46.91	194	89	3	1	540	1	193	7.00E-53	172
TcMYB54	ATMYB92	38.06	310	163	10	1	843	1	301	9.00E-53	171
TcMYB54	AtMYB93	65.29	121	42	1	1	363	1	120	1.00E-52	171
TcMYB54	ATMYB66	62.2	127	45	2	22	393	8	131	1.00E-52	166
TcMYB54	ATMYB106	61.19	134	52	1	10	411	47	179	1.00E-52	172
TcMYB54	ATMYB35	61.98	121	46	1	1	363	1	120	5.00E-52	168
TcMYB54	AtMYB49	69.3	114	35	0	22	363	7	120	5.00E-52	168
TcMYB54	AtMYB20	50.6	166	82	2	1	498	1	163	5.00E-52	167
TcMYB54	ATMYB122	57.66	137	58	1	1	411	1	136	7.00E-52	168
TcMYB54	ATMYB23	65.14	109	38	0	25	351	8	116	1.00E-51	164
TcMYB54	ATMYB72	62.6	123	46	1	4	372	4	125	1.00E-51	166
TcMYB54	AtMYB53	59.29	140	53	2	1	408	1	139	2.00E-51	167
TcMYB54	ATMYB86	59.85	137	55	1	1	411	1	136	2.00E-51	168
TcMYB54	AtMYB42	67.52	117	38	1	1	351	1	116	2.00E-51	166
TcMYB54	ATMYB58	46.82	173	92	3	4	522	4	166	2.00E-51	165

TcMYB54	AtMYB60	47.89	190	85	5	1	528	1	188	4.00E-51	165
TcMYB54	AtMYB76	56.34	142	62	2	1	426	1	138	4.00E-51	166
TcMYB54	ATMYB0	63.72	113	41	0	43	381	16	128	5.00E-51	163
TcMYB54	AtMYB114	66.67	105	35	0	34	348	7	111	5.00E-51	160
TcMYB54	AtMYB36	48.24	170	87	3	1	507	1	166	7.00E-51	166
TcMYB54	ATMYB30	49.71	175	76	3	1	489	1	172	8.00E-51	165
TcMYB54	AtMYB40	40.43	230	132	4	1	675	1	212	1.00E-50	163
TcMYB54	ATMYB63	52.74	146	69	1	4	441	4	148	1.00E-50	164
TcMYB54	ATMYB61	62.2	127	48	2	1	381	1	125	2.00E-50	166
TcMYB54	AtMYB43	38.65	282	161	9	1	810	1	269	2.00E-50	164
TcMYB54	ATMYB75	66.67	105	35	0	34	348	7	111	5.00E-50	161
TcMYB54	AtMYB50	38.04	276	146	8	1	753	1	266	1.00E-49	162
TcMYB54	ATMYB84	63.33	120	43	2	1	357	1	119	2.00E-49	161
TcMYB54	ATMYB37	58.59	128	52	2	1	381	1	127	2.00E-49	162
TcMYB54	ATMYB90	65.14	109	38	0	34	360	7	115	3.00E-49	159
TcMYB54	AtMYB113	53.24	139	65	2	1	417	1	131	3.00E-49	159
TcMYB54	ATMYB31	63.25	117	43	1	1	351	1	116	2.00E-48	159
TcMYB54	ATMYB67	36.74	264	126	7	16	684	16	273	2.00E-48	159
TcMYB54	ATMYB71	59.17	120	49	0	28	387	15	134	2.00E-48	157
TcMYB54	AtMYB103	61.02	118	46	1	1	354	1	117	2.00E-48	160
TcMYB54	ATMYB68	61.67	120	45	2	1	357	1	119	2.00E-48	160
TcMYB54	AtMYB10	54.01	137	63	1	7	417	5	140	4.00E-48	155
TcMYB54	ATMYB46	55.22	134	51	1	37	411	18	151	5.00E-48	157
TcMYB54	ATMYB38	52.38	147	69	2	1	438	1	146	5.00E-48	157
TcMYB54	ATMYB87	51.7	147	66	3	1	426	1	146	6.00E-48	157
TcMYB54	ATMYB57	55.04	129	58	0	25	411	21	149	9.00E-48	154
TcMYB54	ATMYB3	61.06	113	44	0	22	360	15	127	1.00E-47	154
TcMYB54	ATMYB2	57.76	116	49	0	10	357	11	126	3.00E-47	154
TcMYB54	AtMYB83	33.86	316	190	8	37	927	30	309	3.00E-47	156
TcMYB54	AtMYB24	59.48	116	47	0	22	369	12	127	4.00E-47	152
TcMYB54	AtMYB79	60	115	46	0	43	387	8	122	8.00E-47	153
TcMYB54	ATMYB95	52.67	150	61	3	1	420	1	149	1.00E-46	153
TcMYB54	ATMYB55	50.31	163	66	3	1	444	1	162	1.00E-46	155
TcMYB54	ATMYB99	48.5	167	79	4	4	483	3	164	2.00E-46	152
TcMYB54	ATMYB121	38.69	199	119	3	25	612	23	212	3.00E-46	152
TcMYB54	AtMYB112	64.15	106	38	0	37	354	32	137	2.00E-45	149
TcMYB54	AtMYB47	50	156	64	3	1	426	1	155	3.00E-45	149
TcMYB54	ATMYB26	40	230	115	6	1	621	1	216	6.00E-45	151
TcMYB54	AtMYB62	59.46	111	45	0	22	354	14	124	2.00E-43	145
TcMYB54	ATMYB101	58.1	105	44	0	34	348	17	121	6.00E-43	148
TcMYB54	ATMYB120	49.25	134	68	0	37	438	26	159	6.00E-43	149
TcMYB54	AtMYB108	62.26	106	40	0	37	354	19	124	7.00E-43	144
TcMYB54	AtMYB116	59.62	104	42	0	43	354	20	123	5.00E-42	141
TcMYB54	ATMYB48	40.23	174	104	1	25	546	3	163	2.00E-41	139
TcMYB54	AtMYB81	57.01	107	46	1	43	363	22	127	3.00E-40	140
TcMYB54	AtMYB97	45	140	77	1	37	456	19	157	4.00E-40	139
TcMYB54	ATMYB33	53.39	118	53	2	16	363	23	139	1.00E-39	140
TcMYB54	ATMYB65	53.98	113	52	1	25	363	37	148	1.00E-39	140
TcMYB54	ATMYB78	53.23	124	44	1	25	354	22	145	2.00E-39	135
TcMYB54	AtMYB18	54.4	125	57	1	43	417	12	135	2.00E-39	134
TcMYB54	ATMYB59-3	48.78	123	63	0	43	411	10	132	4.00E-39	132
TcMYB54	AtMYB19	52	125	60	1	43	417	14	135	2.00E-38	131
TcMYB54	AtMYB27	56.48	108	47	0	25	348	5	112	2.00E-38	130
TcMYB54	AtMYB45	52.48	101	48	0	43	345	20	120	6.00E-35	122
TcMYB54	AtMYB104	32.49	237	155	7	43	738	18	219	3.00E-32	117
TcMYB54	AtMYB117	41.6	125	73	1	22	396	91	214	1.00E-29	109
TcMYB54	ATMYB105	35.4	161	102	4	22	498	100	254	6.00E-29	107
TcMYB54	ATMYB118	45.63	103	56	1	22	330	182	283	1.00E-27	105
TcMYB54	ATMYB118	30	60	41	1	187	363	184	243	2.00E-04	35
TcMYB54	ATMYB119	42.19	128	73	3	43	423	105	229	3.00E-27	104
TcMYB54	AtMYB64	40.98	122	72	2	37	402	103	221	1.00E-26	102
TcMYB54	ATMYB73	44.04	109	61	1	43	369	13	120	2.00E-26	100
TcMYB54	ATMYB73	31.25	64	43	1	202	390	13	76	3.00E-04	34.3
TcMYB54	AtMYB98	45.63	103	56	1	22	330	210	311	4.00E-26	100
TcMYB54	AtMYB98	29.17	72	50	1	142	354	197	268	1.00E-04	35.8
TcMYB54	AtMYB70	42.99	107	61	1	43	363	13	118	5.00E-26	99
TcMYB54	AtMYB70	31.25	64	43	1	202	390	13	76	3.00E-04	34.3
TcMYB54	ATMYB54	42.06	107	62	1	40	360	5	110	7.00E-26	97.1
TcMYB54	ATMYB52	41.96	112	65	1	40	375	4	114	8.00E-26	97.1
TcMYB54	AtMYB115	37.1	124	74	2	37	396	156	278	1.00E-25	98.6
TcMYB54	AtMYB100	40.65	123	72	2	25	390	20	141	2.00E-25	95.5
TcMYB54	AtMYB109	38.46	156	82	6	4	429	40	193	3.00E-25	98.2
TcMYB54	ATMYB110	40	110	66	1	40	369	66	174	3.00E-25	96.7
TcMYB54	AtMYB1	42.24	116	61	2	43	372	55	169	5.00E-25	97.4
TcMYB54	ATMYB69	38.35	133	81	2	22	417	12	143	1.00E-24	94
TcMYB54	ATMYB25	45.63	103	55	2	43	348	50	150	1.00E-24	95.9
TcMYB54	ATMYB44	44.12	102	57	1	43	348	6	106	2.00E-24	94.7
TcMYB54	ATMYB77	39.37	127	77	2	43	423	6	129	4.00E-24	93.6
TcMYB54	AtMYB124	33.9	177	111	7	52	564	28	187	2.00E-23	93.6
TcMYB54	AtMYB56	40	115	64	2	31	360	84	197	5.00E-23	90.9

TcMYB54	AtMYB89	38.14	118	72	2	46	396	57	173	8.00E-23	87.4
TcMYB54	AtMYB22	33.06	121	81	1	25	387	48	166	1.00E-20	82.8
TcMYB54	ATMYB91	34.45	119	76	1	43	393	4	122	1.00E-20	84.3
TcMYB54	ATMYB88	39.45	109	66	1	52	378	33	140	3.00E-20	84.3
TcMYB55	ATMYB4	64.29	154	53	1	1	456	1	154	2.00E-70	214
TcMYB55	ATMYB111	80.17	116	23	0	1	348	1	116	6.00E-69	212
TcMYB55	ATMYB12	81.03	116	22	0	1	348	1	116	4.00E-68	211
TcMYB55	MYB7	69.29	127	39	0	1	381	1	127	2.00E-66	203
TcMYB55	AtMYB6	68.5	127	40	0	1	381	1	127	2.00E-66	202
TcMYB55	ATMYB3	68.5	127	40	0	1	381	1	127	3.00E-66	202
TcMYB55	MYB8	67.46	126	41	0	1	378	1	126	8.00E-66	199
TcMYB55	ATMYB5	68.8	125	39	0	10	384	15	139	3.00E-65	199
TcMYB55	AtMYB32	62.41	141	53	0	1	423	1	141	4.00E-65	200
TcMYB55	AtMYB107	66.41	128	43	0	1	384	1	128	2.00E-63	197
TcMYB55	ATMYB11	41.91	272	150	5	1	792	1	249	3.00E-63	197
TcMYB55	AtMYB9	67.19	128	42	0	1	384	1	128	5.00E-63	196
TcMYB55	AtMYB17	40.15	274	159	5	1	807	1	269	7.00E-63	195
TcMYB55	AtMYB41	64.06	128	46	0	1	384	1	128	1.00E-61	191
TcMYB55	ATMYB29	36.47	329	195	7	1	945	1	322	2.00E-61	192
TcMYB55	ATMYB102	65.62	128	44	0	1	384	1	128	2.00E-61	192
TcMYB55	ATMYB16	64.84	128	45	0	1	384	1	128	2.00E-61	192
TcMYB55	ATMYB34	55.26	152	68	1	1	456	1	148	5.00E-61	190
TcMYB55	ATMYB80	56.58	152	66	0	1	456	1	152	6.00E-61	191
TcMYB55	AtMYB20	46.12	232	104	4	1	633	1	221	1.00E-60	188
TcMYB55	AtMYB93	64.84	128	45	0	1	384	1	128	5.00E-60	189
TcMYB55	AtMYB74	64.34	129	45	1	1	384	1	129	7.00E-60	188
TcMYB55	AtMYB40	38.63	277	169	7	1	828	1	257	2.00E-59	185
TcMYB55	ATMYB15	57.24	152	65	2	1	456	1	144	7.00E-59	184
TcMYB55	AtMYB43	65.62	128	44	0	1	384	1	128	1.00E-58	185
TcMYB55	ATMYB28	61.72	128	49	0	1	384	1	128	3.00E-58	185
TcMYB55	AtMYB76	43.23	229	117	5	1	648	1	223	9.00E-58	183
TcMYB55	AtMYB85	58.04	143	60	0	1	429	1	143	9.00E-58	181
TcMYB55	ATMYB106	63.71	124	45	0	10	381	47	170	2.00E-57	183
TcMYB55	AtMYB42	56.95	151	65	1	1	453	1	150	1.00E-56	178
TcMYB55	AtMYB49	63.28	128	47	0	1	384	1	128	2.00E-56	179
TcMYB55	ATMYB86	36.39	305	191	6	1	906	1	269	3.00E-56	179
TcMYB55	AtMYB51	60.94	128	49	1	1	381	1	128	5.00E-56	179
TcMYB55	ATMYB92	60.16	128	51	0	1	384	1	128	7.00E-56	178
TcMYB55	AtMYB53	60.16	128	51	0	1	384	1	128	7.00E-56	177
TcMYB55	ATMYB122	54.42	147	67	0	1	441	1	147	9.00E-56	177
TcMYB55	ATMYB13	65.52	116	40	0	1	348	1	116	1.00E-55	174
TcMYB55	ATMYB14	64.1	117	42	0	1	351	1	117	1.00E-55	174
TcMYB55	ATMYB61	52.05	171	79	2	1	504	1	171	2.00E-55	177
TcMYB55	AtMYB50	60.94	128	50	0	1	384	1	128	2.00E-54	173
TcMYB55	ATMYB35	42.08	221	114	4	1	621	1	221	3.00E-54	173
TcMYB55	ATMYB99	59.26	135	48	1	4	387	3	137	6.00E-53	167
TcMYB55	AtMYB60	42.44	205	118	3	1	615	1	193	8.00E-53	168
TcMYB55	ATMYB67	34.35	294	169	8	16	825	16	305	2.00E-52	168
TcMYB55	ATMYB95	57.03	128	55	0	1	384	1	128	2.00E-51	164
TcMYB55	ATMYB123	52.12	165	75	3	34	516	14	174	3.00E-51	164
TcMYB55	ATMYB31	61.21	116	45	0	1	348	1	116	3.00E-51	166
TcMYB55	AtMYB47	55.47	128	57	0	1	384	1	128	3.00E-51	164
TcMYB55	ATMYB55	57.55	139	47	1	1	381	1	139	7.00E-51	165
TcMYB55	ATMYB94	59.48	116	47	0	1	348	1	116	1.00E-50	164
TcMYB55	ATMYB72	62.07	116	44	0	4	351	4	119	1.00E-50	163
TcMYB55	ATMYB58	62.07	116	44	0	4	351	4	119	1.00E-50	162
TcMYB55	ATMYB63	52.63	152	71	2	4	456	4	151	1.00E-50	163
TcMYB55	AtMYB82	56.15	130	48	1	22	384	8	137	2.00E-50	159
TcMYB55	ATMYB96	49.67	151	76	1	1	453	1	137	3.00E-50	164
TcMYB55	ATMYB30	59.83	117	47	0	1	351	1	117	4.00E-50	162
TcMYB55	AtMYB103	49.02	153	78	1	1	459	1	150	7.00E-49	160
TcMYB55	AtMYB10	46.2	171	91	2	7	516	5	163	7.00E-49	157
TcMYB55	ATMYB66	46.45	155	78	3	37	486	17	169	1.00E-48	155
TcMYB55	ATMYB23	61.54	104	40	0	37	348	13	116	2.00E-47	152
TcMYB55	ATMYB87	30.36	303	195	6	1	861	1	289	3.00E-47	154
TcMYB55	AtMYB114	60	105	42	0	31	345	7	111	1.00E-46	147
TcMYB55	ATMYB75	52.38	126	52	1	31	384	7	132	2.00E-45	148
TcMYB55	ATMYB37	48.3	147	72	2	1	429	1	147	2.00E-45	150
TcMYB55	AtMYB113	35.19	287	179	8	1	840	1	243	3.00E-45	147
TcMYB55	AtMYB83	59.09	110	45	0	25	354	27	136	7.00E-45	149
TcMYB55	ATMYB90	60	105	42	0	31	345	7	111	7.00E-45	147
TcMYB55	ATMYB0	58.65	104	43	0	37	348	15	118	8.00E-45	146
TcMYB55	ATMYB26	54.4	125	48	1	1	348	1	125	2.00E-44	149
TcMYB55	ATMYB38	51.97	127	60	1	1	378	1	127	2.00E-44	147
TcMYB55	ATMYB46	58.1	105	44	0	34	348	18	122	8.00E-44	145
TcMYB55	AtMYB36	53.33	120	55	1	1	357	1	120	2.00E-43	145
TcMYB55	ATMYB101	58.1	105	44	0	31	345	17	121	3.00E-43	148
TcMYB55	ATMYB84	51.67	120	57	1	1	357	1	120	3.00E-43	144
TcMYB55	ATMYB68	52.5	120	56	1	1	357	1	120	4.00E-43	145
TcMYB55	ATMYB71	58.1	105	44	0	31	345	17	121	7.00E-43	142

TcMYB55	AtMYB79	58.25	103	43	0	37	345	7	109	8.00E-43	142
TcMYB55	ATMYB120	58.72	109	45	0	22	348	22	130	3.00E-42	146
TcMYB55	ATMYB57	53.45	116	54	0	34	381	25	140	1.00E-41	137
TcMYB55	ATMYB121	55.77	104	46	0	31	342	26	129	2.00E-41	138
TcMYB55	AtMYB108	48.28	145	75	1	25	459	16	155	1.00E-40	138
TcMYB55	AtMYB116	50.43	115	57	0	37	381	19	133	1.00E-40	137
TcMYB55	AtMYB24	53.1	113	53	0	22	360	13	125	1.00E-40	134
TcMYB55	AtMYB62	51.69	118	57	0	34	387	19	136	3.00E-40	135
TcMYB55	AtMYB97	55.14	107	48	0	22	342	15	121	5.00E-40	137
TcMYB55	ATMYB3	51.82	110	53	0	34	363	20	129	5.00E-40	133
TcMYB55	AtMYB112	53.77	106	49	0	25	342	29	134	6.00E-40	134
TcMYB55	ATMYB33	54.81	104	47	0	34	345	32	135	1.00E-39	139
TcMYB55	ATMYB2	52.88	104	49	0	34	345	20	123	1.00E-39	134
TcMYB55	AtMYB81	53.77	106	49	0	25	342	17	122	1.00E-38	135
TcMYB55	ATMYB65	53.85	104	48	0	34	345	41	144	1.00E-38	136
TcMYB55	AtMYB18	56.31	103	45	0	37	345	11	113	7.00E-38	129
TcMYB55	ATMYB48	50.46	109	54	0	31	357	6	114	1.00E-36	125
TcMYB55	ATMYB59-3	50.47	107	53	0	37	357	9	115	2.00E-36	124
TcMYB55	AtMYB19	53.4	103	48	0	37	345	13	115	4.00E-36	124
TcMYB55	AtMYB27	54.81	104	47	0	34	345	9	112	9.00E-36	122
TcMYB55	ATMYB78	30.34	323	211	10	25	951	23	308	1.00E-35	124
TcMYB55	AtMYB45	48.04	102	53	0	37	342	19	120	3.00E-34	119
TcMYB55	AtMYB64	28.46	267	175	7	40	792	105	340	9.00E-29	107
TcMYB55	AtMYB104	43.52	108	61	1	40	363	18	124	2.00E-28	106
TcMYB55	ATMYB73	44.86	107	59	1	40	360	13	118	3.00E-28	104
TcMYB55	AtMYB70	44.86	107	59	1	40	360	13	118	5.00E-28	103
TcMYB55	AtMYB109	31.44	194	110	4	40	552	56	243	2.00E-27	103
TcMYB55	ATMYB44	42.02	119	65	3	40	384	6	123	1.00E-25	97.4
TcMYB55	AtMYB98	35.16	182	111	4	25	549	212	375	2.00E-25	98.6
TcMYB55	ATMYB118	38.36	146	90	3	25	462	184	309	2.00E-25	98.6
TcMYB55	ATMYB77	42.31	104	60	1	40	351	6	108	3.00E-25	96.3
TcMYB55	ATMYB25	43.4	106	60	1	40	357	50	154	3.00E-25	97.1
TcMYB55	ATMYB119	42.59	108	62	1	34	357	103	209	4.00E-25	97.4
TcMYB55	ATMYB119	31.03	58	39	1	190	360	102	159	1.00E-04	35.8
TcMYB55	AtMYB1	40.95	105	62	1	40	354	55	158	1.00E-24	95.9
TcMYB55	AtMYB115	39.62	106	64	1	40	357	158	262	1.00E-23	92.4
TcMYB55	ATMYB54	42.72	103	58	2	40	345	6	106	2.00E-23	90.1
TcMYB55	ATMYB105	37.93	116	69	2	19	357	97	211	3.00E-23	90.9
TcMYB55	ATMYB52	39.62	106	64	1	40	357	5	109	5.00E-23	89
TcMYB55	ATMYB110	38.83	103	63	1	40	348	67	168	3.00E-22	87.8
TcMYB55	AtMYB117	33.82	136	90	2	34	441	96	223	4.00E-22	88.2
TcMYB55	ATMYB69	37.38	107	67	1	25	345	14	119	9.00E-22	85.5
TcMYB55	AtMYB100	37.29	118	72	2	25	372	21	137	2.00E-20	81.6
TcMYB55	AtMYB56	37.17	113	66	2	22	345	82	193	6.00E-20	81.6
TcMYB55	ATMYB88	25	308	214	8	49	921	33	323	1.00E-19	82
TcMYB55	AtMYB124	31.94	144	93	2	49	465	28	170	1.00E-19	81.6
TcMYB55	ATMYB91	32.26	124	77	2	37	387	3	126	7.00E-19	79
TcMYB55	AtMYB89	36	100	64	1	43	342	57	155	2.00E-18	75.1
TcMYB55	AtMYB22	33.04	112	75	2	25	360	49	158	5.00E-16	69.3
TcMYB56	ATMYB16	59.84	249	94	3	1	729	1	226	7.00E-102	298
TcMYB56	ATMYB106	62.66	233	87	3	10	708	47	265	2.00E-100	297
TcMYB56	AtMYB17	50	286	136	7	1	837	1	279	4.00E-86	257
TcMYB56	AtMYB107	75.56	135	33	0	1	405	1	135	3.00E-79	240
TcMYB56	AtMYB93	55.25	219	77	4	1	594	1	216	3.00E-78	239
TcMYB56	AtMYB9	49.8	255	124	5	1	753	1	247	4.00E-77	235
TcMYB56	AtMYB53	53.64	220	95	3	1	639	1	219	4.00E-75	229
TcMYB56	AtMYB74	67.79	149	47	1	1	444	1	149	4.00E-74	227
TcMYB56	ATMYB102	65.56	151	50	1	1	447	1	151	2.00E-73	226
TcMYB56	AtMYB43	49.03	257	121	5	1	741	1	244	9.00E-73	224
TcMYB56	ATMYB92	72.39	134	37	0	1	402	1	134	2.00E-72	223
TcMYB56	AtMYB41	61.35	163	63	1	1	489	1	159	1.00E-71	219
TcMYB56	ATMYB96	43.53	278	148	6	1	807	1	267	6.00E-70	217
TcMYB56	ATMYB28	72.39	134	37	0	1	402	1	134	2.00E-69	216
TcMYB56	ATMYB29	70.14	144	43	1	1	432	1	143	3.00E-69	215
TcMYB56	AtMYB49	72.09	129	36	0	1	387	1	129	5.00E-69	214
TcMYB56	AtMYB20	73.08	130	35	0	1	390	1	130	2.00E-68	211
TcMYB56	ATMYB15	52.15	209	99	5	1	624	1	198	4.00E-68	210
TcMYB56	ATMYB14	77.97	118	26	0	1	354	1	118	2.00E-67	207
TcMYB56	AtMYB85	70.99	131	38	0	1	393	1	131	4.00E-67	207
TcMYB56	AtMYB76	71.21	132	38	0	1	396	1	132	8.00E-67	209
TcMYB56	AtMYB32	67.42	132	43	0	1	396	1	132	1.00E-66	206
TcMYB56	AtMYB51	36.51	367	218	8	1	1056	1	345	1.00E-66	209
TcMYB56	ATMYB34	66.91	136	45	0	1	408	1	136	4.00E-66	206
TcMYB56	MYB8	61.9	147	52	1	1	429	1	147	4.00E-66	202
TcMYB56	AtMYB60	59.35	155	63	1	1	465	1	150	6.00E-66	204
TcMYB56	AtMYB42	66.22	148	47	1	1	435	1	148	8.00E-66	204
TcMYB56	ATMYB30	56.89	167	72	1	1	501	1	166	2.00E-65	205
TcMYB56	AtMYB6	65.19	135	47	0	1	405	1	135	3.00E-65	201
TcMYB56	ATMYB94	56.14	171	71	3	1	501	1	170	4.00E-65	204
TcMYB56	MYB7	65.91	132	45	0	1	396	1	132	5.00E-65	202

TcMYB56	ATMYB4	65.15	132	46	0	1	396	1	132	8.00E-65	202
TcMYB56	ATMYB80	67.69	130	42	0	1	390	1	130	1.00E-64	202
TcMYB56	ATMYB122	42.91	275	153	6	1	813	1	248	1.00E-64	203
TcMYB56	ATMYB3	64.39	132	47	0	1	396	1	132	2.00E-63	197
TcMYB56	ATMYB86	63.33	150	51	1	1	438	1	150	6.00E-63	199
TcMYB56	ATMYB31	50.79	189	86	3	1	546	1	188	9.00E-63	198
TcMYB56	AtMYB50	63.89	144	52	1	1	432	1	141	1.00E-62	197
TcMYB56	ATMYB13	75.86	116	28	0	1	348	1	116	1.00E-62	195
TcMYB56	ATMYB5	67.74	124	40	0	13	384	16	139	2.00E-62	194
TcMYB56	ATMYB35	64.12	131	47	0	1	393	1	131	2.00E-61	194
TcMYB56	ATMYB58	72.41	116	32	0	4	351	4	119	2.00E-61	192
TcMYB56	AtMYB40	54.78	157	70	1	1	468	1	157	5.00E-61	191
TcMYB56	ATMYB61	66.41	131	44	0	1	393	1	131	7.00E-61	194
TcMYB56	ATMYB72	56.1	164	72	2	4	495	4	156	2.00E-60	191
TcMYB56	ATMYB63	60.84	143	56	0	4	432	4	146	3.00E-60	190
TcMYB56	ATMYB95	53.04	181	75	1	1	513	1	181	3.00E-59	187
TcMYB56	ATMYB111	69.83	116	35	0	1	348	1	116	6.00E-59	188
TcMYB56	ATMYB99	62.41	133	43	1	4	381	3	135	1.00E-58	184
TcMYB56	AtMYB10	57.72	149	63	1	7	453	5	150	8.00E-58	182
TcMYB56	AtMYB47	53.94	165	74	1	1	489	1	165	9.00E-58	183
TcMYB56	ATMYB11	68.97	116	36	0	1	348	1	116	4.00E-57	184
TcMYB56	ATMYB12	67.24	116	38	0	1	348	1	116	6.00E-57	184
TcMYB56	ATMYB55	61.54	143	43	1	1	393	1	143	1.00E-56	182
TcMYB56	ATMYB67	62.81	121	45	0	16	378	16	136	1.00E-55	179
TcMYB56	AtMYB103	59.38	128	52	0	1	384	1	128	1.00E-52	173
TcMYB56	AtMYB36	54.43	158	63	2	1	447	1	158	2.00E-52	171
TcMYB56	ATMYB84	66.67	117	38	1	1	348	1	117	5.00E-52	169
TcMYB56	ATMYB37	61.79	123	46	1	1	366	1	123	5.00E-52	170
TcMYB56	ATMYB26	60.8	125	40	1	1	348	1	125	7.00E-52	171
TcMYB56	ATMYB87	56.55	145	61	3	1	429	1	143	2.00E-51	167
TcMYB56	ATMYB38	56	150	64	3	1	444	1	145	3.00E-51	167
TcMYB56	ATMYB68	53.5	157	62	3	1	438	1	155	6.00E-51	168
TcMYB56	ATMYB46	57.46	134	57	0	34	435	18	151	9.00E-50	162
TcMYB56	AtMYB83	68.81	109	34	0	22	348	26	134	1.00E-49	164
TcMYB56	ATMYB101	33.44	302	197	5	31	924	17	305	7.00E-48	163
TcMYB56	AtMYB81	51.45	138	67	1	25	438	17	153	8.00E-47	159
TcMYB56	AtMYB24	56.91	123	53	0	34	402	17	139	2.00E-46	152
TcMYB56	ATMYB66	62.5	104	39	0	37	348	17	120	5.00E-46	150
TcMYB56	ATMYB123	66.36	107	36	0	34	354	14	120	9.00E-46	151
TcMYB56	AtMYB112	57.89	114	48	0	25	366	29	142	2.00E-45	150
TcMYB56	ATMYB3	51.13	133	65	0	34	432	20	152	3.00E-45	149
TcMYB56	ATMYB23	61.54	104	40	0	37	348	13	116	3.00E-45	149
TcMYB56	ATMYB33	60.19	108	43	0	22	345	28	135	4.00E-45	156
TcMYB56	ATMYB57	50	134	67	0	34	435	25	158	9.00E-45	147
TcMYB56	AtMYB82	55.56	126	47	1	34	384	12	137	1.00E-44	146
TcMYB56	ATMYB71	62.86	105	39	0	31	345	17	121	2.00E-44	148
TcMYB56	ATMYB0	60.58	104	41	0	37	348	15	118	4.00E-44	146
TcMYB56	AtMYB79	63.11	103	38	0	37	345	7	109	5.00E-44	147
TcMYB56	ATMYB65	60.58	104	41	0	34	345	41	144	2.00E-43	152
TcMYB56	ATMYB120	60.91	110	43	1	34	363	26	134	2.00E-43	151
TcMYB56	AtMYB108	55.65	115	51	0	22	366	15	129	3.00E-43	147
TcMYB56	AtMYB19	55.65	124	49	1	37	390	13	136	4.00E-43	145
TcMYB56	ATMYB121	61.54	104	40	0	31	342	26	129	6.00E-43	144
TcMYB56	AtMYB114	60	105	42	0	31	345	7	111	1.00E-42	139
TcMYB56	AtMYB116	54.78	115	52	0	37	381	19	133	1.00E-42	144
TcMYB56	ATMYB75	50	142	54	2	31	405	7	148	3.00E-42	142
TcMYB56	AtMYB45	46.71	152	64	2	37	441	19	170	3.00E-42	142
TcMYB56	AtMYB97	55.36	112	50	1	28	363	17	127	1.00E-41	144
TcMYB56	AtMYB18	59.43	106	43	0	37	354	11	116	2.00E-41	140
TcMYB56	ATMYB2	49.19	124	63	0	34	405	20	143	4.00E-41	139
TcMYB56	ATMYB90	50.68	148	55	3	31	420	7	154	5.00E-41	139
TcMYB56	AtMYB62	53.45	116	54	0	34	381	19	134	3.00E-40	137
TcMYB56	AtMYB113	54.78	115	52	1	1	345	1	111	2.00E-39	134
TcMYB56	ATMYB48	54.29	105	48	0	31	345	6	110	1.00E-38	132
TcMYB56	ATMYB59-3	54.37	103	47	0	37	345	9	111	3.00E-38	131
TcMYB56	AtMYB27	57.69	104	44	0	34	345	9	112	4.00E-38	130
TcMYB56	ATMYB78	47.29	129	54	1	22	366	22	150	7.00E-38	132
TcMYB56	AtMYB104	47.22	108	57	1	40	363	18	124	6.00E-31	114
TcMYB56	AtMYB70	48.6	107	55	1	40	360	13	118	7.00E-30	110
TcMYB56	ATMYB73	47.66	107	56	1	40	360	13	118	2.00E-29	109
TcMYB56	ATMYB44	42.52	127	69	3	40	408	6	131	2.00E-28	106
TcMYB56	ATMYB119	40.91	132	78	2	34	429	103	228	8.00E-28	106
TcMYB56	ATMYB77	46.15	104	56	1	40	351	6	108	1.00E-27	103
TcMYB56	ATMYB25	49.02	102	52	1	40	345	50	150	9.00E-27	102
TcMYB56	AtMYB98	45.45	110	60	1	25	354	212	320	1.00E-26	103
TcMYB56	AtMYB1	47.17	106	52	2	40	345	55	155	2.00E-26	102
TcMYB56	AtMYB64	42.86	105	60	1	40	354	105	208	4.00E-26	101
TcMYB56	ATMYB118	44.64	112	60	2	25	354	184	292	5.00E-26	101
TcMYB56	ATMYB118	30	60	41	1	184	360	184	243	1.00E-04	35.8
TcMYB56	ATMYB52	45.1	102	56	1	40	345	5	105	8.00E-26	97.8

TcMYB56	ATMYB54	39.86	138	80	3	40	444	6	140	3.00E-25	96.3
TcMYB56	AtMYB115	43.69	103	58	2	40	348	158	258	5.00E-25	97.8
TcMYB56	AtMYB109	45.28	106	58	1	40	357	56	160	1.00E-24	97.4
TcMYB56	ATMYB105	33.12	160	96	4	25	471	102	256	2.00E-23	92.4
TcMYB56	ATMYB69	38.32	107	66	1	25	345	14	119	9.00E-23	89.4
TcMYB56	AtMYB117	41.35	104	61	1	34	345	96	198	2.00E-22	90.1
TcMYB56	AtMYB100	38.14	118	71	2	25	372	21	137	4.00E-21	84.3
TcMYB56	AtMYB56	40.2	102	61	1	40	345	93	193	1.00E-20	84.7
TcMYB56	AtMYB89	35.45	110	71	1	43	372	57	165	1.00E-19	79
TcMYB56	ATMYB110	26.9	197	127	6	40	579	67	261	2.00E-19	80.5
TcMYB56	ATMYB91	35.64	101	63	1	49	345	7	107	6.00E-18	77
TcMYB56	AtMYB22	33.93	112	74	1	25	360	49	158	2.00E-17	74.3
TcMYB56	ATMYB88	38.38	99	61	1	49	345	33	130	6.00E-17	74.7
TcMYB56	AtMYB124	38.38	99	61	1	49	345	28	125	4.00E-16	72
TcMYB57	ATMYB23	65.42	107	37	0	19	339	10	116	1.00E-53	167
TcMYB57	ATMYB66	58.4	125	52	1	19	393	14	131	6.00E-53	165
TcMYB57	ATMYB123	55.56	144	62	1	10	435	9	152	3.00E-52	165
TcMYB57	AtMYB82	63.96	111	40	0	31	363	14	124	5.00E-52	162
TcMYB57	MYB7	57.04	135	48	1	16	390	9	143	2.00E-51	163
TcMYB57	ATMYB3	67.31	104	34	0	28	339	13	116	2.00E-50	160
TcMYB57	AtMYB32	50.34	149	72	2	28	468	13	160	3.00E-50	160
TcMYB57	AtMYB6	65.38	104	36	0	28	339	13	116	6.00E-50	158
TcMYB57	ATMYB4	60.83	120	45	1	28	381	13	132	1.00E-49	159
TcMYB57	ATMYB0	62.62	107	40	0	25	345	14	120	2.00E-49	157
TcMYB57	ATMYB5	68	100	32	0	40	339	28	127	5.00E-49	156
TcMYB57	AtMYB114	69.7	99	30	0	40	336	13	111	2.00E-48	151
TcMYB57	MYB8	61.54	104	40	0	28	339	13	116	5.00E-48	152
TcMYB57	AtMYB49	70	100	30	0	40	339	17	116	6.00E-48	155
TcMYB57	ATMYB46	52.17	138	66	1	37	450	22	158	3.00E-47	152
TcMYB57	AtMYB74	67	100	33	0	40	339	18	117	4.00E-47	154
TcMYB57	ATMYB86	35.42	271	165	6	16	798	9	264	5.00E-47	154
TcMYB57	AtMYB51	36.23	276	170	6	40	849	18	278	1.00E-46	153
TcMYB57	ATMYB75	61.54	117	38	1	40	369	13	129	2.00E-46	150
TcMYB57	ATMYB102	35.23	281	162	6	40	822	17	288	2.00E-46	152
TcMYB57	AtMYB41	66	100	34	0	40	339	17	116	4.00E-46	150
TcMYB57	ATMYB12	65	100	35	0	40	339	17	116	4.00E-46	152
TcMYB57	ATMYB106	37.94	253	157	5	40	798	60	281	6.00E-46	152
TcMYB57	ATMYB111	64	100	36	0	40	339	17	116	6.00E-46	151
TcMYB57	ATMYB90	43.81	194	91	5	40	567	13	192	1.00E-45	147
TcMYB57	ATMYB67	51.52	132	58	1	16	393	19	150	1.00E-45	149
TcMYB57	ATMYB15	47.86	140	73	0	40	459	17	156	2.00E-45	148
TcMYB57	AtMYB107	65	100	35	0	40	339	17	116	2.00E-45	149
TcMYB57	ATMYB11	62.75	102	38	0	40	345	17	118	3.00E-45	149
TcMYB57	AtMYB19	34.04	285	163	9	13	792	8	266	6.00E-45	146
TcMYB57	ATMYB80	60	110	44	0	16	345	9	118	7.00E-45	147
TcMYB57	AtMYB53	64	100	36	0	40	339	17	116	1.00E-44	147
TcMYB57	AtMYB50	32.32	297	173	7	16	822	9	292	1.00E-44	147
TcMYB57	AtMYB83	54.62	119	54	0	37	393	34	152	2.00E-44	147
TcMYB57	AtMYB60	35.53	228	139	3	40	699	17	243	2.00E-44	145
TcMYB57	ATMYB16	67	100	33	0	40	339	17	116	2.00E-44	146
TcMYB57	ATMYB94	58.72	109	45	0	40	366	17	125	3.00E-44	146
TcMYB57	ATMYB28	54.14	133	61	2	40	438	17	145	4.00E-44	147
TcMYB57	AtMYB17	63	100	37	0	40	339	17	116	5.00E-44	145
TcMYB57	ATMYB61	53.17	126	57	1	16	387	9	134	6.00E-44	146
TcMYB57	AtMYB113	65.66	99	34	0	40	336	13	111	7.00E-44	143
TcMYB57	AtMYB9	64	100	36	0	40	339	17	116	8.00E-44	145
TcMYB57	ATMYB92	62	100	38	0	40	339	17	116	1.00E-43	145
TcMYB57	ATMYB34	62.14	103	39	0	40	348	17	119	1.00E-43	144
TcMYB57	ATMYB30	59.81	107	43	0	40	360	17	123	2.00E-43	144
TcMYB57	ATMYB31	64	100	36	0	40	339	17	116	2.00E-43	144
TcMYB57	AtMYB79	57.94	107	45	0	40	360	11	117	2.00E-43	142
TcMYB57	AtMYB76	65	100	35	0	40	339	17	116	2.00E-43	144
TcMYB57	ATMYB14	62	100	38	0	40	339	17	116	3.00E-43	141
TcMYB57	ATMYB96	55.08	118	53	0	40	393	17	134	3.00E-43	144
TcMYB57	ATMYB37	60	110	43	1	40	366	17	126	4.00E-43	143
TcMYB57	ATMYB29	63.11	103	38	0	40	348	17	119	4.00E-43	143
TcMYB57	ATMYB13	64	100	36	0	40	339	17	116	5.00E-43	140
TcMYB57	ATMYB35	35.41	257	159	6	16	765	9	242	6.00E-43	142
TcMYB57	AtMYB103	60.58	104	41	0	31	342	14	117	6.00E-43	144
TcMYB57	ATMYB71	52.99	117	55	1	40	390	23	135	1.00E-42	140
TcMYB57	ATMYB72	51.13	133	59	1	16	396	11	143	1.00E-42	141
TcMYB57	ATMYB95	47.92	144	75	1	40	471	17	159	2.00E-42	140
TcMYB57	AtMYB93	62	100	38	0	40	339	17	116	2.00E-42	142
TcMYB57	ATMYB63	56.36	110	48	0	40	369	19	128	3.00E-42	140
TcMYB57	ATMYB122	62	100	38	0	40	339	17	116	3.00E-42	141
TcMYB57	AtMYB42	47.77	157	82	3	40	510	17	161	4.00E-42	139
TcMYB57	ATMYB26	33.67	294	184	7	16	864	9	273	6.00E-42	141
TcMYB57	ATMYB121	53.85	117	54	1	40	390	32	146	9.00E-42	138
TcMYB57	ATMYB101	58.42	101	42	0	40	342	23	123	1.00E-41	142
TcMYB57	AtMYB116	59.63	109	44	0	40	366	23	131	1.00E-41	138

TcMYB57	ATMYB58	57.14	105	45	0	40	354	19	123	2.00E-41	137
TcMYB57	ATMYB84	61.54	104	39	1	40	348	17	120	2.00E-41	138
TcMYB57	AtMYB18	59	100	41	0	37	336	14	113	2.00E-41	137
TcMYB57	AtMYB36	60.58	104	40	1	40	348	17	120	3.00E-41	138
TcMYB57	ATMYB59-3	40.52	153	91	1	16	474	5	153	3.00E-41	135
TcMYB57	ATMYB57	43.24	148	81	2	40	474	30	172	4.00E-41	134
TcMYB57	ATMYB55	54.55	121	43	1	16	342	9	129	5.00E-41	138
TcMYB57	ATMYB120	59.41	101	41	0	40	342	31	131	5.00E-41	141
TcMYB57	ATMYB87	58.65	104	42	1	40	348	17	120	7.00E-41	137
TcMYB57	ATMYB3	53.21	109	51	0	40	366	25	133	7.00E-41	134
TcMYB57	ATMYB68	55.08	118	52	2	40	390	17	131	1.00E-40	138
TcMYB57	AtMYB47	38.99	218	114	4	40	636	17	234	3.00E-40	134
TcMYB57	AtMYB27	44.08	152	76	1	22	450	8	159	4.00E-40	133
TcMYB57	AtMYB24	54.21	107	49	0	40	360	22	128	6.00E-40	132
TcMYB57	ATMYB38	30.37	270	171	6	40	798	17	280	6.00E-40	134
TcMYB57	AtMYB85	60	100	40	0	40	339	17	116	8.00E-40	133
TcMYB57	ATMYB48	46.72	137	72	2	28	435	7	139	9.00E-40	132
TcMYB57	AtMYB62	57.8	109	46	0	40	366	24	132	9.00E-40	133
TcMYB57	AtMYB45	54.55	99	45	0	37	333	22	120	1.00E-39	132
TcMYB57	AtMYB10	55.05	109	49	0	40	366	19	127	2.00E-39	131
TcMYB57	AtMYB112	48.53	136	69	2	40	444	37	170	3.00E-39	130
TcMYB57	ATMYB33	50.93	108	53	0	19	342	30	137	1.00E-38	135
TcMYB57	ATMYB2	47.83	115	59	1	40	381	25	139	2.00E-38	129
TcMYB57	AtMYB81	51.92	104	50	0	16	327	17	120	4.00E-38	132
TcMYB57	AtMYB108	34.39	253	155	7	40	765	24	269	4.00E-38	130
TcMYB57	AtMYB20	56.44	101	44	0	40	342	17	117	2.00E-37	127
TcMYB57	ATMYB65	50.5	101	50	0	40	342	46	146	2.00E-37	132
TcMYB57	ATMYB99	54.21	107	42	1	40	339	18	124	4.00E-37	125
TcMYB57	AtMYB43	57.43	101	43	0	40	342	17	117	5.00E-37	127
TcMYB57	AtMYB97	56.25	96	42	0	40	327	24	119	8.00E-37	128
TcMYB57	AtMYB40	34.23	222	135	5	40	672	17	234	5.00E-36	123
TcMYB57	AtMYB104	25.41	303	206	6	16	864	13	314	5.00E-35	123
TcMYB57	ATMYB78	48.06	129	52	2	40	381	31	159	3.00E-34	119
TcMYB57	ATMYB54	42.62	122	70	2	28	393	5	123	4.00E-30	107
TcMYB57	ATMYB52	43.75	112	63	1	28	363	4	114	3.00E-29	105
TcMYB57	ATMYB105	43.75	112	63	1	22	357	104	214	4.00E-29	106
TcMYB57	AtMYB115	39.13	138	82	3	40	447	161	296	5.00E-29	106
TcMYB57	AtMYB1	30.93	236	156	6	40	726	58	273	1.00E-28	106
TcMYB57	ATMYB73	50	96	48	1	40	327	16	110	7.00E-28	102
TcMYB57	ATMYB73	39.22	51	30	2	190	339	13	59	8.00E-05	35.8
TcMYB57	ATMYB69	36.17	141	89	3	22	441	16	149	1.00E-27	100
TcMYB57	AtMYB117	46.53	101	54	1	40	342	101	200	2.00E-27	102
TcMYB57	ATMYB25	49.48	97	48	2	40	327	53	147	3.00E-27	102
TcMYB57	AtMYB70	46.08	102	55	1	40	345	16	116	3.00E-27	100
TcMYB57	AtMYB70	31.03	58	39	1	190	360	13	70	2.00E-04	34.3
TcMYB57	AtMYB56	39.1	133	74	2	28	405	92	223	4.00E-27	100
TcMYB57	AtMYB109	51.04	96	47	1	40	327	59	153	9.00E-27	100
TcMYB57	AtMYB109	39.22	51	30	2	190	339	56	102	4.00E-05	37
TcMYB57	ATMYB44	46.08	102	55	1	40	345	9	109	2.00E-26	98.6
TcMYB57	ATMYB118	38.71	124	72	2	7	366	181	303	2.00E-26	100
TcMYB57	ATMYB110	40	110	66	1	28	357	66	174	5.00E-26	97.4
TcMYB57	AtMYB64	38.58	127	78	2	40	420	108	225	5.00E-25	96.3
TcMYB57	ATMYB119	44.09	93	52	1	40	318	108	199	5.00E-25	96.3
TcMYB57	ATMYB119	34	50	32	1	181	327	102	151	5.00E-06	39.7
TcMYB57	AtMYB98	39.09	110	67	1	40	369	220	328	1.00E-24	95.5
TcMYB57	AtMYB100	26.99	226	155	6	40	687	29	225	3.00E-24	91.3
TcMYB57	ATMYB77	42.16	102	59	1	40	345	9	109	5.00E-24	92
TcMYB57	ATMYB91	42.86	112	62	1	31	360	4	115	7.00E-24	92.4
TcMYB57	ATMYB88	41.46	123	72	4	40	408	33	148	2.00E-22	89.4
TcMYB57	AtMYB124	29.33	208	130	7	40	612	28	230	9.00E-22	87
TcMYB57	AtMYB22	34.62	104	68	2	10	321	47	148	3.00E-20	80.9
TcMYB57	AtMYB89	39.6	101	61	1	40	342	59	158	4.00E-20	79
TcMYB58	ATMYB4	59.05	210	73	3	1	591	1	207	2.00E-87	254
TcMYB58	ATMYB3	59.8	199	75	3	1	582	1	195	5.00E-83	242
TcMYB58	MYB7	60	205	74	4	1	591	1	199	1.00E-81	239
TcMYB58	AtMYB6	68.59	156	49	2	1	468	1	151	4.00E-80	234
TcMYB58	AtMYB32	58.08	198	82	2	1	591	1	194	1.00E-79	234
TcMYB58	MYB8	55.92	211	82	6	1	600	1	205	7.00E-78	227
TcMYB58	AtMYB9	66.42	137	46	1	1	411	1	134	2.00E-67	205
TcMYB58	AtMYB107	63.5	137	50	1	1	411	1	134	2.00E-65	199
TcMYB58	AtMYB85	50.79	189	85	3	1	543	1	188	1.00E-64	196
TcMYB58	ATMYB5	52.81	178	77	3	13	525	16	184	3.00E-64	194
TcMYB58	ATMYB92	65.62	128	44	0	1	384	1	128	5.00E-63	193
TcMYB58	AtMYB17	65.62	128	44	0	1	384	1	128	1.00E-62	191
TcMYB58	AtMYB50	49.49	196	99	2	1	588	1	185	1.00E-62	192
TcMYB58	ATMYB86	50	210	103	5	1	624	1	203	4.00E-62	192
TcMYB58	ATMYB111	74.14	116	30	0	1	348	1	116	4.00E-62	191
TcMYB58	AtMYB41	42.19	237	132	3	1	696	1	231	6.00E-62	189
TcMYB58	ATMYB102	64.84	128	45	0	1	384	1	128	7.00E-62	191
TcMYB58	ATMYB61	54.88	164	73	1	1	489	1	164	1.00E-61	191

TcMYB58	AtMYB42	47.83	184	96	1	1	552	1	180	2.00E-61	188
TcMYB58	ATMYB16	63.64	132	48	0	1	396	1	132	4.00E-61	188
TcMYB58	ATMYB12	73.28	116	31	0	1	348	1	116	5.00E-61	189
TcMYB58	AtMYB43	42.15	242	130	3	1	696	1	238	8.00E-61	187
TcMYB58	AtMYB93	63.28	128	47	0	1	384	1	128	2.00E-60	187
TcMYB58	ATMYB28	48.57	175	90	1	1	525	1	173	2.00E-60	187
TcMYB58	AtMYB74	62.79	129	47	1	1	384	1	129	3.00E-60	186
TcMYB58	AtMYB53	62.5	128	48	0	1	384	1	128	4.00E-60	185
TcMYB58	ATMYB34	62.12	132	50	0	1	396	1	132	7.00E-60	184
TcMYB58	ATMYB80	57.53	146	62	1	1	438	1	142	1.00E-59	184
TcMYB58	ATMYB29	47.67	193	101	3	1	579	1	174	2.00E-59	184
TcMYB58	ATMYB122	52.41	166	72	2	1	477	1	166	2.00E-59	184
TcMYB58	AtMYB51	39.23	260	132	5	1	702	1	260	2.00E-59	184
TcMYB58	ATMYB11	71.55	116	33	0	1	348	1	116	5.00E-59	183
TcMYB58	AtMYB49	64.84	128	45	0	1	384	1	128	1.00E-58	182
TcMYB58	AtMYB20	60.16	128	51	0	1	384	1	128	1.00E-58	181
TcMYB58	ATMYB13	60.9	133	52	1	1	399	1	132	5.00E-58	178
TcMYB58	ATMYB15	44.21	190	106	1	1	570	1	179	5.00E-58	179
TcMYB58	AtMYB40	53.05	164	76	3	1	489	1	159	5.00E-58	178
TcMYB58	ATMYB106	63.71	124	45	0	10	381	47	170	7.00E-58	182
TcMYB58	AtMYB76	40.95	232	137	3	1	696	1	209	2.00E-57	179
TcMYB58	ATMYB14	59.56	136	52	1	1	399	1	136	3.00E-57	176
TcMYB58	ATMYB55	53.8	171	67	3	1	477	1	166	4.00E-57	179
TcMYB58	ATMYB67	46.08	204	108	4	16	621	16	212	7.00E-57	177
TcMYB58	ATMYB99	59.26	135	48	1	4	387	3	137	1.00E-56	174
TcMYB58	ATMYB58	57.45	141	60	1	4	426	4	141	1.00E-56	175
TcMYB58	ATMYB72	47.12	191	101	3	4	576	4	177	2.00E-56	175
TcMYB58	AtMYB10	54.93	142	64	0	7	432	5	146	1.00E-55	172
TcMYB58	AtMYB103	42.01	219	121	2	1	639	1	218	3.00E-55	174
TcMYB58	AtMYB60	63.49	126	46	0	1	378	1	126	3.00E-55	172
TcMYB58	ATMYB63	66.09	115	39	0	4	348	4	118	3.00E-55	172
TcMYB58	ATMYB31	62.6	123	46	0	1	369	1	123	3.00E-54	171
TcMYB58	ATMYB35	56.25	128	56	0	1	384	1	128	3.00E-54	170
TcMYB58	ATMYB123	60.31	131	52	2	37	429	15	139	7.00E-54	167
TcMYB58	ATMYB30	64.23	123	44	0	1	369	1	123	8.00E-54	169
TcMYB58	ATMYB66	48.47	163	80	2	25	501	13	173	8.00E-54	166
TcMYB58	ATMYB94	55.92	152	56	2	1	423	1	152	1.00E-53	169
TcMYB58	AtMYB83	70.87	103	30	0	40	348	32	134	3.00E-53	169
TcMYB58	ATMYB87	49.4	168	81	3	1	492	1	165	7.00E-53	166
TcMYB58	ATMYB95	54.36	149	65	2	1	438	1	147	2.00E-52	164
TcMYB58	ATMYB26	47.67	172	81	3	1	489	1	162	2.00E-52	167
TcMYB58	ATMYB37	58.73	126	51	1	1	375	1	126	3.00E-52	166
TcMYB58	ATMYB96	65.52	116	40	0	1	348	1	116	7.00E-52	165
TcMYB58	ATMYB84	58.33	120	49	1	1	357	1	120	3.00E-51	162
TcMYB58	ATMYB46	66.99	103	34	0	40	348	20	122	7.00E-51	160
TcMYB58	ATMYB68	58.33	120	49	1	1	357	1	120	1.00E-50	162
TcMYB58	AtMYB36	59.17	120	48	1	1	357	1	120	1.00E-50	161
TcMYB58	ATMYB0	60.91	110	43	0	22	351	10	119	2.00E-50	158
TcMYB58	ATMYB23	63.11	103	38	0	40	348	14	116	3.00E-50	157
TcMYB58	AtMYB47	47.56	164	81	2	1	477	1	163	1.00E-49	157
TcMYB58	AtMYB114	65.69	102	35	0	40	345	10	111	2.00E-49	152
TcMYB58	ATMYB38	56.3	119	51	1	1	354	1	119	2.00E-49	157
TcMYB58	AtMYB82	63.21	106	39	0	40	357	14	119	1.00E-48	152
TcMYB58	ATMYB90	60.53	114	45	0	40	381	10	123	2.00E-47	150
TcMYB58	ATMYB75	64.71	102	36	0	40	345	10	111	3.00E-47	150
TcMYB58	ATMYB71	64.76	105	37	0	40	354	20	124	5.00E-47	150
TcMYB58	AtMYB19	64.15	106	38	0	34	351	12	117	1.00E-46	149
TcMYB58	ATMYB57	59.63	109	44	0	22	348	21	129	2.00E-46	147
TcMYB58	AtMYB79	51.77	141	68	1	40	462	8	142	2.00E-46	148
TcMYB58	ATMYB121	45.22	157	86	1	40	510	29	179	5.00E-46	148
TcMYB58	AtMYB113	42.02	188	98	2	40	570	10	197	4.00E-45	145
TcMYB58	AtMYB24	57.94	107	45	0	40	360	19	125	8.00E-45	143
TcMYB58	AtMYB18	63.3	109	39	1	28	351	7	115	1.00E-44	144
TcMYB58	ATMYB101	61.76	102	39	0	40	345	20	121	2.00E-44	149
TcMYB58	AtMYB112	56.8	125	52	2	40	408	34	155	3.00E-44	142
TcMYB58	AtMYB81	61.39	101	39	0	40	342	22	122	5.00E-44	146
TcMYB58	ATMYB3	59.41	101	41	0	40	342	22	122	9.00E-44	140
TcMYB58	AtMYB27	64.71	102	36	0	40	345	11	112	1.00E-43	140
TcMYB58	ATMYB120	63.11	103	38	0	40	348	28	130	2.00E-43	147
TcMYB58	AtMYB45	48.09	131	68	0	34	426	18	148	2.00E-43	141
TcMYB58	ATMYB2	35.56	239	142	5	22	702	16	243	2.00E-43	141
TcMYB58	AtMYB108	58.88	107	44	0	22	342	15	121	7.00E-43	141
TcMYB58	AtMYB97	60.4	101	40	0	40	342	21	121	1.00E-42	142
TcMYB58	ATMYB65	55.75	113	47	1	16	345	32	144	2.00E-42	144
TcMYB58	ATMYB33	57.84	102	43	0	40	345	34	135	2.00E-42	144
TcMYB58	AtMYB116	49.17	120	61	0	22	381	14	133	9.00E-42	137
TcMYB58	ATMYB48	42.95	156	84	3	22	474	4	152	1.00E-40	134
TcMYB58	AtMYB62	48.36	122	63	0	22	387	15	136	4.00E-40	133
TcMYB58	ATMYB59-3	54.9	102	46	0	40	345	10	111	2.00E-39	130
TcMYB58	ATMYB78	53.39	118	41	1	31	342	25	142	1.00E-38	130

TcMYB58	AtMYB104	48.51	101	52	0	40	342	18	118	3.00E-32	114
TcMYB58	ATMYB73	46.55	116	62	1	13	360	4	118	5.00E-31	110
TcMYB58	ATMYB73	25	80	59	1	199	435	13	92	8.00E-05	35.4
TcMYB58	ATMYB25	53.92	102	47	1	40	345	50	150	7.00E-31	110
TcMYB58	AtMYB70	49.09	110	56	1	31	360	10	118	1.00E-30	108
TcMYB58	AtMYB70	32.26	62	41	1	190	372	10	71	4.00E-05	36.2
TcMYB58	AtMYB1	50.47	107	53	1	31	351	52	157	1.00E-30	110
TcMYB58	AtMYB1	32.79	61	40	1	184	363	50	110	8.00E-06	38.5
TcMYB58	AtMYB115	34.92	189	123	6	40	606	158	330	2.00E-30	108
TcMYB58	ATMYB44	46.72	122	61	3	31	384	3	123	1.00E-29	106
TcMYB58	AtMYB98	44.35	115	64	1	40	384	217	330	1.00E-29	108
TcMYB58	ATMYB119	35.33	167	99	3	40	513	105	269	2.00E-29	107
TcMYB58	ATMYB77	47.62	105	55	1	31	345	3	106	4.00E-29	104
TcMYB58	AtMYB64	45.63	103	55	2	40	345	105	205	3.00E-28	103
TcMYB58	AtMYB64	25.81	62	45	1	199	381	105	166	1.00E-04	35
TcMYB58	ATMYB118	45.1	102	56	1	40	345	189	289	5.00E-28	103
TcMYB58	ATMYB118	32.14	56	37	1	199	363	189	244	4.00E-05	36.6
TcMYB58	AtMYB109	50.98	102	50	1	40	345	56	156	1.00E-27	102
TcMYB58	AtMYB109	32.26	62	41	1	199	381	56	117	4.00E-04	33.5
TcMYB58	ATMYB54	41.35	104	60	2	37	345	5	106	7.00E-26	94.7
TcMYB58	ATMYB52	38.89	126	77	3	37	414	4	122	2.00E-25	94
TcMYB58	ATMYB105	38.79	116	70	2	37	381	106	219	5.00E-25	94
TcMYB58	AtMYB117	42.72	103	58	2	40	345	98	198	3.00E-24	92.4
TcMYB58	AtMYB100	39.09	110	67	1	31	360	23	131	2.00E-23	88.2
TcMYB58	ATMYB69	33.33	132	88	2	40	435	19	148	6.00E-23	87
TcMYB58	ATMYB110	38.46	104	64	1	37	348	66	168	1.00E-22	87.4
TcMYB58	AtMYB56	39.81	103	62	1	37	345	92	193	3.00E-22	86.3
TcMYB58	AtMYB89	38	100	62	1	43	342	57	155	4.00E-20	78.2
TcMYB58	AtMYB22	34.62	104	68	1	31	342	51	152	2.00E-19	77.4
TcMYB58	AtMYB22	25	52	39	0	184	339	49	100	2.00E-04	33.9
TcMYB58	ATMYB91	36.36	99	61	1	34	324	2	100	2.00E-18	75.9
TcMYB58	AtMYB124	31.45	124	81	2	31	390	22	144	8.00E-17	71.6
TcMYB58	ATMYB88	34.29	105	69	1	31	345	27	130	4.00E-16	69.7
TcMYB59	ATMYB5	62.71	118	44	0	10	363	22	139	3.00E-55	171
TcMYB59	ATMYB123	70.37	108	32	0	4	327	11	118	5.00E-55	170
TcMYB59	AtMYB74	53.9	141	65	1	4	426	10	148	1.00E-53	169
TcMYB59	ATMYB102	53.42	146	68	0	4	441	9	154	5.00E-53	168
TcMYB59	AtMYB82	51.33	150	71	2	13	456	12	157	1.00E-51	160
TcMYB59	AtMYB17	57.25	131	53	1	10	393	11	141	2.00E-51	162
TcMYB59	AtMYB41	50.34	149	64	1	4	420	9	157	3.00E-51	161
TcMYB59	ATMYB66	53.52	142	65	1	19	441	18	159	1.00E-50	157
TcMYB59	ATMYB111	55.15	136	55	1	10	399	11	146	2.00E-50	161
TcMYB59	ATMYB23	56.3	135	59	1	19	423	14	146	2.00E-50	157
TcMYB59	ATMYB3	51.06	141	69	1	13	435	12	150	3.00E-50	158
TcMYB59	AtMYB53	57.98	119	50	0	10	366	11	129	3.00E-50	159
TcMYB59	ATMYB16	56.59	129	56	1	10	396	11	136	7.00E-50	159
TcMYB59	AtMYB49	47.74	155	79	1	13	471	12	166	8.00E-50	159
TcMYB59	ATMYB92	53.42	146	64	2	10	435	11	151	8.00E-50	159
TcMYB59	MYB7	61.26	111	43	0	13	345	12	122	2.00E-49	156
TcMYB59	ATMYB30	48.57	140	72	0	4	423	9	148	2.00E-49	158
TcMYB59	ATMYB4	48.1	158	82	3	16	489	13	159	7.00E-49	155
TcMYB59	AtMYB114	61.9	105	40	0	10	324	7	111	7.00E-49	150
TcMYB59	AtMYB113	49.37	158	74	4	10	465	7	158	7.00E-49	154
TcMYB59	ATMYB90	57.72	123	52	1	10	378	7	124	8.00E-49	154
TcMYB59	ATMYB106	41.92	198	114	3	4	594	52	235	1.00E-48	157
TcMYB59	AtMYB107	56.3	119	52	0	10	366	11	129	1.00E-48	155
TcMYB59	AtMYB6	48.95	143	73	0	16	444	13	155	2.00E-48	153
TcMYB59	AtMYB32	53.54	127	59	0	16	396	13	139	2.00E-48	154
TcMYB59	ATMYB86	47.03	185	98	2	13	567	12	191	2.00E-48	156
TcMYB59	ATMYB0	39.44	213	126	2	19	648	16	227	3.00E-48	152
TcMYB59	ATMYB13	53.57	140	64	2	10	426	11	147	4.00E-48	152
TcMYB59	ATMYB15	51.06	141	66	2	10	423	11	147	6.00E-48	153
TcMYB59	ATMYB75	48.1	158	77	4	10	468	7	158	6.00E-48	152
TcMYB59	ATMYB12	61.32	106	41	0	10	327	11	116	2.00E-47	154
TcMYB59	AtMYB9	56.3	119	52	0	10	366	11	129	6.00E-47	152
TcMYB59	ATMYB14	59.81	107	43	0	10	330	11	117	7.00E-47	149
TcMYB59	ATMYB46	36.59	246	142	6	13	708	18	250	1.00E-46	149
TcMYB59	AtMYB50	50	144	72	0	13	444	12	155	1.00E-46	150
TcMYB59	ATMYB58	47.65	149	75	2	13	450	14	159	1.00E-46	149
TcMYB59	AtMYB93	55.46	119	53	0	10	366	11	129	2.00E-46	151
TcMYB59	AtMYB60	44.97	149	79	1	10	447	11	159	2.00E-46	149
TcMYB59	AtMYB51	59.82	112	45	0	10	345	12	123	2.00E-46	151
TcMYB59	ATMYB95	49.32	146	68	1	10	429	11	156	3.00E-46	148
TcMYB59	ATMYB72	41.33	196	111	3	13	588	14	207	3.00E-46	149
TcMYB59	ATMYB34	55.08	118	53	0	10	363	11	128	5.00E-46	148
TcMYB59	MYB8	49.19	124	63	0	13	384	12	135	6.00E-46	145
TcMYB59	ATMYB122	36.64	232	146	4	10	702	11	233	2.00E-45	148
TcMYB59	ATMYB63	47.3	148	78	1	13	456	14	156	3.00E-45	146
TcMYB59	ATMYB84	55.36	112	49	1	4	336	9	120	4.00E-45	146
TcMYB59	ATMYB94	56.6	106	46	0	10	327	11	116	5.00E-45	147

TcMYB59	ATMYB87	47.68	151	75	4	13	453	12	158	7.00E-45	145
TcMYB59	AtMYB103	45.89	146	74	1	13	435	12	157	7.00E-45	147
TcMYB59	AtMYB85	40.91	198	114	4	10	594	11	200	1.00E-44	144
TcMYB59	AtMYB42	56.41	117	51	0	13	363	12	128	1.00E-44	144
TcMYB59	ATMYB80	54.7	117	53	0	13	363	12	128	1.00E-44	145
TcMYB59	ATMYB96	56.6	106	46	0	10	327	11	116	1.00E-44	146
TcMYB59	ATMYB61	51.13	133	60	1	13	396	12	144	2.00E-44	146
TcMYB59	ATMYB35	43.64	165	78	1	4	453	9	173	2.00E-44	144
TcMYB59	ATMYB11	56.6	106	46	0	10	327	11	116	3.00E-44	145
TcMYB59	ATMYB68	54.46	112	50	1	4	336	9	120	6.00E-44	145
TcMYB59	AtMYB83	48.99	149	76	2	13	459	30	172	6.00E-44	144
TcMYB59	ATMYB37	56.25	112	48	1	13	345	12	123	9.00E-44	143
TcMYB59	ATMYB31	56.19	105	46	0	13	327	12	116	1.00E-43	143
TcMYB59	AtMYB20	44.52	155	83	1	10	465	11	165	2.00E-43	141
TcMYB59	AtMYB43	40.35	228	129	6	10	672	11	222	2.00E-43	142
TcMYB59	AtMYB36	53.57	112	51	1	4	336	9	120	3.00E-43	142
TcMYB59	ATMYB29	45.75	153	83	2	10	468	11	158	3.00E-43	142
TcMYB59	AtMYB10	47.06	136	72	1	13	420	14	147	3.00E-43	139
TcMYB59	ATMYB28	45.58	147	72	1	10	426	11	157	4.00E-43	142
TcMYB59	ATMYB38	44.1	161	84	4	4	468	9	165	5.00E-43	140
TcMYB59	ATMYB121	44.1	161	77	2	10	453	26	185	5.00E-43	140
TcMYB59	AtMYB76	50	126	63	0	10	387	11	136	1.00E-42	140
TcMYB59	ATMYB71	56.48	108	47	0	10	333	17	124	2.00E-42	138
TcMYB59	ATMYB67	49.22	128	65	0	13	396	22	149	2.00E-42	139
TcMYB59	AtMYB47	49.59	121	61	0	10	372	11	131	7.00E-42	137
TcMYB59	ATMYB59-3	34.29	210	134	3	19	636	10	212	1.00E-41	135
TcMYB59	AtMYB112	55.34	103	46	0	13	321	32	134	1.00E-41	135
TcMYB59	AtMYB79	36.41	206	116	4	19	591	8	213	2.00E-41	135
TcMYB59	ATMYB57	43.36	143	81	1	7	435	23	163	3.00E-41	133
TcMYB59	ATMYB48	46.62	133	71	1	10	408	6	135	4.00E-41	134
TcMYB59	ATMYB55	54.47	123	44	1	13	345	12	134	6.00E-41	136
TcMYB59	AtMYB19	39.49	195	113	5	19	588	14	196	8.00E-41	134
TcMYB59	ATMYB26	54.62	119	45	1	13	342	12	130	2.00E-40	135
TcMYB59	AtMYB45	32.62	233	154	3	19	708	20	233	2.00E-40	132
TcMYB59	ATMYB120	55.24	105	47	0	13	327	26	130	3.00E-40	137
TcMYB59	AtMYB97	34.19	234	135	4	13	657	19	252	4.00E-40	135
TcMYB59	AtMYB18	37.92	240	139	8	19	708	12	219	4.00E-40	132
TcMYB59	AtMYB27	53.64	110	51	0	13	342	9	118	5.00E-40	131
TcMYB59	ATMYB101	40.13	152	91	1	10	465	17	165	6.00E-40	136
TcMYB59	AtMYB108	51.79	112	54	0	13	348	19	130	1.00E-39	132
TcMYB59	ATMYB3	50.46	109	54	0	13	339	20	128	2.00E-39	129
TcMYB59	AtMYB116	53.92	102	47	0	19	324	20	121	2.00E-39	130
TcMYB59	AtMYB81	39.1	156	95	1	1	468	16	170	3.00E-39	134
TcMYB59	AtMYB62	50	118	59	1	13	366	19	134	3.00E-39	130
TcMYB59	ATMYB99	53.1	113	46	1	10	327	12	124	4.00E-39	129
TcMYB59	AtMYB40	54.72	106	48	0	10	327	11	116	6.00E-39	129
TcMYB59	ATMYB2	46.09	115	62	0	13	357	20	134	1.00E-38	129
TcMYB59	AtMYB24	51.43	105	51	0	13	327	17	121	2.00E-38	126
TcMYB59	ATMYB33	39.75	161	86	3	13	462	32	189	2.00E-37	130
TcMYB59	ATMYB65	47.66	107	56	0	4	324	38	144	4.00E-36	127
TcMYB59	ATMYB78	29.76	289	141	6	13	693	26	314	1.00E-33	117
TcMYB59	AtMYB104	33.97	156	103	1	1	468	12	166	3.00E-32	114
TcMYB59	ATMYB54	39.19	148	88	3	16	453	5	145	3.00E-30	105
TcMYB59	AtMYB115	33.97	156	103	2	13	480	156	307	5.00E-28	102
TcMYB59	AtMYB117	43.27	104	59	1	13	324	96	198	4.00E-27	100
TcMYB59	ATMYB118	42.06	107	62	1	4	324	184	289	4.00E-27	100
TcMYB59	ATMYB118	28.17	71	46	2	130	327	169	239	1.00E-04	35
TcMYB59	ATMYB73	45.1	102	56	1	19	324	13	113	8.00E-27	98.6
TcMYB59	ATMYB105	41.12	107	63	1	4	324	102	207	9.00E-27	98.6
TcMYB59	ATMYB44	43.52	108	61	1	19	342	6	112	1.00E-26	97.8
TcMYB59	ATMYB25	44.34	106	59	1	19	336	50	154	1.00E-26	98.6
TcMYB59	AtMYB70	44.12	102	57	1	19	324	13	113	3.00E-26	97.1
TcMYB59	AtMYB98	37.23	137	86	2	13	423	215	349	4.00E-26	98.2
TcMYB59	ATMYB52	42.72	103	59	1	16	324	4	105	5.00E-26	95.1
TcMYB59	ATMYB119	35.43	127	82	1	13	393	103	228	2.00E-25	95.9
TcMYB59	AtMYB1	40.17	117	70	1	19	369	55	170	3.00E-25	95.5
TcMYB59	AtMYB56	37.31	134	82	2	16	411	92	224	1.00E-24	92.8
TcMYB59	AtMYB109	44.23	104	58	1	19	330	56	158	1.00E-24	93.6
TcMYB59	AtMYB100	37.74	106	66	1	4	321	21	125	1.00E-24	90.9
TcMYB59	AtMYB64	33.11	148	99	2	19	462	105	249	2.00E-24	93.2
TcMYB59	ATMYB69	34.31	137	90	1	19	429	19	154	7.00E-23	86.7
TcMYB59	ATMYB77	41.18	102	60	1	19	324	6	106	8.00E-23	87.4
TcMYB59	ATMYB91	40.38	104	60	1	19	324	4	107	1.00E-22	87.8
TcMYB59	ATMYB110	36.54	104	66	1	16	327	66	168	2.00E-21	83.6
TcMYB59	ATMYB88	38.38	99	61	1	28	324	33	130	3.00E-19	78.6
TcMYB59	AtMYB22	34.91	106	69	2	4	321	49	152	4.00E-19	76.6
TcMYB59	AtMYB89	40.74	108	60	2	22	333	57	163	6.00E-19	74.7
TcMYB59	AtMYB124	32.33	133	77	3	28	387	28	159	1.00E-18	77
TcMYB60	ATMYB61	72	175	39	3	1	495	1	175	1.00E-86	263
TcMYB60	ATMYB55	68.65	185	33	2	1	480	1	185	2.00E-84	256

TcMYB60	AtMYB50	76.82	151	35	1	1	453	1	149	3.00E-84	255
TcMYB60	ATMYB86	85.27	129	19	0	1	387	1	129	5.00E-81	248
TcMYB60	ATMYB67	55.93	177	78	2	10	540	14	177	5.00E-67	210
TcMYB60	AtMYB32	62.24	143	54	1	1	429	1	141	7.00E-64	201
TcMYB60	ATMYB26	73.6	125	24	1	1	348	1	125	2.00E-63	203
TcMYB60	AtMYB103	34.69	392	232	11	1	1104	1	361	2.00E-63	203
TcMYB60	AtMYB107	69.53	128	39	0	1	384	1	128	3.00E-63	201
TcMYB60	AtMYB17	68.99	129	40	0	1	387	1	129	6.00E-62	197
TcMYB60	AtMYB9	67.97	128	41	0	1	384	1	128	2.00E-61	197
TcMYB60	MYB7	60.56	142	56	0	1	426	1	142	5.00E-61	193
TcMYB60	ATMYB4	55.9	161	62	1	1	456	1	161	1.00E-60	193
TcMYB60	ATMYB16	67.18	131	43	0	1	393	1	131	1.00E-60	194
TcMYB60	ATMYB3	64.12	131	47	0	1	393	1	131	1.00E-59	189
TcMYB60	AtMYB43	67.18	131	43	0	1	393	1	131	1.00E-59	191
TcMYB60	AtMYB93	41.63	257	146	4	1	759	1	228	2.00E-59	192
TcMYB60	AtMYB83	51.74	172	83	0	25	540	27	198	2.00E-59	191
TcMYB60	AtMYB6	63.36	131	48	0	1	393	1	131	3.00E-59	187
TcMYB60	AtMYB20	63.04	138	51	0	1	414	1	138	3.00E-59	189
TcMYB60	AtMYB85	43.4	235	126	4	1	684	1	220	5.00E-59	188
TcMYB60	ATMYB34	52.69	167	77	2	1	495	1	166	9.00E-59	188
TcMYB60	ATMYB35	55.28	161	68	1	1	471	1	161	2.00E-58	188
TcMYB60	ATMYB106	69.67	122	37	0	16	381	49	170	2.00E-58	190
TcMYB60	ATMYB92	35.13	316	180	5	1	873	1	316	3.00E-58	188
TcMYB60	AtMYB42	50.27	187	93	2	1	561	1	171	4.00E-58	186
TcMYB60	ATMYB102	63.64	132	48	0	1	396	1	132	4.00E-58	188
TcMYB60	MYB8	61.83	131	50	0	1	393	1	131	6.00E-58	183
TcMYB60	ATMYB80	48.35	182	94	1	1	546	1	179	7.00E-58	187
TcMYB60	AtMYB51	37.5	312	189	9	1	918	1	276	1.00E-57	187
TcMYB60	AtMYB74	62.41	133	49	1	1	396	1	133	2.00E-57	186
TcMYB60	ATMYB95	56.58	152	65	1	1	453	1	152	2.00E-56	181
TcMYB60	ATMYB46	72.48	109	30	0	28	354	16	124	3.00E-56	181
TcMYB60	AtMYB53	63.28	128	47	0	1	384	1	128	3.00E-56	182
TcMYB60	AtMYB40	59.72	144	58	1	1	432	1	143	3.00E-56	181
TcMYB60	ATMYB28	32.25	369	226	10	1	1035	1	358	9.00E-56	182
TcMYB60	AtMYB41	60.61	132	52	0	1	396	1	132	1.00E-55	180
TcMYB60	AtMYB49	64.84	128	45	0	1	384	1	128	1.00E-55	181
TcMYB60	ATMYB29	44.44	207	115	2	1	621	1	196	2.00E-55	181
TcMYB60	ATMYB122	48.85	174	73	1	1	474	1	174	3.00E-54	177
TcMYB60	ATMYB5	53.42	161	75	1	16	498	17	173	3.00E-54	175
TcMYB60	ATMYB13	48.3	176	87	1	1	516	1	176	6.00E-54	174
TcMYB60	AtMYB47	39.56	225	129	3	1	654	1	222	2.00E-53	174
TcMYB60	ATMYB14	55.03	149	67	0	1	447	1	149	4.00E-53	172
TcMYB60	AtMYB45	54.36	149	68	0	25	471	15	163	4.00E-53	172
TcMYB60	ATMYB63	47.09	189	99	3	4	567	4	180	9.00E-53	172
TcMYB60	AtMYB76	45.79	190	96	1	1	549	1	190	4.00E-52	172
TcMYB60	ATMYB15	37.2	250	116	5	1	627	1	248	5.00E-52	170
TcMYB60	ATMYB99	60.9	133	45	1	4	381	3	135	2.00E-51	167
TcMYB60	ATMYB72	40.83	240	120	4	4	657	4	241	2.00E-51	169
TcMYB60	ATMYB58	42.93	205	117	2	4	618	4	204	2.00E-50	166
TcMYB60	ATMYB111	63.79	116	42	0	1	348	1	116	9.00E-49	163
TcMYB60	AtMYB19	42.22	225	99	4	25	606	9	233	1.00E-48	160
TcMYB60	AtMYB10	60.5	119	47	0	7	363	5	123	3.00E-48	159
TcMYB60	ATMYB12	62.07	116	44	0	1	348	1	116	5.00E-48	162
TcMYB60	AtMYB60	59.02	122	50	0	1	366	1	122	8.00E-48	159
TcMYB60	ATMYB11	61.21	116	45	0	1	348	1	116	1.00E-47	160
TcMYB60	AtMYB36	47.65	170	80	2	1	483	1	170	2.00E-47	160
TcMYB60	AtMYB18	67.59	108	35	0	22	345	6	113	3.00E-47	157
TcMYB60	ATMYB94	59.32	118	48	0	1	354	1	118	2.00E-46	157
TcMYB60	ATMYB96	59.32	118	48	0	1	354	1	118	5.00E-46	156
TcMYB60	ATMYB37	56.91	123	52	1	1	366	1	123	2.00E-45	154
TcMYB60	ATMYB30	48.41	157	81	2	1	471	1	143	2.00E-45	154
TcMYB60	ATMYB31	49.04	157	80	2	1	471	1	142	3.00E-45	154
TcMYB60	ATMYB84	58.97	117	47	1	1	348	1	117	1.00E-44	151
TcMYB60	ATMYB123	62.96	108	40	0	25	348	11	118	2.00E-44	149
TcMYB60	AtMYB79	63.81	105	38	0	37	351	7	111	2.00E-44	149
TcMYB60	AtMYB82	48.65	148	62	2	25	426	9	156	3.00E-44	147
TcMYB60	ATMYB121	42.78	180	103	1	25	564	24	198	8.00E-44	148
TcMYB60	ATMYB38	55.12	127	56	1	1	378	1	127	1.00E-43	149
TcMYB60	ATMYB3	47.89	142	74	1	31	456	19	155	1.00E-43	146
TcMYB60	ATMYB68	58.12	117	48	1	1	348	1	117	1.00E-43	150
TcMYB60	ATMYB66	59.62	104	42	0	37	348	17	120	1.00E-43	145
TcMYB60	ATMYB71	62.86	105	39	0	37	351	19	123	2.00E-43	147
TcMYB60	ATMYB87	57.26	117	49	1	1	348	1	117	4.00E-43	147
TcMYB60	ATMYB75	42.93	184	90	4	34	540	8	183	5.00E-43	145
TcMYB60	ATMYB23	56.48	108	47	0	25	348	9	116	6.00E-43	144
TcMYB60	AtMYB116	55.65	115	51	0	37	381	19	133	4.00E-42	144
TcMYB60	AtMYB97	59.81	107	43	0	34	354	19	125	4.00E-42	147
TcMYB60	ATMYB0	56.07	107	47	0	28	348	12	118	4.00E-42	142
TcMYB60	AtMYB24	57.27	110	47	0	31	360	16	125	6.00E-42	141
TcMYB60	ATMYB57	54.31	116	53	0	34	381	25	140	9.00E-42	140

TcMYB60	ATMYB101	60.38	106	42	0	34	351	18	123	1.00E-41	147
TcMYB60	AtMYB114	59.62	104	42	0	34	345	8	111	1.00E-41	138
TcMYB60	AtMYB81	49.24	132	56	1	25	387	17	148	1.00E-41	146
TcMYB60	AtMYB62	55.08	118	53	0	28	381	17	134	4.00E-41	141
TcMYB60	ATMYB120	61.68	107	41	0	34	354	26	132	5.00E-41	146
TcMYB60	ATMYB33	58.49	106	44	0	34	351	32	137	8.00E-40	143
TcMYB60	ATMYB90	50.77	130	55	1	34	396	8	137	2.00E-39	136
TcMYB60	ATMYB59-3	54.05	111	51	0	25	357	5	115	3.00E-39	135
TcMYB60	ATMYB65	39.23	209	126	4	34	657	41	241	3.00E-39	142
TcMYB60	AtMYB108	55.86	111	49	0	34	366	19	129	3.00E-39	137
TcMYB60	AtMYB112	54.81	104	47	0	31	342	31	134	9.00E-39	134
TcMYB60	AtMYB113	56.48	108	47	0	34	357	8	115	1.00E-38	134
TcMYB60	ATMYB48	41.83	153	89	1	37	495	8	154	1.00E-38	134
TcMYB60	AtMYB27	48.09	131	68	1	25	417	6	131	4.00E-37	129
TcMYB60	ATMYB2	50.45	111	55	0	34	366	20	130	7.00E-37	129
TcMYB60	AtMYB104	49.11	112	57	1	25	360	13	121	1.00E-34	126
TcMYB60	ATMYB78	37.56	197	96	5	34	543	26	211	3.00E-34	124
TcMYB60	ATMYB119	30.51	236	157	6	28	714	101	318	6.00E-29	110
TcMYB60	ATMYB44	40.27	149	84	4	40	471	6	153	1.00E-28	107
TcMYB60	ATMYB77	50	102	51	1	40	345	6	106	2.00E-28	107
TcMYB60	ATMYB73	41.43	140	82	2	40	459	13	150	3.00E-28	107
TcMYB60	AtMYB70	50.98	102	50	1	40	345	13	113	1.00E-27	105
TcMYB60	AtMYB98	44.14	111	62	1	25	357	212	321	2.00E-27	106
TcMYB60	ATMYB118	43.33	120	65	3	25	375	184	301	5.00E-27	105
TcMYB60	ATMYB25	51.46	103	49	2	40	345	50	150	7.00E-27	103
TcMYB60	AtMYB1	50	102	51	1	40	345	55	155	2.00E-26	103
TcMYB60	AtMYB64	33.77	154	100	3	40	495	105	250	3.00E-26	102
TcMYB60	ATMYB105	42.86	112	63	2	25	357	102	211	7.00E-26	100
TcMYB60	AtMYB115	42.16	102	59	1	25	330	153	253	8.00E-26	100
TcMYB60	ATMYB54	46.23	106	56	2	40	354	6	109	9.00E-26	98.2
TcMYB60	ATMYB52	43.4	106	60	1	40	357	5	109	1.00E-25	98.2
TcMYB60	AtMYB109	50.49	103	50	2	40	345	56	156	1.00E-25	100
TcMYB60	AtMYB117	44.44	108	59	2	34	354	96	201	5.00E-25	98.6
TcMYB60	ATMYB110	33.99	153	101	3	40	498	67	208	2.00E-24	95.9
TcMYB60	ATMYB88	30.43	253	143	8	10	669	19	256	4.00E-24	97.4
TcMYB60	AtMYB100	35.03	157	100	3	25	489	21	174	5.00E-24	93.2
TcMYB60	ATMYB69	38.18	110	68	1	25	354	14	122	2.00E-23	91.7
TcMYB60	AtMYB56	39.05	105	64	1	40	354	93	196	2.00E-22	90.5
TcMYB60	AtMYB89	34.48	116	76	1	25	372	51	165	4.00E-20	80.9
TcMYB60	AtMYB124	37.14	105	66	1	49	363	28	131	1.00E-19	83.6
TcMYB60	ATMYB91	38.46	104	62	1	49	354	7	110	2.00E-19	82
TcMYB60	AtMYB22	34.82	112	73	2	25	360	49	158	1.00E-18	78.2
TcMYB61	ATMYB5	75.2	125	31	0	13	387	16	140	9.00E-73	221
TcMYB61	MYB7	57.58	165	66	2	1	483	1	165	3.00E-68	210
TcMYB61	AtMYB32	60	150	60	0	1	450	1	150	9.00E-67	206
TcMYB61	AtMYB17	67.94	131	42	0	1	393	1	131	1.00E-66	207
TcMYB61	ATMYB4	63.04	138	51	0	1	414	1	138	3.00E-66	205
TcMYB61	ATMYB111	61.01	159	58	2	1	465	1	159	3.00E-66	207
TcMYB61	ATMYB3	60.69	145	57	1	1	435	1	142	1.00E-65	203
TcMYB61	AtMYB6	63.91	133	48	0	1	399	1	133	4.00E-65	201
TcMYB61	MYB8	64.29	126	45	0	1	378	1	126	3.00E-64	198
TcMYB61	AtMYB42	58.24	170	71	1	1	510	1	165	9.00E-64	199
TcMYB61	AtMYB43	43.43	251	140	3	1	747	1	243	1.00E-63	200
TcMYB61	AtMYB41	43.03	251	143	4	1	753	1	239	1.00E-63	199
TcMYB61	AtMYB107	67.19	128	42	0	1	384	1	128	2.00E-63	199
TcMYB61	AtMYB85	69.77	129	39	0	1	387	1	129	2.00E-63	197
TcMYB61	AtMYB20	57.83	166	67	2	1	489	1	166	4.00E-63	197
TcMYB61	ATMYB16	52.66	188	76	2	1	525	1	185	8.00E-63	198
TcMYB61	ATMYB12	74.14	116	30	0	1	348	1	116	1.00E-62	199
TcMYB61	AtMYB9	37.61	351	216	8	1	1044	1	333	1.00E-62	198
TcMYB61	ATMYB102	67.97	128	41	0	1	384	1	128	2.00E-62	198
TcMYB61	ATMYB106	41.88	277	145	6	10	792	47	315	6.00E-62	197
TcMYB61	ATMYB15	57.24	152	53	1	1	420	1	152	6.00E-61	192
TcMYB61	AtMYB74	41.57	255	147	5	1	759	1	225	2.00E-60	191
TcMYB61	ATMYB11	53.67	177	76	3	1	513	1	172	6.00E-60	191
TcMYB61	AtMYB93	65.62	128	44	0	1	384	1	128	7.00E-60	191
TcMYB61	ATMYB14	44.05	227	113	3	1	639	1	219	2.00E-59	186
TcMYB61	ATMYB13	61.87	139	53	1	1	417	1	131	9.00E-59	185
TcMYB61	ATMYB86	39.74	302	172	6	1	876	1	291	2.00E-58	187
TcMYB61	ATMYB29	43.97	232	128	3	1	690	1	220	2.00E-58	187
TcMYB61	AtMYB49	42.46	252	136	4	1	729	1	224	4.00E-58	186
TcMYB61	ATMYB92	63.28	128	47	0	1	384	1	128	5.00E-58	186
TcMYB61	AtMYB53	63.28	128	47	0	1	384	1	128	5.00E-58	185
TcMYB61	AtMYB40	40.7	258	147	6	1	756	1	235	2.00E-57	182
TcMYB61	AtMYB51	49.72	181	78	3	1	504	1	181	2.00E-57	184
TcMYB61	ATMYB72	37.87	301	177	8	4	876	4	289	2.00E-56	181
TcMYB61	ATMYB122	38.83	273	150	5	1	768	1	270	3.00E-56	181
TcMYB61	ATMYB28	54.84	155	65	1	1	450	1	155	5.00E-56	181
TcMYB61	ATMYB63	51.57	159	76	1	4	477	4	162	8.00E-56	179
TcMYB61	ATMYB58	66.09	115	39	0	4	348	4	118	1.00E-55	177

TcMYB61	ATMYB123	62.41	141	53	3	34	456	14	145	2.00E-55	176
TcMYB61	ATMYB99	50	184	72	4	4	495	3	186	6.00E-55	175
TcMYB61	AtMYB50	52.47	162	69	2	1	462	1	162	1.00E-54	176
TcMYB61	ATMYB80	59.38	128	52	0	1	384	1	128	2.00E-54	176
TcMYB61	ATMYB34	58.91	129	53	0	1	387	1	129	2.00E-54	175
TcMYB61	AtMYB76	39.15	258	143	5	1	732	1	248	8.00E-54	175
TcMYB61	ATMYB61	34.99	363	210	13	1	1011	1	338	1.00E-53	175
TcMYB61	ATMYB31	39.67	242	138	4	1	702	1	238	1.00E-53	174
TcMYB61	ATMYB35	57.36	129	55	0	1	387	1	129	2.00E-53	173
TcMYB61	AtMYB10	48.17	164	81	1	7	486	5	168	2.00E-53	171
TcMYB61	ATMYB67	37.61	226	141	3	16	693	16	228	4.00E-52	169
TcMYB61	ATMYB30	58.14	129	54	1	1	387	1	127	4.00E-52	170
TcMYB61	AtMYB60	61.21	116	45	0	1	348	1	116	5.00E-52	168
TcMYB61	ATMYB94	38.14	236	141	4	1	693	1	230	6.00E-52	170
TcMYB61	AtMYB114	67.59	108	35	0	22	345	4	111	2.00E-51	162
TcMYB61	ATMYB96	39.75	239	144	5	1	717	1	224	3.00E-51	168
TcMYB61	AtMYB36	37.56	221	133	2	1	648	1	221	6.00E-51	167
TcMYB61	ATMYB66	57.04	135	49	2	40	417	18	150	1.00E-50	162
TcMYB61	ATMYB95	57.81	128	54	0	1	384	1	128	1.00E-50	164
TcMYB61	ATMYB75	52.17	161	73	3	22	492	4	157	1.00E-50	164
TcMYB61	AtMYB113	48.65	185	93	5	1	549	1	176	2.00E-50	163
TcMYB61	ATMYB90	66.67	108	36	0	22	345	4	111	5.00E-50	162
TcMYB61	ATMYB84	59.17	120	48	1	1	357	1	120	2.00E-49	162
TcMYB61	ATMYB55	56.74	141	49	1	1	387	1	141	3.00E-49	163
TcMYB61	ATMYB23	64.08	103	37	0	40	348	14	116	5.00E-49	159
TcMYB61	AtMYB82	65.69	102	35	0	40	345	14	115	5.00E-49	158
TcMYB61	ATMYB87	35.08	248	156	4	1	729	1	238	9.00E-49	160
TcMYB61	ATMYB0	46.5	157	84	0	40	510	16	172	3.00E-48	157
TcMYB61	AtMYB47	54.26	129	59	0	1	387	1	129	4.00E-48	158
TcMYB61	ATMYB68	58.33	120	49	1	1	357	1	120	7.00E-48	160
TcMYB61	ATMYB38	55.12	127	56	1	1	378	1	127	6.00E-47	155
TcMYB61	AtMYB103	54.69	128	58	0	1	384	1	128	9.00E-47	157
TcMYB61	ATMYB37	58.47	118	48	1	1	351	1	118	2.00E-46	155
TcMYB61	ATMYB101	37.93	232	138	4	31	708	17	245	3.00E-46	158
TcMYB61	AtMYB83	54.69	128	58	0	22	405	26	153	5.00E-45	152
TcMYB61	ATMYB120	30.06	346	235	7	34	1050	26	333	1.00E-44	154
TcMYB61	ATMYB57	43.1	174	90	3	34	528	25	198	2.00E-44	146
TcMYB61	AtMYB24	49.25	134	68	0	34	435	17	150	3.00E-44	146
TcMYB61	AtMYB112	55.93	118	50	1	25	372	29	146	6.00E-44	146
TcMYB61	AtMYB79	61.76	102	39	0	40	345	8	109	1.00E-43	146
TcMYB61	ATMYB46	60.95	105	41	0	34	348	18	122	1.00E-43	146
TcMYB61	ATMYB71	60	105	42	0	31	345	17	121	2.00E-43	145
TcMYB61	AtMYB116	55.26	114	51	0	40	381	20	133	3.00E-43	145
TcMYB61	ATMYB26	53.97	126	49	1	1	351	1	126	6.00E-43	147
TcMYB61	ATMYB3	50.83	120	59	0	4	363	10	129	8.00E-43	142
TcMYB61	AtMYB108	60.38	106	42	0	25	342	16	121	1.00E-42	145
TcMYB61	ATMYB121	51.61	124	60	0	31	402	26	149	2.00E-42	143
TcMYB61	AtMYB62	61.54	104	40	0	34	345	19	122	3.00E-42	143
TcMYB61	ATMYB48	46.15	143	75	1	31	453	6	148	1.00E-41	140
TcMYB61	AtMYB81	41.05	190	97	5	25	549	17	200	1.00E-41	145
TcMYB61	AtMYB97	35.83	240	150	4	28	735	17	254	7.00E-41	142
TcMYB61	ATMYB2	53.85	104	48	0	34	345	20	123	1.00E-40	138
TcMYB61	ATMYB59-3	56.6	106	46	0	40	357	10	115	4.00E-40	135
TcMYB61	AtMYB27	58.65	104	43	0	34	345	9	112	4.00E-39	133
TcMYB61	ATMYB33	52.88	104	49	0	34	345	32	135	1.00E-38	138
TcMYB61	ATMYB65	41.67	156	91	2	34	501	41	193	1.00E-38	138
TcMYB61	AtMYB18	56.19	105	46	0	40	354	12	116	1.00E-36	128
TcMYB61	ATMYB78	50	120	46	1	25	342	23	142	1.00E-36	129
TcMYB61	AtMYB19	54.29	105	48	0	40	354	14	118	2.00E-36	127
TcMYB61	AtMYB45	33.64	217	121	4	22	603	14	230	7.00E-35	122
TcMYB61	AtMYB104	44.44	108	60	1	40	363	18	124	1.00E-28	108
TcMYB61	ATMYB119	33.16	190	121	5	22	573	99	279	3.00E-27	105
TcMYB61	ATMYB25	35.67	171	86	4	40	480	50	215	3.00E-27	103
TcMYB61	AtMYB64	37.5	152	87	4	40	471	105	250	5.00E-27	104
TcMYB61	ATMYB73	43.93	107	60	1	40	360	13	118	3.00E-26	100
TcMYB61	ATMYB118	34.78	161	103	4	25	501	184	331	5.00E-26	101
TcMYB61	AtMYB115	44.79	96	53	1	40	327	158	252	5.00E-26	100
TcMYB61	AtMYB115	22.54	173	132	6	193	705	156	308	3.00E-04	34.7
TcMYB61	AtMYB98	41.8	122	69	2	25	384	212	330	8.00E-26	100
TcMYB61	ATMYB54	39.53	129	78	3	40	426	6	131	2.00E-25	96.7
TcMYB61	ATMYB105	35.51	138	89	1	22	435	101	237	2.00E-25	98.2
TcMYB61	ATMYB52	45.1	102	56	1	40	345	5	105	3.00E-25	96.3
TcMYB61	AtMYB109	44.66	103	56	2	40	345	56	156	1.00E-24	97.1
TcMYB61	AtMYB109	38.89	54	32	1	199	357	56	109	1.00E-04	36.2
TcMYB61	AtMYB70	42.16	102	59	1	40	345	13	113	2.00E-24	95.1
TcMYB61	ATMYB77	43.14	102	58	1	40	345	6	106	2.00E-24	94.7
TcMYB61	AtMYB117	43.27	104	59	1	34	345	96	198	3.00E-24	95.5
TcMYB61	ATMYB44	43.14	102	58	1	40	345	6	106	4.00E-24	94.4
TcMYB61	AtMYB1	40.74	108	60	2	40	351	55	157	4.00E-24	95.5
TcMYB61	AtMYB1	30.43	69	47	1	184	387	50	118	6.00E-05	37

TcMYB61	AtMYB100	35.62	146	90	3	49	474	29	173	9.00E-24	91.7
TcMYB61	ATMYB110	29.58	213	137	5	40	639	67	277	6.00E-23	90.9
TcMYB61	AtMYB56	28.57	196	140	4	40	627	93	275	4.00E-22	89
TcMYB61	ATMYB91	25.24	313	212	10	40	912	4	301	6.00E-22	89
TcMYB61	ATMYB69	39.25	107	65	1	25	345	14	119	1.00E-21	85.9
TcMYB61	AtMYB89	39.81	108	64	3	37	357	54	159	2.00E-20	81.3
TcMYB61	AtMYB22	30	130	91	2	49	438	57	182	2.00E-17	74.3
TcMYB61	ATMYB88	37.86	103	64	1	37	345	29	130	4.00E-17	75.1
TcMYB61	AtMYB124	30.19	159	103	4	37	489	24	176	9.00E-17	73.9
TcMYB62	ATMYB54	48.7	230	109	4	79	741	4	223	2.00E-67	205
TcMYB62	ATMYB54	40	50	26	2	862	999	194	243	6.00E-05	36.2
TcMYB62	ATMYB52	80.53	113	22	0	79	417	3	115	3.00E-67	205
TcMYB62	ATMYB52	32.89	76	44	2	793	999	170	245	2.00E-04	34.7
TcMYB62	AtMYB117	64.67	150	53	2	82	531	97	230	1.00E-65	204
TcMYB62	ATMYB105	45.49	233	119	4	1	675	80	309	2.00E-64	200
TcMYB62	AtMYB56	76.15	109	26	0	79	405	91	199	7.00E-59	186
TcMYB62	ATMYB110	57.05	156	61	3	16	465	38	187	7.00E-58	182
TcMYB62	ATMYB69	72.22	108	30	0	76	399	16	123	6.00E-57	178
TcMYB62	AtMYB89	60.95	105	41	0	88	402	57	161	2.00E-43	141
TcMYB62	ATMYB44	48.03	127	61	1	85	450	6	132	1.00E-40	137
TcMYB62	AtMYB109	53.85	104	48	0	85	396	56	159	6.00E-40	138
TcMYB62	ATMYB77	54.46	101	46	0	85	387	6	106	2.00E-39	134
TcMYB62	ATMYB25	52.78	108	51	0	85	408	50	157	9.00E-39	134
TcMYB62	AtMYB1	53.27	107	50	0	85	405	55	161	1.00E-38	134
TcMYB62	ATMYB73	51.49	101	49	0	85	387	13	113	1.00E-37	130
TcMYB62	AtMYB70	51.49	101	49	0	85	387	13	113	8.00E-37	127
TcMYB62	AtMYB64	44.53	128	71	2	22	405	86	208	2.00E-31	115
TcMYB62	AtMYB115	46.3	108	58	0	82	405	157	264	3.00E-31	114
TcMYB62	ATMYB118	41.18	136	80	2	61	468	181	312	1.00E-30	113
TcMYB62	ATMYB119	46.9	113	60	1	85	423	105	214	5.00E-30	111
TcMYB62	AtMYB98	39.44	142	83	2	16	432	193	334	2.00E-29	109
TcMYB62	AtMYB51	46.08	102	54	1	85	387	15	116	2.00E-28	105
TcMYB62	ATMYB63	43.36	113	62	2	70	402	11	122	6.00E-28	103
TcMYB62	ATMYB23	44.12	102	56	1	85	387	14	115	7.00E-28	101
TcMYB62	ATMYB67	41.09	129	74	2	85	465	24	151	8.00E-28	103
TcMYB62	ATMYB92	40.91	132	66	3	85	444	14	144	9.00E-28	103
TcMYB62	ATMYB66	44.12	102	56	1	85	387	18	119	9.00E-28	100
TcMYB62	AtMYB27	41.53	118	67	3	70	417	6	120	1.00E-27	101
TcMYB62	AtMYB100	45.45	99	53	2	91	384	28	125	5.00E-27	99.8
TcMYB62	ATMYB15	43.4	106	59	1	85	399	14	119	5.00E-27	100
TcMYB62	AtMYB19	47.57	103	52	2	85	387	14	115	5.00E-27	100
TcMYB62	AtMYB103	46.96	115	59	2	85	423	14	127	7.00E-27	102
TcMYB62	AtMYB53	45.37	108	57	2	70	387	9	115	1.00E-26	100
TcMYB62	AtMYB104	34.75	141	91	1	37	456	2	142	1.00E-26	101
TcMYB62	AtMYB36	34.55	165	91	4	85	528	14	178	1.00E-26	100
TcMYB62	ATMYB122	46.88	96	50	1	85	369	14	109	2.00E-26	100
TcMYB62	ATMYB0	43.14	102	57	1	85	387	16	117	2.00E-26	98.2
TcMYB62	AtMYB41	45.1	102	55	1	85	387	14	115	2.00E-26	99.4
TcMYB62	AtMYB10	44.66	103	55	2	85	387	16	117	2.00E-26	98.2
TcMYB62	AtMYB82	42.86	112	63	1	55	387	4	115	2.00E-26	97.1
TcMYB62	ATMYB58	38.85	139	84	2	70	483	11	142	3.00E-26	98.6
TcMYB62	AtMYB93	44.12	102	56	1	85	387	14	115	4.00E-26	99.8
TcMYB62	ATMYB14	46.6	103	53	2	85	387	14	115	5.00E-26	97.4
TcMYB62	ATMYB14	32.08	53	36	1	85	243	67	118	8.00E-05	35.8
TcMYB62	ATMYB80	46.6	103	53	2	85	387	14	115	6.00E-26	98.6
TcMYB62	AtMYB18	47.42	97	49	2	85	369	12	107	6.00E-26	97.8
TcMYB62	ATMYB101	31.2	266	163	8	85	822	20	279	7.00E-26	100
TcMYB62	ATMYB34	38.58	127	77	2	19	396	6	118	8.00E-26	97.8
TcMYB62	AtMYB47	42.72	103	57	2	85	387	14	115	9.00E-26	97.1
TcMYB62	AtMYB47	26.58	79	53	2	25	246	42	119	2.00E-04	34.7
TcMYB62	ATMYB84	35.47	172	99	6	85	564	14	182	1.00E-25	97.8
TcMYB62	ATMYB33	40.83	120	70	1	85	441	34	153	1.00E-25	100
TcMYB62	ATMYB35	45.1	102	55	1	85	387	14	115	1.00E-25	97.8
TcMYB62	AtMYB74	33.75	160	94	3	85	528	15	173	1.00E-25	97.8
TcMYB62	AtMYB49	43.14	102	57	1	85	387	14	115	2.00E-25	97.1
TcMYB62	AtMYB45	46.39	97	50	2	85	369	20	115	3.00E-25	95.5
TcMYB62	ATMYB16	44.12	102	56	1	85	387	14	115	3.00E-25	96.7
TcMYB62	ATMYB106	45.63	103	54	2	85	387	57	158	4.00E-25	97.4
TcMYB62	ATMYB65	42.73	110	62	1	85	411	43	152	4.00E-25	98.6
TcMYB62	ATMYB72	44.66	103	55	2	85	387	16	117	4.00E-25	95.9
TcMYB62	AtMYB17	45.63	103	54	2	85	387	14	115	4.00E-25	95.9
TcMYB62	ATMYB86	44.76	105	56	2	85	393	14	117	5.00E-25	96.7
TcMYB62	AtMYB114	38.05	113	69	1	85	420	10	122	5.00E-25	91.7
TcMYB62	ATMYB121	42.11	114	64	2	70	405	24	136	5.00E-25	95.1
TcMYB62	ATMYB75	34.38	128	83	1	85	465	10	137	6.00E-25	94.4
TcMYB62	AtMYB97	40.91	110	64	1	85	411	21	130	1.00E-24	96.3
TcMYB62	AtMYB9	42.16	102	58	1	85	387	14	115	1.00E-24	95.5
TcMYB62	AtMYB76	46.08	102	54	1	85	387	14	115	1.00E-24	95.5
TcMYB62	ATMYB28	43.81	105	58	1	85	396	14	118	1.00E-24	95.9
TcMYB62	ATMYB68	36.69	139	81	3	85	480	14	152	2.00E-24	95.5

TcMYB62	ATMYB13	44.66	103	55	2	85	387	14	115	2.00E-24	93.2
TcMYB62	ATMYB13	27.87	61	44	1	85	267	67	126	5.00E-04	33.5
TcMYB62	ATMYB71	36.55	145	90	3	85	513	20	161	2.00E-24	93.6
TcMYB62	ATMYB59-3	42.2	109	61	2	85	405	10	117	2.00E-24	92.8
TcMYB62	ATMYB95	42.72	103	57	2	85	387	14	115	2.00E-24	93.6
TcMYB62	ATMYB48	43.12	109	60	2	85	405	9	116	2.00E-24	93.2
TcMYB62	AtMYB107	42.16	102	58	1	85	387	14	115	2.00E-24	94.4
TcMYB62	ATMYB55	35.26	156	87	3	85	510	14	167	2.00E-24	94.7
TcMYB62	AtMYB50	44.66	103	55	2	85	387	14	115	3.00E-24	94
TcMYB62	ATMYB37	42.59	108	60	2	70	387	9	116	3.00E-24	94
TcMYB62	AtMYB83	35.53	152	97	1	85	537	32	183	4.00E-24	94
TcMYB62	ATMYB38	43.69	103	56	2	85	387	14	116	4.00E-24	93.2
TcMYB62	AtMYB40	44.66	103	55	2	85	387	14	115	4.00E-24	92.4
TcMYB62	AtMYB81	41.82	110	63	1	85	411	22	131	5.00E-24	94.7
TcMYB62	MYB8	38.24	102	62	1	85	387	14	115	8.00E-24	90.5
TcMYB62	ATMYB5	40.2	102	60	1	85	387	25	126	8.00E-24	91.3
TcMYB62	ATMYB29	45.83	96	51	1	85	369	14	109	9.00E-24	92.8
TcMYB62	AtMYB112	32.43	185	124	5	85	636	34	190	1.00E-23	90.9
TcMYB62	ATMYB61	44.34	106	57	2	85	396	14	118	1.00E-23	92.8
TcMYB62	ATMYB26	33.8	142	81	3	85	471	14	155	1.00E-23	92.8
TcMYB62	ATMYB120	35.81	148	94	3	85	525	28	167	1.00E-23	94
TcMYB62	ATMYB3	40.78	103	59	2	85	387	14	115	1.00E-23	90.9
TcMYB62	ATMYB123	33.14	169	108	4	85	576	16	171	1.00E-23	90.9
TcMYB62	ATMYB46	42.59	108	60	2	85	402	20	126	1.00E-23	91.3
TcMYB62	AtMYB6	38.32	107	64	2	85	399	14	119	2.00E-23	90.1
TcMYB62	AtMYB79	35.17	145	92	3	85	513	8	149	2.00E-23	90.5
TcMYB62	ATMYB102	42.72	103	57	2	85	387	14	115	2.00E-23	92
TcMYB62	ATMYB4	40.78	103	59	2	85	387	14	115	2.00E-23	90.9
TcMYB62	AtMYB43	29.12	261	162	7	85	798	14	266	2.00E-23	91.7
TcMYB62	ATMYB88	29.03	186	127	3	94	636	33	217	5.00E-23	92
TcMYB62	MYB7	33.77	154	84	6	85	492	14	166	6.00E-23	89.4
TcMYB62	ATMYB57	37.32	142	87	4	64	483	20	148	6.00E-23	87.8
TcMYB62	ATMYB90	38.83	103	62	1	85	390	10	112	1.00E-22	88.2
TcMYB62	AtMYB42	35.92	142	79	3	85	474	14	154	1.00E-22	88.6
TcMYB62	AtMYB113	37.14	105	65	1	85	396	10	114	2.00E-22	87.4
TcMYB62	AtMYB22	37.96	108	67	1	94	417	57	163	2.00E-22	87.4
TcMYB62	AtMYB22	28.89	45	32	0	235	369	52	96	5.00E-04	33.5
TcMYB62	AtMYB32	39.81	103	60	2	85	387	14	115	3.00E-22	87.4
TcMYB62	AtMYB62	37.7	122	74	2	85	444	21	141	4.00E-22	87.4
TcMYB62	ATMYB2	35.51	138	88	2	64	474	15	142	4.00E-22	87
TcMYB62	ATMYB111	37.84	111	68	1	85	414	14	124	6.00E-22	87.8
TcMYB62	AtMYB116	35.25	122	77	2	85	444	20	140	7.00E-22	86.7
TcMYB62	ATMYB94	41.18	102	59	1	85	387	14	115	7.00E-22	87.4
TcMYB62	AtMYB108	40.52	116	67	3	64	405	14	124	8.00E-22	87
TcMYB62	AtMYB20	42.45	106	59	2	85	396	14	118	9.00E-22	86.3
TcMYB62	ATMYB30	41.18	102	59	1	85	387	14	115	1.00E-21	86.7
TcMYB62	ATMYB87	31.79	151	101	3	85	531	14	158	2.00E-21	85.9
TcMYB62	ATMYB96	40.2	102	60	1	85	387	14	115	2.00E-21	86.3
TcMYB62	ATMYB31	40.2	102	60	1	85	387	14	115	2.00E-21	85.9
TcMYB62	AtMYB85	42.72	103	57	2	85	387	14	115	3.00E-21	84.7
TcMYB62	AtMYB124	38.83	103	63	0	94	402	28	130	4.00E-21	86.3
TcMYB62	ATMYB12	38.24	102	62	1	85	387	14	115	5.00E-21	85.5
TcMYB62	AtMYB24	41.05	95	55	1	85	366	19	113	2.00E-20	81.3
TcMYB62	ATMYB3	41.05	95	55	1	85	366	22	116	4.00E-20	80.5
TcMYB62	AtMYB60	38.24	102	62	1	85	387	14	115	5.00E-20	81.3
TcMYB62	ATMYB11	37.25	102	63	1	85	387	14	115	6.00E-20	82
TcMYB62	ATMYB99	40.91	110	56	3	85	387	15	123	1.00E-18	77
TcMYB62	ATMYB91	33.64	110	70	1	85	405	4	113	1.00E-18	78.2
TcMYB62	ATMYB78	34.88	129	69	3	64	405	21	145	2.00E-18	77.4
TcMYB63	AtMYB50	68.45	187	56	3	25	576	2	184	3.00E-84	253
TcMYB63	ATMYB61	43.26	356	190	8	28	1059	3	311	1.00E-79	243
TcMYB63	ATMYB86	48.74	277	126	5	28	810	3	268	1.00E-78	240
TcMYB63	ATMYB55	57.69	208	74	3	28	609	3	208	1.00E-76	234
TcMYB63	ATMYB67	53.14	207	97	5	4	624	7	197	8.00E-69	213
TcMYB63	AtMYB32	59.33	150	61	1	28	477	3	146	4.00E-62	194
TcMYB63	MYB7	61.43	140	54	0	28	447	3	142	6.00E-61	191
TcMYB63	ATMYB35	46.01	213	109	3	28	648	3	212	6.00E-61	193
TcMYB63	ATMYB106	42.57	249	143	2	37	783	49	285	1.00E-60	194
TcMYB63	AtMYB42	55.95	168	74	2	28	531	3	166	1.00E-60	191
TcMYB63	AtMYB43	46.26	227	103	2	28	651	3	229	2.00E-60	192
TcMYB63	ATMYB26	66.91	139	37	1	31	420	4	142	2.00E-60	193
TcMYB63	ATMYB122	50.81	185	91	2	28	582	3	181	5.00E-60	191
TcMYB63	ATMYB16	50	196	89	3	28	588	3	194	6.00E-60	191
TcMYB63	ATMYB4	57.53	146	62	0	28	465	3	148	3.00E-59	187
TcMYB63	AtMYB85	48.51	202	104	4	28	633	3	191	5.00E-59	186
TcMYB63	AtMYB93	50.52	194	89	2	28	588	3	194	9.00E-59	189
TcMYB63	AtMYB103	58.78	148	61	1	31	474	4	147	1.00E-58	189
TcMYB63	ATMYB3	62.79	129	48	0	28	414	3	131	1.00E-58	185
TcMYB63	AtMYB17	54.09	159	73	0	28	504	3	161	2.00E-58	186
TcMYB63	ATMYB102	34.51	339	187	8	28	939	3	337	2.00E-58	187

TcMYB63	AtMYB6	52.66	169	80	1	28	534	3	165	2.00E-58	184
TcMYB63	AtMYB107	39.72	282	150	6	28	813	3	284	3.00E-58	186
TcMYB63	MYB8	62.02	129	49	0	28	414	3	131	1.00E-57	181
TcMYB63	ATMYB80	42.34	248	108	7	37	675	6	248	2.00E-57	184
TcMYB63	ATMYB46	52.51	179	83	2	49	579	16	192	2.00E-57	182
TcMYB63	AtMYB20	62.5	136	51	0	28	435	3	138	4.00E-57	182
TcMYB63	AtMYB53	69.83	116	35	0	55	402	12	127	5.00E-57	182
TcMYB63	AtMYB51	47.14	210	109	4	28	651	3	209	5.00E-57	184
TcMYB63	AtMYB74	64.57	127	45	0	19	399	1	127	7.00E-57	182
TcMYB63	ATMYB28	45.41	207	106	2	28	627	3	207	8.00E-57	184
TcMYB63	ATMYB34	44.93	207	114	1	28	648	3	205	9.00E-57	181
TcMYB63	AtMYB9	63.2	125	46	0	28	402	3	127	5.00E-56	181
TcMYB63	ATMYB92	64.46	121	43	0	55	417	12	132	7.00E-56	180
TcMYB63	ATMYB72	47.62	189	93	3	19	567	1	184	1.00E-55	179
TcMYB63	ATMYB5	63.71	124	45	0	37	408	17	140	2.00E-55	176
TcMYB63	AtMYB40	55.41	157	67	2	19	480	1	156	3.00E-55	176
TcMYB63	ATMYB13	55.49	164	68	3	19	495	1	161	5.00E-55	175
TcMYB63	AtMYB41	63.71	124	45	0	28	399	3	126	1.00E-54	176
TcMYB63	AtMYB10	53.09	162	72	2	25	498	4	163	1.00E-54	174
TcMYB63	AtMYB83	66.94	124	37	1	13	372	12	135	1.00E-54	177
TcMYB63	ATMYB63	41.81	232	120	5	19	669	1	221	2.00E-54	175
TcMYB63	ATMYB29	44.17	206	115	1	28	645	3	199	3.00E-54	176
TcMYB63	AtMYB49	66.95	118	39	0	46	399	9	126	5.00E-54	175
TcMYB63	AtMYB76	45.45	209	107	3	40	645	7	215	9.00E-54	175
TcMYB63	ATMYB95	46.96	181	90	2	55	579	12	180	1.00E-53	172
TcMYB63	ATMYB15	42.65	204	114	4	28	630	3	199	9.00E-53	171
TcMYB63	ATMYB99	57.55	139	52	1	19	414	1	139	2.00E-52	169
TcMYB63	ATMYB14	45.07	213	106	6	28	633	3	203	4.00E-52	168
TcMYB63	ATMYB58	54	150	68	2	19	465	1	145	1.00E-51	167
TcMYB63	AtMYB47	60.17	118	47	0	55	408	12	129	2.00E-50	164
TcMYB63	AtMYB60	44.63	177	97	1	28	555	3	179	1.00E-49	162
TcMYB63	AtMYB19	43.48	207	115	3	46	660	9	199	2.00E-49	161
TcMYB63	AtMYB45	51.43	140	68	0	46	465	15	154	3.00E-49	160
TcMYB63	ATMYB37	43.5	200	112	3	28	624	3	182	1.00E-48	161
TcMYB63	ATMYB12	52.38	147	70	0	28	468	3	149	3.00E-48	161
TcMYB63	ATMYB111	64.91	114	40	0	28	369	3	116	5.00E-48	160
TcMYB63	ATMYB94	33.81	352	224	10	28	1056	3	318	8.00E-48	159
TcMYB63	AtMYB36	44.2	181	96	3	28	555	3	179	8.00E-48	159
TcMYB63	ATMYB96	51.68	149	72	2	28	474	3	148	2.00E-47	159
TcMYB63	ATMYB30	48.99	149	76	0	28	474	3	151	3.00E-47	157
TcMYB63	AtMYB18	43.87	212	111	4	49	660	8	217	5.00E-47	155
TcMYB63	ATMYB11	43.08	195	99	1	28	576	3	197	5.00E-47	157
TcMYB63	ATMYB71	44.57	184	102	3	58	609	19	192	1.00E-46	154
TcMYB63	AtMYB79	42.79	201	112	2	58	651	7	207	1.00E-46	154
TcMYB63	ATMYB123	61.02	118	45	1	19	369	1	118	2.00E-46	153
TcMYB63	ATMYB66	42.46	179	98	3	58	579	17	193	6.00E-46	150
TcMYB63	ATMYB84	59.32	118	47	1	28	378	3	120	7.00E-46	153
TcMYB63	ATMYB57	48.1	158	77	2	13	471	6	161	5.00E-45	148
TcMYB63	ATMYB38	50	142	70	1	28	450	3	144	5.00E-45	150
TcMYB63	ATMYB23	45.73	164	87	4	46	531	9	167	7.00E-45	148
TcMYB63	ATMYB68	58.47	118	48	1	28	378	3	120	1.00E-44	152
TcMYB63	ATMYB31	60.53	114	45	0	28	369	3	116	1.00E-44	150
TcMYB63	ATMYB3	47.13	157	82	3	52	519	19	169	2.00E-44	147
TcMYB63	ATMYB87	47.77	157	80	2	28	492	3	159	2.00E-44	149
TcMYB63	ATMYB121	44.31	167	93	1	46	546	24	188	4.00E-44	147
TcMYB63	ATMYB0	57.94	107	45	0	49	369	12	118	5.00E-44	146
TcMYB63	AtMYB82	57.94	107	45	0	46	366	9	115	2.00E-43	143
TcMYB63	AtMYB114	60.58	104	41	0	55	366	8	111	3.00E-43	141
TcMYB63	AtMYB62	39.3	201	122	2	49	651	17	203	8.00E-43	144
TcMYB63	AtMYB116	45.58	147	80	0	58	498	19	165	1.00E-42	144
TcMYB63	AtMYB108	56.03	116	51	0	55	402	19	134	2.00E-42	144
TcMYB63	AtMYB112	57.01	107	46	0	52	372	31	137	2.00E-42	142
TcMYB63	ATMYB59-3	43.95	157	82	1	46	498	5	161	2.00E-42	142
TcMYB63	AtMYB81	48.12	133	58	1	46	411	17	149	2.00E-42	147
TcMYB63	ATMYB90	55.46	119	44	1	55	384	8	126	2.00E-42	142
TcMYB63	ATMYB48	48.53	136	70	0	58	465	8	143	3.00E-42	142
TcMYB63	ATMYB75	55	120	46	1	55	390	8	127	3.00E-42	142
TcMYB63	ATMYB101	42.86	161	92	1	55	537	18	177	3.00E-42	147
TcMYB63	ATMYB33	61.54	104	40	0	55	366	32	135	5.00E-42	147
TcMYB63	AtMYB97	59.22	103	42	0	55	363	19	121	1.00E-41	144
TcMYB63	ATMYB120	58.88	107	44	0	55	375	26	132	1.00E-41	146
TcMYB63	AtMYB24	53.72	121	56	0	52	414	16	136	2.00E-41	139
TcMYB63	ATMYB65	59.62	104	42	0	55	366	41	144	1.00E-40	144
TcMYB63	AtMYB113	56.48	108	47	0	55	378	8	115	7.00E-40	135
TcMYB63	AtMYB27	56.07	107	47	0	46	366	6	112	3.00E-39	134
TcMYB63	ATMYB2	47.83	115	60	0	55	399	20	134	1.00E-37	130
TcMYB63	AtMYB104	51.33	113	55	1	46	384	13	124	3.00E-36	129
TcMYB63	ATMYB78	46.15	130	56	1	55	402	26	155	5.00E-36	127
TcMYB63	AtMYB98	45.05	111	61	1	46	378	212	321	3.00E-30	113
TcMYB63	ATMYB77	33.66	205	129	4	61	654	6	201	1.00E-29	109

TcMYB63	ATMYB25	36.81	182	105	4	61	576	50	229	2.00E-29	110
TcMYB63	ATMYB73	49.07	108	54	2	61	381	13	118	4.00E-29	108
TcMYB63	ATMYB44	50.49	103	50	2	61	366	6	106	4.00E-29	108
TcMYB63	AtMYB70	49.07	108	54	2	61	381	13	118	1.00E-28	107
TcMYB63	ATMYB118	40.16	122	71	2	7	366	169	289	3.00E-28	108
TcMYB63	ATMYB118	29.09	55	38	1	220	381	189	243	1.00E-04	35.8
TcMYB63	ATMYB54	32.99	197	121	5	61	618	6	191	3.00E-28	104
TcMYB63	ATMYB119	39.39	132	79	3	49	441	101	225	9.00E-28	106
TcMYB63	AtMYB115	44.76	105	56	2	43	351	150	253	1.00E-27	105
TcMYB63	AtMYB64	31.77	192	130	6	61	633	105	282	3.00E-27	105
TcMYB63	AtMYB1	41.43	140	81	3	61	477	55	188	4.00E-27	104
TcMYB63	AtMYB109	51.46	103	49	2	61	366	56	156	2.00E-26	102
TcMYB63	ATMYB52	36.81	144	84	3	61	471	5	146	3.00E-26	99
TcMYB63	ATMYB105	41.88	117	65	3	37	378	97	211	4.00E-26	100
TcMYB63	AtMYB117	43.52	108	60	2	55	375	96	201	7.00E-25	97.4
TcMYB63	AtMYB100	38.39	112	69	1	46	381	21	131	3.00E-23	90.5
TcMYB63	ATMYB69	38.74	111	67	2	46	375	14	122	3.00E-23	90.5
TcMYB63	ATMYB110	32.82	131	88	2	61	453	67	191	6.00E-23	90.9
TcMYB63	AtMYB56	39.05	105	64	1	61	375	93	196	8.00E-23	90.9
TcMYB63	ATMYB91	28.39	236	147	7	70	711	7	233	3.00E-22	89.7
TcMYB63	ATMYB88	27.8	223	137	5	70	666	33	246	4.00E-20	84.3
TcMYB63	AtMYB124	32.35	136	89	2	70	468	28	162	9.00E-20	83.2
TcMYB63	AtMYB22	37.5	112	69	4	46	378	49	156	2.00E-19	80.1
TcMYB63	AtMYB89	32.43	111	75	1	46	378	51	160	3.00E-18	75.1
TcMYB64	ATMYB77	53.64	220	93	5	19	651	2	213	8.00E-68	211
TcMYB64	ATMYB44	54.55	209	95	4	19	645	2	200	5.00E-67	209
TcMYB64	ATMYB73	50.88	228	105	6	22	684	10	215	7.00E-64	201
TcMYB64	AtMYB70	49.39	247	105	7	22	702	10	241	1.00E-63	200
TcMYB64	AtMYB109	52.38	168	79	3	13	513	50	206	2.00E-52	173
TcMYB64	AtMYB1	44.55	202	91	3	10	552	48	249	1.00E-48	163
TcMYB64	ATMYB25	60.18	113	45	0	4	342	41	153	2.00E-43	148
TcMYB64	ATMYB119	37.27	161	98	1	7	480	97	257	5.00E-37	132
TcMYB64	AtMYB56	53.85	104	48	0	31	342	93	196	1.00E-36	129
TcMYB64	ATMYB52	50	104	52	0	31	342	5	108	1.00E-36	127
TcMYB64	ATMYB54	47.15	123	65	0	31	399	6	128	2.00E-36	127
TcMYB64	ATMYB105	52.88	104	49	0	31	342	107	210	2.00E-36	129
TcMYB64	AtMYB117	51.85	108	52	0	31	354	98	205	3.00E-36	129
TcMYB64	ATMYB69	50.96	104	51	0	31	342	19	122	1.00E-35	125
TcMYB64	AtMYB64	45.76	118	64	0	28	381	104	221	1.00E-34	125
TcMYB64	ATMYB110	33.48	227	128	4	31	642	67	274	9.00E-34	121
TcMYB64	ATMYB118	46.56	131	70	1	19	411	185	311	3.00E-33	122
TcMYB64	ATMYB59-3	31.02	216	147	5	31	672	10	202	9.00E-33	116
TcMYB64	ATMYB121	36.22	196	123	4	31	612	29	214	2.00E-32	117
TcMYB64	ATMYB63	39.52	167	94	4	31	510	16	178	7.00E-32	115
TcMYB64	ATMYB46	41.84	141	81	1	31	450	20	160	1.00E-31	115
TcMYB64	ATMYB48	39.02	164	98	4	1	486	2	151	1.00E-31	114
TcMYB64	ATMYB101	37.5	184	105	5	31	552	20	199	2.00E-31	117
TcMYB64	AtMYB98	50	102	51	0	28	333	216	317	4.00E-31	116
TcMYB64	ATMYB14	41.43	140	78	5	31	438	14	146	3.00E-30	110
TcMYB64	AtMYB116	43.24	111	62	1	19	348	16	126	5.00E-30	110
TcMYB64	ATMYB96	30.54	239	155	5	31	714	14	227	1.00E-29	110
TcMYB64	AtMYB17	51.46	103	48	2	31	333	14	115	1.00E-29	109
TcMYB64	AtMYB115	47	100	53	0	31	330	158	257	3.00E-29	110
TcMYB64	AtMYB62	44.44	108	58	2	31	348	21	127	3.00E-29	108
TcMYB64	AtMYB97	50.5	101	49	1	31	330	21	121	3.00E-29	110
TcMYB64	AtMYB79	47.62	105	53	2	31	339	8	111	3.00E-29	107
TcMYB64	AtMYB51	50	102	50	1	31	333	15	116	3.00E-29	109
TcMYB64	ATMYB30	35.24	210	116	7	31	600	14	215	4.00E-29	108
TcMYB64	ATMYB57	35.62	160	102	3	7	483	21	170	4.00E-29	105
TcMYB64	ATMYB92	49.51	103	50	2	31	333	14	115	4.00E-29	108
TcMYB64	ATMYB65	48.08	104	53	1	31	339	43	146	5.00E-29	111
TcMYB64	ATMYB72	39.46	147	87	3	31	465	16	160	6.00E-29	107
TcMYB64	AtMYB24	33.53	170	106	3	13	501	13	182	7.00E-29	105
TcMYB64	ATMYB34	48.57	105	53	1	31	342	14	118	1.00E-28	107
TcMYB64	AtMYB41	49.51	103	50	2	31	333	14	115	1.00E-28	106
TcMYB64	ATMYB35	46.73	107	55	2	31	345	14	119	1.00E-28	107
TcMYB64	AtMYB10	42.11	133	75	2	31	423	16	147	1.00E-28	105
TcMYB64	ATMYB15	39.13	138	82	2	31	438	14	150	2.00E-28	106
TcMYB64	AtMYB45	38.27	162	96	4	31	504	20	177	2.00E-28	105
TcMYB64	AtMYB107	47.57	103	52	2	31	333	14	115	2.00E-28	106
TcMYB64	ATMYB120	50.5	101	49	1	31	330	28	128	2.00E-28	109
TcMYB64	AtMYB83	46.72	122	62	2	31	387	32	153	3.00E-28	107
TcMYB64	ATMYB67	42.75	131	73	3	31	417	24	151	3.00E-28	106
TcMYB64	ATMYB33	48.08	104	53	1	31	339	34	137	3.00E-28	108
TcMYB64	AtMYB60	39.46	147	78	3	31	438	14	159	6.00E-28	104
TcMYB64	ATMYB71	45.71	105	55	2	31	339	20	123	6.00E-28	104
TcMYB64	ATMYB26	44.44	117	55	2	31	351	14	130	7.00E-28	106
TcMYB64	AtMYB89	43.43	99	56	0	34	330	57	155	7.00E-28	102
TcMYB64	ATMYB122	41.3	138	71	3	31	414	14	151	8.00E-28	105
TcMYB64	ATMYB80	47.06	102	53	1	31	333	14	115	8.00E-28	105

TcMYB64	AtMYB27	44.95	109	58	2	31	351	11	118	9.00E-28	103
TcMYB64	AtMYB53	48.54	103	51	2	31	333	14	115	1.00E-27	104
TcMYB64	ATMYB13	39.33	150	79	4	31	444	14	159	1.00E-27	103
TcMYB64	ATMYB94	29.7	266	185	6	31	822	14	268	1.00E-27	105
TcMYB64	AtMYB18	43.9	123	65	2	31	387	12	134	1.00E-27	103
TcMYB64	AtMYB40	41.67	120	65	3	1	345	1	119	1.00E-27	103
TcMYB64	AtMYB47	37.18	156	79	4	31	441	14	168	2.00E-27	103
TcMYB64	ATMYB106	42.11	133	76	2	31	426	57	183	2.00E-27	105
TcMYB64	ATMYB87	39.58	144	76	3	31	429	14	157	2.00E-27	103
TcMYB64	ATMYB3	38.81	134	81	2	31	429	22	150	2.00E-27	102
TcMYB64	AtMYB19	47.57	103	52	2	31	333	14	115	3.00E-27	102
TcMYB64	AtMYB74	48.04	102	52	1	31	333	15	116	3.00E-27	103
TcMYB64	ATMYB55	39.87	158	70	4	31	429	14	171	3.00E-27	103
TcMYB64	ATMYB37	49.51	103	50	2	31	333	14	116	3.00E-27	103
TcMYB64	ATMYB31	40.15	132	76	3	31	417	14	144	3.00E-27	103
TcMYB64	ATMYB95	47.57	103	52	2	31	333	14	115	4.00E-27	102
TcMYB64	ATMYB86	48.54	103	51	2	31	333	14	115	5.00E-27	103
TcMYB64	ATMYB38	49.51	103	50	2	31	333	14	116	5.00E-27	102
TcMYB64	ATMYB16	46.6	103	53	2	31	333	14	115	6.00E-27	102
TcMYB64	AtMYB103	45.37	108	58	1	31	351	14	121	6.00E-27	103
TcMYB64	ATMYB2	45.05	111	59	2	19	345	18	127	6.00E-27	101
TcMYB64	AtMYB36	39.86	138	77	4	31	426	14	146	7.00E-27	102
TcMYB64	ATMYB3	41.09	129	75	1	31	414	14	142	1.00E-26	100
TcMYB64	ATMYB111	39.35	155	88	4	31	477	14	167	1.00E-26	102
TcMYB64	ATMYB58	39.19	148	86	4	31	462	16	162	2.00E-26	100
TcMYB64	ATMYB66	43.24	111	61	2	7	333	10	119	2.00E-26	98.6
TcMYB64	AtMYB9	47.57	103	52	2	31	333	14	115	2.00E-26	101
TcMYB64	ATMYB102	47.06	102	53	1	31	333	14	115	2.00E-26	101
TcMYB64	ATMYB0	40.62	128	73	2	7	381	8	135	2.00E-26	99
TcMYB64	AtMYB49	45.1	102	55	1	31	333	14	115	3.00E-26	100
TcMYB64	AtMYB93	46.08	102	54	1	31	333	14	115	3.00E-26	101
TcMYB64	ATMYB61	38.85	157	92	4	31	489	14	163	3.00E-26	101
TcMYB64	AtMYB108	42.73	110	61	2	22	345	18	126	3.00E-26	100
TcMYB64	AtMYB82	43.24	111	61	2	7	333	6	115	3.00E-26	97.8
TcMYB64	AtMYB50	40.85	142	71	4	31	417	14	154	3.00E-26	100
TcMYB64	ATMYB28	34.29	175	114	2	31	552	14	181	4.00E-26	101
TcMYB64	AtMYB81	48.51	101	51	1	31	330	22	122	4.00E-26	102
TcMYB64	AtMYB85	39.71	136	80	3	31	432	14	142	7.00E-26	98.6
TcMYB64	MYB7	38.31	154	90	4	31	477	14	167	7.00E-26	98.6
TcMYB64	ATMYB23	45.63	103	54	2	31	333	14	115	1.00E-25	96.7
TcMYB64	MYB8	45.1	102	55	1	31	333	14	115	2.00E-25	95.9
TcMYB64	AtMYB6	46.08	102	54	1	31	333	14	115	3.00E-25	96.3
TcMYB64	ATMYB4	39.42	137	82	2	31	438	14	144	5.00E-25	96.7
TcMYB64	ATMYB5	46.08	102	54	1	31	333	25	126	5.00E-25	95.9
TcMYB64	ATMYB68	46.6	103	53	2	31	333	14	116	7.00E-25	97.8
TcMYB64	AtMYB104	44.76	105	53	2	31	330	18	118	7.00E-25	97.8
TcMYB64	ATMYB123	44.66	103	55	2	31	333	16	117	8.00E-25	95.5
TcMYB64	ATMYB84	45.63	103	54	2	31	333	14	116	1.00E-24	96.3
TcMYB64	AtMYB76	45.1	102	55	1	31	333	14	115	1.00E-24	96.7
TcMYB64	ATMYB29	45.54	101	54	1	31	330	14	114	1.00E-24	96.3
TcMYB64	AtMYB112	39.62	106	63	1	31	345	34	139	1.00E-24	94.4
TcMYB64	AtMYB20	41.12	107	61	2	31	345	14	119	2.00E-24	95.1
TcMYB64	AtMYB100	32.61	138	92	2	7	417	20	155	3.00E-24	93.2
TcMYB64	AtMYB43	42.06	107	60	2	31	345	14	119	4.00E-24	94.7
TcMYB64	AtMYB114	40.95	105	60	2	31	339	10	113	6.00E-24	89.7
TcMYB64	AtMYB32	45.63	103	54	2	31	333	14	115	7.00E-24	93.2
TcMYB64	ATMYB12	43.14	102	57	1	31	333	14	115	7.00E-24	94.7
TcMYB64	ATMYB12	23.84	151	112	4	19	462	63	207	1.00E-04	35.8
TcMYB64	ATMYB11	45.1	102	55	1	31	333	14	115	1.00E-23	94
TcMYB64	AtMYB42	43.69	103	56	2	31	333	14	115	1.00E-23	92.8
TcMYB64	ATMYB78	36.67	120	61	2	31	345	28	147	3.00E-22	89.4
TcMYB64	ATMYB75	40	105	61	2	31	339	10	113	3.00E-22	87.8
TcMYB64	AtMYB113	40.57	106	61	2	31	342	10	114	4.00E-22	87.4
TcMYB64	ATMYB99	42.73	110	54	3	31	333	15	123	8.00E-22	86.7
TcMYB64	ATMYB88	30.92	152	100	2	40	480	33	178	1.00E-21	89
TcMYB64	ATMYB88	29.85	67	47	1	19	219	78	143	9.00E-05	36.6
TcMYB64	ATMYB90	40	105	61	2	31	339	10	113	2.00E-21	85.5
TcMYB64	AtMYB22	27.46	193	139	4	19	594	50	227	2.00E-21	85.5
TcMYB64	AtMYB124	34	100	66	0	40	339	28	127	2.00E-20	85.1
TcMYB64	AtMYB124	31.82	66	45	1	19	216	73	137	7.00E-06	40
TcMYB64	ATMYB91	35.58	104	64	1	40	342	7	110	5.00E-18	77.4
TcMYB65	AtMYB62	69.09	110	34	0	7	336	13	122	2.00E-60	184
TcMYB65	ATMYB71	48.11	185	94	1	16	564	15	199	3.00E-60	184
TcMYB65	AtMYB116	66.97	109	36	0	10	336	13	121	1.00E-57	177
TcMYB65	AtMYB112	69.81	106	32	0	16	333	29	134	1.00E-57	176
TcMYB65	AtMYB108	67.59	108	35	0	10	333	14	121	5.00E-57	177
TcMYB65	ATMYB59-3	55.71	140	57	1	16	420	5	144	2.00E-56	173
TcMYB65	ATMYB3	66.98	106	35	0	16	333	17	122	4.00E-56	172
TcMYB65	ATMYB48	61.42	127	45	1	13	381	3	129	5.00E-56	172
TcMYB65	AtMYB79	51.28	156	76	1	16	483	3	149	2.00E-55	171

TcMYB65	AtMYB24	66.04	106	36	0	16	333	14	119	2.00E-55	169
TcMYB65	ATMYB57	48.8	166	82	1	7	495	19	184	7.00E-55	168
TcMYB65	ATMYB121	62.5	120	43	1	1	354	17	136	1.00E-54	169
TcMYB65	ATMYB2	62.39	109	41	0	10	336	15	123	2.00E-54	169
TcMYB65	ATMYB78	60.66	122	34	1	10	333	21	142	2.00E-53	167
TcMYB65	AtMYB20	42.86	182	103	3	25	567	12	183	5.00E-49	155
TcMYB65	AtMYB85	61.74	115	43	1	25	366	12	126	7.00E-49	154
TcMYB65	ATMYB3	55.8	138	60	1	13	423	8	145	8.00E-49	154
TcMYB65	MYB8	60.5	119	46	1	13	366	8	126	9.00E-49	152
TcMYB65	MYB7	63.03	119	43	1	13	366	8	126	2.00E-48	153
TcMYB65	AtMYB27	59.65	114	46	0	10	351	4	117	4.00E-48	152
TcMYB65	ATMYB4	63.03	119	43	1	13	366	8	126	6.00E-48	152
TcMYB65	AtMYB42	65.38	104	36	0	25	336	12	115	7.00E-48	152
TcMYB65	AtMYB40	40.17	229	105	7	25	615	12	232	1.00E-47	151
TcMYB65	AtMYB6	55.63	142	58	3	13	423	8	149	2.00E-47	150
TcMYB65	AtMYB43	50.7	142	69	2	25	447	12	150	4.00E-47	152
TcMYB65	AtMYB32	47.56	164	81	3	13	489	8	168	4.00E-47	150
TcMYB65	AtMYB107	61.11	108	42	0	13	336	8	115	6.00E-47	151
TcMYB65	ATMYB12	62.73	110	41	0	13	342	8	117	7.00E-47	152
TcMYB65	ATMYB14	60.91	110	43	0	13	342	8	117	8.00E-47	149
TcMYB65	ATMYB13	45.24	168	92	2	13	516	8	166	3.00E-46	147
TcMYB65	AtMYB49	62.04	108	41	0	13	336	8	115	3.00E-46	149
TcMYB65	AtMYB41	57.5	120	50	1	13	369	8	127	4.00E-46	148
TcMYB65	ATMYB66	42.51	167	96	1	7	507	10	174	7.00E-46	145
TcMYB65	ATMYB5	40.57	212	119	5	25	639	23	227	9.00E-46	146
TcMYB65	ATMYB101	43.26	178	95	3	13	528	14	186	2.00E-45	151
TcMYB65	ATMYB11	60.91	110	43	0	13	342	8	117	4.00E-45	147
TcMYB65	ATMYB63	37.81	201	125	3	13	615	10	201	5.00E-45	145
TcMYB65	ATMYB61	50.34	145	71	2	16	447	9	145	7.00E-45	147
TcMYB65	AtMYB9	59.26	108	44	0	13	336	8	115	1.00E-44	145
TcMYB65	ATMYB92	41.88	191	106	3	13	570	8	188	1.00E-44	145
TcMYB65	ATMYB99	56.35	126	47	2	13	366	9	134	2.00E-44	142
TcMYB65	ATMYB86	57.63	118	49	1	16	366	9	126	2.00E-44	145
TcMYB65	AtMYB74	55.37	121	53	1	10	369	8	128	2.00E-44	144
TcMYB65	ATMYB23	53.98	113	52	0	13	351	8	120	3.00E-44	141
TcMYB65	AtMYB83	58.47	118	47	1	22	369	29	146	3.00E-44	144
TcMYB65	ATMYB46	61.32	106	41	0	19	336	16	121	4.00E-44	142
TcMYB65	ATMYB72	42.78	180	89	4	13	510	10	185	6.00E-44	142
TcMYB65	ATMYB111	60.36	111	44	0	13	345	8	118	7.00E-44	144
TcMYB65	AtMYB50	55.74	122	53	1	16	378	9	130	7.00E-44	143
TcMYB65	ATMYB102	57.5	120	50	1	13	369	8	127	8.00E-44	144
TcMYB65	ATMYB58	54.05	111	51	0	13	345	10	120	1.00E-43	141
TcMYB65	AtMYB103	52.99	117	55	1	16	366	9	122	2.00E-43	143
TcMYB65	AtMYB97	61.17	103	40	0	25	333	19	121	2.00E-43	143
TcMYB65	ATMYB67	44.83	145	79	3	16	447	19	157	3.00E-43	141
TcMYB65	ATMYB106	41.28	172	91	2	25	510	55	226	3.00E-43	143
TcMYB65	ATMYB30	53.45	116	54	0	25	372	12	127	5.00E-43	141
TcMYB65	AtMYB53	53.78	119	54	1	13	366	8	126	5.00E-43	140
TcMYB65	ATMYB96	40.56	180	107	2	13	552	8	170	6.00E-43	141
TcMYB65	AtMYB51	59.62	104	42	0	25	336	13	116	6.00E-43	141
TcMYB65	AtMYB60	58.49	106	44	0	25	342	12	117	7.00E-43	139
TcMYB65	ATMYB123	59.43	106	43	0	19	336	12	117	8.00E-43	139
TcMYB65	ATMYB35	38.38	185	112	2	13	561	8	192	8.00E-43	140
TcMYB65	ATMYB34	56.48	108	47	0	13	336	8	115	9.00E-43	139
TcMYB65	ATMYB120	59.26	108	44	0	25	348	26	133	1.00E-42	144
TcMYB65	AtMYB82	48.36	122	63	0	7	372	6	127	3.00E-42	135
TcMYB65	AtMYB17	57.39	115	48	1	25	366	12	126	3.00E-42	138
TcMYB65	ATMYB94	55.96	109	48	0	25	351	12	120	3.00E-42	139
TcMYB65	ATMYB26	50.79	126	53	1	16	366	9	134	3.00E-42	140
TcMYB65	ATMYB33	56.48	108	47	0	25	348	32	139	4.00E-42	142
TcMYB65	AtMYB10	48.84	129	60	1	13	381	10	138	4.00E-42	136
TcMYB65	ATMYB0	52.73	110	52	0	7	336	8	117	5.00E-42	135
TcMYB65	ATMYB95	53.33	120	55	1	25	381	12	131	6.00E-42	137
TcMYB65	ATMYB122	43.68	174	97	4	19	537	10	170	6.00E-42	138
TcMYB65	ATMYB80	49.22	128	64	1	13	393	8	135	7.00E-42	138
TcMYB65	ATMYB29	45.33	150	78	3	19	456	10	159	7.00E-42	138
TcMYB65	ATMYB65	54.63	108	49	0	25	348	41	148	7.00E-42	142
TcMYB65	ATMYB31	56.48	108	47	0	13	336	8	115	8.00E-42	138
TcMYB65	AtMYB93	56.48	108	47	0	13	336	8	115	9.00E-42	139
TcMYB65	ATMYB16	58.65	104	43	0	25	336	12	115	1.00E-41	137
TcMYB65	ATMYB15	57.41	108	46	0	13	336	8	115	2.00E-41	135
TcMYB65	AtMYB76	48.41	126	64	1	19	393	10	135	4.00E-41	136
TcMYB65	AtMYB19	52.25	111	53	0	7	339	6	116	8.00E-41	134
TcMYB65	AtMYB81	55.66	106	47	0	16	333	17	122	2.00E-40	136
TcMYB65	ATMYB28	45.07	142	74	3	19	432	10	151	3.00E-40	135
TcMYB65	ATMYB55	42.46	179	77	5	16	474	9	185	2.00E-39	132
TcMYB65	AtMYB18	42.77	173	96	4	19	528	8	175	3.00E-39	130
TcMYB65	AtMYB45	51.85	108	52	0	10	333	13	120	4.00E-39	129
TcMYB65	ATMYB37	41.33	150	87	2	13	459	8	153	4.00E-38	128
TcMYB65	ATMYB38	47.9	119	61	1	13	366	8	126	1.00E-37	126

TcMYB65	AtMYB36	37.57	173	105	3	13	522	8	177	1.00E-37	127
TcMYB65	ATMYB87	42.14	159	91	3	25	498	12	158	1.00E-37	126
TcMYB65	ATMYB84	47.54	122	63	1	13	375	8	129	2.00E-37	126
TcMYB65	AtMYB47	49.57	115	57	1	25	366	12	126	6.00E-37	124
TcMYB65	ATMYB68	51.38	109	52	1	13	336	8	116	1.00E-36	125
TcMYB65	AtMYB114	48.18	110	57	0	7	336	2	111	7.00E-35	114
TcMYB65	ATMYB75	48.18	110	57	0	7	336	2	111	5.00E-34	115
TcMYB65	ATMYB90	38.01	171	106	2	7	519	2	158	5.00E-34	115
TcMYB65	AtMYB113	49.04	104	53	0	25	336	8	111	2.00E-33	114
TcMYB65	ATMYB119	34.78	161	91	5	7	447	97	255	7.00E-30	108
TcMYB65	ATMYB25	48.04	102	53	1	31	336	50	150	2.00E-29	105
TcMYB65	AtMYB70	45.28	106	58	1	31	348	13	117	5.00E-29	103
TcMYB65	ATMYB77	42.45	106	61	1	31	348	6	110	7.00E-29	103
TcMYB65	AtMYB64	42.34	111	63	2	7	336	97	205	1.00E-28	104
TcMYB65	AtMYB109	38.36	146	84	4	31	450	56	199	2.00E-28	103
TcMYB65	AtMYB1	47.57	103	54	1	31	339	55	156	2.00E-28	103
TcMYB65	AtMYB98	32.95	173	109	3	7	504	209	379	6.00E-28	102
TcMYB65	AtMYB104	43.56	101	57	0	31	333	18	118	1.00E-27	101
TcMYB65	ATMYB73	44.12	102	57	1	31	336	13	113	2.00E-27	99.8
TcMYB65	ATMYB118	44.44	108	60	1	13	336	183	289	2.00E-27	101
TcMYB65	ATMYB105	43.4	106	59	2	25	339	105	208	2.00E-26	97.4
TcMYB65	AtMYB117	43.27	104	58	2	31	339	98	199	4.00E-26	97.1
TcMYB65	ATMYB44	42.16	102	59	1	31	336	6	106	5.00E-26	95.9
TcMYB65	ATMYB54	42.73	110	62	2	31	357	6	113	4.00E-25	92.4
TcMYB65	ATMYB52	44.66	103	56	2	31	336	5	105	1.00E-24	91.3
TcMYB65	AtMYB100	39.09	110	67	1	13	342	20	128	2.00E-24	90.5
TcMYB65	AtMYB115	38.89	108	66	1	10	333	151	257	3.00E-24	92
TcMYB65	AtMYB89	37.29	118	71	2	7	351	48	164	3.00E-24	88.6
TcMYB65	ATMYB110	38.24	102	63	1	31	336	67	167	1.00E-23	89.7
TcMYB65	ATMYB69	37.84	111	69	1	4	336	10	119	2.00E-23	88.2
TcMYB65	AtMYB56	37.84	111	69	1	31	363	93	202	2.00E-23	89.4
TcMYB65	AtMYB124	40.4	99	59	1	40	336	28	125	1.00E-21	85.1
TcMYB65	ATMYB91	39.36	94	55	1	40	315	7	100	6.00E-21	82.8
TcMYB65	ATMYB88	39.39	99	60	1	40	336	33	130	1.00E-20	82.8
TcMYB65	AtMYB22	34.21	114	75	2	13	354	48	159	5.00E-20	79
TcMYB66	ATMYB13	72.03	118	33	0	1	354	1	118	6.00E-65	194
TcMYB66	ATMYB14	71.79	117	33	0	1	351	1	117	2.00E-64	193
TcMYB66	AtMYB107	70.69	116	34	0	1	348	1	116	3.00E-64	195
TcMYB66	ATMYB16	71.55	116	33	0	1	348	1	116	9.00E-64	194
TcMYB66	ATMYB15	58.55	152	53	1	1	426	1	152	1.00E-63	192
TcMYB66	ATMYB58	69.42	121	37	0	4	366	4	124	2.00E-63	191
TcMYB66	ATMYB63	49.74	193	90	3	4	561	4	188	3.00E-63	192
TcMYB66	AtMYB17	66.93	127	42	0	1	381	1	127	7.00E-63	191
TcMYB66	ATMYB94	48.48	198	92	3	1	564	1	198	1.00E-62	191
TcMYB66	AtMYB9	70.69	116	34	0	1	348	1	116	1.00E-62	191
TcMYB66	AtMYB41	68.1	116	37	0	1	348	1	116	1.00E-61	187
TcMYB66	ATMYB72	69.57	115	35	0	4	348	4	118	8.00E-61	186
TcMYB66	ATMYB106	70.8	113	33	0	10	348	47	159	8.00E-61	188
TcMYB66	ATMYB102	67.24	116	38	0	1	348	1	116	1.00E-60	187
TcMYB66	AtMYB60	60.14	138	55	0	1	414	1	138	1.00E-60	184
TcMYB66	ATMYB30	45.85	205	107	3	1	603	1	205	2.00E-60	186
TcMYB66	ATMYB96	52	175	80	3	1	513	1	174	2.00E-60	186
TcMYB66	AtMYB93	68.97	116	36	0	1	348	1	116	1.00E-59	184
TcMYB66	AtMYB10	71.05	114	33	0	7	348	5	118	3.00E-59	180
TcMYB66	AtMYB74	66.67	117	38	1	1	348	1	117	5.00E-59	182
TcMYB66	MYB8	64.41	118	42	0	1	354	1	118	2.00E-57	174
TcMYB66	AtMYB85	57.14	147	58	1	1	426	1	147	4.00E-57	175
TcMYB66	AtMYB42	49.44	180	88	2	1	531	1	176	7.00E-57	175
TcMYB66	ATMYB92	66.38	116	39	0	1	348	1	116	9.00E-57	176
TcMYB66	AtMYB53	66.38	116	39	0	1	348	1	116	1.00E-56	175
TcMYB66	AtMYB43	65.55	119	41	0	1	357	1	119	1.00E-56	176
TcMYB66	ATMYB31	55.63	151	67	2	1	453	1	145	2.00E-56	176
TcMYB66	AtMYB6	64.66	116	41	0	1	348	1	116	2.00E-56	172
TcMYB66	AtMYB20	63.87	119	43	0	1	357	1	119	2.00E-56	174
TcMYB66	ATMYB3	64.66	116	41	0	1	348	1	116	3.00E-56	172
TcMYB66	MYB7	62.93	116	43	0	1	348	1	116	9.00E-56	172
TcMYB66	ATMYB4	62.71	118	44	0	1	354	1	118	1.00E-55	172
TcMYB66	AtMYB32	63.79	116	42	0	1	348	1	116	1.00E-55	172
TcMYB66	ATMYB12	47.03	185	95	1	1	546	1	185	2.00E-55	174
TcMYB66	ATMYB80	61.34	119	46	0	1	357	1	119	1.00E-54	170
TcMYB66	AtMYB49	62.93	116	43	0	1	348	1	116	2.00E-54	170
TcMYB66	AtMYB36	52.12	165	71	2	1	471	1	165	2.00E-54	170
TcMYB66	ATMYB35	49.7	165	73	1	1	465	1	165	4.00E-54	169
TcMYB66	ATMYB34	63.79	116	42	0	1	348	1	116	4.00E-54	168
TcMYB66	ATMYB111	64.04	114	41	0	1	342	1	114	5.00E-54	169
TcMYB66	ATMYB11	62.6	123	46	0	1	369	1	123	5.00E-54	169
TcMYB66	ATMYB5	63.39	112	41	0	13	348	16	127	1.00E-53	166
TcMYB66	ATMYB122	62.07	116	44	0	1	348	1	116	2.00E-53	168
TcMYB66	ATMYB87	66.67	117	38	1	1	348	1	117	3.00E-53	166
TcMYB66	AtMYB40	54.17	144	66	1	1	432	1	139	6.00E-53	164

TcMYB66	ATMYB84	65.55	119	40	1	1	354	1	119	1.00E-52	165
TcMYB66	ATMYB29	54.68	139	58	1	1	402	1	139	1.00E-52	166
TcMYB66	AtMYB50	45.88	194	90	4	1	537	1	194	2.00E-52	164
TcMYB66	ATMYB68	49.43	174	87	3	1	519	1	170	2.00E-52	166
TcMYB66	ATMYB37	63.25	117	42	1	1	348	1	117	3.00E-52	164
TcMYB66	AtMYB51	61.54	117	44	1	1	348	1	117	6.00E-52	164
TcMYB66	ATMYB61	62.93	116	43	0	1	348	1	116	2.00E-51	163
TcMYB66	ATMYB99	60.66	122	41	1	4	348	3	124	4.00E-51	159
TcMYB66	ATMYB38	64.1	117	41	1	1	348	1	117	5.00E-51	160
TcMYB66	ATMYB86	62.93	116	43	0	1	348	1	116	9.00E-51	161
TcMYB66	ATMYB55	59.38	128	40	1	1	348	1	128	1.00E-50	161
TcMYB66	ATMYB28	60.34	116	46	0	1	348	1	116	2.00E-50	161
TcMYB66	AtMYB103	60.53	114	45	0	1	342	1	114	7.00E-50	159
TcMYB66	ATMYB26	57.6	125	44	1	1	348	1	125	1.00E-49	159
TcMYB66	ATMYB95	61.21	116	45	0	1	348	1	116	2.00E-49	155
TcMYB66	ATMYB67	60.36	111	44	0	16	348	16	126	4.00E-49	156
TcMYB66	AtMYB76	58.62	116	48	0	1	348	1	116	5.00E-49	156
TcMYB66	AtMYB47	58.97	117	48	0	1	351	1	117	1.00E-47	151
TcMYB66	AtMYB79	50.34	149	72	2	37	477	7	145	6.00E-46	146
TcMYB66	ATMYB101	61.54	104	40	0	31	342	17	120	8.00E-46	151
TcMYB66	ATMYB71	41.8	189	110	1	31	597	17	195	1.00E-45	146
TcMYB66	AtMYB81	49.26	136	68	1	25	429	17	152	1.00E-45	150
TcMYB66	AtMYB97	54.62	119	53	1	34	387	19	137	2.00E-45	148
TcMYB66	AtMYB108	60.75	107	42	0	22	342	15	121	3.00E-45	146
TcMYB66	ATMYB120	61.9	105	40	0	34	348	26	130	5.00E-45	150
TcMYB66	ATMYB123	50.38	133	65	1	34	429	14	146	9.00E-45	143
TcMYB66	AtMYB83	59.46	111	45	0	22	354	26	136	2.00E-44	145
TcMYB66	ATMYB33	59.81	107	43	0	22	342	28	134	2.00E-44	148
TcMYB66	AtMYB45	59.8	102	41	0	37	342	19	120	5.00E-44	141
TcMYB66	ATMYB46	47.68	151	73	1	34	468	18	168	6.00E-44	142
TcMYB66	AtMYB112	60.19	103	41	0	34	342	32	134	9.00E-44	140
TcMYB66	ATMYB3	47.95	146	74	2	34	465	20	164	2.00E-43	139
TcMYB66	ATMYB66	56.73	104	45	0	37	348	17	120	2.00E-43	138
TcMYB66	AtMYB24	59.22	103	42	0	34	342	17	119	3.00E-43	138
TcMYB66	ATMYB65	49.62	133	67	1	34	432	41	171	7.00E-43	144
TcMYB66	ATMYB23	56.73	104	45	0	37	348	13	116	7.00E-43	137
TcMYB66	AtMYB114	55.77	104	46	0	31	342	7	110	1.00E-42	134
TcMYB66	AtMYB19	59.8	102	41	0	37	342	13	114	3.00E-42	137
TcMYB66	AtMYB62	36.57	216	123	4	34	639	19	223	4.00E-42	137
TcMYB66	ATMYB2	56.31	103	45	0	34	342	20	122	1.00E-41	135
TcMYB66	AtMYB18	58.82	102	42	0	37	342	11	112	2.00E-41	135
TcMYB66	AtMYB116	54.24	118	52	1	37	384	19	136	2.00E-41	135
TcMYB66	ATMYB57	56.31	103	45	0	34	342	25	127	2.00E-41	133
TcMYB66	ATMYB0	45.39	141	75	1	37	453	15	155	3.00E-41	133
TcMYB66	AtMYB82	50.91	110	54	0	25	354	9	118	8.00E-41	131
TcMYB66	ATMYB121	55.66	106	47	0	25	342	24	129	1.00E-40	133
TcMYB66	ATMYB75	54.81	104	47	0	31	342	7	110	3.00E-40	131
TcMYB66	ATMYB90	54.81	104	47	0	31	342	7	110	3.00E-40	131
TcMYB66	AtMYB113	51.75	114	55	1	1	342	1	110	1.00E-39	129
TcMYB66	ATMYB78	50.41	121	46	1	22	342	22	142	2.00E-39	131
TcMYB66	ATMYB59-3	43.45	145	80	1	37	465	9	153	3.00E-38	125
TcMYB66	AtMYB27	54.37	103	47	0	34	342	9	111	6.00E-37	122
TcMYB66	ATMYB48	52.88	104	49	0	31	342	6	109	2.00E-36	122
TcMYB66	AtMYB104	47.57	103	54	0	25	333	13	115	2.00E-31	111
TcMYB66	ATMYB119	45.54	101	55	1	40	342	105	204	1.00E-29	107
TcMYB66	ATMYB77	48.31	118	58	3	40	384	6	122	2.00E-29	104
TcMYB66	AtMYB1	51.43	105	47	2	40	342	55	154	3.00E-29	105
TcMYB66	AtMYB70	50.5	101	50	1	40	342	13	112	1.00E-28	102
TcMYB66	AtMYB98	43.4	106	60	1	25	342	212	316	1.00E-28	104
TcMYB66	AtMYB98	23.49	149	113	4	169	612	207	342	1.00E-06	40.8
TcMYB66	AtMYB115	37.14	140	87	2	34	450	156	293	2.00E-28	102
TcMYB66	ATMYB44	48.51	101	52	1	40	342	6	105	4.00E-28	101
TcMYB66	AtMYB109	45.11	133	62	3	40	405	56	186	7.00E-28	102
TcMYB66	ATMYB73	48.51	101	52	1	40	342	13	112	1.00E-27	100
TcMYB66	AtMYB64	41.75	103	60	1	34	342	103	204	2.00E-27	101
TcMYB66	ATMYB118	36.77	155	86	4	25	453	184	336	3.00E-27	100
TcMYB66	ATMYB52	46	100	54	1	28	327	1	99	1.00E-26	95.9
TcMYB66	ATMYB54	46.88	96	51	1	40	327	6	100	4.00E-26	94.7
TcMYB66	ATMYB25	34.62	182	115	4	40	573	50	206	5.00E-26	96.7
TcMYB66	ATMYB105	44.55	101	56	1	25	327	102	201	8.00E-26	95.5
TcMYB66	AtMYB117	45.83	96	52	1	40	327	98	192	3.00E-24	91.7
TcMYB66	ATMYB69	43.88	98	55	1	25	318	14	110	6.00E-24	89
TcMYB66	ATMYB91	35.25	139	87	3	49	456	7	143	1.00E-22	87
TcMYB66	AtMYB56	42.71	96	55	1	40	327	93	187	3.00E-22	85.5
TcMYB66	AtMYB100	36.7	109	69	1	25	351	21	128	7.00E-22	83.2
TcMYB66	ATMYB110	40.78	103	59	2	40	342	67	166	4.00E-21	82.4
TcMYB66	AtMYB89	35.29	102	66	1	43	348	57	157	1.00E-19	76.3
TcMYB66	AtMYB22	30.15	136	95	2	25	432	49	180	3.00E-19	76.6
TcMYB66	AtMYB124	38.78	98	60	1	49	342	28	124	8.00E-18	73.9
TcMYB66	ATMYB88	37.76	98	61	1	49	342	33	129	2.00E-17	72.8

TcMYB67	ATMYB86	51.67	209	98	4	13	630	5	206	4.00E-64	197
TcMYB67	AtMYB50	61.44	153	59	2	13	471	5	151	3.00E-62	191
TcMYB67	ATMYB61	50.52	194	80	3	13	546	5	195	6.00E-61	189
TcMYB67	ATMYB67	48.69	191	98	3	16	588	16	189	2.00E-58	181
TcMYB67	ATMYB55	67.42	132	31	2	13	372	5	135	2.00E-56	177
TcMYB67	AtMYB103	68.14	113	36	1	13	351	5	116	3.00E-55	175
TcMYB67	AtMYB85	62.9	124	46	1	16	387	6	128	4.00E-55	171
TcMYB67	AtMYB42	62.9	124	46	1	16	387	6	128	6.00E-54	169
TcMYB67	AtMYB32	57.25	131	56	1	16	408	6	135	8.00E-54	168
TcMYB67	AtMYB6	58.33	132	54	2	16	408	6	136	1.00E-53	167
TcMYB67	ATMYB106	41.35	237	119	4	1	651	44	279	1.00E-53	171
TcMYB67	ATMYB80	62.1	124	47	1	16	387	6	128	4.00E-53	168
TcMYB67	ATMYB4	58.02	131	55	1	16	408	6	135	4.00E-53	167
TcMYB67	ATMYB92	54.19	155	65	3	37	483	13	166	6.00E-53	168
TcMYB67	ATMYB3	43.78	201	112	3	16	615	6	199	8.00E-53	165
TcMYB67	AtMYB20	62.1	124	47	1	16	387	6	128	8.00E-53	166
TcMYB67	AtMYB93	61.07	131	51	1	16	408	6	135	9.00E-53	168
TcMYB67	ATMYB26	65.85	123	33	2	13	354	5	126	9.00E-53	168
TcMYB67	AtMYB43	61.07	131	51	1	16	408	6	135	9.00E-53	167
TcMYB67	ATMYB102	62.1	124	47	1	16	387	6	128	2.00E-52	167
TcMYB67	ATMYB46	67.24	116	38	1	4	351	8	122	3.00E-52	164
TcMYB67	MYB7	52.05	146	70	1	16	453	6	150	3.00E-52	164
TcMYB67	ATMYB16	58.78	131	54	1	16	408	6	135	4.00E-52	166
TcMYB67	AtMYB53	65.81	117	40	1	37	387	13	128	1.00E-51	164
TcMYB67	AtMYB107	60.48	124	49	1	16	387	6	128	1.00E-51	164
TcMYB67	AtMYB74	60	125	49	2	16	387	6	129	2.00E-51	164
TcMYB67	ATMYB5	56.3	135	59	2	16	420	17	147	8.00E-51	160
TcMYB67	AtMYB17	59.68	124	50	1	16	387	6	128	2.00E-50	160
TcMYB67	MYB8	58.82	119	49	1	16	372	6	123	2.00E-50	158
TcMYB67	AtMYB9	60.48	124	49	1	16	387	6	128	2.00E-50	161
TcMYB67	ATMYB35	58.87	124	51	1	16	387	6	128	7.00E-50	159
TcMYB67	AtMYB83	65.22	115	40	1	7	351	21	134	1.00E-49	159
TcMYB67	AtMYB49	60	125	50	1	13	387	5	128	1.00E-49	159
TcMYB67	AtMYB41	58.06	124	52	1	16	387	6	128	2.00E-49	157
TcMYB67	ATMYB99	47.16	176	87	2	10	519	5	178	2.00E-49	156
TcMYB67	AtMYB10	50	160	78	3	16	489	8	157	4.00E-49	155
TcMYB67	ATMYB72	65.18	112	39	1	16	351	8	118	2.00E-48	155
TcMYB67	ATMYB111	55.26	152	60	4	16	447	6	156	4.00E-48	155
TcMYB67	AtMYB19	62.96	108	40	1	25	348	9	115	5.00E-48	153
TcMYB67	AtMYB40	47.4	173	87	4	10	516	4	173	6.00E-48	153
TcMYB67	ATMYB12	55	140	63	2	16	435	6	139	1.00E-47	155
TcMYB67	ATMYB29	56.35	126	55	1	10	387	4	128	2.00E-47	154
TcMYB67	ATMYB11	58.06	124	52	2	16	387	6	123	2.00E-47	154
TcMYB67	AtMYB76	40.47	215	127	4	10	651	4	201	3.00E-47	153
TcMYB67	ATMYB95	60.66	122	48	1	37	402	13	133	3.00E-47	151
TcMYB67	ATMYB58	63.39	112	41	1	16	351	8	118	4.00E-47	151
TcMYB67	ATMYB37	60.17	118	47	0	16	369	6	123	6.00E-47	152
TcMYB67	ATMYB28	54.96	131	59	1	10	402	4	133	7.00E-47	153
TcMYB67	ATMYB34	53.79	132	61	1	1	396	1	131	1.00E-46	150
TcMYB67	ATMYB84	60.18	113	45	0	16	354	6	118	2.00E-46	150
TcMYB67	ATMYB68	59.29	113	46	0	16	354	6	118	5.00E-46	151
TcMYB67	AtMYB51	57.6	125	52	2	1	372	1	124	6.00E-46	150
TcMYB67	ATMYB123	64.55	110	39	1	22	351	10	118	6.00E-46	147
TcMYB67	ATMYB13	61.4	114	44	1	10	351	4	116	6.00E-46	147
TcMYB67	ATMYB14	44.39	187	104	5	16	576	6	169	1.00E-45	146
TcMYB67	ATMYB38	60.18	113	45	0	16	354	6	118	2.00E-45	147
TcMYB67	AtMYB18	39.11	248	144	9	28	750	8	238	3.00E-45	146
TcMYB67	AtMYB36	58.41	113	47	0	16	354	6	118	6.00E-45	147
TcMYB67	ATMYB63	61.61	112	43	1	16	351	8	118	6.00E-45	146
TcMYB67	AtMYB45	58.04	112	47	1	10	345	10	120	7.00E-45	145
TcMYB67	ATMYB122	35.84	279	157	6	1	771	1	278	1.00E-44	146
TcMYB67	ATMYB15	36.71	237	148	6	16	720	6	217	4.00E-44	144
TcMYB67	AtMYB47	59.32	118	47	2	37	387	13	128	5.00E-44	143
TcMYB67	ATMYB23	55.45	110	49	1	22	351	8	116	6.00E-43	139
TcMYB67	AtMYB82	59.62	104	42	1	37	348	13	115	1.00E-42	137
TcMYB67	ATMYB31	46.41	153	82	2	16	474	6	154	2.00E-42	140
TcMYB67	ATMYB66	57.14	105	45	1	37	351	17	120	2.00E-42	137
TcMYB67	AtMYB79	61.54	104	40	1	37	348	7	109	1.00E-41	137
TcMYB67	ATMYB121	58.33	108	45	1	22	345	23	129	1.00E-41	137
TcMYB67	ATMYB87	54.87	113	51	0	16	354	6	118	1.00E-41	138
TcMYB67	ATMYB71	61.54	104	40	1	37	348	19	121	2.00E-41	136
TcMYB67	ATMYB94	51.15	131	64	2	16	408	6	126	2.00E-41	138
TcMYB67	AtMYB108	62.14	103	39	1	37	345	20	121	4.00E-41	137
TcMYB67	ATMYB96	50.7	142	70	3	16	441	6	139	6.00E-41	137
TcMYB67	ATMYB30	50	136	68	1	1	408	1	135	6.00E-41	136
TcMYB67	ATMYB0	52.25	111	53	1	22	354	10	119	6.00E-41	134
TcMYB67	AtMYB60	51.15	131	64	1	16	408	6	135	9.00E-40	132
TcMYB67	AtMYB112	59.22	103	42	1	37	345	33	134	2.00E-39	130
TcMYB67	AtMYB24	57.8	109	46	1	37	363	18	125	4.00E-39	129
TcMYB67	ATMYB3	42.86	175	100	4	37	561	21	177	1.00E-38	128

TcMYB67	AtMYB116	40.24	169	101	3	7	513	9	161	3.00E-38	128
TcMYB67	ATMYB57	55.24	105	47	1	37	351	26	129	9.00E-38	125
TcMYB67	ATMYB65	51.75	114	55	1	7	348	32	144	5.00E-37	130
TcMYB67	ATMYB33	53.51	114	53	1	7	348	23	135	7.00E-37	129
TcMYB67	AtMYB62	49.14	116	59	1	22	369	15	129	1.00E-36	124
TcMYB67	AtMYB114	52.88	104	49	1	37	348	9	111	2.00E-36	119
TcMYB67	ATMYB59-3	50.93	108	53	1	25	348	5	111	4.00E-36	121
TcMYB67	ATMYB48	50.46	109	54	1	22	348	3	110	5.00E-36	122
TcMYB67	ATMYB101	53.85	104	48	1	37	348	19	121	7.00E-36	126
TcMYB67	ATMYB120	55.24	105	47	1	37	351	27	130	9.00E-36	126
TcMYB67	AtMYB27	40.23	174	99	4	22	528	5	169	3.00E-35	119
TcMYB67	ATMYB75	52.88	104	49	1	37	348	9	111	3.00E-35	119
TcMYB67	AtMYB81	54.9	102	46	1	40	345	22	122	1.00E-34	122
TcMYB67	ATMYB90	52.88	104	49	1	37	348	9	111	2.00E-34	117
TcMYB67	AtMYB97	52.43	103	49	1	37	345	20	121	1.00E-33	119
TcMYB67	AtMYB113	50.96	104	51	1	37	348	9	111	1.00E-33	115
TcMYB67	ATMYB78	47.54	122	50	2	22	345	22	142	2.00E-33	116
TcMYB67	ATMYB2	48.08	104	54	1	37	348	21	123	5.00E-33	114
TcMYB67	AtMYB104	50.53	95	47	1	49	333	21	114	6.00E-30	108
TcMYB67	ATMYB105	44.19	129	70	3	4	384	93	217	7.00E-27	99.4
TcMYB67	AtMYB1	36.56	186	117	6	40	594	55	215	8.00E-27	100
TcMYB67	ATMYB25	50	104	51	3	40	348	50	150	6.00E-26	97.4
TcMYB67	ATMYB73	50	104	51	3	40	348	13	113	6.00E-26	96.7
TcMYB67	AtMYB64	41.03	117	66	2	7	348	91	205	1.00E-25	97.1
TcMYB67	AtMYB70	49.04	104	52	3	40	348	13	113	1.00E-25	95.5
TcMYB67	ATMYB119	42.72	103	59	1	40	348	105	205	4.00E-25	95.9
TcMYB67	AtMYB109	50	104	51	3	40	348	56	156	4.00E-25	95.5
TcMYB67	ATMYB44	47.12	104	54	3	40	348	6	106	5.00E-25	94
TcMYB67	ATMYB54	48.98	98	49	3	40	330	6	100	6.00E-25	92.4
TcMYB67	AtMYB117	45.63	103	56	1	40	348	98	198	1.00E-24	93.6
TcMYB67	ATMYB118	44.23	104	57	3	40	348	189	289	7.00E-24	92.4
TcMYB67	ATMYB52	31.12	196	124	5	40	594	5	192	7.00E-24	89.7
TcMYB67	AtMYB56	47.42	97	51	2	40	330	93	187	9.00E-24	90.9
TcMYB67	ATMYB77	46.15	104	55	3	40	348	6	106	1.00E-23	90.1
TcMYB67	AtMYB115	42.86	98	56	2	40	333	158	253	9.00E-23	88.6
TcMYB67	ATMYB110	38.71	124	76	2	40	411	67	187	1.00E-22	87.4
TcMYB67	AtMYB98	38.46	117	72	2	40	390	217	331	2.00E-22	88.2
TcMYB67	AtMYB100	46.32	95	51	2	49	333	29	121	4.00E-22	84.7
TcMYB67	ATMYB69	42.42	99	56	3	37	330	18	113	3.00E-19	77.4
TcMYB67	AtMYB89	35.65	115	71	3	10	345	43	155	2.00E-17	70.9
TcMYB67	AtMYB22	38.54	96	58	3	49	333	57	148	1.00E-16	70.1
TcMYB67	ATMYB88	37	100	63	2	49	348	33	130	3.00E-16	70.5
TcMYB67	ATMYB91	33.96	106	68	2	37	348	3	107	9.00E-16	68.6
TcMYB67	AtMYB124	36	100	64	2	49	348	28	125	4.00E-15	67
TcMYB68	ATMYB105	69.23	130	40	1	70	459	100	225	2.00E-62	191
TcMYB68	ATMYB52	75.89	112	27	1	91	426	5	112	2.00E-62	188
TcMYB68	AtMYB117	68.66	134	40	2	91	486	98	227	9.00E-62	190
TcMYB68	ATMYB54	72.97	111	30	1	91	423	6	112	3.00E-59	180
TcMYB68	ATMYB69	49.73	183	88	5	22	558	2	167	3.00E-56	173
TcMYB68	AtMYB56	71.17	111	32	1	91	423	93	199	1.00E-54	171
TcMYB68	ATMYB110	61.42	127	49	1	58	438	56	178	2.00E-52	164
TcMYB68	AtMYB89	52.03	123	59	2	52	420	46	161	3.00E-40	130
TcMYB68	AtMYB109	50.89	112	55	1	79	414	52	159	1.00E-38	131
TcMYB68	AtMYB1	51.82	110	53	1	79	408	51	156	2.00E-37	127
TcMYB68	ATMYB25	49.56	113	57	1	88	426	49	157	4.00E-36	124
TcMYB68	ATMYB73	50	106	53	1	88	405	12	113	2.00E-35	121
TcMYB68	ATMYB44	51.89	106	51	1	88	405	5	106	2.00E-35	120
TcMYB68	AtMYB70	49.06	106	54	1	88	405	12	113	3.00E-35	120
TcMYB68	ATMYB77	49.06	106	54	1	88	405	5	106	1.00E-33	116
TcMYB68	ATMYB119	47.15	123	65	2	76	444	100	215	4.00E-33	117
TcMYB68	ATMYB80	37.82	156	95	3	67	528	6	158	1.00E-31	111
TcMYB68	AtMYB64	45.83	120	65	2	79	438	101	213	2.00E-30	109
TcMYB68	ATMYB23	45.05	111	61	2	73	405	8	115	2.00E-30	105
TcMYB68	ATMYB67	43.41	129	73	3	19	405	3	125	3.00E-30	107
TcMYB68	AtMYB10	42.37	118	68	2	67	420	8	122	7.00E-30	104
TcMYB68	ATMYB66	44.55	110	61	2	76	405	13	119	1.00E-29	103
TcMYB68	ATMYB0	44.14	111	62	2	73	405	10	117	3.00E-29	102
TcMYB68	AtMYB49	45.69	116	63	2	58	405	3	115	3.00E-29	104
TcMYB68	AtMYB81	42.11	133	77	2	70	468	15	144	3.00E-29	106
TcMYB68	AtMYB51	43.36	113	64	2	67	405	7	116	5.00E-29	104
TcMYB68	ATMYB72	42.37	118	68	2	67	420	8	122	8.00E-29	103
TcMYB68	AtMYB53	44.64	112	62	2	70	405	7	115	1.00E-28	103
TcMYB68	MYB8	39.32	117	71	1	67	417	6	119	2.00E-28	100
TcMYB68	ATMYB63	33.33	186	124	4	67	624	8	170	3.00E-28	101
TcMYB68	AtMYB41	43.36	113	64	2	67	405	6	115	3.00E-28	101
TcMYB68	ATMYB118	40.68	118	70	1	70	423	182	295	3.00E-28	103
TcMYB68	ATMYB15	41.38	116	68	2	67	414	6	118	5.00E-28	100
TcMYB68	ATMYB3	41.88	117	68	2	67	417	6	119	6.00E-28	100
TcMYB68	AtMYB107	43.36	113	64	2	67	405	6	115	9.00E-28	100
TcMYB68	AtMYB103	39.69	131	79	3	49	441	3	127	9.00E-28	101

TcMYB68	ATMYB122	43.93	107	60	2	67	387	6	109	1.00E-27	100
TcMYB68	ATMYB4	42.74	117	67	2	67	417	6	119	2.00E-27	99
TcMYB68	AtMYB45	48.48	99	51	1	91	387	20	115	3.00E-27	98.2
TcMYB68	AtMYB115	40.54	111	66	1	91	423	158	264	4.00E-27	99.8
TcMYB68	ATMYB92	44.64	112	62	2	70	405	7	115	4.00E-27	99.4
TcMYB68	AtMYB9	42.48	113	65	2	67	405	6	115	4.00E-27	99.4
TcMYB68	AtMYB18	43.4	106	60	1	91	408	12	114	5.00E-27	98.2
TcMYB68	AtMYB98	35.29	136	88	2	10	417	193	321	8.00E-27	99.8
TcMYB68	ATMYB101	43.7	119	67	2	73	429	14	129	8.00E-27	100
TcMYB68	AtMYB74	43.86	114	63	3	67	405	6	116	9.00E-27	98.2
TcMYB68	ATMYB86	39.17	120	73	2	58	417	3	119	1.00E-26	98.6
TcMYB68	MYB7	39.82	113	68	1	67	405	6	115	1.00E-26	97.1
TcMYB68	ATMYB34	38.46	117	72	2	67	417	6	119	1.00E-26	97.4
TcMYB68	ATMYB35	42.48	113	65	2	67	405	6	115	1.00E-26	97.8
TcMYB68	ATMYB33	36.43	140	83	2	28	429	7	143	1.00E-26	99.8
TcMYB68	AtMYB6	41.59	113	66	2	67	405	6	115	1.00E-26	95.9
TcMYB68	AtMYB60	34.42	154	101	3	67	528	6	153	2.00E-26	96.7
TcMYB68	AtMYB40	39.83	118	71	2	58	411	3	117	2.00E-26	96.3
TcMYB68	ATMYB102	41.88	117	68	2	55	405	2	115	2.00E-26	97.8
TcMYB68	AtMYB27	33.78	148	98	2	91	534	11	153	2.00E-26	95.5
TcMYB68	AtMYB19	40.57	106	63	1	91	408	14	116	4.00E-26	95.5
TcMYB68	ATMYB106	39.82	113	68	2	67	405	49	158	4.00E-26	97.4
TcMYB68	ATMYB61	39.17	120	73	2	58	417	3	119	4.00E-26	97.1
TcMYB68	ATMYB28	28.42	190	134	5	55	618	2	181	4.00E-26	97.1
TcMYB68	AtMYB104	35.77	123	79	2	55	423	6	122	5.00E-26	97.1
TcMYB68	AtMYB82	42.2	109	63	1	91	417	14	119	5.00E-26	93.6
TcMYB68	AtMYB32	36.62	142	90	3	67	492	6	140	6.00E-26	95.1
TcMYB68	AtMYB17	35.06	174	108	6	67	573	6	171	6.00E-26	95.5
TcMYB68	ATMYB13	42.48	113	65	2	67	405	6	115	7.00E-26	94.4
TcMYB68	ATMYB14	37.31	134	84	2	67	468	6	136	7.00E-26	94.4
TcMYB68	ATMYB95	42.2	109	63	2	91	417	14	119	8.00E-26	94.7
TcMYB68	AtMYB97	41.59	113	66	1	91	429	21	130	1.00E-25	96.3
TcMYB68	AtMYB93	40.71	113	67	2	67	405	6	115	1.00E-25	95.9
TcMYB68	AtMYB50	37.5	120	75	2	58	417	3	119	1.00E-25	95.1
TcMYB68	ATMYB48	38.76	129	79	2	91	477	9	134	2.00E-25	93.6
TcMYB68	ATMYB94	39.66	116	70	1	67	414	6	118	2.00E-25	94.7
TcMYB68	ATMYB120	43.36	113	64	2	91	429	28	137	2.00E-25	96.3
TcMYB68	ATMYB58	37.5	120	75	2	67	426	8	124	2.00E-25	93.6
TcMYB68	ATMYB96	39.66	116	70	1	67	414	6	118	2.00E-25	94.7
TcMYB68	ATMYB16	38.94	113	69	2	67	405	6	115	2.00E-25	94.4
TcMYB68	ATMYB65	38.94	113	69	1	91	429	43	152	3.00E-25	95.9
TcMYB68	ATMYB121	35.77	137	88	2	34	444	10	143	3.00E-25	93.2
TcMYB68	ATMYB31	39.82	113	68	1	67	405	6	115	4.00E-25	94
TcMYB68	AtMYB100	41.67	108	62	3	97	417	28	130	4.00E-25	92
TcMYB68	ATMYB26	37.6	125	72	2	49	405	3	124	4.00E-25	94.4
TcMYB68	ATMYB59-3	40.71	113	67	2	85	423	8	117	6.00E-25	91.7
TcMYB68	ATMYB30	37.93	116	72	1	67	414	6	118	6.00E-25	93.2
TcMYB68	ATMYB5	38.05	113	70	1	67	405	17	126	7.00E-25	91.7
TcMYB68	ATMYB29	39.64	111	67	2	55	387	2	109	8.00E-25	93.2
TcMYB68	ATMYB111	31.79	173	118	3	67	585	6	168	8.00E-25	93.2
TcMYB68	AtMYB36	32.12	165	111	4	67	558	6	150	1.00E-24	92.8
TcMYB68	AtMYB47	39.45	109	66	2	91	417	14	119	1.00E-24	91.7
TcMYB68	ATMYB123	39.66	116	70	2	91	438	16	128	1.00E-24	91.3
TcMYB68	AtMYB76	41.9	105	61	2	91	405	14	115	2.00E-24	92
TcMYB68	AtMYB79	40.8	125	74	3	91	465	8	128	3.00E-24	90.1
TcMYB68	ATMYB2	40.17	117	70	2	34	384	8	116	4.00E-24	90.1
TcMYB68	ATMYB55	35.11	131	74	2	58	417	3	131	4.00E-24	91.3
TcMYB68	AtMYB43	37.82	119	74	2	58	414	3	118	7.00E-24	90.5
TcMYB68	ATMYB46	39.09	110	67	2	91	420	20	126	9.00E-24	89.4
TcMYB68	AtMYB62	40	115	69	2	70	414	14	125	1.00E-23	89.4
TcMYB68	AtMYB114	39.09	110	67	2	76	405	5	111	1.00E-23	85.5
TcMYB68	AtMYB83	34.11	129	85	1	34	420	13	138	1.00E-23	89.7
TcMYB68	AtMYB20	36.97	119	75	2	58	414	3	118	2.00E-23	88.6
TcMYB68	AtMYB116	36.51	126	77	2	46	414	2	124	3.00E-23	88.2
TcMYB68	ATMYB84	37.5	120	74	3	67	423	6	122	4.00E-23	88.2
TcMYB68	ATMYB75	36.89	122	77	2	76	441	5	123	4.00E-23	87
TcMYB68	ATMYB71	38.4	125	77	3	91	465	20	140	6.00E-23	87
TcMYB68	ATMYB12	38.94	113	69	2	67	405	6	115	1.00E-22	87.8
TcMYB68	AtMYB42	37.93	116	72	2	58	405	3	115	1.00E-22	86.7
TcMYB68	ATMYB68	35.83	120	76	2	67	423	6	122	2.00E-22	87
TcMYB68	AtMYB22	32.59	135	90	3	1	402	23	152	2.00E-22	85.1
TcMYB68	ATMYB37	36.44	118	75	2	67	420	6	121	2.00E-22	86.3
TcMYB68	AtMYB85	37.93	116	72	2	58	405	3	115	3.00E-22	85.1
TcMYB68	AtMYB108	38.32	107	66	1	64	384	12	115	4.00E-22	85.5
TcMYB68	ATMYB38	37.72	114	70	2	67	405	6	116	4.00E-22	85.1
TcMYB68	ATMYB87	28.57	189	129	6	67	615	6	183	6.00E-22	84.7
TcMYB68	AtMYB112	41.84	98	57	1	91	384	34	128	7.00E-22	83.6
TcMYB68	ATMYB57	37.27	110	69	1	91	420	27	133	2.00E-21	81.6
TcMYB68	ATMYB11	37.17	113	71	2	67	405	6	115	3.00E-21	83.6
TcMYB68	ATMYB90	35.88	131	84	3	76	468	5	131	4.00E-21	81.6

TcMYB68	AtMYB113	38.53	109	67	2	91	417	10	115	5.00E-21	81.3
TcMYB68	ATMYB99	37.4	123	70	3	58	405	4	123	1.00E-20	80.5
TcMYB68	AtMYB24	33.8	142	91	5	91	507	19	154	3.00E-20	78.6
TcMYB68	ATMYB3	35.19	108	70	1	91	414	22	126	7.00E-20	77.8
TcMYB68	ATMYB88	32.52	123	83	2	52	420	19	135	1.00E-19	79.3
TcMYB68	AtMYB124	32.61	138	93	3	7	420	2	130	2.00E-19	78.6
TcMYB68	ATMYB78	33.58	134	75	3	25	384	8	136	3.00E-19	77.4
TcMYB68	ATMYB91	35.19	108	70	1	91	414	4	110	2.00E-18	75.9
TcMYB69	AtMYB82	88.57	35	4	0	1	105	81	115	4.00E-19	75.9
TcMYB69	ATMYB5	58.46	65	27	1	1	195	92	153	5.00E-19	76.6
TcMYB69	ATMYB90	56.06	66	23	1	1	180	77	142	2.00E-18	75.1
TcMYB69	ATMYB0	74.42	43	11	0	1	129	83	125	2.00E-18	74.7
TcMYB69	ATMYB23	66.67	45	15	0	1	135	81	125	3.00E-18	73.9
TcMYB69	AtMYB114	82.86	35	6	0	1	105	77	111	4.00E-18	71.6
TcMYB69	ATMYB66	76.32	38	9	0	1	114	85	122	9.00E-18	72.4
TcMYB69	ATMYB3	39.47	114	68	2	1	339	81	191	1.00E-17	73.2
TcMYB69	ATMYB75	85.71	35	5	0	1	105	77	111	1.00E-17	72.8
TcMYB69	AtMYB20	32.52	163	108	4	1	483	81	241	1.00E-17	73.2
TcMYB69	AtMYB6	80.56	36	7	0	1	108	81	116	2.00E-17	72
TcMYB69	AtMYB113	82.86	35	6	0	1	105	77	111	3.00E-17	71.6
TcMYB69	ATMYB4	80.56	36	7	0	1	108	81	116	5.00E-17	71.6
TcMYB69	AtMYB32	48.19	83	26	2	1	198	81	163	8.00E-17	70.9
TcMYB69	AtMYB42	35	120	78	2	1	360	81	195	1.00E-16	70.5
TcMYB69	MYB8	75	36	9	0	1	108	81	116	1.00E-16	69.3
TcMYB69	AtMYB85	31.29	147	91	3	1	411	81	220	1.00E-16	70.1
TcMYB69	MYB7	77.78	36	8	0	1	108	81	116	2.00E-16	69.7
TcMYB69	ATMYB12	80.56	36	7	0	1	108	81	116	3.00E-16	70.1
TcMYB69	AtMYB9	80.56	36	7	0	1	108	81	116	5.00E-16	69.3
TcMYB69	AtMYB107	77.78	36	8	0	1	108	81	116	8.00E-16	68.6
TcMYB69	ATMYB123	77.78	36	8	0	1	108	83	118	9.00E-16	67.8
TcMYB69	ATMYB111	70.73	41	12	0	1	123	81	121	9.00E-16	68.6
TcMYB69	AtMYB49	77.78	36	8	0	1	108	81	116	1.00E-15	67.8
TcMYB69	AtMYB41	28.17	213	108	8	1	504	81	281	3.00E-15	66.6
TcMYB69	ATMYB72	64.44	45	16	0	4	138	84	128	4.00E-15	66.2
TcMYB69	AtMYB53	72.22	36	10	0	1	108	81	116	5.00E-15	66.2
TcMYB69	AtMYB74	69.44	36	11	0	1	108	82	117	7.00E-15	65.9
TcMYB69	ATMYB37	34.06	138	85	4	13	408	86	207	7.00E-15	65.9
TcMYB69	AtMYB17	75	36	9	0	1	108	81	116	8.00E-15	65.5
TcMYB69	ATMYB92	72.22	36	10	0	1	108	81	116	1.00E-14	65.5
TcMYB69	ATMYB102	72.22	36	10	0	1	108	81	116	1.00E-14	65.5
TcMYB69	ATMYB13	72.22	36	10	0	1	108	81	116	1.00E-14	64.3
TcMYB69	ATMYB80	72.22	36	10	0	4	111	82	117	2.00E-14	64.7
TcMYB69	ATMYB46	28.79	198	140	6	4	594	88	243	2.00E-14	64.3
TcMYB69	ATMYB63	40.96	83	49	1	1	249	83	151	2.00E-14	64.3
TcMYB69	ATMYB16	75	36	9	0	1	108	81	116	3.00E-14	63.9
TcMYB69	ATMYB31	73.68	38	10	0	1	114	81	118	3.00E-14	63.9
TcMYB69	ATMYB15	69.44	36	11	0	1	108	81	116	3.00E-14	63.5
TcMYB69	ATMYB61	38.37	86	53	1	1	258	81	163	4.00E-14	63.9
TcMYB69	ATMYB106	75	36	9	0	1	108	124	159	4.00E-14	63.9
TcMYB69	AtMYB43	72.22	36	10	0	1	108	81	116	4.00E-14	63.5
TcMYB69	ATMYB11	72.22	36	10	0	1	108	81	116	5.00E-14	63.5
TcMYB69	ATMYB99	29.88	164	108	5	1	471	89	234	6.00E-14	62.4
TcMYB69	ATMYB14	59.52	42	17	0	1	126	81	122	6.00E-14	62.4
TcMYB69	ATMYB87	38.37	86	53	1	13	270	86	165	7.00E-14	62.8
TcMYB69	ATMYB67	26.63	169	98	4	1	429	91	257	1.00E-13	62.4
TcMYB69	ATMYB94	66.67	42	14	0	1	126	81	122	1.00E-13	62.4
TcMYB69	AtMYB60	68.29	41	13	0	1	123	81	121	1.00E-13	62
TcMYB69	ATMYB71	62.79	43	16	0	1	129	87	129	1.00E-13	61.6
TcMYB69	ATMYB30	75	36	9	0	1	108	81	116	1.00E-13	62
TcMYB69	ATMYB96	75	36	9	0	1	108	81	116	2.00E-13	62
TcMYB69	ATMYB58	58.54	41	17	0	1	123	83	123	2.00E-13	61.2
TcMYB69	AtMYB51	69.44	36	11	0	1	108	82	117	2.00E-13	61.6
TcMYB69	AtMYB93	69.44	36	11	0	1	108	81	116	2.00E-13	61.6
TcMYB69	AtMYB79	60.47	43	17	0	1	129	75	117	2.00E-13	60.8
TcMYB69	AtMYB76	35.35	99	59	3	1	282	81	178	3.00E-13	61.2
TcMYB69	AtMYB83	59.52	42	17	0	1	126	99	140	3.00E-13	61.2
TcMYB69	AtMYB27	71.43	35	10	0	1	105	78	112	4.00E-13	60.1
TcMYB69	ATMYB55	36.78	87	55	1	1	261	93	162	4.00E-13	60.8
TcMYB69	AtMYB10	53.06	49	20	1	1	138	83	131	5.00E-13	59.7
TcMYB69	ATMYB86	72.22	36	10	0	1	108	81	116	5.00E-13	60.5
TcMYB69	ATMYB26	66.67	36	12	0	1	108	90	125	5.00E-13	60.5
TcMYB69	ATMYB29	25.1	255	164	8	1	684	81	324	7.00E-13	60.1
TcMYB69	ATMYB34	47.76	67	35	2	1	201	81	144	7.00E-13	59.7
TcMYB69	AtMYB50	69.44	36	11	0	1	108	81	116	8.00E-13	59.7
TcMYB69	ATMYB59-3	68.57	35	11	0	1	105	77	111	9.00E-13	58.9
TcMYB69	ATMYB28	42.47	73	42	1	1	219	81	150	1.00E-12	59.7
TcMYB69	AtMYB103	66.67	36	12	0	1	108	81	116	1.00E-12	59.7
TcMYB69	ATMYB48	68.57	35	11	0	1	105	76	110	1.00E-12	58.9
TcMYB69	AtMYB45	37.63	93	57	2	1	276	87	176	1.00E-12	58.9
TcMYB69	AtMYB18	45.59	68	37	0	4	207	80	147	1.00E-12	58.9

TcMYB69	ATMYB84	63.89	36	13	0	1	108	82	117	1.00E-12	58.9
TcMYB69	ATMYB35	65.71	35	12	0	4	108	82	116	2.00E-12	58.5
TcMYB69	ATMYB68	71.88	32	9	0	13	108	86	117	2.00E-12	58.5
TcMYB69	ATMYB95	26.04	169	117	3	4	486	82	250	3.00E-12	57.8
TcMYB69	AtMYB36	71.88	32	9	0	13	108	86	117	3.00E-12	58.2
TcMYB69	ATMYB120	66.67	36	12	0	1	108	95	130	3.00E-12	58.5
TcMYB69	AtMYB97	67.65	34	11	0	1	102	88	121	5.00E-12	57.8
TcMYB69	ATMYB101	62.86	35	13	0	1	105	87	121	5.00E-12	57.8
TcMYB69	ATMYB33	62.86	35	13	0	1	105	101	135	5.00E-12	57.8
TcMYB69	AtMYB81	67.65	34	11	0	1	102	89	122	7.00E-12	57.4
TcMYB69	ATMYB121	60	40	16	0	1	120	96	135	7.00E-12	56.6
TcMYB69	ATMYB65	62.86	35	13	0	1	105	110	144	1.00E-11	57
TcMYB69	ATMYB57	52.38	42	20	0	1	126	94	135	1.00E-11	55.5
TcMYB69	AtMYB19	62.86	35	13	0	1	105	81	115	2.00E-11	55.1
TcMYB69	ATMYB2	65.71	35	12	0	1	105	89	123	2.00E-11	55.1
TcMYB69	AtMYB40	61.11	36	14	0	1	108	81	116	3.00E-11	54.7
TcMYB69	AtMYB116	27.93	179	95	8	1	435	87	260	3.00E-11	54.7
TcMYB69	ATMYB122	58.33	36	15	0	1	108	81	116	4.00E-11	54.7
TcMYB69	ATMYB38	68.75	32	10	0	13	108	86	117	5.00E-11	54.3
TcMYB69	AtMYB24	28.8	125	81	3	1	351	86	206	7.00E-11	53.1
TcMYB69	ATMYB3	56.41	39	17	0	1	117	89	127	8.00E-11	53.1
TcMYB69	AtMYB112	64.71	34	12	0	1	102	101	134	9.00E-11	53.1
TcMYB69	ATMYB110	32.91	79	53	1	4	240	134	211	9.00E-11	53.5
TcMYB69	ATMYB105	51.22	41	20	0	4	126	174	214	1.00E-10	53.5
TcMYB69	ATMYB25	39.13	69	34	1	10	192	119	187	1.00E-10	53.1
TcMYB69	AtMYB62	62.86	35	13	0	1	105	88	122	2.00E-10	52.8
TcMYB69	AtMYB108	64.71	34	12	0	1	102	88	121	2.00E-10	52.4
TcMYB69	AtMYB47	54.05	37	17	0	1	111	81	117	3.00E-10	52
TcMYB69	AtMYB117	48.78	41	21	0	4	126	165	205	5.00E-10	51.6
TcMYB69	ATMYB54	30.67	75	52	0	4	228	73	147	7.00E-10	50.4
TcMYB69	AtMYB1	58.82	34	14	0	4	105	122	155	2.00E-09	50.1
TcMYB69	AtMYB104	62.07	29	11	0	1	87	85	113	3.00E-09	49.3
TcMYB69	AtMYB109	58.33	36	15	0	4	111	123	158	4.00E-09	48.9
TcMYB69	AtMYB98	67.86	28	9	0	4	87	284	311	4.00E-09	48.9
TcMYB69	ATMYB73	28.57	126	82	1	4	357	80	205	5.00E-09	48.5
TcMYB69	ATMYB118	67.86	28	9	0	4	87	256	283	6.00E-09	48.5
TcMYB69	AtMYB100	62.07	29	11	0	4	90	93	121	6.00E-09	47.8
TcMYB69	ATMYB52	50	34	17	0	4	105	72	105	8.00E-09	47.4
TcMYB69	AtMYB56	50	34	17	0	4	105	160	193	1.00E-08	47.4
TcMYB69	AtMYB70	58.82	34	14	0	4	105	80	113	1.00E-08	47
TcMYB69	ATMYB44	55.88	34	15	0	4	105	73	106	2.00E-08	46.6
TcMYB69	AtMYB22	27.18	103	74	4	1	306	119	209	2.00E-08	46.2
TcMYB69	AtMYB115	42.86	49	26	1	1	141	224	272	2.00E-08	46.6
TcMYB69	ATMYB78	67.86	28	9	0	19	102	115	142	3.00E-08	46.2
TcMYB69	ATMYB69	44.44	45	25	0	4	138	86	130	4.00E-08	45.4
TcMYB69	ATMYB77	60	30	12	0	16	105	77	106	8.00E-08	44.7
TcMYB69	ATMYB119	60.71	28	11	0	4	87	172	199	1.00E-07	44.7
TcMYB69	AtMYB64	57.14	28	12	0	4	87	172	199	1.00E-06	41.6
TcMYB69	AtMYB124	33.7	92	47	3	7	240	93	184	1.00E-06	41.6
TcMYB69	ATMYB88	50	46	23	1	7	144	98	142	6.00E-06	39.3
TcMYB69	AtMYB89	34.15	41	27	0	4	126	123	163	3.00E-05	36.2
TcMYB70	ATMYB105	78.95	38	8	0	1	114	183	220	2.00E-15	68.2
TcMYB70	AtMYB117	75.68	37	9	0	1	111	174	210	1.00E-14	66.2
TcMYB70	ATMYB52	49.23	65	17	1	1	147	81	145	1.00E-13	62.4
TcMYB70	ATMYB110	28.05	164	86	4	1	396	143	297	2.00E-12	59.3
TcMYB70	ATMYB54	80.65	31	6	0	1	93	82	112	3.00E-12	58.2
TcMYB70	AtMYB56	80.65	31	6	0	1	93	169	199	5.00E-12	58.2
TcMYB70	ATMYB69	88.89	27	3	0	4	84	96	122	2.00E-10	52.8
TcMYB70	ATMYB48	41.82	55	30	1	4	162	87	141	3.00E-07	43.1
TcMYB70	ATMYB59-3	39.62	53	32	0	4	162	88	140	4.00E-07	42.7
TcMYB70	ATMYB25	30.26	76	53	1	1	228	126	198	6.00E-07	42.7
TcMYB70	AtMYB1	61.54	26	10	0	1	78	131	156	6.00E-07	42.7
TcMYB70	AtMYB89	62.07	29	11	0	4	90	133	161	9.00E-07	41.2
TcMYB70	AtMYB109	53.57	28	13	0	1	84	132	159	4.00E-06	40
TcMYB70	ATMYB119	51.35	37	18	1	4	114	182	215	6.00E-06	39.7
TcMYB70	ATMYB77	70.83	24	7	0	4	75	83	106	7.00E-06	39.3
TcMYB70	ATMYB44	70.83	24	7	0	4	75	83	106	7.00E-06	39.3
TcMYB70	AtMYB36	21.88	192	149	7	1	573	92	232	7.00E-06	39.3
TcMYB70	AtMYB70	66.67	24	8	0	4	75	90	113	9.00E-06	38.9
TcMYB70	ATMYB118	31.25	80	48	1	4	222	266	345	1.00E-05	38.9
TcMYB70	AtMYB22	27.03	111	77	4	4	324	130	237	1.00E-05	38.1
TcMYB70	AtMYB98	50	28	14	0	4	87	294	321	1.00E-05	38.5
TcMYB70	ATMYB73	62.5	24	9	0	4	75	90	113	2.00E-05	37.7
TcMYB70	ATMYB14	35.29	51	33	0	4	156	92	142	2.00E-05	37.4
TcMYB70	ATMYB35	62.5	24	9	0	4	75	92	115	4.00E-05	37
TcMYB70	AtMYB18	52	25	12	0	4	78	90	114	5.00E-05	36.6
TcMYB70	AtMYB64	28.44	109	78	3	4	330	182	272	6.00E-05	36.6
TcMYB70	AtMYB20	51.85	27	13	0	4	84	92	118	6.00E-05	36.2
TcMYB70	AtMYB43	51.85	27	13	0	4	84	92	118	7.00E-05	36.2
TcMYB70	ATMYB0	30.65	62	39	1	1	174	93	154	7.00E-05	35.8

TcMYB70	AtMYB115	29.29	99	61	4	4	273	235	330	7.00E-05	36.2
TcMYB70	ATMYB123	22.48	129	95	3	1	372	93	206	8.00E-05	35.8
TcMYB70	AtMYB19	48	25	13	0	4	78	92	116	8.00E-05	35.8
TcMYB70	ATMYB66	31.75	63	43	1	1	189	95	154	8.00E-05	35.4
TcMYB70	AtMYB60	33.33	54	36	1	4	165	92	143	8.00E-05	35.8
TcMYB70	ATMYB61	23.76	202	142	8	4	573	92	264	1.00E-04	35.8
TcMYB70	AtMYB103	30.51	59	41	0	4	180	92	150	1.00E-04	35.8
TcMYB70	ATMYB23	52	25	12	0	1	75	91	115	1.00E-04	35
TcMYB70	AtMYB79	41.86	43	25	0	4	132	86	128	1.00E-04	35
TcMYB70	ATMYB71	39.53	43	26	0	4	132	98	140	1.00E-04	35
TcMYB70	AtMYB42	33.87	62	41	1	4	189	92	149	1.00E-04	35
TcMYB70	MYB8	48.28	29	15	0	1	87	91	119	2.00E-04	34.7
TcMYB70	ATMYB12	33.33	69	46	1	1	207	91	147	2.00E-04	35
TcMYB70	AtMYB100	31.07	103	63	5	4	288	103	202	2.00E-04	34.7
TcMYB70	ATMYB3	48.28	29	15	0	1	87	91	119	2.00E-04	34.7
TcMYB70	ATMYB46	22.31	121	81	2	4	327	98	218	2.00E-04	34.7
TcMYB70	AtMYB113	36.84	57	36	2	1	171	87	138	2.00E-04	34.3
TcMYB70	ATMYB90	28.72	94	67	3	1	282	87	163	2.00E-04	34.3
TcMYB70	AtMYB85	54.17	24	11	0	4	75	92	115	3.00E-04	34.3
TcMYB70	AtMYB32	33.33	54	36	1	1	162	91	140	3.00E-04	34.3
TcMYB70	ATMYB4	48.28	29	15	0	1	87	91	119	3.00E-04	34.3
TcMYB70	ATMYB63	29.31	58	41	0	4	177	94	151	3.00E-04	34.3
TcMYB70	ATMYB111	41.18	34	20	0	1	102	91	124	3.00E-04	34.3
TcMYB70	ATMYB68	41.94	31	18	0	1	93	92	122	3.00E-04	34.3
TcMYB70	AtMYB27	44.12	34	19	1	4	105	89	120	3.00E-04	33.9
TcMYB70	AtMYB10	48.28	29	15	0	4	90	94	122	3.00E-04	33.9
TcMYB70	AtMYB45	61.11	18	7	0	4	57	98	115	3.00E-04	33.9
TcMYB70	AtMYB40	50	26	13	0	4	81	92	117	3.00E-04	33.9
TcMYB70	MYB7	52	25	12	0	1	75	91	115	3.00E-04	33.9
TcMYB70	ATMYB84	41.94	31	18	0	1	93	92	122	4.00E-04	33.9
TcMYB70	ATMYB86	46.43	28	15	0	4	87	92	119	4.00E-04	33.9
TcMYB70	AtMYB6	52	25	12	0	1	75	91	115	4.00E-04	33.5
TcMYB70	ATMYB121	40.54	37	22	0	4	114	107	143	5.00E-04	33.5
TcMYB70	ATMYB15	30.91	55	34	1	4	156	92	146	5.00E-04	33.5
TcMYB70	AtMYB82	48.28	29	15	0	1	87	91	119	5.00E-04	33.1
TcMYB70	AtMYB104	50	30	15	1	4	93	96	122	5.00E-04	33.5
TcMYB70	ATMYB75	33.33	51	34	0	1	153	87	137	6.00E-04	33.1
TcMYB70	AtMYB41	50	24	12	0	4	75	92	115	6.00E-04	33.1
TcMYB70	AtMYB62	44.44	27	15	0	4	84	99	125	6.00E-04	33.1
TcMYB70	ATMYB87	30	50	34	1	1	147	92	141	6.00E-04	33.1
TcMYB70	ATMYB80	52	25	12	0	1	75	91	115	7.00E-04	33.1
TcMYB70	ATMYB94	26.47	68	49	1	4	204	92	159	7.00E-04	33.1
TcMYB70	ATMYB11	54.17	24	11	0	4	75	92	115	7.00E-04	33.1
TcMYB70	ATMYB55	50	28	14	0	4	87	104	131	7.00E-04	33.1
TcMYB70	ATMYB5	48	25	13	0	1	75	102	126	8.00E-04	32.7
TcMYB70	ATMYB101	50	26	13	0	4	81	98	123	8.00E-04	33.1
TcMYB70	ATMYB95	39.29	28	17	0	4	87	92	119	8.00E-04	32.7
TcMYB70	ATMYB58	38.71	31	19	0	4	96	94	124	8.00E-04	32.7
TcMYB70	AtMYB116	30.95	42	29	0	4	129	98	139	8.00E-04	32.7
TcMYB70	AtMYB53	52	25	12	0	1	75	91	115	9.00E-04	32.7
TcMYB71	ATMYB5	53.85	65	27	1	1	186	107	171	4.00E-17	71.6
TcMYB71	AtMYB20	66.67	42	14	0	1	126	96	137	3.00E-16	69.7
TcMYB71	ATMYB28	27.73	220	159	4	1	660	96	304	3.00E-16	70.1
TcMYB71	ATMYB29	28.7	230	162	6	1	684	96	282	4.00E-16	69.7
TcMYB71	ATMYB4	47.89	71	36	2	1	210	96	162	5.00E-16	68.9
TcMYB71	AtMYB85	38.38	99	53	3	1	273	96	192	5.00E-16	68.6
TcMYB71	AtMYB51	32.35	136	92	1	1	408	97	231	8.00E-16	68.9
TcMYB71	AtMYB32	52.94	51	24	0	1	153	96	146	8.00E-16	68.2
TcMYB71	AtMYB42	32.46	114	77	2	1	342	96	203	1.00E-15	67.8
TcMYB71	AtMYB43	53.33	60	28	1	1	180	96	151	4.00E-15	66.6
TcMYB71	AtMYB50	52.54	59	27	1	1	174	96	154	7.00E-15	65.9
TcMYB71	AtMYB49	55.93	59	20	1	1	159	96	154	7.00E-15	65.9
TcMYB71	ATMYB3	59.57	47	19	1	1	141	96	138	1.00E-14	64.7
TcMYB71	AtMYB17	54	50	21	1	1	144	96	145	2.00E-14	64.7
TcMYB71	ATMYB61	41.79	67	39	0	1	201	96	162	3.00E-14	64.3
TcMYB71	ATMYB67	40.79	76	38	1	1	207	106	181	3.00E-14	63.9
TcMYB71	MYB8	44.62	65	34	2	1	189	96	159	3.00E-14	62.8
TcMYB71	AtMYB107	78.79	33	7	0	1	99	96	128	3.00E-14	63.9
TcMYB71	AtMYB9	78.79	33	7	0	1	99	96	128	4.00E-14	63.9
TcMYB71	AtMYB76	44.78	67	37	1	1	201	96	161	4.00E-14	63.9
TcMYB71	AtMYB6	65.71	35	12	0	1	105	96	130	4.00E-14	62.8
TcMYB71	ATMYB99	44.93	69	35	1	1	198	104	172	6.00E-14	62.4
TcMYB71	ATMYB92	75.76	33	8	0	1	99	96	128	9.00E-14	62.8
TcMYB71	AtMYB53	75.76	33	8	0	1	99	96	128	1.00E-13	62.4
TcMYB71	MYB7	65.71	35	12	0	1	105	96	130	1.00E-13	62
TcMYB71	ATMYB35	49.15	59	23	1	1	156	96	154	2.00E-13	61.6
TcMYB71	ATMYB16	75.76	33	8	0	1	99	96	128	2.00E-13	61.6
TcMYB71	ATMYB80	58.7	46	19	0	1	138	96	141	3.00E-13	61.2
TcMYB71	AtMYB74	55.32	47	21	0	1	141	97	143	3.00E-13	61.2
TcMYB71	ATMYB102	55.32	47	21	0	1	141	96	142	3.00E-13	61.2

TcMYB71	AtMYB40	43.1	58	33	0	7	180	98	155	3.00E-13	60.5
TcMYB71	ATMYB95	50.85	59	26	1	1	168	96	154	7.00E-13	59.7
TcMYB71	AtMYB41	69.7	33	10	0	1	99	96	128	7.00E-13	59.7
TcMYB71	AtMYB93	75.76	33	8	0	1	99	96	128	8.00E-13	60.1
TcMYB71	ATMYB106	57.69	52	22	1	1	156	139	186	8.00E-13	60.1
TcMYB71	ATMYB86	73.53	34	9	0	1	102	96	129	2.00E-12	58.9
TcMYB71	ATMYB34	29.08	141	86	3	1	381	96	234	3.00E-12	58.2
TcMYB71	ATMYB122	46.67	60	31	1	1	177	96	155	6.00E-12	57.4
TcMYB71	AtMYB47	42.03	69	36	2	1	195	96	163	1.00E-11	55.8
TcMYB71	ATMYB55	61.9	42	16	0	1	126	108	149	3.00E-11	55.5
TcMYB71	ATMYB48	39.19	74	45	1	1	222	91	161	3.00E-10	51.6
TcMYB71	ATMYB38	51.06	47	23	1	1	141	97	142	1.00E-09	50.4
TcMYB71	AtMYB10	35.96	89	44	2	4	231	99	187	3.00E-09	48.9
TcMYB71	ATMYB30	44.62	65	33	2	1	186	96	158	3.00E-09	49.3
TcMYB71	ATMYB11	31.58	114	41	5	1	231	96	209	3.00E-09	49.3
TcMYB71	ATMYB0	81.82	22	4	0	1	66	98	119	3.00E-09	48.5
TcMYB71	ATMYB12	30.84	107	65	4	1	294	96	202	4.00E-09	48.9
TcMYB71	ATMYB66	45	60	33	2	1	180	100	151	5.00E-09	47.8
TcMYB71	ATMYB121	38.6	57	35	0	1	171	111	167	6.00E-09	48.1
TcMYB71	ATMYB23	85.71	21	3	0	1	63	96	116	7.00E-09	47.4
TcMYB71	AtMYB82	51.16	43	12	1	1	102	96	138	1.00E-08	46.6
TcMYB71	ATMYB87	48.94	47	24	1	1	141	97	142	1.00E-08	47
TcMYB71	AtMYB36	41.94	62	35	2	1	183	97	156	2.00E-08	47
TcMYB71	ATMYB37	41.38	58	32	1	1	168	97	154	2.00E-08	46.6
TcMYB71	ATMYB111	42.86	63	33	3	1	180	96	157	3.00E-08	46.2
TcMYB71	ATMYB13	41.07	56	33	0	1	168	96	151	4.00E-08	45.4
TcMYB71	AtMYB116	34.38	64	36	1	1	174	102	165	4.00E-08	45.4
TcMYB71	ATMYB84	75	24	6	0	1	72	97	120	5.00E-08	45.4
TcMYB71	AtMYB60	63.33	30	11	0	1	90	96	125	6.00E-08	45.1
TcMYB71	ATMYB120	45	60	33	3	1	180	110	158	6.00E-08	45.4
TcMYB71	ATMYB15	41.03	78	25	4	1	171	96	173	8.00E-08	44.7
TcMYB71	AtMYB114	80	20	4	0	1	60	92	111	8.00E-08	43.1
TcMYB71	ATMYB31	77.27	22	5	0	1	66	96	117	9.00E-08	44.7
TcMYB71	ATMYB96	41.54	65	34	2	1	183	96	150	9.00E-08	44.7
TcMYB71	ATMYB68	70.83	24	7	0	1	72	97	120	1.00E-07	44.7
TcMYB71	ATMYB46	32.39	71	48	2	1	213	102	157	1.00E-07	44.3
TcMYB71	ATMYB59-3	41.38	58	34	2	1	174	92	140	1.00E-07	43.9
TcMYB71	ATMYB63	38.96	77	44	3	1	222	98	171	1.00E-07	43.9
TcMYB71	ATMYB72	39.66	58	35	1	1	174	98	149	1.00E-07	43.9
TcMYB71	AtMYB83	32.22	90	60	3	1	267	114	184	2.00E-07	43.9
TcMYB71	ATMYB75	45.65	46	24	2	1	135	92	127	2.00E-07	43.5
TcMYB71	ATMYB58	33.78	74	46	1	1	213	98	171	2.00E-07	43.5
TcMYB71	ATMYB94	80.95	21	4	0	1	63	96	116	2.00E-07	43.5
TcMYB71	AtMYB113	80	20	4	0	1	60	92	111	2.00E-07	43.1
TcMYB71	ATMYB90	80	20	4	0	1	60	92	111	2.00E-07	43.1
TcMYB71	AtMYB18	25.28	178	127	6	1	516	94	246	3.00E-07	43.1
TcMYB71	AtMYB103	29.63	81	57	1	1	243	96	171	4.00E-07	42.7
TcMYB71	AtMYB79	80	20	4	0	1	60	90	109	4.00E-07	42.4
TcMYB71	ATMYB71	80	20	4	0	1	60	102	121	4.00E-07	42.4
TcMYB71	AtMYB27	80	20	4	0	1	60	93	112	7.00E-07	41.6
TcMYB71	ATMYB14	76.19	21	5	0	1	63	96	116	7.00E-07	41.6
TcMYB71	ATMYB26	48.57	35	18	0	1	105	105	139	7.00E-07	42
TcMYB71	AtMYB97	38.98	59	29	2	1	156	103	160	1.00E-06	41.2
TcMYB71	AtMYB19	42.55	47	27	2	1	141	96	136	2.00E-06	40.4
TcMYB71	ATMYB123	71.43	21	6	0	1	63	98	118	2.00E-06	40
TcMYB71	AtMYB24	60	25	10	0	1	75	101	125	3.00E-06	39.7
TcMYB71	ATMYB101	70	20	6	0	1	60	102	121	3.00E-06	40
TcMYB71	ATMYB33	70	20	6	0	1	60	116	135	4.00E-06	40
TcMYB71	ATMYB65	70	20	6	0	1	60	125	144	4.00E-06	40
TcMYB71	AtMYB62	50	32	16	0	1	96	103	134	5.00E-06	39.3
TcMYB71	ATMYB3	32.26	62	42	0	1	186	104	165	5.00E-06	38.9
TcMYB71	AtMYB45	32.84	67	45	1	1	201	102	166	6.00E-06	38.9
TcMYB71	AtMYB112	50	34	15	1	1	96	116	149	1.00E-05	38.1
TcMYB71	AtMYB81	73.68	19	5	0	1	57	104	122	1.00E-05	38.5
TcMYB71	ATMYB2	70	20	6	0	1	60	104	123	1.00E-05	38.1
TcMYB71	ATMYB78	73.68	19	5	0	1	57	124	142	2.00E-05	37.7
TcMYB71	AtMYB108	73.68	19	5	0	1	57	103	121	2.00E-05	37.7
TcMYB71	AtMYB109	25.25	198	112	8	1	486	137	328	2.00E-05	37.4
TcMYB71	ATMYB57	27.78	72	51	1	1	213	109	180	4.00E-05	36.2
TcMYB71	AtMYB56	26.09	69	48	2	4	201	175	241	4.00E-05	36.6
TcMYB71	ATMYB25	65	20	7	0	1	60	131	150	5.00E-05	36.2
TcMYB71	ATMYB105	35.56	45	29	1	4	138	189	231	7.00E-05	35.8
TcMYB71	AtMYB1	59.09	22	9	0	1	66	136	157	1.00E-04	35.4
TcMYB71	AtMYB117	30.77	52	36	0	4	159	180	231	2.00E-04	34.3
TcMYB71	ATMYB73	56.52	23	10	0	7	75	96	118	3.00E-04	33.9
TcMYB71	AtMYB104	92.31	13	1	0	4	42	101	113	4.00E-04	33.5
TcMYB71	AtMYB100	73.33	15	4	0	1	45	107	121	7.00E-04	32.3
TcMYB72	AtMYB98	49.64	139	69	1	457	870	181	319	2.00E-45	162
TcMYB72	AtMYB98	37.89	95	59	1	406	690	217	310	6.00E-18	80.1
TcMYB72	ATMYB119	41.67	192	112	3	289	864	24	205	1.00E-42	154

TcMYB72	ATMYB119	35.29	51	32	1	718	867	105	155	3.00E-04	36.6
TcMYB72	ATMYB118	59.62	104	42	0	553	864	186	289	4.00E-41	150
TcMYB72	ATMYB118	33.98	103	66	2	388	690	181	282	8.00E-16	73.6
TcMYB72	ATMYB118	33.96	53	34	1	712	867	187	239	4.00E-05	39.3
TcMYB72	AtMYB64	55.08	118	53	0	511	864	88	205	4.00E-41	149
TcMYB72	AtMYB64	29.7	101	71	1	388	690	99	198	3.00E-13	65.5
TcMYB72	AtMYB64	24.53	106	79	2	553	867	63	155	7.00E-04	35.4
TcMYB72	ATMYB73	58.49	106	44	0	547	864	8	113	5.00E-38	137
TcMYB72	ATMYB73	33.68	95	63	1	406	690	13	106	4.00E-15	70.1
TcMYB72	AtMYB1	53.77	106	49	0	559	876	54	159	1.00E-37	138
TcMYB72	AtMYB1	35.85	106	68	1	373	690	44	148	6.00E-18	79.7
TcMYB72	AtMYB115	54	100	46	0	562	861	158	257	7.00E-36	132
TcMYB72	AtMYB115	41.05	95	56	1	406	690	158	251	6.00E-18	79.3
TcMYB72	ATMYB44	53.77	106	49	0	559	876	5	110	7.00E-36	131
TcMYB72	ATMYB44	35.79	95	61	1	406	690	6	99	9.00E-15	68.9
TcMYB72	ATMYB77	48.72	117	60	0	559	909	5	121	1.00E-35	130
TcMYB72	ATMYB77	36.84	95	60	1	406	690	6	99	2.00E-16	73.9
TcMYB72	AtMYB70	56.86	102	44	0	559	864	12	113	2.00E-35	130
TcMYB72	AtMYB70	30.36	112	78	1	406	741	13	123	5.00E-14	66.6
TcMYB72	AtMYB109	53.64	110	51	0	559	888	55	164	6.00E-35	130
TcMYB72	AtMYB109	31.75	126	86	2	373	750	45	168	1.00E-15	72.8
TcMYB72	ATMYB105	50.93	108	53	0	562	885	107	214	2.00E-33	125
TcMYB72	ATMYB105	30.21	96	67	1	403	690	106	200	1.00E-13	65.9
TcMYB72	ATMYB105	38	50	31	1	397	546	156	204	5.00E-05	38.9
TcMYB72	AtMYB100	53.61	97	45	0	571	861	29	125	2.00E-33	122
TcMYB72	AtMYB100	35.79	95	61	1	406	690	26	119	6.00E-15	68.2
TcMYB72	ATMYB69	49.52	105	53	0	550	864	15	119	2.00E-33	122
TcMYB72	ATMYB69	36.05	86	55	1	406	663	19	103	6.00E-15	68.6
TcMYB72	AtMYB56	53.47	101	47	0	562	864	93	193	3.00E-33	124
TcMYB72	AtMYB56	28.7	108	77	1	367	690	80	186	3.00E-13	64.7
TcMYB72	ATMYB54	49.5	101	51	0	562	864	6	106	6.00E-33	120
TcMYB72	ATMYB54	32.29	96	65	1	403	690	5	99	4.00E-15	68.9
TcMYB72	ATMYB54	36	50	32	1	397	546	55	103	6.00E-04	35
TcMYB72	ATMYB110	50.93	108	53	0	562	885	67	174	8.00E-33	122
TcMYB72	ATMYB110	29.29	99	70	1	394	690	63	160	7.00E-12	60.1
TcMYB72	ATMYB25	49.15	118	60	0	559	912	49	166	8.00E-33	124
TcMYB72	ATMYB25	32.43	111	75	1	370	702	38	147	5.00E-15	70.5
TcMYB72	ATMYB52	48.51	101	52	0	562	864	5	105	9.00E-33	120
TcMYB72	ATMYB52	33.33	96	64	1	403	690	4	98	1.00E-15	70.5
TcMYB72	ATMYB52	34	50	33	1	397	546	54	102	5.00E-04	35.4
TcMYB72	AtMYB117	53.47	101	47	0	562	864	98	198	9.00E-33	123
TcMYB72	AtMYB117	24.84	157	118	2	220	690	46	191	8.00E-14	66.6
TcMYB72	AtMYB117	40	50	30	1	397	546	147	195	4.00E-05	39.3
TcMYB72	AtMYB49	30.1	289	184	8	562	1374	14	291	2.00E-31	119
TcMYB72	AtMYB49	29.81	104	72	2	406	714	14	116	6.00E-12	60.5
TcMYB72	AtMYB89	48.62	109	56	0	565	891	57	165	3.00E-31	114
TcMYB72	AtMYB89	28.17	142	101	3	391	813	50	182	2.00E-14	65.5
TcMYB72	ATMYB63	48.08	104	53	1	562	870	16	119	6.00E-30	113
TcMYB72	ATMYB63	31.86	113	76	2	406	741	16	127	3.00E-14	67
TcMYB72	ATMYB95	38.62	145	88	1	556	987	12	156	1.00E-29	112
TcMYB72	AtMYB53	47.62	105	54	1	556	867	12	116	2.00E-29	112
TcMYB72	AtMYB53	29.81	104	72	2	406	714	14	116	1.00E-11	59.7
TcMYB72	ATMYB65	49.04	104	52	1	556	864	41	144	2.00E-29	116
TcMYB72	ATMYB65	32.8	125	70	4	406	738	43	166	1.00E-12	63.9
TcMYB72	ATMYB92	48.57	105	53	1	556	867	12	116	3.00E-29	112
TcMYB72	ATMYB92	31.73	104	70	2	406	714	14	116	2.00E-12	62.4
TcMYB72	AtMYB9	47.62	105	54	1	556	867	12	116	3.00E-29	112
TcMYB72	AtMYB9	33.65	104	68	2	406	714	14	116	1.00E-13	65.9
TcMYB72	ATMYB58	46.67	105	55	1	562	873	16	120	6.00E-29	110
TcMYB72	ATMYB58	28.97	107	75	2	406	723	16	121	2.00E-11	58.5
TcMYB72	ATMYB35	47.17	106	55	1	553	867	11	116	9.00E-29	110
TcMYB72	ATMYB35	31.13	106	72	2	406	720	14	118	1.00E-10	56.6
TcMYB72	ATMYB26	35.11	188	100	3	562	1059	14	201	9.00E-29	112
TcMYB72	ATMYB26	26.61	124	81	3	391	732	9	127	5.00E-09	51.6
TcMYB72	ATMYB67	45.28	106	56	2	562	873	24	128	9.00E-29	110
TcMYB72	ATMYB67	29.31	116	81	3	391	735	19	129	1.00E-10	56.6
TcMYB72	AtMYB27	44.76	105	56	2	556	864	9	112	1.00E-28	108
TcMYB72	AtMYB27	34.34	99	64	2	406	699	11	108	7.00E-14	65.1
TcMYB72	AtMYB22	45.63	103	56	1	553	861	51	152	1.00E-28	108
TcMYB72	AtMYB22	37.5	96	60	2	403	690	53	146	1.00E-15	70.5
TcMYB72	AtMYB22	28.3	53	38	0	709	867	51	103	9.00E-06	40.8
TcMYB72	AtMYB45	35.91	181	113	3	562	1095	20	193	2.00E-28	108
TcMYB72	AtMYB45	36.19	105	66	2	391	702	15	118	2.00E-13	63.9
TcMYB72	AtMYB107	46.67	105	55	1	556	867	12	116	2.00E-28	109
TcMYB72	AtMYB107	31.73	104	70	2	406	714	14	116	8.00E-13	63.2
TcMYB72	ATMYB72	42.98	114	63	2	562	897	16	128	3.00E-28	108
TcMYB72	ATMYB72	28.44	109	77	2	391	714	11	118	4.00E-12	60.8
TcMYB72	AtMYB93	41.41	128	66	2	556	912	12	139	3.00E-28	110
TcMYB72	AtMYB93	29.81	104	72	2	406	714	14	116	8.00E-11	57.4
TcMYB72	ATMYB102	48.57	105	53	1	556	867	12	116	3.00E-28	110

TcMYB72	ATMYB102	30.77	104	71	2	406	714	14	116	1.00E-11	60.1
TcMYB72	ATMYB33	44.83	116	63	2	520	864	23	135	3.00E-28	112
TcMYB72	ATMYB33	32	125	71	4	406	738	34	157	2.00E-12	62.8
TcMYB72	AtMYB41	46.6	103	54	1	562	867	14	116	3.00E-28	108
TcMYB72	AtMYB41	29.81	104	72	2	406	714	14	116	4.00E-12	60.5
TcMYB72	ATMYB15	46.3	108	57	1	556	876	12	119	4.00E-28	108
TcMYB72	ATMYB15	33.33	105	69	2	406	717	14	117	4.00E-14	66.6
TcMYB72	AtMYB81	46.73	107	56	1	544	861	16	122	5.00E-28	110
TcMYB72	AtMYB81	30.69	101	69	2	403	702	21	120	2.00E-11	59.3
TcMYB72	ATMYB3	45.71	105	56	1	562	873	14	118	7.00E-28	106
TcMYB72	ATMYB3	31.43	105	71	2	403	714	13	116	1.00E-13	64.7
TcMYB72	ATMYB3	34.62	52	34	1	406	561	67	117	6.00E-04	35
TcMYB72	MYB8	42.72	103	58	1	562	867	14	116	8.00E-28	105
TcMYB72	MYB8	30.48	105	72	2	403	714	13	116	6.00E-12	58.9
TcMYB72	ATMYB59-3	40	110	65	1	538	864	2	111	1.00E-27	105
TcMYB72	ATMYB59-3	32.43	111	74	2	391	720	5	114	6.00E-16	71.2
TcMYB72	AtMYB6	43.4	106	59	1	562	876	14	119	2.00E-27	104
TcMYB72	AtMYB6	30.19	106	73	2	400	714	12	116	1.00E-11	58.5
TcMYB72	ATMYB122	46.6	103	54	1	556	861	12	114	3.00E-27	106
TcMYB72	ATMYB122	29	100	70	2	406	702	14	112	2.00E-11	58.9
TcMYB72	AtMYB51	28.3	311	222	10	556	1485	13	271	3.00E-27	107
TcMYB72	AtMYB51	29.25	106	74	2	406	720	15	119	3.00E-12	61.6
TcMYB72	ATMYB14	41.82	110	63	1	562	888	14	123	3.00E-27	104
TcMYB72	ATMYB14	30.84	107	73	2	406	723	14	119	8.00E-15	68.2
TcMYB72	AtMYB74	38.35	133	81	2	556	951	13	144	4.00E-27	106
TcMYB72	AtMYB74	30.19	106	73	2	406	720	15	119	1.00E-11	59.7
TcMYB72	AtMYB18	48.54	103	51	2	562	864	12	113	4.00E-27	105
TcMYB72	AtMYB18	31.36	118	80	3	397	747	9	120	6.00E-12	60.1
TcMYB72	AtMYB50	47.17	106	54	2	556	867	12	116	4.00E-27	105
TcMYB72	AtMYB50	29.73	111	77	2	391	720	9	118	2.00E-10	55.5
TcMYB72	ATMYB48	43.14	102	57	1	562	864	9	110	4.00E-27	104
TcMYB72	ATMYB48	33.96	106	69	2	406	720	9	113	1.00E-15	70.9
TcMYB72	ATMYB86	47.17	106	54	2	556	867	12	116	4.00E-27	106
TcMYB72	ATMYB86	27.03	111	80	2	391	720	9	118	9.00E-10	53.9
TcMYB72	AtMYB32	35.06	174	107	4	562	1065	14	183	4.00E-27	104
TcMYB72	AtMYB32	31.13	106	72	2	400	714	12	116	7.00E-13	62.8
TcMYB72	ATMYB80	42.06	107	61	1	553	870	11	117	5.00E-27	105
TcMYB72	ATMYB80	30.19	106	73	3	406	720	14	114	1.00E-09	53.5
TcMYB72	ATMYB121	45.54	101	54	1	562	861	29	129	5.00E-27	104
TcMYB72	ATMYB121	28.66	164	116	2	391	879	24	186	1.00E-15	70.9
TcMYB72	AtMYB19	47.57	103	52	2	562	864	14	115	5.00E-27	104
TcMYB72	AtMYB19	30.19	106	73	2	388	702	8	112	7.00E-12	59.7
TcMYB72	AtMYB19	29.85	67	46	2	406	603	67	132	4.00E-05	38.9
TcMYB72	ATMYB55	44.44	117	52	1	556	867	12	128	5.00E-27	106
TcMYB72	ATMYB55	27.05	122	76	2	391	717	9	129	4.00E-10	55.1
TcMYB72	ATMYB2	42.16	102	58	1	562	864	22	123	6.00E-27	104
TcMYB72	ATMYB2	33.91	115	75	2	376	717	12	125	4.00E-14	66.6
TcMYB72	ATMYB71	44.34	106	57	2	562	873	20	124	7.00E-27	103
TcMYB72	ATMYB71	33.63	113	74	2	406	741	20	131	2.00E-14	67.4
TcMYB72	AtMYB79	45.63	103	54	2	562	864	8	109	8.00E-27	103
TcMYB72	AtMYB79	32.38	105	70	2	406	717	8	111	2.00E-13	64.3
TcMYB72	ATMYB61	48.11	106	53	2	556	867	12	116	1.00E-26	105
TcMYB72	ATMYB61	29.73	111	77	2	391	720	9	118	8.00E-11	57.4
TcMYB72	MYB7	43.81	105	58	1	562	873	14	118	1.00E-26	103
TcMYB72	MYB7	31.43	105	71	2	403	714	13	116	2.00E-12	61.2
TcMYB72	AtMYB112	39.09	110	66	1	535	861	25	134	1.00E-26	102
TcMYB72	AtMYB112	29.52	105	73	2	406	717	34	137	1.00E-10	55.8
TcMYB72	AtMYB20	46.23	106	55	2	556	867	12	116	2.00E-26	103
TcMYB72	AtMYB20	27.36	106	76	2	406	720	14	118	5.00E-10	54.3
TcMYB72	ATMYB46	44.66	103	56	1	562	867	20	122	2.00E-26	102
TcMYB72	ATMYB46	26.36	110	80	2	394	720	16	124	1.00E-08	49.7
TcMYB72	ATMYB0	39.66	116	69	1	547	891	11	126	2.00E-26	101
TcMYB72	ATMYB0	34.58	107	69	2	406	723	16	121	8.00E-15	67.8
TcMYB72	AtMYB97	46.6	103	54	1	556	861	19	121	3.00E-26	105
TcMYB72	AtMYB97	35	100	64	2	406	702	21	119	3.00E-12	62
TcMYB72	AtMYB103	46.6	103	54	1	562	867	14	116	3.00E-26	104
TcMYB72	AtMYB103	27.03	111	80	2	391	720	9	118	2.00E-10	55.8
TcMYB72	AtMYB43	46.23	106	55	2	556	867	12	116	3.00E-26	103
TcMYB72	AtMYB43	28.3	106	75	2	406	720	14	118	5.00E-10	54.7
TcMYB72	AtMYB85	47.12	104	53	2	562	867	14	116	3.00E-26	102
TcMYB72	AtMYB85	28.3	106	75	2	406	720	14	118	4.00E-10	54.3
TcMYB72	AtMYB17	46.3	108	56	2	556	873	12	118	3.00E-26	102
TcMYB72	AtMYB17	28.85	104	73	2	406	714	14	116	7.00E-12	60.1
TcMYB72	ATMYB13	33.33	183	112	5	556	1074	12	187	4.00E-26	101
TcMYB72	ATMYB13	30.77	104	71	2	406	714	14	116	2.00E-12	61.2
TcMYB72	ATMYB28	44.95	109	59	1	544	867	8	116	6.00E-26	103
TcMYB72	ATMYB28	30.77	104	71	2	406	714	14	116	8.00E-11	57.4
TcMYB72	ATMYB4	43.69	103	57	1	562	867	14	116	6.00E-26	101
TcMYB72	ATMYB4	32.08	106	71	2	400	714	12	116	4.00E-13	63.5
TcMYB72	ATMYB4	36.54	52	33	1	406	561	67	117	4.00E-04	35.8

TcMYB72	AtMYB116	41.18	102	59	1	562	864	20	121	6.00E-26	101
TcMYB72	AtMYB116	30.7	114	78	3	382	720	15	124	2.00E-11	58.5
TcMYB72	ATMYB66	41.75	103	59	1	562	867	18	120	8.00E-26	99
TcMYB72	ATMYB66	34.62	104	67	2	406	714	18	120	6.00E-16	70.5
TcMYB72	AtMYB42	46.15	104	54	2	562	867	14	116	9.00E-26	101
TcMYB72	AtMYB42	24.7	166	108	3	406	852	14	178	2.00E-09	52.8
TcMYB72	ATMYB57	40.91	110	64	1	535	861	18	127	9.00E-26	99
TcMYB72	ATMYB57	32.38	105	70	2	406	717	27	130	2.00E-11	57.4
TcMYB72	AtMYB108	41.35	104	60	1	553	861	18	121	1.00E-25	102
TcMYB72	AtMYB108	30.19	106	73	2	406	720	21	125	8.00E-11	57
TcMYB72	AtMYB10	43.27	104	57	2	562	867	16	118	1.00E-25	99.8
TcMYB72	AtMYB10	33.96	106	69	2	406	720	16	120	2.00E-14	67
TcMYB72	AtMYB24	40.78	103	60	1	562	867	19	121	1.00E-25	99
TcMYB72	AtMYB24	32.48	117	78	2	373	720	8	123	2.00E-12	60.1
TcMYB72	ATMYB16	41.9	105	60	1	556	867	12	116	1.00E-25	101
TcMYB72	ATMYB16	27.36	106	76	2	406	720	14	118	2.00E-10	55.8
TcMYB72	ATMYB101	48.57	105	52	2	556	864	18	121	1.00E-25	104
TcMYB72	ATMYB101	34	100	65	2	394	690	16	114	2.00E-11	59.7
TcMYB72	AtMYB40	40.57	106	61	2	556	867	12	116	2.00E-25	99.8
TcMYB72	AtMYB40	32.71	107	70	4	406	720	14	114	1.00E-08	49.7
TcMYB72	ATMYB29	44.86	107	58	1	544	861	8	114	2.00E-25	101
TcMYB72	ATMYB29	32	100	67	2	406	702	14	112	2.00E-10	56.2
TcMYB72	AtMYB76	43.12	109	61	1	544	867	8	116	3.00E-25	100
TcMYB72	AtMYB76	29.91	107	74	2	406	723	14	119	1.00E-10	56.6
TcMYB72	ATMYB106	41.9	105	60	1	556	867	55	159	4.00E-25	101
TcMYB72	ATMYB106	28.3	106	75	2	406	720	57	161	1.00E-10	57
TcMYB72	ATMYB3	40.78	103	60	1	562	867	22	124	4.00E-25	97.8
TcMYB72	ATMYB3	25.99	177	118	4	406	897	22	192	3.00E-12	60.1
TcMYB72	AtMYB62	39.42	104	62	1	556	864	19	122	4.00E-25	99.4
TcMYB72	AtMYB62	31.78	107	71	4	406	720	21	125	3.00E-12	60.8
TcMYB72	ATMYB123	44.76	105	57	1	556	867	14	118	8.00E-25	97.8
TcMYB72	ATMYB123	26.72	116	84	2	403	747	15	129	5.00E-12	60.1
TcMYB72	ATMYB5	40.78	103	60	1	562	867	25	127	8.00E-25	97.4
TcMYB72	ATMYB5	28.7	108	72	3	406	714	25	127	1.00E-10	55.5
TcMYB72	ATMYB23	40.78	103	60	1	562	867	14	116	1.00E-24	96.3
TcMYB72	ATMYB23	33.33	111	73	2	385	714	7	116	4.00E-16	71.2
TcMYB72	AtMYB83	42.06	107	61	1	550	867	28	134	1.00E-24	99.4
TcMYB72	AtMYB83	29.63	108	75	2	406	726	32	138	3.00E-08	48.9
TcMYB72	ATMYB34	43.69	103	57	1	562	867	14	116	1.00E-24	98.2
TcMYB72	ATMYB34	28.3	106	75	2	406	720	14	118	1.00E-10	56.2
TcMYB72	AtMYB47	42.31	104	59	1	562	870	14	117	1.00E-24	97.4
TcMYB72	AtMYB47	23.64	110	79	3	406	720	14	118	7.00E-08	47.4
TcMYB72	ATMYB96	41.82	110	63	1	562	888	14	123	2.00E-24	98.6
TcMYB72	ATMYB96	32.46	114	76	2	406	744	14	126	6.00E-14	67
TcMYB72	ATMYB96	33.87	62	41	1	406	591	67	127	9.00E-05	38.1
TcMYB72	ATMYB120	45.63	103	55	1	556	861	26	128	2.00E-24	100
TcMYB72	ATMYB120	34	100	65	2	406	702	28	126	7.00E-11	58.2
TcMYB72	AtMYB114	42.59	108	57	2	556	864	8	111	4.00E-24	92
TcMYB72	AtMYB114	29.63	108	75	2	385	705	3	109	1.00E-13	62
TcMYB72	AtMYB36	42.99	107	59	2	553	867	11	117	4.00E-24	97.4
TcMYB72	AtMYB36	31.03	116	78	4	406	747	14	124	4.00E-11	58.2
TcMYB72	ATMYB31	43.93	107	55	2	562	867	14	116	7.00E-24	96.7
TcMYB72	ATMYB31	30.77	104	71	2	406	714	14	116	2.00E-11	59.3
TcMYB72	AtMYB60	44.66	103	56	1	562	867	14	116	1.00E-23	95.1
TcMYB72	AtMYB60	28.95	152	92	5	406	813	14	160	1.00E-13	65.1
TcMYB72	ATMYB94	42.72	103	58	1	562	867	14	116	1.00E-23	96.3
TcMYB72	ATMYB94	31.13	106	72	2	406	720	14	118	7.00E-13	63.5
TcMYB72	AtMYB124	39.8	98	59	0	571	864	28	125	2.00E-23	97.1
TcMYB72	AtMYB124	28.7	108	77	1	415	738	28	134	2.00E-13	66.2
TcMYB72	ATMYB11	39.32	117	70	1	562	909	14	130	2.00E-23	95.5
TcMYB72	ATMYB11	30.77	104	71	2	406	714	14	116	1.00E-10	56.6
TcMYB72	ATMYB30	42.72	103	58	1	562	867	14	116	3.00E-23	94.7
TcMYB72	ATMYB30	30.25	119	78	3	406	747	14	127	1.00E-12	62.4
TcMYB72	ATMYB99	43.36	113	55	3	556	867	13	124	3.00E-23	92.8
TcMYB72	ATMYB99	29.73	111	70	4	406	714	15	124	1.00E-09	52.8
TcMYB72	ATMYB37	42.45	106	59	2	562	873	14	119	3.00E-23	94.7
TcMYB72	ATMYB37	33.03	109	67	4	406	714	14	117	5.00E-11	57.8
TcMYB72	ATMYB75	42.59	108	57	2	556	864	8	111	6.00E-23	92
TcMYB72	ATMYB75	29.63	108	75	2	385	705	3	109	9.00E-13	62
TcMYB72	ATMYB90	29.19	209	143	4	556	1167	8	198	6.00E-23	92
TcMYB72	ATMYB90	29.63	108	75	2	385	705	3	109	4.00E-12	60.1
TcMYB72	ATMYB111	40	110	65	1	556	882	12	121	7.00E-23	94
TcMYB72	ATMYB111	32.69	104	69	2	406	714	14	116	9.00E-12	60.1
TcMYB72	AtMYB82	37.8	127	78	1	562	939	14	140	7.00E-23	90.5
TcMYB72	AtMYB82	31.68	101	68	2	406	705	14	113	8.00E-13	61.2
TcMYB72	ATMYB84	42.06	107	60	2	553	867	11	117	1.00E-22	92.8
TcMYB72	ATMYB84	29.55	132	82	5	406	768	14	144	6.00E-11	57.4
TcMYB72	ATMYB12	30.82	159	109	2	562	1035	14	165	2.00E-22	92.8
TcMYB72	ATMYB12	26.73	217	147	7	406	1020	14	219	4.00E-12	61.6
TcMYB72	ATMYB68	41.12	107	61	2	553	867	11	117	3.00E-22	92.8

TcMYB72	ATMYB68	28.48	158	104	5	406	852	14	164	8.00E-11	57.4
TcMYB72	ATMYB78	34.78	115	60	2	562	861	28	142	4.00E-22	91.3
TcMYB72	ATMYB78	27.73	119	72	2	406	720	28	146	2.00E-09	52.8
TcMYB72	ATMYB87	39.62	106	62	2	562	873	14	119	4.00E-22	90.9
TcMYB72	ATMYB87	33.63	113	73	4	406	738	14	121	7.00E-12	60.1
TcMYB72	ATMYB88	37.76	98	61	0	571	864	33	130	7.00E-22	92.8
TcMYB72	ATMYB88	29.46	112	79	1	415	750	33	143	9.00E-15	70.5
TcMYB72	AtMYB104	40.95	105	57	2	562	861	18	118	9.00E-22	91.3
TcMYB72	AtMYB104	32.67	101	63	3	403	690	17	112	4.00E-12	61.6
TcMYB72	AtMYB113	40.74	108	59	2	556	864	8	111	3.00E-21	87
TcMYB72	AtMYB113	31.68	101	68	2	406	705	10	109	4.00E-12	60.1
TcMYB72	ATMYB38	36.61	112	69	1	553	882	11	122	1.00E-20	86.3
TcMYB72	ATMYB38	31.78	107	71	4	406	720	14	115	2.00E-09	52.4
TcMYB72	ATMYB91	37.23	94	56	1	571	843	7	100	1.00E-16	75.5
TcMYB72	ATMYB91	29.23	130	89	3	415	795	7	129	6.00E-14	67
TcMYB72	ATMYB91	35.71	70	45	3	406	615	59	123	6.00E-04	35.4
