# Supplementary figures and images for: The R2R3-MYB transcription factor family in Taxus chinensis: identification, characterization, expression profiling and posttranscriptional regulation analysis
Source: PeerJ. 2020 Feb 17;8:e8473. doi: 10.7717/peerj.8473 (PMC7032060; doi:10.7717/peerj.8473)

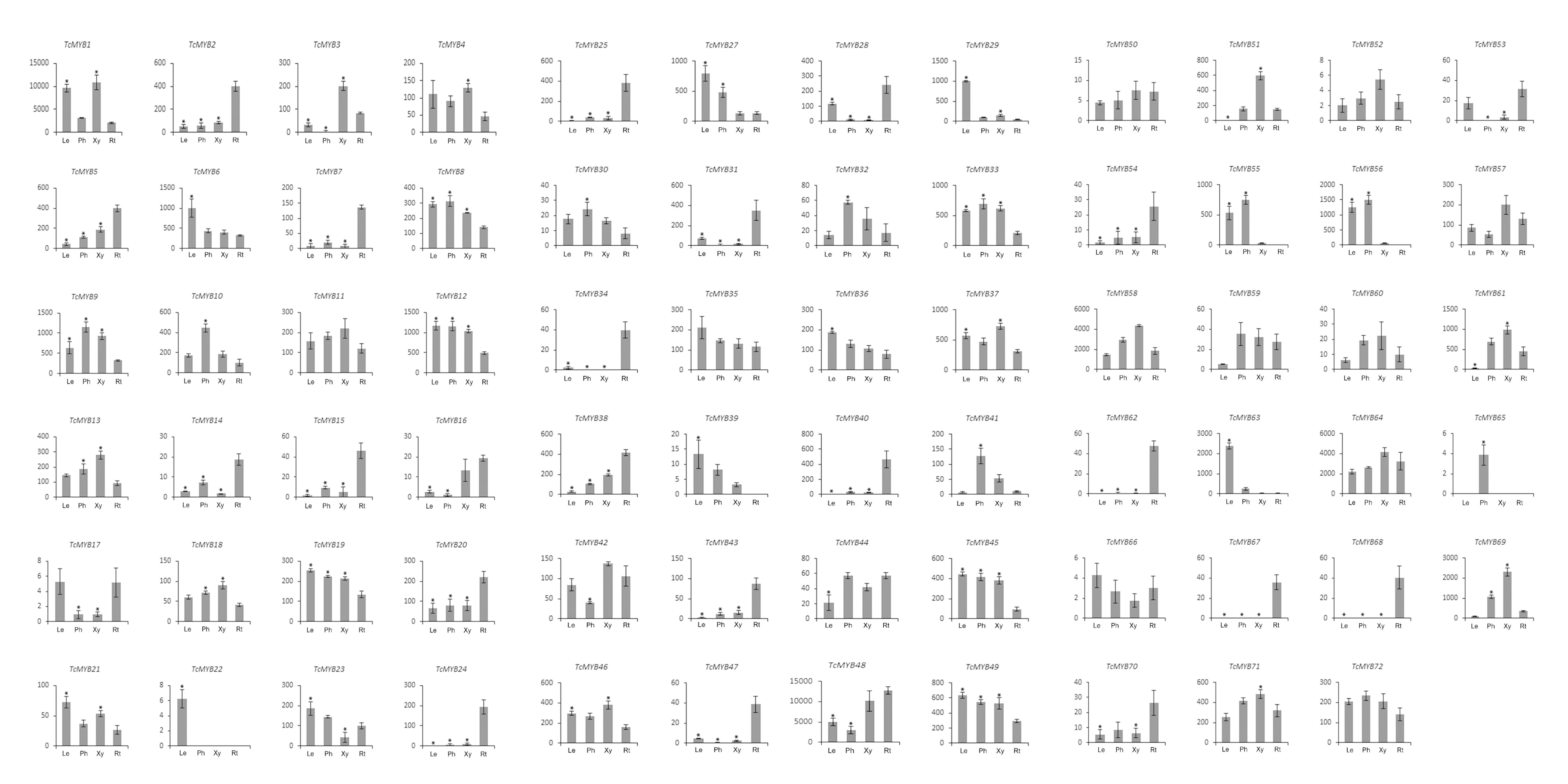

Supplement: Figure S1 — The relative expression of TcMYBs were compared to Tcactin *10000. [file peerj-08-8473-s004.png]

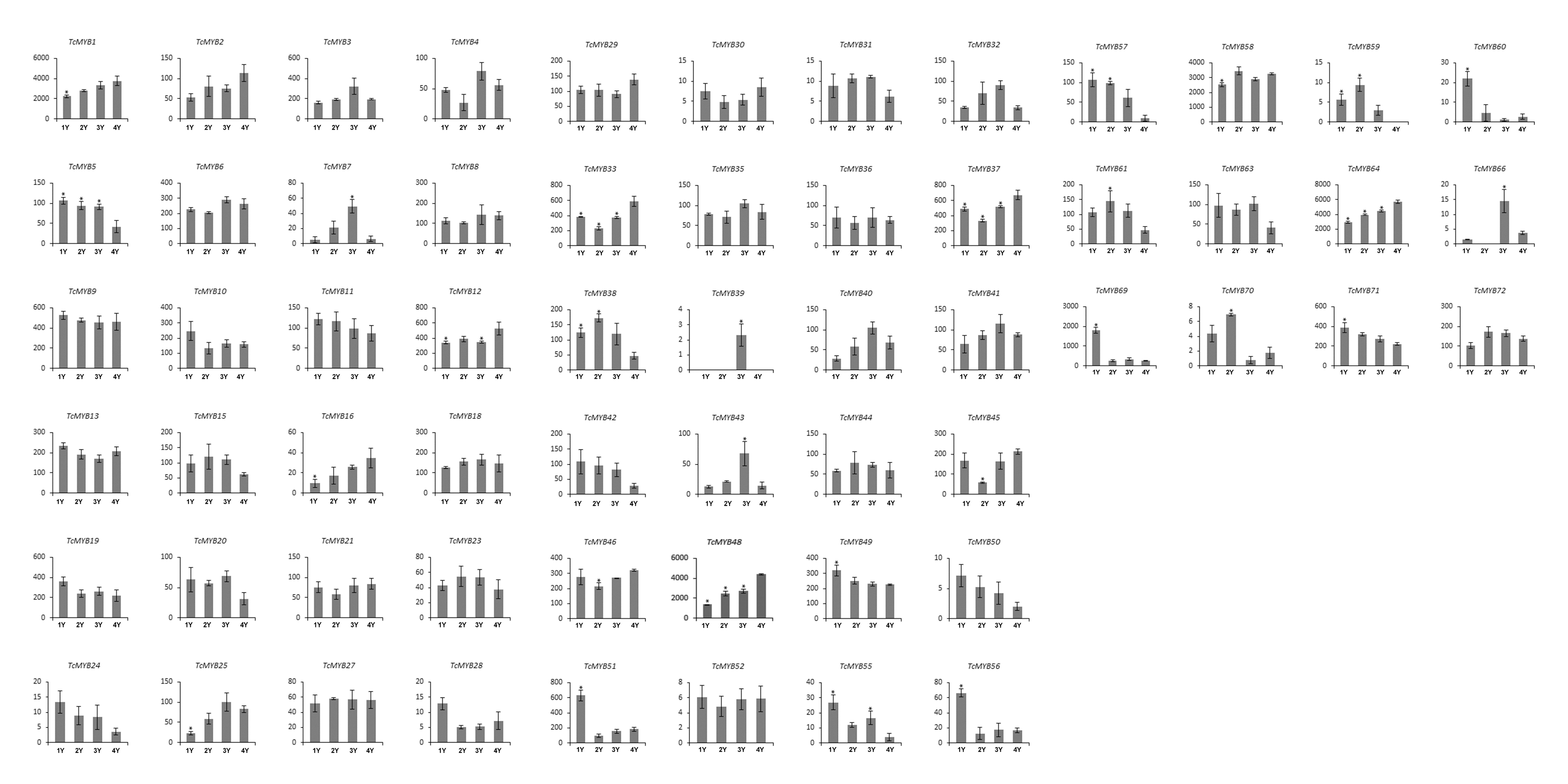

Supplement: Figure S2 — The relative expression of TcMYBs were compared to Tcactin *10000. [file peerj-08-8473-s005.png]

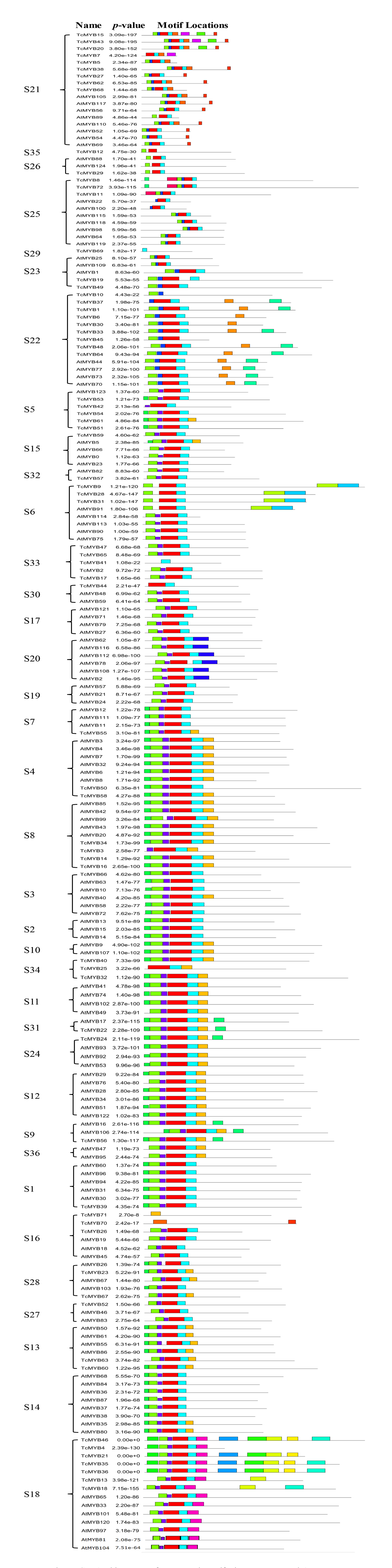

Supplement: Figure S3 — The motifs were identified by the MEME package. [file peerj-08-8473-s006.png]

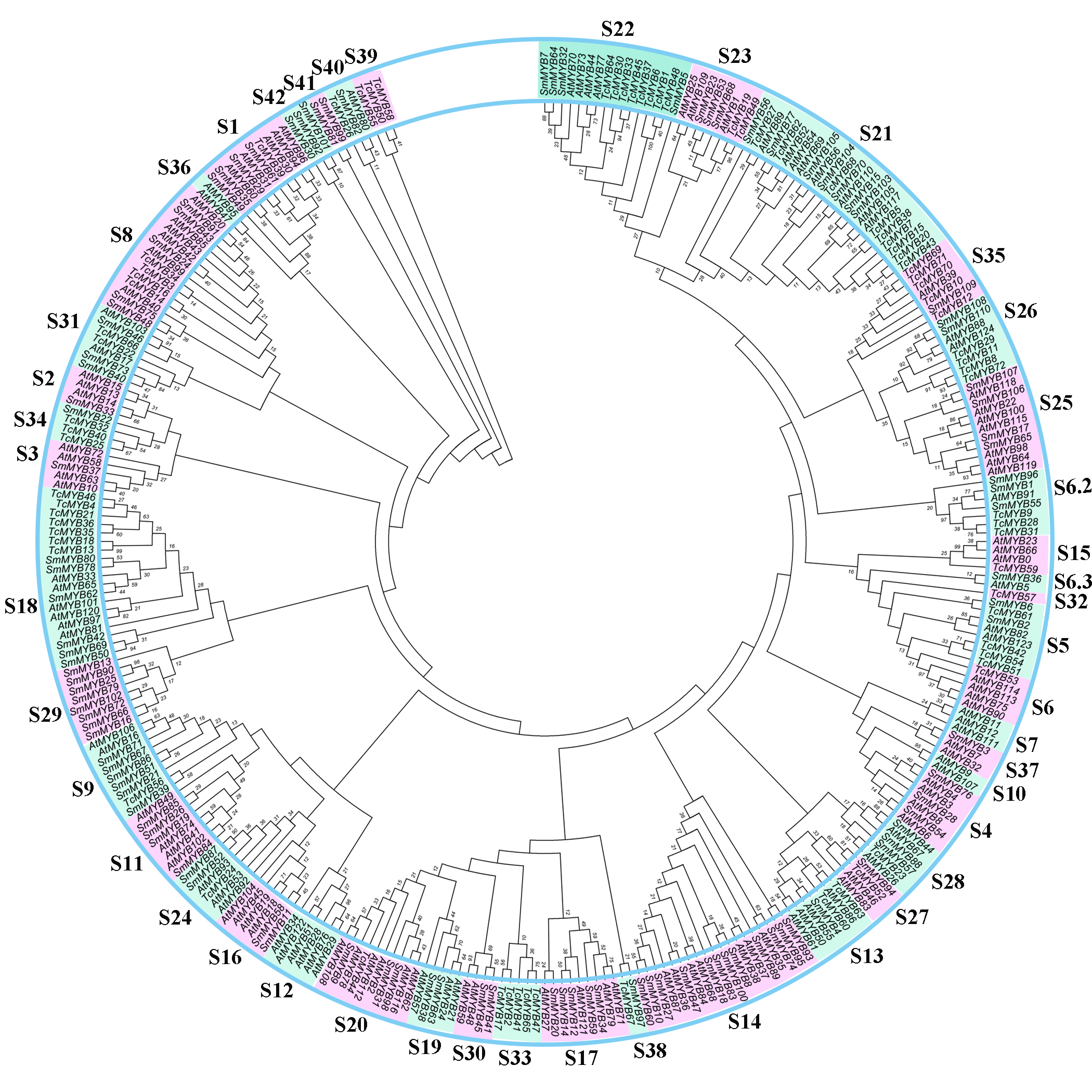

Supplement: Figure S4 — The phylogenetic relationships were constructed using MEGA 7.0 (100 bootstrap replicates, values <10 are not shown). [file peerj-08-8473-s007.png]
